# Nicotine and oxidative stress induced exomic variations are concordant and overrepresented in cancer-associated genes

**DOI:** 10.18632/oncotarget.2033

**Published:** 2014-05-28

**Authors:** Jasmin H. Bavarva, Hongseok Tae, Lauren McIver, Harold R. Garner

**Affiliations:** ^1^ Virginia Bioinformatics Institute, Virginia Tech, Blacksburg, VA, USA

**Keywords:** Nicotine, Exome sequencing, MUC4, Biomarker, Mutation targets

## Abstract

Although the connection between cancer and cigarette smoke is well established, nicotine is not characterized as a carcinogen. Here, we used exome sequencing to identify nicotine and oxidative stress-induced somatic mutations in normal human epithelial cells and its correlation with cancer. We identified over 6,400 SNVs, indels and microsatellites in each of the stress exposed cells relative to the control, of which, 2,159 were consistently observed at all nicotine doses. These included 429 nsSNVs including 158 novel and 79 cancer-associated. Over 80% of consistently nicotine induced variants overlap with variations detected in oxidative stressed cells, indicating that nicotine induced genomic alterations could be mediated through oxidative stress. Nicotine induced mutations were distributed across 1,585 genes, of which 49% were associated with cancer. MUC family genes were among the top mutated genes. Analysis of 591 lung carcinoma tumor exomes from The Cancer Genome Atlas (TCGA) revealed that 20% of non-small-cell lung cancer tumors in smokers have mutations in at least one of the MUC4, MUC6 or MUC12 genes in contrast to only 6% in non-smokers. These results indicate that nicotine induces genomic variations, promotes instability potentially mediated by oxidative stress, implicating nicotine in carcinogenesis, and establishes MUC genes as potential targets.

## INTRODUCTION

The increased incidence of cancer in the last 50-60 years may be largely attributed to two factors: the aging of the population, and the increased exposure to disease promoting agents present in general and occupational environments [1]. There are currently two opposite interpretations for this growing incidence of cancer. The first considers that environmental pollutants and chemicals can only make minor contributions to the overall cancer incidence and therefore increases in the size and aging of the population, and lifestyle influences such as smoking, alcohol consumption and diet can explain most of the increased cancer incidence [2]. Conversely, the second interpretation, citing that these arguments are not sufficient, estimates that in addition to these factors, there are contributions from the environment such as exposure to diverse chemical and biological agents, which may play a major role in the occurrence of the disease [3].

Nicotine is one of over 4,000 chemicals found in cigarette smoke. The connection between cancer and cigarette smoke is well established due to the presence of a number of carcinogenic substances in cigarette smoke [4]. However, nicotine is considered as an addictive substance in cigarette smoke, but not as a carcinogen. Because nicotine is not yet considered a carcinogen, it is increasingly being used as a therapeutic. The market for smoking cessation products that utilizes nicotine is growing rapidly and expected to reach $2.3 billion by 2016 in addition to nicotine consumption through tobacco [5]. Recently, the Food and Drug Administration (FDA) relaxed the restrictions on many nicotine products and removed the duration-of-use limits, which may signal to consumers that the consumption of these products is safe, even for extended periods (Section 918 Report to Congress, dated 22 April 2013, Department of Health and Human Services, FDA).

Microarray based studies have shown that a 1mM nicotine exposure can suppress immune response and modulate gene expression of immune system associated genes, including changes in NF-ĸB [6, 7]. Aberrant activation of NF-ĸB through oncogenic mutations in regulatory genes is associated with cancer [8]. Also, nicotine administration through dermal patches applied to mice has shown immunosuppressive and anti-inflammatory effects at nicotine concentrations lower than those used in experiments described herein [9]. In a 2007 study, in mice, prolonged nicotine exposure is reported to be genotoxic, particularly for bone marrow [10]. In contrast, a 1995 in-vitro assay based study conducted by the R.J. Reynolds Tabaco Company reported that nicotine and its major metabolites do not increase the frequency of mutations and are not genotoxic [11]. Recently, we have shown that nicotine could promote an environment for cancer genesis by modulating expression and splicing patterns of numerous genes [12].

Here, we explored and characterized in depth the genomic influence of nicotine and its genotoxic mechanism mediated through oxidative stress, using massively parallel sequencing in a controlled cell line experiment. This study suggests that nicotine exposure can adversely affect the human genome by inducing somatic mutations and over the period of significant exposure, may contribute to increased cancer incidence, characterizing nicotine as a carcinogen or mutagen. We further identified specific mutation targets that could be used for lung cancer diagnosis, prognosis and as an indicator for those exposed to nicotine. Importantly, results presented herein along with previous publications indicate that the recent action by the FDA to eliminate duration-of-use limits on nicotine products may need to be re-evaluated.

## RESULTS

### Genetic variations induced by nicotine stress

We targeted 201,071 exons (62.2 Mb target sequence) covering 20,794 genes in nicotine (0.5, 3 and 5mM) and hydrogen peroxide stressed normal breast epithelial cells, and sequenced them at high coverage (>50x average). 41 million 150bp reads on average were generated per sample. Exome enrichment efficiency was 98% (197,839 target exons were on average fully covered). This enabled 60.9 Mb of target sequences to be analyzed per sample for stress induced exomic changes, including single nucleotide, indel, and microsatellite variations.

The comparison of exomic changes indicated that all sequenced samples (control and experimental) exhibit between 10,000 and 10,700 non-synonymous single nucleotide variants (nsSNVs) with respect to the human genome reference, hg19. This is as expected because the 1000 genome project (1kGP) estimated that the typical exome differs from the reference human genome sequence at 10,000 to 11,000 non-synonymous sites [13]. This confirms that the samples in our study (control and experimental) were of good quality and the analysis criteria used were technically comparable. Further, by performing the initial experimental scans using this global microsatellite array that quantitates overall genome-wide microsatellite content changes it was possible to confirm biological and technical reproducibility. These experiments identified and confirmed overall comparable genomic changes in multiple independent experiments with nicotine and oxidative stress (Supplementary Fig. S1 and S2), thus providing confidence that the detailed sequencing experiment was being conducted on highly reproducible alterations induced by stress exposure.

Compared to the unexposed control, we identified a total of 6,506, 6,610, and 7,138 single nucleotide (SNVs), indel and microsatellite variations in 0.5, 3 and 5mM nicotine stressed cells, respectively. These included over 1,200 nsSNVs. In comparison, we identified 6,804 total variations in hydrogen peroxide (oxidative stress) stressed cells, which included 1,251 nsSNVs, of which 211 were cancer associated and 191 were predicted as functionally damaging by Polyphen (Table 1).

**Table 1:** Exomic variants in nicotine and hydrogen peroxide stressed cells compared to the untreated control

		Nicotine		H2O2
	0.5mM	3mM	5mM	
Total variants	6,506	6,610	7,138	6,804
All SNVs and indels	6,449	6,535	7,076	6,732
nonsynonymous SNVs	1,258	1,203	1,386	1,251
novel nonsynonymous SNVs	470	453	468	464
synonymous SNVs	885	954	1,095	921
stopgain SNVs	25	19	23	19
stoploss SNVs	0	1	3	1
Polyphen damaging	195	199	188	191
COSMIC	203	208	239	211
frameshift indels	21	24	28	23
All variable microsatellites	57	75	62	72
exomic	2	1	1	1
intronic	10	17	13	18
3' UTRs	31	41	31	35
5' UTRs	1	4	1	4
downstream	3	2	1	3
upstream	1	0	0	1
intergenic	9	10	15	10

To identify only variants consistently present in different nicotine experiments, we report herein only those variants that were observed in all three dose experiments. This resulted in to the identification of 2,159 variants consistently present in all three experiments compared to the unexposed control (Table 2). Of these 2,159, 429 were nsSNVs of which 158 were novel (not previously recorded in dbSNP 137). The COSMIC (Catalogue of Somatic Mutations in Cancer) database indicated 79 of the 2,159 mutations had an association with cancer. Polyphen predicted 59 of the nsSNVs to be functionally damaging (Table 2).

**Table 2:** Genetic variations found to be concordant among treated cells as compared to untreated cells

	All nicotine vs control	All nicotine and H2O2 vs control
All SNVs and indels	2,159	1,739 (81%)
nonsynonymous SNVs	429	361 (84%)
novel nonsynonymous SNVs	158	139 (88%)
synonymous SNVs	339	292 (86%)
stopgain SNVs	8	8 (100%)
Polyphen Damaging	59	50 (85%)
COSMIC	79	65 (82%)
Frameshift indels	6	4 (67%)
Microsatellites	8	8 (100%)

Overlapping variations detected in all doses of nicotine and oxidative stress. Numbers in the parenthesis indicates the percentages of nicotine-induced mutations that were consistently overlapping with those induced by oxidative stress.

Nicotine exposure showed a slight dose-dependent effect as indicated by total number of variants detected in three nicotine dose exposures. However, the number of variants at the lowest dose were significant in number, indicating that there exists a possible threshold for genomic destabilization and nicotine could be genotoxic at less than LD_5_ (0.5mM) dose even though it may not be inducing cell death. Note, the systemic nicotine level in smokers is reported to be up to 444nM following smoking [14]. Its daytime average is 99nM and 154nM in blood while undertaking transdermal nicotine and nasal spray as a therapy, respectively [15]. The actual concentration of nicotine from transdermal patches at the skin can be high, equivalent to 5.1mg/cm^2^ [16]. Optical absorbance measurements indicate the nicotine concentration at skin contact to be greater than 1mM, in agreement the concentration found 1 mm below the skin surface where it enters blood vessels [17, 18], thus confirming the range of doses used in these experiments are physiologically appropriate.

Overall, 57 to 75 microsatellite loci varied in all stressed cell cultures compared to the control (Table 1, Supplementary Table S1). Of variable microsatellites, eight were consistently detected in all three nicotine exposure doses. These eight variable microsatellites were also consistent with that of oxidative stressed exome. They were distributed as follows: exon (1), intron (1), 3'UTR (5), and intergenic (1). The exomic microsatellite repeat variation was found in FAM157B, which is a large gene with unknown function. PRELP, SGPL1, IGJ, HIATL1, and MIER1 acquired repeat variations in 3' untranslated regions (3' UTRs). 3' UTRs often contain several regulatory elements that govern the spatial and temporal expression of mRNA [19]. Although, these are relatively understudied genes, PRELP and MIER1, both are associated with leukemia [20, 21].

Nicotine induced somatic mutations (SNVs, indels and microsatellites) were distributed in 1,585 genes (Supplementary Table S2). Out of the 1,585 genes, 301 harbored more than one mutation and four of them contained more than 10 mutations. Of particular note, several members of the mucin (MUC) family of genes harbored numerous variations in all samples. Gene expression alterations in mucin family genes accompany the development of cancer [22]. Mucins are used as diagnostic markers in cancer, and are under investigation as therapeutic targets for cancer [22]. At 5mM nicotine exposure, MUC4, MUC12, and MUC6 harbored 116, 52, and 25 variations, respectively. MUC4, in particular, was a consistently the top mutated gene upon stress exposures. It had 99, 110, and 116 variations upon 0.5, 3, and 5mM nicotine exposure, respectively and 115 variations upon oxidative stress.

### Biological implications and potential mechanisms

**Figure 1:**
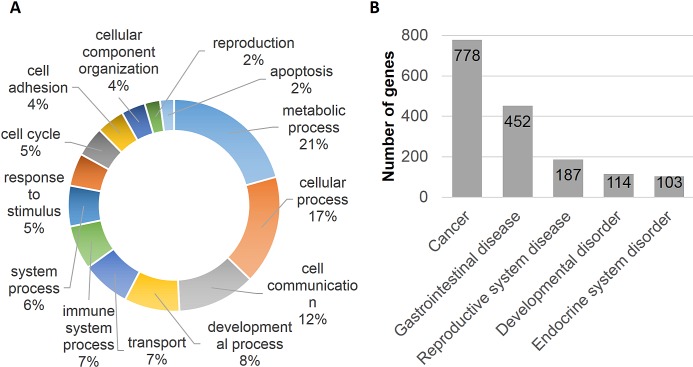
Gene Ontology and disease and disorder enrichment analysis (A) Ontology analysis of genes harboring variations (SNVs, indels and microsatellites) reveals a number of biological processes that are enriched. (B) Ingenuity Pathway Analysis (IPA) on this group of genes (n=1,585) indicates a statistically significant association with a number of diseases. It further illustrates the overrepresentation of cancer (49%) and gastrointestinal disease associated genes (29%).

**Figure 2:**
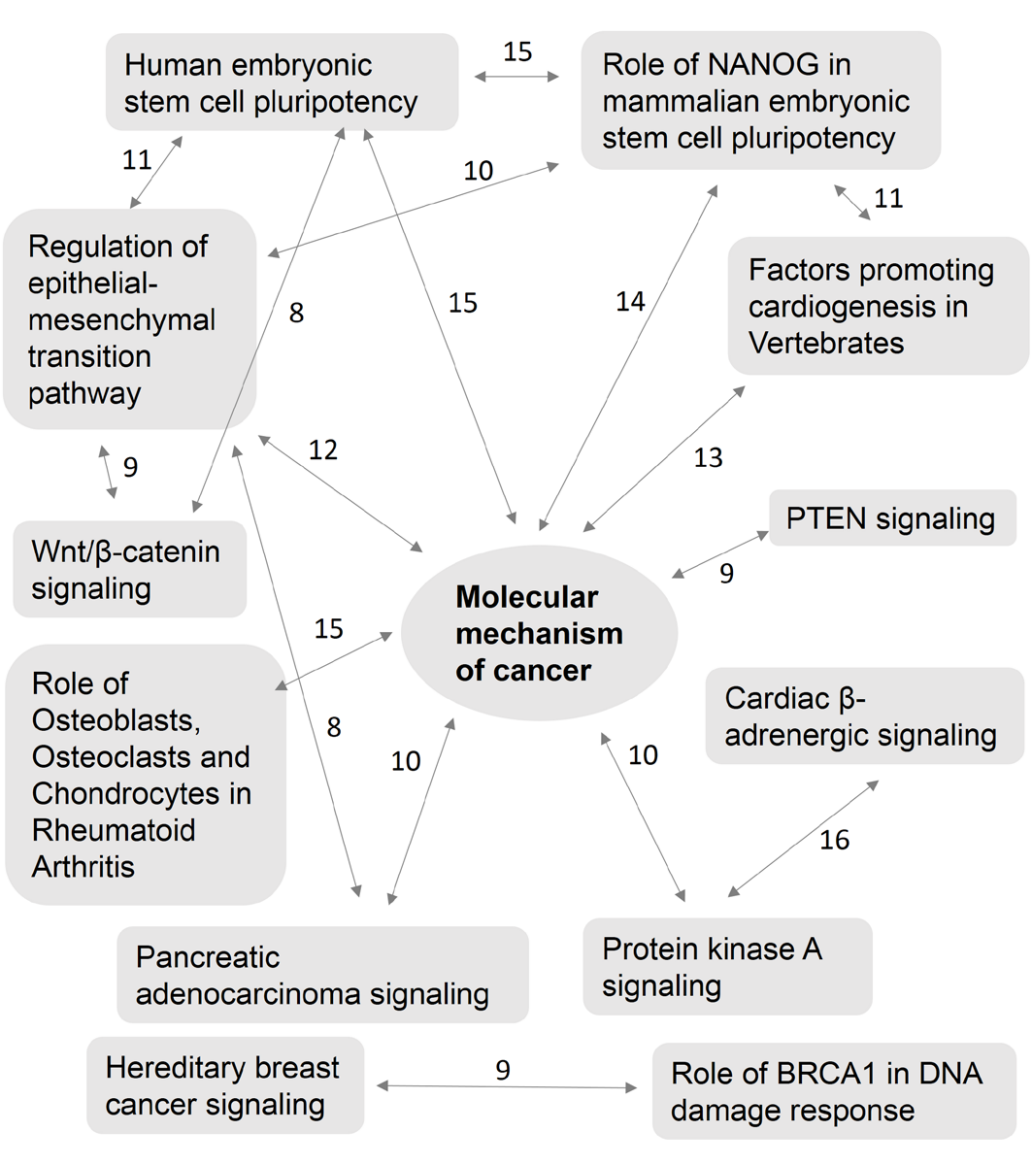
Complex interaction of multiple canonical pathways and their common genes Ingenuity pathway analysis of 1,585 affected genes revealed enrichment of canonical pathways associated with cancer including a number of genes playing a role in multiple canonical pathways indicating complex biological interactions. Numbers above lines indicate genes common in both canonical pathways.

Polyphen and COSMIC analysis of nicotine stress-induced somatic mutations revealed a number of possible biological implications. We performed gene ontology enrichment analysis of the 1,585 genes mutated. The PANTHER classification system identified 1,433 genes, of which 21% of the genes were associated with metabolic and 17% genes were associated with cellular processes (Fig. 1A). We imported these 1,585 genes into the Ingenuity Pathway Analysis (IPA) suite to further investigate gene-gene interactions, diseases and pathway associations. IPA identified cancer and gastrointestinal disease associations with statistical significance (p <0.05) because of the large number of mutated genes associated with these diseases (779 and 452, respectively) (Fig. 1B). It also confirmed the statistically significant overabundance of genes associated with cancer. Canonical pathway enrichment analysis in IPA revealed strong association of these genes with cancer associated canonical pathways. These included, “molecular mechanism of cancer, PTEN signaling, Wnt/β-catenin signaling, pancreatic adenocarcinoma signaling and hereditary breast cancer signaling” (p<0.05). A number of genes were also associated with multiple cancer associated canonical pathways revealing potentially complex biological interactions and implications (Fig. 2). “Molecular mechanism of cancer”, a canonical pathway, shows involvement of 35 mutated genes (Supplementary Fig. S3).

Increased oxidative stress is a common feature observed in different types of tumors. Comparing doses with equivalent impact, LD_50_ (5mM and 4mM doses of nicotine and hydrogen peroxide, respectively), we found that 44% of the somatic mutations were overlapping. However, of the consistently induced variants at all nicotine doses, 81% of the SNVs and indels and 100% of the microsatellite variations were concordant with those observed in the oxidative stress induced samples (Table 2). These mutations were distributed in 1,320 unique genes. Further, we performed pathway and disease association analysis of genes that were mutated upon oxidative stress (n=3,760), which confirmed the highest association to be with cancer (n=1,763) and gastrointestinal disease (n=1,070). This indicates that nicotine induced genomic changes are potentially mediated by oxidative stress.

### MUC4, MUC6, and MUC12 mutations in the lung cancer

To identify a possible association of MUC4, MUC6, and MUC12 with lung cancer, we analyzed the publicly available The Cancer Genome Atlas (TCGA) exome sequence data for lung adenocarcinoma and lung squamous cell carcinoma. We found that 18% (66 of 360) lung adenocarcinoma tumor samples in smokers had mutations in at least one of these three MUC genes (p-value ≤0.03), in contrast to just 7% (4 of 58) from non-smokers. Similarly, 23% (38 of 167) squamous cell carcinoma tumor samples from smokers had mutations in at least one of these three MUC genes, whereas no mutations (0 of 6) were detected in any of three MUC genes in non-smokers (p-value ≤0.3). Overall, 20% of all non-small-cell lung carcinoma (NSCLC) tumors in smokers had mutations in at least one of three MUC genes in contrast to only 6% in non-smokers (p-value ≤0.006) (Supplementary Table S3). Although there was no correlation between mutation status of these genes with that of the tumor stage, the significant correlation of mutation status with the smoking history in patients reveals a strong potential to exploit these genes in clinical settings.

## DISCUSSION

Nicotine exposure is pervasive through the use of tobacco and tobacco cessation therapeutics. Here we provide evidence that nicotine is a carcinogenic/mutagenic substance in addition to an addictive one.

**Figure 3:**
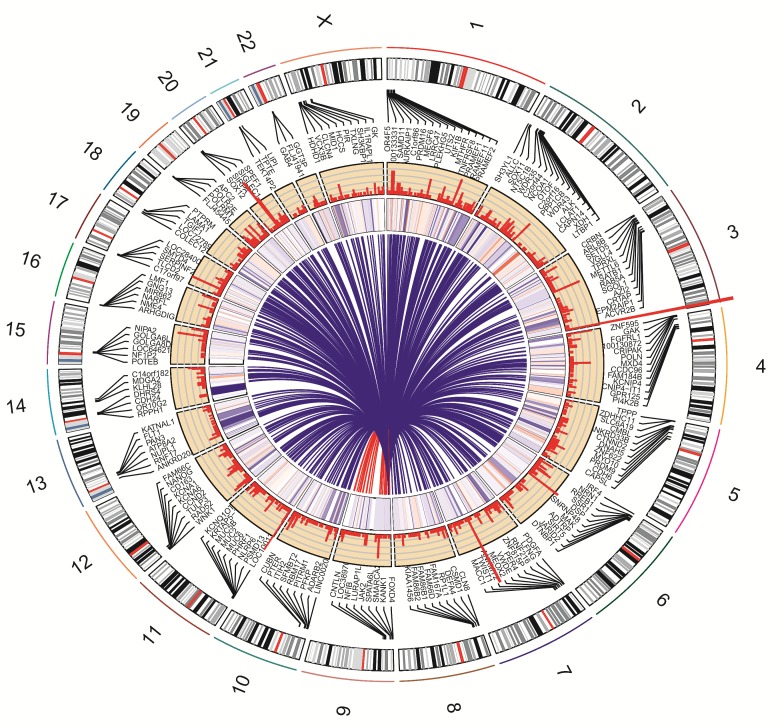
Circos plot depicting the influence of nicotine exposure in normal epithelial cells Circos plot presents a global view of nicotine-induced somatic mutations detected by exome sequencing in this study (Histogram plot in second inner track) with gene expression data derived from the transcriptome sequencing study (Heatmap plot in first inner track). Height of histogram bars illustrates the number of mutations in genes. Heatmap color represents gene expression: Blue (Negative fold change, maximum −2.7) and Red (Positive fold change, maximum 4.7). Link line plot (center) indicates genes that are associated with cancer. Representative genes were noted randomly due to space constraints. The human chromosome ideogram table used was from the UCSC genome browser.

The genome-wide view of nicotine-induced somatic mutations, gene expression changes and mutated cancer associated genes is concisely presented in the Circos plot (Fig. 3). In the recent transcriptome sequencing study, we demonstrated that nicotine exposure differentially regulates 2015 genes and resulted in alternative splicing of 173 genes [12]. We observed that 138 of these differentially expressed and 11 of the alternatively spliced genes acquired mutations upon nicotine exposure as detected in the present study. Both studies identify statistically significant cancer-associated genes differentially expressed and/or mutated upon nicotine exposure. However, none of the mutated MUC genes were reported as differentially expressed in transcriptome study indicating that expression level changes of the altered MUC transcripts (and presumably proteins) may not be associated with nicotine or oxidative stress. Note that these studies utilize the identical model cell line, nicotine concentration and experimental control, which makes results uniquely comparable as outlined previously [23].

We also analyzed microsatellite variations since microsatellites are known to have a role in faster adaptation to environmental stresses [24]. Microsatellites are among the most variable types of DNA sequence in the genome and represents ~3% of the genome, which is twice the coding region [25]. Under nicotine and oxidative stress, approximately 50-70 (0.3%) of the microsatellite loci varied, and were independent of dose. In comparison, we observed on average ~6,000 SNPs in the 62 Mbases sequenced in each exome, or about 0.01% of the bases varied. Thus, in this study, microsatellite loci were ~30 times more mutable than single nucleotide polymorphisms, consistent with previous reports of elevated microsatellite mutability. Thus, microsatellite variability may be a more sensitive measure of the genomic response to cell stress.

Nicotine-induced somatic mutations were concordant with those induced by oxidative stress. Nicotine has been previously reported to induce oxidative stress in cultured cells [26]. Further, cells in tissues and organs are continuously subjected to oxidative stress and free radicals, which may be of exogenous or endogenous (intracellular) origin. The cells withstand these processes via several different defense mechanisms; ranging from free radical scavengers (glutathione (GSH), vitamins C and E and antioxidant enzymes like catalase, superoxide dismutase and various peroxidases) to sophisticated and elaborate DNA repair mechanisms such as base excision repair [27]. Therefore, the intensity of nicotine stress induced genomic damage on an individual basis varies depending on the dynamic equilibrium of the above factors and may be greatly reduced with a healthy lifestyle and better food consumption habits. This may also partially explain why individual smoker's susceptibility of cancer may vary.

Previous studies have demonstrated that nicotine and its metabolites bind to nAchR subunits, which may mediate the carcinogenic effects [28]. It has been suggested that nicotine could cause cell proliferation through Ras-Raf-MEK-ERK signaling pathway [29]. However, at this point, it is unclear whether nicotine exerts its mutagenic effect either through activating downstream signaling pathways or its conversion to a carcinogenic substance. Further, carcinogenic substances often induce transversions (Conversion of G to T). In our study, 5% of the variations measured were transversions (104 out of 2,158 mutations), which suggests this possible carcinogenic characteristic of nicotine.

We subjected cells to a single pulse of nicotine at doses up to LD_50_ to document the extreme of physiological and genotoxic effects. However, long-term exposures (ranging from months to years) at lower concentrations, identical to those found in the plasma after smoking, may be warranted to evaluate the consequences of sustained nicotine consumption. These higher doses, applied as a time pulse, more resembles the local dose experienced by those cells in direct contact with the nicotine patch or spray. Additional studies that better emulate the time course for an average nicotine cessation program should be conducted, as would studies on other epithelial cell lines to characterize long-term effect of nicotine cessation therapy that includes nicotine patches, nasal sprays and “vapor” cigarettes. In addition, epidemiological studies involving analysis of a substantial number of human genomes are warranted to uncover the biological impact of continued nicotine consumption on human health.

A key area of cancer research is to identify and investigate genetic and epigenetic alterations occurring during cancer development that may serve as clinical tools for disease diagnosis and prognosis. We identified 79 consistently occurring mutations that are cancer associated per the COSMIC database. Additionally, exposure induced 429 nsSNVs that may have functional significance. Together, these suggest that nicotine exposure results in many reproducible genetic variations that drive cells towards the cancer state. Of particular note, we observed frequent mutations in a number of MUC family genes, in particular MUC4. Previous studies have associated differential MUC4 expression with a number of cancers, including pancreatic, lung, breast, gall bladder, salivary gland, prostate and ovarian cancer, indicating that MUC4 may be a good candidate as a diagnostic and prognostic marker [30]. For example, in one breast cancer study, silencing MUC4 led to reduced expression of HER2, although the molecular mechanism of this interaction is unknown [31]. Over-expression of HER2 occurs in 30% of breast cancers and has been used effectively as an adjuvant therapy drug target in these patients [32].

MUC4 exhibits a pattern of positive selection under nicotine and oxidative stress as indicated by the positive ratio of nonsynonymous SNVs/ synonymous SNVs (1.7 to 2.3 in all experimental samples). Stress-induced selection pressure on genes is reported to play an important role in evolution [33, 34]. However, the functional significance of positive selection of MUC4 upon nicotine or oxidative stress is unclear at this time.

Lung cancer is the most common cause of cancer related death [35], which is frequently caused by long-term exposure to tobacco smoke [36]. We correlated the mutation frequency of MUC4, MUC6, and MUC12 with the non-small-cell lung carcinoma, and identified a distinct mutation frequency in tumor samples from smokers (20%) and non-smokers (6%), which cumulatively designates these MUC genes as diagnostic and prognostic markers in smokers. It is interesting to note here that MUC genes were not previously reported as the most frequently mutated genes in lung cancer [37]. Because large genome based population scale studies are dominated by frequently mutated genes and their ranked correlation with clinical metadata, it is possible to overlook the impact of an individual gene or a family of genes and their experimental validations. It would be interesting to explore mutation frequencies of MUC genes in germline samples of lung cancer patients that may pre-dispose them to cancer. Further, the study of MUC gene alterations may be warranted in recurrent tumors that were previously exposed to chemotherapy and radiation since both of these would be inducing extreme stress at the cellular level.

In summary, this study utilized an unbiased next-generation sequencing approach to investigate somatic exomic variants induced in response to exposure of nicotine and oxidative stress. It reveals that nicotine exposure causes somatic mutations, which are substantially concordant with those induced from oxidative stress and implicates nicotine in carcinogenesis/mutagenesis. Further, we identified MUC4, MUC6, and MUC12 as consistent mutation target genes for nicotine and oxidative stress. We discovered that 14% of the non-small-cell lung cancer tumors in smokers have mutations in at least one of three MUC genes establishing MUC family genes as strong genetic marker for nicotine stress in smokers and for diagnosis and prognosis in the lung cancer.

## MATERIALS AND METHODS

### Reagents, chemicals, and cell culture

MCF-10A cells were obtained from American type culture collection (ATCC). Cells were cultured in DMEM/F12 medium (Invitrogen), supplemented with horse serum (5% final, Invitrogen), Pen/Strep (1% final, Invitrogen), EGF (20ng/ml final, Peprotech), hydrocortisone (0.5mg/ml final, Sigma), cholera toxin (100ng/ml final, Sigma), and insulin (10ug/ml final, Sigma) at 37°C in a humidified atmosphere containing 5% carbon dioxide. Nicotine was purchased from Sigma (St. Louis, MO, U.S.A.).

### Experiments

Twenty-four hours before application of nicotine and hydrogen peroxide, cells were seeded at a density of approximately 3x105 cells/well in 6-well plates or 5x107/ 500cm^2^ cell culture dishes. Nicotine was diluted in complete culture media at required final concentrations. Dose ranging experiments were carried out in six well plates. Nicotine was applied for a range of doses on cells for 72hrs and at the end of the exposure period, the number of live cells were measured with a cell counter (Biorad). We used 5mM (~LD_50_), 3mM (~LD_25_) and 0.5mM (~LD_5_) doses of nicotine and 4mM (~LD_50_) for hydrogen peroxide (H2O2). Dose for nicotine experiments were within the range of previously reported studies [7, 12]. All isolated DNAs were tested for quality and DNA samples with 260/280 ratio over 1.8 were used for exome sequencing.

### Global Microsatellite Content quantitation array design, manufacturing, processing, and analysis

Each array consists of 41,430 unique repeat probes, each replicated 3-5 times at different positions across the array, for 125,300 probes (features), from which data were obtained. The design included probes to measure all possible cyclic permutations of repeat units from 1-mer to 6-mer, and a variety of controls. Additionally, 7-mer probes were included though this set is not a complete set of all possible cyclic permutations due to array size constraints. All arrays were manufactured by Roche Nimblegen following their standard production methods for maskless photolithography. All DNA test samples were labeled, hybridized to array and scanned in pairs, always including one standard. The data extraction was performed by Roche Nimblegen's standard protocol for aCGH arrays. Array data analysis of the raw hybridization intensities was performed locally. Briefly, a custom pearl script was used to calculate a z-score for each motif family, including replicates and cyclic permutations, followed by the calculation of average and standard errors for each motif family using all replicates and cyclic permutations that a pass z-score cutoff (1.64) for significance. Processed data for each array represented 3,304 unique microsatellites motif families and their intensity values, which themselves were proportional to the global microsatellite content in a given genome. Further, data were log transformed and mean normalized. Then, experimental samples were compared to their respective controls to determine global microsatellites content changes. The data processing, analysis, and hirechial clustering was done using Gene Spring v. 11.5 data analysis software (Agilent). Commercially available Promega human female DNA was used as a control to gauge reproducibility of this array. All motifs continuously monitored in the control DNA confirmed the array reproducibly (R^2^ ≥0.99) when samples were run on three different arrays and compared. Additionally, we have previously demonstrated array specificity and sensitivity by demonstrating the ability of the array to detect Epstein-Barr virus (EBV) transformation within cell line samples by detecting EBV's singular and specific microsatellite motif/locus GAGCAG [38]. Together, these confirm very high confidence in the sensitively and reproducibly of array experiments.

### Exome capture and sequencing

DNA libraries were constructed using Illumina's TruSeq® DNA Sample Preparation Kit-Set A/B (P/N FC-121-2001/2002). Briefly, 1.5μg DNA was fragmented using a Covaris M220 to 400bp. A gel-free method recommended in the protocol was used to prepare the library. The ends were repaired and an ‘A’ base was added to the 3' end, which prepares the DNA fragments for ligation to the adapters that have a single ‘T’ base overhang at their 3' end. The adapters enable PCR amplification and hybridization to the flow cell. The library generated was validated using Agilent 2100 Bioanalyzer and quantitated using Quant-iT dsDNA HS Kit (Invitrogen; Carlsbad, CA). Exome enrichment was performed using a TruSeq® Exome Enrichment Kit (FC-121-1024; Illumina). Samples were pooled (500ng each) and enriched following the manufacturer's standard protocol. Enriched samples were quantitated based on Quant-iT dsDNA HS Kit (Invitrogen) and qPCR.

Libraries were clustered onto a flow cell using TruSeq® Rapid PE Cluster Kit – HS (PE-402-4001), and sequenced for 150 cycles pair-end using TruSeq® Rapid SBS Kits – HS (FC-402-4001) on HiSeq 2500®. Reads that passed the Illumina chastity filter were kept. Reads passed the chastity filter if they had, within the first 25 cycles, no more than one cycle of a chastity below 0.6 (Chastity = Highest intensity/(Highest intensity + Next highest intensity)). An average of 41.4 million high quality 150bp reads (passed Chastity filter) were generated from exome-enriched samples equivalent to 6.2 billion DNA bases per exome. We opted for longer (400bp) DNA fragments for library preparation and longer read length (150bp) for sequencing to enhance the quality and results, especially within repeat regions.

### SNVs, indels, and microsatellite calling from exome sequencing data

We aligned sequence reads to the human genome reference, hg19, using BWA and obtained an average sequence coverage of 50.7x per sample on targeted exomic regions. Reads were locally realigned around Indels, and raw variants (Single nucleotide variations and Indels) were called using GATK Unified Genotyper [39, 40]. We filtered variants with a minimum read depth of ≥5x and mapping quality >30 as a final acceptable variant call. This method has shown >90% of true positives in other studies [41]. We used microsatellite specific genotyping software that requires a minimum of 15 reads completely spanning a locus in order to call the genotype for each sample [42, 43]. This method has shown to have a 95% accuracy. This analysis enabled the calling of on average 22 820 microsatellite loci from each exome-sequenced sample.

### Gene annotation, enrichment, and functional impact analysis

All identified variants (single nucleotide variations and indels) were annotated using ANNOVAR package [44]. Splice site variations were identified as occurring within two base pairs of any intron/exon boundary. Variants that created a stop codon at a variant site were considered as stop-gain variants. Variants that eliminated stop codon at the variant site were considered as stop-loss variants. All identified variations were annotated for a variety of characteristics and analyzed. The Single Nucleotide Polymorphism database (dbSNP 137) was used to check for novel variants. Polyphen 2.0 was used to predict the functional impact of non-synonymous variations [45] (We considered high confident predictions- variations that were identified by Polyphen as “Possible damaging”); The Catalogue of Somatic Mutations in Cancer (COSMIC) database v64 was used to identify somatic cancer variants [46]. The PANTHER classification system was used for gene ontology enrichment analysis [47]. Gene network and pathway analysis was done using Ingenuity Pathway Analysis (IPA). Circos plot was generated with the R statistical software using RCircos package [48]

### Analysis of lung cancer data from The Cancer Genome Atlas (TCGA) dataset

As of 1 November 2013, The Cancer Genome Atlas (TCGA) contained exome sequence data for 499 Lung adenocarcinoma (LUAD) samples and 493 Lung squamous cell carcinoma (LUSC) tumor samples. These are subtypes of non-small cell lung cancer, one of the most common types of lung cancer. We downloaded the clinical metadata and somatic mutations for the LUAD and LUSC sets from TCGA Data Portal (https://tcga-data.nci.nih.gov/tcga/). The somatic mutation file only listed the mutations contained in the tumor samples. Using custom perl scripts, we analyzed the data for each tumor sample and correlated metadata (individual smoking history) and mutations (including MUC4, MUC6, and MUC12). We only considered the 591 samples (418 LUAD and 173 LUSC) for which smoking history was provided and there was at least one mutation identified in the tumor sample. This allowed us to ensure that all samples included in the analysis were also included in the mutation calls provided by TCGA. Further, we grouped these samples according to the pathological stage reported as metadata to correlate tumor grade with the mutation status of MUC genes. R statistical software was used to compute p-values with the fisher.test function for a two-by-two matrix set with the alternative hypothesis as “two.sided”.

## SUPPLEMENTARY MATERIAL FIGURES AND TABLES




