# Supplementary material for: Nicotine and oxidative stress induced exomic variations are concordant and overrepresented in cancer-associated genes
Source: Oncotarget. 2014 May 28;5(13):4788–98. doi: 10.18632/oncotarget.2033 (PMC4148099; doi:10.18632/oncotarget.2033)
Supplement: Supplementary file 1 [file oncotarget-05-4788-s001.pdf]

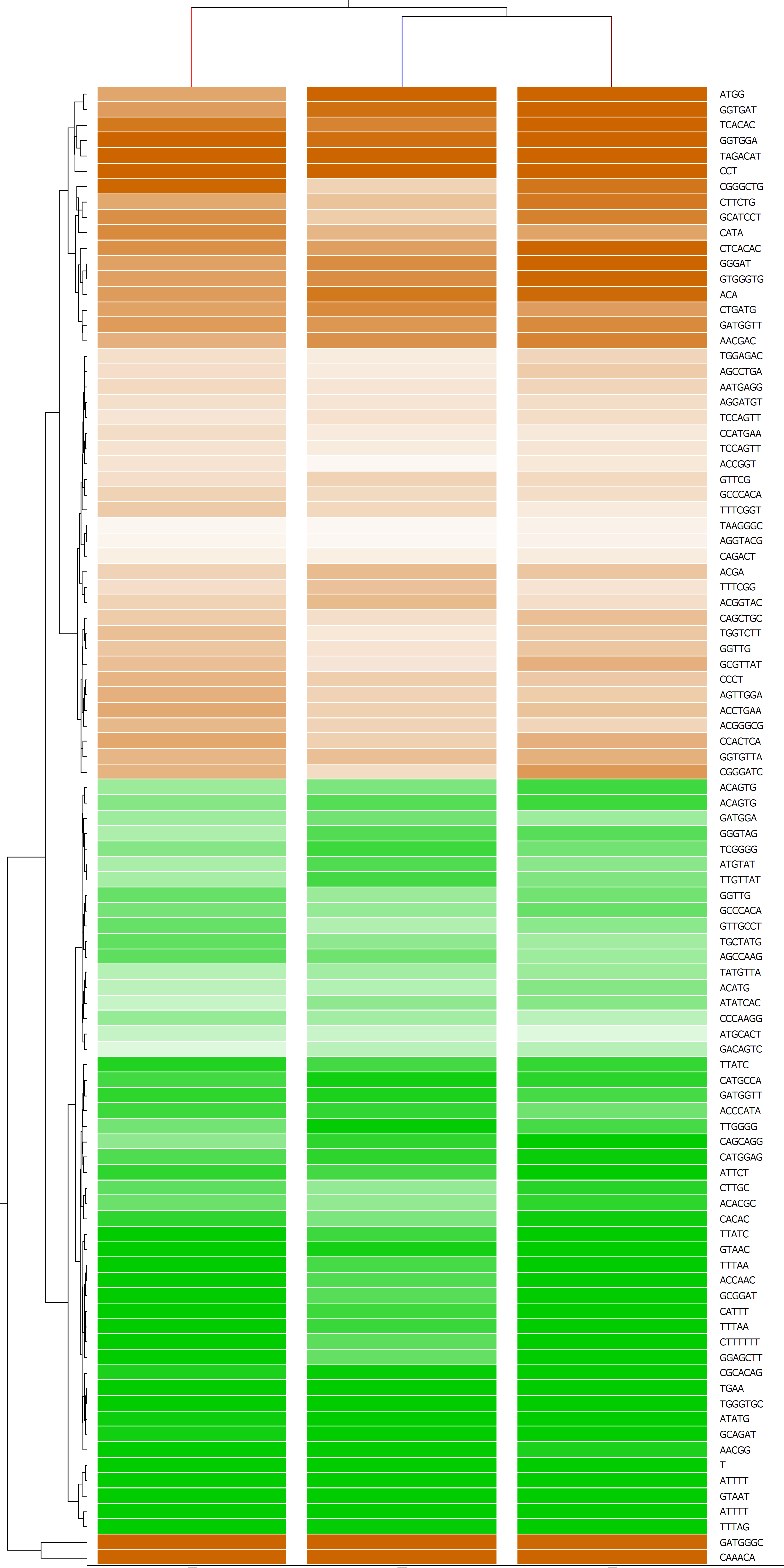
ATGG
GGTGAT
TCACAC
GGTGGA
TAGACAT
CCT
CGGGCTG
CTTCTG
GCATCCT
CATA
CTCACAC
GGGAT
GTGGGTG
ACA
CTGATG
GATGGTT
AACGAC
TGGAGAC
AGCCTGA
AATGAGG
AGGATGT
TCCAGTT
CCATGAA
TCCAGTT
ACCGGT
GTTCG
GCCCACA
TTTCGGT
TAAGGGC
AGGTACG
CAGACT
ACGA
TTTCGG
ACGGTAC
CAGCTGC
TGGTCTT
GGTTG
GCGTTAT
CCCT
AGTTGGA
ACCTGAA
ACGGGCG
CCACTCA
GGTGTTA
CGGGATC
ACAGTG
ACAGTG
GATGGA
GGGTAG
TCGGGG
ATGTAT
TTGTTAT
GGTTG
GCCCACA
GTTGCCT
TGCTATG
AGCCAAG
TATGTTA
ACATG
ATATCAC
CCCAAGG
ATGCACT
GACAGTC
TTATC
CATGCCA
GATGGTT
ACCCATA
TTGGGG
CAGCAGG
CATGGAG
ATTCT
CTTGC
ACACGC
CACAC
TTATC
GTAAC
TTTAA
ACCAAC
GCGGAT
CATTT
TTTAA
CTTTTTT
GGAGCTT
CGCACAG
TGAA
TGGGTGC
ATATG
GCAGAT
AACGG
T
ATTTT
GTAAT
ATTTT
TTTAG
GATGGGC
CAAACA

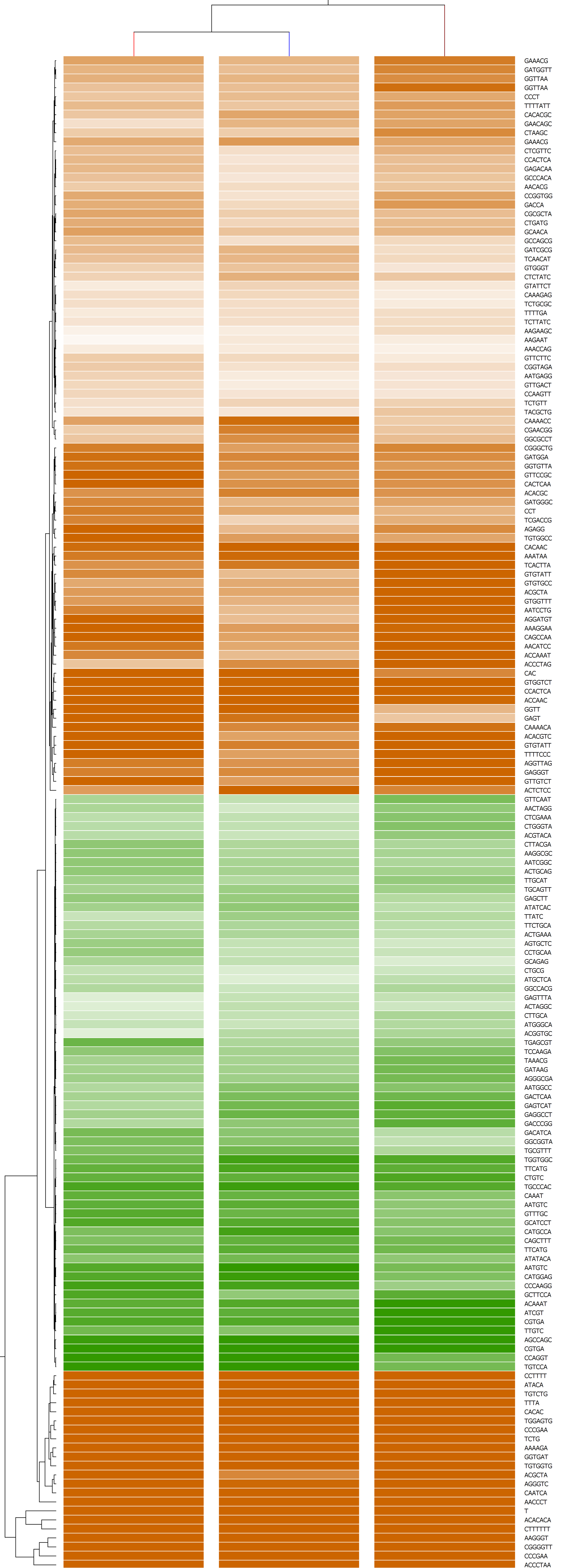

GAAACG
GATGGTT
GGTTAA
GGTTAA
CCCT
TTTTATT
CACACGC
GAACAGC
CTAAGC
GAAACG
CTCGTTC
CCACTCA
GAGACAA
GCCCACA
AACACG
CCGGTGG
GACCA
CGCGCTA
CTGATG
GCAACA
GCCAGCG
GATCGCG
TCAACAT
GTGGGT
CTCTATC
GTATTCT
CAAAGAG
TCTGCGC
TTTTGA
TCTTATC
AAGAAGC
AAGAAT
AAACCAG
GTTCTTC
CGGTAGA
AATGAGG
GTTGACT
CCAAGTT
TCTGTT
TACGCTG
CAAAACC
CGAACGG
GGCGCCT
CGGGCTG
GATGGA
GGTGTTA
GTTCCGC
CACTCAA
ACACGC
GATGGGC
CCT
TCGACCG
AGAGG
TGTGGCC
CACAAC
AAATAA
TCACTTA
GTGTATT
GTGTGCC
ACGCTA
GTGGTTT
AATCCTG
AGGATGT
AAAGGAA
CAGCCAA
AACATCC
ACCAAAT
ACCCTAG
CAC
GTGGTCT
CCACTCA
ACCAAC
GGTT
GAGT
CAAAACA
ACACGTC
GTGTATT
TTTTCCC
AGGTTAG
GAGGGT
GTTGTCT
ACTCTCC
GTTCAAT
AACTAGG
CTCGAAA
CTGGGTA
ACGTACA
CTTACGA
AAGGCGC
AATCGGC
ACTGCAG
TTGCAT
TGCAGTT
GAGCTT
ATATCAC
TTATC
TTCTGCA
ACTGAAA
AGTGCTC
CCTGCAA
GCAGAG
CTGCG
ATGCTCA
GGCCACG
GAGTTTA
ACTAGGC
CTTGCA
ATGGGCA
ACGGTGC
TGAGCGT
TCCAAGA
TAAACG
GATAAG
AGGGCGA
AATGGCC
GACTCAA
GAGTCAT
GAGGCCT
GACCCGG
GACATCA
GGCGGTA
TGCGTTT
TGGTGGC
TTCATG
CTGTC
TGCCCAC
CAAAT
AATGTC
GTTTGC
GCATCCT
CATGCCA
CAGCTTT
TTCATG
ATATACA
AATGTC
CATGGAG
CCCAAGG
GCTTCCA
ACAAAT
ATCGT
CGTGA
TTGTC
AGCCAGC
CGTGA
CCAGGT
TGTCCA
CCTTTT
ATACA
TGTCTG
TTTA
CACAC
TGGAGTG
CCCGAA
TCTG
AAAAGA
GGTGAT
TGTGGTG
ACGCTA
AGGGTC
CAATCA
AACCCT
T
ACACACA
CTTTTTT
AAGGGT
CGGGGTT
CCCGAA
ACCCTAA

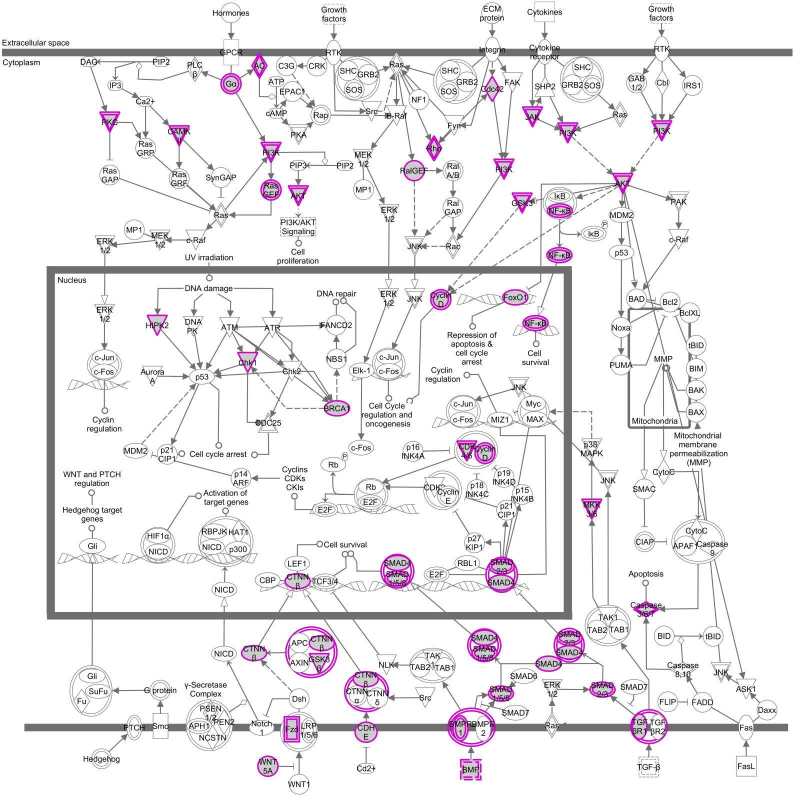

Hormones
Growth factors
ECM protein
Cytokines
Growth factors
Extracellular space
Cytoplasm
GPCR
RTK
Integrin
Cytokine receptor
RTK
DAG
PIP2
PLC β
Gα
AC
C3G
CRK
SHC
GRB2
SOS
Ras
SHC
GRB2
SOS
Cdc42
FAK
SHP2
SHC
GRB2
SOS
GAB 1/2
Cbl
IRS1
IP3
Ca2+
ATP
EPAC1
cAMP
Rap
Src
B-Raf
NF1
Fyn
JAK
PI3K
Ras
PI3K
PKC
CAMK II
Ras GRP
PKA
Rho
PI3K
Ras GAP
Ras GRF
SynGAP
PIP3
PIP2
MEK 1/2
RalGEF
Ral A/B
PI3K
AKT
Ras GEF
AKT
MP1
GSK3
IκB
NF-κB
PAK
MDM2
IκB
P
Ral GAP
ERK 1/2
Ras
PI3K/AKT Signaling
Cell proliferation
MP1
MEK 1/2
c-Raf
ERK 1/2
JNK
Rac
NF-κB
p53
c-Raf
UV irradiation
Nucleus
DNA damage
DNA repair
ERK 1/2
JNK
Cyclin D
FoxO1
BAD
Bcl2
BclXL
ERK 1/2
HIPK2
DNA PK
ATM
ATR
FANCD2
NF-κB
Noxa
Repression of apoptosis & cell cycle arrest
Cell survival
tBID
c-Jun
c-Fos
Aurora A
p53
Chk1
Chk2
NBS1
Elk-1
c-Jun
c-Fos
Cyclin regulation
PUMA
MMP
BIM
JNK
BAK
Cyclin regulation
BRCA1
Cell Cycle regulation and oncogenesis
c-Jun
c-Fos
MIZ1
Myc
MAX
BAX
Mitochondria
CDC25
MDM2
p21 CIP1
Cell cycle arrest
c-Fos
p16 INK4A
CDK 4/6
Cyclin D
p38 MAPK
Mitochondrial membrane permeabilization (MMP)
Rb
P
p19 INK4D
WNT and PTCH regulation
p14 ARF
Cyclins CDKs CKIs
Rb
CDK2
Cyclin E
p18 INK4C
p15 INK4B
CytoC
JNK
Hedgehog target genes
Activation of target genes
E2F
E2F
p21 CIP1
SMAC
MKK 3/6
HIF1α
NICD
RBPJK
HAT1
NICD
p300
p27 KIP1
Gli
Cell survival
CytoC
CIAP
APAF1
Caspase 9
LEF1
RBL1
SMAD4
SMAD 1/5/8
SMAD 2/3
SMAD4
Apoptosis
CBP
CTNN β
TCF3/4
E2F
NICD
Caspase 3/6/7
TAK1
TAB2
TAB1
SMAD4
SMAD 1/5/8
SMAD 2/3
SMAD4
BID
tBID
APC
CTNN β
AXIN
GSK3 β
CTNN β
NICD
TAK 1
TAB2
TAB1
NLK
SMAD4
Caspase 8,10
Gli
SuFu
Fu
G protein
γ-Secretase Complex
CTNN β
CTNN α
CTNN δ
Src
SMAD6
ERK 1/2
SMAD 1/5/8
SMAD 2/3
SMAD7
JNK
ASK1
PSEN 1/2
PEN2
APH1
NCSTN
Dsh
SMAD7
FLIP
FADD
Daxx
PTCH
Smo
Notch 1
Fzd
LRP 1/5/6
CDH E
BMPR 1
BMPR 2
Ras
TGF βR1
TGF βR2
Fas
Hedgehog
WNT 5A
Cd2+
BMP
TGF-β
FasL
WNT1

Common Nicotine Mutations

Chr	Start	End	Ref	Alt	Func.refGene	Gene.refGene	ExonicFunc.refGene	AAChange.refGene	cosmic64	snp137	PolyPhen2 Pred
chr1		69511	A	G	exonic	OR4F5	nonsynonymous SNV	OR4F5:NM_001005484:exon1:c.A421G:p.T141A	NA	rs2691305	B
chr1	663506	663506	T	C	ncRNA_exonic	LOC100133331	NA	NA	NA	NA	NA
chr1	874816	874816	-	T	exonic	SAMD11	frameshift insertion	SAMD11:NM_152486:exon7:c.682_683insT:p.P228fs	NA	rs200996316	NA
chr1	2123473	2123473	T	C	UTR3	C1orf86	NA	NA	NA	NA	NA
chr1	3511934	3511934	A	G	exonic	MEGF6	nonsynonymous SNV	MEGF6:NM_001409:exon3:c.T344C:p.M115T	NA	rs7513275	B
chr1	6526858	6526858	A	-	UTR3	PLEKHG5	NA	NA	NA	NA	NA
chr1	10439620	10439620	-	G	UTR3	KIF1B	NA	NA	NA	NA	NA
chr1	12203319	12203319	T	C	UTR3	TNFRSF8	NA	NA	NA	NA	NA
chr1	12856111	12856111	C	T	exonic	PRAMEF1	nonsynonymous SNV	PRAMEF1:NM_023013:exon4:c.C1391T:p.P464L	NA	rs2982215	D
chr1	12887453	12887453	C	T	exonic	PRAMEF11	nonsynonymous SNV	PRAMEF11:NM_001146344:exon3:c.G404A:p.R135K	NA	rs2486714	NA
chr1	12887480	12887480	C	T	exonic	PRAMEF11	nonsynonymous SNV	PRAMEF11:NM_001146344:exon3:c.G377A:p.C126Y	NA	NA	NA
chr1	12921265	12921265	G	A	exonic	PRAMEF2	synonymous SNV	PRAMEF2:NM_023014:exon4:c.G1056A:p.E352E	NA	rs151184374	NA
chr1	12921272	12921272	G	A	exonic	PRAMEF2	nonsynonymous SNV	PRAMEF2:NM_023014:exon4:c.G1063A:p.V355M	NA	rs145044541	B
chr1	12921274	12921274	G	C	exonic	PRAMEF2	synonymous SNV	PRAMEF2:NM_023014:exon4:c.G1065C:p.V355V	NA	rs17039302	NA
chr1	12953195	12953195	C	T	exonic	PRAMEF10	nonsynonymous SNV	PRAMEF10:NM_001039361:exon4:c.G977A:p.R326H	NA	rs201422133	B
chr1	12955697	12955697	A	G	UTR5	PRAMEF10	NA	NA	NA	rs200630847	NA
chr1	13183780	13183780	A	C	exonic	LOC440563	synonymous SNV	LOC440563:NM_001136561:exon2:c.T93G:p.S31S	ID=COSN199380;OCCURENCE=1(large_intestine)	rs116484938	NA
chr1	15439082	15439082	A	G	ncRNA_exonic	TMEM51-AS1	NA	NA	NA	NA	NA
chr1	15439098	15439098	C	T	ncRNA_exonic	TMEM51-AS1	NA	NA	NA	rs114844404	NA
chr1	15440343	15440343	T	G	ncRNA_exonic	TMEM51-AS1	NA	NA	NA	NA	NA
chr1	16133664	16133664	T	-	UTR3	UQCRHL	NA	NA	NA	NA	NA
chr1	16385178	16385178	G	A	exonic	FAM131C	synonymous SNV	FAM131C:NM_182623:exon7:c.C597T:p.P199P	ID=COSM676526;OCCURENCE=1(prostate),1(lung)	rs80297394	NA
chr1	16890484	16890484	G	C	exonic	NBPF1	unknown	UNKNOWN	NA	rs12117084	NA
chr1	16912164	16912164	C	T	exonic	NBPF1	unknown	UNKNOWN	NA	rs201930116	NA
chr1	16973095	16973095	G	C	ncRNA_exonic	MST1P2	NA	NA	NA	rs75521993	NA
chr1	16975268	16975268	T	C	ncRNA_exonic	MST1P2	NA	NA	NA	rs199945491	NA
chr1	17083101	17083101	A	G	UTR3	MST1L	NA	NA	NA	rs199727569	NA
chr1	17085872	17085872	A	G	exonic	MST1L	nonsynonymous SNV	MST1L:NM_001271733:exon8:c.T949C:p.W317R	D=COSM1127270,COSM1127271;OCCURENCE=1(prostate	rs1806514	NA
chr1	26161107	26161108	AA	-	UTR3	AUNIP	NA	NA	NA	rs139695812	NA
chr1	26608823	26608823	A	G	exonic	UBXN11	synonymous SNV	S390S,UBXN11:NM_145345:exon15:c.T1431C:p.S477S,UI	NA	rs61775084	NA
chr1	28087366	28087366	C	T	UTR3	FAM76A	NA	NA	NA	NA	NA
chr1	28087368	28087368	A	T	UTR3	FAM76A	NA	NA	NA	NA	NA
chr1	28473786	28473786	T	G	UTR3	PTAFR	NA	NA	NA	NA	NA
chr1	33475967	33475967	G	A	UTR3	AK2	NA	NA	NA	rs74066437	NA
chr1	38226760	38226760	-	AA	UTR3	EPHA10	NA	NA	NA	NA	NA
chr1	39879211	39879211	A	G	exonic	KIAA0754	nonsynonymous SNV	KIAA0754:NM_015038:exon1:c.A3274G:p.T1092A	NA	NA	NA
chr1	39879379	39879379	C	G	exonic	KIAA0754	nonsynonymous SNV	KIAA0754:NM_015038:exon1:c.C3442G:p.P1148A	NA	rs34074681	NA
chr1	39976865	39976865	G	A	exonic	BMP8A	synonymous SNV	BMP8A:NM_181809:exon2:c.G354A:p.L118L	NA	NA	NA
chr1	41979207	41979207	T	G	exonic	HIVEP3	synonymous SNV	714:exon7:c.A5685C:p.S1895S,HIVEP3:NM_024503:exon8	NA	NA	NA
chr1	43747937	43747937	-	A	UTR3	C1orf210	NA	NA	NA	NA	NA
chr1	47905783	47905785	TTT	-	UTR3	FOXD2	NA	NA	NA	rs56809638	NA
chr1	50667062	50667062	-	A	UTR3	ELAVL4	NA	NA	NA	NA	NA
chr1	53291327	53291328	AA	-	UTR3	ZYG11B	NA	NA	NA	NA	NA
chr1	61922425	61922425	A	-	UTR3	NFIA	NA	NA	NA	rs67346536	NA
chr1	65901244	65901244	G	T	UTR3	LEPROT	NA	NA	NA	NA	NA
chr1	67465989	67465989	A	T	UTR3	SLC35D1	NA	NA	NA	NA	NA
chr1	67874252	67874252	-	A	UTR3	SERBP1	NA	NA	NA	NA	NA
chr1	89446108	89446108	T	-	UTR3	RBMXL1	NA	NA	NA	NA	NA
chr1	95448279	95448279	G	A	UTR3	ALG14	NA	NA	NA	NA	NA
chr1	1.1E+08	1.1E+08	A	-	UTR3	SORT1	NA	NA	NA	rs111233099	NA
chr1	1.1E+08	1.1E+08	T	-	UTR3	GNAI3	NA	NA	NA	NA	NA
chr1	1.12E+08	1.12E+08	C	-	UTR3	CEPT1	NA	NA	NA	NA	NA
chr1	1.15E+08	1.15E+08	G	T	exonic	AMPD1	synonymous SNV	'72626:exon3:c.C322A:p.R108R,AMPD1:NM_000036:exon4	NA	NA	NA
chr1	1.18E+08	1.18E+08	TT	-	UTR3	PTGFRN	NA	NA	NA	NA	NA
chr1	1.21E+08	1.21E+08	G	T	UTR5	NOTCH2	NA	NA	NA	rs57122008	NA
chr1	1.21E+08	1.21E+08	G	A	exonic	FAM72B	nonsynonymous SNV	FAM72B:NM_001100910:exon3:c.G295A:p.G99R	ID=COSM396486;OCCURENCE=1(lung)	rs1055682	P
chr1	1.45E+08	1.45E+08	TATT	-	UTR3	PDE4DIP	NA	NA	NA	NA	NA
chr1	1.45E+08	1.45E+08	G	A	UTR3	NOTCH2NL	NA	NA	NA	rs201876339	NA
chr1	1.45E+08	1.45E+08	A	G	exonic	NBPF10	nonsynonymous SNV	NBPF10:NM_001039703:exon1:c.A110G:p.N37S	NA	rs12565078	NA
chr1	1.45E+08	1.45E+08	T	C	intronic	NBPF10	NA	NA	NA	NA	NA

chr1	1.47E+08	1.47E+08	A	G	UTR5	BCL9	NA	NA	NA	rs10900376	NA
chr1	1.51E+08	1.51E+08	-	A	UTR3	GOLPH3L	NA	NA	NA	NA	NA
chr1	1.52E+08	1.52E+08	C	T	exonic	RPTN	nonsynonymous SNV	RPTN:NM_001122965:exon3:c.G1510A:p.G504R	NA	NA	B
chr1	1.52E+08	1.52E+08	T	A	exonic	HRNR	synonymous SNV	HRNR:NM_001009931:exon3:c.A6198T:p.P2066P	NA	NA	NA
chr1	1.52E+08	1.52E+08	C	T	exonic	HRNR	nonsynonymous SNV	HRNR:NM_001009931:exon3:c.G4241A:p.C1414Y	NA	rs111689937	NA
chr1	1.52E+08	1.52E+08	C	T	exonic	FLG2	synonymous SNV	FLG2:NM_001014342:exon3:c.G2595A:p.S865S	NA	rs12738471	NA
chr1	1.53E+08	1.53E+08	A	G	UTR3	LCE1C	NA	NA	NA	NA	NA
chr1	1.53E+08	1.53E+08	T	A	exonic	IVL	nonsynonymous SNV	IVL:NM_005547:exon2:c.T614A:p.L205H	NA	NA	NA
chr1	1.53E+08	1.53E+08	T	A	exonic	IVL	nonsynonymous SNV	IVL:NM_005547:exon2:c.T929A:p.M310K	NA	NA	NA
chr1	1.54E+08	1.54E+08	C	A	UTR3	GATAD2B	NA	NA	NA	rs72694218	NA
chr1	1.54E+08	1.54E+08	-	A	UTR3	TPM3	NA	NA	NA	NA	NA
chr1	1.55E+08	1.55E+08	T	G	exonic	KCNN3	nonsynonymous SNV	04087:exon1:c.A707C:p.H236P,KCNN3:NM_002249:exon1	NA	NA	NA
chr1	1.6E+08	1.6E+08	AC	-	UTR3	KCNJ10	NA	NA	NA	NA	NA
chr1	1.6E+08	1.6E+08	T	A	ncRNA_exonic	SUMO1P3	NA	NA	NA	NA	NA
chr1	1.61E+08	1.61E+08	C	G	UTR3	MPZ	NA	NA	NA	NA	NA
chr1	1.69E+08	1.69E+08	A	G	UTR3	XCL1	NA	NA	NA	rs641500	NA
chr1	1.69E+08	1.69E+08	T	A	UTR3	XCL1	NA	NA	NA	rs641496	NA
chr1	1.77E+08	1.77E+08	C	A	exonic	FAM5B	nonsynonymous SNV	FAM5B:NM_021165:exon6:c.C975A:p.D325E	NA	NA	B
chr1	1.86E+08	1.86E+08	A	C	exonic	PRG4	nonsynonymous SNV	7709:exon5:c.A970C:p.T324P,PRG4:NM_001127708:exon	ID=COSM1127223;OCCURENCE=1(prostate)	NA	NA
chr1	1.86E+08	1.86E+08	T	C	exonic	PRG4	synonymous SNV	7709:exon5:c.T1086C:p.T362T,PRG4:NM_001127708:exor	NA	NA	NA
chr1	1.86E+08	1.86E+08	T	C	exonic	PRG4	synonymous SNV	7709:exon5:c.T1110C:p.T370T,PRG4:NM_001127708:exor	ID=COSM424817;OCCURENCE=3(endometrium)	NA	NA
chr1	1.86E+08	1.86E+08	A	C	exonic	PRG4	nonsynonymous SNV	7709:exon5:c.A1252C:p.T418P,PRG4:NM_001127708:exo	NA	NA	NA
chr1	1.86E+08	1.86E+08	T	C	exonic	PRG4	synonymous SNV	7709:exon5:c.T1275C:p.T425T,PRG4:NM_001127708:exo	NA	NA	NA
chr1	1.86E+08	1.86E+08	A	C	exonic	PRG4	nonsynonymous SNV	7709:exon5:c.A1384C:p.T462P,PRG4:NM_001127708:exo	NA	NA	NA
chr1	1.86E+08	1.86E+08	G	C	exonic	PRG4	synonymous SNV	7709:exon5:c.G1596C:p.T532T,PRG4:NM_001127708:exo	NA	NA	NA
chr1	1.97E+08	1.97E+08	C	A	exonic	CFHR2	nonsynonymous SNV	CFHR2:NM_005666:exon2:c.C76A:p.P26T	NA	NA	D
chr1	2.01E+08	2.01E+08	T	C	exonic	IGFN1	nonsynonymous SNV	IGFN1:NM_001164586:exon12:c.T4823C:p.V1608A	NA	rs201505263	NA
chr1	2.01E+08	2.01E+08	T	G	exonic	IGFN1	nonsynonymous SNV	IGFN1:NM_001164586:exon12:c.T5747G:p.V1916G	NA	rs200871453	NA
chr1	2.01E+08	2.01E+08	C	G	exonic	IGFN1	nonsynonymous SNV	IGFN1:NM_001164586:exon12:c.C6395G:p.T2132R	NA	NA	NA
chr1	2.01E+08	2.01E+08	A	-	UTR3	TNNI1	NA	NA	NA	rs35058233	NA
chr1	2.02E+08	2.02E+08	C	-	UTR3	IPO9	NA	NA	NA	rs112011484	NA
chr1	2.02E+08	2.02E+08	A	-	UTR3	ELF3	NA	NA	NA	rs11303270	NA
chr1	2.02E+08	2.02E+08	A	C	UTR3	GPR37L1	NA	NA	NA	NA	NA
chr1	2.02E+08	2.02E+08	TT	-	UTR3	GPR37L1	NA	NA	NA	NA	NA
chr1	2.05E+08	2.05E+08	T	C	UTR5	CDK18	NA	NA	NA	rs77694338	NA
chr1	2.06E+08	2.06E+08	G	C	UTR3	RAB7L1	NA	NA	NA	rs823134	NA
chr1	2.07E+08	2.07E+08	AC	-	UTR3	PIGR	NA	NA	NA	NA	NA
chr1	2.08E+08	2.08E+08	T	C	UTR5	CR2	NA	NA	NA	rs3813946	NA
chr1	2.1E+08	2.1E+08	T	-	UTR5	MIR205HG	NA	NA	NA	NA	NA
chr1	2.28E+08	2.28E+08	-	A	UTR5	CDC42BPA	NA	NA	NA	NA	NA
chr1	2.28E+08	2.28E+08	-	T	UTR3	IBA57	NA	NA	NA	NA	NA
chr1	2.29E+08	2.29E+08	A	G	exonic	OBSCN	nonsynonymous SNV	31R,OBSCN:NM_052843:exon50:c.A13142G:p.H4381R,OB	NA	rs1150912	NA
chr1	2.32E+08	2.32E+08	G	A	ncRNA_UTR3	DISC1	NA	NA	NA	rs821618	NA
chr1	2.33E+08	2.33E+08	G	A	exonic	SIPA1L2	nonsynonymous SNV	SIPA1L2:NM_020808:exon7:c.C2504T:p.A835V	NA	rs142752379	P
chr1	2.37E+08	2.37E+08	G	T	UTR3	MTR	NA	NA	NA	NA	NA
chr1	2.38E+08	2.38E+08	T	-	UTR3	RYR2	NA	NA	NA	NA	NA
chr1	2.4E+08	2.4E+08	G	A	exonic	FMN2	synonymous SNV	FMN2:NM_020066:exon5:c.G2820A:p.A940A	NA	rs145628188	NA
chr1	2.4E+08	2.4E+08	C	T	exonic	FMN2	nonsynonymous SNV	FMN2:NM_020066:exon5:c.C2828T:p.P943L	NA	rs199628038	NA
chr1	2.41E+08	2.41E+08	-	T	UTR3	FMN2	NA	NA	NA	rs139197792	NA
chr1	2.45E+08	2.45E+08	-	GTA	UTR3	C1orf101	NA	NA	NA	rs201380603	NA
chr1	2.49E+08	2.49E+08	A	G	exonic	OR2T3	nonsynonymous SNV	OR2T3:NM_001005495:exon1:c.A829G:p.M277V	NA	rs74498773	P
chr2	5835698	5835698	T	-	UTR3	SOX11	NA	NA	NA	rs67646557	NA
chr2	5839842	5839847	TTTTTT	-	UTR3	SOX11	NA	NA	NA	NA	NA
chr2	24253007	24253007	-	A	UTR3	C2orf44	NA	NA	NA	NA	NA
chr2	24991386	24991388	AAA	-	UTR3	NCOA1	NA	NA	NA	NA	NA
chr2	26680509	26680509	A	T	UTR3	OTOF	NA	NA	NA	NA	NA
chr2	27804802	27804802	A	G	exonic	C2orf16	nonsynonymous SNV	C2orf16:NM_032266:exon1:c.A5363G:p.K1788R	NA	rs187723704	NA
chr2	29023081	29023081	C	T	UTR3	PPP1CB	NA	NA	NA	NA	NA
chr2	29170759	29170759	G	A	UTR3	WDR43	NA	NA	NA	rs13397658	NA
chr2	29448423	29448423	T	G	exonic	ALK	nonsynonymous SNV	ALK:NM_004304:exon19:c.A3076C:p.T1026P	NA	NA	B
chr2	30670215	30670215	A	C	UTR5	LCLAT1	NA	NA	NA	NA	NA
chr2	33787988	33787988	A	T	UTR3	RASGRP3	NA	NA	NA	NA	NA

chr2	33787989	33787989	C	G	UTR3	RASGRP3	NA	NA	NA	NA	NA
chr2	33787990	33787990	T	A	UTR3	RASGRP3	NA	NA	NA	NA	NA
chr2	37479024	37479024	A	-	UTR3	PRKD3	NA	NA	NA	NA	NA
chr2	45172485	45172485	G	A	UTR3	SIX3	NA	NA	NA	rs60528008	NA
chr2	46707808	46707808	C	G	exonic	TMEM247	nonsynonymous SNV	TMEM247:NM_001145051:exon2:c.C382G:p.Q128E	NA	rs74318890	NA
chr2	46852206	46852206	G	T	UTR3	CRIPT	NA	NA	NA	NA	NA
chr2	48605828	48605828	T	-	UTR3	FOXN2	NA	NA	NA	NA	NA
chr2	51259649	51259660	AGAGAGAG	-	UTR5	NRXN1	NA	NA	NA	NA	NA
chr2	55647020	55647020	A	C	UTR5	CCDC88A	NA	NA	NA	NA	NA
chr2	55647025	55647025	A	C	UTR5	CCDC88A	NA	NA	NA	NA	NA
chr2	71043885	71043885	T	G	exonic	CLEC4F	nonsynonymous SNV	58027:exon4:c.A628C:p.I210L,CLEC4F:NM_173535:exon4	NA	NA	P
chr2	85284303	85284303	A	-	UTR3	KCMF1	NA	NA	NA	NA	NA
chr2	85531118	85531118	A	C	exonic	TCF7L1	synonymous SNV	TCF7L1:NM_031283:exon6:c.A759C:p.P253P	NA	NA	NA
chr2	85549667	85549667	G	C	UTR3	TGOLN2	NA	NA	NA	NA	NA
chr2	85554315	85554315	A	G	exonic	TGOLN2	synonymous SNV	180N,TGOLN2:NM_001206844:exon2:c.T540C:p.N180N,T	NA	NA	NA
chr2	87002452	87002453	TT	-	UTR3	RMND5A	NA	NA	NA	NA	NA
chr2	89082379	89082379	G	A	ncRNA_exonic	ANKRD36BP2	NA	NA	NA	rs143191250	NA
chr2	92129614	92129614	G	T	ncRNA_exonic	ACTR3BP2	NA	NA	NA	NA	NA
chr2	97779341	97779341	T	G	UTR5	ANKRD36	NA	NA	NA	rs3866777	NA
chr2	98128228	98128228	G	A	exonic	ANKRD36B	synonymous SNV	ANKRD36B:NM_025190:exon39:c.C3093T:p.S1031S	ID=COSN395741;OCCURENCE=1(lung)	rs201190268	NA
chr2	98129733	98129733	A	C	exonic	ANKRD36B	stopgain SNV	ANKRD36B:NM_025190:exon38:c.T2715G:p.Y905X	NA	rs145533718	NA
chr2	98356250	98356250	G	C	UTR3	ZAP70	NA	NA	NA	rs2278699	NA
chr2	1.02E+08	1.02E+08	A	G	UTR5	RFX8	NA	NA	NA	rs62154334	NA
chr2	1.03E+08	1.03E+08	G	A	UTR3	MAP4K4	NA	NA	NA	NA	NA
chr2	1.07E+08	1.07E+08	AAC	-	UTR3	ST6GAL2	NA	NA	NA	rs140467729	NA
chr2	1.07E+08	1.07E+08	G	A	UTR3	ST6GAL2	NA	NA	NA	NA	NA
chr2	1.13E+08	1.13E+08	G	T	UTR3	ZC3H6	NA	NA	NA	NA	NA
chr2	1.19E+08	1.19E+08	A	-	UTR3	CCDC93	NA	NA	NA	NA	NA
chr2	1.2E+08	1.2E+08	T	G	UTR5	EN1	NA	NA	NA	NA	NA
chr2	1.31E+08	1.31E+08	-	CT	ncRNA_exonic	LOC440905	NA	NA	NA	rs3058424	NA
chr2	1.33E+08	1.33E+08	G	C	ncRNA_exonic	ANKRD30BL	NA	NA	NA	rs112494139	NA
chr2	1.33E+08	1.33E+08	G	A	ncRNA_exonic	ANKRD30BL	NA	NA	NA	rs141716220	NA
chr2	1.33E+08	1.33E+08	G	A	ncRNA_exonic	ANKRD30BL	NA	NA	NA	rs148180857	NA
chr2	1.52E+08	1.52E+08	T	C	exonic	NEB	nonsynonymous SNV	2D,NEB:NM_001164508:exon95:c.A14734G:p.N4912D,NE	NA	rs10909569	NA
chr2	1.53E+08	1.53E+08	T	-	UTR3	ARL5A	NA	NA	NA	NA	NA
chr2	1.53E+08	1.53E+08	C	T	UTR3	CACNB4	NA	NA	NA	rs7597215	NA
chr2	1.7E+08	1.7E+08	-	A	UTR3	LRP2	NA	NA	NA	NA	NA
chr2	1.74E+08	1.74E+08	-	A	ncRNA_UTR3	ZAK	NA	NA	NA	NA	NA
chr2	1.77E+08	1.77E+08	C	T	UTR3	HOXD9	NA	NA	NA	NA	NA
chr2	2.02E+08	2.02E+08	TT	-	UTR3	FAM126B	NA	NA	NA	NA	NA
chr2	2.04E+08	2.04E+08	C	T	UTR3	FAM117B	NA	NA	NA	NA	NA
chr2	2.09E+08	2.09E+08	AG	-	UTR3	FZD5	NA	NA	NA	rs201847302	NA
chr2	2.12E+08	2.12E+08	A	-	UTR3	ERBB4	NA	NA	NA	NA	NA
chr2	2.17E+08	2.17E+08	C	A	UTR3	MREG	NA	NA	NA	NA	NA
chr2	2.2E+08	2.2E+08	G	T	UTR3	FAM134A	NA	NA	NA	NA	NA
chr2	2.25E+08	2.25E+08	C	A	UTR3	WDFY1	NA	NA	NA	NA	NA
chr2	2.33E+08	2.33E+08	T	C	UTR3	DIS3L2	NA	NA	NA	NA	NA
chr2	2.33E+08	2.33E+08	T	-	UTR3	CHRNG	NA	NA	NA	NA	NA
chr2	2.35E+08	2.35E+08	A	C	UTR3	HJURP	NA	NA	NA	rs74270439	NA
chr2	2.41E+08	2.41E+08	A	G	exonic	PRR21	synonymous SNV	PRR21:NM_001080835:exon1:c.T237C:p.H79H	NA	rs117846189	NA
chr3	3214495	3214495	C	A	exonic	CRBN	nonsynonymous SNV	73482:exon4:c.G489T:p.R163S,CRBN:NM_016302:exon4:	NA	NA	D
chr3	11599950	11599950	T	-	UTR3	VGLL4	NA	NA	NA	NA	NA
chr3	14105938	14105938	T	A	ncRNA_exonic	TPRXL	NA	NA	NA	NA	NA
chr3	14105996	14105996	A	G	ncRNA_exonic	TPRXL	NA	NA	NA	NA	NA
chr3	14106174	14106174	T	C	ncRNA_exonic	TPRXL	NA	NA	NA	NA	NA
chr3	28364846	28364846	T	-	UTR3	AZI2	NA	NA	NA	rs200293551	NA
chr3	41281477	41281477	C	T	UTR3	CTNNB1	NA	NA	NA	rs201175238	NA
chr3	47050999	47050999	G	C	UTR3	NBEAL2	NA	NA	NA	NA	NA
chr3	49044650	49044650	A	G	UTR5	WDR6	NA	NA	NA	NA	NA
chr3	49572947	49572947	C	A	UTR3	DAG1	NA	NA	NA	NA	NA
chr3	49694888	49694888	T	C	exonic	BSN	synonymous SNV	BSN:NM_003458:exon5:c.T7899C:p.L2633L	NA	NA	NA
chr3	55502167	55502167	A	-	UTR3	WNT5A	NA	NA	NA	NA	NA

chr3	63988881	63988881	C	T	UTR3	ATXN7	NA	NA	NA	NA	NA
chr3	64672461	64672461	T	G	exonic	ADAMTS9	nonsynonymous SNV	ADAMTS9:NM_182920:exon2:c.A299C:p.Y100S	NA	NA	D
chr3	67049615	67049615	G	T	exonic	KBTBD8	nonsynonymous SNV	KBTBD8:NM_032505:exon2:c.G227T:p.R76I	NA	NA	D
chr3	71821711	71821711	A	-	UTR3	PROK2	NA	NA	NA	rs71713597	NA
chr3	75716090	75716090	T	C	ncRNA_exonic	FLJ20518	NA	NA	NA	rs144150259	NA
chr3	75787116	75787116	T	G	exonic	ZNF717	nonsynonymous SNV	ZNF717:NM_001128223:exon5:c.A1658C:p.H553P	NA	rs142824987	NA
chr3	75787620	75787620	T	C	exonic	ZNF717	nonsynonymous SNV	ZNF717:NM_001128223:exon5:c.A1154G:p.H385R	NA	rs145606249	NA
chr3	75790852	75790852	G	A	exonic	ZNF717	synonymous SNV	ZNF717:NM_001128223:exon3:c.C93T:p.F31F	NA	rs201689840	NA
chr3	98515881	98515882	AA	-	UTR3	DCBLD2	NA	NA	NA	rs56369137	NA
chr3	1.05E+08	1.05E+08	G	T	exonic	CBLB	nonsynonymous SNV	CBLB:NM_170662:exon12:c.C1618A:p.Q540K	NA	NA	D
chr3	1.2E+08	1.2E+08	-	A	UTR3	GSK3B	NA	NA	NA	NA	NA
chr3	1.2E+08	1.2E+08	A	C	exonic	GPR156	nonsynonymous SNV	3271:exon9:c.T1610G:p.V537G,GPR156:NM_153002:exon	NA	NA	D
chr3	1.21E+08	1.21E+08	T	-	UTR3	GTF2E1	NA	NA	NA	rs71133517	NA
chr3	1.23E+08	1.23E+08	T	-	ncRNA_UTR3	MYLK	NA	NA	NA	rs35930843	NA
chr3	1.26E+08	1.26E+08	-	T	UTR3	ZXDC	NA	NA	NA	NA	NA
chr3	1.29E+08	1.29E+08	T	G	exonic	PLXND1	nonsynonymous SNV	PLXND1:NM_015103:exon31:c.A5108C:p.Y1703S	NA	NA	D
chr3	1.34E+08	1.34E+08	C	G	UTR5	CEP63	NA	NA	NA	NA	NA
chr3	1.41E+08	1.41E+08	-	T	UTR3	RNF7	NA	NA	NA	NA	NA
chr3	1.42E+08	1.42E+08	C	T	UTR3	GK5	NA	NA	NA	NA	NA
chr3	1.5E+08	1.5E+08	G	T	UTR3	TSC22D2	NA	NA	NA	rs201497156	NA
chr3	1.52E+08	1.52E+08	T	-	UTR3	MBNL1	NA	NA	NA	rs142162205	NA
chr3	1.7E+08	1.7E+08	G	T	UTR3	CLDN11	NA	NA	NA	NA	NA
chr3	1.7E+08	1.7E+08	C	T	UTR3	CLDN11	NA	NA	NA	NA	NA
chr3	1.84E+08	1.84E+08	GTGTGT	-	UTR3	EPHB3	NA	NA	NA	rs35514470	NA
chr3	1.95E+08	1.95E+08	T	C	ncRNA_exonic	SDHAP2	NA	NA	ID=COSN404759;OCCURENCE=1(lung)	rs6583273	NA
chr3	1.96E+08	1.96E+08	T	C	exonic	MUC4	nonsynonymous SNV	MUC4:NM_018406:exon2:c.A12568G:p.T4190A	NA	rs200813870	NA
chr3	1.96E+08	1.96E+08	A	G	exonic	MUC4	synonymous SNV	MUC4:NM_018406:exon2:c.T12159C:p.N4053N	NA	rs201191776	NA
chr3	1.96E+08	1.96E+08	T	C	exonic	MUC4	nonsynonymous SNV	MUC4:NM_018406:exon2:c.A12136G:p.T4046A	ID=COSM479895;OCCURENCE=2(kidney)	rs62282465	NA
chr3	1.96E+08	1.96E+08	T	A	exonic	MUC4	synonymous SNV	MUC4:NM_018406:exon2:c.A11796T:p.G3932G	NA	rs200897653	NA
chr3	1.96E+08	1.96E+08	A	G	exonic	MUC4	synonymous SNV	MUC4:NM_018406:exon2:c.T11580C:p.T3860T	NA	NA	NA
chr3	1.96E+08	1.96E+08	A	G	exonic	MUC4	nonsynonymous SNV	MUC4:NM_018406:exon2:c.T11162C:p.V3721A	NA	NA	NA
chr3	1.96E+08	1.96E+08	G	A	exonic	MUC4	nonsynonymous SNV	MUC4:NM_018406:exon2:c.C11018T:p.A3673V	NA	rs202062831	NA
chr3	1.96E+08	1.96E+08	C	G	exonic	MUC4	nonsynonymous SNV	MUC4:NM_018406:exon2:c.G10405C:p.D3469H	NA	rs76458038	NA
chr3	1.96E+08	1.96E+08	C	G	exonic	MUC4	synonymous SNV	MUC4:NM_018406:exon2:c.G8733C:p.T2911T	NA	rs28385894	NA
chr3	1.96E+08	1.96E+08	A	G	exonic	MUC4	nonsynonymous SNV	MUC4:NM_018406:exon2:c.T8570C:p.V2857A	NA	NA	NA
chr3	1.96E+08	1.96E+08	C	T	exonic	MUC4	nonsynonymous SNV	MUC4:NM_018406:exon2:c.G8464A:p.A2822T	NA	rs28420515	NA
chr3	1.96E+08	1.96E+08	G	A	exonic	MUC4	nonsynonymous SNV	MUC4:NM_018406:exon2:c.C8282T:p.A2761V	NA	rs201935809	NA
chr3	1.96E+08	1.96E+08	A	G	exonic	MUC4	nonsynonymous SNV	MUC4:NM_018406:exon2:c.T7445C:p.L2482P	NA	NA	NA
chr3	1.96E+08	1.96E+08	A	G	exonic	MUC4	nonsynonymous SNV	MUC4:NM_018406:exon2:c.T7418C:p.V2473A	NA	rs200807157	NA
chr3	1.96E+08	1.96E+08	C	T	exonic	MUC4	synonymous SNV	MUC4:NM_018406:exon2:c.G7320A:p.S2440S	NA	rs71321826	NA
chr3	1.96E+08	1.96E+08	A	G	exonic	MUC4	nonsynonymous SNV	MUC4:NM_018406:exon2:c.T6293C:p.L2098P	NA	rs141952317	NA
chr3	1.96E+08	1.96E+08	A	T	exonic	MUC4	synonymous SNV	MUC4:NM_018406:exon2:c.T6081A:p.T2027T	NA	NA	NA
chr3	1.96E+08	1.96E+08	A	G	exonic	MUC4	synonymous SNV	MUC4:NM_018406:exon2:c.T5550C:p.S1850S	NA	NA	NA
chr3	1.96E+08	1.96E+08	C	T	exonic	MUC4	synonymous SNV	MUC4:NM_018406:exon2:c.G5400A:p.S1800S	NA	NA	NA
chr3	1.96E+08	1.96E+08	C	G	exonic	MUC4	nonsynonymous SNV	MUC4:NM_018406:exon2:c.G4885C:p.D1629H	NA	rs78438024	NA
chr3	1.96E+08	1.96E+08	C	G	exonic	MUC4	nonsynonymous SNV	MUC4:NM_018406:exon2:c.G4789C:p.D1597H	NA	NA	NA
chr3	1.96E+08	1.96E+08	C	G	exonic	MUC4	synonymous SNV	MUC4:NM_018406:exon2:c.G4461C:p.T1487T	NA	rs12489814	NA
chr3	1.96E+08	1.96E+08	C	T	exonic	MUC4	nonsynonymous SNV	MUC4:NM_018406:exon2:c.G4216A:p.A1406T	NA	NA	NA
chr3	1.96E+08	1.96E+08	A	G	exonic	MUC4	synonymous SNV	MUC4:NM_018406:exon2:c.T3885C:p.T1295T	NA	NA	NA
chr3	1.96E+08	1.96E+08	A	G	exonic	MUC4	synonymous SNV	MUC4:NM_018406:exon2:c.T3471C:p.D1157D	NA	NA	NA
chr3	1.96E+08	1.96E+08	A	G	exonic	MUC4	synonymous SNV	MUC4:NM_018406:exon2:c.T3468C:p.T1156T	NA	NA	NA
chr3	1.96E+08	1.96E+08	A	G	exonic	MUC4	synonymous SNV	MUC4:NM_018406:exon2:c.T3276C:p.T1092T	NA	rs112260425	NA
chr3	1.96E+08	1.96E+08	C	T	exonic	MUC4	nonsynonymous SNV	MUC4:NM_018406:exon2:c.G3085A:p.D1029N	NA	rs201613765	NA
chr3	1.96E+08	1.96E+08	T	C	ncRNA_exonic	SDHAP1	NA	NA	NA	rs189732960	NA
chr3	1.96E+08	1.96E+08	A	G	ncRNA_exonic	SDHAP1	NA	NA	NA	rs199975852	NA
chr3	1.96E+08	1.96E+08	A	G	ncRNA_exonic	SDHAP1	NA	NA	NA	rs200738162	NA
chr3	1.96E+08	1.96E+08	T	C	UTR3	FBXO45	NA	NA	NA	rs9834490	NA
chr3	1.96E+08	1.96E+08	T	-	UTR3	CEP19	NA	NA	NA	rs71161952	NA
chr3	1.97E+08	1.97E+08	-	TTTTTTT	UTR3	SENP5	NA	NA	NA	NA	NA
chr3	1.98E+08	1.98E+08	A	C	exonic	FAM157A	synonymous SNV	FAM157A:NM_001145248:exon5:c.A927C:p.R309R	NA	rs202207735	NA
chr4	87567	87567	G	T	UTR3	ZNF595	NA	NA	NA	NA	NA
chr4	87570	87570	C	T	UTR3	ZNF595	NA	NA	NA	NA	NA

chr4	87574	87574	G	T	UTR3	ZNF595	NA	NA	NA	NA	NA
chr4	87604	87604	-	T	UTR3	ZNF595	NA	NA	NA	NA	NA
chr4	1020221	1020221	T	C	UTR3	FGFRL1	NA	NA	NA	NA	NA
chr4	1388662	1388662	G	C	exonic	CRIPAK	synonymous SNV	CRIPAK:NM_175918:exon1:c.G363C:p.P121P	NA	rs9762106	NA
chr4	1388755	1388755	C	G	exonic	CRIPAK	synonymous SNV	CRIPAK:NM_175918:exon1:c.C456G:p.P152P	NA	rs141481663	NA
chr4	1388757	1388757	T	C	exonic	CRIPAK	nonsynonymous SNV	CRIPAK:NM_175918:exon1:c.T458C:p.M153T	ID=COSM225540;OCCURENCE=1(NS)	rs138170890	P
chr4	1388848	1388848	A	C	exonic	CRIPAK	synonymous SNV	CRIPAK:NM_175918:exon1:c.A549C:p.P183P	NA	rs74511366	NA
chr4	1389059	1389059	T	C	exonic	CRIPAK	nonsynonymous SNV	CRIPAK:NM_175918:exon1:c.T760C:p.C254R	NA	NA	D
chr4	1389070	1389070	A	G	exonic	CRIPAK	synonymous SNV	CRIPAK:NM_175918:exon1:c.A771G:p.G257G	NA	rs151096093	NA
chr4	2087366	2087366	G	T	exonic	POLN	nonsynonymous SNV	POLN:NM_181808:exon19:c.C2171A:p.A724E	NA	NA	B
chr4	22439886	22439886	C	A	exonic	GPR125	nonsynonymous SNV	GPR125:NM_145290:exon8:c.G1078T:p.D360Y	NA	NA	D
chr4	25280824	25280824	C	T	UTR3	PI4K2B	NA	NA	NA	NA	NA
chr4	25749200	25749201	AC	-	UTR3	SEL1L3	NA	NA	NA	rs147448161	NA
chr4	37614259	37614260	AA	-	UTR3	RELL1	NA	NA	NA	NA	NA
chr4	41747030	41747030	T	G	UTR3	PHOX2B	NA	NA	NA	NA	NA
chr4	41747047	41747047	T	G	UTR3	PHOX2B	NA	NA	NA	NA	NA
chr4	71521932	71521932	A	-	UTR3	IGJ	NA	NA	NA	NA	NA
chr4	76555253	76555253	G	A	UTR5	CDKL2	NA	NA	NA	NA	NA
chr4	81974755	81974755	-	T	UTR3	BMP3	NA	NA	NA	NA	NA
chr4	82088324	82088324	G	A	exonic	PRKG2	synonymous SNV	PRKG2:NM_006259:exon5:c.C903T:p.C301C	NA	NA	NA
chr4	84011680	84011680	-	A	UTR3	PLAC8	NA	NA	NA	NA	NA
chr4	88536471	88536471	A	G	exonic	DSPP	nonsynonymous SNV	DSPP:NM_014208:exon5:c.A2657G:p.N886S	NA	NA	NA
chr4	88537513	88537513	A	C	exonic	DSPP	nonsynonymous SNV	DSPP:NM_014208:exon5:c.A3699C:p.E1233D	NA	rs112275895	NA
chr4	90646502	90646505	AGAG	-	UTR3	SNCA	NA	NA	NA	rs141457381	NA
chr4	90757919	90757919	T	C	ncRNA_UTR5	SNCA	NA	NA	NA	NA	NA
chr4	94750481	94750481	T	G	exonic	ATOH1	nonsynonymous SNV	ATOH1:NM_005172:exon1:c.T404G:p.V135G	NA	NA	B
chr4	96076626	96076626	-	TT	UTR3	BMPR1B	NA	NA	NA	NA	NA
chr4	1.04E+08	1.04E+08	A	T	UTR3	NFKB1	NA	NA	NA	NA	NA
chr4	1.09E+08	1.09E+08	GTGT	-	ncRNA_exonic	RPL34-AS1	NA	NA	NA	rs150491091	NA
chr4	1.29E+08	1.29E+08	T	C	UTR3	MFSD8	NA	NA	NA	NA	NA
chr4	1.39E+08	1.39E+08	G	T	ncRNA_UTR3	SLC7A11	NA	NA	NA	NA	NA
chr4	1.4E+08	1.4E+08	C	T	UTR3	CCRN4L	NA	NA	NA	NA	NA
chr4	1.4E+08	1.4E+08	A	T	UTR3	CCRN4L	NA	NA	NA	rs74826922	NA
chr4	1.4E+08	1.4E+08	T	-	UTR3	NAA15	NA	NA	NA	rs72425271	NA
chr4	1.46E+08	1.46E+08	A	G	UTR3	OTUD4	NA	NA	NA	NA	NA
chr4	1.58E+08	1.58E+08	A	G	UTR5	GRIA2	NA	NA	NA	NA	NA
chr4	1.6E+08	1.6E+08	AA	-	UTR3	FNIP2	NA	NA	NA	NA	NA
chr5	661502	661502	A	C	UTR3	TPPP	NA	NA	NA	NA	NA
chr5	663244	663244	T	G	UTR3	TPPP	NA	NA	NA	NA	NA
chr5	10279657	10279657	T	-	UTR3	CMBL	NA	NA	NA	NA	NA
chr5	16465810	16465810	A	C	UTR5	ZNF622	NA	NA	NA	NA	NA
chr5	16465854	16465854	A	C	UTR5	ZNF622	NA	NA	NA	NA	NA
chr5	23521146	23521146	G	T	exonic	PRDM9	synonymous SNV	PRDM9:NM_020227:exon6:c.G366T:p.A122A	NA	NA	NA
chr5	35909976	35909976	C	A	exonic	CAPSL	nonsynonymous SNV	42625:exon4:c.G517T:p.D173Y,CAPSL:NM_144647:exon4:	NA	NA	D
chr5	55168106	55168106	G	T	exonic	IL31RA	nonsynonymous SNV	242637:exon4:c.G281T:p.G94V,IL31RA:NM_139017:exon4	NA	NA	B
chr5	71503811	71503811	G	T	UTR3	MAP1B	NA	NA	NA	NA	NA
chr5	78324457	78324457	C	A	exonic	DMGDH	nonsynonymous SNV	DMGDH:NM_013391:exon12:c.G1831T:p.A611S	NA	NA	B
chr5	80738520	80738520	G	T	exonic	SSBP2	nonsynonymous SNV	5735:exon12:c.C711A:p.N237K,SSBP2:NM_001256736:exo	NA	NA	D
chr5	90666826	90666826	A	-	UTR3	ARRDC3	NA	NA	NA	NA	NA
chr5	92905599	92905599	C	A	ncRNA_exonic	FLJ42709	NA	NA	NA	NA	NA
chr5	1.02E+08	1.02E+08	GT	-	UTR3	SLCO4C1	NA	NA	NA	NA	NA
chr5	1.15E+08	1.15E+08	A	C	UTR3	ATG12	NA	NA	NA	rs35903246	NA
chr5	1.15E+08	1.15E+08	-	AGGC	UTR5	AP3S1	NA	NA	NA	rs144226661	NA
chr5	1.15E+08	1.15E+08	C	T	exonic	AQPEP	synonymous SNV	AQPEP:NM_173800:exon1:c.C64T:p.L22L	ID=COSM1130961;OCCURENCE=1(prostate)	rs10062297	NA
chr5	1.22E+08	1.22E+08	CACACACA	-	UTR3	SNX2	NA	NA	NA	rs71851865	NA
chr5	1.23E+08	1.23E+08	G	A	exonic	CEP120	synonymous SNV	226:exon10:c.C1368T:p.F456F,CEP120:NM_153223:exon	NA	NA	NA
chr5	1.28E+08	1.28E+08	A	-	UTR3	FBN2	NA	NA	NA	NA	NA
chr5	1.32E+08	1.32E+08	T	G	ncRNA_intronic	C5orf56	NA	NA	NA	NA	NA
chr5	1.34E+08	1.34E+08	G	C	exonic	PHF15	nonsynonymous SNV	PHF15:NM_015288:exon11:c.G2015C:p.S672T	NA	NA	D
chr5	1.34E+08	1.34E+08	C	A	UTR3	DDX46	NA	NA	NA	NA	NA
chr5	1.37E+08	1.37E+08	A	C	UTR3	HNRNPA0	NA	NA	NA	NA	NA
chr5	1.37E+08	1.37E+08	T	C	UTR3	HNRNPA0	NA	NA	NA	NA	NA

chr5	1.37E+08	1.37E+08	G	C	exonic	HNRNPA0	synonymous SNV	HNRNPA0:NM_006805:exon1:c.C897G:p.G299G	NA	NA	NA
chr5	1.37E+08	1.37E+08	A	C	exonic	HNRNPA0	synonymous SNV	HNRNPA0:NM_006805:exon1:c.T888G:p.G296G	NA	NA	NA
chr5	1.41E+08	1.41E+08	G	T	UTR3	PCDHB9	NA	NA	NA	NA	NA
chr5	1.49E+08	1.49E+08	C	G	ncRNA_exonic	MIR143HG	NA	NA	NA	rs519814	NA
chr5	1.49E+08	1.49E+08	-	T	ncRNA_exonic	MIR143HG	NA	NA	NA	NA	NA
chr5	1.49E+08	1.49E+08	C	T	UTR3	PDE6A	NA	NA	NA	rs72660270	NA
chr5	1.51E+08	1.51E+08	-	A	UTR3	SLC36A2	NA	NA	NA	rs33912867	NA
chr5	1.52E+08	1.52E+08	-	A	UTR3	NMUR2	NA	NA	NA	NA	NA
chr5	1.57E+08	1.57E+08	-	A	UTR3	CYFIP2	NA	NA	NA	NA	NA
chr5	1.58E+08	1.58E+08	-	A	UTR3	EBF1	NA	NA	NA	NA	NA
chr5	1.58E+08	1.58E+08	C	A	UTR3	EBF1	NA	NA	NA	rs112280787	NA
chr5	1.61E+08	1.61E+08	-	T	UTR3	GABRB2	NA	NA	NA	NA	NA
chr5	1.72E+08	1.72E+08	-	A	UTR3	NEURL1B	NA	NA	NA	rs5873335	NA
chr5	1.73E+08	1.73E+08	A	G	exonic	BOD1	synonymous SNV	BOD1:NM_138369:exon3:c.T543C:p.S181S	ID=COSM311048;OCCURENCE=1(lung)	rs77014290	NA
chr5	1.75E+08	1.75E+08	A	C	UTR5	HRH2	NA	NA	NA	NA	NA
chr5	1.76E+08	1.76E+08	A	G	UTR3	EIF4E1B	NA	NA	NA	NA	NA
chr5	1.76E+08	1.76E+08	T	G	UTR3	EIF4E1B	NA	NA	NA	NA	NA
chr6	407629	407629	-	T	UTR3	IRF4	NA	NA	NA	NA	NA
chr6	7249296	7249299	GAGA	-	UTR3	RREB1	NA	NA	NA	rs66585613	NA
chr6	10796303	10796303	T	G	exonic	MAK	nonsynonymous SNV	Q357H,MAK:NM_001242957:exon9:c.A1071C:p.Q357H,M	NA	NA	P
chr6	18129306	18129306	C	A	UTR3	TPMT	NA	NA	NA	NA	NA
chr6	21597697	21597697	C	T	UTR3	SOX4	NA	NA	NA	NA	NA
chr6	24775181	24775181	G	T	UTR5	GMNN	NA	NA	NA	rs2754775	NA
chr6	25769247	25769247	C	A	exonic	SLC17A4	synonymous SNV	SLC17A4:NM_005495:exon3:c.C126A:p.I42I	NA	NA	NA
chr6	29857177	29857177	G	A	ncRNA_exonic	HLA-H	NA	NA	NA	rs1059578	NA
chr6	29892568	29892568	C	A	ncRNA_exonic	HCG4B	NA	NA	NA	NA	NA
chr6	29892570	29892570	A	T	ncRNA_exonic	HCG4B	NA	NA	NA	NA	NA
chr6	30573785	30573785	A	G	exonic	PPP1R10	synonymous SNV	PPP1R10:NM_002714:exon10:c.T768C:p.T256T	NA	NA	NA
chr6	30673064	30673064	C	T	exonic	MDC1	nonsynonymous SNV	MDC1:NM_014641:exon10:c.G3896A:p.R1299Q	NA	rs144657716	NA
chr6	30673432	30673432	C	T	exonic	MDC1	synonymous SNV	MDC1:NM_014641:exon10:c.G3528A:p.Q1176Q	NA	rs78306040	NA
chr6	30918388	30918388	T	C	exonic	DPCR1	nonsynonymous SNV	DPCR1:NM_080870:exon2:c.T2147C:p.L716P	NA	NA	NA
chr6	30918690	30918690	T	C	exonic	DPCR1	nonsynonymous SNV	DPCR1:NM_080870:exon2:c.T2449C:p.S817P	NA	NA	NA
chr6	31022928	31022928	C	T	ncRNA_intronic	HCG22	NA	NA	NA	rs62401699	NA
chr6	31022929	31022929	A	G	ncRNA_intronic	HCG22	NA	NA	NA	rs9262625	NA
chr6	31324525	31324525	C	T	exonic	HLA-B	nonsynonymous SNV	HLA-B:NM_005514:exon2:c.G283A:p.A95T	NA	rs1131213	NA
chr6	31324528	31324528	G	T	exonic	HLA-B	nonsynonymous SNV	HLA-B:NM_005514:exon2:c.C280A:p.Q94K	NA	rs1071817	NA
chr6	31324547	31324547	G	C	exonic	HLA-B	nonsynonymous SNV	HLA-B:NM_005514:exon2:c.C261G:p.N87K	M329391;OCCURENCE=1(haematopoietic_and_lymphoid	rs151341177	NA
chr6	31324549	31324549	T	C	exonic	HLA-B	nonsynonymous SNV	HLA-B:NM_005514:exon2:c.A259G:p.N87D	M329392;OCCURENCE=1(haematopoietic_and_lymphoid	rs143633217	NA
chr6	33054656	33054656	A	C	UTR3	HLA-DPB1	NA	NA	NA	rs930	NA
chr6	33054675	33054675	C	G	UTR3	HLA-DPB1	NA	NA	NA	rs9277529	NA
chr6	38139332	38139353	TGTGTGTG	-	UTR3	BTBD9	NA	NA	NA	rs72137261	NA
chr6	41090722	41090722	C	A	ncRNA_exonic	ADCY10P1	NA	NA	NA	NA	NA
chr6	41158908	41158909	TG	-	UTR3	TREML2	NA	NA	NA	NA	NA
chr6	45517740	45517740	T	-	UTR3	RUNX2	NA	NA	NA	rs11320232	NA
chr6	47592425	47592425	-	TGTGTG	UTR3	CD2AP	NA	NA	NA	NA	NA
chr6	47792560	47792560	T	-	UTR3	OPN5	NA	NA	NA	NA	NA
chr6	49460746	49460746	C	A	UTR3	CENPQ	NA	NA	NA	NA	NA
chr6	49460762	49460762	G	T	UTR3	CENPQ	NA	NA	NA	NA	NA
chr6	57398207	57398207	G	T	exonic	PRIM2	unknown	UNKNOWN	NA	rs71214816	NA
chr6	57512841	57512841	A	G	UTR3	PRIM2	NA	NA	NA	rs78256005	NA
chr6	57512850	57512850	C	G	UTR3	PRIM2	NA	NA	NA	rs77911716	NA
chr6	57512889	57512889	C	T	UTR3	PRIM2	NA	NA	NA	rs75351177	NA
chr6	83074365	83074365	C	T	UTR5	TPBG	NA	NA	NA	rs770911	NA
chr6	97592002	97592005	AAAG	-	ncRNA_UTR3	MMS22L	NA	NA	NA	NA	NA
chr6	97592005	97592008	GAGA	-	ncRNA_UTR3	MMS22L	NA	NA	NA	NA	NA
chr6	99724024	99724024	A	-	UTR3	FAXC	NA	NA	NA	rs11307978	NA
chr6	1.07E+08	1.07E+08	C	A	UTR3	PRDM1	NA	NA	NA	NA	NA
chr6	1.07E+08	1.07E+08	C	T	UTR3	PRDM1	NA	NA	NA	NA	NA
chr6	1.07E+08	1.07E+08	A	-	UTR3	BEND3	NA	NA	NA	NA	NA
chr6	1.32E+08	1.32E+08	C	G	exonic	ARG1	nonsynonymous SNV	0045:exon2:c.C82G:p.P28A,ARG1:NM_001244438:exon2:	NA	NA	P
chr6	1.37E+08	1.37E+08	AA	-	UTR3	PDE7B	NA	NA	NA	NA	NA
chr6	1.39E+08	1.39E+08	A	-	UTR3	KIAA1244	NA	NA	NA	NA	NA

chr6	1.44E+08	1.44E+08	T	-	UTR3	PHACTR2	NA	NA	NA	NA	NA
chr6	1.44E+08	1.44E+08	A	T	ncRNA_exonic	HYMAI	NA	NA	NA	rs1474882	NA
chr6	1.49E+08	1.49E+08	A	-	UTR3	SASH1	NA	NA	NA	rs5880793	NA
chr6	1.5E+08	1.5E+08	T	C	exonic	ULBP2	synonymous SNV	ULBP2:NM_025217:exon2:c.T219C:p.P73P	NA	rs149454018	NA
chr6	1.51E+08	1.51E+08	G	-	UTR3	PPP1R14C	NA	NA	NA	rs201167515	NA
chr6	1.52E+08	1.52E+08	-	A	UTR3	CCDC170	NA	NA	NA	NA	NA
chr6	1.58E+08	1.58E+08	C	T	UTR3	ARID1B	NA	NA	NA	NA	NA
chr6	1.59E+08	1.59E+08	A	-	UTR3	GTF2H5	NA	NA	NA	rs3841147	NA
chr6	1.59E+08	1.59E+08	T	-	UTR3	GTF2H5	NA	NA	NA	NA	NA
chr6	1.59E+08	1.59E+08	A	C	exonic	TULP4	nonsynonymous SNV	TULP4:NM_020245:exon13:c.A2306C:p.N769T	ID=COSM400671;OCCURENCE=1(lung)	NA	B
chr6	1.6E+08	1.6E+08	G	T	UTR3	WTAP	NA	NA	NA	NA	NA
chr6	1.6E+08	1.6E+08	G	T	UTR3	WTAP	NA	NA	NA	NA	NA
chr6	1.6E+08	1.6E+08	G	T	UTR3	WTAP	NA	NA	NA	NA	NA
chr6	1.6E+08	1.6E+08	G	T	UTR3	WTAP	NA	NA	NA	NA	NA
chr6	1.61E+08	1.61E+08	C	A	exonic	MAP3K4	nonsynonymous SNV	22:exon4:c.C1909A:p.Q637K,MAP3K4:NM_006724:exon4	NA	NA	D
chr6	1.68E+08	1.68E+08	A	C	ncRNA_exonic	MLLT4-AS1	NA	NA	NA	NA	NA
chr6	1.68E+08	1.68E+08	A	G	exonic	HGC6.3	nonsynonymous SNV	HGC6.3:NM_001129895:exon1:c.T230C:p.F77S	NA	NA	NA
chr7	537506	537506	-	AA	UTR3	PDGFA	NA	NA	NA	NA	NA
chr7	537549	537549	G	A	UTR3	PDGFA	NA	NA	NA	NA	NA
chr7	5660749	5660749	A	C	UTR3	RNF216	NA	NA	NA	NA	NA
chr7	5886611	5886611	C	G	ncRNA_exonic	ZNF815P	NA	NA	NA	rs308084	NA
chr7	19155484	19155485	AA	-	UTR3	TWIST1	NA	NA	NA	NA	NA
chr7	20180426	20180427	AC	-	UTR3	MACC1	NA	NA	NA	rs10555255	NA
chr7	20180471	20180471	C	G	UTR3	MACC1	NA	NA	NA	NA	NA
chr7	22158512	22158512	C	A	UTR3	RAPGEF5	NA	NA	NA	NA	NA
chr7	23749892	23749892	C	G	UTR5	STK31	NA	NA	NA	NA	NA
chr7	27147452	27147453	AA	-	UTR3	HOXA3	NA	NA	NA	NA	NA
chr7	30412409	30412409	A	-	ncRNA_exonic	DKFZP586I1420	NA	NA	NA	NA	NA
chr7	35734529	35734529	C	G	UTR5	HERPUD2	NA	NA	NA	rs78381277	NA
chr7	35734591	35734591	-	G	UTR5	HERPUD2	NA	NA	NA	rs11394396	NA
chr7	44073817	44073817	A	G	ncRNA_exonic	RASA4CP	NA	NA	NA	NA	NA
chr7	44073823	44073823	T	C	ncRNA_exonic	RASA4CP	NA	NA	NA	NA	NA
chr7	44258943	44258943	G	T	UTR3	CAMK2B	NA	NA	NA	rs74582205	NA
chr7	45123081	45123081	C	G	exonic	NACAD	nonsynonymous SNV	NACAD:NM_001146334:exon2:c.G2698C:p.V900L	NA	NA	NA
chr7	45123379	45123379	G	A	exonic	NACAD	synonymous SNV	NACAD:NM_001146334:exon2:c.C2400T:p.S800S	NA	NA	NA
chr7	45123540	45123540	A	G	exonic	NACAD	nonsynonymous SNV	NACAD:NM_001146334:exon2:c.T2239C:p.S747P	NA	NA	NA
chr7	66774667	66774667	G	T	ncRNA_exonic	STAG3L4	NA	NA	NA	NA	NA
chr7	72336469	72336469	T	-	ncRNA_exonic	SPDYE7P	NA	NA	NA	rs58909282	NA
chr7	72338105	72338105	A	-	ncRNA_exonic	SPDYE7P	NA	NA	NA	NA	NA
chr7	72338148	72338148	T	G	ncRNA_exonic	SPDYE7P	NA	NA	NA	NA	NA
chr7	76131725	76131725	T	C	exonic	DTX2	synonymous SNV	!595:exon7:c.T1341C:p.H447H,DTX2:NM_001102594:exo	NA	NA	NA
chr7	77558429	77558429	G	T	exonic	PHTF2	nonsynonymous SNV	;,PHTF2:NM_001127357:exon10:c.G1021T:p.A341S,PHTF	NA	NA	NA
chr7	1E+08	1E+08	A	C	exonic	NYAP1	nonsynonymous SNV	NYAP1:NM_173564:exon4:c.A797C:p.Y266S	NA	NA	D
chr7	1E+08	1E+08	A	C	exonic	NYAP1	synonymous SNV	NYAP1:NM_173564:exon4:c.A843C:p.P281P	NA	NA	NA
chr7	1E+08	1E+08	C	A	exonic	ZAN	unknown	UNKNOWN	NA	NA	NA
chr7	1.01E+08	1.01E+08	C	G	exonic	MUC12	nonsynonymous SNV	MUC12:NM_001164462:exon2:c.C2632G:p.H878D	NA	NA	NA
chr7	1.01E+08	1.01E+08	T	A	exonic	MUC12	nonsynonymous SNV	MUC12:NM_001164462:exon2:c.T2634A:p.H878Q	NA	NA	NA
chr7	1.01E+08	1.01E+08	T	C	exonic	MUC12	synonymous SNV	MUC12:NM_001164462:exon2:c.T2670C:p.D890D	NA	NA	NA
chr7	1.01E+08	1.01E+08	A	C	exonic	MUC12	nonsynonymous SNV	MUC12:NM_001164462:exon2:c.A2682C:p.L894F	NA	NA	NA
chr7	1.01E+08	1.01E+08	A	T	exonic	MUC12	synonymous SNV	MUC12:NM_001164462:exon2:c.A2721T:p.S907S	NA	NA	NA
chr7	1.01E+08	1.01E+08	A	C	exonic	MUC12	nonsynonymous SNV	MUC12:NM_001164462:exon2:c.A2798C:p.Q933P	NA	NA	NA
chr7	1.01E+08	1.01E+08	G	A	exonic	MUC12	nonsynonymous SNV	MUC12:NM_001164462:exon2:c.G2995A:p.A999T	NA	NA	NA
chr7	1.01E+08	1.01E+08	C	G	exonic	MUC12	nonsynonymous SNV	MUC12:NM_001164462:exon2:c.C3004G:p.H1002D	NA	NA	NA
chr7	1.01E+08	1.01E+08	C	G	exonic	MUC12	nonsynonymous SNV	MUC12:NM_001164462:exon2:c.C3067G:p.R1023G	NA	NA	NA
chr7	1.01E+08	1.01E+08	G	A	exonic	MUC12	nonsynonymous SNV	MUC12:NM_001164462:exon2:c.G3076A:p.G1026S	NA	NA	NA
chr7	1.01E+08	1.01E+08	G	C	exonic	MUC12	nonsynonymous SNV	MUC12:NM_001164462:exon2:c.G7226C:p.S2409T	NA	NA	NA
chr7	1.01E+08	1.01E+08	G	A	exonic	MUC12	nonsynonymous SNV	MUC12:NM_001164462:exon2:c.G8069A:p.R2690H	NA	NA	NA
chr7	1.01E+08	1.01E+08	G	C	exonic	MUC12	nonsynonymous SNV	MUC12:NM_001164462:exon2:c.G10142C:p.S3381T	NA	NA	NA
chr7	1.01E+08	1.01E+08	G	A	exonic	MUC12	nonsynonymous SNV	MUC12:NM_001164462:exon2:c.G10238A:p.S3413N	NA	rs200547720	NA
chr7	1.01E+08	1.01E+08	C	T	exonic	MUC12	nonsynonymous SNV	MUC12:NM_001164462:exon2:c.C11036T:p.T3679I	NA	rs201665808	NA
chr7	1.01E+08	1.01E+08	C	G	exonic	MUC12	nonsynonymous SNV	MUC12:NM_001164462:exon2:c.C11327G:p.P3776R	NA	rs201148522	NA
chr7	1.05E+08	1.05E+08	C	A	ncRNA_exonic	LHFPL3-AS2	NA	NA	NA	NA	NA

chr7	1.06E+08	1.06E+08	G	A	exonic	CDHR3	nonsynonymous SNV	CDHR3:NM_152750:exon12:c.G1586A:p.C529Y	NA	rs6967330	NA
chr7	1.07E+08	1.07E+08	A	C	UTR3	COG5	NA	NA	NA	NA	NA
chr7	1.16E+08	1.16E+08	-	A	UTR3	CAV2	NA	NA	NA	NA	NA
chr7	1.2E+08	1.2E+08	A	C	exonic	KCND2	synonymous SNV	KCND2:NM_012281:exon1:c.A873C:p.T291T	NA	NA	NA
chr7	1.21E+08	1.21E+08	A	C	exonic	WNT16	nonsynonymous SNV	.6087:exon2:c.A253C:p.T85P,WNT16:NM_057168:exon2:c	NA	NA	B
chr7	1.22E+08	1.22E+08	G	A	exonic	AASS	nonsynonymous SNV	AASS:NM_005763:exon14:c.C1456T:p.L486F	NA	NA	D
chr7	1.23E+08	1.23E+08	AA	-	UTR3	SLC13A1	NA	NA	NA	NA	NA
chr7	1.23E+08	1.23E+08	T	G	exonic	WASL	synonymous SNV	WASL:NM_003941:exon9:c.A1119C:p.P373P	NA	NA	NA
chr7	1.28E+08	1.28E+08	A	-	UTR3	LEP	NA	NA	NA	rs112344366	NA
chr7	1.3E+08	1.3E+08	TGTGTG	-	UTR3	CPA4	NA	NA	NA	rs146877444	NA
chr7	1.31E+08	1.31E+08	C	A	exonic	PODXL	nonsynonymous SNV	)18111:exon2:c.G488T:p.S163I,PODXL:NM_005397:exon2	NA	NA	P
chr7	1.39E+08	1.39E+08	A	C	UTR3	HIPK2	NA	NA	NA	NA	NA
chr7	1.4E+08	1.4E+08	AA	-	ncRNA_exonic	NDUFB2-AS1	NA	NA	NA	NA	NA
chr7	1.42E+08	1.42E+08	T	C	exonic	PRSS1	synonymous SNV	PRSS1:NM_002769:exon4:c.T567C:p.L189L	NA	NA	NA
chr7	1.43E+08	1.43E+08	A	G	exonic	CTAGE15	synonymous SNV	CTAGE15:NM_001008747:exon1:c.A1839G:p.G613G	NA	NA	NA
chr7	1.43E+08	1.43E+08	T	A	exonic	CTAGE15	synonymous SNV	CTAGE15:NM_001008747:exon1:c.T1869A:p.P623P	NA	NA	NA
chr7	1.43E+08	1.43E+08	T	C	exonic	CTAGE15	nonsynonymous SNV	CTAGE15:NM_001008747:exon1:c.T1915C:p.S639P	NA	NA	NA
chr7	1.44E+08	1.44E+08	A	G	exonic	CTAGE4	synonymous SNV	CTAGE4:NM_198495:exon1:c.T2268C:p.Y756Y	NA	rs202035285	NA
chr7	1.51E+08	1.51E+08	C	A	exonic	ASB10	nonsynonymous SNV	C260F,ASB10:NM_001142460:exon3:c.G779T:p.C260F,AS	NA	NA	B
chr7	1.52E+08	1.52E+08	A	T	UTR3	GALNT11	NA	NA	NA	NA	NA
chr7	1.52E+08	1.52E+08	C	G	exonic	MLL3	nonsynonymous SNV	MLL3:NM_170606:exon16:c.G2755C:p.V919L	D=COSM1131646,COSM1131647;OCCURENCE=1(prostate	rs111791757	B
chr7	1.55E+08	1.55E+08	C	A	ncRNA_exonic	LOC202781	NA	NA	NA	NA	NA
chr8	1729586	1729586	T	G	UTR3	CLN8	NA	NA	NA	NA	NA
chr8	1733665	1733665	T	G	UTR3	CLN8	NA	NA	NA	NA	NA
chr8	2793892	2793892	-	AT	UTR3	CSMD1	NA	NA	NA	rs138563405	NA
chr8	10464990	10464990	T	C	exonic	RP1L1	synonymous SNV	RP1L1:NM_178857:exon4:c.A6618G:p.Q2206Q	NA	NA	NA
chr8	10465086	10465086	T	C	exonic	RP1L1	synonymous SNV	RP1L1:NM_178857:exon4:c.A6522G:p.Q2174Q	NA	NA	NA
chr8	11279878	11279878	T	G	ncRNA_UTR3	FAM167A	NA	NA	NA	rs113139618	NA
chr8	11973535	11973535	A	G	ncRNA_exonic	FAM66D	NA	NA	NA	NA	NA
chr8	12040998	12040998	G	A	UTR3	FAM86B1	NA	NA	NA	rs141787656	NA
chr8	12283157	12283157	T	C	UTR3	FAM86B2	NA	NA	NA	rs2698896	NA
chr8	17079490	17079491	TT	-	UTR3	ZDHHC2	NA	NA	NA	NA	NA
chr8	17579505	17579505	A	-	UTR5	MTUS1	NA	NA	NA	rs34302917	NA
chr8	20106557	20106557	A	G	UTR3	LZTS1	NA	NA	NA	NA	NA
chr8	23536561	23536563	TTT	-	UTR3	NKX3-1	NA	NA	NA	rs140739091	NA
chr8	28205758	28205758	A	C	UTR3	ZNF395	NA	NA	NA	NA	NA
chr8	41481358	41481358	T	-	UTR3	AGPAT6	NA	NA	NA	NA	NA
chr8	41788246	41788246	A	T	UTR3	KAT6A	NA	NA	NA	NA	NA
chr8	52733242	52733242	C	T	exonic	PCMTD1	nonsynonymous SNV	PCMTD1:NM_052937:exon6:c.G743A:p.R248H	NA	rs79820636	D
chr8	57307835	57307835	C	A	intergenic	dist=74594),PENK(d	NA	NA	NA	NA	NA
chr8	75279333	75279333	-	AAA	UTR3	GDAP1	NA	NA	NA	NA	NA
chr8	86572249	86572249	A	G	UTR3	REXO1L1	NA	NA	NA	rs28564465	NA
chr8	88884730	88884730	A	-	UTR3	DCAF4L2	NA	NA	NA	rs57457601	NA
chr8	92971184	92971184	T	-	UTR3	RUNX1T1	NA	NA	NA	NA	NA
chr8	98787930	98787930	-	GAGCTCCA	UTR5	LAPTM4B	NA	NA	NA	rs146027280	NA
chr8	1.02E+08	1.02E+08	G	A	exonic	PABPC1	nonsynonymous SNV	PABPC1:NM_002568:exon11:c.C1477T:p.R493C	NA	rs62513924	P
chr8	1.16E+08	1.16E+08	C	A	UTR3	TRPS1	NA	NA	NA	NA	NA
chr8	1.23E+08	1.23E+08	C	T	ncRNA_UTR5	HAS2	NA	NA	NA	NA	NA
chr8	1.23E+08	1.23E+08	C	T	ncRNA_UTR5	HAS2	NA	NA	NA	NA	NA
chr8	1.31E+08	1.31E+08	T	C	exonic	ASAP1	nonsynonymous SNV	:exon24:c.A2182G:p.I728V,ASAP1:NM_001247996:exon2	ID=COSM150541;OCCURENCE=1(stomach)	rs966185	B
chr8	1.32E+08	1.32E+08	C	A	exonic	ADCY8	nonsynonymous SNV	ADCY8:NM_001115:exon4:c.G1252T:p.A418S	NA	NA	D
chr8	1.34E+08	1.34E+08	TT	-	UTR3	SLA	NA	NA	NA	NA	NA
chr8	1.46E+08	1.46E+08	C	A	UTR3	ZNF250	NA	NA	NA	NA	NA
chr8	1.46E+08	1.46E+08	T	A	ncRNA_exonic	TMED10P1	NA	NA	NA	NA	NA
chr9	117998	117998	G	T	exonic	FOXD4	nonsynonymous SNV	FOXD4:NM_207305:exon1:c.C122A:p.A41E	ID=COSM226644;OCCURENCE=1(skin)	rs66612967	B
chr9	711797	711797	A	G	exonic	KANK1	nonsynonymous SNV	xon3:c.A1031G:p.E344G,KANK1:NM_001256877:exon4:c.	NA	rs76363411	D
chr9	5128081	5128086	TGTGTG	-	UTR3	JAK2	NA	NA	NA	rs139964957	NA
chr9	14083479	14083479	A	-	UTR3	NFIB	NA	NA	NA	NA	NA
chr9	33043991	33043992	AA	-	UTR3	SMU1	NA	NA	NA	rs141620295	NA
chr9	33111314	33111314	T	A	UTR3	B4GALT1	NA	NA	NA	NA	NA
chr9	33385667	33385667	A	G	exonic	AQP7	synonymous SNV	AQP7:NM_001170:exon7:c.T723C:p.G241G	NA	rs79779983	NA
chr9	33385698	33385698	A	G	exonic	AQP7	nonsynonymous SNV	AQP7:NM_001170:exon7:c.T692C:p.L231P	NA	rs145516206	B

chr9	33385712	33385712	G	A	exonic	AQP7	synonymous SNV	AQP7:NM_001170:exon7:c.C678T:p.N226N	NA	rs115575789	NA
chr9	33385733	33385733	C	T	exonic	AQP7	nonsynonymous SNV	AQP7:NM_001170:exon7:c.G657A:p.M219I	=COSM1180629;OCCURENCE=1(upper_aerodigestive_tra	rs201117022	B
chr9	33385740	33385740	A	T	exonic	AQP7	nonsynonymous SNV	AQP7:NM_001170:exon7:c.T650A:p.L217H	NA	rs201773300	B
chr9	66500435	66500435	C	A	ncRNA_exonic	TGER4P2-CDK2AP2F	NA	NA	NA	rs200229634	NA
chr9	69202059	69202059	T	C	UTR5	FOXD4L6	NA	NA	NA	NA	NA
chr9	69424311	69424311	C	A	UTR3	ANKRD20A4	NA	NA	NA	rs3866289	NA
chr9	70177559	70177559	A	G	exonic	FOXD4L5	nonsynonymous SNV	FOXD4L5:NM_001126334:exon1:c.T425C:p.F142S	NA	NA	NA
chr9	70912543	70912543	A	T	exonic	CBWD3	nonsynonymous SNV	CBWD3:NM_201453:exon12:c.A864T:p.E288D	NA	rs1127334	B
chr9	99538299	99538299	C	A	exonic	ZNF510	nonsynonymous SNV	ZNF510:NM_014930:exon2:c.G53T:p.G18V	NA	NA	D
chr9	99672115	99672115	C	T	ncRNA_exonic	LOC441454	NA	NA	NA	rs201340221	NA
chr9	1.02E+08	1.02E+08	G	T	UTR3	TGFBR1	NA	NA	NA	rs199545273	NA
chr9	1.04E+08	1.04E+08	T	-	UTR3	LPPR1	NA	NA	NA	rs60292880	NA
chr9	1.1E+08	1.1E+08	A	C	exonic	ZNF462	nonsynonymous SNV	ZNF462:NM_021224:exon3:c.A4303C:p.T1435P	NA	NA	B
chr9	1.12E+08	1.12E+08	T	-	UTR3	TMEM245	NA	NA	NA	NA	NA
chr9	1.16E+08	1.16E+08	G	T	UTR3	SNX30	NA	NA	NA	rs113416907	NA
chr9	1.31E+08	1.31E+08	T	C	exonic	CERCAM	nonsynonymous SNV	CERCAM:NM_016174:exon5:c.T590C:p.F197S	NA	NA	P
chr9	1.31E+08	1.31E+08	A	G	exonic	CERCAM	synonymous SNV	CERCAM:NM_016174:exon5:c.A672G:p.E224E	NA	NA	NA
chr9	1.32E+08	1.32E+08	C	G	UTR3	PPP2R4	NA	NA	NA	NA	NA
chr9	1.32E+08	1.32E+08	C	G	UTR3	PPP2R4	NA	NA	NA	NA	NA
chr9	1.32E+08	1.32E+08	T	A	UTR3	PPP2R4	NA	NA	NA	NA	NA
chr9	1.33E+08	1.33E+08	A	-	UTR3	FNBP1	NA	NA	NA	rs5900873	NA
chr9	1.34E+08	1.34E+08	C	T	UTR5	PPAPDC3	NA	NA	NA	rs7041276	NA
chr9	1.35E+08	1.35E+08	C	T	UTR3	NTNG2	NA	NA	NA	NA	NA
chr9	1.36E+08	1.36E+08	T	G	UTR3	ABO	NA	NA	NA	NA	NA
chr9	1.36E+08	1.36E+08	G	T	UTR5	ADAMTSL2	NA	NA	NA	NA	NA
chr9	1.4E+08	1.4E+08	A	G	exonic	KIAA1984	nonsynonymous SNV	KIAA1984:NM_001039374:exon10:c.A1070G:p.E357G	NA	NA	P
chr9	1.41E+08	1.41E+08	T	C	ncRNA_exonic	TUBBP5	NA	NA	NA	rs28578364	NA
chr10	1209490	1209490	A	G	ncRNA_exonic	LINC00200	NA	NA	NA	rs10903391	NA
chr10	1209495	1209495	G	C	ncRNA_exonic	LINC00200	NA	NA	NA	rs11250303	NA
chr10	6157836	6157839	AAAA	-	UTR3	RBM17	NA	NA	NA	rs71390125	NA
chr10	17840303	17840303	T	C	UTR3	TMEM236	NA	NA	NA	rs34022011	NA
chr10	21803421	21803421	-	A	UTR3	SKIDA1	NA	NA	NA	NA	NA
chr10	21807470	21807470	A	-	UTR5	SKIDA1	NA	NA	NA	rs111265769	NA
chr10	21807780	21807781	TT	-	UTR5	SKIDA1	NA	NA	NA	NA	NA
chr10	24873151	24873152	AT	-	UTR3	ARHGAP21	NA	NA	NA	rs35918968	NA
chr10	29801764	29801764	T	C	exonic	SVIL	nonsynonymous SNV	:exon15:c.A2138G:p.E713G,SVIL:NM_021738:exon17:c.A	NA	NA	D
chr10	45496046	45496046	C	A	exonic	C10orf25	stopgain SNV	C10orf25:NM_001039380:exon1:c.G262T:p.E88X	NA	NA	NA
chr10	46963951	46963951	G	A	exonic	SYT15	synonymous SNV	912:exon7:c.C1012T:p.L338L,SYT15:NM_181519:exon7:c.	NA	rs55945664	NA
chr10	49812917	49812917	T	C	UTR5	ARHGAP22	NA	NA	NA	rs17010948	NA
chr10	50223165	50223166	TT	-	UTR3	VSTM4	NA	NA	NA	NA	NA
chr10	50966445	50966445	C	A	exonic	OGDHL	nonsynonymous SNV	.143996:exon2:c.G194T:p.S65I,OGDHL:NM_018245:exon2	NA	NA	D
chr10	51226439	51226439	T	G	exonic	AGAP8	nonsynonymous SNV	81N,AGAP8:NM_001276344:exon7:c.A543C:p.K181N,AG/	NA	rs201124655	B
chr10	51748584	51748584	A	G	exonic	AGAP6	nonsynonymous SNV	AGAP6:NM_001077665:exon1:c.A109G:p.R37G	NA	rs200504295	NA
chr10	60474828	60474828	A	-	ncRNA_exonic	FAM133CP	NA	NA	NA	NA	NA
chr10	62544601	62544601	G	T	exonic	CDK1	nonsynonymous SNV	)1170407:exon3:c.G176T:p.R59L,CDK1:NM_001786:exon3	NA	NA	B
chr10	63955366	63955381	GAAGGAAG	-	UTR3	RTKN2	NA	NA	NA	NA	NA
chr10	70043500	70043500	-	TG	UTR3	PBLD	NA	NA	NA	NA	NA
chr10	71267385	71267385	G	T	UTR3	TSPAN15	NA	NA	NA	NA	NA
chr10	71333106	71333106	G	C	UTR5	NEUROG3	NA	NA	NA	rs10998862	NA
chr10	72328155	72328155	T	-	UTR3	PALD1	NA	NA	NA	rs71012215	NA
chr10	79741920	79741920	T	G	exonic	POLR3A	nonsynonymous SNV	POLR3A:NM_007055:exon28:c.A3751C:p.T1251P	NA	NA	P
chr10	81587323	81587323	A	-	ncRNA_exonic	LOC642361	NA	NA	NA	NA	NA
chr10	87359676	87359676	-	CTCACA	ncRNA_UTR3	GRID1	NA	NA	NA	NA	NA
chr10	88677008	88677008	A	T	exonic	BMPR1A	nonsynonymous SNV	BMPR1A:NM_004329:exon9:c.A793T:p.T265S	NA	NA	P
chr10	88677045	88677045	T	A	exonic	BMPR1A	nonsynonymous SNV	BMPR1A:NM_004329:exon9:c.T830A:p.I277N	NA	NA	D
chr10	89125529	89125529	G	C	ncRNA_exonic	NUTM2D	NA	NA	NA	rs2767120	D
chr10	89619806	89619806	G	A	UTR3	KLLN	NA	NA	NA	NA	NA
chr10	89726612	89726612	G	A	UTR3	PTEN	NA	NA	NA	NA	NA
chr10	99192863	99192863	-	ATAA	UTR3	PGAM1	NA	NA	NA	NA	NA
chr10	99371771	99371771	C	T	UTR3	HOGA1	NA	NA	NA	rs1977642	NA
chr10	1.02E+08	1.02E+08	G	T	UTR3	HIF1AN	NA	NA	NA	rs75780010	NA
chr10	1.02E+08	1.02E+08	G	T	UTR3	HIF1AN	NA	NA	NA	NA	NA

chr10	1.16E+08	1.16E+08	T	C	UTR3	PLEKHS1	NA	NA	NA	NA	NA
chr10	1.18E+08	1.18E+08	-	TG	UTR3	GFRA1	NA	NA	NA	NA	NA
chr10	1.18E+08	1.18E+08	T	A	UTR3	GFRA1	NA	NA	NA	rs142941024	NA
chr10	1.18E+08	1.18E+08	T	A	UTR3	GFRA1	NA	NA	NA	rs145714787	NA
chr10	1.21E+08	1.21E+08	C	A	UTR3	TIAL1	NA	NA	NA	rs111740135	NA
chr10	1.26E+08	1.26E+08	A	C	UTR3	FAM53B	NA	NA	NA	rs1057103	NA
chr10	1.27E+08	1.27E+08	T	A	UTR3	CTBP2	NA	NA	NA	NA	NA
chr10	1.27E+08	1.27E+08	T	A	UTR3	CTBP2	NA	NA	NA	NA	NA
chr10	1.27E+08	1.27E+08	T	-	exonic	CTBP2	frameshift deletion	83914:exon3:c.57delA:p.E19fs,CTBP2:NM_001329:exon3	NA	NA	NA
chr10	1.28E+08	1.28E+08	C	T	UTR5	FANK1	NA	NA	NA	rs79612842	NA
chr10	1.28E+08	1.28E+08	G	C	UTR5	FANK1	NA	NA	NA	rs80288044	NA
chr10	1.28E+08	1.28E+08	C	A	UTR5	FANK1	NA	NA	NA	rs74162870	NA
chr10	1.3E+08	1.3E+08	T	C	UTR3	FOXI2	NA	NA	NA	NA	NA
chr10	1.3E+08	1.3E+08	T	C	UTR3	FOXI2	NA	NA	NA	NA	NA
chr10	1.34E+08	1.34E+08	A	G	exonic	DPYSL4	nonsynonymous SNV	DPYSL4:NM_006426:exon8:c.A773G:p.K258R	NA	NA	B
chr10	1.34E+08	1.34E+08	A	T	UTR3	LRRC27	NA	NA	NA	rs113666011	NA
chr10	1.35E+08	1.35E+08	G	A	exonic	FRG2B	synonymous SNV	FRG2B:NM_001080998:exon3:c.C312T:p.D104D	NA	NA	NA
chr11	128770	128770	C	T	ncRNA_exonic	LOC100133161	NA	NA	NA	NA	NA
chr11	1017277	1017277	A	G	exonic	MUC6	nonsynonymous SNV	MUC6:NM_005961:exon31:c.T5524C:p.S1842P	NA	rs111373859	NA
chr11	1017440	1017440	C	T	exonic	MUC6	synonymous SNV	MUC6:NM_005961:exon31:c.G5361A:p.S1787S	NA	rs78729877	NA
chr11	1018228	1018228	G	A	exonic	MUC6	synonymous SNV	MUC6:NM_005961:exon31:c.C4573T:p.L1525L	NA	NA	NA
chr11	1018290	1018290	G	A	exonic	MUC6	nonsynonymous SNV	MUC6:NM_005961:exon31:c.C4511T:p.P1504L	NA	NA	NA
chr11	1018341	1018341	G	A	exonic	MUC6	nonsynonymous SNV	MUC6:NM_005961:exon31:c.C4460T:p.P1487L	NA	rs79748612	NA
chr11	1090928	1090928	A	C	exonic	MUC2	nonsynonymous SNV	MUC2:NM_002457:exon28:c.A3823C:p.T1275P	NA	NA	NA
chr11	1090937	1090937	A	C	exonic	MUC2	nonsynonymous SNV	MUC2:NM_002457:exon28:c.A3832C:p.T1278P	NA	NA	NA
chr11	1093452	1093452	G	A	exonic	MUC2	synonymous SNV	MUC2:NM_002457:exon30:c.G5271A:p.P1757P	D=COSM1127915,COSM1127916;OCCURENCE=1(prostate	rs34136803	NA
chr11	1264381	1264381	A	C	exonic	MUC5B	nonsynonymous SNV	MUC5B:NM_002458:exon31:c.A6271C:p.T2091P	NA	NA	NA
chr11	1269500	1269500	T	C	exonic	MUC5B	nonsynonymous SNV	MUC5B:NM_002458:exon31:c.T11390C:p.I3797T	NA	NA	NA
chr11	1271763	1271763	A	C	exonic	MUC5B	synonymous SNV	MUC5B:NM_002458:exon31:c.A13653C:p.T4551T	:COSM1152309,COSM924786;OCCURENCE=1(endometriu	NA	NA
chr11	1605892	1605892	A	G	exonic	KRTAP5-1	synonymous SNV	KRTAP5-1:NM_001005922:exon1:c.T588C:p.G196G	NA	NA	NA
chr11	1605916	1605916	A	G	exonic	KRTAP5-1	synonymous SNV	KRTAP5-1:NM_001005922:exon1:c.T564C:p.G188G	NA	NA	NA
chr11	1605919	1605919	G	A	exonic	KRTAP5-1	synonymous SNV	KRTAP5-1:NM_001005922:exon1:c.C561T:p.C187C	NA	NA	NA
chr11	1606166	1606166	G	C	exonic	KRTAP5-1	nonsynonymous SNV	KRTAP5-1:NM_001005922:exon1:c.C314G:p.S105C	NA	NA	NA
chr11	2696063	2696063	A	-	ncRNA_exonic	KCNQ1OT1	NA	NA	NA	rs34219164	NA
chr11	2720927	2720927	C	T	ncRNA_exonic	KCNQ1OT1	NA	NA	NA	rs12360708	NA
chr11	6633033	6633033	A	C	exonic	TAF10	synonymous SNV	TAF10:NM_006284:exon2:c.T249G:p.G83G	NA	NA	NA
chr11	9304488	9304488	A	-	UTR3	TMEM41B	NA	NA	NA	rs56313114	NA
chr11	21555994	21555994	C	A	exonic	NELL1	nonsynonymous SNV	NELL1:NM_006157:exon16:c.C1720A:p.H574N	NA	NA	D
chr11	26690764	26690764	T	C	UTR3	SLC5A12	NA	NA	NA	NA	NA
chr11	33757941	33757941	A	G	UTR5	CD59	NA	NA	NA	rs13614	NA
chr11	35829803	35829803	G	A	UTR3	TRIM44	NA	NA	NA	NA	NA
chr11	46383244	46383244	C	A	UTR5	DGKZ	NA	NA	NA	NA	NA
chr11	56959046	56959046	T	G	UTR3	LRRC55	NA	NA	NA	NA	NA
chr11	56959052	56959052	T	G	UTR3	LRRC55	NA	NA	NA	NA	NA
chr11	56959056	56959056	A	G	UTR3	LRRC55	NA	NA	NA	NA	NA
chr11	56959061	56959061	T	G	UTR3	LRRC55	NA	NA	NA	NA	NA
chr11	61405598	61405598	-	AAA	ncRNA_exonic	RPLP0P2	NA	NA	NA	NA	NA
chr11	61560372	61560372	C	T	UTR5	FEN1	NA	NA	NA	NA	NA
chr11	61567669	61567669	T	-	UTR3	FADS1	NA	NA	NA	NA	NA
chr11	64004692	64004692	C	T	exonic	VEGFB	synonymous SNV	43733:exon5:c.C408T:p.D136D,VEGFB:NM_003377:exon5	ID=COSM147267;OCCURENCE=1(stomach)	rs12366035	NA
chr11	65143541	65143543	AAA	-	UTR3	SLC25A45	NA	NA	NA	NA	NA
chr11	66393069	66393069	G	C	exonic	RBM14	synonymous SNV	RBM14:NM_006328:exon2:c.G1722C:p.P574P	NA	NA	NA
chr11	70281360	70281362	TTT	-	UTR3	CTTN	NA	NA	NA	rs71992777	NA
chr11	71238760	71238760	A	C	exonic	KRTAP5-7	synonymous SNV	KRTAP5-7:NM_001012503:exon1:c.A414C:p.S138S	NA	NA	NA
chr11	71708250	71708251	GT	-	UTR3	RNF121	NA	NA	NA	rs3841467	NA
chr11	71715015	71715015	-	G	exonic	NUMA1	frameshift insertion	NUMA1:NM_006185:exon26:c.6254_6255insC:p.R2085fs	NA	NA	NA
chr11	73472134	73472134	A	C	UTR5	RAB6A	NA	NA	NA	rs6592533	NA
chr11	89701844	89701844	T	C	exonic	TRIM64	nonsynonymous SNV	TRIM64:NM_001136486:exon1:c.T173C:p.I58T	NA	rs145750208	NA
chr11	94803548	94803548	G	A	UTR3	SRSF8	NA	NA	NA	rs201925344	NA
chr11	1.01E+08	1.01E+08	T	G	exonic	PGR	synonymous SNV	421P,PGR:NM_001202474:exon1:c.A771C:p.P257P,PGR:N	NA	NA	NA
chr11	1.02E+08	1.02E+08	T	A	UTR3	KIAA1377	NA	NA	NA	NA	NA
chr11	1.03E+08	1.03E+08	T	G	UTR3	DCUN1D5	NA	NA	NA	NA	NA

chr11	1.03E+08	1.03E+08	T	C	exonic	DYNC2H1	synonymous SNV	on83:c.T12096C:p.D4032D,DYNC2H1:NM_001080463:exo	NA	rs2566913	NA
chr11	1.06E+08	1.06E+08	A	C	UTR3	GRIA4	NA	NA	NA	rs188861527	NA
chr11	1.08E+08	1.08E+08	CA	CGA	UTR3	SLC35F2	NA	NA	NA	NA	NA
chr11	1.08E+08	1.08E+08	A	C	UTR3	SLC35F2	NA	NA	NA	rs111576438	NA
chr11	1.1E+08	1.1E+08	A	-	UTR3	ARHGAP20	NA	NA	NA	rs35306856	NA
chr11	1.12E+08	1.12E+08	G	T	UTR3	C11orf57	NA	NA	NA	NA	NA
chr11	1.13E+08	1.13E+08	C	T	UTR3	NCAM1	NA	NA	NA	NA	NA
chr11	1.18E+08	1.18E+08	CA	-	UTR3	FXYD6	NA	NA	NA	rs57385031	NA
chr11	1.19E+08	1.19E+08	A	C	UTR3	DDX6	NA	NA	NA	NA	NA
chr11	1.2E+08	1.2E+08	C	T	UTR3	POU2F3	NA	NA	NA	rs4938797	NA
chr11	1.22E+08	1.22E+08	-	A	ncRNA_exonic	MIR100HG	NA	NA	NA	NA	NA
chr11	1.26E+08	1.26E+08	C	A	exonic	CDON	stopgain SNV	43597:exon6:c.G928T:p.E310X,CDON:NM_016952:exon6:	NA	NA	NA
chr12	863076	863076	T	C	exonic	WNK1	synonymous SNV	14823:exon1:c.T345C:p.A115A,WNK1:NM_018979:exon1	NA	NA	NA
chr12	1021912	1021912	C	-	UTR3	RAD52	NA	NA	NA	rs71441627	NA
chr12	3048755	3048755	A	G	UTR3	TULP3	NA	NA	NA	rs11062424	NA
chr12	5021439	5021439	G	T	exonic	KCNA1	nonsynonymous SNV	KCNA1:NM_000217:exon2:c.G895T:p.V299F	NA	NA	D
chr12	8353374	8353374	G	A	ncRNA_exonic	FAM66C	NA	NA	NA	rs200171719	NA
chr12	8927164	8927164	G	T	UTR3	RIMKLB	NA	NA	NA	NA	NA
chr12	9446339	9446339	C	T	ncRNA_exonic	LOC642846	NA	NA	NA	rs202101079	NA
chr12	9800777	9800777	T	G	ncRNA_exonic	LOC374443	NA	NA	NA	NA	NA
chr12	9849140	9849145	AAAGTA	-	UTR3	CLEC2D	NA	NA	NA	rs150209689	NA
chr12	11420895	11420895	C	T	exonic	PRB3	synonymous SNV	PRB3:NM_006249:exon3:c.G288A:p.P96P	NA	NA	NA
chr12	11421004	11421004	C	G	exonic	PRB3	nonsynonymous SNV	PRB3:NM_006249:exon3:c.G179C:p.R60P	NA	rs71455364	NA
chr12	11461440	11461440	T	C	exonic	PRB4	synonymous SNV	PRB4:NM_002723:exon3:c.A477G:p.P159P	NA	NA	NA
chr12	11461444	11461444	T	G	exonic	PRB4	nonsynonymous SNV	PRB4:NM_002723:exon3:c.A473C:p.H158P	NA	rs59774427	NA
chr12	11461490	11461490	T	C	exonic	PRB4	nonsynonymous SNV	PRB4:NM_002723:exon3:c.A427G:p.R143G	NA	rs113771252	NA
chr12	11461738	11461738	G	C	exonic	PRB4	nonsynonymous SNV	261399:exon3:c.C179G:p.P60R,PRB4:NM_002723:exon3:c	NA	rs79562958	NA
chr12	11546258	11546258	G	T	exonic	PRB2	nonsynonymous SNV	PRB2:NM_006248:exon3:c.C754A:p.Q252K	NA	NA	NA
chr12	12045145	12045146	TT	-	ncRNA_UTR3	ETV6	NA	NA	NA	NA	NA
chr12	19529083	19529083	A	-	UTR3	PLEKHA5	NA	NA	NA	NA	NA
chr12	22839786	22839793	TTTTTTTT	-	UTR3	ETNK1	NA	NA	NA	rs57569708	NA
chr12	39047480	39047480	C	A	UTR3	CPNE8	NA	NA	NA	NA	NA
chr12	50017647	50017647	T	G	intronic	PRPF40B	NA	NA	NA	NA	NA
chr12	50017657	50017657	T	G	intronic	PRPF40B	NA	NA	NA	NA	NA
chr12	50745739	50745739	A	G	exonic	FAM186A	nonsynonymous SNV	FAM186A:NM_001145475:exon4:c.T4876C:p.S1626P	NA	NA	NA
chr12	50746927	50746927	T	G	exonic	FAM186A	nonsynonymous SNV	FAM186A:NM_001145475:exon4:c.A3688C:p.T1230P	NA	NA	NA
chr12	50747248	50747248	A	G	exonic	FAM186A	nonsynonymous SNV	FAM186A:NM_001145475:exon4:c.T3367C:p.F1123L	NA	NA	NA
chr12	51891648	51891648	C	A	intronic	SLC4A8	NA	NA	NA	NA	NA
chr12	52374856	52374856	T	G	exonic	ACVR1B	synonymous SNV	228G,ACVR1B:NM_020327:exon4:c.T528G:p.G176G,ACVI	NA	NA	NA
chr12	52845601	52845601	C	T	exonic	KRT6B	nonsynonymous SNV	KRT6B:NM_005555:exon1:c.G262A:p.G88R	NA	rs201122967	NA
chr12	52845665	52845665	G	T	exonic	KRT6B	synonymous SNV	KRT6B:NM_005555:exon1:c.C198A:p.G66G	NA	rs141114189	NA
chr12	53162729	53162729	A	C	exonic	KRT76	nonsynonymous SNV	KRT76:NM_015848:exon9:c.T1685G:p.V562G	NA	NA	NA
chr12	53810134	53810135	TT	-	UTR3	SP1	NA	NA	NA	NA	NA
chr12	54943322	54943322	A	G	UTR5	PDE1B	NA	NA	NA	NA	NA
chr12	54943340	54943340	A	G	UTR5	PDE1B	NA	NA	NA	NA	NA
chr12	54943354	54943354	A	G	UTR5	PDE1B	NA	NA	NA	NA	NA
chr12	56810470	56810470	G	T	UTR3	TIMELESS	NA	NA	NA	NA	NA
chr12	57487230	57487230	C	A	exonic	NAB2	nonsynonymous SNV	NAB2:NM_005967:exon6:c.C1317A:p.D439E	NA	NA	NA
chr12	58146158	58146158	G	T	UTR5	CDK4	NA	NA	NA	NA	NA
chr12	58146161	58146161	G	T	UTR5	CDK4	NA	NA	NA	NA	NA
chr12	64541138	64541138	A	C	UTR3	SRGAP1	NA	NA	NA	NA	NA
chr12	64541205	64541205	A	C	UTR3	SRGAP1	NA	NA	NA	NA	NA
chr12	66218519	66218519	C	G	ncRNA_UTR5	HMGA2	NA	NA	NA	rs12425965	NA
chr12	70214497	70214497	G	T	UTR3	RAB3IP	NA	NA	NA	rs11177881	NA
chr12	72079926	72079926	T	G	UTR5	TMEM19	NA	NA	NA	NA	NA
chr12	89914886	89914886	A	T	UTR3	ALNT4,POC1B-GALN	NA	NA	NA	rs10745507	NA
chr12	96361580	96361580	G	T	exonic	AMDHD1	stopgain SNV	AMDHD1:NM_152435:exon9:c.G1240T:p.E414X	NA	NA	NA
chr12	1.01E+08	1.01E+08	-	T	UTR3	SLC17A8	NA	NA	NA	NA	NA
chr12	1.07E+08	1.07E+08	T	-	UTR3	CKAP4	NA	NA	NA	rs71442016	NA
chr12	1.09E+08	1.09E+08	C	G	exonic	TMEM119	synonymous SNV	TMEM119:NM_181724:exon2:c.G180C:p.P60P	NA	NA	NA
chr12	1.09E+08	1.09E+08	T	G	exonic	TMEM119	synonymous SNV	TMEM119:NM_181724:exon2:c.A168C:p.P56P	NA	NA	NA
chr12	1.09E+08	1.09E+08	C	G	exonic	TMEM119	synonymous SNV	TMEM119:NM_181724:exon2:c.G165C:p.P55P	NA	NA	NA

chr12	1.14E+08	1.14E+08	T	G	exonic	TPCN1	nonsynonymous SNV	exon18:c.T1463G:p.V488G,TPCN1:NM_001143819:exon1	NA	NA	P
chr12	1.17E+08	1.17E+08	T	A	ncRNA_exonic	LINC00173	NA	NA	NA	NA	NA
chr12	1.18E+08	1.18E+08	TGCTCTGC	-	UTR3	KSR2	NA	NA	NA	NA	NA
chr12	1.2E+08	1.2E+08	G	A	UTR3	CIT	NA	NA	NA	rs139790082	NA
chr12	1.21E+08	1.21E+08	G	A	UTR3	MSI1	NA	NA	NA	NA	NA
chr12	1.21E+08	1.21E+08	C	T	UTR3	MSI1	NA	NA	NA	NA	NA
chr12	1.29E+08	1.29E+08	G	A	intronic	SLC15A4	NA	NA	NA	rs200176633	NA
chr12	1.33E+08	1.33E+08	C	A	exonic	ANKLE2	nonsynonymous SNV	ANKLE2:NM_015114:exon13:c.G2619T:p.W873C	NA	NA	D
chr12	1.34E+08	1.34E+08	A	C	UTR3	ZNF268	NA	NA	NA	NA	NA
chr12	1.34E+08	1.34E+08	A	C	UTR3	ZNF268	NA	NA	NA	NA	NA
chr13	19428581	19428581	T	-	ncRNA_exonic	ANKRD20A9P	NA	NA	NA	NA	NA
chr13	19436148	19436148	C	G	ncRNA_exonic	ANKRD20A9P	NA	NA	NA	rs112815656	NA
chr13	19436179	19436179	A	G	ncRNA_exonic	ANKRD20A9P	NA	NA	NA	rs112011321	NA
chr13	25875755	25875755	A	C	UTR5	NUPL1	NA	NA	NA	NA	NA
chr13	28867509	28867509	C	T	UTR3	PAN3	NA	NA	NA	NA	NA
chr13	28874659	28874664	CACACA	-	UTR3	FLT1	NA	NA	NA	rs147176157	NA
chr13	30780063	30780100	TGTGTGTG	-	UTR3	KATNAL1	NA	NA	NA	NA	NA
chr13	49852512	49852512	G	T	exonic	CDADC1	nonsynonymous SNV	3478:exon7:c.G1077T:p.M359I,CDADC1:NM_030911:exon	NA	NA	D
chr13	58209060	58209060	T	C	exonic	PCDH17	nonsynonymous SNV	PCDH17:NM_001040429:exon1:c.T2380C:p.S794P	NA	NA	D
chr13	66878392	66878392	C	A	UTR3	PCDH9	NA	NA	NA	rs200806980	NA
chr13	75861158	75861158	C	A	exonic	TBC1D4	nonsynonymous SNV	TBC1D4:NM_014832:exon21:c.G3667T:p.A1223S	NA	NA	B
chr13	84455886	84455886	G	A	UTR5	SLITRK1	NA	NA	NA	rs202038566	NA
chr13	96485306	96485306	C	A	exonic	UGGT2	nonsynonymous SNV	UGGT2:NM_020121:exon38:c.G4403T:p.C1468F	NA	NA	D
chr13	1.01E+08	1.01E+08	-	T	UTR3	ZIC2	NA	NA	NA	rs34544768	NA
chr13	1.04E+08	1.04E+08	-	T	ncRNA_exonic	METTL21EP	NA	NA	NA	rs34557658	NA
chr13	1.07E+08	1.07E+08	A	-	UTR3	ARGLU1	NA	NA	NA	NA	NA
chr13	1.09E+08	1.09E+08	GA	-	UTR5	FAM155A	NA	NA	NA	rs35939139	NA
chr13	1.14E+08	1.14E+08	C	A	exonic	F10	nonsynonymous SNV	F10:NM_000504:exon1:c.C7A:p.R3S	NA	NA	P
chr14	22102293	22102293	C	T	exonic	OR10G2	nonsynonymous SNV	OR10G2:NM_001005466:exon1:c.G706A:p.A236T	NA	rs41307110	B
chr14	23518270	23518270	C	A	exonic	CDH24	synonymous SNV	35:exon11:c.G1812T:p.L604L,CDH24:NM_022478:exon12	NA	NA	NA
chr14	45397887	45397887	T	-	UTR3	KLHL28	NA	NA	NA	NA	NA
chr14	47310822	47310822	T	C	UTR3	MDGA2	NA	NA	NA	NA	NA
chr14	50712980	50712980	A	G	UTR3	L2HGDH	NA	NA	NA	rs74541550	NA
chr14	55494090	55494090	G	A	UTR5	SOCS4	NA	NA	NA	rs77678033	NA
chr14	55494091	55494091	G	A	UTR5	SOCS4	NA	NA	NA	rs77911976	NA
chr14	57114800	57114800	A	-	UTR3	TMEM260	NA	NA	NA	NA	NA
chr14	59835968	59835968	C	A	UTR3	DAAM1	NA	NA	NA	rs74057123	NA
chr14	59954397	59954397	C	A	exonic	JKAMP	stopgain SNV	098625:exon3:c.C84A:p.C28X,JKAMP:NM_016475:exon3	NA	NA	NA
chr14	60759368	60759368	T	-	UTR3	PPM1A	NA	NA	NA	NA	NA
chr14	61112736	61112737	TT	-	UTR3	SIX1	NA	NA	NA	NA	NA
chr14	65215655	65215655	T	-	UTR3	SPTB	NA	NA	NA	rs140208257	NA
chr14	67852376	67852376	T	-	UTR3	EIF2S1	NA	NA	NA	rs71727390	NA
chr14	73137344	73137345	TC	-	UTR3	DPF3	NA	NA	NA	NA	NA
chr14	77599159	77599159	-	TCTCACAC	UTR3	ZDHHC22	NA	NA	NA	NA	NA
chr14	78045446	78045446	C	A	exonic	SPTLC2	nonsynonymous SNV	SPTLC2:NM_004863:exon3:c.G334T:p.V112L	NA	NA	B
chr14	89627942	89627942	C	T	UTR3	FOXN3	NA	NA	NA	rs7156172	NA
chr14	89627946	89627946	C	T	UTR3	FOXN3	NA	NA	NA	NA	NA
chr14	92527592	92527592	A	-	UTR3	ATXN3	NA	NA	NA	rs201936460	NA
chr14	95655320	95655320	A	-	UTR3	CLMN	NA	NA	NA	NA	NA
chr14	1.03E+08	1.03E+08	A	C	UTR3	RCOR1	NA	NA	NA	NA	NA
chr14	1.03E+08	1.03E+08	A	C	UTR3	RCOR1	NA	NA	NA	NA	NA
chr14	1.03E+08	1.03E+08	T	C	UTR3	RCOR1	NA	NA	NA	NA	NA
chr14	1.05E+08	1.05E+08	T	C	exonic	AHNAK2	synonymous SNV	AHNAK2:NM_138420:exon7:c.A9513G:p.P3171P	NA	rs2819427	NA
chr14	1.05E+08	1.05E+08	A	G	exonic	AHNAK2	nonsynonymous SNV	AHNAK2:NM_138420:exon7:c.T6326C:p.V2109A	NA	rs201948776	NA
chr14	1.05E+08	1.05E+08	T	C	exonic	AHNAK2	nonsynonymous SNV	AHNAK2:NM_138420:exon7:c.A6145G:p.T2049A	NA	rs201406401	NA
chr14	1.06E+08	1.06E+08	C	T	ncRNA_exonic	KIAA0125	NA	NA	NA	rs11850004	NA
chr15	21071781	21071781	A	G	UTR5	POTEB	NA	NA	NA	NA	NA
chr15	21940266	21940266	-	A	ncRNA_exonic	LOC646214	NA	NA	NA	NA	NA
chr15	22742718	22742718	T	A	exonic	GOLGA6L1	nonsynonymous SNV	GOLGA6L1:NM_001001413:exon8:c.T1103A:p.L368Q	ID=COSM230296;OCCURENCE=1(NS),2(endometrium)	rs7171381	NA
chr15	23377464	23377464	T	C	ncRNA_exonic	HERC2P2	NA	NA	NA	rs2920545	NA
chr15	28518112	28518112	C	A	exonic	HERC2	nonsynonymous SNV	HERC2:NM_004667:exon8:c.G839T:p.S280I	NA	rs200859595	B
chr15	31664441	31664441	T	C	exonic	KLF13	nonsynonymous SNV	KLF13:NM_015995:exon2:c.T806C:p.L269P	NA	NA	NA

chr15	40655873	40655873	G	C	exonic	DISP2	nonsynonymous SNV	DISP2:NM_033510:exon2:c.G167C:p.C56S	NA	rs1898882	D
chr15	41871513	41871513	T	A	UTR3	TYRO3	NA	NA	NA	NA	NA
chr15	43652835	43652835	A	-	UTR3	ZSCAN29	NA	NA	NA	rs67982601	NA
chr15	48065010	48065010	A	C	UTR3	SEMA6D	NA	NA	NA	rs56345741	NA
chr15	50643858	50643865	GAGGGAGG	-	ncRNA_exonic	FLJ10038	NA	NA	NA	rs146731069	NA
chr15	52413807	52413807	-	T	UTR3	GNB5	NA	NA	NA	NA	NA
chr15	55904895	55904895	A	T	UTR3	PRTG	NA	NA	NA	NA	NA
chr15	57840202	57840202	T	-	UTR3	CGNL1	NA	NA	NA	NA	NA
chr15	68594394	68594423	TGTGTGTG	-	UTR3	ITGA11	NA	NA	NA	NA	NA
chr15	70341342	70341342	A	-	UTR3	TLE3	NA	NA	NA	NA	NA
chr15	70341854	70341854	A	-	UTR3	TLE3	NA	NA	NA	rs11324999	NA
chr15	72452675	72452677	AAA	-	UTR3	GRAMD2	NA	NA	NA	NA	NA
chr15	72958366	72958366	T	G	exonic	GOLGA6B	nonsynonymous SNV	GOLGA6B:NM_018652:exon17:c.T1851G:p.H617Q	NA	rs200655139	NA
chr15	82634574	82634574	C	T	UTR3	GOLGA6L10	NA	NA	NA	rs28482898	NA
chr15	82634594	82634594	A	G	UTR3	GOLGA6L10	NA	NA	NA	rs4039143	NA
chr15	82637315	82637315	C	T	exonic	GOLGA6L10	synonymous SNV	GOLGA6L10:NM_001164465:exon6:c.G771A:p.L257L	NA	rs200605893	NA
chr15	82637377	82637377	G	A	exonic	GOLGA6L10	nonsynonymous SNV	GOLGA6L10:NM_001164465:exon6:c.C709T:p.R237C	NA	rs200328108	NA
chr15	82637378	82637378	T	C	exonic	GOLGA6L10	synonymous SNV	GOLGA6L10:NM_001164465:exon6:c.A708G:p.L236L	NA	NA	NA
chr15	82637419	82637419	A	G	exonic	GOLGA6L10	nonsynonymous SNV	GOLGA6L10:NM_001164465:exon6:c.T667C:p.C223R	NA	NA	NA
chr15	82637420	82637420	C	T	exonic	GOLGA6L10	synonymous SNV	GOLGA6L10:NM_001164465:exon6:c.G666A:p.L222L	NA	NA	NA
chr15	83013917	83013917	C	T	intergenic	ist=27490),UBE2Q2	NA	NA	NA	NA	NA
chr15	83502439	83502439	T	-	UTR3	WHAMM	NA	NA	NA	NA	NA
chr15	85053033	85053033	C	T	ncRNA_exonic	GOLGA6L5	NA	NA	NA	NA	NA
chr15	85185301	85185301	G	A	ncRNA_exonic	SCAND2P	NA	NA	NA	rs147659526	NA
chr15	89398883	89398883	A	T	exonic	ACAN	nonsynonymous SNV	;:exon12:c.A3067T:p.T1023S,ACAN:NM_013227:exon12:c	NA	NA	NA
chr15	90349667	90349667	T	G	exonic	ANPEP	nonsynonymous SNV	ANPEP:NM_001150:exon2:c.A148C:p.T50P	NA	NA	B
chr15	91425783	91425783	T	C	UTR3	FURIN	NA	NA	NA	NA	NA
chr15	92690366	92690366	A	C	exonic	SLCO3A1	synonymous SNV	5044:exon8:c.A1665C:p.T555T,SLCO3A1:NM_013272:exo	NA	NA	NA
chr15	96881005	96881005	A	-	UTR3	NR2F2	NA	NA	NA	rs34678417	NA
chr15	98981194	98981196	GAT	-	UTR3	FAM169B	NA	NA	NA	NA	NA
chr15	98981251	98981253	GAT	-	UTR3	FAM169B	NA	NA	NA	NA	NA
chr15	98981261	98981263	GGA	-	UTR3	FAM169B	NA	NA	NA	NA	NA
chr15	98981290	98981290	T	-	UTR3	FAM169B	NA	NA	NA	NA	NA
chr15	1E+08	1E+08	CAGCAG	-	exonic	MEF2A	nonframeshift deletion	1EF2A:NM_001130927:exon10:c.1048_1053del:p.350_35	NA	NA	NA
chr16	449109	449109	A	G	exonic	NME4	nonsynonymous SNV	NME4:NM_005009:exon2:c.A211G:p.M71V	NA	NA	D
chr16	822742	822742	T	G	intergenic	st=2465),RPUSD1(d	NA	NA	NA	NA	NA
chr16	848213	848213	T	G	UTR3	GNG13	NA	NA	NA	NA	NA
chr16	1036733	1036733	C	T	UTR3	SOX8	NA	NA	NA	NA	NA
chr16	1258193	1258193	A	C	exonic	CACNA1H	nonsynonymous SNV	)7:exon16:c.A3335C:p.D1112A,CACNA1H:NM_021098:exo	NA	rs202114960	NA
chr16	1291608	1291608	A	G	exonic	TPSAB1	nonsynonymous SNV	TPSAB1:NM_003294:exon4:c.A407G:p.H136R	NA	rs1064780	B
chr16	1306802	1306802	A	G	exonic	TPSD1	nonsynonymous SNV	TPSD1:NM_012217:exon3:c.A259G:p.I87V	NA	rs2401930	B
chr16	1306817	1306817	G	A	exonic	TPSD1	nonsynonymous SNV	TPSD1:NM_012217:exon3:c.G274A:p.A92T	ID=COSM226769;OCCURENCE=1(skin)	rs1141968	B
chr16	1414996	1414996	T	-	UTR3	UNKL	NA	NA	NA	rs112378427	NA
chr16	1591962	1591962	G	A	exonic	TMEM204	synonymous SNV	)0:exon2:c.G321A:p.T107T,TMEM204:NM_001256541:ex	NA	rs2076443	NA
chr16	1878295	1878295	G	A	UTR3	FAHD1	NA	NA	NA	NA	NA
chr16	2052345	2052345	T	G	exonic	ZNF598	nonsynonymous SNV	ZNF598:NM_178167:exon7:c.A757C:p.T253P	NA	NA	NA
chr16	2121850	2121850	-	C	exonic	TSC2	frameshift insertion	SC2:NM_001077183:exon19:c.2012_2013insC:p.G671fs,T	NA	NA	NA
chr16	3077670	3077670	A	C	UTR3	THOC6	NA	NA	NA	NA	NA
chr16	3703880	3703880	C	T	UTR5	DNASE1	NA	NA	NA	rs77563984	NA
chr16	3703881	3703881	C	G	UTR5	DNASE1	NA	NA	NA	rs79356805	NA
chr16	15818992	15818992	A	-	UTR3	NDE1	NA	NA	NA	NA	NA
chr16	16367669	16367669	T	A	exonic	NOMO3	nonsynonymous SNV	NOMO3:NM_001004067:exon19:c.T2178A:p.N726K	NA	rs370986	B
chr16	19131760	19131760	T	-	UTR3	ITPRIPL2	NA	NA	NA	rs112434410	NA
chr16	19513697	19513702	TTTTTT	-	UTR3	GDE1	NA	NA	NA	NA	NA
chr16	19563849	19563851	AAA	-	UTR3	CCP110	NA	NA	NA	NA	NA
chr16	22546469	22546469	A	C	exonic	LOC100132247	nonsynonymous SNV	LOC100132247:NM_001135865:exon7:c.A2165C:p.H722F	NA	NA	NA
chr16	23073703	23073703	A	T	UTR3	USP31	NA	NA	NA	rs8063453	NA
chr16	23076644	23076644	A	-	UTR3	USP31	NA	NA	NA	NA	NA
chr16	27080038	27080038	T	-	UTR3	C16orf82	NA	NA	NA	NA	NA
chr16	29821984	29821984	T	-	UTR3	MAZ	NA	NA	NA	rs71650270	NA
chr16	29910394	29910394	A	C	UTR5	SEZ6L2	NA	NA	NA	NA	NA
chr16	55798759	55798759	T	C	ncRNA_exonic	CES1P1	NA	NA	NA	rs71374087	NA

chr16	55798778	55798778	A	G	ncRNA_exonic	CES1P1	NA	NA	NA	rs76167618	NA
chr16	55798783	55798783	A	G	ncRNA_exonic	CES1P1	NA	NA	NA	rs75712934	NA
chr16	56691861	56691861	T	C	UTR5	MT1F	NA	NA	NA	NA	NA
chr16	66544113	66544113	-	A	UTR3	TK2	NA	NA	NA	NA	NA
chr16	67905065	67905065	C	T	UTR3	NUTF2	NA	NA	NA	NA	NA
chr16	68678505	68678505	G	T	UTR5	CDH3	NA	NA	NA	NA	NA
chr16	69151479	69151479	-	T	UTR3	HAS3	NA	NA	NA	NA	NA
chr16	69339663	69339663	T	-	UTR3	SNTB2	NA	NA	NA	NA	NA
chr16	70010369	70010369	C	G	ncRNA_exonic	PDXDC2P	NA	NA	NA	rs187431035	NA
chr16	85125545	85125549	TTTTT	-	UTR3	KIAA0513	NA	NA	NA	NA	NA
chr16	85695241	85695241	G	A	exonic	GSE1	synonymous SNV	4473:exon8:c.G1818A:p.S606S,GSE1:NM_014615:exon9:c	NA	NA	NA
chr16	85695242	85695242	C	G	exonic	GSE1	nonsynonymous SNV	4473:exon8:c.C1819G:p.P607A,GSE1:NM_014615:exon9:c	NA	NA	D
chr16	85695243	85695243	C	G	exonic	GSE1	nonsynonymous SNV	4473:exon8:c.C1820G:p.P607R,GSE1:NM_014615:exon9:c	NA	NA	D
chr16	85695244	85695244	C	A	exonic	GSE1	synonymous SNV	4473:exon8:c.C1821A:p.P607P,GSE1:NM_014615:exon9:c	NA	NA	NA
chr16	88506287	88506287	-	CTCTGACCA	UTR3	ZNF469	NA	NA	NA	rs3838248	NA
chr16	89346906	89346906	T	G	exonic	ANKRD11	nonsynonymous SNV	.5S,ANKRD11:NM_013275:exon9:c.A6044C:p.Y2015S,ANK	NA	NA	D
chr17	263576	263576	T	C	exonic	C17orf97	synonymous SNV	C17orf97:NM_001013672:exon2:c.T942C:p.G314G	NA	rs111445631	NA
chr17	1657980	1657980	G	-	UTR3	SERPINF2	NA	NA	NA	rs67887323	NA
chr17	1683882	1683882	C	A	UTR3	SMYD4	NA	NA	NA	NA	NA
chr17	2938272	2938272	T	-	UTR3	RAP1GAP2	NA	NA	NA	rs71830536	NA
chr17	7190439	7190439	T	C	UTR3	SLC2A4	NA	NA	NA	NA	NA
chr17	7215429	7215429	T	-	UTR3	EIF5A	NA	NA	NA	NA	NA
chr17	8294348	8294348	T	-	UTR3	RNF222	NA	NA	NA	NA	NA
chr17	11513845	11513845	G	T	exonic	DNAH9	nonsynonymous SNV	DNAH9:NM_001372:exon3:c.G747T:p.K249N	NA	NA	B
chr17	15621245	15621245	-	T	UTR3	ZNF286A	NA	NA	NA	NA	NA
chr17	20407483	20407483	T	C	ncRNA_exonic	KRT16P3	NA	NA	NA	NA	NA
chr17	20767707	20767707	C	T	UTR3	CCDC144NL	NA	NA	NA	NA	NA
chr17	21203941	21203941	G	A	exonic	MAP2K3	nonsynonymous SNV	2756:exon4:c.G163A:p.A55T,MAP2K3:NM_145109:exon4	NA	rs2305873	B
chr17	21207844	21207844	C	T	exonic	MAP2K3	synonymous SNV	756:exon8:c.C588T:p.A196A,MAP2K3:NM_145109:exon8	NA	rs2230436	NA
chr17	21320854	21320854	A	-	UTR3	KCNJ12	NA	NA	NA	rs67230821	NA
chr17	21911267	21911268	TG	-	ncRNA_exonic	FLJ36000	NA	NA	NA	NA	NA
chr17	31618865	31618865	A	C	exonic	ASIC2	nonsynonymous SNV	ASIC2:NM_183377:exon1:c.T269G:p.V90G	NA	NA	NA
chr17	33765127	33765127	A	-	UTR3	SLFN13	NA	NA	NA	rs11337992	NA
chr17	34493108	34493108	T	G	UTR3	TBC1D3B	NA	NA	NA	NA	NA
chr17	36883387	36883387	T	-	UTR3	MLLT6	NA	NA	NA	NA	NA
chr17	37221649	37221650	TT	-	ncRNA_UTR3	PLXDC1	NA	NA	NA	rs149203975	NA
chr17	39254143	39254143	C	T	exonic	KRTAP4-8	nonsynonymous SNV	KRTAP4-8:NM_031960:exon1:c.G194A:p.C65Y	NA	NA	NA
chr17	39340786	39340786	A	G	exonic	KRTAP4-1	synonymous SNV	KRTAP4-1:NM_033060:exon2:c.T264C:p.C88C	NA	rs2320228	NA
chr17	39675023	39675023	T	C	exonic	KRT15	synonymous SNV	KRT15:NM_002275:exon1:c.A57G:p.R19R	NA	NA	NA
chr17	40465420	40465420	G	-	UTR3	STAT3	NA	NA	NA	NA	NA
chr17	41144609	41144609	G	A	UTR3	RUNDC1	NA	NA	NA	rs201072724	NA
chr17	41196822	41196823	TT	-	UTR3	BRCA1	NA	NA	NA	NA	NA
chr17	42248239	42248239	C	A	exonic	ASB16	synonymous SNV	ASB16:NM_080863:exon1:c.C82A:p.R28R	NA	rs144229400	NA
chr17	42266708	42266708	A	G	exonic	TMUB2	synonymous SNV	98G,TMUB2:NM_177441:exon2:c.A294G:p.G98G,TMUB2:	NA	NA	NA
chr17	42851738	42851738	T	G	exonic	ADAM11	synonymous SNV	ADAM11:NM_002390:exon12:c.T1068G:p.G356G	NA	NA	NA
chr17	43101699	43101708	TGTGTGTG	-	UTR3	DCAKD	NA	NA	NA	NA	NA
chr17	45214564	45214564	A	T	exonic	CDC27	nonsynonymous SNV	091:exon14:c.T1885A:p.C629S,CDC27:NM_001256:exon1	D=COSM1179254,COSM1179255;OCCURENCE=1(prostate	rs201184967	NA
chr17	45214606	45214606	G	T	exonic	CDC27	nonsynonymous SNV	91:exon14:c.C1843A:p.H615N,CDC27:NM_001256:exon1	NA	rs79260965	NA
chr17	45214636	45214636	T	C	exonic	CDC27	nonsynonymous SNV	91:exon14:c.A1813G:p.N605D,CDC27:NM_001256:exon1	NA	rs200200993	NA
chr17	47132506	47132506	A	T	UTR3	IGF2BP1	NA	NA	NA	NA	NA
chr17	47373330	47373330	G	A	UTR3	ZNF652	NA	NA	NA	NA	NA
chr17	47373333	47373333	C	A	UTR3	ZNF652	NA	NA	NA	NA	NA
chr17	48771502	48771502	G	A	UTR3	ANKRD40	NA	NA	NA	rs11079923	NA
chr17	48771507	48771507	G	A	UTR3	ANKRD40	NA	NA	NA	rs199897066	NA
chr17	54965932	54965932	A	G	UTR3	TRIM25	NA	NA	NA	rs7615	NA
chr17	55184422	55184422	A	C	exonic	AKAP1	nonsynonymous SNV	P,AKAP1:NM_001242902:exon3:c.A1597C:p.T533P,AKAP	NA	rs202031778	P
chr17	58524282	58524282	A	-	UTR3	APPBP2	NA	NA	NA	NA	NA
chr17	60021734	60021734	T	-	UTR3	MED13	NA	NA	NA	NA	NA
chr17	61574642	61574642	G	A	exonic	ACE	nonsynonymous SNV	.R664Q,ACE:NM_152830:exon14:c.G2114A:p.R705Q,ACE:	NA	rs200754517	D
chr17	62541835	62541835	A	-	UTR3	SMURF2	NA	NA	NA	NA	NA
chr17	73498845	73498845	T	G	exonic	CASKIN2	synonymous SNV	543:exon17:c.A2064C:p.P688P,CASKIN2:NM_020753:exor	NA	NA	NA
chr17	75090386	75090386	T	C	ncRNA_exonic	LINC00338	NA	NA	NA	NA	NA

chr17	75090396	75090396	G	A	ncRNA_exonic	LINC00338	NA	NA	NA	NA	NA
chr17	76083017	76083017	A	C	exonic	TNRC6C	synonymous SNV	40:exon14:c.A3636C:p.P1212P,TNRC6C:NM_018996:exon	NA	NA	NA
chr17	76102335	76102335	A	-	UTR3	TNRC6C	NA	NA	NA	rs58424290	NA
chr17	76798445	76798445	C	A	exonic	USP36	nonsynonymous SNV	USP36:NM_025090:exon17:c.G2983T:p.A995S	NA	rs145562419	B
chr17	77807715	77807715	G	T	UTR3	CBX4	NA	NA	NA	NA	NA
chr17	78326270	78326270	A	G	ncRNA_exonic	LOC100294362	NA	NA	NA	NA	NA
chr17	79517455	79517455	A	C	exonic	C17orf70	synonymous SNV	C17orf70:NM_025161:exon3:c.T1065G:p.G355G	ID=COSM309626;OCCURENCE=1(lung)	rs202147413	NA
chr17	79615572	79615572	A	G	UTR3	TSPAN10	NA	NA	NA	rs7405453	NA
chr18	3412167	3412167	C	T	UTR5	TGIF1	NA	NA	NA	rs11571505	NA
chr18	9861101	9861101	A	-	UTR3	RAB31	NA	NA	NA	NA	NA
chr18	9887252	9887252	C	A	exonic	TXNDC2	nonsynonymous SNV	98529:exon2:c.C776A:p.A259E,TXNDC2:NM_032243:exon	NA	NA	NA
chr18	31322986	31322986	G	A	exonic	ASXL3	synonymous SNV	ASXL3:NM_030632:exon12:c.G3174A:p.K1058K	NA	NA	NA
chr18	42644796	42644796	C	A	UTR3	SETBP1	NA	NA	NA	NA	NA
chr18	45363933	45363933	A	-	UTR3	SMAD2	NA	NA	NA	NA	NA
chr18	47350146	47350146	T	C	UTR3	MYO5B	NA	NA	NA	NA	NA
chr18	47350148	47350148	A	G	UTR3	MYO5B	NA	NA	NA	NA	NA
chr18	48610376	48610376	G	A	UTR3	SMAD4	NA	NA	NA	NA	NA
chr18	55215503	55215503	C	A	UTR3	FECH	NA	NA	NA	NA	NA
chr18	61265349	61265349	T	-	UTR3	SERPINB13	NA	NA	NA	rs57085048	NA
chr18	61325815	61325815	A	G	exonic	SERPINB3	nonsynonymous SNV	SERPINB3:NM_006919:exon5:c.T401C:p.V134A	NA	rs148254791	B
chr18	61325816	61325816	C	T	exonic	SERPINB3	nonsynonymous SNV	SERPINB3:NM_006919:exon5:c.G400A:p.V134I	NA	rs61754491	B
chr18	64171913	64171913	A	T	UTR3	CDH19	NA	NA	NA	NA	NA
chr18	72593013	72593013	A	C	exonic	ZNF407	nonsynonymous SNV	.89:exon5:c.A5066C:p.D1689A,ZNF407:NM_017757:exon	NA	NA	NA
chr19	199295	199295	G	A	ncRNA_exonic	FLJ45445	NA	NA	NA	NA	NA
chr19	199442	199442	G	T	ncRNA_exonic	FLJ45445	NA	NA	NA	rs57399907	NA
chr19	624882	624882	T	C	exonic	POLRMT	nonsynonymous SNV	POLRMT:NM_005035:exon5:c.A977G:p.E326G	NA	NA	P
chr19	1095352	1095352	C	G	UTR5	POLR2E	NA	NA	NA	rs17554924	NA
chr19	1095355	1095355	C	T	UTR5	POLR2E	NA	NA	NA	rs17554917	NA
chr19	1472591	1472591	T	C	UTR3	APC2	NA	NA	NA	NA	NA
chr19	1472601	1472601	A	C	UTR3	APC2	NA	NA	NA	NA	NA
chr19	1597572	1597572	G	T	UTR3	UQCR11	NA	NA	NA	NA	NA
chr19	1925930	1925930	G	C	UTR3	SCAMP4	NA	NA	NA	NA	NA
chr19	4045624	4045624	C	G	UTR3	ZBTB7A	NA	NA	NA	NA	NA
chr19	4511737	4511737	T	C	exonic	PLIN4	synonymous SNV	PLIN4:NM_001080400:exon3:c.A2193G:p.K731K	NA	rs57610751	NA
chr19	4512676	4512676	T	G	exonic	PLIN4	synonymous SNV	PLIN4:NM_001080400:exon3:c.A1254C:p.T418T	NA	rs59593546	NA
chr19	4513044	4513044	C	T	exonic	PLIN4	nonsynonymous SNV	PLIN4:NM_001080400:exon3:c.G886A:p.G296S	NA	rs56366613	NA
chr19	4523091	4523091	T	-	UTR3	PLIN5	NA	NA	NA	rs71168910	NA
chr19	5915980	5915980	T	G	UTR3	CAPS	NA	NA	NA	NA	NA
chr19	7965897	7965897	C	G	UTR3	LRRC8E	NA	NA	NA	NA	NA
chr19	9761701	9761701	C	T	UTR3	ZNF562	NA	NA	NA	NA	NA
chr19	10579172	10579172	G	C	UTR3	PDE4A	NA	NA	NA	NA	NA
chr19	10579177	10579177	A	C	UTR3	PDE4A	NA	NA	NA	NA	NA
chr19	11558341	11558346	GAGGAG	-	exonic	PRKCSH	nonframeshift deletion	11:c.937_942del:p.313_314del,PRKCSH:NM_002743:exon	NA	NA	NA
chr19	11562297	11562297	C	T	UTR3	ELAVL3	NA	NA	NA	NA	NA
chr19	11563263	11563263	G	A	UTR3	ELAVL3	NA	NA	NA	NA	NA
chr19	13317726	13317726	A	T	UTR3	CACNA1A	NA	NA	NA	NA	NA
chr19	13317759	13317767	TTTTTTTTT	-	UTR3	CACNA1A	NA	NA	NA	NA	NA
chr19	17001071	17001071	C	A	exonic	F2RL3	nonsynonymous SNV	F2RL3:NM_003950:exon2:c.C797A:p.A266D	NA	NA	D
chr19	17038890	17038890	C	A	exonic	CPAMD8	nonsynonymous SNV	CPAMD8:NM_015692:exon25:c.G3440T:p.S1147I	NA	NA	NA
chr19	17714607	17714607	T	-	UTR3	UNC13A	NA	NA	NA	NA	NA
chr19	18260796	18260796	T	-	UTR3	MAST3	NA	NA	NA	rs61501122	NA
chr19	18703077	18703077	T	C	UTR3	C19orf60	NA	NA	NA	rs3170474	NA
chr19	20803652	20803652	-	T	UTR3	ZNF626	NA	NA	NA	rs139108386	NA
chr19	20806673	20806673	C	T	UTR3	ZNF626	NA	NA	NA	NA	NA
chr19	22363817	22363817	A	G	exonic	ZNF676	synonymous SNV	ZNF676:NM_001001411:exon3:c.T702C:p.F234F	NA	NA	NA
chr19	35850711	35850711	A	G	exonic	FFAR3	nonsynonymous SNV	FFAR3:NM_005304:exon2:c.A919G:p.M307V	NA	rs12459836	B
chr19	36114999	36114999	C	T	UTR3	HAUS5	NA	NA	NA	NA	NA
chr19	36135645	36135645	T	A	exonic	ETV2	nonsynonymous SNV	ETV2:NM_014209:exon7:c.T920A:p.I307N	NA	NA	NA
chr19	36135646	36135646	C	G	exonic	ETV2	nonsynonymous SNV	ETV2:NM_014209:exon7:c.C921G:p.I307M	NA	NA	NA
chr19	36135647	36135647	G	A	exonic	ETV2	nonsynonymous SNV	ETV2:NM_014209:exon7:c.G922A:p.V308M	NA	NA	NA
chr19	36135649	36135649	G	C	exonic	ETV2	synonymous SNV	ETV2:NM_014209:exon7:c.G924C:p.V308V	NA	NA	NA
chr19	36135650	36135650	C	G	exonic	ETV2	nonsynonymous SNV	ETV2:NM_014209:exon7:c.C925G:p.R309G	NA	NA	NA

chr19	37759367	37759367	C	A	ncRNA_exonic	LOC284412	NA	NA	NA	rs201738321	NA
chr19	38032097	38032097	-	A	UTR3	ZNF793	NA	NA	NA	NA	NA
chr19	38795905	38795905	C	A	UTR3	YIF1B	NA	NA	NA	NA	NA
chr19	40021823	40021823	-	T	UTR3	EID2B	NA	NA	NA	NA	NA
chr19	41515263	41515263	A	G	exonic	CYP2B6	nonsynonymous SNV	CYP2B6:NM_000767:exon5:c.A785G:p.K262R	NA	rs2279343	B
chr19	41811707	41811707	T	C	exonic	HNRNPUL1	nonsynonymous SNV	0:exon14:c.T2389C:p.Y797H,HNRNPUL1:NM_144732:exo	NA	NA	NA
chr19	42799307	42799307	A	C	exonic	CIC	synonymous SNV	CIC:NM_015125:exon20:c.A4791C:p.P1597P	NA	NA	NA
chr19	43376101	43376101	G	T	exonic	PSG1	nonsynonymous SNV	.A176E,PSG1:NM_001184826:exon3:c.C527A:p.A176E,PS(	NA	NA	NA
chr19	44117603	44117603	T	A	exonic	SRRM5	nonsynonymous SNV	SRRM5:NM_001145641:exon1:c.T1330A:p.Y444N	ID=COSM474846;OCCURENCE=1(kidney),5(endometrium)	rs201848853	NA
chr19	45262965	45262965	C	-	UTR3	BCL3	NA	NA	NA	NA	NA
chr19	45323171	45323187	CCCAACTCA	-	UTR3	BCAM	NA	NA	NA	rs3842409	NA
chr19	46052048	46052048	-	TTATTTAT	UTR3	OPA3	NA	NA	NA	NA	NA
chr19	46273463	46273507	:AGCAGCAC	-	UTR3	DMPK	NA	NA	NA	NA	NA
chr19	46442771	46442771	G	A	UTR3	NOVA2	NA	NA	NA	NA	NA
chr19	47278314	47278319	ACACAC	-	UTR3	SLC1A5	NA	NA	NA	NA	NA
chr19	48984071	48984071	C	G	UTR3	CYTH2	NA	NA	NA	NA	NA
chr19	54378026	54378027	TT	-	UTR3	MYADM	NA	NA	NA	NA	NA
chr19	54378031	54378031	T	C	UTR3	MYADM	NA	NA	NA	NA	NA
chr19	54725798	54725798	G	T	exonic	LILRB3	nonsynonymous SNV	31450:exon4:c.C560A:p.T187N,LILRB3:NM_006864:exon4	ID=COSM1158374;OCCURENCE=1(pancreas)	rs1052968	B
chr19	54725835	54725835	G	C	exonic	LILRB3	nonsynonymous SNV	31450:exon4:c.C523G:p.R175G,LILRB3:NM_006864:exon4	NA	rs201948566	B
chr19	54742303	54742303	C	T	UTR3	LILRA6	NA	NA	NA	rs71365449	NA
chr19	54818552	54818552	G	-	UTR3	LILRA5	NA	NA	NA	NA	NA
chr19	54818578	54818578	G	A	UTR3	LILRA5	NA	NA	NA	NA	NA
chr19	55286864	55286864	A	C	exonic	KIR2DL1	synonymous SNV	KIR2DL1:NM_014218:exon4:c.A618C:p.P206P	NA	rs201422291	NA
chr19	55607307	55607307	T	G	exonic	PPP1R12C	nonsynonymous SNV	1618:exon9:c.A1155C:p.E385D,PPP1R12C:NM_017607:ex	NA	NA	P
chr20	309179	309179	T	A	UTR3	SOX12	NA	NA	NA	NA	NA
chr20	309180	309180	T	A	UTR3	SOX12	NA	NA	NA	NA	NA
chr20	309181	309181	T	G	UTR3	SOX12	NA	NA	NA	NA	NA
chr20	1895794	1895794	G	A	exonic	SIRPA	synonymous SNV	o.V43V,SIRPA:NM_001040022:exon3:c.G129A:p.V43V,SIR	NA	rs1135192	NA
chr20	16033819	16033822	TTTG	-	UTR3	MACROD2	NA	NA	NA	rs11471411	NA
chr20	17971045	17971045	A	G	UTR3	MGME1	NA	NA	NA	NA	NA
chr20	19982007	19982010	TTTT	-	UTR3	RIN2	NA	NA	NA	NA	NA
chr20	25262727	25262727	A	C	exonic	PYGB	nonsynonymous SNV	PYGB:NM_002862:exon12:c.A1462C:p.T488P	NA	NA	D
chr20	25595952	25595952	A	-	UTR3	NANP	NA	NA	NA	NA	NA
chr20	25753637	25753637	C	G	ncRNA_exonic	FAM182B	NA	NA	NA	rs75733040	NA
chr20	25753646	25753646	G	C	ncRNA_exonic	FAM182B	NA	NA	NA	rs78538493	NA
chr20	25753703	25753703	G	A	ncRNA_exonic	FAM182B	NA	NA	NA	rs76136647	NA
chr20	25753734	25753734	T	G	ncRNA_exonic	FAM182B	NA	NA	NA	rs76753216	NA
chr20	25754060	25754060	C	T	ncRNA_exonic	FAM182B	NA	NA	NA	rs74458126	NA
chr20	25754073	25754073	T	A	ncRNA_exonic	FAM182B	NA	NA	NA	rs73904672	NA
chr20	25754607	25754607	C	A	ncRNA_exonic	FAM182B	NA	NA	NA	rs115482967	NA
chr20	25754620	25754620	A	G	ncRNA_exonic	FAM182B	NA	NA	NA	NA	NA
chr20	25754622	25754622	A	G	ncRNA_exonic	FAM182B	NA	NA	NA	NA	NA
chr20	25754689	25754689	T	C	ncRNA_exonic	FAM182B	NA	NA	NA	rs79458516	NA
chr20	25754756	25754756	G	A	ncRNA_exonic	FAM182B	NA	NA	NA	rs76339741	NA
chr20	25754774	25754774	A	C	ncRNA_exonic	FAM182B	NA	NA	NA	NA	NA
chr20	25754782	25754782	G	T	ncRNA_exonic	FAM182B	NA	NA	NA	NA	NA
chr20	26063630	26063630	C	T	ncRNA_exonic	FAM182A	NA	NA	NA	NA	NA
chr20	26063651	26063651	A	G	ncRNA_exonic	FAM182A	NA	NA	NA	NA	NA
chr20	26063683	26063683	C	T	ncRNA_exonic	FAM182A	NA	NA	NA	rs145735552	NA
chr20	26064144	26064144	T	C	ncRNA_exonic	FAM182A	NA	NA	NA	NA	NA
chr20	26064389	26064389	A	T	ncRNA_exonic	FAM182A	NA	NA	NA	rs200923238	NA
chr20	26064961	26064961	G	C	ncRNA_exonic	FAM182A	NA	NA	NA	NA	NA
chr20	26084261	26084261	A	G	ncRNA_exonic	NCOR1P1	NA	NA	NA	rs61752354	NA
chr20	26084262	26084262	G	A	ncRNA_exonic	NCOR1P1	NA	NA	NA	rs79403010	NA
chr20	29611898	29611898	G	C	ncRNA_exonic	FRG1B	NA	NA	NA	NA	NA
chr20	29611950	29611950	C	T	ncRNA_exonic	FRG1B	NA	NA	NA	NA	NA
chr20	29612047	29612047	-	G	ncRNA_exonic	FRG1B	NA	NA	NA	NA	NA
chr20	29612048	29612048	-	CTTC	ncRNA_exonic	FRG1B	NA	NA	NA	NA	NA
chr20	29612050	29612050	-	CGA	ncRNA_exonic	FRG1B	NA	NA	NA	NA	NA
chr20	29612063	29612063	T	C	ncRNA_exonic	FRG1B	NA	NA	NA	NA	NA
chr20	33098206	33098206	C	T	UTR3	ITCH	NA	NA	NA	NA	NA

chr20	33098244	33098244	G	T	UTR3	ITCH	NA	NA	NA	NA	NA
chr20	42090055	42090055	A	T	UTR3	SRSF6	NA	NA	NA	NA	NA
chr20	42694558	42694558	-	CCG	exonic	TOX2	nonframeshift insertion	1_1042insCCG:p.S347delinsSP,TOX2:NM_001098797:exo	NA	rs199841880	NA
chr20	43955092	43955092	C	A	UTR3	SDC4	NA	NA	NA	NA	NA
chr20	43955096	43955096	C	A	UTR3	SDC4	NA	NA	NA	NA	NA
chr20	48251757	48251757	C	T	UTR3	B4GALT5	NA	NA	NA	NA	NA
chr20	48251758	48251758	A	C	UTR3	B4GALT5	NA	NA	NA	NA	NA
chr20	48251759	48251759	C	G	UTR3	B4GALT5	NA	NA	NA	NA	NA
chr20	49367485	49367485	A	T	UTR3	PARD6B	NA	NA	NA	NA	NA
chr20	49367490	49367490	G	T	UTR3	PARD6B	NA	NA	NA	NA	NA
chr20	49367491	49367491	A	T	UTR3	PARD6B	NA	NA	NA	NA	NA
chr20	52560855	52560856	TT	-	UTR3	BCAS1	NA	NA	NA	rs74631158	NA
chr20	55213716	55213717	TT	-	UTR3	TFAP2C	NA	NA	NA	NA	NA
chr20	60709499	60709499	C	T	UTR3	LSM14B	NA	NA	NA	NA	NA
chr20	60790986	60790986	T	G	UTR3	HRH3	NA	NA	NA	NA	NA
chr20	61145196	61145196	G	A	ncRNA_exonic	C20orf166-AS1	NA	NA	NA	rs12479469	NA
chr20	61732921	61732921	T	C	ncRNA_exonic	HAR1A	NA	NA	NA	rs73147778	NA
chr20	61888686	61888686	A	G	ncRNA_exonic	FLJ16779	NA	NA	NA	NA	NA
chr20	61888896	61888896	A	G	ncRNA_exonic	FLJ16779	NA	NA	NA	rs35166508	NA
chr21	9907222	9907222	T	C	ncRNA_exonic	TEKT4P2	NA	NA	NA	rs1052822	NA
chr21	10942995	10942995	G	A	exonic	TPTE	stopgain SNV	R160X,TPTE:NM_199259:exon11:c.C538T:p.R180X,TPTE:N	ID=COSM376393;OCCURENCE=2(lung)	rs147014138	NA
chr21	27253257	27253257	G	C	UTR3	APP	NA	NA	NA	NA	NA
chr21	28338952	28338952	T	-	UTR5	ADAMTS5	NA	NA	NA	NA	NA
chr21	28338953	28338953	G	-	UTR5	ADAMTS5	NA	NA	NA	NA	NA
chr21	34809434	34809434	T	-	UTR3	IFNGR2	NA	NA	NA	rs71657077	NA
chr21	43131861	43131861	A	G	ncRNA_exonic	LINC00479	NA	NA	NA	rs9982491	NA
chr21	44838931	44838931	A	G	exonic	SIK1	nonsynonymous SNV	SIK1:NM_173354:exon11:c.T1432C:p.S478P	NA	NA	P
chr21	45564932	45564932	T	-	UTR3	C21orf33	NA	NA	NA	rs66662664	NA
chr21	46684129	46684129	-	CTCA	UTR3	POFUT2	NA	NA	NA	rs140397390	NA
chr21	47603640	47603640	C	G	UTR5	SPATC1L	NA	NA	NA	NA	NA
chr21	47721985	47721985	-	TGG	exonic	C21orf58	nonframeshift insertion	1orf58:NM_058180:exon8:c.897_898insCCA:p.H299delins	NA	rs71318063	NA
chr22	19492888	19492888	G	A	exonic	CDC45	nonsynonymous SNV	190I,CDC45:NM_003504:exon10:c.G708A:p.M236I,CDC4!	NA	NA	B
chr22	19834263	19834263	C	A	UTR3	C22orf29	NA	NA	NA	NA	NA
chr22	21044156	21044156	C	A	ncRNA_exonic	POM121L4P	NA	NA	NA	rs192873282	NA
chr22	21649095	21649095	C	-	ncRNA_exonic	POM121L8P	NA	NA	NA	NA	NA
chr22	25044743	25044743	A	G	ncRNA_exonic	POM121L10P	NA	NA	NA	rs71242990	NA
chr22	25054716	25054716	C	G	ncRNA_exonic	POM121L10P	NA	NA	NA	rs3888507	NA
chr22	25264460	25264460	T	C	exonic	SGSM1	nonsynonymous SNV	497:exon11:c.T1112C:p.L371P,SGSM1:NM_001098498:ex	NA	NA	NA
chr22	25603066	25603066	C	G	exonic	CRYBB3	nonsynonymous SNV	CRYBB3:NM_004076:exon6:c.C523G:p.R175G	NA	NA	P
chr22	26847960	26847974	CGCGCGCG(	GCA	UTR3	HPS4	NA	NA	NA	NA	NA
chr22	29885823	29885823	A	T	exonic	NEFH	nonsynonymous SNV	NEFH:NM_021076:exon4:c.A2194T:p.T732S	NA	rs145125701	NA
chr22	29932727	29932727	C	A	exonic	THOC5	nonsynonymous SNV	78:exon7:c.G600T:p.R200S,THOC5:NM_001002877:exon8	NA	NA	P
chr22	30221159	30221159	G	A	exonic	ASCC2	nonsynonymous SNV	242906:exon3:c.C169T:p.P57S,ASCC2:NM_032204:exon4:	NA	NA	B
chr22	36662951	36662951	A	-	UTR3	APOL1	NA	NA	NA	rs11321251	NA
chr22	38687878	38687878	C	T	UTR3	CSNK1E	NA	NA	NA	NA	NA
chr22	39414807	39414807	C	A	UTR3	APOBEC3C	NA	NA	NA	NA	NA
chr22	40720295	40720295	T	-	UTR3	TNRC6B	NA	NA	NA	rs71722737	NA
chr22	42979736	42979736	C	A	UTR3	POLDIP3	NA	NA	NA	NA	NA
chr22	46436143	46436143	-	T	ncRNA_exonic	LOC100271722	NA	NA	NA	NA	NA
chr22	46712068	46712068	C	A	exonic	GTSE1	synonymous SNV	GTSE1:NM_016426:exon7:c.C1191A:p.A397A	NA	NA	NA
chr22	50655686	50655686	C	G	exonic	SELO	unknown	UNKNOWN	NA	NA	B
chrX	8434161	8434161	G	A	exonic	VCX3B	nonsynonymous SNV	VCX3B:NM_001001888:exon3:c.G478A:p.V160M	NA	rs6640132	NA
chrX	10204764	10204764	T	-	UTR3	CLCN4	NA	NA	NA	NA	NA
chrX	10416148	10416148	T	-	UTR3	MID1	NA	NA	NA	NA	NA
chrX	15511350	15511350	T	G	UTR5	PIR	NA	NA	NA	NA	NA
chrX	19552343	19552343	G	T	UTR3	SH3KBP1	NA	NA	NA	NA	NA
chrX	30748293	30748293	T	C	UTR3	GK	NA	NA	NA	rs5972221	NA
chrX	31196550	31196550	T	-	UTR3	DMD	NA	NA	NA	rs11333372	NA
chrX	37027663	37027663	A	C	exonic	FAM47C	nonsynonymous SNV	FAM47C:NM_001013736:exon1:c.A1180C:p.T394P	NA	NA	NA
chrX	37028267	37028267	C	G	exonic	FAM47C	nonsynonymous SNV	FAM47C:NM_001013736:exon1:c.C1784G:p.P595R	NA	NA	NA
chrX	37028402	37028402	A	C	exonic	FAM47C	nonsynonymous SNV	FAM47C:NM_001013736:exon1:c.A1919C:p.Y640S	NA	NA	NA
chrX	37028629	37028629	G	C	exonic	FAM47C	nonsynonymous SNV	FAM47C:NM_001013736:exon1:c.G2146C:p.A716P	NA	rs61999275	NA

chrX	38664818	38664818	T	-	UTR3	MID1IP1	NA	NA	NA	NA	NA
chrX	46919651	46919651	T	A	UTR3	PHF16	NA	NA	NA	NA	NA
chrX	54224160	54224160	-	A	UTR3	WNK3	NA	NA	NA	NA	NA
chrX	55478566	55478566	G	T	UTR5	MAGEH1	NA	NA	NA	NA	NA
chrX	70520238	70520238	T	-	ncRNA_UTR3	NONO	NA	NA	NA	rs34459863	NA
chrX	72297562	72297565	ACAC	-	UTR3	PABPC1L2A	NA	NA	NA	NA	NA
chrX	72298326	72298326	T	C	UTR3	PABPC1L2A	NA	NA	NA	rs182890337	NA
chrX	73071940	73071940	T	A	ncRNA_exonic	XIST	NA	NA	NA	NA	NA
chrX	77268407	77268407	C	A	exonic	ATP7A	nonsynonymous SNV	ATP7A:NM_000052:exon10:c.C2204A:p.A735D	NA	NA	D
chrX	84343124	84343127	ATAA	-	UTR3	APOOL	NA	NA	NA	NA	NA
chrX	1E+08	1E+08	GC	-	UTR3	TMEM35	NA	NA	NA	rs201592497	NA
chrX	1.02E+08	1.02E+08	TT	-	UTR3	BHLHB9	NA	NA	NA	rs201112932	NA
chrX	1.1E+08	1.1E+08	C	T	UTR3	CHRDL1	NA	NA	NA	rs12007691	NA
chrX	1.31E+08	1.31E+08	GAGAGAGA	-	UTR3	RAP2C	NA	NA	NA	NA	NA
chrX	1.31E+08	1.31E+08	-	A	UTR3	RAP2C	NA	NA	NA	NA	NA
chrX	1.41E+08	1.41E+08	C	G	exonic	MAGEC1	synonymous SNV	MAGEC1:NM_005462:exon4:c.C723G:p.S241S	ID=COSM150831;OCCURENCE=1(stomach)	rs176041	NA
chrX	1.45E+08	1.45E+08	TTTT	-	UTR3	SLITRK2	NA	NA	NA	NA	NA
chrX	1.5E+08	1.5E+08	C	A	exonic	MAMLD1	nonsynonymous SNV	.25N,MAMLD1:NM_001177465:exon3:c.C374A:p.T125N,I	NA	NA	P
chrX	1.53E+08	1.53E+08	T	C	UTR3	BGN	NA	NA	NA	rs1126598	NA
chrX	1.53E+08	1.53E+08	T	G	UTR3	HCFC1	NA	NA	ID=COSN414031;OCCURENCE=1(urinary_tract)	NA	NA
chrX	1.53E+08	1.53E+08	T	G	exonic	OPN1LW	nonsynonymous SNV	OPN1LW:NM_020061:exon3:c.T538G:p.S180A	NA	rs949431	B
chr1	69511	69511	A	G	exonic	OR4F5	nonsynonymous SNV	OR4F5:NM_001005484:exon1:c.A421G:p.T141A	NA	rs2691305	B
chr1	1310668	1310668	A	C	UTR5	AURKAIP1	NA	NA	NA	rs2242398	NA
chr1	3319390	3319390	C	T	exonic	PRDM16	nonsynonymous SNV	2114:exon6:c.C712T:p.L238F,PRDM16:NM_199454:exon6	NA	NA	NA
chr1	3696890	3696890	A	C	UTR3	LRRC47	NA	NA	NA	rs8379	NA
chr1	7907805	7907805	-	T	UTR3	UTS2	NA	NA	NA	rs112820691	NA
chr1	11846092	11846092	-	T	UTR3	MTHFR	NA	NA	NA	rs112870018	NA
chr1	12888545	12888545	C	T	UTR5	PRAMEF11	NA	NA	NA	rs2982101	NA
chr1	12919642	12919642	G	A	exonic	PRAMEF2	nonsynonymous SNV	PRAMEF2:NM_023014:exon3:c.G382A:p.A128T	NA	rs142476002	B
chr1	12919833	12919833	A	G	exonic	PRAMEF2	synonymous SNV	PRAMEF2:NM_023014:exon3:c.A573G:p.P191P	ID=COSM246757;OCCURENCE=1(prostate)	NA	NA
chr1	12919840	12919840	T	C	exonic	PRAMEF2	nonsynonymous SNV	PRAMEF2:NM_023014:exon3:c.T580C:p.Y194H	ID=COSM246759;OCCURENCE=1(prostate)	NA	B
chr1	13328199	13328199	A	C	UTR3	PRAMEF3	NA	NA	NA	rs56684054	NA
chr1	13328239	13328239	C	A	UTR3	PRAMEF3	NA	NA	NA	rs28394386	NA
chr1	15899478	15899478	T	C	UTR3	AGMAT	NA	NA	NA	rs11587541	NA
chr1	16111413	16111413	C	-	UTR3	FBLIM1	NA	NA	NA	rs149301626	NA
chr1	16375063	16375063	C	G	exonic	CLCNKB	nonsynonymous SNV	CLCNKB:NM_000085:exon7:c.C641G:p.A214G	NA	rs1889789	B
chr1	16375064	16375064	A	C	exonic	CLCNKB	synonymous SNV	CLCNKB:NM_000085:exon7:c.A642C:p.A214A	NA	rs1889790	NA
chr1	16890441	16890441	C	T	exonic	NBPF1	unknown	UNKNOWN	NA	rs3863779	NA
chr1	16902943	16902943	T	C	exonic	NBPF1	unknown	UNKNOWN	NA	rs3896429	NA
chr1	16915434	16915434	T	C	exonic	NBPF1	unknown	UNKNOWN	NA	rs202129231	NA
chr1	16915513	16915513	C	G	exonic	NBPF1	unknown	UNKNOWN	NA	rs61773582	NA
chr1	17023376	17023376	C	T	ncRNA_exonic	ESPNP	NA	NA	NA	rs613579	NA
chr1	17023403	17023403	G	A	ncRNA_exonic	ESPNP	NA	NA	NA	rs10907267	NA
chr1	17046549	17046549	G	A	ncRNA_exonic	ESPNP	NA	NA	NA	rs1755332	NA
chr1	17081606	17081606	A	C	UTR3	MST1L	NA	NA	NA	rs28661960	NA
chr1	17082701	17082701	A	T	UTR3	MST1L	NA	NA	NA	rs1831696	NA
chr1	20671961	20671961	G	A	exonic	VWA5B1	nonsynonymous SNV	VWA5B1:NM_001039500:exon17:c.G2639A:p.R880H	NA	rs11582960	NA
chr1	21808223	21808223	T	C	exonic	NBPF3	synonymous SNV	.453L,NBPF3:NM_001256416:exon13:c.T1531C:p.L511L,N	NA	rs145079058	NA
chr1	22357549	22357549	G	T	ncRNA_exonic	LINC00339	NA	NA	NA	rs2473291	NA
chr1	22418256	22418256	G	T	UTR3	CDC42	NA	NA	NA	NA	NA
chr1	23695593	23695593	-	G	ncRNA_exonic	C1orf213	NA	NA	NA	NA	NA
chr1	23886300	23886300	C	-	upstream	ID3	NA	NA	NA	rs139377135	NA
chr1	24999676	24999676	-	T	UTR3	SRRM1	NA	NA	NA	rs113632210	NA
chr1	25747230	25747230	G	C	exonic	RHCE	nonsynonymous SNV	38616:exon1:c.C48G:p.C16W,RHCE:NM_138617:exon1:c.(	NA	rs201634828	B
chr1	26786627	26786627	G	A	exonic	DHDDS	nonsynonymous SNV	43565:exon7:c.G640A:p.V214M,DHDDS:NM_024887:exo	ID=COSM146454;OCCURENCE=1(stomach)	rs3816539	B
chr1	28087457	28087457	G	T	UTR3	FAM76A	NA	NA	NA	rs74422309	NA
chr1	32638774	32638774	-	AAAAA	UTR3	KPNA6	NA	NA	NA	NA	NA
chr1	33148362	33148362	-	CT	UTR3	RBBP4	NA	NA	NA	rs5773380	NA
chr1	33475767	33475767	G	A	UTR3	AK2	NA	NA	NA	rs74066436	NA
chr1	33475791	33475791	A	G	UTR3	AK2	NA	NA	NA	rs6884	NA
chr1	38327384	38327384	A	G	UTR3	INPP5B	NA	NA	NA	rs12729989	NA
chr1	38423061	38423061	C	T	UTR3	SF3A3	NA	NA	NA	rs28667771	NA

chr1	39796845	39796855	TTTTTTTTT	-	intronic	MACF1	NA	NA	NA	rs201626891	NA
chr1	39835817	39835817	A	G	exonic	MACF1	nonsynonymous SNV	MACF1:NM_012090:exon47:c.A6868G:p.M2290V	NA	rs2296172	B
chr1	44084739	44084739	C	T	exonic	PTPRF	synonymous SNV	:exon26:c.C4485T:p.R1495R,PTPRF:NM_002840:exon27:	NA	rs1143701	NA
chr1	47099235	47099235	A	C	intergenic	st=16672),ATPAF1(	NA	NA	NA	rs4660950	NA
chr1	51820325	51820325	C	T	UTR3	EPS15	NA	NA	NA	rs11554033	NA
chr1	52817556	52817556	A	-	UTR3	CC2D1B	NA	NA	NA	rs60552348	NA
chr1	54681920	54681920	G	C	exonic	MRPL37	nonsynonymous SNV	MRPL37:NM_016491:exon6:c.G1097C:p.C366S	NA	rs13571	B
chr1	61921185	61921185	-	T	UTR3	NFIA	NA	NA	NA	rs111405657	NA
chr1	62702803	62702803	-	T	UTR3	KANK4	NA	NA	NA	NA	NA
chr1	62960101	62960101	A	G	exonic	DOCK7	synonymous SNV	01:exon39:c.T4942C:p.L1648L,DOCK7:NM_033407:exon3	NA	rs10889335	NA
chr1	64036764	64036764	A	G	exonic	EFCAB7	nonsynonymous SNV	EFCAB7:NM_032437:exon13:c.A1780G:p.I594V	NA	rs199608136	B
chr1	66831370	66831370	G	A	exonic	PDE4B	synonymous SNV	40:exon11:c.G1260A:p.E420E,PDE4B:NM_001037341:exo	NA	rs783036	NA
chr1	78343510	78343510	C	T	UTR3	FAM73A	NA	NA	NA	rs55760347	NA
chr1	85462665	85462665	T	G	UTR5	MCOLN2	NA	NA	NA	rs11161505	NA
chr1	89225976	89225976	T	C	exonic	PKN2	synonymous SNV	PKN2:NM_006256:exon3:c.T421C:p.L141L	NA	rs430600	NA
chr1	92149277	92149277	C	T	UTR3	TGFBR3	NA	NA	NA	rs1131243	NA
chr1	93620393	93620393	G	A	exonic	TMED5	nonsynonymous SNV	TMED5:NM_016040:exon4:c.C524T:p.T175I	NA	rs1060622	D
chr1	94982785	94982785	A	T	UTR3	ABCD3	NA	NA	NA	rs77888949	NA
chr1	94984053	94984053	A	-	UTR3	ABCD3	NA	NA	NA	rs144257807	NA
chr1	95363001	95363001	T	C	UTR3	CNN3	NA	NA	NA	rs3789699	NA
chr1	97770920	97770920	C	T	exonic	DPYD	nonsynonymous SNV	DPYD:NM_000110:exon18:c.G2194A:p.V732I	NA	rs1801160	D
chr1	1.01E+08	1.01E+08	TTTTTTTTT	-	UTR3	RTCA	NA	NA	NA	NA	NA
chr1	1.03E+08	1.03E+08	G	A	exonic	COL11A1	nonsynonymous SNV	09:exon51:c.C3851T:p.P1284L,COL11A1:NM_001854:exo	NA	rs3753841	NA
chr1	1.11E+08	1.11E+08	G	A	UTR3	SLC16A4	NA	NA	NA	rs185712869	NA
chr1	1.12E+08	1.12E+08	T	C	exonic	OVGP1	nonsynonymous SNV	OVGP1:NM_002557:exon11:c.A1562G:p.E521G	NA	rs75512011	B
chr1	1.12E+08	1.12E+08	C	T	exonic	OVGP1	synonymous SNV	OVGP1:NM_002557:exon11:c.G1560A:p.G520G	NA	NA	NA
chr1	1.12E+08	1.12E+08	G	C	exonic	OVGP1	nonsynonymous SNV	OVGP1:NM_002557:exon11:c.C1553G:p.T518S	NA	rs45455292	B
chr1	1.12E+08	1.12E+08	A	G	exonic	OVGP1	nonsynonymous SNV	OVGP1:NM_002557:exon11:c.T1540C:p.Y514H	NA	rs1126656	B
chr1	1.12E+08	1.12E+08	A	G	exonic	OVGP1	nonsynonymous SNV	OVGP1:NM_002557:exon11:c.T1531C:p.S511P	NA	rs56294468	B
chr1	1.13E+08	1.13E+08	T	G	exonic	ST7L	synonymous SNV	3727:exon7:c.A672C:p.L224L,ST7L:NM_138728:exon7:c.A	NA	NA	NA
chr1	1.15E+08	1.15E+08	G	A	UTR5	CSDE1	NA	NA	NA	rs41274132	NA
chr1	1.45E+08	1.45E+08	A	G	UTR5	NBPF8,NBPF9	NA	NA	NA	rs201543878	NA
chr1	1.45E+08	1.45E+08	T	C	UTR5	NBPF8,NBPF9	NA	NA	NA	rs199745076	NA
chr1	1.45E+08	1.45E+08	G	T	exonic	SEC22B	unknown	UNKNOWN	NA	rs2596331	NA
chr1	1.45E+08	1.45E+08	T	C	UTR3	SEC22B	NA	NA	NA	rs7550440	NA
chr1	1.48E+08	1.48E+08	T	C	exonic	NBPF14	synonymous SNV	NBPF14:NM_015383:exon22:c.A2766G:p.X922X	NA	rs79370452	NA
chr1	1.49E+08	1.49E+08	T	C	ncRNA_exonic	LOC388692	NA	NA	NA	rs149624901	NA
chr1	1.52E+08	1.52E+08	T	A	exonic	HRNR	nonsynonymous SNV	HRNR:NM_001009931:exon3:c.A7406T:p.Q2469L	NA	rs75280488	NA
chr1	1.52E+08	1.52E+08	G	A	exonic	HRNR	nonsynonymous SNV	HRNR:NM_001009931:exon3:c.C5642T:p.S1881F	NA	rs61814941	NA
chr1	1.53E+08	1.53E+08	G	T	UTR3	LCE1E	NA	NA	NA	rs114470093	NA
chr1	1.61E+08	1.61E+08	C	G	exonic	CD48	nonsynonymous SNV	6030:exon2:c.G304C:p.E102Q,CD48:NM_001778:exon2:	ID=COSM146667;OCCURENCE=1(stomach)	rs2295615	D
chr1	1.61E+08	1.61E+08	-	A	UTR3	USF1	NA	NA	NA	NA	NA
chr1	1.62E+08	1.62E+08	C	G	UTR5	SH2D1B	NA	NA	NA	rs66518016	NA
chr1	1.63E+08	1.63E+08	T	-	UTR3	RGS5	NA	NA	NA	NA	NA
chr1	1.67E+08	1.67E+08	-	A	UTR3	POGK	NA	NA	NA	NA	NA
chr1	1.68E+08	1.68E+08	A	G	exonic	ADCY10	synonymous SNV	749:exon16:c.T1938C:p.G646G,ADCY10:NM_018417:exon	NA	rs203795	NA
chr1	1.7E+08	1.7E+08	C	T	exonic	SELP	synonymous SNV	SELP:NM_003005:exon14:c.G2346A:p.T782T	NA	rs6128	NA
chr1	1.7E+08	1.7E+08	C	T	exonic	KIFAP3	synonymous SNV	17:exon14:c.G1398A:p.G466G,KIFAP3:NM_014970:exon1	NA	rs33943686	NA
chr1	1.7E+08	1.7E+08	A	G	ncRNA_exonic	LOC284688	NA	NA	NA	rs3795614	NA
chr1	1.7E+08	1.7E+08	T	C	ncRNA_exonic	LOC284688	NA	NA	NA	rs3795615	NA
chr1	1.71E+08	1.71E+08	G	T	ncRNA_exonic	FMO6P	NA	NA	NA	rs11812061	NA
chr1	1.77E+08	1.77E+08	C	T	exonic	PAPPA2	synonymous SNV	PAPPA2:NM_020318:exon22:c.C5262T:p.D1754D	NA	rs12118034	NA
chr1	1.78E+08	1.78E+08	G	A	exonic	SEC16B	synonymous SNV	SEC16B:NM_033127:exon8:c.C954T:p.S318S	NA	rs35891059	NA
chr1	1.8E+08	1.8E+08	A	G	exonic	TDRD5	nonsynonymous SNV	9089:exon7:c.A1072G:p.K358E,TDRD5:NM_001199091:ex	NA	rs6704505	B
chr1	1.8E+08	1.8E+08	A	C	exonic	TOR1AIP1	synonymous SNV	1267578:exon1:c.A64C:p.R22R,TOR1AIP1:NM_015602:ex	NA	NA	NA
chr1	1.82E+08	1.82E+08	G	A	exonic	CACNA1E	nonsynonymous SNV	6T,CACNA1E:NM_000721:exon44:c.G5863A:p.A1955T,CA	NA	rs704326	NA
chr1	1.97E+08	1.97E+08	T	G	UTR3	CFHR5	NA	NA	NA	rs10922153	NA
chr1	2.01E+08	2.01E+08	TTTATTTATT	-	UTR3	KIF14	NA	NA	NA	NA	NA
chr1	2.01E+08	2.01E+08	A	G	exonic	IGFN1	nonsynonymous SNV	IGFN1:NM_001164586:exon12:c.A5416G:p.S1806G	NA	NA	NA
chr1	2.02E+08	2.02E+08	AA	-	UTR3	ELF3	NA	NA	NA	rs77731786	NA
chr1	2.02E+08	2.02E+08	T	-	UTR3	GPR37L1	NA	NA	NA	rs35555096	NA
chr1	2.04E+08	2.04E+08	T	C	exonic	ATP2B4	synonymous SNV	01396:exon3:c.T318C:p.L106L,ATP2B4:NM_001684:exon	NA	rs2228445	NA

chr1	2.07E+08	2.07E+08	T	G	UTR3	C1orf116	NA	NA	NA	rs3020	NA
chr1	2.07E+08	2.07E+08	C	T	UTR3	C1orf116	NA	NA	NA	rs10877	NA
chr1	2.08E+08	2.08E+08	-	T	UTR3	PLXNA2	NA	NA	NA	NA	NA
chr1	2.12E+08	2.12E+08	T	G	UTR3	DTL	NA	NA	NA	rs1387815	NA
chr1	2.13E+08	2.13E+08	A	G	UTR3	NSL1	NA	NA	NA	rs11800642	NA
chr1	2.21E+08	2.21E+08	A	G	UTR5	HLX	NA	NA	NA	rs2738752	NA
chr1	2.21E+08	2.21E+08	T	-	UTR5	HLX	NA	NA	NA	rs11300127	NA
chr1	2.23E+08	2.23E+08	G	C	exonic	TAF1A	synonymous SNV	G,TAF1A:NM_001201536:exon10:c.C1143G:p.G381G,TAF	NA	rs1134898	NA
chr1	2.25E+08	2.25E+08	A	G	exonic	DNAH14	nonsynonymous SNV	DNAH14:NM_001373:exon37:c.A5771G:p.K1924R	NA	rs191528375	NA
chr1	2.27E+08	2.27E+08	A	G	UTR3	C1orf95	NA	NA	NA	rs114523191	NA
chr1	2.28E+08	2.28E+08	AAAA	-	UTR5	CDC42BPA	NA	NA	NA	rs199516755	NA
chr1	2.28E+08	2.28E+08	G	A	exonic	OBSCN	nonsynonymous SNV	0M,OBSCN:NM_052843:exon30:c.G8158A:p.V2720M,OB	NA	rs1188697	NA
chr1	2.3E+08	2.3E+08	G	A	UTR3	TAF5L	NA	NA	NA	rs3820359	NA
chr1	2.3E+08	2.3E+08	C	T	intronic	PGBD5	NA	NA	NA	rs2009265	NA
chr1	2.31E+08	2.31E+08	C	A	UTR3	FAM89A	NA	NA	NA	NA	NA
chr1	2.33E+08	2.33E+08	C	T	exonic	SIPA1L2	synonymous SNV	SIPA1L2:NM_020808:exon1:c.G219A:p.K73K	NA	rs4649383	NA
chr1	2.35E+08	2.35E+08	T	C	exonic	TARBP1	nonsynonymous SNV	TARBP1:NM_005646:exon12:c.A2032G:p.S678G	NA	rs4920246	B
chr1	2.35E+08	2.35E+08	T	C	exonic	ARID4B	synonymous SNV	.E299E,ARID4B:NM_016374:exon11:c.A897G:p.E299E,AR	NA	rs12731746	NA
chr1	2.36E+08	2.36E+08	G	C	UTR3	B3GALNT2	NA	NA	NA	rs6429095	NA
chr1	2.36E+08	2.36E+08	G	A	UTR3	B3GALNT2	NA	NA	NA	rs6429096	NA
chr1	2.38E+08	2.38E+08	C	G	UTR3	RYR2	NA	NA	NA	rs12406863	NA
chr1	2.49E+08	2.49E+08	G	A	exonic	OR2T34	nonsynonymous SNV	OR2T34:NM_001001821:exon1:c.C766T:p.L256F	NA	rs200427293	B
chr1	2.49E+08	2.49E+08	C	G	exonic	OR2T34	synonymous SNV	OR2T34:NM_001001821:exon1:c.G765C:p.L255L	NA	rs201475070	NA
chr1	2.49E+08	2.49E+08	C	-	UTR5	SH3BP5L	NA	NA	NA	rs28362662	NA
chr2	224919	224919	A	G	exonic	SH3YL1	synonymous SNV	SH3YL1:NM_015677:exon9:c.T783C:p.S261S	NA	rs2290911	NA
chr2	3718692	3718692	C	T	UTR5	ALLC	NA	NA	NA	rs13409104	NA
chr2	5839842	5839846	TTTTT	-	UTR3	SOX11	NA	NA	NA	rs200138663	NA
chr2	18768850	18768850	A	G	exonic	T5C1B,NT5C1B-RDH	synonymous SNV	NT5C1B:NM_001199088:exon2:c.T39C:p.P13P,NT5C1B-RI	NA	rs16985306	NA
chr2	20131079	20131079	T	C	exonic	WDR35	nonsynonymous SNV	exon24:c.A2915G:p.E972G,WDR35:NM_001006657:exon	NA	rs1191778	B
chr2	29023100	29023100	T	C	UTR3	PPP1CB	NA	NA	NA	NA	NA
chr2	30865267	30865267	T	-	UTR3	LCLAT1	NA	NA	NA	NA	NA
chr2	30865753	30865753	-	TT	UTR3	LCLAT1	NA	NA	NA	NA	NA
chr2	31396115	31396115	G	A	UTR3	CAPN14	NA	NA	NA	rs2044244	NA
chr2	31610701	31610701	C	T	exonic	XDH	synonymous SNV	XDH:NM_000379:exon8:c.G627A:p.E209E	NA	rs45575032	NA
chr2	33412077	33412077	G	A	exonic	LTBP1	synonymous SNV	26A,LTBP1:NM_001166265:exon2:c.G378A:p.A126A,LTBP	NA	rs1065324	NA
chr2	33447202	33447202	C	T	exonic	LTBP1	synonymous SNV	94N,LTBP1:NM_001166265:exon5:c.C882T:p.N294N,LTBP	NA	rs2290448	NA
chr2	33951299	33951299	-	TT	ncRNA_exonic	MYADML	NA	NA	NA	rs71409649	NA
chr2	37230734	37230734	C	T	exonic	HEATR5B	synonymous SNV	HEATR5B:NM_019024:exon31:c.G5001A:p.Q1667Q	NA	rs17497654	NA
chr2	37323460	37323460	G	C	UTR3	GPATCH11	NA	NA	NA	rs12474977	NA
chr2	38522260	38522260	A	T	UTR3	ATL2	NA	NA	NA	rs6987	NA
chr2	44546016	44546016	C	T	UTR3	PREPL	NA	NA	NA	rs8410	NA
chr2	46746963	46746963	A	G	UTR5	ATP6V1E2	NA	NA	NA	rs4952830	NA
chr2	58388696	58388696	A	G	exonic	FANCL	synonymous SNV	4636:exon12:c.T996C:p.S332S,FANCL:NM_018062:exon1	NA	rs848291	NA
chr2	63283478	63283478	C	A	UTR3	OTX1	NA	NA	NA	NA	NA
chr2	64119863	64119863	-	T	UTR3	VPS54	NA	NA	NA	NA	NA
chr2	71036190	71036190	A	-	UTR3	CLEC4F	NA	NA	NA	rs149426278	NA
chr2	71429725	71429725	T	C	UTR5	PAIP2B	NA	NA	NA	rs56871884	NA
chr2	72707874	72707874	A	G	exonic	EXOC6B	synonymous SNV	EXOC6B:NM_015189:exon17:c.T1671C:p.V557V	NA	rs653220	NA
chr2	85546302	85546302	T	-	UTR3	TGOLN2	NA	NA	NA	NA	NA
chr2	85549650	85549650	G	C	UTR3	TGOLN2	NA	NA	NA	NA	NA
chr2	85776490	85776490	-	TTTA	UTR3	GGCX	NA	NA	NA	rs5832649	NA
chr2	86016656	86016656	G	A	UTR3	ATOH8	NA	NA	NA	rs13035450	NA
chr2	91970053	91970053	A	G	ncRNA_exonic	GGT8P	NA	NA	NA	rs200455966	NA
chr2	91970089	91970089	G	C	ncRNA_exonic	GGT8P	NA	NA	NA	rs147452528	NA
chr2	91970112	91970112	C	T	ncRNA_exonic	GGT8P	NA	NA	NA	rs139857385	NA
chr2	96676301	96676301	C	A	ncRNA_exonic	FAHD2CP	NA	NA	NA	rs2443817	NA
chr2	97915895	97915895	T	C	exonic	ANKRD36	synonymous SNV	ANKRD36:NM_001164315:exon75:c.T5817C:p.F1939F	NA	rs201565865	NA
chr2	97915896	97915896	G	A	exonic	ANKRD36	nonsynonymous SNV	ANKRD36:NM_001164315:exon75:c.G5818A:p.E1940K	NA	rs199628660	NA
chr2	98127653	98127653	T	G	exonic	ANKRD36B	nonsynonymous SNV	ANKRD36B:NM_025190:exon39:c.A3668C:p.N1223T	NA	rs62157161	NA
chr2	98127659	98127659	T	C	exonic	ANKRD36B	nonsynonymous SNV	ANKRD36B:NM_025190:exon39:c.A3662G:p.E1221G	NA	NA	NA
chr2	99237792	99237792	C	T	UTR3	MGAT4A	NA	NA	NA	rs11892744	NA
chr2	1.07E+08	1.07E+08	AACAAC	-	UTR3	ST6GAL2	NA	NA	NA	rs200860434	NA

chr2	1.09E+08	1.09E+08	T	-	UTR3	SULT1C2	NA	NA	NA	rs35976654	NA
chr2	1.09E+08	1.09E+08	C	T	UTR3	GCC2	NA	NA	NA	rs1138412	NA
chr2	1.09E+08	1.09E+08	-	TT	UTR3	GCC2	NA	NA	NA	rs201168024	NA
chr2	1.14E+08	1.14E+08	G	C	ncRNA_exonic	DDX11L2	NA	NA	NA	rs7340345	NA
chr2	1.2E+08	1.2E+08	T	G	UTR5	EN1	NA	NA	NA	rs4989649	NA
chr2	1.21E+08	1.21E+08	G	A	exonic	EPB41L5	nonsynonymous SNV	037:exon17:c.G1384A:p.A462T,EPB41L5:NM_020909:exon	NA	rs1034489	B
chr2	1.31E+08	1.31E+08	C	A	exonic	POTEF	nonsynonymous SNV	POTEF:NM_001099771:exon17:c.G2601T:p.E867D	NA	rs2599794	NA
chr2	1.32E+08	1.32E+08	A	G	UTR5	ARHGEF4	NA	NA	NA	rs3739127	NA
chr2	1.33E+08	1.33E+08	C	T	ncRNA_exonic	ANKRD30BL	NA	NA	NA	rs112570337	NA
chr2	1.33E+08	1.33E+08	G	A	ncRNA_exonic	ANKRD30BL	NA	NA	NA	rs112577594	NA
chr2	1.33E+08	1.33E+08	A	C	ncRNA_exonic	ANKRD30BL	NA	NA	NA	rs148060042	NA
chr2	1.57E+08	1.57E+08	G	C	UTR3	GPD2	NA	NA	NA	rs1991776	NA
chr2	1.9E+08	1.9E+08	A	C	exonic	DIRC1	nonsynonymous SNV	DIRC1:NM_052952:exon2:c.T151G:p.S51A	NA	rs72902678	NA
chr2	1.92E+08	1.92E+08	G	A	exonic	MYO1B	synonymous SNV	490E,MYO1B:NM_001161819:exon16:c.G1470A:p.E490E,	NA	rs13018796	NA
chr2	1.98E+08	1.98E+08	A	T	UTR3	PGAP1	NA	NA	NA	NA	NA
chr2	1.99E+08	1.99E+08	G	A	exonic	RFTN2	synonymous SNV	RFTN2:NM_144629:exon3:c.C369T:p.L123L	NA	rs10497806	NA
chr2	2.01E+08	2.01E+08	-	T	UTR3	SPATS2L	NA	NA	NA	NA	NA
chr2	2.01E+08	2.01E+08	A	-	UTR3	SPATS2L	NA	NA	NA	rs11313633	NA
chr2	2.02E+08	2.02E+08	A	-	UTR3	TRAK2	NA	NA	NA	rs138690285	NA
chr2	2.07E+08	2.07E+08	T	C	exonic	NRP2	synonymous SNV	264:exon6:c.T870C:p.N290N,NRP2:NM_201266:exon6:c.T	NA	rs2228639	NA
chr2	2.11E+08	2.11E+08	T	G	exonic	ACADL	nonsynonymous SNV	ACADL:NM_001608:exon9:c.A997C:p.K333Q	NA	rs2286963	D
chr2	2.16E+08	2.16E+08	G	A	exonic	BARD1	nonsynonymous SNV	BARD1:NM_000465:exon1:c.C70T:p.P24S	NA	rs1048108	B
chr2	2.2E+08	2.2E+08	A	G	UTR3	PRKAG3	NA	NA	NA	rs6436094	NA
chr2	2.24E+08	2.24E+08	T	C	exonic	KCNE4	synonymous SNV	KCNE4:NM_080671:exon2:c.T417C:p.P139P	NA	rs10201907	NA
chr2	2.26E+08	2.26E+08	TGG	-	UTR5	NYAP2	NA	NA	NA	rs10578238	NA
chr2	2.28E+08	2.28E+08	C	T	exonic	COL4A4	synonymous SNV	COL4A4:NM_000092:exon42:c.G4080A:p.P1360P	NA	rs2228556	NA
chr2	2.28E+08	2.28E+08	C	T	exonic	COL4A4	nonsynonymous SNV	COL4A4:NM_000092:exon42:c.G3979A:p.V1327M	NA	rs2229813	NA
chr2	2.28E+08	2.28E+08	G	A	exonic	COL4A4	nonsynonymous SNV	COL4A4:NM_000092:exon21:c.C1444T:p.P482S	NA	rs2229814	B
chr2	2.31E+08	2.31E+08	T	C	UTR3	SP140	NA	NA	NA	rs933957	NA
chr2	2.35E+08	2.35E+08	AAACAAAC	-	UTR3	HJURP	NA	NA	NA	rs150635580	NA
chr2	2.35E+08	2.35E+08	-	TTAT	UTR3	TRPM8	NA	NA	NA	rs138810119	NA
chr2	2.35E+08	2.35E+08	T	-	UTR3	ARL4C	NA	NA	NA	rs11476419	NA
chr2	2.39E+08	2.39E+08	C	A	exonic	ESPNL	synonymous SNV	ESPNL:NM_194312:exon2:c.C403A:p.R135R	NA	rs34878344	NA
chr2	2.4E+08	2.4E+08	-	ATAGATAG	UTR3	HDAC4	NA	NA	NA	NA	NA
chr2	2.4E+08	2.4E+08	G	A	exonic	HDAC4	synonymous SNV	HDAC4:NM_006037:exon21:c.C2565T:p.P855P	NA	rs1063639	NA
chr2	2.41E+08	2.41E+08	C	T	exonic	PRR21	nonsynonymous SNV	PRR21:NM_001080835:exon1:c.G712A:p.V238I	NA	rs199895422	NA
chr2	2.41E+08	2.41E+08	C	T	exonic	PRR21	nonsynonymous SNV	PRR21:NM_001080835:exon1:c.G292A:p.V98I	D=COSM1129269,COSM1129268;OCCURENCE=1(prostate	rs112152720	B
chr2	2.41E+08	2.41E+08	G	A	exonic	ANKMY1	nonsynonymous SNV	ANKMY1:NM_016552:exon10:c.C1946T:p.T649M	NA	rs35044862	B
chr2	2.42E+08	2.42E+08	C	T	exonic	PASK	nonsynonymous SNV	52124:exon6:c.G748A:p.V250I,PASK:NM_015148:exon6:c	ID=COSM149119,COSM149120;OCCURENCE=1(stomach)	rs1470414	B
chr2	2.43E+08	2.43E+08	A	C	UTR3	BOK	NA	NA	NA	rs12993567	NA
chr2	2.43E+08	2.43E+08	T	A	ncRNA_exonic	LOC728323	NA	NA	NA	rs1817840	NA
chr3	5222488	5222488	-	AGGGGATA	UTR3	ARL8B	NA	NA	NA	rs143599604	NA
chr3	9519364	9519364	T	-	UTR3	SETD5	NA	NA	NA	rs58994037	NA
chr3	14105964	14105964	T	C	ncRNA_exonic	TPRXL	NA	NA	NA	NA	NA
chr3	15469001	15469001	A	C	UTR5	METTL6	NA	NA	NA	rs2290536	NA
chr3	18427924	18427924	G	T	exonic	SATB1	synonymous SNV	P462P,SATB1:NM_001195470:exon8:c.C1386A:p.P462P,SA	NA	rs2229261	NA
chr3	19988758	19988758	-	CGG	UTR5	RAB5A	NA	NA	NA	NA	NA
chr3	20212189	20212189	A	G	UTR3	SGOL1	NA	NA	NA	rs1052930	NA
chr3	33174156	33174156	T	G	exonic	CRTAP	synonymous SNV	CRTAP:NM_006371:exon5:c.T1032G:p.T344T	ID=COSM149349;OCCURENCE=1(stomach)	rs1135127	NA
chr3	33174168	33174168	G	A	exonic	CRTAP	synonymous SNV	CRTAP:NM_006371:exon5:c.G1044A:p.S348S	NA	rs1135128	NA
chr3	37028931	37028931	-	T	UTR3	EPM2AIP1	NA	NA	NA	rs199570773	NA
chr3	38493852	38493852	C	A	ncRNA_exonic	ACVR2B-AS1	NA	NA	NA	rs3762788	NA
chr3	38908944	38908944	T	C	exonic	SCN11A	synonymous SNV	SCN11A:NM_014139:exon23:c.A3819G:p.E1273E	NA	rs148945365	NA
chr3	41281460	41281460	C	T	UTR3	CTNNB1	NA	NA	NA	NA	NA
chr3	41281464	41281464	C	T	UTR3	CTNNB1	NA	NA	NA	NA	NA
chr3	46945263	46945263	-	A	UTR3	PTH1R	NA	NA	NA	NA	NA
chr3	49273996	49273996	C	T	exonic	CCDC36	synonymous SNV	1135197:exon3:c.C72T:p.S24S,CCDC36:NM_178173:exon	NA	rs12631989	NA
chr3	49315576	49315576	T	-	UTR3	USP4	NA	NA	NA	rs11309567	NA
chr3	54354510	54354510	T	C	exonic	CACNA2D3	synonymous SNV	CACNA2D3:NM_018398:exon3:c.T246C:p.I82I	NA	rs9879885	NA
chr3	58304608	58304608	C	G	UTR3	RPP14	NA	NA	NA	rs3210776	NA
chr3	59908126	59908126	A	G	exonic	FHIT	synonymous SNV	166243:exon8:c.T294C:p.H98H,FHIT:NM_002012:exon8:c.	ID=COSM149406;OCCURENCE=1(stomach)	rs1385816	NA
chr3	74344356	74344356	T	G	exonic	CNTN3	synonymous SNV	CNTN3:NM_020872:exon18:c.A2433C:p.A811A	NA	rs10490832	NA

chr3	75715734	75715734	G	A	ncRNA_exonic	FLJ20518	NA	NA	NA	rs138571914	NA
chr3	75716122	75716122	T	C	ncRNA_exonic	FLJ20518	NA	NA	NA	rs139598107	NA
chr3	75716303	75716303	T	A	ncRNA_exonic	FLJ20518	NA	NA	NA	rs150165388	NA
chr3	75786288	75786288	A	G	exonic	ZNF717	nonsynonymous SNV	ZNF717:NM_001128223:exon5:c.T2486C:p.L829P	D=COSM1191698;OCCURENCE=1(central_nervous_system	rs147227180	NA
chr3	75786298	75786298	T	C	exonic	ZNF717	nonsynonymous SNV	ZNF717:NM_001128223:exon5:c.A2476G:p.K826E	NA	rs140045886	NA
chr3	75787202	75787202	G	T	exonic	ZNF717	synonymous SNV	ZNF717:NM_001128223:exon5:c.C1572A:p.V524V	NA	rs76713735	NA
chr3	75788199	75788199	T	C	exonic	ZNF717	nonsynonymous SNV	ZNF717:NM_001128223:exon5:c.A575G:p.H192R	NA	rs146090776	NA
chr3	1.13E+08	1.13E+08	A	-	UTR3	GTPBP8	NA	NA	NA	rs200301737	NA
chr3	1.19E+08	1.19E+08	C	G	exonic	C3orf30	nonsynonymous SNV	C3orf30:NM_152539:exon2:c.C1419G:p.D473E	NA	rs9289122	B
chr3	1.22E+08	1.22E+08	A	G	UTR3	CASR	NA	NA	NA	rs9740	NA
chr3	1.24E+08	1.24E+08	T	C	exonic	KALRN	synonymous SNV	54:exon2:c.T243C:p.L81L,KALRN:NM_001024660:exon35:c	NA	rs1660038	NA
chr3	1.29E+08	1.29E+08	C	T	exonic	EFCAB12	nonsynonymous SNV	EFCAB12:NM_207307:exon3:c.G590A:p.R197H	NA	rs62266958	NA
chr3	1.29E+08	1.29E+08	G	A	UTR5	EFCAB12	NA	NA	NA	rs3796391	NA
chr3	1.3E+08	1.3E+08	G	A	exonic	ALG1L2	synonymous SNV	ALG1L2:NM_001136152:exon2:c.G45A:p.P15P	NA	rs6804080	NA
chr3	1.3E+08	1.3E+08	T	-	intronic	COL6A5	NA	NA	NA	rs11355796	NA
chr3	1.36E+08	1.36E+08	G	A	UTR3	MSL2	NA	NA	NA	NA	NA
chr3	1.36E+08	1.36E+08	G	A	UTR3	MSL2	NA	NA	NA	rs78145147	NA
chr3	1.42E+08	1.42E+08	T	-	UTR3	GK5	NA	NA	NA	rs139089663	NA
chr3	1.42E+08	1.42E+08	A	-	UTR3	GK5	NA	NA	NA	rs137909394	NA
chr3	1.5E+08	1.5E+08	AAA	-	UTR3	TSC22D2	NA	NA	NA	rs71739355	NA
chr3	1.59E+08	1.59E+08	A	G	exonic	MFSD1	nonsynonymous SNV	67903:exon7:c.A688G:p.I230V,MFSD1:NM_022736:exon8	NA	rs3765083	B
chr3	1.65E+08	1.65E+08	C	T	UTR3	BCHE	NA	NA	NA	rs3495	NA
chr3	1.67E+08	1.67E+08	T	A	exonic	ZBBX	nonsynonymous SNV	K160N,ZBBX:NM_001199202:exon9:c.A393T:p.K131N,ZBI	NA	rs4619784	P
chr3	1.67E+08	1.67E+08	A	G	exonic	WDR49	nonsynonymous SNV	WDR49:NM_178824:exon14:c.T1952C:p.L651P	NA	rs13060964	B
chr3	1.71E+08	1.71E+08	A	G	exonic	PLD1	synonymous SNV	)81:exon16:c.T1770C:p.R590R,PLD1:NM_002662:exon17:	NA	rs2124147	NA
chr3	1.86E+08	1.86E+08	G	-	UTR3	ETV5	NA	NA	NA	rs200001879	NA
chr3	1.86E+08	1.86E+08	A	G	UTR3	DGKG	NA	NA	NA	rs13089860	NA
chr3	1.86E+08	1.86E+08	T	G	UTR3	TBCCD1	NA	NA	NA	rs4686430	NA
chr3	1.87E+08	1.87E+08	C	T	UTR3	RTP4	NA	NA	NA	rs9028	NA
chr3	1.88E+08	1.88E+08	G	A	ncRNA_exonic	FLJ42393	NA	NA	NA	rs344952	NA
chr3	1.94E+08	1.94E+08	G	A	UTR3	CPN2	NA	NA	NA	rs1048996	NA
chr3	1.94E+08	1.94E+08	-	G	UTR3	ATP13A3	NA	NA	NA	NA	NA
chr3	1.95E+08	1.95E+08	G	A	UTR3	XXYLT1	NA	NA	NA	rs2720930	NA
chr3	1.95E+08	1.95E+08	-	GGCG	UTR3	APOD	NA	NA	NA	rs146445395	NA
chr3	1.95E+08	1.95E+08	T	G	UTR3	APOD	NA	NA	NA	NA	NA
chr3	1.96E+08	1.96E+08	C	T	exonic	MUC4	nonsynonymous SNV	37D,MUC4:NM_004532:exon3:c.G263A:p.G88D,MUC4:NI	NA	rs2259292	NA
chr3	1.96E+08	1.96E+08	G	T	exonic	MUC4	nonsynonymous SNV	MUC4:NM_018406:exon2:c.C11200A:p.P3734T	NA	rs201456607	NA
chr3	1.96E+08	1.96E+08	G	C	exonic	MUC4	synonymous SNV	MUC4:NM_018406:exon2:c.C10653G:p.T3551T	NA	NA	NA
chr3	1.96E+08	1.96E+08	C	G	exonic	MUC4	nonsynonymous SNV	MUC4:NM_018406:exon2:c.G9903C:p.E3301D	NA	NA	NA
chr3	1.96E+08	1.96E+08	A	G	exonic	MUC4	nonsynonymous SNV	MUC4:NM_018406:exon2:c.T9290C:p.V3097A	NA	rs79582237	NA
chr3	1.96E+08	1.96E+08	C	T	exonic	MUC4	nonsynonymous SNV	MUC4:NM_018406:exon2:c.G8656A:p.A2886T	NA	rs201497773	NA
chr3	1.96E+08	1.96E+08	T	C	exonic	MUC4	nonsynonymous SNV	MUC4:NM_018406:exon2:c.A8200G:p.T2734A	NA	NA	NA
chr3	1.96E+08	1.96E+08	A	G	exonic	MUC4	synonymous SNV	MUC4:NM_018406:exon2:c.T5700C:p.G1900G	NA	NA	NA
chr3	1.96E+08	1.96E+08	A	G	exonic	MUC4	nonsynonymous SNV	MUC4:NM_018406:exon2:c.T4318C:p.S1440P	NA	NA	NA
chr3	1.96E+08	1.96E+08	A	G	exonic	MUC4	synonymous SNV	MUC4:NM_018406:exon2:c.T3996C:p.T1332T	NA	rs79619815	NA
chr3	1.96E+08	1.96E+08	C	G	exonic	MUC4	nonsynonymous SNV	MUC4:NM_018406:exon2:c.G3981C:p.M1327I	NA	rs200851738	NA
chr3	1.96E+08	1.96E+08	T	A	exonic	MUC4	synonymous SNV	MUC4:NM_018406:exon2:c.A3723T:p.A1241A	NA	NA	NA
chr4	844781	844781	G	A	exonic	GAK	synonymous SNV	GAK:NM_005255:exon26:c.C3600T:p.T1200T	ID=COSM287830;OCCURENCE=1(large_intestine)	rs75072999	NA
chr4	860192	860192	A	G	exonic	GAK	synonymous SNV	GAK:NM_005255:exon22:c.T3003C:p.S1001S	NA	rs1064207	NA
chr4	1191486	1191486	-	T	ncRNA_exonic	LOC100130872	NA	NA	NA	NA	NA
chr4	2252201	2252201	C	T	UTR3	MXD4	NA	NA	NA	rs1052682	NA
chr4	7044357	7044357	A	G	exonic	CCDC96	synonymous SNV	CCDC96:NM_153376:exon1:c.T309C:p.V103V	NA	rs871133	NA
chr4	17634213	17634213	A	G	exonic	FAM184B	nonsynonymous SNV	FAM184B:NM_015688:exon18:c.T3125C:p.V1042A	NA	rs6825562	NA
chr4	20731213	20731213	-	TTTTAATCT	UTR3	KCNIP4	NA	NA	NA	NA	NA
chr4	20731232	20731242	AAATGGAAT	-	UTR3	KCNIP4	NA	NA	NA	rs151224175	NA
chr4	20852244	20852244	A	G	exonic	KCNIP4	synonymous SNV	001035004:exon2:c.T24C:p.P8P,KCNIP4:NM_025221:exon	NA	rs3765122	NA
chr4	21846581	21846581	-	AAA	ncRNA_exonic	KCNIP4-IT1	NA	NA	NA	NA	NA
chr4	31147973	31147980	AAATAAAT	-	UTR3	PCDH7	NA	NA	NA	rs145102344	NA
chr4	37613930	37613930	G	A	UTR3	RELL1	NA	NA	NA	rs17495047	NA
chr4	39451560	39451565	AATAAA	-	UTR3	KLB	NA	NA	NA	rs145759953	NA
chr4	39458051	39458051	A	G	exonic	RPL9	synonymous SNV	24921:exon4:c.T366C:p.Y122Y,RPL9:NM_000661:exon5:c	NA	rs1065744	NA
chr4	39478946	39478946	C	T	UTR3	LIAS	NA	NA	NA	rs11934814	NA

chr4	54876132	54876132	C	-	UTR3	CHIC2	NA	NA	NA	rs68107873	NA
chr4	56899377	56899377	A	G	UTR3	CEP135	NA	NA	NA	rs12512313	NA
chr4	57326996	57326996	C	T	UTR3	PAICS	NA	NA	NA	rs1140982	NA
chr4	57368635	57368662	'ATATGTAT/	-	UTR3	SRP72	NA	NA	NA	rs200531104	NA
chr4	71117016	71117016	T	G	UTR3	CSN3	NA	NA	NA	rs62308392	NA
chr4	72435944	72435973	TTCAGCCTC	-	UTR3	SLC4A4	NA	NA	NA	NA	NA
chr4	76720885	76720885	T	C	exonic	USO1	unknown	UNKNOWN	NA	rs13119659	NA
chr4	76871270	76871270	C	A	UTR3	SDAD1	NA	NA	NA	NA	NA
chr4	77701688	77701688	A	-	UTR3	SHROOM3	NA	NA	NA	rs11325137	NA
chr4	88536880	88536880	C	T	exonic	DSPP	synonymous SNV	DSPP:NM_014208:exon5:c.C3066T:p.S1022S	NA	NA	NA
chr4	88537180	88537180	T	C	exonic	DSPP	synonymous SNV	DSPP:NM_014208:exon5:c.T3366C:p.N1122N	NA	NA	NA
chr4	88537294	88537294	T	C	exonic	DSPP	synonymous SNV	DSPP:NM_014208:exon5:c.T3480C:p.S1160S	NA	NA	NA
chr4	91759710	91759710	-	A	intronic	CCSER1	NA	NA	NA	NA	NA
chr4	99850451	99850451	T	-	UTR5	EIF4E	NA	NA	NA	NA	NA
chr4	1E+08	1E+08	A	G	exonic	ADH1B	synonymous SNV	ADH1B:NM_000668:exon6:c.T753C:p.I251I	NA	rs1789882	NA
chr4	1E+08	1E+08	C	T	exonic	ADH1C	unknown	UNKNOWN	NA	rs1693425	NA
chr4	1E+08	1E+08	-	T	UTR3	TRMT10A	NA	NA	NA	rs149160816	NA
chr4	1.11E+08	1.11E+08	T	C	exonic	ENPEP	nonsynonymous SNV	ENPEP:NM_001977:exon2:c.T653C:p.V218A	ID=COSM1131302;OCCURENCE=1(prostate)	rs1126483	B
chr4	1.15E+08	1.15E+08	CAAATTAG(	-	UTR3	ARSJ	NA	NA	NA	NA	NA
chr4	1.23E+08	1.23E+08	T	C	UTR3	TMEM155	NA	NA	NA	rs4342205	NA
chr4	1.24E+08	1.24E+08	G	A	UTR3	SPATA5	NA	NA	NA	rs2132078	NA
chr4	1.47E+08	1.47E+08	A	-	UTR3	MMAA	NA	NA	NA	rs33978754	NA
chr4	1.54E+08	1.54E+08	T	G	exonic	TRIM2	synonymous SNV	30067:exon3:c.T324G:p.T108T,TRIM2:NM_015271:exon3	NA	rs2289409	NA
chr4	1.56E+08	1.56E+08	A	G	UTR5	MAP9	NA	NA	ID=COSN404819;OCCURENCE=1(lung)	rs17377679	NA
chr4	1.6E+08	1.6E+08	C	T	UTR5	PPID	NA	NA	NA	rs2070629	NA
chr4	1.6E+08	1.6E+08	A	-	UTR3	FNIP2	NA	NA	NA	rs11340968	NA
chr4	1.76E+08	1.76E+08	G	T	UTR5	ADAM29	NA	NA	NA	rs7689299	NA
chr4	1.76E+08	1.76E+08	C	T	UTR5	ADAM29	NA	NA	NA	rs62334419	NA
chr4	1.77E+08	1.77E+08	A	-	UTR3	WDR17	NA	NA	NA	rs70962454	NA
chr4	1.77E+08	1.77E+08	A	G	exonic	SPATA4	synonymous SNV	SPATA4:NM_144644:exon4:c.T510C:p.Y170Y	NA	rs2291244	NA
chr4	1.84E+08	1.84E+08	G	T	exonic	WWC2	nonsynonymous SNV	WWC2:NM_024949:exon16:c.G2446T:p.V816F	NA	rs11734376	NA
chr4	1.86E+08	1.86E+08	-	ATT	UTR3	CASP3	NA	NA	NA	rs4647696	NA
chr4	1.86E+08	1.86E+08	G	A	exonic	CCDC111	synonymous SNV	CCDC111:NM_152683:exon8:c.G948A:p.Q316Q	NA	rs34985821	NA
chr5	796054	796055	CT	-	UTR3	ZDHHC11	NA	NA	NA	NA	NA
chr5	796106	796106	T	C	UTR3	ZDHHC11	NA	NA	NA	NA	NA
chr5	1222213	1222214	TG	-	UTR3	SLC6A19	NA	NA	NA	rs35482871	NA
chr5	10657091	10657091	-	AA	UTR3	ANKRD33B	NA	NA	NA	NA	NA
chr5	10972889	10972889	T	-	UTR3	CTNND2	NA	NA	NA	rs70947237	NA
chr5	13701536	13701536	T	C	exonic	DNAH5	nonsynonymous SNV	DNAH5:NM_001369:exon77:c.A13348G:p.I4450V	NA	rs3734110	B
chr5	16673975	16673975	C	G	exonic	MYO10	nonsynonymous SNV	MYO10:NM_012334:exon36:c.G4988C:p.S1663T	NA	rs25901	NA
chr5	31317952	31317952	T	C	exonic	CDH6	synonymous SNV	CDH6:NM_004932:exon11:c.T1803C:p.H601H	NA	rs2229575	NA
chr5	40964885	40964885	A	T	exonic	C7	nonsynonymous SNV	C7:NM_000587:exon14:c.A1792T:p.T598S	NA	rs60714178	NA
chr5	42756940	42756940	C	T	UTR5	CCDC152	NA	NA	NA	rs3797310	NA
chr5	55206444	55206444	G	A	exonic	IL31RA	nonsynonymous SNV	;29N,IL31RA:NM_139017:exon12:c.G1586A:p.S529N,IL31	NA	rs161704	P
chr5	59825300	59825300	T	C	ncRNA_exonic	PART1	NA	NA	NA	rs26949	NA
chr5	59842971	59842971	A	G	ncRNA_exonic	PART1	NA	NA	NA	rs153152	NA
chr5	64963082	64963082	-	CT	UTR3	SGTB	NA	NA	NA	rs2067306	NA
chr5	64963466	64963469	TTTG	-	UTR3	SGTB	NA	NA	NA	rs33916729	NA
chr5	68739819	68739819	G	A	intergenic	!(dist=1929),OCLN(c	NA	NA	NA	NA	NA
chr5	75948650	75948650	A	G	exonic	IQGAP2	nonsynonymous SNV	IQGAP2:NM_006633:exon18:c.A2170G:p.I724V	NA	rs2431363	B
chr5	76003258	76003258	C	T	UTR3	IQGAP2	NA	NA	NA	rs464494	NA
chr5	78181577	78181577	T	C	exonic	ARSB	synonymous SNV	)46:exon5:c.A972G:p.G324G,ARSB:NM_198709:exon6:c.A	NA	rs72762973	NA
chr5	79048562	79048562	C	T	exonic	CMYA5	synonymous SNV	CMYA5:NM_153610:exon6:c.C11055T:p.F3685F	ID=COSM149872;OCCURENCE=1(stomach)	rs1366272	NA
chr5	82936729	82936729	T	-	UTR3	HAPLN1	NA	NA	NA	NA	NA
chr5	95115959	95115959	C	T	exonic	RHOBTB3	nonsynonymous SNV	RHOBTB3:NM_014899:exon9:c.C1286T:p.T429M	NA	rs41276257	P
chr5	96111228	96111228	A	G	UTR3	ERAP1	NA	NA	NA	rs3198304	NA
chr5	96111801	96111801	-	TG	UTR3	ERAP1	NA	NA	NA	rs148692674	NA
chr5	96111927	96111927	-	CATTCATG/	UTR3	ERAP1	NA	NA	NA	rs142866043	NA
chr5	96322360	96322360	G	A	exonic	LNPEP	nonsynonymous SNV	;75:exon4:c.G1117A:p.V373I,LNPEP:NM_175920:exon4:c.	NA	rs41276279	B
chr5	96429323	96429323	-	A	UTR3	LIX1	NA	NA	NA	NA	NA
chr5	1.22E+08	1.22E+08	CACACACA	-	UTR3	SNX2	NA	NA	NA	rs138443824	NA
chr5	1.26E+08	1.26E+08	-	TTT	UTR3	ALDH7A1	NA	NA	NA	NA	NA

chr5	1.28E+08	1.28E+08	G	C	exonic	FBN2	synonymous SNV	FBN2:NM_001999:exon1:c.C183G:p.P61P	NA	rs73348287	NA
chr5	1.31E+08	1.31E+08	ACACACAC	-	UTR3	LYRM7	NA	NA	NA	rs36230431	NA
chr5	1.31E+08	1.31E+08	G	T	UTR3	CDC42SE2	NA	NA	NA	NA	NA
chr5	1.32E+08	1.32E+08	C	T	exonic	SLC22A4	nonsynonymous SNV	SLC22A4:NM_003059:exon9:c.C1507T:p.L503F	NA	rs1050152	B
chr5	1.32E+08	1.32E+08	A	G	UTR3	UQCRQ	NA	NA	NA	rs17166297	NA
chr5	1.32E+08	1.32E+08	G	A	UTR3	UQCRQ	NA	NA	NA	rs17624157	NA
chr5	1.36E+08	1.36E+08	-	AA	UTR3	SMAD5	NA	NA	NA	NA	NA
chr5	1.37E+08	1.37E+08	G	A	exonic	PKD2L2	nonsynonymous SNV	8448:exon8:c.G1144A:p.V382I,PKD2L2:NM_014386:exon	NA	rs1880458	B
chr5	1.39E+08	1.39E+08	T	A	UTR5	MATR3	NA	NA	NA	rs12153162	NA
chr5	1.41E+08	1.41E+08	A	C	UTR3	PCDHB6	NA	NA	NA	rs56172324	NA
chr5	1.41E+08	1.41E+08	C	T	UTR3	PCDHB9	NA	NA	NA	rs2907325	NA
chr5	1.44E+08	1.44E+08	T	-	UTR3	YIPF5	NA	NA	NA	rs144356469	NA
chr5	1.45E+08	1.45E+08	C	T	exonic	SH3RF2	nonsynonymous SNV	SH3RF2:NM_152550:exon5:c.C799T:p.R267C	NA	rs758037	D
chr5	1.46E+08	1.46E+08	G	A	exonic	LARS	synonymous SNV	LARS:NM_020117:exon28:c.C2889T:p.N963N	NA	rs11540216	NA
chr5	1.46E+08	1.46E+08	G	A	exonic	LARS	synonymous SNV	LARS:NM_020117:exon19:c.C1747T:p.L583L	NA	rs11540217	NA
chr5	1.46E+08	1.46E+08	A	G	exonic	TCERG1	synonymous SNV	06:exon21:c.A3057G:p.S1019S,TCERG1:NM_006706:exon	NA	rs4705103	NA
chr5	1.51E+08	1.51E+08	A	G	exonic	GM2A	nonsynonymous SNV	05:exon2:c.A205G:p.M69V,GM2A:NM_001167607:exon2	NA	rs153478	B
chr5	1.51E+08	1.51E+08	G	A	exonic	SLC36A2	synonymous SNV	SLC36A2:NM_181776:exon2:c.C187T:p.L63L	NA	rs192192	NA
chr5	1.54E+08	1.54E+08	C	T	UTR3	FAXDC2	NA	NA	NA	rs4865	NA
chr5	1.54E+08	1.54E+08	C	T	UTR3	MRPL22	NA	NA	NA	rs286595	NA
chr5	1.59E+08	1.59E+08	A	-	UTR5	EBF1	NA	NA	NA	rs5872586	NA
chr5	1.6E+08	1.6E+08	-	A	UTR3	PWWP2A	NA	NA	NA	NA	NA
chr5	1.72E+08	1.72E+08	C	T	exonic	STK10	synonymous SNV	STK10:NM_005990:exon6:c.G756A:p.S252S	NA	rs2306962	NA
chr5	1.75E+08	1.75E+08	G	A	exonic	THOC3	synonymous SNV	THOC3:NM_032361:exon2:c.C357T:p.S119S	NA	rs200408233	NA
chr5	1.76E+08	1.76E+08	A	G	UTR3	TSPAN17	NA	NA	NA	NA	NA
chr5	1.77E+08	1.77E+08	A	C	intronic	FAM193B	NA	NA	NA	NA	NA
chr5	1.78E+08	1.78E+08	T	C	exonic	RMND5B	synonymous SNV	RMND5B:NM_022762:exon9:c.T888C:p.P296P	NA	rs61751561	NA
chr5	1.78E+08	1.78E+08	T	C	exonic	ZNF354B	synonymous SNV	ZNF354B:NM_058230:exon4:c.T243C:p.G81G	ID=COSM150026;OCCURENCE=1(stomach)	rs11952817	NA
chr5	1.79E+08	1.79E+08	G	A	exonic	SQSTM1	synonymous SNV	R,SQSTM1:NM_001142298:exon7:c.G684A:p.R228R,SQST	NA	rs4797	NA
chr5	1.8E+08	1.8E+08	A	G	intronic	ZFP62	NA	NA	NA	rs2127380	NA
chr6	6007196	6007196	G	-	UTR5	NRN1	NA	NA	NA	rs11285278	NA
chr6	7283735	7283735	C	T	UTR3	SSR1	NA	NA	NA	rs111496266	NA
chr6	7283759	7283759	T	C	UTR3	SSR1	NA	NA	NA	rs9505115	NA
chr6	7288297	7288297	G	A	UTR3	SSR1	NA	NA	NA	rs8955	NA
chr6	7289958	7289959	CA	-	UTR3	SSR1	NA	NA	NA	rs16740	NA
chr6	7576527	7576527	G	A	exonic	DSP	synonymous SNV	44:exon19:c.G2631A:p.R877R,DSP:NM_004415:exon19:c	NA	rs1016835	NA
chr6	7611993	7611998	TTTCTG	-	UTR3	SNRNP48	NA	NA	NA	rs146960918	NA
chr6	11714214	11714215	AA	-	UTR3	ADTRP	NA	NA	NA	NA	NA
chr6	13579828	13579828	A	C	UTR5	SIRT5	NA	NA	NA	rs2804911	NA
chr6	15521302	15521302	-	AAA	UTR3	JARID2	NA	NA	NA	rs34405141	NA
chr6	15523807	15523807	T	A	UTR3	DTNBP1	NA	NA	NA	rs55944541	NA
chr6	16327864	16327864	-	TGC	exonic	ATXN1	nonframeshift insertion	8_679insGCA:p.H226delinsQH,ATXN1:NM_000332:exon8	NA	NA	NA
chr6	17799575	17799575	A	G	exonic	KIF13A	synonymous SNV	567:exon22:c.T2712C:p.A904A,KIF13A:NM_001105568:ex	NA	rs3734235	NA
chr6	18467582	18467582	A	G	UTR3	RNF144B	NA	NA	NA	rs531972	NA
chr6	20534770	20534770	C	T	UTR5	CDKAL1	NA	NA	NA	NA	NA
chr6	21201493	21201493	G	A	exonic	CDKAL1	synonymous SNV	CDKAL1:NM_017774:exon15:c.G1536A:p.S512S	NA	rs9465994	NA
chr6	21594175	21594175	C	T	UTR5	SOX4	NA	NA	NA	NA	NA
chr6	21597712	21597712	A	T	UTR3	SOX4	NA	NA	NA	NA	NA
chr6	24805465	24805466	AA	-	UTR3	FAM65B	NA	NA	NA	rs67150453	NA
chr6	29640095	29640111	AATAAATA	-	UTR3	MOG	NA	NA	NA	NA	NA
chr6	29867070	29867070	T	A	intergenic	st=8214),HCG4B(dis	NA	NA	NA	rs9259499	NA
chr6	29867247	29867247	A	C	intergenic	st=8391),HCG4B(dis	NA	NA	NA	rs3128995	NA
chr6	29911970	29911970	G	A	exonic	HLA-A	nonsynonymous SNV	42758:exon4:c.G691A:p.G231S,HLA-A:NM_002116:exon4	NA	rs1059578	B
chr6	29912087	29912087	G	T	exonic	HLA-A	nonsynonymous SNV	42758:exon4:c.G808T:p.A270S,HLA-A:NM_002116:exon4	NA	rs1059632	B
chr6	30078330	30078330	C	T	exonic	TRIM31	synonymous SNV	TRIM31:NM_007028:exon4:c.G639A:p.A213A	NA	rs2239529	NA
chr6	30232538	30232538	G	T	ncRNA_exonic	HLA-L	NA	NA	NA	rs28780091	NA
chr6	30232672	30232672	C	T	ncRNA_exonic	HLA-L	NA	NA	NA	rs3129701	NA
chr6	30954334	30954334	C	A	exonic	MUC21	nonsynonymous SNV	MUC21:NM_001010909:exon2:c.C382A:p.P128T	ID=COSM1186994;OCCURENCE=1(lung)	rs143481246	NA
chr6	31026534	31026534	C	T	ncRNA_intronic	HCG22	NA	NA	NA	rs2523847	NA
chr6	31088202	31088202	A	G	UTR5	CDSN	NA	NA	NA	rs2302399	NA
chr6	31237767	31237767	T	C	exonic	HLA-C	nonsynonymous SNV	3042:exon5:c.A991G:p.M331V,HLA-C:NM_002117:exon5	NA	rs1130935	B
chr6	31237774	31237774	G	C	exonic	HLA-C	synonymous SNV	3042:exon5:c.C984G:p.V328V,HLA-C:NM_002117:exon5	NA	rs41540512	NA

chr6	31905130	31905130	G	A	exonic	C2	synonymous SNV	.A127A,C2:NM_001145903:exon6:c.G627A:p.A209A,C2:N	NA	rs1042663	NA
chr6	32714901	32714901	C	T	downstream	HLA-DQA2	NA	NA	NA	rs9276442	NA
chr6	33037419	33037419	G	A	exonic	HLA-DPA1	synonymous SNV	,HLA-DPA1:NM_001242524:exon3:c.C345T:p.N115N,HLA	NA	rs1126543	NA
chr6	33037424	33037424	T	C	exonic	HLA-DPA1	nonsynonymous SNV	,HLA-DPA1:NM_001242524:exon3:c.A340G:p.T114A,HLA	NA	rs1126542	B
chr6	33037639	33037639	G	A	exonic	HLA-DPA1	nonsynonymous SNV	V,HLA-DPA1:NM_001242524:exon3:c.C125T:p.A42V,HLA-	ID=COSM328641;OCCURENCE=4(kidney)	rs1126534	B
chr6	33037640	33037640	C	T	exonic	HLA-DPA1	nonsynonymous SNV	T,HLA-DPA1:NM_001242524:exon3:c.G124A:p.A42T,HLA-	NA	rs1126533	B
chr6	33054331	33054331	T	C	UTR3	HLA-DPB1	NA	NA	NA	rs1126723	NA
chr6	33054550	33054550	G	A	UTR3	HLA-DPB1	NA	NA	NA	rs931	NA
chr6	33054552	33054552	C	G	UTR3	HLA-DPB1	NA	NA	NA	rs928	NA
chr6	33179686	33179686	T	G	exonic	RING1	synonymous SNV	RING1:NM_002931:exon6:c.T1026G:p.G342G	NA	NA	NA
chr6	35549193	35549193	-	CATGGTGA	UTR3	FKBP5	NA	NA	NA	NA	NA
chr6	39048491	39048491	A	C	exonic	GLP1R	synonymous SNV	GLP1R:NM_002062:exon12:c.A1200C:p.I400I	NA	rs1126476	NA
chr6	43586698	43586698	-	G	UTR3	POLH	NA	NA	NA	NA	NA
chr6	45517740	45517741	TT	-	UTR3	RUNX2	NA	NA	NA	NA	NA
chr6	46726915	46726915	C	A	UTR3	ANKRD66	NA	NA	NA	rs9472840	NA
chr6	52360331	52360331	G	A	UTR3	EFHC1	NA	NA	NA	rs3789771	NA
chr6	57512775	57512775	T	G	UTR3	PRIM2	NA	NA	NA	rs7773110	NA
chr6	57512779	57512779	G	T	UTR3	PRIM2	NA	NA	NA	rs76296076	NA
chr6	57512903	57512903	C	A	UTR3	PRIM2	NA	NA	NA	rs75209982	NA
chr6	57513126	57513126	C	G	UTR3	PRIM2	NA	NA	NA	rs56375552	NA
chr6	57513182	57513182	A	G	UTR3	PRIM2	NA	NA	NA	rs1801400	NA
chr6	87993555	87993555	T	-	UTR3	GJB7	NA	NA	NA	NA	NA
chr6	89888744	89888744	C	T	exonic	GABRR1	synonymous SNV	:exon10:c.G1185A:p.A395A,GABRR1:NM_001256704:exo	NA	rs1796743	NA
chr6	97592942	97592942	-	C	ncRNA_UTR3	MMS22L	NA	NA	NA	NA	NA
chr6	99956560	99956560	T	C	exonic	USP45	nonsynonymous SNV	USP45:NM_001080481:exon3:c.A199G:p.K67E	ID=COSM150144;OCCURENCE=1(stomach)	rs7744845	D
chr6	1.07E+08	1.07E+08	G	A	UTR5	RTN4IP1	NA	NA	NA	rs9384625	NA
chr6	1.07E+08	1.07E+08	AA	-	UTR3	BEND3	NA	NA	NA	rs200868848	NA
chr6	1.08E+08	1.08E+08	T	G	UTR3	SEC63	NA	NA	NA	rs592989	NA
chr6	1.1E+08	1.1E+08	G	C	UTR3	PPIL6	NA	NA	NA	rs746794	NA
chr6	1.12E+08	1.12E+08	T	A	UTR3	LAMA4	NA	NA	NA	rs7758715	NA
chr6	1.12E+08	1.12E+08	C	T	UTR3	LAMA4	NA	NA	NA	rs7738331	NA
chr6	1.3E+08	1.3E+08	T	G	UTR3	L3MBTL3	NA	NA	NA	NA	NA
chr6	1.33E+08	1.33E+08	A	G	ncRNA_exonic	VNN3	NA	NA	NA	rs2294759	NA
chr6	1.35E+08	1.35E+08	-	T	UTR3	HBS1L	NA	NA	NA	NA	NA
chr6	1.39E+08	1.39E+08	AA	-	UTR3	NHSL1	NA	NA	NA	NA	NA
chr6	1.39E+08	1.39E+08	C	T	exonic	CCDC28A	synonymous SNV	CCDC28A:NM_015439:exon1:c.C126T:p.S42S	NA	rs11154999	NA
chr6	1.44E+08	1.44E+08	A	G	UTR3	ADAT2	NA	NA	NA	rs9496619	NA
chr6	1.51E+08	1.51E+08	A	G	UTR3	MTHFD1L	NA	NA	NA	rs7646	NA
chr6	1.51E+08	1.51E+08	G	A	UTR3	MTHFD1L	NA	NA	NA	rs7543	NA
chr6	1.54E+08	1.54E+08	C	T	exonic	OPRM1	synonymous SNV	OPRM1:NM_001008505:exon4:c.C1333T:p.L445L	NA	rs562859	NA
chr6	1.55E+08	1.55E+08	G	A	exonic	TIAM2	synonymous SNV	TIAM2:NM_012454:exon7:c.G2034A:p.Q678Q	NA	rs1032141	NA
chr6	1.59E+08	1.59E+08	G	A	UTR3	GTF2H5	NA	NA	NA	rs9457163	NA
chr6	1.61E+08	1.61E+08	C	T	exonic	SLC22A2	synonymous SNV	SLC22A2:NM_003058:exon10:c.G1506A:p.V502V	NA	rs316003	NA
chr6	1.62E+08	1.62E+08	G	T	UTR3	PARK2	NA	NA	NA	rs68121389	NA
chr6	1.67E+08	1.67E+08	T	C	UTR5	T	NA	NA	NA	rs3099266	NA
chr6	1.68E+08	1.68E+08	G	C	UTR5	CCR6	NA	NA	NA	rs1012656	NA
chr6	1.68E+08	1.68E+08	T	C	exonic	TCP10L2	synonymous SNV	TCP10L2:NM_001145121:exon8:c.T1029C:p.H343H	NA	rs202096111	NA
chr6	1.68E+08	1.68E+08	G	A	exonic	FRMD1	synonymous SNV	FRMD1:NM_024919:exon1:c.C171T:p.L57L	NA	rs34054991	NA
chr6	1.7E+08	1.7E+08	C	T	exonic	THBS2	synonymous SNV	THBS2:NM_003247:exon6:c.G777A:p.S259S	NA	rs61730651	NA
chr7	537560	537560	T	A	UTR3	PDGFA	NA	NA	NA	NA	NA
chr7	2567705	2567705	A	G	UTR3	LFNG	NA	NA	NA	NA	NA
chr7	2567715	2567715	T	G	UTR3	LFNG	NA	NA	NA	NA	NA
chr7	9673930	9673930	G	-	ncRNA_exonic	PER4	NA	NA	NA	rs5882228	NA
chr7	9675063	9675063	C	A	ncRNA_exonic	PER4	NA	NA	NA	NA	NA
chr7	12370657	12370657	A	G	UTR3	VWDE	NA	NA	NA	rs2253353	NA
chr7	12370703	12370703	T	-	UTR3	VWDE	NA	NA	NA	rs3217131	NA
chr7	12370794	12370794	T	C	UTR3	VWDE	NA	NA	NA	rs1059002	NA
chr7	15725798	15725800	TGG	-	exonic	MEOX2	nonframeshift deletion	MEOX2:NM_005924:exon1:c.228_230del:p.76_77del	ID=COSM150907;OCCURENCE=1(stomach),1(oesophagus)	rs113582077	NA
chr7	15726117	15726117	-	TT	UTR5	MEOX2	NA	NA	NA	NA	NA
chr7	15726129	15726129	A	C	UTR5	MEOX2	NA	NA	NA	rs11975534	NA
chr7	16666759	16666759	G	C	exonic	ANKMY2	synonymous SNV	ANKMY2:NM_020319:exon3:c.C177G:p.L59L	NA	rs11531477	NA
chr7	21599233	21599233	C	T	exonic	DNAH11	synonymous SNV	DNAH11:NM_001277115:exon4:c.C705T:p.N235N	NA	rs10950854	NA

chr7	23531029	23531029	-	AA	ncRNA_exonic	RPS2P32	NA	NA	NA	NA	NA
chr7	25219341	25219341	C	T	UTR5	C7orf31	NA	NA	NA	rs117565579	NA
chr7	35131355	35131355	-	TG	ncRNA_intronic	DPY19L2P1	NA	NA	NA	rs146160027	NA
chr7	38725698	38725698	A	C	ncRNA_exonic	FAM183B	NA	NA	NA	NA	NA
chr7	44842433	44842434	AA	-	UTR3	PPIA	NA	NA	NA	rs17860097	NA
chr7	44868971	44868971	G	A	UTR3	H2AFV	NA	NA	NA	rs66566005	NA
chr7	45123457	45123457	C	T	exonic	NACAD	synonymous SNV	NACAD:NM_001146334:exon2:c.G2322A:p.S774S	NA	rs62457076	NA
chr7	65159983	65159983	A	G	ncRNA_exonic	INTS4L2	NA	NA	NA	rs147104756	NA
chr7	73818404	73818404	C	T	UTR3	CLIP2	NA	NA	NA	rs2240436	NA
chr7	75165018	75165018	G	A	UTR3	HIP1	NA	NA	NA	rs1167829	NA
chr7	76669085	76669085	T	A	ncRNA_exonic	LOC100132832	NA	NA	ID=COSN405014;OCCURENCE=1(lung)	NA	NA
chr7	91875204	91875204	T	C	UTR5	KRIT1	NA	NA	NA	NA	NA
chr7	92775019	92775019	T	C	UTR5	SAMD9L	NA	NA	NA	rs28662	NA
chr7	98792835	98792835	G	A	exonic	KPNA7	synonymous SNV	KPNA7:NM_001145715:exon4:c.C411T:p.A137A	NA	rs61751722	NA
chr7	1E+08	1E+08	T	C	exonic	ZAN	unknown	UNKNOWN	NA	NA	NA
chr7	1.01E+08	1.01E+08	C	G	exonic	MUC12	nonsynonymous SNV	MUC12:NM_001164462:exon2:c.C8078G:p.P2693R	NA	NA	NA
chr7	1.01E+08	1.01E+08	G	A	exonic	MUC12	synonymous SNV	MUC12:NM_001164462:exon2:c.G9999A:p.P3333P	NA	rs199843676	NA
chr7	1.01E+08	1.01E+08	G	A	exonic	COL26A1	unknown	UNKNOWN	NA	rs17393069	NA
chr7	1.03E+08	1.03E+08	A	G	exonic	RELN	synonymous SNV	:exon50:c.T7887C:p.P2629P,RELN:NM_173054:exon50:c.	NA	rs56345626	NA
chr7	1.08E+08	1.08E+08	A	G	UTR5	LAMB1	NA	NA	NA	rs2070917	NA
chr7	1.18E+08	1.18E+08	G	C	UTR5	NAA38	NA	NA	NA	rs2896242	NA
chr7	1.3E+08	1.3E+08	-	TG	UTR3	CPA4	NA	NA	NA	NA	NA
chr7	1.3E+08	1.3E+08	A	-	UTR3	COPG2	NA	NA	NA	rs61354155	NA
chr7	1.35E+08	1.35E+08	C	T	exonic	WDR91	synonymous SNV	WDR91:NM_014149:exon14:c.G2028A:p.S676S	NA	rs292557	NA
chr7	1.38E+08	1.38E+08	A	T	UTR3	SVOPL	NA	NA	NA	rs1439793	NA
chr7	1.42E+08	1.42E+08	T	C	exonic	PRSS2	unknown	UNKNOWN	NA	NA	NA
chr7	1.43E+08	1.43E+08	C	T	exonic	CLCN1	synonymous SNV	CLCN1:NM_000083:exon17:c.C2154T:p.D718D	NA	rs2272251	NA
chr7	1.43E+08	1.43E+08	T	C	exonic	EPHA1	synonymous SNV	EPHA1:NM_005232:exon17:c.A2742G:p.P914P	NA	rs1804527	NA
chr7	1.53E+08	1.53E+08	C	T	exonic	ACTR3B	nonsynonymous SNV	0135:exon8:c.C748T:p.R250W,ACTR3B:NM_020445:exon	NA	rs140327402	B
chr7	1.55E+08	1.55E+08	T	G	UTR3	CNPY1	NA	NA	NA	rs112409058	NA
chr7	1.55E+08	1.55E+08	A	G	UTR5	CNPY1	NA	NA	NA	rs6947243	NA
chr8	1733482	1733482	C	G	UTR3	CLN8	NA	NA	NA	rs111861349	NA
chr8	6793581	6793581	A	G	exonic	DEFA4	synonymous SNV	DEFA4:NM_001925:exon3:c.T255C:p.G85G	NA	rs2738100	NA
chr8	10466031	10466031	A	C	exonic	RP1L1	nonsynonymous SNV	RP1L1:NM_178857:exon4:c.T5577G:p.D1859E	ID=COSM748980;OCCURENCE=1(lung)	NA	NA
chr8	12886083	12886083	C	T	UTR3	KIAA1456	NA	NA	NA	rs7464342	NA
chr8	22069386	22069386	T	C	UTR3	BMP1	NA	NA	NA	rs7001	NA
chr8	22546609	22546609	G	A	UTR3	EGR3	NA	NA	NA	NA	NA
chr8	22900701	22900701	G	A	exonic	TNFRSF10B	nonsynonymous SNV	3842:exon2:c.C200T:p.A67V,TNFRSF10B:NM_147187:exc	NA	rs1047266	P
chr8	23190941	23190941	T	C	exonic	LOXL2	synonymous SNV	LOXL2:NM_002318:exon5:c.A939G:p.S313S	NA	rs1010156	NA
chr8	23536563	23536563	-	T	UTR3	NKX3-1	NA	NA	NA	NA	NA
chr8	33356074	33356074	A	G	exonic	MAK16	nonsynonymous SNV	MAK16:NM_032509:exon10:c.A830G:p.Q277R	NA	rs6468171	B
chr8	38033989	38033989	G	C	UTR5	LSM1	NA	NA	NA	rs2270376	NA
chr8	39311600	39311600	C	T	ncRNA_exonic	ADAM3A	NA	NA	NA	rs7813010	NA
chr8	40438757	40438757	T	C	exonic	ZMAT4	nonsynonymous SNV	5731:exon5:c.A373G:p.T125A,ZMAT4:NM_024645:exon	ID=COSM1179839;OCCURENCE=1(prostate)	rs17851751	B
chr8	52730987	52730987	C	A	UTR3	PCMTD1	NA	NA	NA	rs201437100	NA
chr8	75946695	75946695	T	G	UTR3	CRISPLD1	NA	NA	NA	rs13248650	NA
chr8	76476959	76476959	C	A	UTR3	HNF4G	NA	NA	NA	rs2941479	NA
chr8	76478991	76478991	G	A	UTR3	HNF4G	NA	NA	NA	rs11774375	NA
chr8	82712242	82712242	-	AA	UTR3	SNX16	NA	NA	NA	NA	NA
chr8	87666251	87666251	T	G	exonic	CNGB3	nonsynonymous SNV	CNGB3:NM_019098:exon7:c.A892C:p.T298P	NA	rs4961206	B
chr8	90914272	90914272	A	G	UTR5	OSGIN2	NA	NA	NA	rs2250670	NA
chr8	1.04E+08	1.04E+08	G	A	exonic	ODF1	nonsynonymous SNV	ODF1:NM_024410:exon2:c.G647A:p.S216N	NA	rs2916569	NA
chr8	1.21E+08	1.21E+08	C	G	exonic	TAF2	nonsynonymous SNV	TAF2:NM_003184:exon11:c.G1340C:p.S447T	ID=COSM150523;OCCURENCE=1(stomach)	rs9297605	B
chr8	1.23E+08	1.23E+08	T	A	ncRNA_UTR5	HAS2	NA	NA	NA	rs3853253	NA
chr8	1.31E+08	1.31E+08	A	G	exonic	FAM49B	synonymous SNV	23:exon8:c.T585C:p.T195T,FAM49B:NM_001256763:exor	NA	rs1051893	NA
chr8	1.34E+08	1.34E+08	G	A	exonic	TG	nonsynonymous SNV	TG:NM_003235:exon44:c.G7589A:p.R2530Q	NA	rs1133076	B
chr8	1.34E+08	1.34E+08	T	-	UTR3	ST3GAL1	NA	NA	NA	rs61689158	NA
chr8	1.42E+08	1.42E+08	A	G	exonic	SLC45A4	synonymous SNV	SLC45A4:NM_001080431:exon7:c.T2070C:p.G690G	NA	rs7017848	NA
chr8	1.42E+08	1.42E+08	T	C	ncRNA_exonic	LOC731779	NA	NA	NA	rs6987289	NA
chr8	1.45E+08	1.45E+08	CT	-	UTR3	FAM83H	NA	NA	NA	rs34283115	NA
chr8	1.45E+08	1.45E+08	G	T	UTR3	GRINA	NA	NA	NA	rs9100	NA
chr9	2029199	2029199	G	A	exonic	SMARCA2	synonymous SNV	3070:exon2:c.G177A:p.T59T,SMARCA2:NM_139045:exon	NA	rs10964471	NA

chr9	4622453	4622453	T	C	exonic	SPATA6L	nonsynonymous SNV	SPATA6L:NM_001039395:exon6:c.A553G:p.R185G	NA	rs10974657	P
chr9	12775861	12775861	-	GCGGCGG	exonic	LURAP1L	nonframeshift insertion	.:NM_203403:exon1:c.147_148insGGCGGCGGC:p.G49deli	NA	rs139315731	NA
chr9	14993419	14993419	G	T	ncRNA_exonic	LOC389705	NA	NA	NA	rs4548277	NA
chr9	17273731	17273731	A	G	exonic	CNTLN	nonsynonymous SNV	.4395:exon6:c.A850G:p.T284A,CNTLN:NM_017738:exon6	NA	rs3808795	B
chr9	19060197	19060197	A	T	exonic	HAUS6	nonsynonymous SNV	890:exon15:c.T1549A:p.S517T,HAUS6:NM_017645:exon1	NA	rs41269003	D
chr9	20346377	20346377	-	A	UTR3	MLLT3	NA	NA	NA	NA	NA
chr9	27062666	27062666	G	A	exonic	IFT74	nonsynonymous SNV	79M,IFT74:NM_001099223:exon20:c.G1735A:p.V579M,I	NA	rs138591335	P
chr9	27205021	27205021	G	A	exonic	TEK	synonymous SNV	TEK:NM_000459:exon14:c.G2322A:p.R774R	NA	rs542913	NA
chr9	33111199	33111199	C	T	UTR3	B4GALT1	NA	NA	NA	rs74995636	NA
chr9	33386465	33386465	A	G	exonic	AQP7	nonsynonymous SNV	AQP7:NM_001170:exon5:c.T343C:p.Y115H	NA	rs74668961	B
chr9	38398153	38398153	A	T	UTR3	ALDH1B1	NA	NA	NA	rs74363930	NA
chr9	41952522	41952522	T	A	ncRNA_exonic	MGC21881	NA	NA	NA	rs62539305	NA
chr9	41953020	41953020	T	C	ncRNA_exonic	MGC21881	NA	NA	NA	rs201318708	NA
chr9	41960898	41960898	G	T	ncRNA_exonic	KGFLP2	NA	NA	NA	rs201761160	NA
chr9	66501326	66501326	T	C	ncRNA_exonic	TGER4P2-CDK2AP2F	NA	NA	NA	rs28692535	NA
chr9	66501329	66501329	C	G	ncRNA_exonic	TGER4P2-CDK2AP2F	NA	NA	NA	rs201855807	NA
chr9	67281729	67281729	G	A	ncRNA_exonic	AQP7P1	NA	NA	NA	rs9696090	NA
chr9	69663721	69663721	C	G	ncRNA_exonic	LOC100133920	NA	NA	NA	rs199847383	NA
chr9	69664842	69664842	T	A	ncRNA_exonic	LOC100133920	NA	NA	NA	rs75601058	NA
chr9	70175764	70175764	A	G	UTR3	FOXD4L5	NA	NA	NA	rs201445301	NA
chr9	70175856	70175856	C	T	UTR3	FOXD4L5	NA	NA	NA	rs187159666	NA
chr9	77407636	77407636	C	T	exonic	TRPM6	synonymous SNV	09Q,TRPM6:NM_001177311:exon19:c.G2427A:p.Q809Q,	NA	rs4145894	NA
chr9	87285595	87285595	C	G	UTR5	NTRK2	NA	NA	NA	rs1187325	NA
chr9	88959938	88959938	C	T	exonic	ZCCHC6	synonymous SNV	L317L,ZCCHC6:NM_001185074:exon5:c.G951A:p.L317L,Z	NA	rs791323	NA
chr9	89763574	89763574	A	C	UTR5	C9orf170	NA	NA	NA	rs461126	NA
chr9	94985637	94985637	T	C	exonic	IARS	nonsynonymous SNV	:exon32:c.A3544G:p.K1182E,IARS:NM_013417:exon32:c.	NA	rs556155	B
chr9	1E+08	1E+08	T	C	exonic	TDRD7	nonsynonymous SNV	TDRD7:NM_014290:exon4:c.T449C:p.V150A	ID=COSM150645;OCCURENCE=1(stomach)	rs2045732	B
chr9	1.02E+08	1.02E+08	A	C	UTR5	SEC61B	NA	NA	NA	NA	NA
chr9	1.13E+08	1.13E+08	C	T	UTR3	PALM2	NA	NA	NA	rs10816907	NA
chr9	1.16E+08	1.16E+08	C	A	UTR5	ZNF883	NA	NA	NA	rs1324934	NA
chr9	1.17E+08	1.17E+08	A	G	exonic	AKNA	nonsynonymous SNV	AKNA:NM_030767:exon21:c.T3907C:p.S1303P	NA	rs2250242	B
chr9	1.17E+08	1.17E+08	A	G	exonic	DFNB31	synonymous SNV	;8G,DFNB31:NM_001173425:exon6:c.T1353C:p.G451G,DI	NA	rs4979387	NA
chr9	1.23E+08	1.23E+08	C	G	exonic	CDK5RAP2	nonsynonymous SNV	0L,CDK5RAP2:NM_001011649:exon31:c.G4618C:p.V1540	NA	rs4837768	B
chr9	1.27E+08	1.27E+08	G	A	exonic	GPR144	nonsynonymous SNV	GPR144:NM_001161808:exon10:c.G1694A:p.S565N	NA	NA	NA
chr9	1.32E+08	1.32E+08	T	C	exonic	TBC1D13	nonsynonymous SNV	TBC1D13:NM_018201:exon8:c.T569C:p.V190A	NA	rs1572912	B
chr9	1.33E+08	1.33E+08	T	G	UTR3	TOR1B	NA	NA	NA	rs1220509	NA
chr9	1.35E+08	1.35E+08	G	T	UTR3	SETX	NA	NA	NA	rs11787894	NA
chr9	1.36E+08	1.36E+08	C	T	exonic	RALGDS	synonymous SNV	108V,RALGDS:NM_001271775:exon3:c.G372A:p.V124V,R	NA	rs3761824	NA
chr9	1.36E+08	1.36E+08	C	T	exonic	ADAMTSL2	synonymous SNV	320:exon17:c.C2436T:p.R812R,ADAMTSL2:NM_014694:e	NA	rs147931149	NA
chr9	1.38E+08	1.38E+08	-	C	UTR3	OBP2A	NA	NA	NA	rs112851559	NA
chr10	1205801	1205801	C	A	ncRNA_exonic	LINC00200	NA	NA	NA	rs12763661	NA
chr10	1228330	1228330	G	T	UTR3	ADARB2	NA	NA	NA	rs1129226	NA
chr10	3172145	3172145	C	T	exonic	PFKP	synonymous SNV	:exon17:c.C1818T:p.F606F,PFKP:NM_001242339:exon19:	NA	rs1052337	NA
chr10	3208566	3208566	C	T	exonic	PITRM1	synonymous SNV	Q59Q,PITRM1:NM_001242307:exon4:c.G273A:p.Q91Q,PI	NA	rs114690446	NA
chr10	3208567	3208567	T	G	exonic	PITRM1	nonsynonymous SNV	.Q59P,PITRM1:NM_001242307:exon4:c.A272C:p.Q91P,PI	NA	rs28416720	NA
chr10	7285632	7285632	A	G	exonic	SFMBT2	synonymous SNV	39:exon9:c.T1008C:p.D336D,SFMBT2:NM_001029880:ex	NA	rs2248474	NA
chr10	7780655	7780655	G	A	exonic	ITIH2	nonsynonymous SNV	ITIH2:NM_002216:exon16:c.G2029A:p.V677M	NA	rs188916946	B
chr10	16547058	16547058	A	G	exonic	PTER	synonymous SNV	.836:exon4:c.A738G:p.Q246Q,PTER:NM_001001484:exon	NA	rs7904014	NA
chr10	17088006	17088006	T	C	exonic	CUBN	synonymous SNV	CUBN:NM_001081:exon24:c.A3417G:p.L1139L	NA	rs1801228	NA
chr10	17130199	17130199	G	A	exonic	CUBN	synonymous SNV	CUBN:NM_001081:exon15:c.C1911T:p.L637L	NA	rs41289311	NA
chr10	18242311	18242311	A	G	exonic	SLC39A12	nonsynonymous SNV	.45195:exon2:c.A106G:p.S36G,SLC39A12:NM_152725:ex	NA	rs10764176	B
chr10	21807780	21807780	T	-	UTR5	SKIDA1	NA	NA	NA	rs112940679	NA
chr10	27224492	27224492	G	C	ncRNA_exonic	LINC00202-1	NA	NA	NA	rs11015390	NA
chr10	32580205	32580205	C	T	exonic	EPC1	nonsynonymous SNV	M287I,EPC1:NM_001272019:exon6:c.G648A:p.M216I,EP	NA	rs72789797	P
chr10	33165422	33165422	G	A	UTR3	C10orf68	NA	NA	NA	rs12217414	NA
chr10	33214802	33214802	A	G	exonic	ITGB1	synonymous SNV	C261C,ITGB1:NM_002211:exon6:c.T783C:p.C261C,ITGB1	NA	rs2298141	NA
chr10	45649320	45649320	A	G	ncRNA_exonic	RSU1P2	NA	NA	NA	rs7478624	NA
chr10	50029121	50029121	C	T	exonic	WDFY4	synonymous SNV	WDFY4:NM_020945:exon34:c.C5724T:p.H1908H	NA	rs79406124	NA
chr10	65225899	65225899	-	GGCGGC	ncRNA_exonic	JMJD1C-AS1	NA	NA	NA	rs3841602	NA
chr10	71899708	71899708	A	G	exonic	TYSND1	nonsynonymous SNV	TYSND1:NM_173555:exon4:c.T1673C:p.L558P	NA	NA	B
chr10	72536921	72536921	A	G	exonic	TBATA	synonymous SNV	TBATA:NM_152710:exon7:c.T678C:p.D226D	NA	rs151019808	NA
chr10	73767481	73767481	A	C	exonic	CHST3	nonsynonymous SNV	CHST3:NM_004273:exon3:c.A692C:p.D231A	NA	NA	B

chr10	81114813	81114813	C	G	UTR3	PPIF	NA	NA	NA	rs8837	NA
chr10	82126808	82126808	C	G	UTR3	DYDC2	NA	NA	NA	rs1047952	NA
chr10	86018493	86018493	-	A	UTR3	RGR	NA	NA	NA	rs34014972	NA
chr10	89127510	89127523	GGACTGGC	-	ncRNA_exonic	NUTM2D	NA	NA	NA	NA	NA
chr10	90585819	90585819	G	A	exonic	ANKRD22	synonymous SNV	ANKRD22:NM_144590:exon4:c.C375T:p.G125G	NA	rs7893917	NA
chr10	1.02E+08	1.02E+08	C	T	exonic	PKD2L1	nonsynonymous SNV	3837:exon6:c.G1036A:p.V346I,PKD2L1:NM_016112:exon	NA	rs2278842	P
chr10	1.04E+08	1.04E+08	C	A	UTR3	TMEM180	NA	NA	NA	rs10883724	NA
chr10	1.05E+08	1.05E+08	T	C	UTR3	CALHM1	NA	NA	NA	rs192524959	NA
chr10	1.14E+08	1.14E+08	A	G	ncRNA_exonic	GUCY2GP	NA	NA	NA	rs1887136	NA
chr10	1.16E+08	1.16E+08	C	G	UTR5	ABLIM1	NA	NA	NA	rs141394498	NA
chr10	1.17E+08	1.17E+08	T	C	exonic	FAM160B1	synonymous SNV	051:exon14:c.T1893C:p.F631F,FAM160B1:NM_020940:e	NA	rs3180654	NA
chr10	1.18E+08	1.18E+08	-	TT	UTR3	GFRA1	NA	NA	NA	NA	NA
chr10	1.18E+08	1.18E+08	-	G	exonic	PNLIPRP2	unknown	UNKNOWN	NA	NA	NA
chr10	1.18E+08	1.18E+08	A	G	UTR3	HSPA12A	NA	NA	NA	rs7084566	NA
chr10	1.19E+08	1.19E+08	T	C	ncRNA_exonic	EMX2OS	NA	NA	NA	rs2768325	NA
chr10	1.19E+08	1.19E+08	C	G	ncRNA_exonic	EMX2OS	NA	NA	NA	rs2768330	NA
chr10	1.2E+08	1.2E+08	G	-	UTR5	RAB11FIP2	NA	NA	NA	rs67350544	NA
chr10	1.25E+08	1.25E+08	G	A	UTR3	ACADSB	NA	NA	NA	rs1140593	NA
chr10	1.27E+08	1.27E+08	G	C	exonic	CTBP2	nonsynonymous SNV	CTBP2:NM_022802:exon1:c.C1615G:p.Q539E	NA	rs2946994	P
chr10	1.27E+08	1.27E+08	A	G	exonic	CTBP2	nonsynonymous SNV	CTBP2:NM_022802:exon1:c.T1363C:p.Y455H	NA	rs3012075	D
chr10	1.34E+08	1.34E+08	C	T	UTR3	LRRC27	NA	NA	NA	rs72863837	NA
chr10	1.34E+08	1.34E+08	A	G	UTR3	LRRC27	NA	NA	NA	rs72863838	NA
chr10	1.35E+08	1.35E+08	T	C	exonic	SYCE1	nonsynonymous SNV	SYCE1:NM_001143764:exon13:c.A962G:p.H321R	ID=COSM147095;OCCURENCE=1(stomach)	rs3737031	NA
chr10	1.35E+08	1.35E+08	A	T	exonic	FRG2B	nonsynonymous SNV	FRG2B:NM_001080998:exon1:c.T44A:p.I15N	NA	rs200661929	NA
chr11	128625	128625	A	G	ncRNA_exonic	LOC100133161	NA	NA	NA	NA	NA
chr11	237087	237087	A	G	exonic	PSMD13	nonsynonymous SNV	02817:exon1:c.A38G:p.N13S,PSMD13:NM_175932:exon1	NA	rs1045288	B
chr11	284257	284257	G	A	exonic	NLRP6	synonymous SNV	700:exon6:c.G2226A:p.A742A,NLRP6:NM_138329:exon6	NA	rs11246050	NA
chr11	611919	611919	C	G	UTR3	PHRF1	NA	NA	NA	rs702966	NA
chr11	1016609	1016609	C	G	exonic	MUC6	nonsynonymous SNV	MUC6:NM_005961:exon31:c.G6192C:p.M2064I	NA	rs73403291	NA
chr11	1017317	1017317	G	T	exonic	MUC6	synonymous SNV	MUC6:NM_005961:exon31:c.C5484A:p.T1828T	NA	rs33943903	NA
chr11	1017337	1017337	T	C	exonic	MUC6	nonsynonymous SNV	MUC6:NM_005961:exon31:c.A5464G:p.T1822A	NA	rs76686156	NA
chr11	1017338	1017338	C	A	exonic	MUC6	nonsynonymous SNV	MUC6:NM_005961:exon31:c.G5463T:p.Q1821H	NA	rs78943453	NA
chr11	1018168	1018170	TCG	-	exonic	MUC6	nonframeshift deletion	IUC6:NM_005961:exon31:c.4631_4633del:p.1544_1545d	NA	rs111749447	NA
chr11	1018300	1018300	G	T	exonic	MUC6	nonsynonymous SNV	MUC6:NM_005961:exon31:c.C4501A:p.P1501T	NA	rs113744996	NA
chr11	1018304	1018304	G	C	exonic	MUC6	synonymous SNV	MUC6:NM_005961:exon31:c.C4497G:p.A1499A	NA	rs113835477	NA
chr11	1081757	1081757	G	C	exonic	MUC2	nonsynonymous SNV	MUC2:NM_002457:exon13:c.G1685C:p.S562T	NA	rs57737240	NA
chr11	2672432	2672432	-	A	ncRNA_exonic	KCNQ1OT1	NA	NA	NA	rs34514246	NA
chr11	2699252	2699252	-	T	ncRNA_exonic	KCNQ1OT1	NA	NA	NA	NA	NA
chr11	2924850	2924850	-	C	UTR5	SLC22A18AS	NA	NA	NA	NA	NA
chr11	6411935	6411935	T	C	exonic	SMPD1	nonsynonymous SNV	543:exon1:c.T107C:p.V36A,SMPD1:NM_001007593:exon	NA	rs1050228	NA
chr11	11985134	11985139	TTTACT	-	UTR3	DKK3	NA	NA	NA	rs142843448	NA
chr11	12316389	12316389	A	C	exonic	MICALCL	nonsynonymous SNV	MICALCL:NM_032867:exon3:c.A1411C:p.T471P	NA	rs3812754	B
chr11	17519742	17519742	C	G	exonic	USH1C	nonsynonymous SNV	9:exon19:c.G1557C:p.E519D,USH1C:NM_153676:exon24	NA	rs1064074	P
chr11	26690702	26690702	C	T	UTR3	SLC5A12	NA	NA	NA	rs76831336	NA
chr11	34642668	34642668	A	G	UTR5	EHF	NA	NA	NA	rs286925	NA
chr11	34642728	34642728	A	T	UTR5	EHF	NA	NA	NA	rs286924	NA
chr11	35201842	35201842	C	T	exonic	CD44	synonymous SNV	35H,CD44:NM_001001391:exon3:c.C255T:p.H85H,CD44:N	NA	rs1071695	NA
chr11	43940644	43940644	G	T	exonic	ALKBH3	synonymous SNV	ALKBH3:NM_139178:exon9:c.G726T:p.G242G	NA	rs1048928	NA
chr11	46342943	46342943	A	-	UTR3	CREB3L1	NA	NA	NA	NA	NA
chr11	48346661	48346669	TTGCTGATC	-	exonic	OR4C3	nonframeshift deletion	OR4C3:NM_001004702:exon1:c.169_177del:p.57_59del	ID=COSM1163639;OCCURENCE=2(pancreas)	rs138704181	NA
chr11	62294717	62294717	A	T	exonic	AHNAK	nonsynonymous SNV	AHNAK:NM_001620:exon5:c.T7172A:p.L2391H	NA	NA	P
chr11	62378801	62378801	T	C	exonic	EML3	synonymous SNV	EML3:NM_153265:exon3:c.A210G:p.P70P	NA	rs11553576	NA
chr11	62434173	62434173	G	A	exonic	METTL12	nonsynonymous SNV	METTL12:NM_001043229:exon3:c.G373A:p.G125S	NA	rs11231181	B
chr11	64138805	64138805	T	C	exonic	RPS6KA4	synonymous SNV	044:exon17:c.T2154C:p.N718N,RPS6KA4:NM_003942:exo	NA	rs11542299	NA
chr11	64807447	64807447	A	C	ncRNA_exonic	ARL2-SNX15	NA	NA	NA	rs664118	NA
chr11	67415054	67415054	C	T	UTR5	ACY3	NA	NA	NA	rs2514036	NA
chr11	71708250	71708253	GTGT	-	UTR3	RNF121	NA	NA	NA	NA	NA
chr11	72385393	72385393	A	C	UTR5	PDE2A	NA	NA	NA	NA	NA
chr11	73670645	73670645	T	C	exonic	DNAJB13	synonymous SNV	DNAJB13:NM_153614:exon3:c.T279C:p.H93H	NA	rs653263	NA
chr11	89059054	89059055	TA	-	UTR3	NOX4	NA	NA	NA	NA	NA
chr11	94150790	94150790	T	C	UTR3	MRE11A	NA	NA	NA	rs2155209	NA
chr11	1.06E+08	1.06E+08	-	A	UTR3	GRIA4	NA	NA	NA	NA	NA

chr11	1.08E+08	1.08E+08	A	G	UTR3	SLC35F2	NA	NA	NA	rs4754259	NA
chr11	1.1E+08	1.1E+08	AA	-	UTR3	ARHGAP20	NA	NA	NA	rs200762479	NA
chr11	1.11E+08	1.11E+08	-	AGGA	UTR3	POU2AF1	NA	NA	NA	NA	NA
chr11	1.12E+08	1.12E+08	G	-	splicing	.61-1C>-,NM_0010[illegible]	NA	NA	NA	rs10708475	NA
chr11	1.13E+08	1.13E+08	-	G	UTR5	NCAM1	NA	NA	NA	rs145493999	NA
chr11	1.17E+08	1.17E+08	G	A	exonic	PCSK7	synonymous SNV	PCSK7:NM_004716:exon17:c.C2196T:p.D732D	NA	rs139944026	NA
chr11	1.17E+08	1.17E+08	C	G	exonic	BACE1	synonymous SNV	012104:exon5:c.G786C:p.V262V,BACE1:NM_138971:exor	NA	rs638405	NA
chr11	1.17E+08	1.17E+08	G	T	exonic	DSCAML1	nonsynonymous SNV	DSCAML1:NM_020693:exon4:c.C694A:p.H232N	ID=COSM147330;OCCURENCE=1(stomach)	rs3741280	NA
chr11	1.18E+08	1.18E+08	CACA	-	UTR3	FXYD6	NA	NA	NA	NA	NA
chr11	1.19E+08	1.19E+08	G	C	UTR3	DDX6	NA	NA	NA	rs487728	NA
chr11	1.19E+08	1.19E+08	-	T	UTR3	DDX6	NA	NA	NA	rs139078958	NA
chr11	1.25E+08	1.25E+08	-	T	UTR3	MSANTD2	NA	NA	NA	rs137996267	NA
chr11	1.25E+08	1.25E+08	G	A	exonic	ROBO4	synonymous SNV	ROBO4:NM_019055:exon2:c.C103T:p.L35L	NA	rs73016370	NA
chr11	1.25E+08	1.25E+08	G	A	UTR3	SLC37A2	NA	NA	NA	NA	NA
chr11	1.25E+08	1.25E+08	C	T	exonic	STT3A	synonymous SNV	STT3A:NM_152713:exon16:c.C1911T:p.C637C	NA	rs17140116	NA
chr11	1.25E+08	1.25E+08	C	T	UTR5	CHEK1	NA	NA	NA	rs558351	NA
chr11	1.26E+08	1.26E+08	C	T	exonic	DCPS	synonymous SNV	DCPS:NM_014026:exon1:c.C63T:p.H21H	NA	rs3740915	NA
chr11	1.3E+08	1.3E+08	-	T	UTR3	ZBTB44	NA	NA	NA	NA	NA
chr11	1.3E+08	1.3E+08	G	A	UTR3	ZBTB44	NA	NA	NA	rs184424056	NA
chr11	1.3E+08	1.3E+08	A	G	UTR3	ZBTB44	NA	NA	NA	rs2297070	NA
chr11	1.34E+08	1.34E+08	G	A	intronic	IGSF9B	NA	NA	NA	rs329677	NA
chr11	1.34E+08	1.34E+08	G	A	UTR3	JAM3	NA	NA	NA	rs597320	NA
chr12	1022452	1022452	C	T	UTR3	RAD52	NA	NA	NA	rs1051669	NA
chr12	4414167	4414167	T	-	UTR3	CCND2	NA	NA	NA	rs61152953	NA
chr12	4919197	4919197	T	C	UTR5	KCNA6	NA	NA	NA	NA	NA
chr12	4919248	4919248	A	G	exonic	KCNA6	nonsynonymous SNV	KCNA6:NM_002235:exon1:c.A41G:p.E14G	NA	NA	P
chr12	7649484	7649484	T	C	exonic	CD163	nonsynonymous SNV	!44:exon5:c.A1024G:p.I342V,CD163:NM_203416:exon5:c	NA	rs4883263	NA
chr12	7945559	7945559	T	C	exonic	NANOG	synonymous SNV	NANOG:NM_024865:exon2:c.T165C:p.P55P	NA	rs4294629	NA
chr12	7945640	7945640	G	T	exonic	NANOG	nonsynonymous SNV	NANOG:NM_024865:exon2:c.G246T:p.K82N	NA	rs2889551	B
chr12	7945670	7945670	G	A	exonic	NANOG	synonymous SNV	NANOG:NM_024865:exon2:c.G276A:p.P92P	NA	rs4354764	NA
chr12	7945757	7945757	C	T	exonic	NANOG	synonymous SNV	NANOG:NM_024865:exon2:c.C363T:p.S121S	NA	rs4438116	NA
chr12	7948584	7948584	C	A	UTR3	NANOG	NA	NA	NA	rs35212015	NA
chr12	8353486	8353486	C	G	ncRNA_exonic	FAM66C	NA	NA	NA	rs200786680	NA
chr12	10468648	10468648	T	C	UTR3	KLRD1	NA	NA	NA	rs7966660	NA
chr12	10560957	10560957	T	C	exonic	KLRC4	nonsynonymous SNV	KLRC4:NM_013431:exon3:c.A311G:p.N104S	NA	rs2617170	NA
chr12	11420471	11420471	C	G	exonic	PRB3	nonsynonymous SNV	PRB3:NM_006249:exon3:c.G712C:p.G238R	NA	NA	NA
chr12	12046813	12046813	A	G	ncRNA_UTR3	ETV6	NA	NA	NA	rs2710275	NA
chr12	12943043	12943045	TTC	-	UTR3	APOLD1	NA	NA	NA	rs146851735	NA
chr12	18435399	18435401	CCC	-	exonic	PIK3C2G	nonframeshift deletion	PIK3C2G:NM_004570:exon2:c.384_386del:p.128_129del	NA	rs55845540	NA
chr12	21329738	21329738	A	G	exonic	SLCO1B1	nonsynonymous SNV	SLCO1B1:NM_006446:exon5:c.A388G:p.N130D	NA	rs2306283	B
chr12	25146594	25146594	A	T	UTR3	C12orf77	NA	NA	NA	rs28561442	NA
chr12	45307633	45307633	T	C	UTR5	NELL2	NA	NA	NA	rs374144	NA
chr12	49298059	49298059	G	A	UTR5	CCDC65	NA	NA	NA	rs3809147	NA
chr12	49726255	49726255	-	G	UTR3	C1QL4	NA	NA	NA	rs112341833	NA
chr12	50747134	50747134	T	G	exonic	FAM186A	nonsynonymous SNV	FAM186A:NM_001145475:exon4:c.A3481C:p.T1161P	NA	NA	NA
chr12	51723499	51723499	T	C	exonic	CELA1	nonsynonymous SNV	CELA1:NM_001971:exon7:c.A728G:p.Q243R	NA	rs17860364	B
chr12	52282060	52282060	C	T	exonic	ANKRD33	synonymous SNV	ANKRD33:NM_182608:exon1:c.C90T:p.R30R	NA	rs697634	NA
chr12	52994955	52994955	G	A	exonic	KRT72	synonymous SNV	p.P94P,KRT72:NM_001146226:exon1:c.C282T:p.P94P,KRT	NA	rs61747192	NA
chr12	53045626	53045626	T	C	exonic	KRT2	nonsynonymous SNV	KRT2:NM_000423:exon1:c.A301G:p.S101G	NA	rs2634041	NA
chr12	53070145	53070145	G	A	exonic	KRT1	synonymous SNV	KRT1:NM_006121:exon7:c.C1389T:p.R463R	NA	rs936958	NA
chr12	53810134	53810136	TTT	-	UTR3	SP1	NA	NA	NA	rs147945503	NA
chr12	54475448	54475448	G	C	ncRNA_exonic	LOC100240735	NA	NA	NA	rs4016762	NA
chr12	54905815	54905815	C	G	exonic	NCKAP1L	synonymous SNV	84976:exon9:c.C717G:p.L239L,NCKAP1L:NM_005337:exo	NA	rs2458409	NA
chr12	54973122	54973123	AT	-	UTR3	PPP1R1A	NA	NA	NA	rs67431222	NA
chr12	63041689	63041689	A	-	UTR3	PPM1H	NA	NA	NA	NA	NA
chr12	64538324	64538324	G	T	UTR3	SRGAP1	NA	NA	NA	rs789736	NA
chr12	71094955	71094955	C	T	exonic	PTPRR	nonsynonymous SNV	016:exon4:c.G538A:p.V180I,PTPRR:NM_001207015:exon	NA	rs35387004	B
chr12	76747082	76747082	T	-	UTR3	OSBPL8	NA	NA	NA	rs34398462	NA
chr12	78530979	78530979	G	A	exonic	NAV3	synonymous SNV	NAV3:NM_014903:exon19:c.G4464A:p.Q1488Q	NA	rs1852464	NA
chr12	93196422	93196422	T	G	exonic	EEA1	nonsynonymous SNV	EEA1:NM_003566:exon19:c.A2428C:p.K810Q	NA	rs10745623	B
chr12	93963873	93963873	C	T	ncRNA_UTR5	SOCS2	NA	NA	NA	rs2200160	NA
chr12	1.01E+08	1.01E+08	TTTTTTTTT	-	UTR3	SLC17A8	NA	NA	NA	rs71091442	NA

chr12	1.01E+08	1.01E+08	G	A	exonic	ANO4	nonsynonymous SNV	ANO4:NM_178826:exon6:c.G455A:p.R152K	NA	NA	B
chr12	1.03E+08	1.03E+08	G	A	exonic	PARPBP	synonymous SNV	PARPBP:NM_017915:exon2:c.G138A:p.A46A	NA	rs2036771	NA
chr12	1.03E+08	1.03E+08	-	TG	UTR3	IGF1	NA	NA	NA	rs34621369	NA
chr12	1.04E+08	1.04E+08	G	A	exonic	STAB2	synonymous SNV	STAB2:NM_017564:exon6:c.G558A:p.A186A	NA	rs147053330	NA
chr12	1.04E+08	1.04E+08	C	T	exonic	STAB2	synonymous SNV	STAB2:NM_017564:exon31:c.C3339T:p.N1113N	ID=COSM147590;OCCURENCE=1(stomach)	rs703651	NA
chr12	1.04E+08	1.04E+08	A	G	UTR3	NT5DC3	NA	NA	NA	rs10735389	NA
chr12	1.04E+08	1.04E+08	C	T	ncRNA_exonic	GNN	NA	NA	NA	rs11612613	NA
chr12	1.06E+08	1.06E+08	-	ATGAAG	UTR3	NUAK1	NA	NA	NA	rs11283073	NA
chr12	1.07E+08	1.07E+08	A	G	exonic	CRY1	synonymous SNV	CRY1:NM_004075:exon5:c.T636C:p.G212G	NA	rs8192440	NA
chr12	1.14E+08	1.14E+08	G	C	exonic	RBM19	synonymous SNV	506T,RBM19:NM_001146699:exon15:c.C1818G:p.T606T,I	NA	rs2290790	NA
chr12	1.19E+08	1.19E+08	C	T	exonic	TAOK3	nonsynonymous SNV	TAOK3:NM_016281:exon4:c.G140A:p.S47N	NA	rs428073	B
chr12	1.2E+08	1.2E+08	A	-	UTR3	SRRM4	NA	NA	NA	rs11305482	NA
chr12	1.23E+08	1.23E+08	A	G	UTR3	HCAR1	NA	NA	NA	rs7313367	NA
chr12	1.29E+08	1.29E+08	A	G	UTR3	TMEM132C	NA	NA	NA	NA	NA
chr12	1.31E+08	1.31E+08	G	T	UTR5	GPR133	NA	NA	NA	rs56090874	NA
chr13	25356053	25356053	T	C	exonic	RNF17	synonymous SNV	84993:exon6:c.T582C:p.F194F,RNF17:NM_031277:exon6	NA	rs9707144	NA
chr13	26125490	26125490	G	A	exonic	ATP8A2	synonymous SNV	ATP8A2:NM_016529:exon11:c.G906A:p.A302A	NA	rs9581388	NA
chr13	28874659	28874666	CACACACA	-	UTR3	FLT1	NA	NA	NA	rs200186804	NA
chr13	32890572	32890572	G	A	UTR5	BRCA2	NA	NA	NA	rs1799943	NA
chr13	36744800	36744800	G	A	exonic	DC169-SOHLH2,SOH	synonymous SNV	n10:c.C1125T:p.Y375Y,CCDC169-SOHLH2:NM_00119891C	ID=COSM147666;OCCURENCE=1(stomach)	rs2296967	NA
chr13	36876372	36876375	TATC	-	UTR3	SPG20	NA	NA	NA	rs150662490	NA
chr13	41133064	41133064	-	T	UTR3	FOXO1	NA	NA	NA	NA	NA
chr13	41385658	41385658	C	A	ncRNA_UTR3	SLC25A15	NA	NA	NA	rs41515050	NA
chr13	46537757	46537757	G	A	UTR3	ZC3H13	NA	NA	NA	rs17350833	NA
chr13	50102992	50102992	T	C	UTR3	PHF11	NA	NA	NA	rs3033	NA
chr13	50466780	50466780	C	T	ncRNA_exonic	CTAGE10P	NA	NA	NA	rs189224500	NA
chr13	60240491	60240491	C	A	UTR3	DIAPH3	NA	NA	NA	rs200390524	NA
chr13	1.04E+08	1.04E+08	-	T	ncRNA_exonic	METTL21EP	NA	NA	NA	NA	NA
chr13	1.11E+08	1.11E+08	G	A	exonic	COL4A2	nonsynonymous SNV	COL4A2:NM_001846:exon22:c.G1550A:p.R517K	NA	rs7990383	B
chr13	1.14E+08	1.14E+08	ACAC	-	UTR3	ATP11A	NA	NA	NA	rs72007310	NA
chr13	1.14E+08	1.14E+08	AT	-	UTR3	ATP11A	NA	NA	NA	rs3832908	NA
chr13	1.14E+08	1.14E+08	C	A	UTR3	DCUN1D2	NA	NA	NA	rs9577553	NA
chr14	20811332	20811332	C	T	ncRNA_exonic	RPPH1	NA	NA	NA	rs3093872	NA
chr14	20811476	20811476	-	A	ncRNA_exonic	RPPH1	NA	NA	NA	NA	NA
chr14	24409799	24409799	A	G	ncRNA_exonic	DHRS4-AS1	NA	NA	NA	rs113551151	NA
chr14	24410262	24410262	C	G	ncRNA_exonic	DHRS4-AS1	NA	NA	NA	rs58941536	NA
chr14	50448984	50448984	A	C	UTR3	C14orf182	NA	NA	NA	rs7161196	NA
chr14	55533924	55533925	TT	-	UTR3	MAPK1IP1L	NA	NA	NA	NA	NA
chr14	61112736	61112736	T	-	UTR3	SIX1	NA	NA	NA	rs112733948	NA
chr14	61177729	61177729	-	TATATA	UTR3	SIX4	NA	NA	NA	rs142920801	NA
chr14	64937308	64937317	AAAAAAAA	-	UTR3	AKAP5	NA	NA	NA	NA	NA
chr14	69352230	69352230	G	A	exonic	ACTN1	synonymous SNV	L,ACTN1:NM_001130004:exon12:c.C1297T:p.L433L,ACTN	NA	rs15993	NA
chr14	73459917	73459917	C	T	exonic	ZFYVE1	synonymous SNV	ZFYVE1:NM_021260:exon4:c.G1137A:p.E379E	NA	rs2333016	NA
chr14	75388803	75388803	G	A	UTR5	RPS6KL1	NA	NA	NA	rs7158047	NA
chr14	77491190	77491190	-	AG	UTR3	IRF2BPL	NA	NA	NA	rs200696373	NA
chr14	77599140	77599140	-	TCTCTCTCT(	UTR3	ZDHHC22	NA	NA	NA	rs142042377	NA
chr14	88647759	88647759	C	T	UTR3	KCNK10	NA	NA	NA	rs201163029	NA
chr14	92582472	92582472	G	A	UTR3	NDUFB1	NA	NA	NA	rs13350	NA
chr14	94912896	94912896	T	G	exonic	SERPINA11	nonsynonymous SNV	SERPINA11:NM_001080451:exon3:c.A689C:p.E230A	NA	rs57740714	D
chr14	94933709	94933709	C	T	exonic	SERPINA9	synonymous SNV	39:exon3:c.G693A:p.E231E,SERPINA9:NM_001042518:exc	NA	rs6575433	NA
chr14	95107973	95107973	C	T	ncRNA_exonic	SERPINA13P	NA	NA	NA	rs4905226	NA
chr14	96871104	96871104	G	A	exonic	AK7	nonsynonymous SNV	AK7:NM_152327:exon3:c.G305A:p.R102Q	NA	rs2275554	P
chr14	1.01E+08	1.01E+08	A	T	UTR5	WARS	NA	NA	NA	rs7143006	NA
chr14	1.05E+08	1.05E+08	C	T	UTR5	AKT1	NA	NA	NA	rs10138227	NA
chr14	1.05E+08	1.05E+08	C	T	ncRNA_exonic	LINC00638	NA	NA	NA	rs12894160	NA
chr14	1.05E+08	1.05E+08	C	G	exonic	AHNAK2	nonsynonymous SNV	AHNAK2:NM_138420:exon7:c.G9625C:p.V3209L	NA	rs201181175	NA
chr14	1.07E+08	1.07E+08	G	C	ncRNA_exonic	LINC00221	NA	NA	NA	rs10873578	NA
chr14	1.07E+08	1.07E+08	C	G	ncRNA_exonic	LINC00221	NA	NA	ID=COSN393151;OCCURENCE=1(lung)	rs11160980	NA
chr14	1.07E+08	1.07E+08	T	G	ncRNA_exonic	LINC00221	NA	NA	NA	rs11160981	NA
chr15	21134563	21134563	A	G	ncRNA_exonic	NF1P2	NA	NA	NA	rs145897182	NA
chr15	22703218	22703218	A	G	ncRNA_exonic	GOLGA8DP	NA	NA	NA	rs1822555	NA
chr15	23005380	23005380	-	C	UTR3	NIPA2	NA	NA	NA	rs35420735	NA

chr15	23448270	23448270	G	T	ncRNA_exonic	GOLGA8EP	NA	NA	NA	rs200235850	NA
chr15	24813154	24813154	T	C	ncRNA_exonic	PWRN1	NA	NA	NA	rs28574764	NA
chr15	28096538	28096538	A	G	exonic	OCA2	synonymous SNV	OCA2:NM_000275:exon22:c.T2328C:p.A776A	NA	rs1800419	NA
chr15	41024303	41024303	T	C	UTR3	RAD51	NA	NA	NA	rs11855560	NA
chr15	41099899	41099899	-	GGGGC	exonic	ZFYVE19	frameshift insertion	.112_113insGGGGC:p.W38fs,ZFYVE19:NM_001258420:ex	NA	rs142730574	NA
chr15	42159290	42159290	C	T	exonic	SPTBN5	nonsynonymous SNV	SPTBN5:NM_016642:exon36:c.G6347A:p.R2116Q	NA	rs2290553	NA
chr15	42834962	42834962	C	T	UTR3	LRRC57	NA	NA	NA	rs75705010	NA
chr15	52611451	52611451	A	G	exonic	MYO5A	synonymous SNV	)5:exon37:c.T4884C:p.D1628D,MYO5A:NM_000259:exon	NA	rs2290332	NA
chr15	60714879	60714879	-	A	UTR3	NARG2	NA	NA	NA	NA	NA
chr15	62146078	62146078	A	-	UTR3	VPS13C	NA	NA	NA	rs5813123	NA
chr15	71276491	71276491	G	A	exonic	LRRC49	nonsynonymous SNV	360Q,LRRC49:NM_017691:exon11:c.G1064A:p.R355Q,LRF	NA	NA	B
chr15	75581791	75581791	G	A	exonic	GOLGA6D	synonymous SNV	GOLGA6D:NM_001145224:exon8:c.G618A:p.K206K	NA	NA	NA
chr15	78792881	78792883	AAA	-	UTR3	IREB2	NA	NA	NA	NA	NA
chr15	81585177	81585177	A	C	exonic	IL16	synonymous SNV	:exon11:c.A1701C:p.P567P,IL16:NM_001172128:exon12:c	NA	NA	NA
chr15	1E+08	1E+08	C	T	ncRNA_exonic	DNM1P46	NA	NA	NA	NA	NA
chr15	1E+08	1E+08	A	G	ncRNA_exonic	DNM1P46	NA	NA	NA	rs2458579	NA
chr15	1.03E+08	1.03E+08	C	T	ncRNA_exonic	WASH3P	NA	NA	NA	rs1045862	NA
chr16	332992	332992	T	C	UTR3	ARHGDIG	NA	NA	NA	rs443338	NA
chr16	781633	781633	G	A	exonic	NARFL	synonymous SNV	NARFL:NM_022493:exon9:c.C966T:p.H322H	ID=COSM1178439;OCCURENCE=1(prostate)	rs3829492	NA
chr16	903965	903965	T	G	UTR3	LMF1	NA	NA	NA	NA	NA
chr16	1139446	1139446	G	T	UTR3	C1QTNF8	NA	NA	NA	rs183188	NA
chr16	1291454	1291454	G	A	exonic	TPSAB1	nonsynonymous SNV	TPSAB1:NM_003294:exon4:c.G253A:p.A85T	NA	rs1141968	B
chr16	1448690	1448690	A	C	UTR3	UNKL	NA	NA	NA	NA	NA
chr16	2031405	2031405	T	C	UTR5	NOXO1	NA	NA	NA	rs56268858	NA
chr16	4751045	4751045	C	T	exonic	ANKS3	nonsynonymous SNV	2929:exon8:c.G889A:p.A297T,ANKS3:NM_133450:exon11	ID=COSM148013;OCCURENCE=1(stomach)	rs863980	B
chr16	10524657	10524659	GAC	-	exonic	ATF7IP2	nonframeshift deletion	3:c.180_182del:p.60_61del,ATF7IP2:NM_001256160:exon	NA	rs56252625	NA
chr16	12061912	12061912	-	T	UTR3	TNFRSF17	NA	NA	NA	NA	NA
chr16	14529754	14529755	CA	-	UTR3	PARN	NA	NA	NA	NA	NA
chr16	18839362	18839362	T	C	exonic	SMG1	synonymous SNV	SMG1:NM_015092:exon55:c.A9732G:p.A3244A	NA	rs12445870	NA
chr16	20748331	20748331	C	A	exonic	THUMPD1	nonsynonymous SNV	THUMPD1:NM_017736:exon4:c.G933T:p.E311D	NA	rs11074471	NA
chr16	21078693	21078693	T	G	exonic	DNAH3	synonymous SNV	DNAH3:NM_017539:exon24:c.A3429C:p.A1143A	NA	rs861424	NA
chr16	23598584	23598584	C	T	exonic	NDUFAB1	synonymous SNV	NDUFAB1:NM_005003:exon2:c.G225A:p.T75T	NA	rs139679834	NA
chr16	24836091	24836091	A	-	UTR3	TNRC6A	NA	NA	NA	NA	NA
chr16	46693621	46693621	-	A	UTR3	VPS35	NA	NA	NA	rs34926621	NA
chr16	46773999	46773999	C	A	exonic	MYLK3	nonsynonymous SNV	MYLK3:NM_182493:exon2:c.G538T:p.V180L	ID=COSM148070,COSM148071;OCCURENCE=1(stomach)	rs28407821	D
chr16	48258198	48258198	C	T	exonic	ABCC11	nonsynonymous SNV	180R,ABCC11:NM_032583:exon5:c.G538A:p.G180R,ABCC	NA	rs17822931	D
chr16	50264211	50264211	-	A	UTR3	PAPD5	NA	NA	NA	rs34413257	NA
chr16	55794514	55794514	C	T	ncRNA_exonic	CES1P1	NA	NA	NA	rs7199449	NA
chr16	56510842	56510842	G	C	UTR3	OGFOD1	NA	NA	NA	rs111625702	NA
chr16	68857441	68857441	T	C	exonic	CDH1	synonymous SNV	CDH1:NM_004360:exon13:c.T2076C:p.A692A	NA	rs1801552	NA
chr16	69385641	69385641	A	G	exonic	TMED6	nonsynonymous SNV	TMED6:NM_144676:exon1:c.T16C:p.F6L	NA	rs76116020	B
chr16	70164334	70164334	A	G	exonic	PDPR	nonsynonymous SNV	PDPR:NM_017990:exon7:c.A616G:p.I206V	ID=COSM148098;OCCURENCE=1(stomach)	rs10852462	NA
chr16	70506907	70506907	T	C	exonic	FUK	synonymous SNV	FUK:NM_145059:exon15:c.T1428C:p.P476P	ID=COSM1128894;OCCURENCE=1(prostate)	rs7192865	NA
chr16	70569215	70569215	A	T	exonic	SF3B3	synonymous SNV	SF3B3:NM_012426:exon6:c.A717T:p.P239P	NA	rs33910368	NA
chr16	74452071	74452071	G	A	exonic	CLEC18B	synonymous SNV	CLEC18B:NM_001011880:exon3:c.C342T:p.P114P	NA	rs62056016	NA
chr16	74920191	74920191	G	A	exonic	WDR59	synonymous SNV	WDR59:NM_030581:exon24:c.C2523T:p.R841R	NA	rs14308	NA
chr16	75327916	75327916	A	G	exonic	CFDP1	synonymous SNV	CFDP1:NM_006324:exon7:c.T834C:p.L278L	NA	rs6624	NA
chr16	84522897	84522897	G	C	exonic	TLDC1	nonsynonymous SNV	TLDC1:NM_020947:exon4:c.C516G:p.D172E	ID=COSM1128850;OCCURENCE=1(prostate)	rs436278	B
chr16	84779168	84779168	G	A	exonic	USP10	nonsynonymous SNV	3:exon4:c.G1081A:p.E361K,USP10:NM_001272075:exon5	NA	NA	NA
chr16	85009567	85009567	T	C	UTR3	ZDHHC7	NA	NA	NA	NA	NA
chr16	89334787	89334787	C	T	UTR3	ANKRD11	NA	NA	NA	rs9635535	NA
chr17	1606883	1606883	T	G	UTR3	TLCD2	NA	NA	NA	rs8068949	NA
chr17	2310332	2310332	G	A	ncRNA_exonic	LOC284009	NA	NA	NA	rs74415860	NA
chr17	3352294	3352294	A	G	exonic	SPATA22	nonsynonymous SNV	.170697:exon6:c.T479C:p.I160T,SPATA22:NM_001170698:	NA	rs1488689	B
chr17	3352331	3352331	C	T	exonic	SPATA22	nonsynonymous SNV	)697:exon6:c.G442A:p.V148M,SPATA22:NM_001170698:	NA	rs1488690	B
chr17	3564711	3564711	-	G	UTR3	CTNS	NA	NA	NA	NA	NA
chr17	5288983	5288983	T	C	UTR3	RABEP1	NA	NA	NA	rs7426	NA
chr17	6556194	6556194	-	AGAAG	UTR3	C17orf100	NA	NA	NA	rs144808411	NA
chr17	7482019	7482019	A	-	ncRNA_exonic	SENP3-EIF4A1	NA	NA	NA	NA	NA
chr17	7838406	7838406	T	C	exonic	CNTROB	synonymous SNV	57144:exon4:c.T537C:p.N179N,CNTROB:NM_053051:exor	NA	NA	NA
chr17	8064081	8064081	-	G	UTR3	VAMP2	NA	NA	NA	NA	NA
chr17	8633310	8633310	C	T	UTR3	CCDC42	NA	NA	NA	rs1971773	NA

chr17	16842912	16842912	A	G	exonic	TNFRSF13B	synonymous SNV	TNFRSF13B:NM_012452:exon5:c.T831C:p.S277S	NA	rs11078355	NA
chr17	18682505	18682505	T	C	exonic	FBXW10	nonsynonymous SNV	5:exon13:c.T2894C:p.V965A,FBXW10:NM_001267585:exc	ID=COSM436168;OCCURENCE=1(prostate)	rs1024657	B
chr17	18694277	18694277	G	A	exonic	TVP23B	nonsynonymous SNV	TVP23B:NM_016078:exon3:c.G164A:p.G55E	NA	rs61075345	B
chr17	20768134	20768134	C	T	UTR3	CCDC144NL	NA	NA	NA	rs201667905	NA
chr17	21217727	21217727	T	C	UTR3	MAP2K3	NA	NA	NA	rs2363370	NA
chr17	21217764	21217764	G	A	UTR3	MAP2K3	NA	NA	NA	rs2363192	NA
chr17	21311916	21311916	C	T	UTR5	KCNJ12,KCNJ18	NA	NA	NA	rs1657716	NA
chr17	21311940	21311940	T	C	UTR5	KCNJ12,KCNJ18	NA	NA	NA	rs1714903	NA
chr17	21319407	21319407	C	T	exonic	KCNJ12,KCNJ18	synonymous SNV	194958:exon3:c.C753T:p.I251I,KCNJ12:NM_021012:exon3	NA	rs16962951	NA
chr17	21319465	21319465	T	C	exonic	KCNJ12,KCNJ18	synonymous SNV	.94958:exon3:c.T811C:p.L271L,KCNJ12:NM_021012:exon3	ID=COSM312199;OCCURENCE=1(lung)	rs77724065	NA
chr17	21323033	21323033	C	T	UTR3	KCNJ12	NA	NA	NA	rs72842126	NA
chr17	21323034	21323034	G	A	UTR3	KCNJ12	NA	NA	NA	rs72842128	NA
chr17	21323049	21323049	C	T	UTR3	KCNJ12	NA	NA	NA	rs75724776	NA
chr17	26206414	26206414	C	A	UTR3	LYRM9	NA	NA	NA	rs3751972	NA
chr17	27187789	27187789	C	T	UTR3	ERAL1	NA	NA	NA	rs6803	NA
chr17	29862282	29862282	A	-	UTR3	RAB11FIP4	NA	NA	NA	rs35637322	NA
chr17	33307586	33307586	G	A	UTR5	LIG3	NA	NA	NA	rs12945428	NA
chr17	33448818	33448818	T	C	exonic	FNDC8	nonsynonymous SNV	FNDC8:NM_017559:exon1:c.T106C:p.S36P	NA	rs1871892	B
chr17	37223344	37223344	G	T	ncRNA_UTR3	PLXDC1	NA	NA	NA	rs72821577	NA
chr17	39211189	39211189	C	G	exonic	KRTAP2-2	nonsynonymous SNV	KRTAP2-2:NM_033032:exon1:c.G275C:p.C92S	NA	NA	NA
chr17	39395019	39395019	G	T	UTR3	KRTAP9-8	NA	NA	NA	rs74975222	NA
chr17	39727816	39727816	T	G	exonic	KRT9	synonymous SNV	KRT9:NM_000226:exon1:c.A429C:p.G143G	NA	rs8075921	NA
chr17	39958344	39958344	T	C	UTR3	LEPREL4	NA	NA	NA	rs1061843	NA
chr17	41064988	41064995	TTTTAGAA	-	UTR3	G6PC	NA	NA	NA	rs71157674	NA
chr17	42876925	42876925	-	A	UTR3	GJC1	NA	NA	NA	NA	NA
chr17	46802286	46802286	T	C	UTR3	HOXB13	NA	NA	NA	NA	NA
chr17	46941419	46941419	G	T	UTR3	CALCOCO2	NA	NA	NA	rs6504584	NA
chr17	54450134	54450134	G	A	exonic	ANKFN1	synonymous SNV	ANKFN1:NM_153228:exon6:c.G738A:p.K246K	NA	rs8069322	NA
chr17	54912339	54912339	G	A	exonic	DGKE	synonymous SNV	DGKE:NM_003647:exon2:c.G183A:p.G61G	NA	rs1048159	NA
chr17	60342186	60342186	T	C	ncRNA_exonic	TBC1D3P2	NA	NA	NA	rs11550753	NA
chr17	62122727	62122727	A	G	exonic	ERN1	nonsynonymous SNV	ERN1:NM_001433:exon20:c.T2645C:p.L882P	NA	NA	NA
chr17	62892413	62892413	G	A	exonic	LRRC37A3	synonymous SNV	LRRC37A3:NM_199340:exon3:c.C963T:p.A321A	NA	rs3894937	NA
chr17	64208285	64208285	C	G	exonic	APOH	nonsynonymous SNV	APOH:NM_000042:exon8:c.G1004C:p.W335S	NA	rs1801690	D
chr17	67017930	67017930	T	C	exonic	ABCA9	nonsynonymous SNV	ABCA9:NM_080283:exon18:c.A2354G:p.N785S	NA	rs17684521	D
chr17	72301432	72301432	A	G	exonic	DNAI2	synonymous SNV	2810:exon9:c.A1062G:p.E354E,DNAI2:NM_023036:exon9	NA	rs8073660	NA
chr17	72521897	72521897	G	A	exonic	CD300LB	synonymous SNV	CD300LB:NM_174892:exon2:c.C471T:p.I157I	NA	rs17553512	NA
chr17	74675810	74675810	G	A	UTR3	MXRA7	NA	NA	NA	rs1065604	NA
chr17	74922706	74922706	C	T	exonic	MGAT5B	synonymous SNV	F,MGAT5B:NM_001199172:exon10:c.C1185T:p.F395F,M(	NA	rs33966966	NA
chr17	74928758	74928758	C	T	exonic	MGAT5B	synonymous SNV	2F,MGAT5B:NM_001199172:exon11:c.C1323T:p.F441F,M	NA	rs79286496	NA
chr17	76497920	76497920	C	A	exonic	DNAH17	nonsynonymous SNV	DNAH17:NM_173628:exon34:c.G5226T:p.M1742I	NA	rs690844	NA
chr17	76525759	76525759	G	C	exonic	DNAH17	nonsynonymous SNV	DNAH17:NM_173628:exon22:c.C3302G:p.A1101G	NA	rs61741523	NA
chr17	76670610	76670610	T	G	UTR3	CYTH1	NA	NA	NA	NA	NA
chr18	334742	334742	C	T	exonic	COLEC12	nonsynonymous SNV	COLEC12:NM_130386:exon6:c.G1816A:p.G606S	NA	rs2305027	D
chr18	2943456	2943456	-	TGTT	ncRNA_exonic	LOC727896	NA	NA	NA	NA	NA
chr18	6986227	6986227	G	A	exonic	LAMA1	nonsynonymous SNV	LAMA1:NM_005559:exon37:c.C5288T:p.A1763V	NA	rs12607841	B
chr18	8069868	8069868	C	T	exonic	PTPRM	synonymous SNV	5244:exon8:c.C1317T:p.N439N,PTPRM:NM_002845:exon8	NA	rs2230601	NA
chr18	9887607	9887607	T	C	exonic	TXNDC2	synonymous SNV	98529:exon2:c.T1131C:p.S377S,TXNDC2:NM_032243:exon	NA	NA	NA
chr18	14542867	14542867	C	T	exonic	POTEC	synonymous SNV	POTEC:NM_001137671:exon1:c.G279A:p.T93T	NA	rs200779556	NA
chr18	14779969	14779969	C	G	exonic	ANKRD30B	nonsynonymous SNV	ANKRD30B:NM_001145029:exon11:c.C1431G:p.F477L	D=COSM1130088,COSM1130087;OCCURENCE=1(prostate	rs9675365	NA
chr18	20577669	20577669	G	A	exonic	RBBP8	synonymous SNV	705K,RBBP8:NM_203291:exon14:c.G2115A:p.K705K,RBBI	NA	rs17852769	NA
chr18	33848663	33848663	-	TACAT	UTR3	MOCOS	NA	NA	NA	rs111317566	NA
chr18	42644774	42644774	A	-	UTR3	SETBP1	NA	NA	NA	rs57937684	NA
chr18	43331751	43331751	A	T	UTR3	SLC14A1	NA	NA	NA	rs544373	NA
chr18	47088655	47088655	T	G	UTR5	LIPG	NA	NA	NA	rs34474737	NA
chr18	47908556	47908556	G	A	exonic	SKA1	nonsynonymous SNV	039535:exon4:c.G271A:p.V91I,SKA1:NM_145060:exon4:c	NA	rs6507992	B
chr18	48325302	48325302	C	A	UTR3	MRO	NA	NA	NA	rs2849256	NA
chr18	50432602	50432602	C	G	exonic	DCC	nonsynonymous SNV	DCC:NM_005215:exon3:c.C601G:p.R201G	NA	rs2229080	D
chr18	51887123	51887123	C	T	exonic	C18orf54	synonymous SNV	C18orf54:NM_173529:exon2:c.C181T:p.L61L	NA	rs1657904	NA
chr18	55862847	55862847	G	A	UTR5	NEDD4L	NA	NA	NA	rs1008899	NA
chr18	56962800	56962801	AA	-	UTR3	CPLX4	NA	NA	NA	rs61050481	NA
chr18	56963852	56963852	T	G	UTR3	CPLX4	NA	NA	NA	rs17696504	NA
chr18	59712463	59712463	G	A	UTR3	PIGN	NA	NA	NA	rs28375341	NA

chr18	59780393	59780393	T	A	exonic	PIGN	nonsynonymous SNV	27:exon15:c.A1408T:p.I470L,PIGN:NM_176787:exon16:c.	NA	rs3862712	NA
chr18	59814268	59814268	G	A	exonic	PIGN	synonymous SNV	327:exon8:c.C741T:p.H247H,PIGN:NM_176787:exon9:c.C	NA	rs9320000	NA
chr18	60237388	60237388	A	G	exonic	ZCCHC2	synonymous SNV	ZCCHC2:NM_017742:exon12:c.A1899G:p.S633S	NA	rs8096750	NA
chr18	61377579	61377579	A	C	exonic	SERPINB11	unknown	UNKNOWN	NA	rs1395268	NA
chr18	67509199	67509199	C	T	UTR3	DOK6	NA	NA	NA	rs72953879	NA
chr18	67509199	67509199	-	GTGTGTGT	UTR3	DOK6	NA	NA	NA	rs143503551	NA
chr18	67836217	67836217	A	G	exonic	RTTN	synonymous SNV	RTTN:NM_173630:exon12:c.T1563C:p.I521I	NA	rs200975487	NA
chr18	72168608	72168608	G	A	exonic	CNDP2	synonymous SNV	168499:exon2:c.G105A:p.P35P,CNDP2:NM_018235:exon3	ID=COSM148444;OCCURENCE=1(stomach)	rs2303463	NA
chr18	74728109	74728109	-	TT	UTR3	MBP	NA	NA	NA	NA	NA
chr19	1086993	1086993	T	-	UTR3	POLR2E	NA	NA	NA	NA	NA
chr19	1954128	1954128	T	C	ncRNA_exonic	CSNK1G2-AS1	NA	NA	NA	rs62129474	NA
chr19	3593004	3593004	A	G	UTR3	GIPC3	NA	NA	NA	rs17433775	NA
chr19	3767265	3767265	C	T	exonic	MRPL54	synonymous SNV	MRPL54:NM_172251:exon3:c.C291T:p.F97F	NA	rs7239	NA
chr19	3805793	3805793	T	C	UTR3	ZFR2	NA	NA	NA	rs4807513	NA
chr19	3823292	3823292	G	A	exonic	ZFR2	synonymous SNV	ZFR2:NM_015174:exon8:c.C1323T:p.P441P	NA	rs61744821	NA
chr19	4153655	4153655	G	T	UTR5	CREB3L3	NA	NA	NA	NA	NA
chr19	4236996	4236996	G	A	exonic	EBI3	nonsynonymous SNV	EBI3:NM_005755:exon5:c.G601A:p.V201I	NA	rs4740	B
chr19	4442999	4442999	T	G	exonic	CHAF1A	nonsynonymous SNV	CHAF1A:NM_005483:exon15:c.T2848G:p.S950A	NA	rs243383	B
chr19	4502937	4502937	A	C	UTR3	PLIN4	NA	NA	NA	NA	NA
chr19	5153606	5153606	G	A	UTR3	KDM4B	NA	NA	NA	rs11514	NA
chr19	5206775	5206775	A	G	UTR3	PTPRS	NA	NA	NA	rs1143700	NA
chr19	5910015	5910015	-	G	UTR3	VMAC	NA	NA	NA	rs76336657	NA
chr19	6312290	6312290	T	C	exonic	ACER1	nonsynonymous SNV	ACER1:NM_133492:exon3:c.A220G:p.M74V	NA	rs72981971	D
chr19	6718387	6718387	G	C	exonic	C3	nonsynonymous SNV	C3:NM_000064:exon3:c.C304G:p.R102G	NA	rs2230199	B
chr19	7056553	7056553	C	T	exonic	MBD3L3	nonsynonymous SNV	MBD3L3:NM_001164425:exon2:c.G407A:p.R136H	NA	rs199680919	NA
chr19	7547074	7547074	A	C	exonic	PEX11G	nonsynonymous SNV	70539:exon3:c.T273G:p.C91W,PEX11G:NM_080662:exon	NA	rs2303146	B
chr19	7977929	7977929	C	-	UTR3	MAP2K7	NA	NA	NA	NA	NA
chr19	7977931	7977933	CTC	-	UTR3	MAP2K7	NA	NA	NA	NA	NA
chr19	7978407	7978407	G	A	UTR3	MAP2K7	NA	NA	NA	rs4804835	NA
chr19	10089262	10089262	G	A	exonic	COL5A3	synonymous SNV	COL5A3:NM_015719:exon41:c.C3006T:p.P1002P	NA	rs12610207	NA
chr19	10179657	10179657	G	A	ncRNA_exonic	C3P1	NA	NA	NA	rs897803	NA
chr19	10664256	10664256	-	TAA	UTR3	KRI1	NA	NA	NA	rs201847069	NA
chr19	11406839	11406839	C	G	UTR5	TSPAN16	NA	NA	NA	rs453766	NA
chr19	12091196	12091196	C	A	UTR3	ZNF763	NA	NA	NA	rs78037958	NA
chr19	12091196	12091196	-	A	UTR3	ZNF763	NA	NA	NA	NA	NA
chr19	12774537	12774537	G	A	exonic	MAN2B1	nonsynonymous SNV	28:exon5:c.C743T:p.P248L,MAN2B1:NM_001173498:exo	NA	rs117843968	D
chr19	13054781	13054781	G	T	UTR3	CALR	NA	NA	NA	rs1049481	NA
chr19	14682804	14682804	G	C	exonic	NDUFB7	synonymous SNV	NDUFB7:NM_004146:exon1:c.C9G:p.A3A	NA	rs9543	NA
chr19	14769339	14769339	C	G	exonic	EMR3	nonsynonymous SNV	EMR3:NM_032571:exon5:c.G379C:p.E127Q	NA	rs4606855	B
chr19	15996820	15996820	G	A	exonic	CYP4F2	synonymous SNV	CYP4F2:NM_001082:exon9:c.C1029T:p.H343H	NA	rs2074900	NA
chr19	17452859	17452863	CTTCC	-	UTR3	GTPBP3	NA	NA	NA	rs5827368	NA
chr19	17660300	17660300	G	A	exonic	FAM129C	nonsynonymous SNV	524:exon15:c.G1807A:p.G603S,FAM129C:NM_173544:exc	ID=COSM148530;OCCURENCE=1(stomach)	rs11666267	B
chr19	18499815	18499815	G	C	UTR3	GDF15	NA	NA	NA	rs1054564	NA
chr19	18499858	18499858	T	C	UTR3	GDF15	NA	NA	NA	rs1054221	NA
chr19	19023853	19023853	T	C	exonic	COPE	synonymous SNV	G:p.L43L,COPE:NM_199442:exon2:c.A129G:p.L43L,COPE:N	ID=COSM1178458;OCCURENCE=1(prostate)	rs3177137	NA
chr19	20722787	20722787	A	T	UTR3	ZNF737	NA	NA	NA	rs138165402	NA
chr19	21306251	21306251	G	A	UTR3	ZNF714	NA	NA	NA	rs10413226	NA
chr19	22156855	22156855	G	A	exonic	ZNF208	synonymous SNV	ZNF208:NM_007153:exon4:c.C981T:p.T327T	NA	rs200969060	NA
chr19	22363413	22363413	C	T	exonic	ZNF676	nonsynonymous SNV	ZNF676:NM_001001411:exon3:c.G1106A:p.G369E	NA	rs75690456	B
chr19	22363826	22363826	G	C	exonic	ZNF676	synonymous SNV	ZNF676:NM_001001411:exon3:c.C693G:p.G231G	NA	rs7252593	NA
chr19	22375868	22375868	C	T	exonic	ZNF676	nonsynonymous SNV	ZNF676:NM_001001411:exon2:c.G80A:p.G27E	NA	rs8104929	B
chr19	22379587	22379587	G	T	UTR5	ZNF676	NA	NA	NA	rs2360001	NA
chr19	23556605	23556605	A	G	exonic	ZNF91	synonymous SNV	ZNF91:NM_003430:exon3:c.T192C:p.Y64Y	ID=COSM288088;OCCURENCE=1(large_intestine)	rs34740519	NA
chr19	32877006	32877006	A	C	UTR3	ZNF507	NA	NA	NA	NA	NA
chr19	33088072	33088072	-	ATCA	UTR3	ANKRD27	NA	NA	NA	rs143829091	NA
chr19	39980412	39980412	C	T	exonic	TIMM50	synonymous SNV	TIMM50:NM_001001563:exon11:c.C1323T:p.F441F	NA	rs114194716	NA
chr19	40389741	40389741	A	G	exonic	FCGBP	nonsynonymous SNV	FCGBP:NM_003890:exon18:c.T8441C:p.V2814A	NA	rs141158749	NA
chr19	41111069	41111069	G	A	exonic	LTBP4	unknown	UNKNOWN	NA	rs2303729	NA
chr19	41903220	41903220	G	A	exonic	EXOSC5	nonsynonymous SNV	EXOSC5:NM_020158:exon1:c.C14T:p.T5M	NA	rs10853751	B
chr19	42355886	42355886	-	TTTG	UTR3	DMRTC2	NA	NA	NA	NA	NA
chr19	44174249	44174249	C	T	exonic	PLAUR	synonymous SNV	A:p.P8P,PLAUR:NM_001005377:exon1:c.G24A:p.P8P,PLA	NA	rs62639324	NA
chr19	46093211	46093211	T	C	UTR3	GPR4	NA	NA	NA	rs1045217	NA

chr19	47824788	47824788	-	C	UTR3	C5AR1	NA	NA	NA	rs35042977	NA
chr19	48305566	48305566	C	T	exonic	TPRX1	synonymous SNV	TPRX1:NM_198479:exon2:c.G702A:p.P234P	ID=COSM998808;OCCURENCE=1(endometrium)	rs12463317	NA
chr19	48343993	48343993	G	A	UTR3	CRX	NA	NA	NA	rs55835533	NA
chr19	49571425	49571425	G	C	UTR3	KCNA7	NA	NA	NA	rs62127899	NA
chr19	51835892	51835892	-	G	exonic	VSIG10L	frameshift insertion	SIG10L:NM_001163922:exon10:c.2577_2578insC:p.A859	NA	NA	NA
chr19	51841417	51841417	C	T	exonic	VSIG10L	nonsynonymous SNV	VSIG10L:NM_001163922:exon6:c.G1775A:p.R592Q	NA	rs34380065	NA
chr19	51850290	51850290	G	A	exonic	ETFB	nonsynonymous SNV	.4763:exon4:c.C734T:p.T245M,ETFB:NM_001985:exon5:c	NA	rs1130426	P
chr19	53381429	53381429	G	T	UTR3	ZNF320	NA	NA	NA	rs2162919	NA
chr19	53381495	53381507	TTTTTTTTT	-	UTR3	ZNF320	NA	NA	NA	rs71183850	NA
chr19	53431516	53431516	T	C	ncRNA_exonic	ZNF321P	NA	NA	NA	rs12984436	NA
chr19	53914545	53914545	-	T	UTR3	ZNF765	NA	NA	NA	NA	NA
chr19	53996692	53996695	TTTC	-	UTR3	ZNF813	NA	NA	NA	rs139084525	NA
chr19	54378454	54378467	iTGTGTGTG	-	UTR3	MYADM	NA	NA	NA	NA	NA
chr19	54726870	54726870	C	G	UTR5	LILRB3	NA	NA	NA	rs117942579	NA
chr19	54962467	54962467	G	A	UTR5	LENG8	NA	NA	NA	rs1035451	NA
chr19	55247827	55247827	-	TCCT	UTR3	KIR3DL3	NA	NA	NA	NA	NA
chr19	55247987	55247987	-	CCTTTCCTC/	UTR3	KIR3DL3	NA	NA	NA	NA	NA
chr19	55253544	55253544	A	G	exonic	KIR2DL3	synonymous SNV	KIR2DL3:NM_015868:exon3:c.A189G:p.E63E	NA	rs34790392	NA
chr19	55253552	55253552	T	A	exonic	KIR2DL3	nonsynonymous SNV	KIR2DL3:NM_015868:exon3:c.T197A:p.F66Y	ID=COSM321259;OCCURENCE=1(lung)	rs673568	NA
chr19	55253601	55253601	G	A	exonic	KIR2DL3	synonymous SNV	KIR2DL3:NM_015868:exon3:c.G246A:p.K82K	NA	rs201337670	NA
chr19	55294454	55294454	C	T	exonic	KIR2DL1	nonsynonymous SNV	KIR2DL1:NM_014218:exon6:c.C796T:p.R266C	NA	rs151328241	NA
chr19	55330019	55330019	C	T	exonic	KIR3DL1	nonsynonymous SNV	KIR3DL1:NM_013289:exon3:c.C320T:p.S107L	NA	rs143159382	NA
chr19	55358681	55358681	T	A	exonic	KIR2DS4	unknown	UNKNOWN	NA	NA	NA
chr19	55401412	55401412	T	C	UTR3	FCAR	NA	NA	NA	rs59103589	NA
chr19	55401447	55401447	G	T	UTR3	FCAR	NA	NA	NA	rs58560391	NA
chr19	55401474	55401474	T	C	UTR3	FCAR	NA	NA	NA	rs60304316	NA
chr19	55401481	55401481	A	G	UTR3	FCAR	NA	NA	NA	rs59309328	NA
chr19	55424424	55424426	TTT	-	UTR3	NCR1	NA	NA	NA	rs200857637	NA
chr19	55435140	55435140	T	C	exonic	NLRP7	nonsynonymous SNV	A,NLRP7:NM_001127255:exon11:c.A3082G:p.T1028A,NL	NA	rs7256020	B
chr19	55441995	55441995	A	G	exonic	NLRP7	synonymous SNV	o.Y894Y,NLRP7:NM_139176:exon9:c.T2598C:p.Y866Y,NLR	NA	rs269951	NA
chr19	55715309	55715309	C	T	exonic	PTPRH	nonsynonymous SNV	PTPRH:NM_002842:exon5:c.G727A:p.V243I	NA	rs45535035	B
chr19	55715319	55715319	C	G	exonic	PTPRH	synonymous SNV	PTPRH:NM_002842:exon5:c.G717C:p.S239S	NA	rs9304763	NA
chr19	56047448	56047448	A	G	exonic	SBK2	nonsynonymous SNV	SBK2:NM_001101401:exon2:c.T214C:p.C72R	NA	rs310453	B
chr19	56162776	56162776	C	T	exonic	CCDC106	synonymous SNV	CCDC106:NM_013301:exon5:c.C441T:p.S147S	NA	rs2287791	NA
chr19	56388364	56388364	T	A	exonic	NLRP4	stopgain SNV	NLRP4:NM_134444:exon8:c.T2528A:p.L843X	NA	NA	NA
chr19	57770975	57770975	T	C	UTR3	ZNF805	NA	NA	NA	rs10420472	NA
chr19	57841082	57841082	G	A	UTR3	ZNF543	NA	NA	NA	rs78764250	NA
chr19	58968875	58968875	A	C	UTR3	ZNF324B	NA	NA	NA	rs1077420	NA
chr20	3677736	3677736	T	G	exonic	SIGLEC1	synonymous SNV	SIGLEC1:NM_023068:exon9:c.A2376C:p.V792V	NA	rs673114	NA
chr20	3762095	3762095	T	C	UTR5	SPEF1	NA	NA	NA	rs2281479	NA
chr20	6104068	6104068	C	T	UTR5	FERMT1	NA	NA	NA	rs2295433	NA
chr20	13695607	13695607	T	G	exonic	ESF1	nonsynonymous SNV	5380:exon14:c.A2470C:p.I824L,ESF1:NM_016649:exon14:	NA	rs34414644	B
chr20	17463053	17463053	G	A	UTR3	PCSK2	NA	NA	NA	NA	NA
chr20	25124088	25124088	G	A	ncRNA_exonic	LOC284798	NA	NA	NA	rs6050363	NA
chr20	25752057	25752057	G	A	ncRNA_exonic	FAM182B	NA	NA	NA	NA	NA
chr20	26064330	26064330	C	T	ncRNA_exonic	FAM182A	NA	NA	NA	rs151022794	NA
chr20	26064408	26064408	T	A	ncRNA_exonic	FAM182A	NA	NA	NA	NA	NA
chr20	29623159	29623159	A	G	ncRNA_exonic	FRG1B	NA	NA	NA	NA	NA
chr20	29632638	29632638	C	A	ncRNA_exonic	FRG1B	NA	NA	NA	rs9647043	NA
chr20	29633900	29633900	A	G	ncRNA_exonic	FRG1B	NA	NA	ID=COSM1136772,COSM1136771;OCCURENCE=1(kidney)	rs60081496	NA
chr20	31505189	31505189	C	T	intergenic	dist=66978),SUN5(di	NA	NA	NA	rs13043425	NA
chr20	31627291	31627291	A	G	exonic	BPIFB6	nonsynonymous SNV	BPIFB6:NM_174897:exon10:c.A1039G:p.S347G	NA	rs4911287	B
chr20	31676804	31676804	A	C	exonic	BPIFB4	nonsynonymous SNV	BPIFB4:NM_182519:exon6:c.A959C:p.N320T	NA	rs2889732	D
chr20	35740794	35740794	T	C	exonic	MROH8	unknown	UNKNOWN	NA	rs1744760	NA
chr20	42170327	42170327	C	T	UTR3	L3MBTL1	NA	NA	NA	rs1062943	NA
chr20	48570261	48570261	A	G	UTR3	RNF114	NA	NA	NA	rs141239729	NA
chr20	52184901	52184901	A	C	UTR3	ZNF217	NA	NA	NA	NA	NA
chr20	52560856	52560857	TT	-	UTR3	BCAS1	NA	NA	NA	NA	NA
chr20	52836074	52836074	T	C	UTR3	PFDN4	NA	NA	NA	rs6023044	NA
chr20	55940426	55940426	G	A	exonic	RAE1	synonymous SNV	.5885:exon5:c.G303A:p.V101V,RAE1:NM_003610:exon5:c	NA	rs6099582	NA
chr20	57571763	57571763	A	G	exonic	CTSZ	synonymous SNV	CTSZ:NM_001336:exon5:c.T732C:p.S244S	NA	rs9760	NA
chr20	57609945	57609945	C	A	ncRNA_UTR3	SLMO2	NA	NA	NA	NA	NA

chr20	57609945	57609945	-	A	ncRNA_UTR3	SLMO2	NA	NA	NA	NA	NA
chr20	60709531	60709531	T	-	UTR3	LSM14B	NA	NA	NA	rs11477488	NA
chr20	61941747	61941747	A	G	exonic	COL20A1	synonymous SNV	COL20A1:NM_020882:exon11:c.A1278G:p.G426G	NA	rs4809287	NA
chr21	15558346	15558346	G	A	exonic	LIPI	synonymous SNV	LIPI:NM_198996:exon3:c.C540T:p.S180S	NA	rs397517	NA
chr21	15646405	15646405	C	A	ncRNA_exonic	ABCC13	NA	NA	NA	rs2236001	NA
chr21	26969703	26969703	T	C	exonic	MRPL39	synonymous SNV	446:exon6:c.A612G:p.K204K,MRPL39:NM_080794:exon6	NA	rs1135618	NA
chr21	31964885	31964885	T	G	exonic	KRTAP6-3	nonsynonymous SNV	KRTAP6-3:NM_181605:exon1:c.T121G:p.C41G	NA	NA	NA
chr21	34923934	34923934	T	C	exonic	SON	synonymous SNV	195:exon3:c.T2397C:p.S799S,SON:NM_138927:exon3:c.T.	COSM1030344,COSM1030343;OCCURENCE=2(endometri	rs200724919	NA
chr21	35237608	35237608	T	C	exonic	ITSN1	synonymous SNV	ITSN1:NM_003024:exon32:c.T4044C:p.D1348D	NA	rs9976801	NA
chr21	38121746	38121750	TGACT	-	UTR3	SIM2	NA	NA	NA	rs113168269	NA
chr21	38391582	38391582	A	G	UTR3	DSCR6	NA	NA	NA	rs188497473	NA
chr21	43131734	43131734	T	A	ncRNA_exonic	LINC00479	NA	NA	NA	rs2838094	NA
chr21	43306769	43306769	T	-	UTR3	C2CD2	NA	NA	NA	rs11347156	NA
chr21	44451935	44451935	-	A	UTR3	PKNOX1	NA	NA	NA	rs138842830	NA
chr21	45647837	45647853	ACCACCCC(	-	UTR3	ICOSLG	NA	NA	NA	rs67226187	NA
chr22	17469049	17469049	C	A	exonic	GAB4	stopgain SNV	GAB4:NM_001037814:exon3:c.G487T:p.G163X	NA	rs28502153	NA
chr22	18520582	18520582	G	A	ncRNA_exonic	FLJ41941	NA	NA	NA	rs975826	NA
chr22	18775061	18775061	G	A	ncRNA_exonic	GGT3P	NA	NA	NA	rs189425726	NA
chr22	20387276	20387276	A	G	ncRNA_exonic	PI4KAP1	NA	NA	NA	rs2629350	NA
chr22	20390444	20390444	A	G	ncRNA_exonic	PI4KAP1	NA	NA	NA	rs1892850	NA
chr22	22054764	22054764	-	T	UTR3	YPEL1	NA	NA	NA	NA	NA
chr22	24314006	24314006	G	T	UTR3	DDTL	NA	NA	NA	rs1006771	NA
chr22	25006264	25006264	A	C	UTR5	GGT1	NA	NA	NA	rs199833779	NA
chr22	25023441	25023441	C	T	exonic	GGT1	nonsynonymous SNV	2365:exon12:c.C1063T:p.R355W,GGT1:NM_005265:exon1	NA	rs200419006	B
chr22	25024072	25024072	G	T	exonic	GGT1	nonsynonymous SNV	2365:exon14:c.G1361T:p.C454F,GGT1:NM_005265:exon1	NA	rs199681469	D
chr22	25043061	25043061	-	CCGG	ncRNA_exonic	POM121L10P	NA	NA	NA	NA	NA
chr22	26847961	26847972	CGCGCGCG	-	UTR3	HPS4	NA	NA	NA	NA	NA
chr22	29754944	29754944	T	G	exonic	AP1B1	nonsynonymous SNV	N99T,AP1B1:NM_001166019:exon5:c.A296C:p.N99T,AP1E	NA	NA	D
chr22	39079921	39079921	T	-	UTR3	TOMM22	NA	NA	NA	NA	NA
chr22	44276171	44276171	T	G	UTR3	PNPLA5	NA	NA	NA	rs470093	NA
chr22	44708655	44708655	T	C	UTR5	KIAA1644	NA	NA	NA	rs7510924	NA
chr22	45592689	45592689	T	G	UTR3	KIAA0930	NA	NA	NA	NA	NA
chrX	7023835	7023835	G	A	exonic	HDHD1	nonsynonymous SNV	178136:exon2:c.C106T:p.R36C,HDHD1:NM_012080:exon2	NA	NA	NA
chrX	8433461	8433461	T	C	UTR5	VCX3B	NA	NA	ID=COSN414126;OCCURENCE=1(urinary_tract)	rs200308612	NA
chrX	11129475	11129475	-	GGC	UTR5	HCCS	NA	NA	NA	rs35297358	NA
chrX	16859628	16859628	G	A	exonic	TXLNG	synonymous SNV	683:exon8:c.G930A:p.Q310Q,TXLNG:NM_018360:exon10	NA	rs5924530	NA
chrX	28807442	28807442	G	A	UTR5	IL1RAPL1	NA	NA	)=COSN414057,COSN414056;OCCURENCE=2(urinary_trac	rs6526806	NA
chrX	47341999	47341999	-	CGTGCGCG	UTR5	ZNF41	NA	NA	NA	NA	NA
chrX	48045837	48045837	T	C	UTR3	SSX5	NA	NA	NA	rs184913384	NA
chrX	48206014	48206023	TGTGTGTC	-	UTR3	SSX3	NA	NA	NA	rs72172699	NA
chrX	48770831	48770831	-	A	UTR3	PIM2	NA	NA	NA	NA	NA
chrX	48847497	48847497	T	C	exonic	GRIPAP1	synonymous SNV	672:exon6:c.A324G:p.E108E,GRIPAP1:NM_020137:exon7	NA	rs11545861	NA
chrX	68061228	68061228	-	C	UTR3	EFNB1	NA	NA	NA	NA	NA
chrX	73037544	73037544	C	A	ncRNA_exonic	TSIX	NA	NA	NA	rs5937751	NA
chrX	91089881	91089881	C	G	UTR5	PCDH11X	NA	NA	NA	rs3795211	NA
chrX	1.1E+08	1.1E+08	GAGG	-	UTR3	CHRDL1	NA	NA	NA	rs71931043	NA
chrX	1.19E+08	1.19E+08	G	A	exonic	CXorf56	synonymous SNV	5111G,CXorf56:NM_001170569:exon4:c.C228T:p.G76G,C)	NA	rs5910611	NA
chrX	1.31E+08	1.31E+08	GA	-	UTR3	RAP2C	NA	NA	NA	NA	NA
chrX	1.41E+08	1.41E+08	G	C	exonic	MAGEC1	nonsynonymous SNV	MAGEC1:NM_005462:exon4:c.G841C:p.V281L	NA	rs176044	NA
chrX	1.48E+08	1.48E+08	G	A	UTR3	AFF2	NA	NA	NA	rs144890448	NA
chrX	1.52E+08	1.52E+08	C	T	UTR3	PNMA5	NA	NA	NA	NA	NA
chrX	1.52E+08	1.52E+08	A	G	UTR3	PNMA5	NA	NA	NA	NA	NA
chrX	1.53E+08	1.53E+08	CTCTCTCTC	-	UTR3	BGN	NA	NA	NA	rs36205859	NA

H2O2 Mutations

Chr	Start	End	Ref	Alt	Func.refGene	Gene.refGene	ExonicFunc.refGene	AAChange.refGene	cosmic64	snp137	PolyPhen2 Pred
chr1		69511	A	G	exonic	OR4F5	nonsynonymous SNV	OR4F5:NM_001005484:exon1:c.A421G:p.T141A	NA	rs2691305	B
chr1	663815	663815	G	A	ncRNA_exonic	LOC100133331	NA	NA	NA	rs67356487	NA
chr1	788844	788844	C	G	ncRNA_exonic	LOC643837	NA	NA	NA	rs10159337	NA
chr1	1263706	1263706	T	C	UTR3	GLTPD1	NA	NA	NA	NA	NA
chr1	1375814	1375814	A	C	UTR3	VWA1	NA	NA	NA	NA	NA
chr1	1375819	1375819	A	C	UTR3	VWA1	NA	NA	NA	rs146709436	NA
chr1	1469598	1469598	C	G	UTR3	ATAD3A	NA	NA	NA	rs9439464	NA
chr1	1886475	1886475	C	A	UTR3	KIAA1751	NA	NA	NA	NA	NA
chr1	1900232	1900232	T	C	exonic	KIAA1751	nonsynonymous SNV	KIAA1751:NM_001080484:exon11:c.A1087G:p.I363V	NA	rs16824588	NA
chr1	2123473	2123473	T	C	UTR3	C1orf86	NA	NA	NA	NA	NA
chr1	2282610	2282610	T	G	ncRNA_exonic	LOC100129534	NA	NA	NA	NA	NA
chr1	2984087	2984087	C	A	ncRNA_exonic	FLJ42875	NA	NA	NA	rs2297829	NA
chr1	3352764	3352764	T	-	UTR3	PRDM16	NA	NA	NA	rs35439737	NA
chr1	6526858	6526858	A	-	UTR3	PLEKHG5	NA	NA	NA	NA	NA
chr1	7828174	7828174	A	-	UTR3	CAMTA1	NA	NA	NA	rs59402168	NA
chr1	10439620	10439620	-	G	UTR3	KIF1B	NA	NA	NA	NA	NA
chr1	12203319	12203319	T	C	UTR3	TNFRSF8	NA	NA	NA	NA	NA
chr1	12788406	12788406	G	A	UTR3	AADACL3	NA	NA	NA	rs3000929	NA
chr1	12854068	12854068	T	C	exonic	PRAMEF1	nonsynonymous SNV	PRAMEF1:NM_023013:exon3:c.T292C:p.W98R	NA	rs71245736	P
chr1	12856010	12856010	C	G	exonic	PRAMEF1	nonsynonymous SNV	PRAMEF1:NM_023013:exon4:c.C1290G:p.F430L	NA	rs2142947	B
chr1	12856111	12856111	C	T	exonic	PRAMEF1	nonsynonymous SNV	PRAMEF1:NM_023013:exon4:c.C1391T:p.P464L	NA	rs2982215	D
chr1	12887453	12887453	C	T	exonic	PRAMEF11	nonsynonymous SNV	PRAMEF11:NM_001146344:exon3:c.G404A:p.R135K	NA	rs2486714	NA
chr1	12887480	12887480	C	T	exonic	PRAMEF11	nonsynonymous SNV	PRAMEF11:NM_001146344:exon3:c.G377A:p.C126Y	NA	NA	NA
chr1	12919044	12919044	T	C	exonic	PRAMEF2	synonymous SNV	PRAMEF2:NM_023014:exon2:c.T180C:p.P60P	NA	NA	NA
chr1	12921265	12921265	G	A	exonic	PRAMEF2	synonymous SNV	PRAMEF2:NM_023014:exon4:c.G1056A:p.E352E	NA	rs151184374	NA
chr1	12921272	12921272	G	A	exonic	PRAMEF2	nonsynonymous SNV	PRAMEF2:NM_023014:exon4:c.G1063A:p.V355M	NA	rs145044541	B
chr1	12921274	12921274	G	C	exonic	PRAMEF2	synonymous SNV	PRAMEF2:NM_023014:exon4:c.G1065C:p.V355V	NA	rs17039302	NA
chr1	12941832	12941832	T	C	exonic	PRAMEF4	nonsynonymous SNV	PRAMEF4:NM_001009611:exon3:c.A718G:p.I240V	NA	rs6686974	NA
chr1	12942930	12942930	G	A	exonic	PRAMEF4	nonsynonymous SNV	PRAMEF4:NM_001009611:exon2:c.C286T:p.R96C	NA	rs2994117	NA
chr1	12953195	12953195	C	T	exonic	PRAMEF10	nonsynonymous SNV	PRAMEF10:NM_001039361:exon4:c.G977A:p.R326H	NA	rs201422133	B
chr1	13036294	13036294	G	T	exonic	PRAMEF22	nonsynonymous SNV	PRAMEF22:NM_001100631:exon2:c.G366T:p.R122S	NA	rs200761485	D
chr1	13183188	13183188	T	A	exonic	LOC440563	nonsynonymous SNV	LOC440563:NM_001136561:exon2:c.A685T:p.M229L	NA	rs144308453	NA
chr1	13183528	13183528	A	C	exonic	LOC440563	nonsynonymous SNV	LOC440563:NM_001136561:exon2:c.T345G:p.F115L	NA	rs76937665	NA
chr1	13183532	13183532	C	T	exonic	LOC440563	nonsynonymous SNV	LOC440563:NM_001136561:exon2:c.G341A:p.G114D	NA	rs77505369	NA
chr1	13183533	13183533	C	T	exonic	LOC440563	nonsynonymous SNV	LOC440563:NM_001136561:exon2:c.G340A:p.G114S	NA	rs77556803	NA
chr1	13183780	13183780	A	C	exonic	LOC440563	synonymous SNV	LOC440563:NM_001136561:exon2:c.T93G:p.S31S	OSN199380;OCCURENCE=1(large_intes	rs116484938	NA
chr1	15439082	15439082	A	G	ncRNA_exonic	TMEM51-AS1	NA	NA	NA	NA	NA
chr1	15439098	15439098	C	T	ncRNA_exonic	TMEM51-AS1	NA	NA	NA	rs114844404	NA
chr1	15440343	15440343	T	G	ncRNA_exonic	TMEM51-AS1	NA	NA	NA	NA	NA
chr1	15444487	15444487	G	A	ncRNA_UTR3	KAZN	NA	NA	NA	rs2235783	NA
chr1	15896199	15896199	G	A	UTR3	DNAJC16	NA	NA	NA	rs2145397	NA
chr1	16333143	16333146	AATA	-	UTR3	C1orf64	NA	NA	NA	rs146554621	NA
chr1	16385178	16385178	G	A	exonic	FAM131C	synonymous SNV	FAM131C:NM_182623:exon7:c.C597T:p.P199P	OSM676526;OCCURENCE=1(prostate),1	rs80297394	NA
chr1	16793964	16793964	C	T	ncRNA_exonic	CROCCP3	NA	NA	NA	NA	NA
chr1	16890428	16890428	T	A	UTR3	NBPF1	NA	NA	NA	rs3900708	NA
chr1	16913677	16913677	T	C	exonic	NBPF1	unknown	UNKNOWN	NA	rs200743207	NA
chr1	16945182	16945184	AAT	-	ncRNA_exonic	CROCCP2	NA	NA	ID=COSN385177;OCCURENCE=1(lung)	NA	NA
chr1	16945409	16945409	T	A	ncRNA_exonic	CROCCP2	NA	NA	NA	rs13566	NA
chr1	16945504	16945504	C	T	ncRNA_exonic	CROCCP2	NA	NA	NA	rs11378	NA
chr1	16973095	16973095	G	C	ncRNA_exonic	MST1P2	NA	NA	NA	rs75521993	NA
chr1	16974021	16974021	G	A	ncRNA_exonic	MST1P2	NA	NA	NA	rs142740321	NA
chr1	16975268	16975268	T	C	ncRNA_exonic	MST1P2	NA	NA	NA	rs199945491	NA
chr1	16976641	16976641	C	T	ncRNA_exonic	MST1P2	NA	NA	NA	rs1057417	NA
chr1	16976649	16976649	C	T	ncRNA_exonic	MST1P2	NA	NA	NA	rs1057333	NA
chr1	16976681	16976681	G	T	ncRNA_exonic	MST1P2	NA	NA	ID=COSN162976;OCCURENCE=1(breast)	rs3175284	NA
chr1	16976825	16976825	C	A	ncRNA_exonic	MST1P2	NA	NA	ID=COSN404465;OCCURENCE=1(lung)	rs2761525	NA
chr1	17023126	17023126	C	G	ncRNA_exonic	ESPNP	NA	NA	NA	rs2610633	NA
chr1	17082975	17082975	A	G	UTR3	MST1L	NA	NA	NA	rs1033403	NA
chr1	17083101	17083101	A	G	UTR3	MST1L	NA	NA	NA	rs199727569	NA
chr1	17083787	17083787	G	A	exonic	MST1L	synonymous SNV	MST1L:NM_001271733:exon15:c.C2010T:p.A670A	NA	rs201551314	NA
chr1	22418253	22418253	G	T	UTR3	CDC42	NA	NA	NA	NA	NA
chr1	23518535	23518535	T	G	UTR3	HTR1D	NA	NA	NA	NA	NA
chr1	23518571	23518571	T	G	UTR3	HTR1D	NA	NA	NA	NA	NA
chr1	23695629	23695629	T	G	ncRNA_exonic	C1orf213	NA	NA	NA	NA	NA

chr1	26161107	26161108	AA	-	UTR3	AUNIP	NA	NA	NA	rs139695812	NA
chr1	26608814	26608814	A	G	exonic	UBXN11	synonymous SNV	79C:p.P393P,UBXN11:NM_145345:exon15:c.T1440C:p.P480P,UBXN11	NA	rs6672357	NA
chr1	26608823	26608823	A	G	exonic	UBXN11	synonymous SNV	70C:p.S390S,UBXN11:NM_145345:exon15:c.T1431C:p.S477S,UBXN11:	NA	rs61775084	NA
chr1	26608829	26608829	G	A	exonic	UBXN11	synonymous SNV	4T:p.G388G,UBXN11:NM_145345:exon15:c.C1425T:p.G475G,UBXN11	NA	rs61775085	NA
chr1	26608831	26608831	C	T	exonic	UBXN11	nonsynonymous SNV	2A:p.G388S,UBXN11:NM_145345:exon15:c.G1423A:p.G475S,UBXN11	NA	rs61775086	NA
chr1	26608835	26608835	A	T	exonic	UBXN11	synonymous SNV	8A:p.G386G,UBXN11:NM_145345:exon15:c.T1419A:p.G473G,UBXN11	NA	rs61775087	NA
chr1	26797045	26797045	A	C	UTR3	DHDDS	NA	NA	NA	NA	NA
chr1	27190855	27190855	T	C	UTR3	SFN	NA	NA	NA	rs112547702	NA
chr1	27248273	27248273	C	A	UTR5	NUDC	NA	NA	NA	rs41311274	NA
chr1	28087357	28087357	A	T	UTR3	FAM76A	NA	NA	NA	NA	NA
chr1	28087366	28087366	C	T	UTR3	FAM76A	NA	NA	NA	NA	NA
chr1	28087368	28087368	A	T	UTR3	FAM76A	NA	NA	NA	NA	NA
chr1	28087371	28087371	C	T	UTR3	FAM76A	NA	NA	NA	NA	NA
chr1	28473786	28473786	T	G	UTR3	PTAFR	NA	NA	NA	NA	NA
chr1	29063236	29063236	G	A	UTR5	YTHDF2	NA	NA	NA	rs3738067	NA
chr1	31838551	31838551	-	A	UTR3	FABP3	NA	NA	NA	NA	NA
chr1	32799514	32799514	A	-	UTR3	MARCKSL1	NA	NA	NA	rs113450741	NA
chr1	33475967	33475967	G	A	UTR3	AK2	NA	NA	NA	rs74066437	NA
chr1	36386884	36386884	C	T	UTR3	AGO1	NA	NA	NA	NA	NA
chr1	36386884	36386884	-	T	UTR3	AGO1	NA	NA	NA	NA	NA
chr1	38226760	38226760	-	AA	UTR3	EPHA10	NA	NA	NA	NA	NA
chr1	38267253	38267253	G	A	UTR3	MANEAL	NA	NA	NA	rs28489009	NA
chr1	39879211	39879211	A	G	exonic	KIAA0754	nonsynonymous SNV	KIAA0754:NM_015038:exon1:c.A3274G:p.T1092A	NA	NA	NA
chr1	39879379	39879379	C	G	exonic	KIAA0754	nonsynonymous SNV	KIAA0754:NM_015038:exon1:c.C3442G:p.P1148A	NA	rs34074681	NA
chr1	39976865	39976865	G	A	exonic	BMP8A	synonymous SNV	BMP8A:NM_181809:exon2:c.G354A:p.L118L	NA	NA	NA
chr1	40149642	40149642	G	T	exonic	HPCAL4	synonymous SNV	HPCAL4:NM_016257:exon3:c.C345A:p.R115R	NA	rs784634	NA
chr1	41154813	41154813	C	G	ncRNA_exonic	LOC100130557	NA	NA	NA	rs552147	NA
chr1	43272841	43272841	A	T	UTR3	CCDC23	NA	NA	NA	rs1044898	NA
chr1	43747937	43747937	-	A	UTR3	C1orf210	NA	NA	NA	NA	NA
chr1	44600190	44600191	CT	-	UTR3	KLF17	NA	NA	NA	rs34068395	NA
chr1	46806393	46806393	T	C	UTR5	NSUN4	NA	NA	NA	rs41293273	NA
chr1	46806429	46806429	A	T	UTR5	NSUN4	NA	NA	NA	rs41293275	NA
chr1	47905783	47905785	TTT	-	UTR3	FOXD2	NA	NA	NA	rs56809638	NA
chr1	50667062	50667062	-	A	UTR3	ELAVL4	NA	NA	NA	NA	NA
chr1	53291327	53291328	AA	-	UTR3	ZYG11B	NA	NA	NA	NA	NA
chr1	53535478	53535478	G	A	exonic	PODN	nonsynonymous SNV	M_153703:exon2:c.G95A:p.R32H,PODN:NM_001199081:exon3:c.G38	NA	rs1288386	P
chr1	61869783	61869783	G	A	exonic	NFIA	synonymous SNV	001145511:exon8:c.G1059A:p.P353P,NFIA:NM_005595:exon8:c.G108	NA	rs2236059	NA
chr1	61922425	61922425	A	-	UTR3	NFIA	NA	NA	NA	rs67346536	NA
chr1	65901244	65901244	G	T	UTR3	LEPROT	NA	NA	NA	NA	NA
chr1	67465988	67465988	A	-	UTR3	SLC35D1	NA	NA	NA	rs36007577	NA
chr1	67465989	67465989	A	T	UTR3	SLC35D1	NA	NA	NA	NA	NA
chr1	89446108	89446108	T	-	UTR3	RBMXL1	NA	NA	NA	NA	NA
chr1	89448896	89448896	A	T	exonic	RBMXL1	nonsynonymous SNV	_019610:exon2:c.T614A:p.V205D,RBMXL1:NM_001162536:exon3:c.T6	NA	rs200284841	B
chr1	90486412	90486412	T	G	exonic	ZNF326	synonymous SNV	ZNF326:NM_182976:exon10:c.T1236G:p.V412V	NA	rs2816881	NA
chr1	94586693	94586693	G	A	UTR5	ABCA4	NA	NA	NA	rs200102393	NA
chr1	94995411	94995411	C	A	UTR3	F3	NA	NA	NA	rs841691	NA
chr1	94995491	94995491	C	T	UTR3	F3	NA	NA	NA	rs841692	NA
chr1	95448279	95448279	G	A	UTR3	ALG14	NA	NA	NA	NA	NA
chr1	97279218	97279222	TTTTT	-	UTR3	PTBP2	NA	NA	NA	rs72223356	NA
chr1	1.1E+08	1.1E+08	A	-	UTR3	SORT1	NA	NA	NA	rs111233099	NA
chr1	1.1E+08	1.1E+08	T	-	UTR3	GNAI3	NA	NA	NA	NA	NA
chr1	1.11E+08	1.11E+08	-	C	UTR3	LRIF1	NA	NA	NA	rs142921680	NA
chr1	1.12E+08	1.12E+08	C	-	UTR3	CEPT1	NA	NA	NA	NA	NA
chr1	1.13E+08	1.13E+08	T	C	exonic	CTTNBP2NL	synonymous SNV	CTTNBP2NL:NM_018704:exon4:c.T234C:p.N78N	=COSM146582;OCCURENCE=1(stomac	rs2256883	NA
chr1	1.15E+08	1.15E+08	G	T	exonic	AMPD1	synonymous SNV	_001172626:exon3:c.C322A:p.R108R,AMPD1:NM_000036:exon4:c.C3	NA	NA	NA
chr1	1.18E+08	1.18E+08	TT	-	UTR3	PTGFRN	NA	NA	NA	NA	NA
chr1	1.18E+08	1.18E+08	G	T	UTR3	TRIM45	NA	NA	NA	rs12118664	NA
chr1	1.2E+08	1.2E+08	T	C	exonic	WARS2	nonsynonymous SNV	WARS2:NM_015836:exon6:c.A866G:p.E289G	NA	NA	B
chr1	1.21E+08	1.21E+08	T	C	exonic	NOTCH2	nonsynonymous SNV	_001200001:exon2:c.A137G:p.N46S,NOTCH2:NM_024408:exon2:c.A1	NA	rs141882865	B
chr1	1.21E+08	1.21E+08	C	T	exonic	NOTCH2	nonsynonymous SNV	_001200001:exon2:c.G112A:p.E38K,NOTCH2:NM_024408:exon2:c.G1	NA	rs146242578	B
chr1	1.21E+08	1.21E+08	G	C	exonic	NOTCH2	nonsynonymous SNV	_001200001:exon1:c.C57G:p.C19W,NOTCH2:NM_024408:exon1:c.C5	132738;OCCURENCE=1(central_nervou	rs11810554	NA
chr1	1.21E+08	1.21E+08	G	A	exonic	NOTCH2	synonymous SNV	NM_001200001:exon1:c.C15T:p.R5R,NOTCH2:NM_024408:exon1:c.C1	NA	rs77243975	NA
chr1	1.21E+08	1.21E+08	G	T	UTR5	NOTCH2	NA	NA	NA	rs57122008	NA
chr1	1.21E+08	1.21E+08	G	A	UTR3	FCGR1B	NA	NA	NA	rs15571	NA
chr1	1.45E+08	1.45E+08	C	T	ncRNA_exonic	PFN1P2	NA	NA	NA	rs17855859	NA
chr1	1.45E+08	1.45E+08	T	G	exonic	NBPF8,NBPF9	unknown	UNKNOWN	NA	rs678781	NA

chr1	1.45E+08	1.45E+08	TATT	-	UTR3	PDE4DIP	NA	NA	NA	NA	NA
chr1	1.45E+08	1.45E+08	T	C	exonic	PDE4DIP	nonsynonymous SNV	p.E2223G,PDE4DIP:NM_014644:exon40:c.A6668G:p.E2223G,PDE4DIP:	=COSM1127421;OCCURENCE=2(prostat	rs3844239	B
chr1	1.45E+08	1.45E+08	T	C	exonic	SEC22B	unknown	UNKNOWN	NA	rs2590131	NA
chr1	1.45E+08	1.45E+08	C	T	exonic	SEC22B	unknown	UNKNOWN	NA	rs2794040	NA
chr1	1.45E+08	1.45E+08	A	G	UTR5	NOTCH2NL	NA	NA	SN404541,COSN404542;OCCURENCE=1	rs56159748	NA
chr1	1.45E+08	1.45E+08	A	G	exonic	NOTCH2NL	synonymous SNV	NOTCH2NL:NM_203458:exon3:c.A156G:p.R52R	NA	rs10910778	NA
chr1	1.45E+08	1.45E+08	C	T	exonic	NOTCH2NL	nonsynonymous SNV	NOTCH2NL:NM_203458:exon4:c.C473T:p.T158I	NA	rs75586173	D
chr1	1.45E+08	1.45E+08	T	G	UTR3	NOTCH2NL	NA	NA	NA	rs1062341	NA
chr1	1.45E+08	1.45E+08	G	A	UTR3	NOTCH2NL	NA	NA	NA	rs201876339	NA
chr1	1.45E+08	1.45E+08	G	A	UTR3	NOTCH2NL	NA	NA	NA	rs4649854	NA
chr1	1.45E+08	1.45E+08	G	A	UTR3	NOTCH2NL	NA	NA	NA	rs4649855	NA
chr1	1.45E+08	1.45E+08	A	G	UTR3	NOTCH2NL	NA	NA	NA	rs4649856	NA
chr1	1.45E+08	1.45E+08	G	A	UTR3	NOTCH2NL	NA	NA	NA	rs61814565	NA
chr1	1.45E+08	1.45E+08	A	C	UTR3	NOTCH2NL	NA	NA	NA	NA	NA
chr1	1.45E+08	1.45E+08	T	C	exonic	NBPF10	nonsynonymous SNV	NBPF10:NM_001039703:exon10:c.T1462C:p.S488P	M462841,COSM462842;OCCURENCE=2	rs199529643	NA
chr1	1.45E+08	1.45E+08	C	T	exonic	NBPF10	synonymous SNV	NBPF10:NM_001039703:exon14:c.C1983T:p.Y661Y	NA	NA	NA
chr1	1.45E+08	1.45E+08	A	G	exonic	NBPF10	nonsynonymous SNV	NBPF10:NM_001039703:exon14:c.A2014G:p.I672V	NA	NA	NA
chr1	1.45E+08	1.45E+08	T	C	intronic	NBPF10	NA	NA	NA	NA	NA
chr1	1.45E+08	1.45E+08	GAAGAC	-	intronic	NBPF10	NA	NA	NA	NA	NA
chr1	1.45E+08	1.45E+08	A	C	intronic	NBPF10	NA	NA	NA	rs12128680	NA
chr1	1.46E+08	1.46E+08	A	-	ncRNA_exonic	GNRHR2	NA	NA	NA	rs148070621	NA
chr1	1.47E+08	1.47E+08	A	G	UTR5	BCL9	NA	NA	NA	rs10900376	NA
chr1	1.49E+08	1.49E+08	G	A	exonic	NBPF16	nonsynonymous SNV	NBPF16:NM_001102663:exon16:c.G1777A:p.G593S	NA	rs201036679	B
chr1	1.49E+08	1.49E+08	A	G	exonic	NBPF16	nonsynonymous SNV	NBPF16:NM_001102663:exon16:c.A1828G:p.R610G	NA	rs16826707	B
chr1	1.49E+08	1.49E+08	A	C	exonic	NBPF16	synonymous SNV	NBPF16:NM_001102663:exon16:c.C1844C:p.P615P	NA	rs6695216	B
chr1	1.49E+08	1.49E+08	T	C	ncRNA_exonic	LOC388692	NA	NA	NA	rs61789482	NA
chr1	1.49E+08	1.49E+08	C	G	ncRNA_exonic	LOC388692	NA	NA	NA	rs144294857	NA
chr1	1.49E+08	1.49E+08	A	G	ncRNA_exonic	LOC388692	NA	NA	NA	rs2454126	NA
chr1	1.51E+08	1.51E+08	-	A	UTR3	GOLPH3L	NA	NA	NA	NA	NA
chr1	1.52E+08	1.52E+08	T	G	UTR3	CGN	NA	NA	NA	NA	NA
chr1	1.52E+08	1.52E+08	TTTCTTTC	-	UTR3	SNX27	NA	NA	NA	NA	NA
chr1	1.52E+08	1.52E+08	C	T	exonic	RPTN	nonsynonymous SNV	RPTN:NM_001122965:exon3:c.G1510A:p.G504R	NA	NA	B
chr1	1.52E+08	1.52E+08	T	C	exonic	RPTN	synonymous SNV	RPTN:NM_001122965:exon3:c.A1035G:p.Q345Q	NA	NA	NA
chr1	1.52E+08	1.52E+08	A	G	exonic	RPTN	synonymous SNV	RPTN:NM_001122965:exon3:c.T1014C:p.G338G	NA	NA	NA
chr1	1.52E+08	1.52E+08	T	A	exonic	HRNR	synonymous SNV	HRNR:NM_001009931:exon3:c.A6198T:p.P2066P	NA	NA	NA
chr1	1.52E+08	1.52E+08	C	T	exonic	HRNR	nonsynonymous SNV	HRNR:NM_001009931:exon3:c.G4241A:p.C1414Y	NA	rs111689937	NA
chr1	1.52E+08	1.52E+08	G	A	exonic	HRNR	synonymous SNV	HRNR:NM_001009931:exon3:c.C3993T:p.G1331G	NA	rs77559827	NA
chr1	1.52E+08	1.52E+08	C	T	exonic	FLG2	synonymous SNV	FLG2:NM_001014342:exon3:c.G2595A:p.S865S	NA	rs12738471	NA
chr1	1.52E+08	1.52E+08	A	G	exonic	FLG2	synonymous SNV	FLG2:NM_001014342:exon3:c.T1851C:p.S617S	=COSM1127384;OCCURENCE=1(prostat	rs6679449	NA
chr1	1.53E+08	1.53E+08	A	C	exonic	KPRP	nonsynonymous SNV	KPRP:NM_001025231:exon2:c.A845C:p.Y282S	NA	NA	D
chr1	1.53E+08	1.53E+08	A	C	exonic	KPRP	synonymous SNV	KPRP:NM_001025231:exon2:c.A891C:p.P297P	NA	NA	NA
chr1	1.53E+08	1.53E+08	T	A	exonic	LCE1D	synonymous SNV	LCE1D:NM_178352:exon2:c.T81A:p.P27P	NA	NA	NA
chr1	1.53E+08	1.53E+08	A	G	UTR3	LCE1C	NA	NA	NA	NA	NA
chr1	1.53E+08	1.53E+08	T	A	exonic	IVL	nonsynonymous SNV	IVL:NM_005547:exon2:c.T614A:p.L205H	NA	NA	NA
chr1	1.53E+08	1.53E+08	T	A	exonic	IVL	nonsynonymous SNV	IVL:NM_005547:exon2:c.T644A:p.L215H	NA	NA	NA
chr1	1.53E+08	1.53E+08	T	A	exonic	IVL	nonsynonymous SNV	IVL:NM_005547:exon2:c.T674A:p.L225H	NA	NA	NA
chr1	1.53E+08	1.53E+08	A	G	exonic	IVL	synonymous SNV	IVL:NM_005547:exon2:c.A1563G:p.K521K	NA	NA	NA
chr1	1.53E+08	1.53E+08	T	G	exonic	LOR	synonymous SNV	LOR:NM_000427:exon2:c.T822G:p.G274G	NA	NA	NA
chr1	1.53E+08	1.53E+08	G	A	exonic	LOR	synonymous SNV	LOR:NM_000427:exon2:c.G870A:p.G290G	NA	rs12043009	NA
chr1	1.54E+08	1.54E+08	A	-	UTR3	GATAD2B	NA	NA	NA	NA	NA
chr1	1.54E+08	1.54E+08	-	A	UTR3	TPM3	NA	NA	NA	NA	NA
chr1	1.55E+08	1.55E+08	T	C	UTR5	ADAR	NA	NA	NA	NA	NA
chr1	1.55E+08	1.55E+08	A	C	UTR5	ADAR	NA	NA	COSN416145;OCCURENCE=1(urinary_tr	NA	NA
chr1	1.55E+08	1.55E+08	T	G	exonic	KCNN3	nonsynonymous SNV	_001204087:exon1:c.A707C:p.H236P,KCNN3:NM_002249:exon1:c.A70	NA	NA	NA
chr1	1.55E+08	1.55E+08	T	C	ncRNA_exonic	GBAP1	NA	NA	NA	rs28445596	NA
chr1	1.55E+08	1.55E+08	G	A	ncRNA_exonic	GBAP1	NA	NA	NA	rs1057941	NA
chr1	1.55E+08	1.55E+08	A	C	exonic	RUSC1	synonymous SNV	)1105203:exon2:c.A1089C:p.A363A,RUSC1:NM_001105204:exon2:c.A1	NA	NA	NA
chr1	1.55E+08	1.55E+08	A	C	exonic	RUSC1	nonsynonymous SNV	)1105203:exon2:c.A1105C:p.T369P,RUSC1:NM_001105204:exon2:c.A1	NA	NA	D
chr1	1.58E+08	1.58E+08	C	T	UTR3	FCRL1	NA	NA	NA	rs1016849	NA
chr1	1.6E+08	1.6E+08	AC	-	UTR3	KCNJ10	NA	NA	NA	NA	NA
chr1	1.6E+08	1.6E+08	T	A	ncRNA_exonic	SUMO1P3	NA	NA	NA	NA	NA
chr1	1.61E+08	1.61E+08	-	A	UTR3	DEDD	NA	NA	NA	NA	NA
chr1	1.61E+08	1.61E+08	C	A	UTR5	PPOX	NA	NA	NA	rs2301286	NA
chr1	1.61E+08	1.61E+08	C	G	UTR3	MPZ	NA	NA	NA	NA	NA
chr1	1.62E+08	1.62E+08	A	G	exonic	FCGR3B	synonymous SNV	.T219C:p.L73L,FCGR3B:NM_001271036:exon3:c.T63C:p.L21L,FCGR3B:I	NA	rs447536	NA
chr1	1.63E+08	1.63E+08	ACAC	-	UTR3	RGS4	NA	NA	NA	rs5778313	NA

chr1	1.68E+08	1.68E+08	T	C	exonic	ADCY10	nonsynonymous SNV	001167749:exon14:c.A1630G:p.I544V,ADCY10:NM_018417:exon17:c.A	NA	rs2071921	NA
chr1	1.68E+08	1.68E+08	ACAC	-	UTR3	TIPRL	NA	NA	NA	rs66543118	NA
chr1	1.69E+08	1.69E+08	A	T	UTR3	ATP1B1	NA	NA	NA	NA	NA
chr1	1.77E+08	1.77E+08	C	A	exonic	FAM5B	nonsynonymous SNV	FAM5B:NM_021165:exon6:c.C975A:p.D325E	NA	NA	B
chr1	1.8E+08	1.8E+08	T	G	exonic	TOR1AIP1	nonsynonymous SNV	M_001267578:exon1:c.T32G:p.V11G,TOR1AIP1:NM_015602:exon1:c.T	NA	NA	B
chr1	1.83E+08	1.83E+08	-	GT	UTR3	NMNAT2	NA	NA	NA	NA	NA
chr1	1.84E+08	1.84E+08	T	G	UTR5	RGL1	NA	NA	NA	NA	NA
chr1	1.84E+08	1.84E+08	T	G	UTR5	RGL1	NA	NA	NA	NA	NA
chr1	1.85E+08	1.85E+08	TT	-	UTR3	RNF2	NA	NA	NA	NA	NA
chr1	1.86E+08	1.86E+08	A	C	exonic	PRG4	nonsynonymous SNV	001127709:exon5:c.A970C:p.T324P,PRG4:NM_001127708:exon6:c.A1	=COSM1127223;OCCURENCE=1(prostat	NA	NA
chr1	1.86E+08	1.86E+08	T	C	exonic	PRG4	synonymous SNV	001127709:exon5:c.T1059C:p.T353T,PRG4:NM_001127708:exon6:c.T1	NA	NA	NA
chr1	1.86E+08	1.86E+08	T	C	exonic	PRG4	synonymous SNV	001127709:exon5:c.T1227C:p.T409T,PRG4:NM_001127708:exon6:c.T	NA	NA	NA
chr1	1.86E+08	1.86E+08	A	C	exonic	PRG4	nonsynonymous SNV	001127709:exon5:c.A1252C:p.T418P,PRG4:NM_001127708:exon6:c.A	NA	NA	NA
chr1	1.86E+08	1.86E+08	T	C	exonic	PRG4	synonymous SNV	001127709:exon5:c.T1275C:p.T425T,PRG4:NM_001127708:exon6:c.T	NA	NA	NA
chr1	1.86E+08	1.86E+08	A	C	exonic	PRG4	nonsynonymous SNV	001127709:exon5:c.A1384C:p.T462P,PRG4:NM_001127708:exon6:c.A	NA	NA	NA
chr1	1.86E+08	1.86E+08	T	C	exonic	PRG4	synonymous SNV	001127709:exon5:c.T1413C:p.T471T,PRG4:NM_001127708:exon6:c.T	NA	NA	NA
chr1	1.86E+08	1.86E+08	T	C	exonic	PRG4	synonymous SNV	001127709:exon5:c.T1485C:p.T495T,PRG4:NM_001127708:exon6:c.T	M392936;OCCURENCE=2(lung),2(endon	NA	NA
chr1	1.86E+08	1.86E+08	A	C	exonic	PRG4	nonsynonymous SNV	001127709:exon5:c.A1510C:p.T504P,PRG4:NM_001127708:exon6:c.A	=COSM1127220;OCCURENCE=1(prostat	NA	NA
chr1	1.86E+08	1.86E+08	G	C	exonic	PRG4	synonymous SNV	001127709:exon5:c.G1596C:p.T532T,PRG4:NM_001127708:exon6:c.G	NA	NA	NA
chr1	1.86E+08	1.86E+08	A	C	exonic	PRG4	nonsynonymous SNV	001127709:exon5:c.A2140C:p.T714P,PRG4:NM_001127708:exon6:c.A	NA	NA	NA
chr1	1.97E+08	1.97E+08	C	A	exonic	CFHR2	nonsynonymous SNV	CFHR2:NM_005666:exon2:c.C76A:p.P26T	NA	NA	D
chr1	1.97E+08	1.97E+08	C	G	UTR3	CFHR5	NA	NA	NA	NA	NA
chr1	1.97E+08	1.97E+08	-	A	UTR3	ZBTB41	NA	NA	NA	rs138564806	NA
chr1	2.01E+08	2.01E+08	TTC	-	UTR3	KIF14	NA	NA	NA	rs146486278	NA
chr1	2.01E+08	2.01E+08	C	T	exonic	IGFN1	synonymous SNV	IGFN1:NM_001164586:exon12:c.C4815T:p.D1605D	NA	NA	NA
chr1	2.01E+08	2.01E+08	T	C	exonic	IGFN1	nonsynonymous SNV	IGFN1:NM_001164586:exon12:c.T4823C:p.V1608A	NA	rs201505263	NA
chr1	2.01E+08	2.01E+08	T	G	exonic	IGFN1	nonsynonymous SNV	IGFN1:NM_001164586:exon12:c.T5099G:p.V1700G	NA	NA	NA
chr1	2.01E+08	2.01E+08	A	G	exonic	IGFN1	nonsynonymous SNV	IGFN1:NM_001164586:exon12:c.A5288G:p.D1763G	NA	rs74428176	NA
chr1	2.01E+08	2.01E+08	C	G	exonic	IGFN1	nonsynonymous SNV	IGFN1:NM_001164586:exon12:c.C5300G:p.A1767G	NA	rs1772845	NA
chr1	2.01E+08	2.01E+08	T	G	exonic	IGFN1	nonsynonymous SNV	IGFN1:NM_001164586:exon12:c.T5359G:p.W1787G	NA	rs1772844	NA
chr1	2.01E+08	2.01E+08	A	G	exonic	IGFN1	nonsynonymous SNV	IGFN1:NM_001164586:exon12:c.A6256G:p.T2086A	NA	rs1772839	NA
chr1	2.01E+08	2.01E+08	A	G	exonic	IGFN1	nonsynonymous SNV	IGFN1:NM_001164586:exon12:c.A6296G:p.E2099G	NA	rs200678915	NA
chr1	2.01E+08	2.01E+08	A	-	UTR3	TNNI1	NA	NA	NA	rs35058233	NA
chr1	2.02E+08	2.02E+08	-	A	UTR3	IPO9	NA	NA	NA	NA	NA
chr1	2.02E+08	2.02E+08	G	A	UTR3	IPO9	NA	NA	NA	rs116072433	NA
chr1	2.02E+08	2.02E+08	G	T	UTR3	IPO9	NA	NA	NA	rs72742088	NA
chr1	2.02E+08	2.02E+08	C	-	UTR3	IPO9	NA	NA	NA	rs112011484	NA
chr1	2.02E+08	2.02E+08	A	-	UTR3	ELF3	NA	NA	NA	rs11303270	NA
chr1	2.02E+08	2.02E+08	A	C	UTR3	GPR37L1	NA	NA	NA	NA	NA
chr1	2.02E+08	2.02E+08	TT	-	UTR3	GPR37L1	NA	NA	NA	NA	NA
chr1	2.02E+08	2.02E+08	T	G	UTR5	PPP1R12B	NA	NA	NA	NA	NA
chr1	2.04E+08	2.04E+08	T	-	exonic	KISS1	stoploss SNV	KISS1:NM_002256:exon3:c.417delA:p.X139W	NA	rs71745629	NA
chr1	2.04E+08	2.04E+08	A	T	ncRNA_exonic	LINC00628	NA	NA	NA	NA	NA
chr1	2.05E+08	2.05E+08	T	C	UTR5	CDK18	NA	NA	NA	rs77694338	NA
chr1	2.06E+08	2.06E+08	C	-	UTR3	RAB7L1	NA	NA	NA	rs34580135	NA
chr1	2.07E+08	2.07E+08	AC	-	UTR3	PIGR	NA	NA	NA	NA	NA
chr1	2.07E+08	2.07E+08	T	-	UTR3	PFKFB2	NA	NA	NA	NA	NA
chr1	2.08E+08	2.08E+08	T	C	exonic	CR1	synonymous SNV	000573:exon26:c.T4356C:p.C1452C,CR1:NM_000651:exon34:c.T5706C	NA	rs61734513	NA
chr1	2.08E+08	2.08E+08	T	C	UTR3	CR1	NA	NA	NA	rs4844385	NA
chr1	2.1E+08	2.1E+08	T	-	UTR5	MIR205HG	NA	NA	NA	NA	NA
chr1	2.11E+08	2.11E+08	T	C	UTR3	KCNH1	NA	NA	NA	rs12126648	NA
chr1	2.12E+08	2.12E+08	GTGT	-	UTR5	RD3	NA	NA	NA	NA	NA
chr1	2.13E+08	2.13E+08	T	C	UTR5	RPS6KC1	NA	NA	NA	rs4951476	NA
chr1	2.19E+08	2.19E+08	A	-	UTR3	RRP15	NA	NA	NA	rs201597960	NA
chr1	2.19E+08	2.19E+08	C	T	UTR3	RRP15	NA	NA	NA	NA	NA
chr1	2.22E+08	2.22E+08	-	T	UTR3	DUSP10	NA	NA	NA	NA	NA
chr1	2.23E+08	2.23E+08	C	T	exonic	HHIPL2	synonymous SNV	HHIPL2:NM_024746:exon1:c.G99A:p.L33L	NA	rs4846382	NA
chr1	2.24E+08	2.24E+08	T	C	UTR5	SUSD4	NA	NA	NA	NA	NA
chr1	2.24E+08	2.24E+08	T	C	UTR5	SUSD4	NA	NA	NA	NA	NA
chr1	2.24E+08	2.24E+08	T	C	UTR5	SUSD4	NA	NA	NA	NA	NA
chr1	2.24E+08	2.24E+08	G	T	exonic	CAPN8	unknown	UNKNOWN	NA	rs35539373	NA
chr1	2.25E+08	2.25E+08	-	T	UTR3	WDR26	NA	NA	NA	rs5781364	NA
chr1	2.26E+08	2.26E+08	A	-	UTR3	ENAH	NA	NA	NA	rs146121857	NA
chr1	2.26E+08	2.26E+08	TT	-	UTR3	ENAH	NA	NA	NA	rs200499155	NA
chr1	2.28E+08	2.28E+08	-	A	UTR5	CDC42BPA	NA	NA	NA	NA	NA
chr1	2.28E+08	2.28E+08	A	G	exonic	WNT9A	synonymous SNV	WNT9A:NM_003395:exon2:c.T153C:p.A51A	NA	rs3795768	NA

chr1	2.28E+08	2.28E+08	-	T	UTR3	IBA57	NA	NA	NA	NA	NA
chr1	2.29E+08	2.29E+08	T	C	exonic	OBSCN	nonsynonymous SNV	:p.C4450R,OBSCN:NM_052843:exon51:c.T13348C:p.C4450R,OBSCN:NI	NA	rs1188732	NA
chr1	2.3E+08	2.3E+08	T	G	exonic	ABCB10	nonsynonymous SNV	ABCB10:NM_012089:exon2:c.A605C:p.N202T	NA	NA	B
chr1	2.3E+08	2.3E+08	A	G	exonic	URB2	synonymous SNV	URB2:NM_014777:exon9:c.A4290G:p.R1430R	NA	rs7521953	NA
chr1	2.3E+08	2.3E+08	T	C	UTR3	URB2	NA	NA	NA	rs3820361	NA
chr1	2.31E+08	2.31E+08	A	G	exonic	ARV1	nonsynonymous SNV	ARV1:NM_022786:exon1:c.A43G:p.N15D	NA	NA	B
chr1	2.31E+08	2.31E+08	C	G	UTR3	TRIM67	NA	NA	NA	rs6541262	NA
chr1	2.31E+08	2.31E+08	G	T	UTR3	TRIM67	NA	NA	NA	rs530363	NA
chr1	2.31E+08	2.31E+08	T	-	UTR3	EXOC8	NA	NA	NA	rs35468792	NA
chr1	2.33E+08	2.33E+08	G	A	exonic	SIPA1L2	nonsynonymous SNV	SIPA1L2:NM_020808:exon7:c.C2504T:p.A835V	NA	rs142752379	P
chr1	2.37E+08	2.37E+08	C	T	exonic	LGALS8	synonymous SNV	NM_006499:exon5:c.C165T:p.S55S,LGALS8:NM_201543:exon5:c.C165	=COSM146831;OCCURENCE=1(stomac	rs1041936	NA
chr1	2.37E+08	2.37E+08	A	G	exonic	LGALS8	nonsynonymous SNV	M_006499:exon5:c.A166G:p.M56V,LGALS8:NM_201543:exon5:c.A166	=COSM146352;OCCURENCE=1(stomac	rs1041937	B
chr1	2.37E+08	2.37E+08	T	C	exonic	HEATR1	nonsynonymous SNV	HEATR1:NM_018072:exon15:c.A1819G:p.M607V	NA	rs2794763	D
chr1	2.37E+08	2.37E+08	A	G	exonic	MTR	synonymous SNV	MTR:NM_000254:exon29:c.A3144G:p.A1048A	NA	rs2229276	NA
chr1	2.37E+08	2.37E+08	G	T	UTR3	MTR	NA	NA	NA	NA	NA
chr1	2.38E+08	2.38E+08	T	-	UTR3	RYR2	NA	NA	NA	NA	NA
chr1	2.4E+08	2.4E+08	GGC	-	exonic	FMN2	nonframeshift deletion	FMN2:NM_020066:exon1:c.160_162del:p.54_54del	4564;OCCURENCE=1(pancreas),1(skin)	rs140531536	NA
chr1	2.4E+08	2.4E+08	C	A	exonic	FMN2	synonymous SNV	FMN2:NM_020066:exon1:c.C1413A:p.A471A	NA	rs10926124	NA
chr1	2.4E+08	2.4E+08	G	A	exonic	FMN2	synonymous SNV	FMN2:NM_020066:exon5:c.G2820A:p.A940A	NA	rs145628188	NA
chr1	2.43E+08	2.43E+08	A	G	ncRNA_exonic	LOC731275	NA	NA	NA	rs148698597	NA
chr1	2.45E+08	2.45E+08	-	GTA	UTR3	C1orf101	NA	NA	NA	rs201380603	NA
chr1	2.48E+08	2.48E+08	-	A	UTR3	TRIM58	NA	NA	NA	rs34769974	NA
chr1	2.48E+08	2.48E+08	G	A	exonic	OR2T8	synonymous SNV	OR2T8:NM_001005522:exon1:c.G435A:p.S145S	NA	rs192581532	NA
chr1	2.48E+08	2.48E+08	G	C	exonic	OR2T8	nonsynonymous SNV	OR2T8:NM_001005522:exon1:c.G437C:p.C146S	NA	rs146023264	B
chr1	2.48E+08	2.48E+08	T	C	exonic	OR2T8	synonymous SNV	OR2T8:NM_001005522:exon1:c.T438C:p.C146C	NA	rs142841709	NA
chr1	2.49E+08	2.49E+08	C	T	exonic	OR2T3	synonymous SNV	OR2T3:NM_001005495:exon1:c.C711T:p.A237A	NA	rs77686526	NA
chr1	2.49E+08	2.49E+08	A	G	exonic	OR2T3	nonsynonymous SNV	OR2T3:NM_001005495:exon1:c.A829G:p.M277V	NA	rs74498773	P
chr1	2.49E+08	2.49E+08	A	T	exonic	OR2T27	nonsynonymous SNV	OR2T27:NM_001001824:exon1:c.T401A:p.V134D	NA	rs1782240	B
chr2	905581	905581	C	T	intergenic	822(dist=41469),SNTG2(dist=	NA	NA	NA	rs113738897	NA
chr2	905693	905693	T	G	intergenic	822(dist=41581),SNTG2(dist=	NA	NA	NA	rs74861779	NA
chr2	1636938	1636938	-	T	UTR3	PXDN	NA	NA	NA	NA	NA
chr2	9546208	9546208	G	A	UTR3	ITGB1BP1	NA	NA	NA	rs112888538	NA
chr2	9546440	9546440	T	G	UTR3	ITGB1BP1	NA	NA	NA	rs13392033	NA
chr2	11782595	11782595	C	A	UTR3	GREB1	NA	NA	NA	NA	NA
chr2	24253007	24253007	-	A	UTR3	C2orf44	NA	NA	NA	NA	NA
chr2	24533411	24533411	G	T	exonic	ITSN2	nonsynonymous SNV	3A:p.P168Q,ITSN2:NM_019595:exon6:c.C503A:p.P168Q,ITSN2:NM_1	NA	NA	NA
chr2	24991386	24991388	AAA	-	UTR3	NCOA1	NA	NA	NA	NA	NA
chr2	25379159	25379159	G	T	UTR3	EFR3B	NA	NA	NA	NA	NA
chr2	26455127	26455127	G	A	exonic	HADHA	synonymous SNV	HADHA:NM_000182:exon6:c.C474T:p.Y158Y	NA	rs11552518	NA
chr2	26680509	26680509	A	T	UTR3	OTOF	NA	NA	NA	NA	NA
chr2	27477627	27477627	-	ACAC	UTR3	SLC30A3	NA	NA	NA	NA	NA
chr2	27804714	27804714	T	C	exonic	C2orf16	nonsynonymous SNV	C2orf16:NM_032266:exon1:c.T5275C:p.C1759R	NA	NA	NA
chr2	27804738	27804738	T	C	exonic	C2orf16	nonsynonymous SNV	C2orf16:NM_032266:exon1:c.T5299C:p.C1767R	NA	rs199708546	NA
chr2	29023081	29023081	C	T	UTR3	PPP1CB	NA	NA	NA	NA	NA
chr2	29023088	29023088	C	T	UTR3	PPP1CB	NA	NA	NA	NA	NA
chr2	29023092	29023092	C	T	UTR3	PPP1CB	NA	NA	NA	rs201829322	NA
chr2	29170759	29170759	G	A	UTR3	WDR43	NA	NA	NA	rs13397658	NA
chr2	29448423	29448423	T	G	exonic	ALK	nonsynonymous SNV	ALK:NM_004304:exon19:c.A3076C:p.T1026P	NA	NA	B
chr2	30670215	30670215	A	C	UTR5	LCLAT1	NA	NA	NA	NA	NA
chr2	30864771	30864774	TTTC	-	UTR3	LCLAT1	NA	NA	NA	NA	NA
chr2	30865285	30865286	TA	-	UTR3	LCLAT1	NA	NA	NA	NA	NA
chr2	32822957	32822957	G	A	exonic	BIRC6	synonymous SNV	BIRC6:NM_016252:exon69:c.G13752A:p.T4584T	148834,COSM148835;OCCURENCE=1(	rs2710625	NA
chr2	33787988	33787988	A	T	UTR3	RASGRP3	NA	NA	NA	NA	NA
chr2	33787989	33787989	C	G	UTR3	RASGRP3	NA	NA	NA	NA	NA
chr2	33787990	33787990	T	A	UTR3	RASGRP3	NA	NA	NA	NA	NA
chr2	33951323	33951323	G	T	ncRNA_exonic	MYADML	NA	NA	NA	NA	NA
chr2	36782886	36782886	G	A	exonic	FEZ2	nonsynonymous SNV	_005102:exon7:c.C985T:p.R329C,FEZ2:NM_001042548:exon8:c.C1066	NA	rs848642	NA
chr2	36810586	36810586	T	C	exonic	FEZ2	synonymous SNV	_001042548:exon3:c.A402G:p.T134T,FEZ2:NM_005102:exon3:c.A402	NA	rs14291	NA
chr2	37479024	37479024	A	-	UTR3	PRKD3	NA	NA	NA	NA	NA
chr2	37871117	37871120	TTTT	-	UTR3	CDC42EP3	NA	NA	NA	rs200620123	NA
chr2	42515437	42515437	A	G	exonic	EML4	nonsynonymous SNV	1145076:exon10:c.A1019G:p.K340R,EML4:NM_019063:exon11:c.A11	NA	rs28651764	B
chr2	42557760	42557760	T	-	UTR3	EML4	NA	NA	NA	NA	NA
chr2	43452183	43452183	A	G	exonic	ZFP36L2	synonymous SNV	ZFP36L2:NM_006887:exon2:c.T760C:p.L254L	NA	rs7933	NA
chr2	45172485	45172485	G	A	UTR3	SIX3	NA	NA	NA	rs60528008	NA
chr2	46707884	46707884	A	G	exonic	TMEM247	nonsynonymous SNV	TMEM247:NM_001145051:exon2:c.A458G:p.Q153R	NA	rs201742486	NA
chr2	46844219	46844219	-	G	UTR5	PIGF	NA	NA	NA	rs5830914	NA

chr2	46852206	46852206	G	T	UTR3	CRIPT	NA	NA	NA	NA	NA
chr2	48742355	48742355	-	TCTCTCTCTC	UTR3	PPP1R21	NA	NA	NA	NA	NA
chr2	50147836	50147841	GTGTGT	-	UTR3	NRXN1	NA	NA	NA	NA	NA
chr2	51259649	51259660	AGAGAGAG	-	UTR5	NRXN1	NA	NA	NA	NA	NA
chr2	61351187	61351187	T	C	UTR3	KIAA1841	NA	NA	NA	rs114498878	NA
chr2	61351188	61351188	T	C	UTR3	KIAA1841	NA	NA	NA	rs114317312	NA
chr2	61351189	61351189	T	A	UTR3	KIAA1841	NA	NA	NA	rs114015142	NA
chr2	85284303	85284303	A	-	UTR3	KCMF1	NA	NA	NA	NA	NA
chr2	85531102	85531102	T	C	exonic	TCF7L1	nonsynonymous SNV	TCF7L1:NM_031283:exon6:c.T743C:p.L248P	NA	NA	D
chr2	85531118	85531118	A	C	exonic	TCF7L1	synonymous SNV	TCF7L1:NM_031283:exon6:c.A759C:p.P253P	NA	NA	NA
chr2	85549667	85549667	G	C	UTR3	TGOLN2	NA	NA	NA	NA	NA
chr2	85554315	85554315	A	G	exonic	TGOLN2	synonymous SNV	C:p.N180N,TGOLN2:NM_001206844:exon2:c.T540C:p.N180N,TGOLN2	NA	NA	NA
chr2	87002452	87002453	TT	-	UTR3	RMND5A	NA	NA	NA	NA	NA
chr2	89082373	89082373	G	A	ncRNA_exonic	ANKRD36BP2	NA	NA	NA	rs140000456	NA
chr2	89082379	89082379	G	A	ncRNA_exonic	ANKRD36BP2	NA	NA	NA	rs143191250	NA
chr2	89082381	89082381	C	T	ncRNA_exonic	ANKRD36BP2	NA	NA	NA	rs140822233	NA
chr2	89082388	89082388	G	T	ncRNA_exonic	ANKRD36BP2	NA	NA	NA	rs142861467	NA
chr2	89104333	89104333	C	T	ncRNA_exonic	ANKRD36BP2	NA	NA	NA	rs148665042	NA
chr2	89104362	89104362	C	T	ncRNA_exonic	ANKRD36BP2	NA	NA	NA	rs62158694	NA
chr2	91963374	91963374	A	G	ncRNA_exonic	GGT8P	NA	NA	NA	NA	NA
chr2	91963419	91963419	A	G	ncRNA_exonic	GGT8P	NA	NA	NA	NA	NA
chr2	91963455	91963455	T	C	ncRNA_exonic	GGT8P	NA	NA	NA	NA	NA
chr2	91963687	91963687	G	A	ncRNA_exonic	GGT8P	NA	NA	NA	rs201619433	NA
chr2	91968630	91968630	T	C	ncRNA_exonic	GGT8P	NA	NA	NA	NA	NA
chr2	91969892	91969892	C	T	ncRNA_exonic	GGT8P	NA	NA	NA	rs5993271	NA
chr2	91969935	91969935	T	C	ncRNA_exonic	GGT8P	NA	NA	NA	rs4059987	NA
chr2	92129578	92129578	G	C	ncRNA_exonic	ACTR3BP2	NA	NA	NA	rs4125437	NA
chr2	92129614	92129614	G	T	ncRNA_exonic	ACTR3BP2	NA	NA	NA	NA	NA
chr2	95537245	95537245	C	G	ncRNA_UTR5	TEKT4	NA	NA	NA	rs4854234	NA
chr2	97779341	97779341	T	G	UTR5	ANKRD36	NA	NA	NA	rs3866777	NA
chr2	97911395	97911395	A	G	exonic	ANKRD36	nonsynonymous SNV	ANKRD36:NM_001164315:exon71:c.A5071G:p.K1691E	NA	rs2595354	NA
chr2	98127671	98127671	A	C	exonic	ANKRD36B	nonsynonymous SNV	ANKRD36B:NM_025190:exon39:c.T3650G:p.M1217R	NA	rs76066788	NA
chr2	98128228	98128228	G	A	exonic	ANKRD36B	synonymous SNV	ANKRD36B:NM_025190:exon39:c.C3093T:p.S1031S	ID=COSN395741;OCCURENCE=1(lung)	rs201190268	NA
chr2	98129733	98129733	A	C	exonic	ANKRD36B	stopgain SNV	ANKRD36B:NM_025190:exon38:c.T2715G:p.Y905X	NA	rs145533718	NA
chr2	1.01E+08	1.01E+08	AAAAATTA	-	UTR3	LONRF2	NA	NA	NA	NA	NA
chr2	1.01E+08	1.01E+08	TCTACTGAA	-	UTR3	LONRF2	NA	NA	NA	NA	NA
chr2	1.03E+08	1.03E+08	G	A	UTR3	MAP4K4	NA	NA	NA	NA	NA
chr2	1.07E+08	1.07E+08	AAC	-	UTR3	ST6GAL2	NA	NA	NA	rs140467729	NA
chr2	1.07E+08	1.07E+08	-	A	UTR3	ST6GAL2	NA	NA	NA	NA	NA
chr2	1.09E+08	1.09E+08	T	C	exonic	SULT1C3	nonsynonymous SNV	SULT1C3:NM_001008743:exon5:c.T581C:p.M194T	NA	rs6722745	B
chr2	1.09E+08	1.09E+08	G	A	exonic	CCDC138	nonsynonymous SNV	CCDC138:NM_144978:exon4:c.G344A:p.R115K	=COSM148924;OCCURENCE=1(stomac	rs6740879	B
chr2	1.13E+08	1.13E+08	G	T	UTR3	ZC3H6	NA	NA	NA	NA	NA
chr2	1.14E+08	1.14E+08	T	C	exonic	FOXD4L1	nonsynonymous SNV	FOXD4L1:NM_012184:exon1:c.T422C:p.F141S	NA	NA	D
chr2	1.14E+08	1.14E+08	A	C	exonic	FOXD4L1	nonsynonymous SNV	FOXD4L1:NM_012184:exon1:c.A431C:p.Y144S	NA	NA	D
chr2	1.14E+08	1.14E+08	A	C	ncRNA_exonic	FAM138B	NA	NA	NA	rs141773324	NA
chr2	1.14E+08	1.14E+08	T	C	UTR5	RABL2A	NA	NA	NA	rs1667097	NA
chr2	1.19E+08	1.19E+08	A	-	UTR3	CCDC93	NA	NA	NA	NA	NA
chr2	1.19E+08	1.19E+08	A	-	UTR3	CCDC93	NA	NA	NA	rs3832171	NA
chr2	1.22E+08	1.22E+08	A	-	UTR3	TFCP2L1	NA	NA	NA	rs36110149	NA
chr2	1.22E+08	1.22E+08	-	TTG	UTR3	CLASP1	NA	NA	NA	rs113839646	NA
chr2	1.28E+08	1.28E+08	C	T	UTR3	CYP27C1	NA	NA	NA	rs35887875	NA
chr2	1.28E+08	1.28E+08	T	-	UTR3	MAP3K2	NA	NA	NA	NA	NA
chr2	1.29E+08	1.29E+08	A	C	UTR3	UGGT1	NA	NA	NA	NA	NA
chr2	1.31E+08	1.31E+08	-	G	ncRNA_exonic	LOC440905	NA	NA	NA	rs34985062	NA
chr2	1.31E+08	1.31E+08	-	CT	ncRNA_exonic	LOC440905	NA	NA	NA	rs3058424	NA
chr2	1.31E+08	1.31E+08	G	A	exonic	POTEF	synonymous SNV	POTEF:NM_001099771:exon17:c.C2172T:p.D724D	NA	rs201958629	NA
chr2	1.31E+08	1.31E+08	T	C	exonic	CCDC74B	nonsynonymous SNV	CCDC74B:NM_207310:exon3:c.A446G:p.H149R	NA	rs138331089	NA
chr2	1.33E+08	1.33E+08	G	A	exonic	C2orf27B	synonymous SNV	C2orf27B:NM_214461:exon3:c.C189T:p.A63A	NA	rs71430413	NA
chr2	1.33E+08	1.33E+08	G	A	ncRNA_exonic	ANKRD30BL	NA	NA	NA	rs112315337	NA
chr2	1.33E+08	1.33E+08	G	A	ncRNA_exonic	ANKRD30BL	NA	NA	NA	rs111401445	NA
chr2	1.33E+08	1.33E+08	G	C	ncRNA_exonic	ANKRD30BL	NA	NA	NA	rs112494139	NA
chr2	1.33E+08	1.33E+08	G	A	ncRNA_exonic	ANKRD30BL	NA	NA	NA	rs148180857	NA
chr2	1.45E+08	1.45E+08	T	-	intergenic	P15(dist=176565),GTDC1(dist	NA	NA	NA	rs61418485	NA
chr2	1.52E+08	1.52E+08	-	T	UTR5	RBM43	NA	NA	NA	rs113906824	NA
chr2	1.52E+08	1.52E+08	G	A	exonic	NMI	nonsynonymous SNV	NMI:NM_004688:exon2:c.C47T:p.S16L	NA	rs1048135	B
chr2	1.53E+08	1.53E+08	C	T	UTR3	CACNB4	NA	NA	NA	rs7597215	NA

chr2	1.53E+08	1.53E+08	A	-	UTR3	STAM2	NA	NA	NA	rs58778946	NA
chr2	1.61E+08	1.61E+08	A	G	exonic	LY75,LY75-CD302	nonsynonymous SNV	C:p.Y1391H,LY75-CD302:NM_001198760:exon30:c.T4171C:p.Y1391H,	D=COSM148967;OCCURENCE=1(stomac	rs2059696	D
chr2	1.61E+08	1.61E+08	-	T	UTR3	RBMS1	NA	NA	NA	rs34176400	NA
chr2	1.67E+08	1.67E+08	A	G	exonic	SCN9A	synonymous SNV	SCN9A:NM_002977:exon10:c.T1119C:p.A373A	NA	rs13414203	NA
chr2	1.7E+08	1.7E+08	-	A	UTR3	LRP2	NA	NA	NA	NA	NA
chr2	1.7E+08	1.7E+08	T	C	UTR3	BBS5	NA	NA	NA	rs7589199	NA
chr2	1.74E+08	1.74E+08	-	A	ncRNA_UTR3	ZAK	NA	NA	NA	NA	NA
chr2	1.77E+08	1.77E+08	G	A	UTR3	KIAA1715	NA	NA	NA	NA	NA
chr2	1.77E+08	1.77E+08	C	T	UTR3	HOXD9	NA	NA	NA	NA	NA
chr2	1.78E+08	1.78E+08	TT	-	UTR3	PDE11A	NA	NA	NA	NA	NA
chr2	1.78E+08	1.78E+08	C	T	UTR3	PDE11A	NA	NA	NA	NA	NA
chr2	1.78E+08	1.78E+08	AGTCTGG	-	UTR3	PDE11A	NA	NA	NA	NA	NA
chr2	1.79E+08	1.79E+08	C	-	splicing	>-,NM_001139518:exon8:c.5	NA	NA	NA	NA	NA
chr2	1.83E+08	1.83E+08	A	C	UTR3	PDE1A	NA	NA	NA	rs1999	NA
chr2	1.89E+08	1.89E+08	A	-	UTR3	GULP1	NA	NA	NA	rs5837097	NA
chr2	1.92E+08	1.92E+08	C	A	UTR5	NAB1	NA	NA	NA	rs2293765	NA
chr2	1.97E+08	1.97E+08	T	-	UTR3	SLC39A10	NA	NA	NA	rs66775415	NA
chr2	1.98E+08	1.98E+08	G	A	UTR3	PGAP1	NA	NA	NA	NA	NA
chr2	2.02E+08	2.02E+08	-	TTAA	UTR3	PPIL3	NA	NA	NA	rs145843683	NA
chr2	2.02E+08	2.02E+08	TT	-	UTR3	FAM126B	NA	NA	NA	NA	NA
chr2	2.04E+08	2.04E+08	C	T	UTR3	FAM117B	NA	NA	NA	NA	NA
chr2	2.04E+08	2.04E+08	TT	-	UTR3	FAM117B	NA	NA	NA	NA	NA
chr2	2.04E+08	2.04E+08	T	C	UTR3	WDR12	NA	NA	NA	rs6722332	NA
chr2	2.04E+08	2.04E+08	A	C	UTR3	RAPH1	NA	NA	NA	rs201945070	NA
chr2	2.07E+08	2.07E+08	A	-	UTR3	INO80D	NA	NA	NA	rs11306784	NA
chr2	2.07E+08	2.07E+08	AA	-	UTR3	INO80D	NA	NA	NA	rs143392413	NA
chr2	2.09E+08	2.09E+08	AG	-	UTR3	FZD5	NA	NA	NA	rs201847302	NA
chr2	2.09E+08	2.09E+08	T	-	UTR3	PLEKHM3	NA	NA	NA	NA	NA
chr2	2.11E+08	2.11E+08	CACA	-	UTR3	MAP2	NA	NA	NA	rs112861196	NA
chr2	2.18E+08	2.18E+08	-	CTGT	ncRNA_exonic	DIRC3	NA	NA	NA	rs112187824	NA
chr2	2.19E+08	2.19E+08	-	C	UTR3	SLC11A1	NA	NA	NA	NA	NA
chr2	2.2E+08	2.2E+08	G	T	UTR3	FAM134A	NA	NA	NA	NA	NA
chr2	2.2E+08	2.2E+08	G	T	UTR3	FAM134A	NA	NA	NA	rs146041546	NA
chr2	2.24E+08	2.24E+08	C	A	UTR3	ACSL3	NA	NA	NA	rs1071621	NA
chr2	2.25E+08	2.25E+08	C	A	UTR3	WDFY1	NA	NA	NA	NA	NA
chr2	2.33E+08	2.33E+08	T	C	UTR5	PTMA	NA	NA	NA	rs12415	NA
chr2	2.33E+08	2.33E+08	C	T	UTR5	PTMA	NA	NA	NA	rs8868	NA
chr2	2.33E+08	2.33E+08	T	C	UTR3	DIS3L2	NA	NA	NA	NA	NA
chr2	2.34E+08	2.34E+08	A	G	exonic	EFHD1	nonsynonymous SNV	_001243252:exon3:c.A269G:p.K90R,EFHD1:NM_025202:exon3:c.A557	NA	rs11550699	B
chr2	2.34E+08	2.34E+08	CAGCAGCTC	-	exonic	GIGYF2	nonframeshift deletion	n27:c.3613_3633del:p.1205_1211del,GIGYF2:NM_001103147:exon29:	NA	NA	NA
chr2	2.41E+08	2.41E+08	C	T	exonic	PRR21	synonymous SNV	PRR21:NM_001080835:exon1:c.G768A:p.S256S	NA	NA	NA
chr2	2.41E+08	2.41E+08	G	C	exonic	PRR21	nonsynonymous SNV	PRR21:NM_001080835:exon1:c.C271G:p.P91A	NA	rs112308001	P
chr2	2.41E+08	2.41E+08	A	G	exonic	PRR21	synonymous SNV	PRR21:NM_001080835:exon1:c.T237C:p.H79H	NA	rs117846189	NA
chr2	2.41E+08	2.41E+08	G	A	exonic	PRR21	nonsynonymous SNV	PRR21:NM_001080835:exon1:c.C209T:p.T70I	NA	rs150205960	P
chr2	2.42E+08	2.42E+08	T	C	exonic	FARP2	synonymous SNV	FARP2:NM_014808:exon23:c.T2586C:p.P862P	NA	NA	NA
chr2	2.42E+08	2.42E+08	A	C	exonic	FARP2	synonymous SNV	FARP2:NM_014808:exon23:c.A2623C:p.R875R	NA	NA	NA
chr2	2.43E+08	2.43E+08	T	A	UTR3	BOK	NA	NA	NA	rs12619342	NA
chr2	2.43E+08	2.43E+08	C	A	UTR3	D2HGDH	NA	NA	NA	NA	NA
chr2	2.43E+08	2.43E+08	G	A	exonic	CXXC11	nonsynonymous SNV	CXXC11:NM_173821:exon2:c.G302A:p.G101E	NA	rs7420371	D
chr3	3190646	3190646	-	TCTCTAAAC	UTR3	TRNT1	NA	NA	NA	rs141431257	NA
chr3	3214495	3214495	C	A	exonic	CRBN	nonsynonymous SNV	_001173482:exon4:c.G489T:p.R163S,CRBN:NM_016302:exon4:c.G492	NA	NA	D
chr3	11599950	11599950	T	-	UTR3	VGLL4	NA	NA	NA	NA	NA
chr3	14105938	14105938	T	A	ncRNA_exonic	TPRXL	NA	NA	NA	NA	NA
chr3	14105955	14105955	T	C	ncRNA_exonic	TPRXL	NA	NA	NA	NA	NA
chr3	14105996	14105996	A	G	ncRNA_exonic	TPRXL	NA	NA	NA	NA	NA
chr3	14106174	14106174	T	C	ncRNA_exonic	TPRXL	NA	NA	NA	NA	NA
chr3	14200047	14200047	C	A	exonic	XPC	stopgain SNV	001145769:exon9:c.G1225T:p.E409X,XPC:NM_004628:exon9:c.G1336	NA	NA	NA
chr3	15090909	15090914	CCTTAG	-	UTR3	MRPS25	NA	NA	NA	rs148431054	NA
chr3	15451978	15451978	T	C	UTR3	METTL6	NA	NA	NA	rs6805196	NA
chr3	15469025	15469025	A	C	UTR5	METTL6	NA	NA	NA	NA	NA
chr3	15482997	15482999	TTT	-	UTR3	EAF1	NA	NA	NA	NA	NA
chr3	21448176	21448180	AAAAA	-	ncRNA_exonic	VENTXP7	NA	NA	NA	NA	NA
chr3	28364846	28364846	T	-	UTR3	AZI2	NA	NA	NA	rs200293551	NA
chr3	30045699	30045699	-	T	UTR3	RBMS3	NA	NA	NA	NA	NA
chr3	38888227	38888227	C	T	exonic	SCN11A	synonymous SNV	SCN11A:NM_014139:exon26:c.G5334A:p.L1778L	NA	rs4640498	NA
chr3	39137879	39137879	-	A	UTR3	WDR48	NA	NA	NA	rs11413912	NA

chr3	40498933	40498933	A	G	UTR5	RPL14	NA	NA	NA	rs4973904	NA
chr3	41281477	41281477	C	T	UTR3	CTNNB1	NA	NA	NA	rs201175238	NA
chr3	42825568	42825568	A	-	UTR3	HIGD1A	NA	NA	NA	rs5848628	NA
chr3	44607013	44607013	T	C	exonic	ZKSCAN7	nonsynonymous SNV	M_018651:exon3:c.T458C:p.F153S,ZKSCAN7:NM_025169:exon3:c.T45	NA	rs13081859	B
chr3	44964485	44964485	C	G	UTR3	ZDHHC3	NA	NA	NA	rs1979946	NA
chr3	45960561	45960561	-	AA	UTR3	FYCO1	NA	NA	NA	rs145514188	NA
chr3	47050997	47050997	T	C	UTR3	NBEAL2	NA	NA	NA	NA	NA
chr3	47050999	47050999	G	C	UTR3	NBEAL2	NA	NA	NA	NA	NA
chr3	48451952	48451952	A	C	exonic	PLXNB1	nonsynonymous SNV	1130082:exon30:c.T5432G:p.V1811G,PLXNB1:NM_002673:exon30:c.T5	=COSM328123;OCCURENCE=1(pancrea	NA	P
chr3	49044650	49044650	A	G	UTR5	WDR6	NA	NA	NA	NA	NA
chr3	49572947	49572947	C	A	UTR3	DAG1	NA	NA	NA	NA	NA
chr3	49694888	49694888	T	C	exonic	BSN	synonymous SNV	BSN:NM_003458:exon5:c.T7899C:p.L2633L	NA	NA	NA
chr3	49726070	49726070	G	A	exonic	MST1	nonsynonymous SNV	MST1:NM_020998:exon1:c.C55T:p.P19S	NA	rs62262686	D
chr3	51738256	51738256	A	G	UTR3	TEX264	NA	NA	NA	rs4067	NA
chr3	52015205	52015205	C	A	ncRNA_UTR3	ABHD14A	NA	NA	NA	NA	NA
chr3	55502167	55502167	A	-	UTR3	WNT5A	NA	NA	NA	NA	NA
chr3	64084148	64084148	A	-	ncRNA_exonic	PRICKLE2-AS1	NA	NA	NA	rs35195442	NA
chr3	64672461	64672461	T	G	exonic	ADAMTS9	nonsynonymous SNV	ADAMTS9:NM_182920:exon2:c.A299C:p.Y100S	NA	NA	D
chr3	67049615	67049615	G	T	exonic	KBTBD8	nonsynonymous SNV	KBTBD8:NM_032505:exon2:c.G227T:p.R76I	NA	NA	D
chr3	71007273	71007273	T	-	UTR3	FOXP1	NA	NA	NA	NA	NA
chr3	71821711	71821711	A	-	UTR3	PROK2	NA	NA	NA	rs71713597	NA
chr3	75716090	75716090	T	C	ncRNA_exonic	FLJ20518	NA	NA	NA	rs144150259	NA
chr3	75786748	75786748	C	T	exonic	ZNF717	nonsynonymous SNV	ZNF717:NM_001128223:exon5:c.G2026A:p.D676N	NA	rs112332186	NA
chr3	75787015	75787015	C	T	exonic	ZNF717	nonsynonymous SNV	ZNF717:NM_001128223:exon5:c.G1759A:p.G587S	COSM1173002;OCCURENCE=1(oesopha	rs111576331	NA
chr3	75787043	75787043	G	A	exonic	ZNF717	synonymous SNV	ZNF717:NM_001128223:exon5:c.C1731T:p.F577F	NA	rs184833776	NA
chr3	75787116	75787116	T	G	exonic	ZNF717	nonsynonymous SNV	ZNF717:NM_001128223:exon5:c.A1658C:p.H553P	NA	rs142824987	NA
chr3	75787620	75787620	T	C	exonic	ZNF717	nonsynonymous SNV	ZNF717:NM_001128223:exon5:c.A1154G:p.H385R	NA	rs145606249	NA
chr3	75790852	75790852	G	A	exonic	ZNF717	synonymous SNV	ZNF717:NM_001128223:exon3:c.C93T:p.F31F	NA	rs201689840	NA
chr3	75790864	75790864	T	C	exonic	ZNF717	synonymous SNV	ZNF717:NM_001128223:exon3:c.A81G:p.V27V	NA	rs75271400	NA
chr3	75790870	75790870	C	T	exonic	ZNF717	synonymous SNV	ZNF717:NM_001128223:exon3:c.G75A:p.E25E	NA	rs78652530	NA
chr3	98515881	98515882	AA	-	UTR3	DCBLD2	NA	NA	NA	rs56369137	NA
chr3	1.05E+08	1.05E+08	G	T	exonic	CBLB	nonsynonymous SNV	CBLB:NM_170662:exon12:c.C1618A:p.Q540K	NA	NA	D
chr3	1.08E+08	1.08E+08	AA	-	UTR3	BBX	NA	NA	NA	rs60360146	NA
chr3	1.08E+08	1.08E+08	A	T	UTR3	BBX	NA	NA	NA	rs201477170	NA
chr3	1.12E+08	1.12E+08	C	T	UTR5	TAGLN3	NA	NA	NA	NA	NA
chr3	1.2E+08	1.2E+08	A	C	exonic	GPR156	nonsynonymous SNV	001168271:exon9:c.T1643G:p.V548G,GPR156:NM_153002:exon9:c.T1	NA	NA	D
chr3	1.2E+08	1.2E+08	A	C	exonic	GPR156	nonsynonymous SNV	001168271:exon9:c.T1610G:p.V537G,GPR156:NM_153002:exon9:c.T1	NA	NA	D
chr3	1.23E+08	1.23E+08	T	-	ncRNA_UTR3	MYLK	NA	NA	NA	rs35930843	NA
chr3	1.26E+08	1.26E+08	-	T	UTR3	ZXDC	NA	NA	NA	NA	NA
chr3	1.26E+08	1.26E+08	T	A	UTR3	ZXDC	NA	NA	NA	rs79064850	NA
chr3	1.29E+08	1.29E+08	T	G	UTR3	CNBP	NA	NA	NA	NA	NA
chr3	1.29E+08	1.29E+08	T	G	exonic	PLXND1	nonsynonymous SNV	PLXND1:NM_015103:exon31:c.A5108C:p.Y1703S	NA	NA	D
chr3	1.34E+08	1.34E+08	C	G	UTR5	CEP63	NA	NA	NA	NA	NA
chr3	1.39E+08	1.39E+08	C	T	UTR3	PRR23C	NA	NA	NA	rs74940990	NA
chr3	1.41E+08	1.41E+08	-	T	UTR3	RNF7	NA	NA	NA	NA	NA
chr3	1.42E+08	1.42E+08	C	T	UTR3	GK5	NA	NA	NA	NA	NA
chr3	1.42E+08	1.42E+08	C	T	UTR3	GK5	NA	NA	NA	NA	NA
chr3	1.42E+08	1.42E+08	C	T	UTR3	GK5	NA	NA	NA	rs74651712	NA
chr3	1.42E+08	1.42E+08	G	A	exonic	TRPC1	nonsynonymous SNV	M_001251845:exon1:c.G40A:p.A14T,TRPC1:NM_003304:exon1:c.G40	NA	rs78975236	NA
chr3	1.44E+08	1.44E+08	-	AAAATGTTA	UTR3	C3orf58	NA	NA	NA	rs11276482	NA
chr3	1.47E+08	1.47E+08	T	-	UTR5	ZIC1	NA	NA	NA	rs57723801	NA
chr3	1.5E+08	1.5E+08	G	T	UTR3	TSC22D2	NA	NA	NA	rs201497156	NA
chr3	1.52E+08	1.52E+08	T	-	UTR3	MBNL1	NA	NA	NA	rs142162205	NA
chr3	1.53E+08	1.53E+08	C	T	UTR3	RAP2B	NA	NA	NA	NA	NA
chr3	1.54E+08	1.54E+08	C	-	UTR3	ARHGEF26	NA	NA	NA	NA	NA
chr3	1.54E+08	1.54E+08	TA	-	UTR3	DHX36	NA	NA	NA	rs71790534	NA
chr3	1.58E+08	1.58E+08	A	G	UTR3	SHOX2	NA	NA	NA	rs6775325	NA
chr3	1.67E+08	1.67E+08	A	C	exonic	SERPINI2	nonsynonymous SNV	NM_006217:exon2:c.T16G:p.L6V,SERPINI2:NM_001012303:exon3:c.T4	NA	rs17246389	P
chr3	1.7E+08	1.7E+08	C	G	UTR3	PHC3	NA	NA	NA	rs6444896	NA
chr3	1.7E+08	1.7E+08	C	T	UTR3	CLDN11	NA	NA	NA	NA	NA
chr3	1.8E+08	1.8E+08	T	G	UTR3	CCDC39	NA	NA	NA	NA	NA
chr3	1.81E+08	1.81E+08	G	A	ncRNA_exonic	SOX2-OT	NA	NA	NA	rs140276576	NA
chr3	1.81E+08	1.81E+08	G	A	ncRNA_exonic	SOX2-OT	NA	NA	NA	NA	NA
chr3	1.81E+08	1.81E+08	G	A	ncRNA_exonic	SOX2-OT	NA	NA	NA	NA	NA
chr3	1.81E+08	1.81E+08	G	A	ncRNA_exonic	SOX2-OT	NA	NA	NA	NA	NA
chr3	1.81E+08	1.81E+08	G	A	ncRNA_exonic	SOX2-OT	NA	NA	NA	NA	NA

chr3	1.81E+08	1.81E+08	G	A	ncRNA_exonic	SOX2-OT	NA	NA	NA	NA	NA
chr3	1.81E+08	1.81E+08	G	A	ncRNA_exonic	SOX2-OT	NA	NA	NA	NA	NA
chr3	1.81E+08	1.81E+08	G	A	ncRNA_exonic	SOX2-OT	NA	NA	NA	NA	NA
chr3	1.81E+08	1.81E+08	GCACACAC	-	ncRNA_exonic	SOX2-OT	NA	NA	NA	rs201706406	NA
chr3	1.84E+08	1.84E+08	GTGTGT	-	UTR3	EPHB3	NA	NA	NA	rs35514470	NA
chr3	1.85E+08	1.85E+08	-	T	UTR3	TMEM41A	NA	NA	NA	NA	NA
chr3	1.88E+08	1.88E+08	AG	-	ncRNA_exonic	FLJ42393	NA	NA	NA	rs10616335	NA
chr3	1.89E+08	1.89E+08	-	A	UTR3	TPRG1	NA	NA	NA	rs143657517	NA
chr3	1.9E+08	1.9E+08	TGTGTG	-	UTR3	LEPREL1	NA	NA	NA	NA	NA
chr3	1.95E+08	1.95E+08	-	CTC	UTR3	XXYLT1	NA	NA	NA	rs74955255	NA
chr3	1.95E+08	1.95E+08	G	A	ncRNA_exonic	SDHAP2	NA	NA	NA	rs56170658	NA
chr3	1.95E+08	1.95E+08	C	G	ncRNA_exonic	SDHAP2	NA	NA	NA	rs1996904	NA
chr3	1.95E+08	1.95E+08	A	G	ncRNA_exonic	SDHAP2	NA	NA	ID=COSN404758;OCCURENCE=1(lung)	rs6583272	NA
chr3	1.95E+08	1.95E+08	T	C	ncRNA_exonic	SDHAP2	NA	NA	ID=COSN404759;OCCURENCE=1(lung)	rs6583273	NA
chr3	1.95E+08	1.95E+08	T	G	UTR3	MUC20	NA	NA	NA	rs12496423	NA
chr3	1.95E+08	1.95E+08	T	C	UTR3	MUC20	NA	NA	NA	rs2550266	NA
chr3	1.95E+08	1.95E+08	GT	-	UTR3	MUC4	NA	NA	NA	rs142596516	NA
chr3	1.96E+08	1.96E+08	T	C	exonic	MUC4	nonsynonymous SNV	MUC4:NM_018406:exon2:c.A12568G:p.T4190A	NA	rs200813870	NA
chr3	1.96E+08	1.96E+08	T	G	exonic	MUC4	nonsynonymous SNV	MUC4:NM_018406:exon2:c.A12544C:p.T4182P	NA	rs138720131	NA
chr3	1.96E+08	1.96E+08	A	G	exonic	MUC4	synonymous SNV	MUC4:NM_018406:exon2:c.T12159C:p.N4053N	NA	rs201191776	NA
chr3	1.96E+08	1.96E+08	A	G	exonic	MUC4	nonsynonymous SNV	MUC4:NM_018406:exon2:c.T12026C:p.V4009A	NA	rs201051713	NA
chr3	1.96E+08	1.96E+08	C	T	exonic	MUC4	nonsynonymous SNV	MUC4:NM_018406:exon2:c.G11944A:p.A3982T	NA	NA	NA
chr3	1.96E+08	1.96E+08	A	G	exonic	MUC4	synonymous SNV	MUC4:NM_018406:exon2:c.T11799C:p.H3933H	NA	rs62282469	NA
chr3	1.96E+08	1.96E+08	T	A	exonic	MUC4	synonymous SNV	MUC4:NM_018406:exon2:c.A11796T:p.G3932G	NA	rs200897653	NA
chr3	1.96E+08	1.96E+08	C	T	exonic	MUC4	synonymous SNV	MUC4:NM_018406:exon2:c.G11784A:p.S3928S	NA	rs74616719	NA
chr3	1.96E+08	1.96E+08	A	T	exonic	MUC4	synonymous SNV	MUC4:NM_018406:exon2:c.T11643A:p.A3881A	NA	rs3103960	NA
chr3	1.96E+08	1.96E+08	A	G	exonic	MUC4	nonsynonymous SNV	MUC4:NM_018406:exon2:c.T11450C:p.V3817A	OSM1042849;OCCURENCE=1(endometı	rs199746940	NA
chr3	1.96E+08	1.96E+08	A	G	exonic	MUC4	nonsynonymous SNV	MUC4:NM_018406:exon2:c.T11177C:p.V3726A	NA	NA	NA
chr3	1.96E+08	1.96E+08	A	G	exonic	MUC4	synonymous SNV	MUC4:NM_018406:exon2:c.T11166C:p.S3722S	NA	NA	NA
chr3	1.96E+08	1.96E+08	A	G	exonic	MUC4	nonsynonymous SNV	MUC4:NM_018406:exon2:c.T11162C:p.V3721A	NA	NA	NA
chr3	1.96E+08	1.96E+08	C	G	exonic	MUC4	nonsynonymous SNV	MUC4:NM_018406:exon2:c.G10508C:p.S3503T	NA	rs201339881	NA
chr3	1.96E+08	1.96E+08	C	G	exonic	MUC4	nonsynonymous SNV	MUC4:NM_018406:exon2:c.G10405C:p.D3469H	NA	rs76458038	NA
chr3	1.96E+08	1.96E+08	C	T	exonic	MUC4	synonymous SNV	MUC4:NM_018406:exon2:c.G10152A:p.S3384S	NA	rs2453135	NA
chr3	1.96E+08	1.96E+08	C	G	exonic	MUC4	nonsynonymous SNV	MUC4:NM_018406:exon2:c.G10127C:p.R3376P	NA	rs425562	NA
chr3	1.96E+08	1.96E+08	T	C	exonic	MUC4	nonsynonymous SNV	MUC4:NM_018406:exon2:c.A9950G:p.D3317G	NA	NA	NA
chr3	1.96E+08	1.96E+08	A	G	exonic	MUC4	synonymous SNV	MUC4:NM_018406:exon2:c.T9735C:p.H3245H	NA	NA	NA
chr3	1.96E+08	1.96E+08	C	G	exonic	MUC4	nonsynonymous SNV	MUC4:NM_018406:exon2:c.G9639C:p.Q3213H	NA	NA	NA
chr3	1.96E+08	1.96E+08	A	G	exonic	MUC4	synonymous SNV	MUC4:NM_018406:exon2:c.T9564C:p.T3188T	NA	rs76276263	NA
chr3	1.96E+08	1.96E+08	A	G	exonic	MUC4	nonsynonymous SNV	MUC4:NM_018406:exon2:c.T9530C:p.V3177A	NA	rs144952520	NA
chr3	1.96E+08	1.96E+08	C	T	exonic	MUC4	nonsynonymous SNV	MUC4:NM_018406:exon2:c.G9422A:p.S3141N	NA	NA	NA
chr3	1.96E+08	1.96E+08	A	G	exonic	MUC4	synonymous SNV	MUC4:NM_018406:exon2:c.T9324C:p.T3108T	D=COSM479908;OCCURENCE=1(kidney	rs200879287	NA
chr3	1.96E+08	1.96E+08	T	C	exonic	MUC4	nonsynonymous SNV	MUC4:NM_018406:exon2:c.A9049G:p.I3017V	NA	rs71321844	NA
chr3	1.96E+08	1.96E+08	A	G	exonic	MUC4	nonsynonymous SNV	MUC4:NM_018406:exon2:c.T8801C:p.L2934P	NA	NA	NA
chr3	1.96E+08	1.96E+08	C	G	exonic	MUC4	synonymous SNV	MUC4:NM_018406:exon2:c.G8733C:p.T2911T	NA	rs28385894	NA
chr3	1.96E+08	1.96E+08	A	G	exonic	MUC4	nonsynonymous SNV	MUC4:NM_018406:exon2:c.T8570C:p.V2857A	NA	NA	NA
chr3	1.96E+08	1.96E+08	C	T	exonic	MUC4	nonsynonymous SNV	MUC4:NM_018406:exon2:c.G8464A:p.A2822T	NA	rs28420515	NA
chr3	1.96E+08	1.96E+08	C	T	exonic	MUC4	nonsynonymous SNV	MUC4:NM_018406:exon2:c.G8416A:p.A2806T	NA	NA	NA
chr3	1.96E+08	1.96E+08	T	C	exonic	MUC4	nonsynonymous SNV	MUC4:NM_018406:exon2:c.A8392G:p.T2798A	NA	rs202157220	NA
chr3	1.96E+08	1.96E+08	T	G	exonic	MUC4	nonsynonymous SNV	MUC4:NM_018406:exon2:c.A8224C:p.T2742P	NA	NA	NA
chr3	1.96E+08	1.96E+08	G	T	exonic	MUC4	nonsynonymous SNV	MUC4:NM_018406:exon2:c.C7888A:p.P2630T	NA	NA	NA
chr3	1.96E+08	1.96E+08	T	C	exonic	MUC4	nonsynonymous SNV	MUC4:NM_018406:exon2:c.A7768G:p.T2590A	NA	rs76889457	NA
chr3	1.96E+08	1.96E+08	T	G	exonic	MUC4	nonsynonymous SNV	MUC4:NM_018406:exon2:c.A7637C:p.H2546P	NA	rs2453137	NA
chr3	1.96E+08	1.96E+08	C	G	exonic	MUC4	nonsynonymous SNV	MUC4:NM_018406:exon2:c.G7573C:p.D2525H	NA	NA	NA
chr3	1.96E+08	1.96E+08	A	G	exonic	MUC4	synonymous SNV	MUC4:NM_018406:exon2:c.T7518C:p.S2506S	NA	NA	NA
chr3	1.96E+08	1.96E+08	C	G	exonic	MUC4	nonsynonymous SNV	MUC4:NM_018406:exon2:c.G7487C:p.R2496P	NA	rs201759480	NA
chr3	1.96E+08	1.96E+08	C	G	exonic	MUC4	synonymous SNV	MUC4:NM_018406:exon2:c.G7461C:p.S2487S	NA	rs3096347	NA
chr3	1.96E+08	1.96E+08	A	G	exonic	MUC4	nonsynonymous SNV	MUC4:NM_018406:exon2:c.T7445C:p.L2482P	NA	NA	NA
chr3	1.96E+08	1.96E+08	A	G	exonic	MUC4	nonsynonymous SNV	MUC4:NM_018406:exon2:c.T7418C:p.V2473A	NA	rs200807157	NA
chr3	1.96E+08	1.96E+08	C	A	exonic	MUC4	nonsynonymous SNV	MUC4:NM_018406:exon2:c.G7400T:p.G2467V	.COSM149594;OCCURENCE=1(stomach)	rs74867514	NA
chr3	1.96E+08	1.96E+08	C	G	exonic	MUC4	nonsynonymous SNV	MUC4:NM_018406:exon2:c.G7189C:p.D2397H	NA	NA	NA
chr3	1.96E+08	1.96E+08	A	G	exonic	MUC4	nonsynonymous SNV	MUC4:NM_018406:exon2:c.T6977C:p.L2326P	NA	rs71637184	NA
chr3	1.96E+08	1.96E+08	C	T	exonic	MUC4	synonymous SNV	MUC4:NM_018406:exon2:c.G6795A:p.V2265V	NA	NA	NA
chr3	1.96E+08	1.96E+08	C	G	exonic	MUC4	nonsynonymous SNV	MUC4:NM_018406:exon2:c.G6711C:p.Q2237H	NA	rs201069012	NA
chr3	1.96E+08	1.96E+08	A	G	exonic	MUC4	nonsynonymous SNV	MUC4:NM_018406:exon2:c.T6677C:p.L2226P	NA	rs78501179	NA
chr3	1.96E+08	1.96E+08	A	G	exonic	MUC4	nonsynonymous SNV	MUC4:NM_018406:exon2:c.T6293C:p.L2098P	NA	rs141952317	NA
chr3	1.96E+08	1.96E+08	A	T	exonic	MUC4	synonymous SNV	MUC4:NM_018406:exon2:c.T6081A:p.T2027T	NA	NA	NA

chr3	1.96E+08	1.96E+08	A	G	exonic	MUC4	synonymous SNV	MUC4:NM_018406:exon2:c.T6030C:p.S2010S	NA	NA	NA
chr3	1.96E+08	1.96E+08	A	G	exonic	MUC4	synonymous SNV	MUC4:NM_018406:exon2:c.T5991C:p.H1997H	NA	rs201804964	NA
chr3	1.96E+08	1.96E+08	C	T	exonic	MUC4	synonymous SNV	MUC4:NM_018406:exon2:c.G5979A:p.V1993V	NA	NA	NA
chr3	1.96E+08	1.96E+08	C	G	exonic	MUC4	synonymous SNV	MUC4:NM_018406:exon2:c.G5805C:p.T1935T	NA	rs3107750	NA
chr3	1.96E+08	1.96E+08	C	T	exonic	MUC4	synonymous SNV	MUC4:NM_018406:exon2:c.G5400A:p.S1800S	NA	NA	NA
chr3	1.96E+08	1.96E+08	T	A	exonic	MUC4	synonymous SNV	MUC4:NM_018406:exon2:c.A5328T:p.P1776P	NA	NA	NA
chr3	1.96E+08	1.96E+08	A	C	exonic	MUC4	synonymous SNV	MUC4:NM_018406:exon2:c.T5106G:p.V1702V	NA	rs71321841	NA
chr3	1.96E+08	1.96E+08	G	A	exonic	MUC4	nonsynonymous SNV	MUC4:NM_018406:exon2:c.C4919T:p.S1640L	NA	rs202024324	NA
chr3	1.96E+08	1.96E+08	C	G	exonic	MUC4	nonsynonymous SNV	MUC4:NM_018406:exon2:c.G4885C:p.D1629H	NA	rs78438024	NA
chr3	1.96E+08	1.96E+08	C	G	exonic	MUC4	nonsynonymous SNV	MUC4:NM_018406:exon2:c.G4789C:p.D1597H	NA	NA	NA
chr3	1.96E+08	1.96E+08	T	G	exonic	MUC4	nonsynonymous SNV	MUC4:NM_018406:exon2:c.A4517C:p.H1506P	NA	NA	NA
chr3	1.96E+08	1.96E+08	A	G	exonic	MUC4	synonymous SNV	MUC4:NM_018406:exon2:c.T4509C:p.T1503T	NA	NA	NA
chr3	1.96E+08	1.96E+08	T	A	exonic	MUC4	synonymous SNV	MUC4:NM_018406:exon2:c.A4347T:p.A1449A	NA	rs77907507	NA
chr3	1.96E+08	1.96E+08	C	T	exonic	MUC4	nonsynonymous SNV	MUC4:NM_018406:exon2:c.G4216A:p.A1406T	NA	NA	NA
chr3	1.96E+08	1.96E+08	A	G	exonic	MUC4	synonymous SNV	MUC4:NM_018406:exon2:c.T4092C:p.T1364T	NA	rs200702396	NA
chr3	1.96E+08	1.96E+08	A	G	exonic	MUC4	synonymous SNV	MUC4:NM_018406:exon2:c.T3885C:p.T1295T	NA	NA	NA
chr3	1.96E+08	1.96E+08	C	G	exonic	MUC4	synonymous SNV	MUC4:NM_018406:exon2:c.G3837C:p.T1279T	NA	rs200143504	NA
chr3	1.96E+08	1.96E+08	C	G	exonic	MUC4	nonsynonymous SNV	MUC4:NM_018406:exon2:c.G3783C:p.Q1261H	NA	NA	NA
chr3	1.96E+08	1.96E+08	T	G	exonic	MUC4	synonymous SNV	MUC4:NM_018406:exon2:c.A3705C:p.V1235V	NA	rs202086228	NA
chr3	1.96E+08	1.96E+08	C	G	exonic	MUC4	synonymous SNV	MUC4:NM_018406:exon2:c.G3549C:p.T1183T	NA	rs201387180	NA
chr3	1.96E+08	1.96E+08	C	G	exonic	MUC4	nonsynonymous SNV	MUC4:NM_018406:exon2:c.G3495C:p.Q1165H	NA	rs201497377	NA
chr3	1.96E+08	1.96E+08	A	G	exonic	MUC4	synonymous SNV	MUC4:NM_018406:exon2:c.T3471C:p.D1157D	NA	NA	NA
chr3	1.96E+08	1.96E+08	A	G	exonic	MUC4	synonymous SNV	MUC4:NM_018406:exon2:c.T3468C:p.T1156T	NA	NA	NA
chr3	1.96E+08	1.96E+08	A	G	exonic	MUC4	nonsynonymous SNV	MUC4:NM_018406:exon2:c.T3434C:p.V1145A	1149615,COSM149616;OCCURENCE=1(	rs199883835	NA
chr3	1.96E+08	1.96E+08	C	T	exonic	MUC4	nonsynonymous SNV	MUC4:NM_018406:exon2:c.G3085A:p.D1029N	NA	rs201613765	NA
chr3	1.96E+08	1.96E+08	T	C	ncRNA_exonic	SDHAP1	NA	NA	NA	rs189732960	NA
chr3	1.96E+08	1.96E+08	A	G	ncRNA_exonic	SDHAP1	NA	NA	NA	rs199975852	NA
chr3	1.96E+08	1.96E+08	A	G	ncRNA_exonic	SDHAP1	NA	NA	NA	rs200738162	NA
chr3	1.97E+08	1.97E+08	-	TTTTTTT	UTR3	SENP5	NA	NA	NA	NA	NA
chr3	1.98E+08	1.98E+08	GCAGCAGC	-	exonic	FAM157A	nonframeshift deletion	FAM157A:NM_001145248:exon2:c.210_218del:p.70_73del	NA	NA	NA
chr3	1.98E+08	1.98E+08	A	C	exonic	FAM157A	synonymous SNV	FAM157A:NM_001145248:exon5:c.A927C:p.R309R	NA	rs202207735	NA
chr4	87567	87567	G	T	UTR3	ZNF595	NA	NA	NA	NA	NA
chr4	87570	87570	C	T	UTR3	ZNF595	NA	NA	NA	NA	NA
chr4	87574	87574	G	T	UTR3	ZNF595	NA	NA	NA	NA	NA
chr4	1020099	1020099	G	A	UTR3	FGFRL1	NA	NA	NA	NA	NA
chr4	1020221	1020221	T	C	UTR3	FGFRL1	NA	NA	NA	NA	NA
chr4	1388662	1388662	G	C	exonic	CRIPAK	synonymous SNV	CRIPAK:NM_175918:exon1:c.G363C:p.P121P	NA	rs9762106	NA
chr4	1388755	1388755	C	G	exonic	CRIPAK	synonymous SNV	CRIPAK:NM_175918:exon1:c.C456G:p.P152P	NA	rs141481663	NA
chr4	1388757	1388757	T	C	exonic	CRIPAK	nonsynonymous SNV	CRIPAK:NM_175918:exon1:c.T458C:p.M153T	ID=COSM225540;OCCURENCE=1(NS)	rs138170890	P
chr4	1388848	1388848	A	C	exonic	CRIPAK	synonymous SNV	CRIPAK:NM_175918:exon1:c.A549C:p.P183P	NA	rs74511366	NA
chr4	1389059	1389059	T	C	exonic	CRIPAK	nonsynonymous SNV	CRIPAK:NM_175918:exon1:c.T760C:p.C254R	NA	NA	D
chr4	1389070	1389070	A	G	exonic	CRIPAK	synonymous SNV	CRIPAK:NM_175918:exon1:c.A771G:p.G257G	NA	rs151096093	NA
chr4	2087366	2087366	G	T	exonic	POLN	nonsynonymous SNV	POLN:NM_181808:exon19:c.C2171A:p.A724E	NA	NA	B
chr4	2233366	2233366	G	T	UTR3	HAUS3	NA	NA	NA	rs3762940	NA
chr4	2932486	2932486	T	G	UTR3	MFSD10	NA	NA	NA	NA	NA
chr4	5814242	5814242	G	T	UTR3	EVC	NA	NA	NA	NA	NA
chr4	6594947	6594947	A	C	exonic	MAN2B2	nonsynonymous SNV	MAN2B2:NM_015274:exon6:c.A728C:p.Q243P	NA	rs2301796	B
chr4	7743171	7743171	A	G	UTR3	SORCS2	NA	NA	NA	NA	NA
chr4	8582513	8582513	T	G	UTR5	GPR78	NA	NA	NA	rs4235270	NA
chr4	17804654	17804654	T	-	UTR3	DCAF16	NA	NA	NA	NA	NA
chr4	20731086	20731088	ATA	-	UTR3	KCNIP4	NA	NA	NA	rs75646296	NA
chr4	20731095	20731112	TTTAAATG	-	UTR3	KCNIP4	NA	NA	NA	rs139112400	NA
chr4	20852166	20852166	A	G	exonic	KCNIP4	synonymous SNV	IM_001035004:exon2:c.T102C:p.N34N,KCNIP4:NM_025221:exon2:c.T	NA	rs3765121	NA
chr4	22439886	22439886	C	A	exonic	GPR125	nonsynonymous SNV	GPR125:NM_145290:exon8:c.G1078T:p.D360Y	NA	NA	D
chr4	24832309	24832309	C	T	intronic	CCDC149	NA	NA	NA	rs12511680	NA
chr4	25280824	25280824	C	T	UTR3	PI4K2B	NA	NA	NA	NA	NA
chr4	25749200	25749201	AC	-	UTR3	SEL1L3	NA	NA	NA	rs147448161	NA
chr4	37614259	37614260	AA	-	UTR3	RELL1	NA	NA	NA	NA	NA
chr4	38626841	38626841	G	C	ncRNA_exonic	FLJ13197	NA	NA	NA	rs34075114	NA
chr4	39501647	39501647	-	A	UTR3	UGDH	NA	NA	NA	rs71645103	NA
chr4	41747030	41747030	T	G	UTR3	PHOX2B	NA	NA	NA	NA	NA
chr4	46314593	46314593	T	C	exonic	GABRA2	synonymous SNV	_001114175:exon4:c.A396G:p.K132K,GABRA2:NM_000807:exon5:c.A3	=COSM1178493;OCCURENCE=1(prostat	rs279858	NA
chr4	71347281	71347281	G	T	exonic	MUC7	nonsynonymous SNV	:p.A274S,MUC7:NM_001145006:exon4:c.G820T:p.A274S,MUC7:NM_(	NA	NA	NA
chr4	71521932	71521932	A	-	UTR3	IGJ	NA	NA	NA	NA	NA
chr4	74963303	74963303	-	AAAT	UTR3	CXCL2	NA	NA	NA	rs149438582	NA
chr4	74964830	74964830	C	T	exonic	CXCL2	nonsynonymous SNV	CXCL2:NM_002089:exon1:c.G8A:p.R3H	NA	rs186397980	B

chr4	75858518	75858518	G	C	UTR5	PARM1	NA	NA	NA	rs75120017	NA
chr4	76555253	76555253	G	A	UTR5	CDKL2	NA	NA	NA	NA	NA
chr4	78635593	78635614	AAAAAAAA	-	UTR3	CNOT6L	NA	NA	NA	NA	NA
chr4	80824302	80824302	-	A	UTR3	ANTXR2	NA	NA	NA	NA	NA
chr4	80993796	80993796	G	T	UTR5	ANTXR2	NA	NA	NA	rs13140055	NA
chr4	82088324	82088324	G	A	exonic	PRKG2	synonymous SNV	PRKG2:NM_006259:exon5:c.C903T:p.C301C	NA	NA	NA
chr4	83841089	83841116	GATTAAAA	-	UTR3	THAP9	NA	NA	NA	NA	NA
chr4	88225032	88225032	-	GTTA	UTR3	HSD17B13	NA	NA	NA	rs141759852	NA
chr4	88536471	88536471	A	G	exonic	DSPP	nonsynonymous SNV	DSPP:NM_014208:exon5:c.A2657G:p.N886S	NA	NA	NA
chr4	88536475	88536475	A	C	exonic	DSPP	nonsynonymous SNV	DSPP:NM_014208:exon5:c.A2661C:p.E887D	NA	NA	NA
chr4	88536883	88536883	T	C	exonic	DSPP	synonymous SNV	DSPP:NM_014208:exon5:c.T3069C:p.N1023N	NA	rs139018672	NA
chr4	88536899	88536899	A	G	exonic	DSPP	nonsynonymous SNV	DSPP:NM_014208:exon5:c.A3085G:p.N1029D	NA	rs150637282	NA
chr4	88536901	88536901	C	T	exonic	DSPP	synonymous SNV	DSPP:NM_014208:exon5:c.C3087T:p.N1029N	NA	rs148817324	NA
chr4	88537135	88537135	T	C	exonic	DSPP	synonymous SNV	DSPP:NM_014208:exon5:c.T3321C:p.D1107D	NA	NA	NA
chr4	88537150	88537150	C	T	exonic	DSPP	synonymous SNV	DSPP:NM_014208:exon5:c.C3336T:p.S1112S	NA	NA	NA
chr4	88537261	88537261	A	T	exonic	DSPP	nonsynonymous SNV	DSPP:NM_014208:exon5:c.A3447T:p.E1149D	NA	rs200612038	NA
chr4	88537441	88537441	C	T	exonic	DSPP	synonymous SNV	DSPP:NM_014208:exon5:c.C3627T:p.D1209D	NA	NA	NA
chr4	88537509	88537509	A	G	exonic	DSPP	nonsynonymous SNV	DSPP:NM_014208:exon5:c.A3695G:p.N1232S	ID=COSM226972;OCCURENCE=1(skin)	rs111876111	NA
chr4	88537513	88537513	A	C	exonic	DSPP	nonsynonymous SNV	DSPP:NM_014208:exon5:c.A3699C:p.E1233D	NA	rs112275895	NA
chr4	90757919	90757919	T	C	ncRNA_UTR5	SNCA	NA	NA	NA	NA	NA
chr4	94750481	94750481	T	G	exonic	ATOH1	nonsynonymous SNV	ATOH1:NM_005172:exon1:c.T404G:p.V135G	NA	NA	B
chr4	96076626	96076626	-	TT	UTR3	BMPR1B	NA	NA	NA	NA	NA
chr4	1E+08	1E+08	G	C	UTR5	MTTP	NA	NA	NA	rs11944749	NA
chr4	1.01E+08	1.01E+08	AAA	-	UTR3	MTTP	NA	NA	NA	NA	NA
chr4	1.04E+08	1.04E+08	A	T	UTR3	NFKB1	NA	NA	NA	NA	NA
chr4	1.04E+08	1.04E+08	A	T	UTR3	SLC9B1	NA	NA	NA	rs4881610	NA
chr4	1.14E+08	1.14E+08	A	C	exonic	ANK2	nonsynonymous SNV	ANK2:NM_001148:exon38:c.A5702C:p.H1901P	NA	NA	D
chr4	1.14E+08	1.14E+08	T	C	exonic	ANK2	synonymous SNV	ANK2:NM_001148:exon38:c.T5739C:p.R1913R	NA	NA	NA
chr4	1.19E+08	1.19E+08	T	C	exonic	PRSS12	synonymous SNV	PRSS12:NM_003619:exon6:c.A1281G:p.Q427Q	)=COSM149728;OCCURENCE=1(stomac	rs2292597	NA
chr4	1.29E+08	1.29E+08	T	C	UTR3	MFSD8	NA	NA	NA	NA	NA
chr4	1.3E+08	1.3E+08	G	A	UTR3	PHF17	NA	NA	NA	NA	NA
chr4	1.39E+08	1.39E+08	G	T	ncRNA_UTR3	SLC7A11	NA	NA	NA	NA	NA
chr4	1.4E+08	1.4E+08	C	T	UTR3	CCRN4L	NA	NA	NA	NA	NA
chr4	1.4E+08	1.4E+08	A	T	UTR3	CCRN4L	NA	NA	NA	rs74826922	NA
chr4	1.4E+08	1.4E+08	T	-	UTR3	NAA15	NA	NA	NA	rs72425271	NA
chr4	1.46E+08	1.46E+08	A	G	UTR3	OTUD4	NA	NA	NA	NA	NA
chr4	1.53E+08	1.53E+08	A	-	UTR3	FBXW7	NA	NA	NA	rs3841114	NA
chr4	1.57E+08	1.57E+08	A	-	UTR3	GUCY1A3	NA	NA	NA	rs34880260	NA
chr4	1.58E+08	1.58E+08	A	G	UTR5	GRIA2	NA	NA	NA	NA	NA
chr4	1.59E+08	1.59E+08	G	A	exonic	TMEM144	synonymous SNV	TMEM144:NM_018342:exon9:c.G564A:p.V188V	NA	rs12504074	NA
chr4	1.59E+08	1.59E+08	C	A	UTR3	TMEM144	NA	NA	NA	rs1047725	NA
chr4	1.6E+08	1.6E+08	AA	-	UTR3	FNIP2	NA	NA	NA	NA	NA
chr4	1.83E+08	1.83E+08	A	T	ncRNA_exonic	MGC45800	NA	NA	NA	rs11132113	NA
chr4	1.84E+08	1.84E+08	-	ATTTT	ncRNA_exonic	FAM92A1P2	NA	NA	NA	rs10645315	NA
chr4	1.85E+08	1.85E+08	-	A	UTR3	STOX2	NA	NA	NA	NA	NA
chr4	1.87E+08	1.87E+08	C	A	exonic	CYP4V2	nonsynonymous SNV	CYP4V2:NM_207352:exon6:c.C775A:p.Q259K	NA	rs13146272	B
chr4	1.87E+08	1.87E+08	C	T	UTR3	CYP4V2	NA	NA	NA	rs62348765	NA
chr5	443236	443236	C	G	UTR5	C5orf55	NA	NA	NA	rs115554641	NA
chr5	661471	661471	A	C	UTR3	TPPP	NA	NA	NA	rs148811543	NA
chr5	661489	661489	G	C	UTR3	TPPP	NA	NA	NA	rs13160060	NA
chr5	661496	661496	C	G	UTR3	TPPP	NA	NA	NA	rs13174941	NA
chr5	661502	661502	A	C	UTR3	TPPP	NA	NA	NA	NA	NA
chr5	663032	663032	G	A	UTR3	TPPP	NA	NA	NA	NA	NA
chr5	663113	663113	C	T	UTR3	TPPP	NA	NA	NA	rs111333472	NA
chr5	663185	663185	C	T	UTR3	TPPP	NA	NA	NA	rs9313157	NA
chr5	663244	663244	T	G	UTR3	TPPP	NA	NA	NA	NA	NA
chr5	833915	833915	G	T	exonic	ZDHHC11	nonsynonymous SNV	ZDHHC11:NM_024786:exon7:c.C908A:p.A303D	1334,COSM131335;OCCURENCE=1(liver	rs605088	B
chr5	1036435	1036435	G	A	exonic	NKD2	synonymous SNV	_001271082:exon9:c.G723A:p.T241T,NKD2:NM_033120:exon9:c.G723	NA	rs73026757	NA
chr5	10239261	10239261	G	A	exonic	FAM173B	nonsynonymous SNV	24T:p.T75M,FAM173B:NM_001258389:exon2:c.C224T:p.T75M,FAM17	NA	rs2438652	B
chr5	10279657	10279657	T	-	UTR3	CMBL	NA	NA	NA	NA	NA
chr5	14696103	14696103	T	-	UTR3	FAM105B	NA	NA	NA	rs35172360	NA
chr5	15937663	15937663	C	A	UTR3	FBXL7	NA	NA	NA	rs113454368	NA
chr5	16465854	16465854	A	C	UTR5	ZNF622	NA	NA	NA	NA	NA
chr5	16794916	16794916	G	A	exonic	MYO10	synonymous SNV	MYO10:NM_012334:exon4:c.C306T:p.S102S	NA	rs396514	NA
chr5	17276053	17276053	-	CT	UTR3	BASP1	NA	NA	NA	NA	NA
chr5	23521146	23521146	G	T	exonic	PRDM9	synonymous SNV	PRDM9:NM_020227:exon6:c.G366T:p.A122A	NA	NA	NA

chr5	23527323	23527323	C	G	exonic	PRDM9	nonsynonymous SNV	PRDM9:NM_020227:exon11:c.C2126G:p.T709S	NA	rs200539936	B
chr5	23527492	23527492	A	T	exonic	PRDM9	nonsynonymous SNV	PRDM9:NM_020227:exon11:c.A2295T:p.R765S	CURENCE=1(haematopoietic_and_lymp	rs112815500	B
chr5	23527555	23527555	A	C	exonic	PRDM9	nonsynonymous SNV	PRDM9:NM_020227:exon11:c.A2358C:p.R786S	M226129;OCCURENCE=1(NS),2(endom	rs200853373	B
chr5	23527721	23527721	C	A	exonic	PRDM9	nonsynonymous SNV	PRDM9:NM_020227:exon11:c.C2524A:p.R842S	NA	rs201643800	B
chr5	33937999	33937999	A	C	exonic	RXFP3	nonsynonymous SNV	RXFP3:NM_016568:exon1:c.A1154C:p.N385T	NA	NA	D
chr5	35909976	35909976	C	A	exonic	CAPSL	nonsynonymous SNV	_001042625:exon4:c.G517T:p.D173Y,CAPSL:NM_144647:exon4:c.G51	NA	NA	D
chr5	43042495	43042495	T	G	ncRNA_exonic	LOC153684	NA	NA	NA	NA	NA
chr5	43290195	43290195	A	-	UTR3	HMGCS1	NA	NA	NA	NA	NA
chr5	52388455	52388455	A	-	UTR3	ITGA2	NA	NA	NA	NA	NA
chr5	55168106	55168106	G	T	exonic	IL31RA	nonsynonymous SNV	1_001242637:exon4:c.G281T:p.G94V,IL31RA:NM_139017:exon4:c.G28	NA	NA	B
chr5	61000547	61000547	T	-	UTR3	C5orf64	NA	NA	NA	rs34116602	NA
chr5	70672628	70672628	T	-	ncRNA_exonic	PMCHL2	NA	NA	NA	rs199622932	NA
chr5	71503811	71503811	G	T	UTR3	MAP1B	NA	NA	NA	NA	NA
chr5	78324457	78324457	C	A	exonic	DMGDH	nonsynonymous SNV	DMGDH:NM_013391:exon12:c.G1831T:p.A611S	NA	NA	B
chr5	78809641	78809641	C	A	UTR5	HOMER1	NA	NA	NA	NA	NA
chr5	80738520	80738520	G	T	exonic	SSBP2	nonsynonymous SNV	001256735:exon12:c.C711A:p.N237K,SSBP2:NM_001256736:exon12:c	NA	NA	D
chr5	90666826	90666826	A	-	UTR3	ARRDC3	NA	NA	NA	NA	NA
chr5	92905599	92905599	C	A	ncRNA_exonic	FLJ42709	NA	NA	NA	NA	NA
chr5	93488502	93488502	A	T	UTR3	KIAA0825	NA	NA	NA	rs72771613	NA
chr5	93488608	93488608	T	A	UTR3	KIAA0825	NA	NA	NA	rs6892655	NA
chr5	94278129	94278129	T	C	exonic	MCTP1	synonymous SNV	_001002796:exon4:c.A321G:p.V107V,MCTP1:NM_024717:exon4:c.A9	NA	rs293035	NA
chr5	1.02E+08	1.02E+08	GT	-	UTR3	SLCO4C1	NA	NA	NA	NA	NA
chr5	1.09E+08	1.09E+08	AA	-	UTR3	MAN2A1	NA	NA	NA	NA	NA
chr5	1.15E+08	1.15E+08	-	AGGC	UTR5	AP3S1	NA	NA	NA	rs144226661	NA
chr5	1.15E+08	1.15E+08	C	T	exonic	COMMD10	synonymous SNV	COMMD10:NM_016144:exon5:c.C408T:p.T136T	)=COSM149902;OCCURENCE=1(stomac	rs1129496	NA
chr5	1.16E+08	1.16E+08	A	-	UTR3	SEMA6A	NA	NA	NA	rs35304104	NA
chr5	1.19E+08	1.19E+08	-	A	UTR3	HSD17B4	NA	NA	NA	NA	NA
chr5	1.22E+08	1.22E+08	CACACACA	-	UTR3	SNX2	NA	NA	NA	rs71851865	NA
chr5	1.22E+08	1.22E+08	A	-	UTR3	SNX24	NA	NA	NA	NA	NA
chr5	1.24E+08	1.24E+08	-	AA	UTR3	ZNF608	NA	NA	NA	NA	NA
chr5	1.26E+08	1.26E+08	C	T	UTR3	3-Mar	NA	NA	NA	rs75301583	NA
chr5	1.26E+08	1.26E+08	T	-	UTR3	C5orf63	NA	NA	NA	NA	NA
chr5	1.28E+08	1.28E+08	T	-	UTR3	SLC12A2	NA	NA	NA	rs71949635	NA
chr5	1.28E+08	1.28E+08	T	-	UTR3	SLC12A2	NA	NA	NA	rs140582306	NA
chr5	1.31E+08	1.31E+08	-	T	UTR3	RAPGEF6	NA	NA	NA	NA	NA
chr5	1.32E+08	1.32E+08	C	T	UTR3	SLC22A5	NA	NA	NA	NA	NA
chr5	1.32E+08	1.32E+08	T	G	ncRNA_intronic	C5orf56	NA	NA	NA	NA	NA
chr5	1.32E+08	1.32E+08	C	T	ncRNA_intronic	C5orf56	NA	NA	NA	rs2522050	NA
chr5	1.34E+08	1.34E+08	C	A	UTR3	DDX46	NA	NA	NA	NA	NA
chr5	1.34E+08	1.34E+08	AAATAAAT	-	UTR3	C5orf24	NA	NA	NA	NA	NA
chr5	1.35E+08	1.35E+08	G	T	ncRNA_exonic	SMAD5-AS1	NA	NA	NA	NA	NA
chr5	1.36E+08	1.36E+08	T	C	ncRNA_exonic	LOC389332	NA	NA	NA	rs6889814	NA
chr5	1.37E+08	1.37E+08	A	C	UTR3	HNRNPA0	NA	NA	NA	NA	NA
chr5	1.37E+08	1.37E+08	T	C	UTR3	HNRNPA0	NA	NA	NA	NA	NA
chr5	1.37E+08	1.37E+08	G	C	exonic	HNRNPA0	synonymous SNV	HNRNPA0:NM_006805:exon1:c.C897G:p.G299G	NA	NA	NA
chr5	1.37E+08	1.37E+08	A	C	exonic	HNRNPA0	synonymous SNV	HNRNPA0:NM_006805:exon1:c.T888G:p.G296G	NA	NA	NA
chr5	1.38E+08	1.38E+08	-	GTGT	UTR3	LRRTM2	NA	NA	NA	NA	NA
chr5	1.39E+08	1.39E+08	C	G	exonic	CXXC5	synonymous SNV	CXXC5:NM_016463:exon2:c.C294G:p.G98G	NA	NA	NA
chr5	1.39E+08	1.39E+08	-	A	UTR3	PURA	NA	NA	NA	NA	NA
chr5	1.41E+08	1.41E+08	G	T	UTR3	PCDHB9	NA	NA	NA	NA	NA
chr5	1.41E+08	1.41E+08	T	C	UTR3	PCDHB9	NA	NA	NA	rs2907326	NA
chr5	1.49E+08	1.49E+08	C	G	ncRNA_exonic	MIR143HG	NA	NA	NA	rs519814	NA
chr5	1.49E+08	1.49E+08	C	A	downstream	CSNK1A1	NA	NA	NA	NA	NA
chr5	1.49E+08	1.49E+08	G	A	downstream	CSNK1A1	NA	NA	NA	NA	NA
chr5	1.49E+08	1.49E+08	C	T	exonic	ARHGEF37	nonsynonymous SNV	ARHGEF37:NM_001001669:exon11:c.C1466T:p.P489L	NA	rs9324624	D
chr5	1.49E+08	1.49E+08	C	T	UTR3	PDE6A	NA	NA	NA	rs72660270	NA
chr5	1.49E+08	1.49E+08	C	T	UTR3	PDE6A	NA	NA	NA	rs888895	NA
chr5	1.5E+08	1.5E+08	T	G	UTR5	TCOF1	NA	NA	NA	rs4565199	NA
chr5	1.5E+08	1.5E+08	A	T	UTR3	SYNPO	NA	NA	NA	NA	NA
chr5	1.51E+08	1.51E+08	T	C	UTR3	SLC36A2	NA	NA	NA	rs364420	NA
chr5	1.51E+08	1.51E+08	-	AAT	UTR3	SLC36A2	NA	NA	NA	rs10681775	NA
chr5	1.51E+08	1.51E+08	-	A	UTR3	SLC36A2	NA	NA	NA	rs33912867	NA
chr5	1.52E+08	1.52E+08	-	A	UTR3	NMUR2	NA	NA	NA	NA	NA
chr5	1.56E+08	1.56E+08	-	GTT	exonic	HAVCR1	nonframeshift insertion	,HAVCR1:NM_001099414:exon4:c.477_478insAAC:p.T159delinsTT,HA	NA	rs139041445	NA
chr5	1.56E+08	1.56E+08	C	G	UTR5	HAVCR1	NA	NA	NA	rs9313422	NA
chr5	1.57E+08	1.57E+08	-	A	UTR3	CYFIP2	NA	NA	NA	NA	NA

chr5	1.58E+08	1.58E+08	-	A	UTR3	EBF1	NA	NA	NA	NA	NA
chr5	1.59E+08	1.59E+08	CA	-	UTR3	UBLCP1	NA	NA	NA	NA	NA
chr5	1.61E+08	1.61E+08	-	T	UTR3	GABRB2	NA	NA	NA	NA	NA
chr5	1.62E+08	1.62E+08	C	T	exonic	GABRG2	synonymous SNV	3T:p.N196N,GABRG2:NM_198903:exon5:c.C588T:p.N196N,GABRG2:NI	=COSM150008;OCCURENCE=1(stomacl	rs211037	NA
chr5	1.68E+08	1.68E+08	C	A	ncRNA_exonic	FBLL1	NA	NA	NA	rs111777993	NA
chr5	1.7E+08	1.7E+08	-	G	ncRNA_exonic	LOC257358	NA	NA	NA	rs201174922	NA
chr5	1.72E+08	1.72E+08	A	G	UTR3	SH3PXD2B	NA	NA	NA	rs10050631	NA
chr5	1.73E+08	1.73E+08	A	G	exonic	BOD1	synonymous SNV	BOD1:NM_138369:exon3:c.T543C:p.S181S	ID=COSM311048;OCCURENCE=1(lung)	rs77014290	NA
chr5	1.76E+08	1.76E+08	T	G	UTR3	EIF4E1B	NA	NA	NA	NA	NA
chr5	1.76E+08	1.76E+08	A	C	exonic	UNC5A	nonsynonymous SNV	UNC5A:NM_133369:exon8:c.A1246C:p.T416P	NA	NA	B
chr5	1.77E+08	1.77E+08	T	G	UTR3	PROP1	NA	NA	NA	NA	NA
chr6	407629	407629	-	T	UTR3	IRF4	NA	NA	NA	NA	NA
chr6	2889933	2889933	A	-	UTR3	SERPINB9	NA	NA	NA	rs10714355	NA
chr6	7249296	7249299	GAGA	-	UTR3	RREB1	NA	NA	NA	rs66585613	NA
chr6	10796303	10796303	T	G	exonic	MAK	nonsynonymous SNV	71C:p.Q357H,MAK:NM_001242957:exon9:c.A1071C:p.Q357H,MAK:NM	NA	NA	P
chr6	11714197	11714197	-	A	UTR3	ADTRP	NA	NA	NA	NA	NA
chr6	11714639	11714639	G	A	UTR3	ADTRP	NA	NA	NA	rs200727372	NA
chr6	15521323	15521323	G	A	UTR3	JARID2	NA	NA	NA	NA	NA
chr6	17688638	17688638	C	A	exonic	NUP153	nonsynonymous SNV	NUP153:NM_005124:exon2:c.G323T:p.S108I	NA	NA	B
chr6	18129306	18129306	C	A	UTR3	TPMT	NA	NA	NA	NA	NA
chr6	21597697	21597697	C	T	UTR3	SOX4	NA	NA	NA	NA	NA
chr6	21597716	21597716	C	T	UTR3	SOX4	NA	NA	NA	NA	NA
chr6	25769247	25769247	C	A	exonic	SLC17A4	synonymous SNV	SLC17A4:NM_005495:exon3:c.C126A:p.I42I	NA	NA	NA
chr6	26525864	26525864	-	A	ncRNA_exonic	HCG11	NA	NA	NA	rs35998206	NA
chr6	29640039	29640039	G	A	UTR3	MOG	NA	NA	NA	rs1984840	NA
chr6	29857177	29857177	G	A	ncRNA_exonic	HLA-H	NA	NA	NA	rs1059578	NA
chr6	29867090	29867090	A	C	intergenic	-H(dist=8234),HCG4B(dist=25	NA	NA	NA	rs9259500	NA
chr6	29867345	29867345	-	CT	intergenic	-H(dist=8489),HCG4B(dist=25	NA	NA	NA	rs142277328	NA
chr6	29867768	29867768	G	A	intergenic	-H(dist=8912),HCG4B(dist=24	NA	NA	NA	rs3094183	NA
chr6	29892568	29892568	C	A	ncRNA_exonic	HCG4B	NA	NA	NA	NA	NA
chr6	29892570	29892570	A	T	ncRNA_exonic	HCG4B	NA	NA	NA	NA	NA
chr6	29912149	29912149	G	C	exonic	HLA-A	synonymous SNV	_001242758:exon4:c.G870C:p.L290L,HLA-A:NM_002116:exon4:c.G87	NA	rs1136832	NA
chr6	30232436	30232436	G	A	ncRNA_exonic	HLA-L	NA	NA	NA	rs3094630	NA
chr6	30232545	30232548	AGAA	-	ncRNA_exonic	HLA-L	NA	NA	NA	rs144443573	NA
chr6	30233356	30233356	-	TTTA	ncRNA_exonic	HLA-L	NA	NA	NA	rs148801141	NA
chr6	30672634	30672634	C	G	exonic	MDC1	synonymous SNV	MDC1:NM_014641:exon10:c.G4326C:p.V1442V	NA	NA	NA
chr6	30673064	30673064	C	T	exonic	MDC1	nonsynonymous SNV	MDC1:NM_014641:exon10:c.G3896A:p.R1299Q	NA	rs144657716	NA
chr6	30673422	30673422	A	G	exonic	MDC1	nonsynonymous SNV	MDC1:NM_014641:exon10:c.T3538C:p.S1180P	NA	rs9461623	P
chr6	30673432	30673432	C	T	exonic	MDC1	synonymous SNV	MDC1:NM_014641:exon10:c.G3528A:p.Q1176Q	NA	rs78306040	NA
chr6	30918208	30918208	T	C	exonic	DPCR1	nonsynonymous SNV	DPCR1:NM_080870:exon2:c.T1967C:p.F656S	NA	NA	NA
chr6	30918690	30918690	T	C	exonic	DPCR1	nonsynonymous SNV	DPCR1:NM_080870:exon2:c.T2449C:p.S817P	NA	NA	NA
chr6	30919155	30919155	G	A	exonic	DPCR1	nonsynonymous SNV	DPCR1:NM_080870:exon2:c.G2914A:p.D972N	NA	rs185499634	NA
chr6	30954600	30954600	A	C	exonic	MUC21	nonsynonymous SNV	MUC21:NM_001010909:exon2:c.A648C:p.R216S	NA	NA	NA
chr6	30954611	30954611	A	G	exonic	MUC21	nonsynonymous SNV	MUC21:NM_001010909:exon2:c.A659G:p.N220S	NA	rs202115956	NA
chr6	30954618	30954618	T	C	exonic	MUC21	synonymous SNV	MUC21:NM_001010909:exon2:c.T666C:p.A222A	NA	NA	NA
chr6	30955127	30955127	C	A	exonic	MUC21	nonsynonymous SNV	MUC21:NM_001010909:exon2:c.C1175A:p.T392N	NA	rs9262397	NA
chr6	31022557	31022557	G	A	ncRNA_intronic	HCG22	NA	NA	NA	rs2523853	NA
chr6	31024175	31024175	T	C	ncRNA_exonic	HCG22	NA	NA	NA	rs117227928	NA
chr6	31025713	31025713	C	A	ncRNA_intronic	HCG22	NA	NA	NA	rs2517524	NA
chr6	31133943	31133943	C	A	UTR5	POU5F1	NA	NA	NA	rs3130932	NA
chr6	31141612	31141612	A	T	ncRNA_exonic	PSORS1C3	NA	NA	NA	rs2269711	NA
chr6	31324525	31324525	C	T	exonic	HLA-B	nonsynonymous SNV	HLA-B:NM_005514:exon2:c.G283A:p.A95T	NA	rs1131213	NA
chr6	31324526	31324526	C	G	exonic	HLA-B	nonsynonymous SNV	HLA-B:NM_005514:exon2:c.G282C:p.Q94H	NA	rs1131212	NA
chr6	31324528	31324528	G	T	exonic	HLA-B	nonsynonymous SNV	HLA-B:NM_005514:exon2:c.C280A:p.Q94K	NA	rs1071817	NA
chr6	31324531	31324531	C	T	exonic	HLA-B	nonsynonymous SNV	HLA-B:NM_005514:exon2:c.G277A:p.A93T	NA	rs1131204	NA
chr6	31324536	31324536	T	G	exonic	HLA-B	nonsynonymous SNV	HLA-B:NM_005514:exon2:c.A272C:p.Y91S	NA	rs1071816	NA
chr6	31324547	31324547	G	C	exonic	HLA-B	nonsynonymous SNV	HLA-B:NM_005514:exon2:c.C261G:p.N87K	.;OCCURENCE=1(haematopoietic_and_l	rs151341177	NA
chr6	31324549	31324549	T	C	exonic	HLA-B	nonsynonymous SNV	HLA-B:NM_005514:exon2:c.A259G:p.N87D	2;OCCURENCE=1(haematopoietic_and_l	rs143633217	NA
chr6	31324586	31324586	C	T	exonic	HLA-B	synonymous SNV	HLA-B:NM_005514:exon2:c.G222A:p.P74P	NA	rs139850307	NA
chr6	31465899	31465899	G	C	UTR5	MICB	NA	NA	NA	rs2516498	NA
chr6	31465905	31465905	G	T	UTR5	MICB	NA	NA	NA	rs2516497	NA
chr6	31465915	31465915	G	C	UTR5	MICB	NA	NA	NA	rs3828916	NA
chr6	31595773	31595784	TGCTGCCC	-	exonic	PRRC2A	nonframeshift deletion	n12:c.1522_1533del:p.508_511del,PRRC2A:NM_080686:exon12:c.152	NA	rs149965706	NA
chr6	31976161	31976161	A	G	exonic	CYP21A2	unknown	UNKNOWN	NA	rs200893608	NA
chr6	32548550	32548550	G	A	exonic	HLA-DRB1	synonymous SNV	HLA-DRB1:NM_002124:exon4:c.C736T:p.L246L	NA	rs1059362	NA
chr6	32549596	32549596	T	C	exonic	HLA-DRB1	synonymous SNV	HLA-DRB1:NM_002124:exon3:c.A390G:p.V130V	NA	rs111823233	NA

chr6	32557504	32557504	G	A	exonic	HLA-DRB1	nonsynonymous SNV	HLA-DRB1:NM_002124:exon1:c.C16T:p.L6F	NA	rs17879020	NA
chr6	32605234	32605234	G	A	UTR5	HLA-DQA1	NA	NA	NA	rs1047987	NA
chr6	32845561	32845561	A	-	intergenic	ist=17933),LOC100294145(dis	NA	NA	NA	rs34093445	NA
chr6	32846167	32846167	C	T	intergenic	ist=18539),LOC100294145(dis	NA	NA	NA	NA	NA
chr6	33032968	33032968	G	A	UTR3	HLA-DPA1	NA	NA	NA	rs8486	NA
chr6	33054353	33054353	G	A	UTR3	HLA-DPB1	NA	NA	NA	rs9277522	NA
chr6	33054355	33054355	A	C	UTR3	HLA-DPB1	NA	NA	NA	rs9277523	NA
chr6	33179668	33179668	T	G	exonic	RING1	synonymous SNV	RING1:NM_002931:exon6:c.T1008G:p.G336G	NA	rs117630006	NA
chr6	33422545	33422545	C	G	UTR5	ZBTB9	NA	NA	NA	rs2274730	NA
chr6	36853786	36853786	-	G	UTR5	C6orf89	NA	NA	NA	NA	NA
chr6	36995222	36995222	G	A	exonic	FGD2	synonymous SNV	FGD2:NM_173558:exon15:c.G1623A:p.T541T	NA	rs2274587	NA
chr6	36995281	36995281	G	A	exonic	FGD2	nonsynonymous SNV	FGD2:NM_173558:exon15:c.G1682A:p.S561N	NA	rs148473105	B
chr6	38139332	38139353	TGTGTGTG	-	UTR3	BTBD9	NA	NA	NA	rs72137261	NA
chr6	38140548	38140548	G	A	UTR3	BTBD9	NA	NA	NA	NA	NA
chr6	39053943	39053948	ACACAC	-	UTR3	GLP1R	NA	NA	NA	NA	NA
chr6	39284184	39284184	A	G	exonic	KCNK16	synonymous SNV	_001135106:exon5:c.T696C:p.Y232Y,KCNK16:NM_032115:exon5:c.T6	NA	rs3734618	NA
chr6	40360273	40360273	C	T	exonic	LRFN2	synonymous SNV	LRFN2:NM_020737:exon3:c.G1779A:p.G593G	NA	NA	NA
chr6	41090722	41090722	C	A	ncRNA_exonic	ADCY10P1	NA	NA	NA	NA	NA
chr6	41158908	41158909	TG	-	UTR3	TREML2	NA	NA	NA	NA	NA
chr6	42931628	42931631	TTTA	-	UTR3	PEX6	NA	NA	NA	rs144286892	NA
chr6	42946943	42946943	G	A	UTR5	PEX6	NA	NA	NA	rs9462859	NA
chr6	43584152	43584152	A	G	UTR3	POLH	NA	NA	NA	rs9472090	NA
chr6	45517740	45517740	T	-	UTR3	RUNX2	NA	NA	NA	rs11320232	NA
chr6	45868268	45868268	-	AAAAAAAA	UTR3	CLIC5	NA	NA	NA	NA	NA
chr6	46672210	46672221	CTCTCACA	-	UTR3	PLA2G7	NA	NA	NA	rs144459411	NA
chr6	47445789	47445789	A	C	UTR5	CD2AP	NA	NA	NA	rs1056434	NA
chr6	47792560	47792560	T	-	UTR3	OPN5	NA	NA	NA	NA	NA
chr6	49460746	49460746	C	A	UTR3	CENPQ	NA	NA	NA	NA	NA
chr6	49460762	49460762	G	T	UTR3	CENPQ	NA	NA	NA	NA	NA
chr6	52272471	52272471	C	A	UTR3	PAQR8	NA	NA	NA	rs2397089	NA
chr6	52362410	52362410	T	G	UTR3	TRAM2	NA	NA	NA	NA	NA
chr6	52362424	52362424	G	T	UTR3	TRAM2	NA	NA	NA	NA	NA
chr6	52365317	52365317	T	G	UTR3	TRAM2	NA	NA	NA	rs9395798	NA
chr6	56033094	56033094	G	A	exonic	COL21A1	nonsynonymous SNV	COL21A1:NM_030820:exon6:c.C1028T:p.T343M	NA	rs35471617	NA
chr6	57393144	57393144	A	T	exonic	PRIM2	unknown	UNKNOWN	=COSM1131820;OCCURENCE=1(prostat	rs3763183	NA
chr6	57398207	57398207	G	T	exonic	PRIM2	unknown	UNKNOWN	NA	rs71214816	NA
chr6	57512841	57512841	A	G	UTR3	PRIM2	NA	NA	NA	rs78256005	NA
chr6	57512850	57512850	C	G	UTR3	PRIM2	NA	NA	NA	rs77911716	NA
chr6	57512889	57512889	C	T	UTR3	PRIM2	NA	NA	NA	rs75351177	NA
chr6	57512902	57512902	G	A	UTR3	PRIM2	NA	NA	NA	rs77563921	NA
chr6	57512916	57512916	-	T	UTR3	PRIM2	NA	NA	NA	rs200683929	NA
chr6	57512933	57512933	G	A	UTR3	PRIM2	NA	NA	NA	rs77947729	NA
chr6	57513082	57513082	C	T	UTR3	PRIM2	NA	NA	NA	rs75390508	NA
chr6	57513140	57513140	-	AACA	UTR3	PRIM2	NA	NA	NA	rs56224260	NA
chr6	57513160	57513160	G	A	UTR3	PRIM2	NA	NA	NA	rs76503032	NA
chr6	57513205	57513205	C	T	UTR3	PRIM2	NA	NA	NA	rs74617308	NA
chr6	57513292	57513292	C	T	UTR3	PRIM2	NA	NA	NA	rs79506978	NA
chr6	57513366	57513366	A	G	UTR3	PRIM2	NA	NA	NA	rs74652947	NA
chr6	70917069	70917070	TT	-	UTR3	COL19A1	NA	NA	NA	NA	NA
chr6	71666227	71666227	G	A	UTR5	B3GAT2	NA	NA	NA	rs9446313	NA
chr6	73907362	73907362	A	C	UTR3	KCNQ5	NA	NA	NA	rs10943079	NA
chr6	74536576	74536576	-	T	UTR3	CD109	NA	NA	NA	rs11451739	NA
chr6	76640781	76640781	C	T	exonic	IMPG1	nonsynonymous SNV	IMPG1:NM_001563:exon15:c.G2132A:p.R711H	NA	rs3734313	D
chr6	80411320	80411320	T	-	UTR3	SH3BGRL2	NA	NA	NA	NA	NA
chr6	83074365	83074365	C	T	UTR5	TPBG	NA	NA	NA	rs770911	NA
chr6	90346603	90346603	C	T	UTR3	LYRM2	NA	NA	NA	rs1134136	NA
chr6	97592002	97592005	AAAG	-	ncRNA_UTR3	MMS22L	NA	NA	NA	NA	NA
chr6	97592005	97592008	GAGA	-	ncRNA_UTR3	MMS22L	NA	NA	NA	NA	NA
chr6	99724024	99724024	A	-	UTR3	FAXC	NA	NA	NA	rs11307978	NA
chr6	1.06E+08	1.06E+08	A	G	UTR3	BVES	NA	NA	NA	rs7771833	NA
chr6	1.07E+08	1.07E+08	C	A	UTR3	PRDM1	NA	NA	NA	NA	NA
chr6	1.07E+08	1.07E+08	C	T	UTR3	PRDM1	NA	NA	NA	NA	NA
chr6	1.07E+08	1.07E+08	T	C	ncRNA_intronic	LOC100422737	NA	NA	NA	rs3117417	NA
chr6	1.07E+08	1.07E+08	A	-	UTR3	BEND3	NA	NA	NA	NA	NA
chr6	1.1E+08	1.1E+08	T	C	exonic	PPIL6	nonsynonymous SNV	_001111298:exon3:c.A329G:p.H110R,PPIL6:NM_173672:exon3:c.A329	NA	rs9398200	P
chr6	1.1E+08	1.1E+08	G	A	UTR5	PPIL6,SMPD2	NA	NA	NA	rs1048203	NA

chr6	1.14E+08	1.14E+08	T	-	UTR3	MARCKS	NA	NA	NA	NA	NA
chr6	1.32E+08	1.32E+08	C	G	exonic	ARG1	nonsynonymous SNV	M_000045:exon2:c.C82G:p.P28A,ARG1:NM_001244438:exon2:c.C82G	NA	NA	P
chr6	1.36E+08	1.36E+08	C	A	UTR5	AHI1	NA	NA	NA	rs13197384	NA
chr6	1.37E+08	1.37E+08	AA	-	UTR3	PDE7B	NA	NA	NA	NA	NA
chr6	1.37E+08	1.37E+08	T	C	UTR3	BCLAF1	NA	NA	NA	rs74299323	NA
chr6	1.39E+08	1.39E+08	A	-	UTR3	KIAA1244	NA	NA	NA	NA	NA
chr6	1.44E+08	1.44E+08	C	T	UTR3	ADAT2	NA	NA	NA	rs9484744	NA
chr6	1.44E+08	1.44E+08	T	-	UTR3	PHACTR2	NA	NA	NA	NA	NA
chr6	1.44E+08	1.44E+08	A	T	ncRNA_exonic	HYMAI	NA	NA	NA	rs1474882	NA
chr6	1.45E+08	1.45E+08	C	T	UTR3	STX11	NA	NA	NA	rs148087652	NA
chr6	1.51E+08	1.51E+08	G	-	UTR3	PPP1R14C	NA	NA	NA	rs201167515	NA
chr6	1.53E+08	1.53E+08	T	C	exonic	MTRF1L	nonsynonymous SNV	_001114184:exon1:c.A112G:p.T38A,MTRF1L:NM_019041:exon1:c.A1	NA	rs3818125	B
chr6	1.56E+08	1.56E+08	AA	-	UTR3	TFB1M,TIAM2	NA	NA	NA	rs139032680	NA
chr6	1.58E+08	1.58E+08	C	T	UTR3	ARID1B	NA	NA	NA	NA	NA
chr6	1.59E+08	1.59E+08	A	-	UTR3	GTF2H5	NA	NA	NA	rs3841147	NA
chr6	1.59E+08	1.59E+08	T	-	UTR3	GTF2H5	NA	NA	NA	NA	NA
chr6	1.59E+08	1.59E+08	A	C	exonic	TULP4	nonsynonymous SNV	TULP4:NM_020245:exon13:c.A2306C:p.N769T	ID=COSM400671;OCCURENCE=1(lung)	NA	B
chr6	1.6E+08	1.6E+08	A	G	exonic	SOD2	nonsynonymous SNV	7C:p.V16A,SOD2:NM_001024465:exon2:c.T47C:p.V16A,SOD2:NM_00	NA	rs4880	NA
chr6	1.6E+08	1.6E+08	G	T	UTR3	WTAP	NA	NA	NA	NA	NA
chr6	1.6E+08	1.6E+08	G	T	UTR3	WTAP	NA	NA	NA	NA	NA
chr6	1.61E+08	1.61E+08	A	-	ncRNA_exonic	LOC729603	NA	NA	NA	NA	NA
chr6	1.61E+08	1.61E+08	TG	-	UTR3	IGF2R	NA	NA	NA	NA	NA
chr6	1.61E+08	1.61E+08	C	A	exonic	MAP3K4	nonsynonymous SNV	_005922:exon4:c.C1909A:p.Q637K,MAP3K4:NM_006724:exon4:c.C19	NA	NA	D
chr6	1.64E+08	1.64E+08	A	C	UTR3	QKI	NA	NA	NA	NA	NA
chr6	1.68E+08	1.68E+08	C	T	ncRNA_exonic	MLLT4-AS1	NA	NA	NA	rs112715448	NA
chr6	1.68E+08	1.68E+08	A	C	ncRNA_exonic	MLLT4-AS1	NA	NA	NA	NA	NA
chr6	1.68E+08	1.68E+08	A	C	ncRNA_exonic	MLLT4-AS1	NA	NA	NA	NA	NA
chr6	1.68E+08	1.68E+08	TG	-	UTR3	MLLT4	NA	NA	NA	NA	NA
chr6	1.68E+08	1.68E+08	C	T	exonic	HGC6.3	nonsynonymous SNV	HGC6.3:NM_001129895:exon1:c.G464A:p.G155E	NA	rs201374833	NA
chr6	1.68E+08	1.68E+08	A	G	exonic	HGC6.3	nonsynonymous SNV	HGC6.3:NM_001129895:exon1:c.T230C:p.F77S	NA	NA	NA
chr6	1.68E+08	1.68E+08	A	T	exonic	KIF25	nonsynonymous SNV	NM_005355:exon2:c.A83T:p.K28M,KIF25:NM_030615:exon2:c.A83T:p	NA	rs4708626	B
chr6	1.71E+08	1.71E+08	G	A	exonic	FAM120B	nonsynonymous SNV	FAM120B:NM_032448:exon2:c.G1088A:p.R363Q	NA	NA	B
chr6	1.71E+08	1.71E+08	G	A	exonic	TBP	synonymous SNV	_001172085:exon2:c.G168A:p.Q56Q,TBP:NM_003194:exon3:c.G228A	SM247746;OCCURENCE=1(prostate),3	rs112083427	NA
chr7	537549	537549	G	A	UTR3	PDGFA	NA	NA	NA	NA	NA
chr7	537550	537550	G	A	UTR3	PDGFA	NA	NA	NA	NA	NA
chr7	537959	537959	T	C	UTR3	PDGFA	NA	NA	NA	rs77670994	NA
chr7	5516293	5516293	T	C	UTR3	FBXL18	NA	NA	NA	rs12535397	NA
chr7	5517337	5517337	A	-	UTR3	FBXL18	NA	NA	NA	NA	NA
chr7	5886611	5886611	C	G	ncRNA_exonic	ZNF815P	NA	NA	NA	rs308084	NA
chr7	6193464	6193464	T	G	exonic	USP42	nonsynonymous SNV	USP42:NM_032172:exon15:c.T2279G:p.L760R	NA	rs61732616	NA
chr7	6442143	6442143	C	-	UTR3	RAC1	NA	NA	NA	rs59685280	NA
chr7	6566322	6566322	T	G	exonic	GRID2IP	synonymous SNV	GRID2IP:NM_001145118:exon3:c.A663C:p.A221A	NA	rs2881723	NA
chr7	20180426	20180427	AC	-	UTR3	MACC1	NA	NA	NA	rs10555255	NA
chr7	20796144	20796144	G	T	UTR3	ABCB5	NA	NA	NA	rs12112555	NA
chr7	21941862	21941865	TCTT	-	UTR3	CDCA7L	NA	NA	NA	rs60370798	NA
chr7	22158512	22158512	C	A	UTR3	RAPGEF5	NA	NA	NA	NA	NA
chr7	23749892	23749892	C	G	UTR5	STK31	NA	NA	NA	NA	NA
chr7	26252966	26252966	C	T	UTR3	CBX3	NA	NA	NA	rs28407029	NA
chr7	27147452	27147453	AA	-	UTR3	HOXA3	NA	NA	NA	NA	NA
chr7	29687058	29687058	T	G	ncRNA_exonic	LOC646762	NA	NA	NA	rs28457783	NA
chr7	29687091	29687091	C	T	ncRNA_exonic	LOC646762	NA	NA	NA	rs28390296	NA
chr7	29687927	29687927	T	C	ncRNA_exonic	LOC646762	NA	NA	NA	rs1728641	NA
chr7	29923572	29923572	T	C	exonic	WIPF3	synonymous SNV	WIPF3:NM_001080529:exon5:c.T462C:p.N154N	NA	rs28439342	NA
chr7	30412409	30412409	A	-	ncRNA_exonic	DKFZP586I1420	NA	NA	NA	NA	NA
chr7	30796018	30796018	-	TTTT	ncRNA_UTR3	INMT	NA	NA	NA	NA	NA
chr7	30796273	30796273	T	C	ncRNA_UTR3	INMT	NA	NA	NA	rs7787251	NA
chr7	32525555	32525556	AA	-	UTR3	LSM5	NA	NA	NA	rs10598963	NA
chr7	35734529	35734529	C	G	UTR5	HERPUD2	NA	NA	NA	rs78381277	NA
chr7	35734591	35734591	-	G	UTR5	HERPUD2	NA	NA	NA	rs11394396	NA
chr7	37960263	37960275	GCAGTGGC	-	UTR5	EPDR1	NA	NA	468;OCCURENCE=1(ovary),1(breast),1(	rs201513905	NA
chr7	38725735	38725735	A	C	ncRNA_exonic	FAM183B	NA	NA	NA	NA	NA
chr7	41729121	41729121	-	T	UTR3	INHBA	NA	NA	NA	NA	NA
chr7	42001570	42001570	A	-	UTR3	GLI3	NA	NA	NA	rs144064690	NA
chr7	43590153	43590153	T	G	exonic	HECW1	nonsynonymous SNV	HECW1:NM_015052:exon27:c.T4358G:p.V1453G	NA	NA	P
chr7	44073817	44073817	A	G	ncRNA_exonic	RASA4CP	NA	NA	NA	NA	NA
chr7	44073823	44073823	T	C	ncRNA_exonic	RASA4CP	NA	NA	NA	NA	NA

chr7	44258882	44258882	G	T	UTR3	CAMK2B	NA	NA	NA	rs113136811	NA
chr7	44258923	44258923	G	T	UTR3	CAMK2B	NA	NA	NA	NA	NA
chr7	44873374	44873374	C	T	UTR3	H2AFV	NA	NA	NA	rs36047398	NA
chr7	44917408	44917408	A	-	UTR3	PURB	NA	NA	NA	NA	NA
chr7	45123081	45123081	C	G	exonic	NACAD	nonsynonymous SNV	NACAD:NM_001146334:exon2:c.G2698C:p.V900L	NA	NA	NA
chr7	45123337	45123337	C	T	exonic	NACAD	synonymous SNV	NACAD:NM_001146334:exon2:c.G2442A:p.S814S	NA	rs61740885	NA
chr7	45123379	45123379	G	A	exonic	NACAD	synonymous SNV	NACAD:NM_001146334:exon2:c.C2400T:p.S800S	NA	NA	NA
chr7	47568718	47568718	C	T	UTR5	TNS3	NA	NA	NA	rs10261525	NA
chr7	51261280	51261280	C	T	exonic	COBL	synonymous SNV	COBL:NM_015198:exon3:c.G252A:p.A84A	NA	rs1437488	NA
chr7	64436818	64436818	-	A	UTR3	ZNF117	NA	NA	NA	NA	NA
chr7	65171393	65171393	G	A	ncRNA_exonic	INTS4L2	NA	NA	NA	rs12532775	NA
chr7	66774667	66774667	G	T	ncRNA_exonic	STAG3L4	NA	NA	NA	NA	NA
chr7	72336469	72336469	T	-	ncRNA_exonic	SPDYE7P	NA	NA	NA	rs58909282	NA
chr7	72338105	72338105	A	-	ncRNA_exonic	SPDYE7P	NA	NA	NA	NA	NA
chr7	72413581	72413581	G	A	exonic	POM121	nonsynonymous SNV	.72020:exon11:c.G2254A:p.V752I,POM121:NM_001257190:exon14:c.	NA	rs71554687	B
chr7	76131725	76131725	T	C	exonic	DTX2	synonymous SNV	001102595:exon7:c.T1341C:p.H447H,DTX2:NM_001102594:exon8:c.T	NA	NA	NA
chr7	77558429	77558429	G	T	exonic	PHTF2	nonsynonymous SNV	.A337S,PHTF2:NM_001127357:exon10:c.G1021T:p.A341S,PHTF2:NM_	NA	NA	NA
chr7	92088538	92088538	-	A	UTR3	GATAD1	NA	NA	NA	NA	NA
chr7	92985252	92985252	T	C	exonic	CCDC132	synonymous SNV	017667:exon27:c.T2635C:p.L879L,CCDC132:NM_001257998:exon28:c.	NA	rs2374639	NA
chr7	94923585	94923585	C	T	UTR3	PPP1R9A	NA	NA	NA	NA	NA
chr7	95727014	95727014	T	C	UTR3	DYNC1I1	NA	NA	NA	rs7794344	NA
chr7	98444287	98444287	C	T	UTR3	TMEM130	NA	NA	NA	rs1047035	NA
chr7	99766216	99766216	C	A	UTR5	GAL3ST4	NA	NA	NA	NA	NA
chr7	1E+08	1E+08	A	C	exonic	NYAP1	nonsynonymous SNV	NYAP1:NM_173564:exon4:c.A797C:p.Y266S	NA	NA	D
chr7	1E+08	1E+08	C	G	exonic	NYAP1	nonsynonymous SNV	NYAP1:NM_173564:exon4:c.C815G:p.A272G	NA	NA	B
chr7	1E+08	1E+08	T	G	exonic	NYAP1	synonymous SNV	NYAP1:NM_173564:exon4:c.T816G:p.A272A	NA	NA	NA
chr7	1E+08	1E+08	G	C	exonic	NYAP1	nonsynonymous SNV	NYAP1:NM_173564:exon4:c.G836C:p.G279A	NA	NA	P
chr7	1E+08	1E+08	T	-	UTR3	AGFG2	NA	NA	NA	rs145117814	NA
chr7	1E+08	1E+08	C	A	exonic	ZAN	unknown	UNKNOWN	NA	NA	NA
chr7	1E+08	1E+08	T	C	exonic	ZAN	unknown	UNKNOWN	NA	NA	NA
chr7	1E+08	1E+08	T	C	exonic	ZAN	unknown	UNKNOWN	SM393154,COSM393155;OCCURENCE=	NA	NA
chr7	1E+08	1E+08	T	C	exonic	ZAN	unknown	UNKNOWN	NA	NA	NA
chr7	1.01E+08	1.01E+08	T	C	exonic	MUC12	synonymous SNV	MUC12:NM_001164462:exon2:c.T2670C:p.D890D	NA	NA	NA
chr7	1.01E+08	1.01E+08	A	C	exonic	MUC12	nonsynonymous SNV	MUC12:NM_001164462:exon2:c.A2682C:p.L894F	NA	NA	NA
chr7	1.01E+08	1.01E+08	A	T	exonic	MUC12	synonymous SNV	MUC12:NM_001164462:exon2:c.A2721T:p.S907S	NA	NA	NA
chr7	1.01E+08	1.01E+08	T	C	exonic	MUC12	synonymous SNV	MUC12:NM_001164462:exon2:c.T2745C:p.G915G	NA	NA	NA
chr7	1.01E+08	1.01E+08	A	C	exonic	MUC12	synonymous SNV	MUC12:NM_001164462:exon2:c.A2748C:p.S916S	NA	NA	NA
chr7	1.01E+08	1.01E+08	T	A	exonic	MUC12	synonymous SNV	MUC12:NM_001164462:exon2:c.T2751A:p.T917T	NA	NA	NA
chr7	1.01E+08	1.01E+08	G	A	exonic	MUC12	nonsynonymous SNV	MUC12:NM_001164462:exon2:c.G2758A:p.A920T	NA	rs192144740	NA
chr7	1.01E+08	1.01E+08	C	A	exonic	MUC12	synonymous SNV	MUC12:NM_001164462:exon2:c.C2784A:p.A928A	NA	rs184584311	NA
chr7	1.01E+08	1.01E+08	A	C	exonic	MUC12	nonsynonymous SNV	MUC12:NM_001164462:exon2:c.A2798C:p.Q933P	NA	NA	NA
chr7	1.01E+08	1.01E+08	A	T	exonic	MUC12	synonymous SNV	MUC12:NM_001164462:exon2:c.A2808T:p.T936T	NA	NA	NA
chr7	1.01E+08	1.01E+08	G	A	exonic	MUC12	nonsynonymous SNV	MUC12:NM_001164462:exon2:c.G2858A:p.S953N	NA	NA	NA
chr7	1.01E+08	1.01E+08	C	A	exonic	MUC12	synonymous SNV	MUC12:NM_001164462:exon2:c.C2913A:p.G971G	NA	rs200215189	NA
chr7	1.01E+08	1.01E+08	A	G	exonic	MUC12	synonymous SNV	MUC12:NM_001164462:exon2:c.A2916G:p.S972S	NA	NA	NA
chr7	1.01E+08	1.01E+08	A	G	exonic	MUC12	nonsynonymous SNV	MUC12:NM_001164462:exon2:c.A2941G:p.S981G	NA	NA	NA
chr7	1.01E+08	1.01E+08	C	T	exonic	MUC12	synonymous SNV	MUC12:NM_001164462:exon2:c.C2946T:p.S982S	NA	NA	NA
chr7	1.01E+08	1.01E+08	G	-	exonic	MUC12	frameshift deletion	MUC12:NM_001164462:exon2:c.2951delG:p.S984fs	NA	NA	NA
chr7	1.01E+08	1.01E+08	T	-	exonic	MUC12	frameshift deletion	MUC12:NM_001164462:exon2:c.2955delT:p.P985fs	NA	NA	NA
chr7	1.01E+08	1.01E+08	GACT	-	exonic	MUC12	frameshift deletion	MUC12:NM_001164462:exon2:c.2957_2960del:p.986_987del	NA	NA	NA
chr7	1.01E+08	1.01E+08	A	G	exonic	MUC12	nonsynonymous SNV	MUC12:NM_001164462:exon2:c.A2990G:p.H997R	NA	NA	NA
chr7	1.01E+08	1.01E+08	C	G	exonic	MUC12	nonsynonymous SNV	MUC12:NM_001164462:exon2:c.C2991G:p.H997Q	NA	NA	NA
chr7	1.01E+08	1.01E+08	G	A	exonic	MUC12	nonsynonymous SNV	MUC12:NM_001164462:exon2:c.G2995A:p.A999T	NA	NA	NA
chr7	1.01E+08	1.01E+08	A	G	exonic	MUC12	synonymous SNV	MUC12:NM_001164462:exon2:c.A3000G:p.S1000S	NA	NA	NA
chr7	1.01E+08	1.01E+08	C	A	exonic	MUC12	nonsynonymous SNV	MUC12:NM_001164462:exon2:c.C3002A:p.T1001N	NA	NA	NA
chr7	1.01E+08	1.01E+08	C	G	exonic	MUC12	nonsynonymous SNV	MUC12:NM_001164462:exon2:c.C3004G:p.H1002D	NA	NA	NA
chr7	1.01E+08	1.01E+08	G	C	exonic	MUC12	synonymous SNV	MUC12:NM_001164462:exon2:c.G3012C:p.T1004T	NA	NA	NA
chr7	1.01E+08	1.01E+08	A	T	exonic	MUC12	nonsynonymous SNV	MUC12:NM_001164462:exon2:c.A3034T:p.T1012S	NA	NA	NA
chr7	1.01E+08	1.01E+08	C	T	exonic	MUC12	nonsynonymous SNV	MUC12:NM_001164462:exon2:c.C3041T:p.P1014L	NA	NA	NA
chr7	1.01E+08	1.01E+08	A	C	exonic	MUC12	nonsynonymous SNV	MUC12:NM_001164462:exon2:c.A3062C:p.Y1021S	NA	NA	NA
chr7	1.01E+08	1.01E+08	C	G	exonic	MUC12	nonsynonymous SNV	MUC12:NM_001164462:exon2:c.C3067G:p.R1023G	NA	NA	NA
chr7	1.01E+08	1.01E+08	A	G	exonic	MUC12	synonymous SNV	MUC12:NM_001164462:exon2:c.A3075G:p.P1025P	NA	NA	NA
chr7	1.01E+08	1.01E+08	G	A	exonic	MUC12	nonsynonymous SNV	MUC12:NM_001164462:exon2:c.G3076A:p.G1026S	NA	NA	NA
chr7	1.01E+08	1.01E+08	G	A	exonic	MUC12	synonymous SNV	MUC12:NM_001164462:exon2:c.G3081A:p.S1027S	NA	NA	NA
chr7	1.01E+08	1.01E+08	C	A	exonic	MUC12	nonsynonymous SNV	MUC12:NM_001164462:exon2:c.C3820A:p.L1274I	NA	NA	NA
chr7	1.01E+08	1.01E+08	T	G	exonic	MUC12	synonymous SNV	MUC12:NM_001164462:exon2:c.T3849G:p.T1283T	NA	NA	NA

chr7	1.01E+08	1.01E+08	A	G	exonic	MUC12	nonsynonymous SNV	MUC12:NM_001164462:exon2:c.A4043G:p.D1348G	NA	rs112404953	NA
chr7	1.01E+08	1.01E+08	G	C	exonic	MUC12	nonsynonymous SNV	MUC12:NM_001164462:exon2:c.G7226C:p.S2409T	NA	NA	NA
chr7	1.01E+08	1.01E+08	A	C	exonic	MUC12	synonymous SNV	MUC12:NM_001164462:exon2:c.A7230C:p.T2410T	NA	NA	NA
chr7	1.01E+08	1.01E+08	A	G	exonic	MUC12	nonsynonymous SNV	MUC12:NM_001164462:exon2:c.A7241G:p.Q2414R	NA	NA	NA
chr7	1.01E+08	1.01E+08	G	A	exonic	MUC12	nonsynonymous SNV	MUC12:NM_001164462:exon2:c.G8069A:p.R2690H	NA	NA	NA
chr7	1.01E+08	1.01E+08	G	C	exonic	MUC12	nonsynonymous SNV	MUC12:NM_001164462:exon2:c.G10142C:p.S3381T	NA	NA	NA
chr7	1.01E+08	1.01E+08	C	T	exonic	MUC12	nonsynonymous SNV	MUC12:NM_001164462:exon2:c.C11036T:p.T3679I	NA	rs201665808	NA
chr7	1.01E+08	1.01E+08	C	G	exonic	MUC12	nonsynonymous SNV	MUC12:NM_001164462:exon2:c.C11327G:p.P3776R	NA	rs201148522	NA
chr7	1.01E+08	1.01E+08	T	C	exonic	MUC12	nonsynonymous SNV	MUC12:NM_001164462:exon2:c.T12355C:p.F4119L	NA	rs181671450	NA
chr7	1.02E+08	1.02E+08	C	A	UTR3	RASA4	NA	NA	NA	NA	NA
chr7	1.03E+08	1.03E+08	A	-	UTR3	ARMC10	NA	NA	NA	NA	NA
chr7	1.03E+08	1.03E+08	A	T	ncRNA_exonic	RPL19P12	NA	NA	NA	NA	NA
chr7	1.05E+08	1.05E+08	C	A	ncRNA_exonic	LHFPL3-AS2	NA	NA	NA	NA	NA
chr7	1.07E+08	1.07E+08	A	C	UTR3	COG5	NA	NA	NA	NA	NA
chr7	1.14E+08	1.14E+08	A	T	UTR3	PPP1R3A	NA	NA	NA	NA	NA
chr7	1.16E+08	1.16E+08	-	A	UTR3	CAV2	NA	NA	NA	NA	NA
chr7	1.2E+08	1.2E+08	A	C	exonic	KCND2	synonymous SNV	KCND2:NM_012281:exon1:c.A873C:p.T291T	NA	NA	NA
chr7	1.2E+08	1.2E+08	A	T	exonic	KCND2	nonsynonymous SNV	KCND2:NM_012281:exon1:c.A898T:p.I300F	NA	NA	D
chr7	1.21E+08	1.21E+08	A	C	exonic	WNT16	nonsynonymous SNV	NM_016087:exon2:c.A253C:p.T85P,WNT16:NM_057168:exon2:c.A283	NA	NA	B
chr7	1.22E+08	1.22E+08	G	A	exonic	AASS	nonsynonymous SNV	AASS:NM_005763:exon14:c.C1456T:p.L486F	NA	NA	D
chr7	1.23E+08	1.23E+08	C	A	UTR3	SLC13A1	NA	NA	NA	NA	NA
chr7	1.23E+08	1.23E+08	AA	-	UTR3	SLC13A1	NA	NA	NA	NA	NA
chr7	1.27E+08	1.27E+08	-	ACAT	UTR3	PAX4	NA	NA	NA	rs200757052	NA
chr7	1.29E+08	1.29E+08	GCCCACTC	-	exonic	IRF5	nonframeshift deletion	9:exon6:c.550_579del:p.184_193del,IRF5:NM_001098630:exon6:c.50	;OCCURENCE=1(haematopoietic_and_l	NA	NA
chr7	1.3E+08	1.3E+08	TGTGTG	-	UTR3	CPA4	NA	NA	NA	rs146877444	NA
chr7	1.3E+08	1.3E+08	T	C	exonic	CPA5	synonymous SNV	.T261C:p.S87S,CPA5:NM_080385:exon5:c.T261C:p.S87S,CPA5:NM_00	NA	rs7808823	NA
chr7	1.3E+08	1.3E+08	T	C	exonic	CPA5	nonsynonymous SNV	CPA5:NM_001127442:exon11:c.T1073C:p.M358T	NA	rs17164867	NA
chr7	1.3E+08	1.3E+08	C	T	exonic	CPA1	synonymous SNV	CPA1:NM_001868:exon4:c.C474T:p.Y158Y	NA	rs968404	NA
chr7	1.31E+08	1.31E+08	C	A	exonic	PODXL	nonsynonymous SNV	1_001018111:exon2:c.G488T:p.S163I,PODXL:NM_005397:exon2:c.G48	NA	NA	P
chr7	1.37E+08	1.37E+08	CA	-	UTR5	CHRM2	NA	NA	NA	rs35916399	NA
chr7	1.39E+08	1.39E+08	TT	-	UTR3	KIAA1549	NA	NA	NA	NA	NA
chr7	1.39E+08	1.39E+08	GT	-	UTR3	UBN2	NA	NA	NA	NA	NA
chr7	1.39E+08	1.39E+08	A	-	UTR3	UBN2	NA	NA	NA	rs3217265	NA
chr7	1.39E+08	1.39E+08	A	C	UTR3	HIPK2	NA	NA	NA	NA	NA
chr7	1.4E+08	1.4E+08	T	G	exonic	DENND2A	synonymous SNV	DENND2A:NM_015689:exon1:c.A906C:p.L302L	NA	NA	NA
chr7	1.4E+08	1.4E+08	AA	-	ncRNA_exonic	NDUFB2-AS1	NA	NA	NA	NA	NA
chr7	1.43E+08	1.43E+08	A	-	ncRNA_exonic	OR6W1P	NA	NA	NA	NA	NA
chr7	1.43E+08	1.43E+08	A	G	exonic	CTAGE15	synonymous SNV	CTAGE15:NM_001008747:exon1:c.A1839G:p.G613G	NA	NA	NA
chr7	1.43E+08	1.43E+08	T	A	exonic	CTAGE15	synonymous SNV	CTAGE15:NM_001008747:exon1:c.T1869A:p.P623P	NA	NA	NA
chr7	1.43E+08	1.43E+08	T	C	exonic	CTAGE15	nonsynonymous SNV	CTAGE15:NM_001008747:exon1:c.T1915C:p.S639P	NA	NA	NA
chr7	1.44E+08	1.44E+08	A	G	exonic	CTAGE4	synonymous SNV	CTAGE4:NM_198495:exon1:c.T2268C:p.Y756Y	NA	rs202035285	NA
chr7	1.49E+08	1.49E+08	-	C	exonic	KRBA1	unknown	UNKNOWN	NA	rs145097116	NA
chr7	1.51E+08	1.51E+08	C	A	exonic	ASB10	nonsynonymous SNV	79T:p.C260F,ASB10:NM_001142460:exon3:c.G779T:p.C260F,ASB10:N	NA	NA	B
chr7	1.51E+08	1.51E+08	A	C	UTR3	PRKAG2	NA	NA	NA	rs199973452	NA
chr7	1.52E+08	1.52E+08	C	G	exonic	MLL3	nonsynonymous SNV	MLL3:NM_170606:exon16:c.G2755C:p.V919L	1131646,COSM1131647;OCCURENCE=1	rs111791757	B
chr7	1.52E+08	1.52E+08	G	A	exonic	MLL3	nonsynonymous SNV	MLL3:NM_170606:exon14:c.C2315T:p.S772L	NA	rs4024453	P
chr7	1.55E+08	1.55E+08	T	A	UTR3	DPP6	NA	NA	NA	rs78561625	NA
chr7	1.55E+08	1.55E+08	C	A	ncRNA_exonic	LOC202781	NA	NA	NA	NA	NA
chr7	1.59E+08	1.59E+08	G	A	exonic	ESYT2	synonymous SNV	ESYT2:NM_020728:exon17:c.C2091T:p.A697A	NA	rs57686648	NA
chr7	1.59E+08	1.59E+08	G	C	exonic	ESYT2	synonymous SNV	ESYT2:NM_020728:exon17:c.C2082G:p.P694P	NA	rs59980573	NA
chr8	161222	161222	G	C	ncRNA_exonic	RPL23AP53	NA	NA	NA	rs2906360	NA
chr8	1729586	1729586	T	G	UTR3	CLN8	NA	NA	NA	NA	NA
chr8	1733460	1733460	C	T	UTR3	CLN8	NA	NA	NA	NA	NA
chr8	1733665	1733665	T	G	UTR3	CLN8	NA	NA	NA	NA	NA
chr8	1733674	1733674	T	G	UTR3	CLN8	NA	NA	NA	NA	NA
chr8	6359075	6359075	T	-	UTR3	ANGPT2	NA	NA	NA	NA	NA
chr8	10464990	10464990	T	C	exonic	RP1L1	synonymous SNV	RP1L1:NM_178857:exon4:c.A6618G:p.Q2206Q	NA	NA	NA
chr8	10465086	10465086	T	C	exonic	RP1L1	synonymous SNV	RP1L1:NM_178857:exon4:c.A6522G:p.Q2174Q	NA	NA	NA
chr8	10465721	10465721	A	C	exonic	RP1L1	nonsynonymous SNV	RP1L1:NM_178857:exon4:c.T5887G:p.S1963A	NA	rs190549596	NA
chr8	10465919	10465919	A	C	exonic	RP1L1	nonsynonymous SNV	RP1L1:NM_178857:exon4:c.T5689G:p.W1897G	NA	NA	NA
chr8	10582231	10582231	T	-	UTR3	SOX7	NA	NA	NA	rs113669505	NA
chr8	11406593	11406593	T	C	exonic	BLK	synonymous SNV	BLK:NM_001715:exon5:c.T330C:p.S110S	NA	rs3816668	NA
chr8	11973535	11973535	A	G	ncRNA_exonic	FAM66D	NA	NA	NA	NA	NA
chr8	12039821	12039821	G	T	UTR3	FAM86B1	NA	NA	NA	rs143950143	NA
chr8	12040786	12040786	C	G	UTR3	FAM86B1	NA	NA	NA	rs201122164	NA
chr8	12040998	12040998	G	A	UTR3	FAM86B1	NA	NA	NA	rs141787656	NA

chr8	12283157	12283157	T	C	UTR3	FAM86B2	NA	NA	NA	rs2698896	NA
chr8	12283281	12283281	G	A	UTR3	FAM86B2	NA	NA	NA	rs2684074	NA
chr8	12286307	12286307	T	C	exonic	FAM86B2	nonsynonymous SNV	FAM86B2:NM_001137610:exon6:c.A577G:p.I193V	NA	rs148161726	NA
chr8	12286609	12286609	T	C	exonic	FAM86B2	synonymous SNV	FAM86B2:NM_001137610:exon5:c.A357G:p.S119S	NA	rs2684085	NA
chr8	17079490	17079491	TT	-	UTR3	ZDHHC2	NA	NA	NA	NA	NA
chr8	17579505	17579505	A	-	UTR5	MTUS1	NA	NA	NA	rs34302917	NA
chr8	20106557	20106557	A	G	UTR3	LZTS1	NA	NA	NA	NA	NA
chr8	23536561	23536563	TTT	-	UTR3	NKX3-1	NA	NA	NA	rs140739091	NA
chr8	27143184	27143184	A	-	UTR3	TRIM35	NA	NA	NA	rs34388696	NA
chr8	27879723	27879732	GGAGTGCA	-	UTR3	NUGGC	NA	NA	NA	rs113806414	NA
chr8	28205758	28205758	A	C	UTR3	ZNF395	NA	NA	NA	NA	NA
chr8	28925544	28925544	T	G	UTR3	KIF13B	NA	NA	NA	NA	NA
chr8	38034284	38034284	T	C	UTR5	BAG4	NA	NA	NA	rs55962869	NA
chr8	41481358	41481358	T	-	UTR3	AGPAT6	NA	NA	NA	NA	NA
chr8	41583441	41583441	T	C	exonic	ANK1	synonymous SNV	549G:p.V183V,ANK1:NM_020475:exon6:c.A450G:p.V150V,ANK1:NM_	NA	rs6982971	NA
chr8	41788246	41788246	A	T	UTR3	KAT6A	NA	NA	NA	NA	NA
chr8	41790779	41790779	T	G	exonic	KAT6A	synonymous SNV	P1653P,KAT6A:NM_001099412:exon18:c.A4959C:p.P1653P,KAT6A:NM	NA	NA	NA
chr8	42877229	42877229	A	-	UTR3	HOOK3	NA	NA	NA	NA	NA
chr8	52732614	52732614	T	C	UTR3	PCMTD1	NA	NA	NA	NA	NA
chr8	52732641	52732641	T	C	UTR3	PCMTD1	NA	NA	NA	rs73592209	NA
chr8	57075770	57075771	TT	-	UTR3	PLAG1	NA	NA	NA	NA	NA
chr8	57307835	57307835	C	A	intergenic	6C5(dist=74594),PENK(dist=4	NA	NA	NA	NA	NA
chr8	67405682	67405682	A	-	UTR5	C8orf46	NA	NA	NA	rs5892058	NA
chr8	72981327	72981327	A	G	exonic	TRPA1	synonymous SNV	TRPA1:NM_007332:exon3:c.T375C:p.N125N	NA	rs1811457	NA
chr8	81886342	81886342	C	A	UTR3	PAG1	NA	NA	NA	NA	NA
chr8	81886387	81886387	-	TT	UTR3	PAG1	NA	NA	NA	NA	NA
chr8	86572249	86572249	A	G	UTR3	REXO1L1	NA	NA	NA	rs28564465	NA
chr8	86572578	86572578	T	C	UTR3	REXO1L1	NA	NA	NA	rs28399792	NA
chr8	86572958	86572958	T	G	UTR3	REXO1L1	NA	NA	NA	rs145855931	NA
chr8	86786659	86786659	G	T	UTR3	REXO1L1	NA	NA	NA	NA	NA
chr8	90946056	90946056	A	G	UTR3	NBN	NA	NA	NA	rs9995	NA
chr8	92971169	92971170	AG	-	UTR3	RUNX1T1	NA	NA	NA	rs36062285	NA
chr8	92971184	92971184	T	-	UTR3	RUNX1T1	NA	NA	NA	NA	NA
chr8	1.02E+08	1.02E+08	G	A	exonic	PABPC1	nonsynonymous SNV	PABPC1:NM_002568:exon11:c.C1477T:p.R493C	NA	rs62513924	P
chr8	1.02E+08	1.02E+08	G	A	exonic	PABPC1	nonsynonymous SNV	PABPC1:NM_002568:exon8:c.C1205T:p.P402L	NA	rs139094790	B
chr8	1.02E+08	1.02E+08	C	A	exonic	PABPC1	stopgain SNV	PABPC1:NM_002568:exon8:c.G1033T:p.E345X	NA	rs142985461	NA
chr8	1.16E+08	1.16E+08	C	A	UTR3	TRPS1	NA	NA	NA	NA	NA
chr8	1.23E+08	1.23E+08	C	T	ncRNA_UTR5	HAS2	NA	NA	NA	NA	NA
chr8	1.23E+08	1.23E+08	C	T	ncRNA_UTR5	HAS2	NA	NA	NA	NA	NA
chr8	1.34E+08	1.34E+08	TT	-	UTR3	SLA	NA	NA	NA	NA	NA
chr8	1.42E+08	1.42E+08	T	C	exonic	MROH5	unknown	UNKNOWN	NA	rs73713680	NA
chr8	1.42E+08	1.42E+08	C	T	exonic	MROH5	unknown	UNKNOWN	NA	rs73713681	NA
chr8	1.45E+08	1.45E+08	G	C	exonic	MROH6	synonymous SNV	MROH6:NM_001100878:exon1:c.C192G:p.P64P	NA	rs4873804	NA
chr8	1.45E+08	1.45E+08	T	C	exonic	SCRIB	synonymous SNV	15356:exon32:c.A4350G:p.P1450P,SCRIB:NM_182706:exon32:c.A435	NA	rs6991873	NA
chr8	1.46E+08	1.46E+08	C	A	UTR3	ZNF250	NA	NA	NA	NA	NA
chr8	1.46E+08	1.46E+08	T	A	ncRNA_exonic	TMED10P1	NA	NA	NA	NA	NA
chr9	14665	14665	G	A	UTR3	WASH1	NA	NA	ID=COSN405103;OCCURENCE=1(lung)	rs149305563	NA
chr9	14764	14764	C	T	UTR3	WASH1	NA	NA	NA	rs199668463	NA
chr9	117998	117998	G	T	exonic	FOXD4	nonsynonymous SNV	FOXD4:NM_207305:exon1:c.C122A:p.A41E	ID=COSM226644;OCCURENCE=1(skin)	rs66612967	B
chr9	178937	178937	C	T	exonic	CBWD1	synonymous SNV	M_001145356:exon1:c.G33A:p.E11E,CBWD1:NM_018491:exon1:c.G3	NA	rs16925052	NA
chr9	178947	178947	G	A	exonic	CBWD1	nonsynonymous SNV	NM_001145356:exon1:c.C23T:p.A8V,CBWD1:NM_018491:exon1:c.C2	NA	rs16925054	B
chr9	179063	179063	C	G	UTR5	CBWD1	NA	NA	NA	rs75051610	NA
chr9	3824936	3824937	TA	-	UTR3	GLIS3	NA	NA	NA	NA	NA
chr9	14083479	14083479	A	-	UTR3	NFIB	NA	NA	NA	NA	NA
chr9	14993511	14993511	T	G	ncRNA_exonic	LOC389705	NA	NA	NA	rs1880012	NA
chr9	15019719	15019719	T	C	ncRNA_exonic	LOC389705	NA	NA	NA	rs1916311	NA
chr9	16412719	16412730	GAGAGAGA	-	UTR3	BNC2	NA	NA	NA	NA	NA
chr9	16413355	16413355	T	-	UTR3	BNC2	NA	NA	NA	rs5896689	NA
chr9	27274108	27274108	G	T	ncRNA_exonic	LINC00032	NA	NA	NA	rs2057363	NA
chr9	33043991	33043992	AA	-	UTR3	SMU1	NA	NA	NA	rs141620295	NA
chr9	33111314	33111314	T	A	UTR3	B4GALT1	NA	NA	NA	NA	NA
chr9	33253761	33253761	A	-	UTR3	BAG1	NA	NA	NA	NA	NA
chr9	33385733	33385733	C	T	exonic	AQP7	nonsynonymous SNV	AQP7:NM_001170:exon7:c.G657A:p.M219I	180629;OCCURENCE=1(upper_aerodige	rs201117022	B
chr9	33385771	33385771	C	T	exonic	AQP7	nonsynonymous SNV	AQP7:NM_001170:exon7:c.G619A:p.G207S	NA	rs117663392	B
chr9	33386144	33386144	G	A	exonic	AQP7	synonymous SNV	AQP7:NM_001170:exon6:c.C456T:p.V152V	NA	rs76209395	NA
chr9	33386146	33386146	C	A	exonic	AQP7	nonsynonymous SNV	AQP7:NM_001170:exon6:c.G454T:p.V152F	NA	rs76608797	B

chr9	33387047	33387047	T	G	exonic	AQP7	nonsynonymous SNV	AQP7:NM_001170:exon4:c.A188C:p.K63T	NA	rs4008658	B
chr9	33387073	33387073	G	A	exonic	AQP7	synonymous SNV	AQP7:NM_001170:exon4:c.C162T:p.S54S	NA	rs73645276	NA
chr9	33447424	33447424	C	G	exonic	AQP3	synonymous SNV	AQP3:NM_004925:exon1:c.G105C:p.L35L	NA	rs591810	NA
chr9	34833858	34833858	T	A	ncRNA_exonic	FAM205B	NA	NA	NA	rs3739879	NA
chr9	37864593	37864593	-	T	UTR3	DCAF10	NA	NA	NA	NA	NA
chr9	38398304	38398304	T	C	UTR3	ALDH1B1	NA	NA	NA	rs10973781	NA
chr9	41953380	41953380	C	T	ncRNA_exonic	MGC21881	NA	NA	NA	rs78604336	NA
chr9	41954166	41954166	C	T	ncRNA_exonic	MGC21881	NA	NA	NA	rs11262437	NA
chr9	41960604	41960604	C	T	ncRNA_exonic	KGFLP2	NA	NA	NA	rs200748812	NA
chr9	43032807	43032807	A	G	ncRNA_exonic	FAM95B1	NA	NA	NA	rs1965864	NA
chr9	43624925	43624925	A	G	exonic	SPATA31A6	synonymous SNV	SPATA31A6:NM_001145196:exon4:c.T3762C:p.H1254H	NA	rs2762364	NA
chr9	45727897	45727897	C	G	ncRNA_exonic	FAM27A	NA	NA	NA	NA	NA
chr9	45727901	45727901	A	G	ncRNA_exonic	FAM27A	NA	NA	NA	NA	NA
chr9	66500435	66500435	C	A	ncRNA_exonic	PTGER4P2-CDK2AP2P2	NA	NA	NA	rs200229634	NA
chr9	66501021	66501021	C	T	ncRNA_exonic	PTGER4P2-CDK2AP2P2	NA	NA	NA	rs4913214	NA
chr9	66501045	66501045	T	C	ncRNA_exonic	PTGER4P2-CDK2AP2P2	NA	NA	NA	rs62549190	NA
chr9	66501106	66501106	G	A	ncRNA_exonic	PTGER4P2-CDK2AP2P2	NA	NA	NA	rs78246050	NA
chr9	66502730	66502730	T	G	ncRNA_exonic	PTGER4P2-CDK2AP2P2	NA	NA	NA	rs78123270	NA
chr9	66502733	66502733	G	T	ncRNA_exonic	PTGER4P2-CDK2AP2P2	NA	NA	NA	rs75664577	NA
chr9	69202059	69202059	T	C	UTR5	FOXD4L6	NA	NA	NA	NA	NA
chr9	69424288	69424288	T	C	UTR3	ANKRD20A4	NA	NA	NA	rs78290728	NA
chr9	69424311	69424311	C	A	UTR3	ANKRD20A4	NA	NA	NA	rs3866289	NA
chr9	69663680	69663680	A	C	ncRNA_exonic	LOC100133920	NA	NA	NA	rs201199565	NA
chr9	69664901	69664901	T	C	ncRNA_exonic	LOC100133920	NA	NA	NA	NA	NA
chr9	70177312	70177312	C	T	exonic	FOXD4L5	synonymous SNV	FOXD4L5:NM_001126334:exon1:c.G672A:p.A224A	NA	rs2921292	NA
chr9	70177559	70177559	A	G	exonic	FOXD4L5	nonsynonymous SNV	FOXD4L5:NM_001126334:exon1:c.T425C:p.F142S	NA	NA	NA
chr9	70919869	70919869	C	A	UTR3	FOXD4L3	NA	NA	NA	rs200191707	NA
chr9	94495608	94495608	T	C	exonic	ROR2	nonsynonymous SNV	ROR2:NM_004560:exon6:c.A733G:p.T245A	NA	rs10820900	B
chr9	96439004	96439004	-	GCCTCCACC	exonic	PHF2	nonframeshift insertion	NM_005392:exon21:c.2961_2962insCCTGCCTCCACCACA:p.T987delinsT	NA	rs149736720	NA
chr9	99264877	99264877	T	-	UTR3	CDC14B	NA	NA	NA	rs34988377	NA
chr9	99538299	99538299	C	A	exonic	ZNF510	nonsynonymous SNV	ZNF510:NM_014930:exon2:c.G53T:p.G18V	NA	NA	D
chr9	1.01E+08	1.01E+08	T	A	UTR5	CORO2A	NA	NA	NA	rs2275709	NA
chr9	1.01E+08	1.01E+08	G	T	UTR3	GABBR2	NA	NA	NA	rs73657131	NA
chr9	1.02E+08	1.02E+08	G	T	UTR3	TGFBR1	NA	NA	NA	rs199545273	NA
chr9	1.03E+08	1.03E+08	A	C	exonic	NR4A3	nonsynonymous SNV	24C:p.T142P,NR4A3:NM_173199:exon3:c.A424C:p.T142P,NR4A3:NM_	NA	NA	D
chr9	1.1E+08	1.1E+08	A	C	exonic	ZNF462	nonsynonymous SNV	ZNF462:NM_021224:exon3:c.A4303C:p.T1435P	NA	NA	B
chr9	1.12E+08	1.12E+08	G	C	UTR5	IKBKAP	NA	NA	NA	rs2275641	NA
chr9	1.12E+08	1.12E+08	T	-	UTR3	TMEM245	NA	NA	NA	NA	NA
chr9	1.12E+08	1.12E+08	G	A	UTR3	TMEM245	NA	NA	NA	rs1044905	NA
chr9	1.16E+08	1.16E+08	G	T	UTR3	SNX30	NA	NA	NA	rs113416907	NA
chr9	1.16E+08	1.16E+08	T	-	UTR3	SNX30	NA	NA	NA	NA	NA
chr9	1.17E+08	1.17E+08	AA	-	UTR3	AKNA	NA	NA	NA	rs10610956	NA
chr9	1.17E+08	1.17E+08	G	A	UTR5	DFNB31	NA	NA	NA	rs2297814	NA
chr9	1.17E+08	1.17E+08	G	T	UTR5	DFNB31	NA	NA	NA	rs4527950	NA
chr9	1.17E+08	1.17E+08	C	T	UTR5	DFNB31	NA	NA	NA	rs10759714	NA
chr9	1.19E+08	1.19E+08	-	A	UTR3	PAPPA	NA	NA	NA	NA	NA
chr9	1.19E+08	1.19E+08	G	C	UTR5	TRIM32	NA	NA	NA	rs12342207	NA
chr9	1.26E+08	1.26E+08	G	A	UTR3	RABGAP1	NA	NA	NA	rs803718	NA
chr9	1.28E+08	1.28E+08	A	G	exonic	OLFML2A	nonsynonymous SNV	OLFML2A:NM_182487:exon6:c.A925G:p.T309A	NA	rs7874348	B
chr9	1.31E+08	1.31E+08	T	C	exonic	CERCAM	nonsynonymous SNV	CERCAM:NM_016174:exon5:c.T590C:p.F197S	NA	NA	P
chr9	1.31E+08	1.31E+08	A	G	exonic	CERCAM	synonymous SNV	CERCAM:NM_016174:exon5:c.A672G:p.E224E	NA	NA	NA
chr9	1.32E+08	1.32E+08	C	G	UTR3	PPP2R4	NA	NA	NA	NA	NA
chr9	1.32E+08	1.32E+08	C	G	UTR3	PPP2R4	NA	NA	NA	NA	NA
chr9	1.32E+08	1.32E+08	T	A	UTR3	PPP2R4	NA	NA	NA	NA	NA
chr9	1.33E+08	1.33E+08	A	-	UTR3	FNBP1	NA	NA	NA	rs5900873	NA
chr9	1.34E+08	1.34E+08	G	A	exonic	PRRC2B	synonymous SNV	PRRC2B:NM_013318:exon15:c.G4254A:p.K1418K	NA	rs78591540	NA
chr9	1.35E+08	1.35E+08	C	T	UTR3	NTNG2	NA	NA	NA	NA	NA
chr9	1.36E+08	1.36E+08	C	T	UTR3	ABO	NA	NA	NA	rs62641782	NA
chr9	1.36E+08	1.36E+08	T	G	UTR3	ABO	NA	NA	NA	rs199555421	NA
chr9	1.36E+08	1.36E+08	T	G	UTR3	ABO	NA	NA	NA	NA	NA
chr9	1.36E+08	1.36E+08	A	C	exonic	ABO	unknown	UNKNOWN	NA	rs688976	NA
chr9	1.36E+08	1.36E+08	TGTG	-	UTR3	CACFD1	NA	NA	NA	NA	NA
chr9	1.36E+08	1.36E+08	G	T	UTR5	ADAMTSL2	NA	NA	NA	NA	NA
chr9	1.38E+08	1.38E+08	T	C	exonic	OLFM1	synonymous SNV	NM_006334:exon2:c.T183C:p.A61A,OLFM1:NM_014279:exon2:c.T183(	NA	rs500461	NA
chr9	1.38E+08	1.38E+08	T	C	exonic	OBP2A	nonsynonymous SNV	OBP2A:NM_014582:exon6:c.T503C:p.L168P	NA	rs117518871	B
chr9	1.38E+08	1.38E+08	C	T	UTR5	PAEP	NA	NA	NA	rs3748205	NA

chr9	1.39E+08	1.39E+08	ACAC	-	UTR3	QSOX2	NA	NA	NA	rs146198713	NA
chr9	1.39E+08	1.39E+08	G	A	exonic	NOTCH1	synonymous SNV	NOTCH1:NM_017617:exon34:c.C6555T:p.D2185D	NA	rs2229974	NA
chr9	1.39E+08	1.39E+08	A	G	exonic	NOTCH1	synonymous SNV	NOTCH1:NM_017617:exon3:c.T312C:p.N104N	NA	rs4489420	NA
chr9	1.4E+08	1.4E+08	A	G	exonic	KIAA1984	nonsynonymous SNV	KIAA1984:NM_001039374:exon10:c.A1070G:p.E357G	NA	NA	P
chr9	1.41E+08	1.41E+08	A	T	exonic	CACNA1B	synonymous SNV	)00718:exon11:c.A1488T:p.T496T,CACNA1B:NM_001243812:exon11:c.	NA	rs2229951	NA
chr9	1.41E+08	1.41E+08	TG	-	UTR3	CACNA1B	NA	NA	NA	rs5901129	NA
chr9	1.41E+08	1.41E+08	T	C	ncRNA_exonic	TUBBP5	NA	NA	OSM1107244;OCCURENCE=1(endometı	rs62581042	NA
chr9	1.41E+08	1.41E+08	G	C	ncRNA_exonic	TUBBP5	NA	NA	D=COSM487278;OCCURENCE=1(kidney	NA	NA
chr10	1209495	1209495	G	C	ncRNA_exonic	LINC00200	NA	NA	NA	rs11250303	NA
chr10	5136651	5136651	C	G	exonic	AKR1C3	nonsynonymous SNV	NM_001253909:exon1:c.C15G:p.H5Q,AKR1C3:NM_003739:exon1:c.C1	NA	rs12529	NA
chr10	6053568	6053568	C	T	UTR3	IL2RA	NA	NA	NA	rs1570538	NA
chr10	6131411	6131411	-	TCC	UTR5	RBM17	NA	NA	NA	rs151234895	NA
chr10	6157836	6157839	AAAA	-	UTR3	RBM17	NA	NA	NA	rs71390125	NA
chr10	6159016	6159016	-	ATGCTGAAT	UTR3	RBM17	NA	NA	NA	NA	NA
chr10	6159018	6159018	T	A	UTR3	RBM17	NA	NA	NA	rs41295371	NA
chr10	6159018	6159018	-	ACAAGGACT	UTR3	RBM17	NA	NA	NA	NA	NA
chr10	8094861	8094861	G	-	ncRNA_exonic	GATA3-AS1	NA	NA	NA	rs35877776	NA
chr10	11375079	11375090	CCCTCTCA	-	UTR3	CELF2	NA	NA	NA	rs3841462	NA
chr10	11997298	11997298	G	T	exonic	UPF2	nonsynonymous SNV	_015542:exon14:c.C2783A:p.T928K,UPF2:NM_080599:exon14:c.C2783	NA	NA	D
chr10	12875917	12875917	A	-	ncRNA_exonic	LOC283070	NA	NA	NA	NA	NA
chr10	13252313	13252313	-	A	UTR3	MCM10	NA	NA	NA	NA	NA
chr10	17216556	17216556	C	T	exonic	TRDMT1	synonymous SNV	TRDMT1:NM_004412:exon2:c.G168A:p.T56T	NA	rs2273734	NA
chr10	18088648	18088648	A	G	intronic	MRC1	NA	NA	NA	rs142746617	NA
chr10	18088986	18088986	C	A	intronic	MRC1	NA	NA	NA	NA	NA
chr10	18689587	18689587	C	-	UTR5	CACNB2	NA	NA	NA	NA	NA
chr10	18830669	18830669	T	C	UTR3	CACNB2	NA	NA	NA	rs7079776	NA
chr10	21803421	21803421	-	A	UTR3	SKIDA1	NA	NA	NA	NA	NA
chr10	21807470	21807470	A	-	UTR5	SKIDA1	NA	NA	NA	rs111265769	NA
chr10	21807780	21807781	TT	-	UTR5	SKIDA1	NA	NA	NA	NA	NA
chr10	22825596	22825596	C	T	UTR3	PIP4K2A	NA	NA	NA	rs1048244	NA
chr10	23983777	23983777	G	T	UTR5	KIAA1217	NA	NA	NA	rs12772432	NA
chr10	25271270	25271270	G	A	UTR3	ENKUR	NA	NA	NA	rs2307047	NA
chr10	28908890	28908890	A	-	UTR3	WAC	NA	NA	NA	NA	NA
chr10	29801764	29801764	T	C	exonic	SVIL	nonsynonymous SNV	)03174:exon15:c.A2138G:p.E713G,SVIL:NM_021738:exon17:c.A3416G	NA	NA	D
chr10	31816340	31816340	-	AC	UTR3	ZEB1	NA	NA	NA	rs143911186	NA
chr10	38739199	38739199	A	G	ncRNA_exonic	LOC399744	NA	NA	NA	rs17611580	NA
chr10	45496046	45496046	C	A	exonic	C10orf25	stopgain SNV	C10orf25:NM_001039380:exon1:c.G262T:p.E88X	NA	NA	NA
chr10	45649377	45649377	A	C	ncRNA_exonic	RSU1P2	NA	NA	NA	rs11239368	NA
chr10	46248649	46248649	T	C	exonic	FAM21C	nonsynonymous SNV	2C:p.S358P,FAM21C:NM_001169106:exon13:c.T1144C:p.S382P,FAM21	NA	rs184073575	NA
chr10	46961964	46961964	G	A	UTR3	SYT15	NA	NA	NA	rs138447966	NA
chr10	46963951	46963951	G	A	exonic	SYT15	synonymous SNV	M_031912:exon7:c.C1012T:p.L338L,SYT15:NM_181519:exon7:c.C1012	NA	rs55945664	NA
chr10	47000508	47000508	G	A	UTR3	GPRIN2	NA	NA	NA	rs61840973	NA
chr10	47420856	47420856	G	A	ncRNA_exonic	FAM35DP	NA	NA	NA	rs1832163	NA
chr10	50310251	50310251	T	C	UTR3	VSTM4	NA	NA	NA	rs4240499	NA
chr10	50966445	50966445	C	A	exonic	OGDHL	nonsynonymous SNV	M_001143996:exon2:c.G194T:p.S65I,OGDHL:NM_018245:exon2:c.G1	NA	NA	D
chr10	51226439	51226439	T	G	exonic	AGAP8	nonsynonymous SNV	C:p.K181N,AGAP8:NM_001276344:exon7:c.A543C:p.K181N,AGAP8:NN	NA	rs201124655	B
chr10	51748584	51748584	A	G	exonic	AGAP6	nonsynonymous SNV	AGAP6:NM_001077665:exon1:c.A109G:p.R37G	NA	rs200504295	NA
chr10	60474828	60474828	A	-	ncRNA_exonic	FAM133CP	NA	NA	NA	NA	NA
chr10	62544601	62544601	G	T	exonic	CDK1	nonsynonymous SNV	NM_001170407:exon3:c.G176T:p.R59L,CDK1:NM_001786:exon3:c.G1	NA	NA	B
chr10	63955366	63955381	GAAGGAAG	-	UTR3	RTKN2	NA	NA	NA	NA	NA
chr10	70451264	70451264	A	C	exonic	TET1	nonsynonymous SNV	TET1:NM_030625:exon12:c.A6104C:p.H2035P	NA	NA	D
chr10	71267385	71267385	G	T	UTR3	TSPAN15	NA	NA	NA	NA	NA
chr10	72187716	72187716	T	-	UTR3	EIF4EBP2	NA	NA	NA	NA	NA
chr10	73059787	73059788	TG	-	UTR3	UNC5B	NA	NA	NA	NA	NA
chr10	73994765	73994765	T	C	UTR3	ANAPC16	NA	NA	NA	rs6480601	NA
chr10	79741920	79741920	T	G	exonic	POLR3A	nonsynonymous SNV	POLR3A:NM_007055:exon28:c.A3751C:p.T1251P	NA	NA	P
chr10	81114959	81114959	C	T	UTR3	PPIF	NA	NA	NA	rs7898	NA
chr10	81374986	81374986	T	C	UTR3	SFTPA1	NA	NA	NA	rs1059225	NA
chr10	88494752	88494752	A	-	UTR3	LDB3	NA	NA	NA	rs11314847	NA
chr10	88677045	88677045	T	A	exonic	BMPR1A	nonsynonymous SNV	BMPR1A:NM_004329:exon9:c.T830A:p.I277N	NA	NA	D
chr10	88810338	88810338	A	-	UTR3	GLUD1	NA	NA	NA	NA	NA
chr10	89125529	89125529	G	C	ncRNA_exonic	NUTM2D	NA	NA	NA	rs2767120	D
chr10	89127702	89127702	G	T	ncRNA_exonic	NUTM2D	NA	NA	NA	rs3128215	NA
chr10	89128214	89128214	G	A	ncRNA_exonic	NUTM2D	NA	NA	NA	rs77989216	NA
chr10	89129516	89129516	G	A	ncRNA_exonic	NUTM2D	NA	NA	NA	rs143548709	NA
chr10	89264593	89264593	C	G	UTR5	MINPP1	NA	NA	NA	rs2233166	NA

chr10	89619806	89619806	G	A	UTR3	KLLN	NA	NA	NA	NA	NA
chr10	89726612	89726612	G	A	UTR3	PTEN	NA	NA	NA	NA	NA
chr10	89726652	89726652	G	A	UTR3	PTEN	NA	NA	NA	NA	NA
chr10	99192863	99192863	-	ATAA	UTR3	PGAM1	NA	NA	NA	NA	NA
chr10	99192913	99192913	G	A	UTR3	PGAM1	NA	NA	NA	NA	NA
chr10	99435869	99435869	G	A	UTR3	PI4K2A	NA	NA	NA	rs7084287	NA
chr10	1.01E+08	1.01E+08	T	G	UTR5	COX15	NA	NA	NA	rs2281636	NA
chr10	1.02E+08	1.02E+08	GTGTGTGT	-	UTR3	HIF1AN	NA	NA	NA	NA	NA
chr10	1.02E+08	1.02E+08	G	T	UTR3	HIF1AN	NA	NA	NA	NA	NA
chr10	1.03E+08	1.03E+08	G	A	UTR3	SFXN3	NA	NA	NA	rs184783122	NA
chr10	1.04E+08	1.04E+08	C	T	exonic	MGEA5	nonsynonymous SNV	M_001142434:exon1:c.G151A:p.A51T,MGEA5:NM_012215:exon1:c.G1	NA	rs35402016	B
chr10	1.04E+08	1.04E+08	G	T	exonic	FBXL15	nonsynonymous SNV	FBXL15:NM_024326:exon4:c.G886T:p.V296F	NA	NA	P
chr10	1.05E+08	1.05E+08	T	G	UTR3	WBP1L	NA	NA	NA	rs284854	NA
chr10	1.05E+08	1.05E+08	C	T	exonic	CNNM2	synonymous SNV	I_199076:exon6:c.C2244T:p.A748A,CNNM2:NM_017649:exon7:c.C231	NA	rs943037	NA
chr10	1.05E+08	1.05E+08	A	T	UTR3	SH3PXD2A	NA	NA	NA	rs28516372	NA
chr10	1.12E+08	1.12E+08	A	-	UTR3	ADD3	NA	NA	NA	NA	NA
chr10	1.15E+08	1.15E+08	T	C	UTR5	HABP2	NA	NA	NA	rs2240879	NA
chr10	1.16E+08	1.16E+08	C	T	exonic	PLEKHS1	nonsynonymous SNV	.82601:exon10:c.C1024T:p.R342W,PLEKHS1:NM_001193434:exon11:c	NA	rs118170787	D
chr10	1.16E+08	1.16E+08	CCTTCCC	-	UTR3	PLEKHS1	NA	NA	NA	NA	NA
chr10	1.16E+08	1.16E+08	C	A	UTR3	PLEKHS1	NA	NA	NA	NA	NA
chr10	1.16E+08	1.16E+08	T	C	UTR3	PLEKHS1	NA	NA	NA	NA	NA
chr10	1.16E+08	1.16E+08	C	T	UTR3	ADRB1	NA	NA	NA	NA	NA
chr10	1.17E+08	1.17E+08	G	T	exonic	ATRNL1	nonsynonymous SNV	ATRNL1:NM_207303:exon19:c.G3175T:p.A1059S	NA	NA	B
chr10	1.18E+08	1.18E+08	-	TG	UTR3	GFRA1	NA	NA	NA	NA	NA
chr10	1.18E+08	1.18E+08	T	A	UTR3	GFRA1	NA	NA	NA	rs142941024	NA
chr10	1.18E+08	1.18E+08	T	A	UTR3	GFRA1	NA	NA	NA	rs145714787	NA
chr10	1.18E+08	1.18E+08	C	G	UTR3	GFRA1	NA	NA	NA	rs117805221	NA
chr10	1.2E+08	1.2E+08	-	A	UTR3	CACUL1	NA	NA	NA	NA	NA
chr10	1.21E+08	1.21E+08	C	A	UTR3	TIAL1	NA	NA	NA	rs111740135	NA
chr10	1.23E+08	1.23E+08	C	G	UTR5	FGFR2	NA	NA	NA	rs1047111	NA
chr10	1.27E+08	1.27E+08	T	A	UTR3	CTBP2	NA	NA	NA	NA	NA
chr10	1.27E+08	1.27E+08	T	A	UTR3	CTBP2	NA	NA	NA	NA	NA
chr10	1.27E+08	1.27E+08	A	G	UTR3	CTBP2	NA	NA	NA	rs76017768	NA
chr10	1.27E+08	1.27E+08	A	G	UTR3	CTBP2	NA	NA	NA	rs78209416	NA
chr10	1.27E+08	1.27E+08	T	C	UTR3	CTBP2	NA	NA	NA	rs75330281	NA
chr10	1.27E+08	1.27E+08	T	G	UTR3	CTBP2	NA	NA	NA	rs78608514	NA
chr10	1.27E+08	1.27E+08	A	C	UTR3	CTBP2	NA	NA	NA	NA	NA
chr10	1.27E+08	1.27E+08	G	C	UTR3	CTBP2	NA	NA	NA	rs3208596	NA
chr10	1.27E+08	1.27E+08	T	-	exonic	CTBP2	frameshift deletion	_001083914:exon3:c.57delA:p.E19fs,CTBP2:NM_001329:exon3:c.57de	NA	NA	NA
chr10	1.28E+08	1.28E+08	G	C	UTR5	FANK1	NA	NA	NA	rs80288044	NA
chr10	1.28E+08	1.28E+08	C	A	UTR5	FANK1	NA	NA	NA	rs74162870	NA
chr10	1.3E+08	1.3E+08	T	C	UTR3	FOXI2	NA	NA	NA	NA	NA
chr10	1.3E+08	1.3E+08	T	C	UTR3	FOXI2	NA	NA	NA	NA	NA
chr10	1.31E+08	1.31E+08	C	T	exonic	MGMT	synonymous SNV	MGMT:NM_002412:exon1:c.C66T:p.R22R	NA	rs16906252	NA
chr10	1.34E+08	1.34E+08	G	A	exonic	JAKMIP3	synonymous SNV	JAKMIP3:NM_001105521:exon1:c.G108A:p.Q36Q	NA	rs11146177	NA
chr10	1.34E+08	1.34E+08	A	G	exonic	DPYSL4	nonsynonymous SNV	DPYSL4:NM_006426:exon8:c.A773G:p.K258R	NA	NA	B
chr10	1.34E+08	1.34E+08	G	T	UTR3	LRRC27	NA	NA	NA	rs10870302	NA
chr10	1.34E+08	1.34E+08	A	T	UTR3	LRRC27	NA	NA	NA	NA	NA
chr10	1.34E+08	1.34E+08	C	T	UTR3	LRRC27	NA	NA	NA	NA	NA
chr10	1.35E+08	1.35E+08	A	G	exonic	GPR123	synonymous SNV	GPR123:NM_001083909:exon7:c.A834G:p.S278S	NA	rs2806452	NA
chr10	1.35E+08	1.35E+08	A	C	exonic	SYCE1	nonsynonymous SNV	M_001143763:exon1:c.T13G:p.S5A,SYCE1:NM_001143764:exon1:c.T1	NA	NA	NA
chr10	1.35E+08	1.35E+08	G	A	UTR5	FRG2B	NA	NA	NA	rs199872228	NA
chr11	203335	203335	A	G	UTR3	BET1L	NA	NA	NA	NA	NA
chr11	610277	610277	T	C	exonic	PHRF1	nonsynonymous SNV	PHRF1:NM_020901:exon15:c.T4343C:p.V1448A	NA	rs11246212	NA
chr11	621242	621242	C	T	exonic	CDHR5	synonymous SNV	627A:p.P209P,CDHR5:NM_021924:exon7:c.G627A:p.P209P,CDHR5:NM	NA	rs112269777	NA
chr11	1016559	1016559	G	C	exonic	MUC6	nonsynonymous SNV	MUC6:NM_005961:exon31:c.C6242G:p.A2081G	NA	NA	NA
chr11	1016756	1016756	G	A	exonic	MUC6	synonymous SNV	MUC6:NM_005961:exon31:c.C6045T:p.S2015S	NA	NA	NA
chr11	1016919	1016919	C	T	exonic	MUC6	nonsynonymous SNV	MUC6:NM_005961:exon31:c.G5882A:p.R1961K	NA	NA	NA
chr11	1016960	1016960	G	T	exonic	MUC6	synonymous SNV	MUC6:NM_005961:exon31:c.C5841A:p.T1947T	NA	rs113980831	NA
chr11	1017041	1017041	G	T	exonic	MUC6	stopgain SNV	MUC6:NM_005961:exon31:c.C5760A:p.Y1920X	NA	rs75482640	NA
chr11	1017042	1017042	T	G	exonic	MUC6	nonsynonymous SNV	MUC6:NM_005961:exon31:c.A5759C:p.Y1920S	NA	rs80049949	NA
chr11	1017068	1017068	C	T	exonic	MUC6	synonymous SNV	MUC6:NM_005961:exon31:c.G5733A:p.T1911T	NA	rs78992004	NA
chr11	1017277	1017277	A	G	exonic	MUC6	nonsynonymous SNV	MUC6:NM_005961:exon31:c.T5524C:p.S1842P	NA	rs111373859	NA
chr11	1017381	1017381	G	C	exonic	MUC6	nonsynonymous SNV	MUC6:NM_005961:exon31:c.C5420G:p.T1807S	COSM1180594;OCCURENCE=1(upper_a	rs34912894	NA
chr11	1017440	1017440	C	T	exonic	MUC6	synonymous SNV	MUC6:NM_005961:exon31:c.G5361A:p.S1787S	NA	rs78729877	NA
chr11	1017483	1017483	T	G	exonic	MUC6	nonsynonymous SNV	MUC6:NM_005961:exon31:c.A5318C:p.H1773P	NA	rs79986665	NA

chr11	1018048	1018048	A	T	exonic	MUC6	nonsynonymous SNV	MUC6:NM_005961:exon31:c.T4753A:p.F1585I	NA	rs12787400	NA
chr11	1018090	1018090	G	T	exonic	MUC6	nonsynonymous SNV	MUC6:NM_005961:exon31:c.C4711A:p.P1571T	NA	rs201615772	NA
chr11	1018290	1018290	G	A	exonic	MUC6	nonsynonymous SNV	MUC6:NM_005961:exon31:c.C4511T:p.P1504L	NA	NA	NA
chr11	1018341	1018341	G	A	exonic	MUC6	nonsynonymous SNV	MUC6:NM_005961:exon31:c.C4460T:p.P1487L	NA	rs79748612	NA
chr11	1088835	1088835	C	T	exonic	MUC2	nonsynonymous SNV	MUC2:NM_002457:exon26:c.C3620T:p.T1207I	NA	rs41389046	NA
chr11	1090928	1090928	A	C	exonic	MUC2	nonsynonymous SNV	MUC2:NM_002457:exon28:c.A3823C:p.T1275P	NA	NA	NA
chr11	1090937	1090937	A	C	exonic	MUC2	nonsynonymous SNV	MUC2:NM_002457:exon28:c.A3832C:p.T1278P	NA	NA	NA
chr11	1093452	1093452	G	A	exonic	MUC2	synonymous SNV	MUC2:NM_002457:exon30:c.G5271A:p.P1757P	1127915,COSM1127916;OCCURENCE=1	rs34136803	NA
chr11	1266630	1266630	G	A	exonic	MUC5B	synonymous SNV	MUC5B:NM_002458:exon31:c.G8520A:p.T2840T	NA	rs200570144	NA
chr11	1269500	1269500	T	C	exonic	MUC5B	nonsynonymous SNV	MUC5B:NM_002458:exon31:c.T11390C:p.I3797T	NA	NA	NA
chr11	1271763	1271763	A	C	exonic	MUC5B	synonymous SNV	MUC5B:NM_002458:exon31:c.A13653C:p.T4551T	52309,COSM924786;OCCURENCE=1(en	NA	NA
chr11	1575385	1575385	C	G	UTR3	DUSP8	NA	NA	NA	NA	NA
chr11	1605919	1605919	G	A	exonic	KRTAP5-1	synonymous SNV	KRTAP5-1:NM_001005922:exon1:c.C561T:p.C187C	NA	NA	NA
chr11	1606120	1606120	A	G	exonic	KRTAP5-1	synonymous SNV	KRTAP5-1:NM_001005922:exon1:c.T360C:p.G120G	NA	rs189753959	NA
chr11	1606166	1606166	G	C	exonic	KRTAP5-1	nonsynonymous SNV	KRTAP5-1:NM_001005922:exon1:c.C314G:p.S105C	NA	NA	NA
chr11	1643036	1643036	A	C	exonic	KRTAP5-4	synonymous SNV	KRTAP5-4:NM_001012709:exon1:c.T288G:p.G96G	NA	rs58877168	NA
chr11	1705485	1705485	T	C	ncRNA_exonic	FAM99B	NA	NA	NA	rs201023952	NA
chr11	2696063	2696063	A	-	ncRNA_exonic	KCNQ1OT1	NA	NA	NA	rs34219164	NA
chr11	2720927	2720927	C	T	ncRNA_exonic	KCNQ1OT1	NA	NA	NA	rs12360708	NA
chr11	3663224	3663224	G	A	UTR5	ART5	NA	NA	NA	rs115012337	NA
chr11	6550309	6550309	G	A	exonic	DNHD1	nonsynonymous SNV	DNHD1:NM_144666:exon12:c.G2305A:p.G769R	NA	rs2344829	NA
chr11	6566762	6566762	T	G	exonic	DNHD1	synonymous SNV	DNHD1:NM_144666:exon21:c.T4593G:p.G1531G	NA	NA	NA
chr11	6633033	6633033	A	C	exonic	TAF10	synonymous SNV	TAF10:NM_006284:exon2:c.T249G:p.G83G	NA	NA	NA
chr11	6634189	6634189	G	A	UTR3	TPP1	NA	NA	NA	rs7487	NA
chr11	7439264	7439264	C	T	exonic	SYT9	synonymous SNV	SYT9:NM_175733:exon5:c.C1242T:p.T414T	)=COSM147136;OCCURENCE=1(stomacl	rs34058874	NA
chr11	8126477	8126477	G	T	UTR3	TUB	NA	NA	NA	rs55774312	NA
chr11	8159892	8159892	G	T	exonic	RIC3	synonymous SNV	:354A:p.L118L,RIC3:NM_001206672:exon3:c.C354A:p.L118L,RIC3:NM_	NA	rs10839976	NA
chr11	9304488	9304488	A	-	UTR3	TMEM41B	NA	NA	NA	rs56313114	NA
chr11	11863526	11863526	G	T	UTR5	USP47	NA	NA	NA	rs28364721	NA
chr11	18287918	18287918	-	A	UTR5	SAA1	NA	NA	NA	rs200069063	NA
chr11	18477413	18477428	ACCTGCCA.	-	UTR5	LDHAL6A	NA	NA	NA	rs140456495	NA
chr11	21555994	21555994	C	A	exonic	NELL1	nonsynonymous SNV	NELL1:NM_006157:exon16:c.C1720A:p.H574N	NA	NA	D
chr11	26581383	26581384	AC	-	UTR3	MUC15	NA	NA	NA	NA	NA
chr11	26690764	26690764	T	C	UTR3	SLC5A12	NA	NA	NA	NA	NA
chr11	32875438	32875440	AGG	-	UTR3	PRRG4	NA	NA	NA	rs144258001	NA
chr11	33061642	33061642	G	-	UTR5	TCP11L1	NA	NA	NA	rs201592406	NA
chr11	33061647	33061647	A	T	UTR5	TCP11L1	NA	NA	NA	rs74889474	NA
chr11	33094974	33094974	T	G	UTR3	TCP11L1	NA	NA	NA	NA	NA
chr11	33097887	33097887	G	T	ncRNA_exonic	LINC00294	NA	NA	NA	NA	NA
chr11	33757941	33757941	A	G	UTR5	CD59	NA	NA	NA	rs13614	NA
chr11	35829803	35829803	G	A	UTR3	TRIM44	NA	NA	NA	NA	NA
chr11	45263072	45263072	A	C	UTR3	SYT13	NA	NA	NA	rs12283527	NA
chr11	46383244	46383244	C	A	UTR5	DGKZ	NA	NA	NA	NA	NA
chr11	56959046	56959046	T	G	UTR3	LRRC55	NA	NA	NA	NA	NA
chr11	56959052	56959052	T	G	UTR3	LRRC55	NA	NA	NA	NA	NA
chr11	59811098	59811098	G	T	exonic	PLAC1L	nonsynonymous SNV	PLAC1L:NM_173801:exon2:c.G221T:p.R74L	NA	NA	B
chr11	61560372	61560372	C	T	UTR5	FEN1	NA	NA	NA	NA	NA
chr11	61567669	61567669	T	-	UTR3	FADS1	NA	NA	NA	NA	NA
chr11	62295933	62295933	T	C	exonic	AHNAK	nonsynonymous SNV	AHNAK:NM_001620:exon5:c.A5956G:p.T1986A	NA	NA	B
chr11	64004692	64004692	C	T	exonic	VEGFB	synonymous SNV	_001243733:exon5:c.C408T:p.D136D,VEGFB:NM_003377:exon5:c.C40	)=COSM147267;OCCURENCE=1(stomacl	rs12366035	NA
chr11	64578162	64578162	A	G	UTR5	MEN1	NA	NA	NA	NA	NA
chr11	64806270	64806270	C	T	exonic	SNX15	synonymous SNV	SNX15:NM_013306:exon7:c.C891T:p.D297D	NA	rs542175	NA
chr11	65143541	65143543	AAA	-	UTR3	SLC25A45	NA	NA	NA	NA	NA
chr11	65563503	65563503	A	T	UTR3	OVOL1	NA	NA	NA	rs67300439	NA
chr11	66393069	66393069	G	C	exonic	RBM14	synonymous SNV	RBM14:NM_006328:exon2:c.G1722C:p.P574P	NA	NA	NA
chr11	67052439	67052439	C	G	exonic	ADRBK1	synonymous SNV	ADRBK1:NM_001619:exon19:c.C1776G:p.G592G	NA	NA	NA
chr11	68663993	68663993	C	T	exonic	MRPL21	nonsynonymous SNV	M_181514:exon4:c.G386A:p.R129Q,MRPL21:NM_181515:exon4:c.G13	NA	rs143584803	D
chr11	68856916	68856916	C	T	UTR3	TPCN2	NA	NA	NA	rs1005858	NA
chr11	70281360	70281362	TTT	-	UTR3	CTTN	NA	NA	NA	rs71992777	NA
chr11	70281395	70281395	A	G	UTR3	CTTN	NA	NA	NA	NA	NA
chr11	70666765	70666765	C	T	exonic	SHANK2	unknown	UNKNOWN	NA	rs11237599	NA
chr11	71238668	71238668	G	A	exonic	KRTAP5-7	nonsynonymous SNV	KRTAP5-7:NM_001012503:exon1:c.G322A:p.G108R	NA	NA	NA
chr11	71238669	71238669	G	A	exonic	KRTAP5-7	nonsynonymous SNV	KRTAP5-7:NM_001012503:exon1:c.G323A:p.G108E	NA	NA	NA
chr11	71238760	71238760	A	C	exonic	KRTAP5-7	synonymous SNV	KRTAP5-7:NM_001012503:exon1:c.A414C:p.S138S	NA	NA	NA
chr11	71498591	71498591	C	G	exonic	FAM86C1	synonymous SNV	n1:c.C9G:p.P3P,FAM86C1:NM_018172:exon1:c.C9G:p.P3P,FAM86C1:N	NA	rs12270466	NA
chr11	71498601	71498601	G	T	exonic	FAM86C1	nonsynonymous SNV	l:c.G19T:p.A7S,FAM86C1:NM_018172:exon1:c.G19T:p.A7S,FAM86C1:I	NA	rs12283300	P

chr11	71708250	71708251	GT	-	UTR3	RNF121	NA	NA	NA	rs3841467	NA
chr11	71715015	71715015	-	G	exonic	NUMA1	frameshift insertion	NUMA1:NM_006185:exon26:c.6254_6255insC:p.R2085fs	NA	NA	NA
chr11	77811990	77811990	T	C	ncRNA_UTR3	ALG8	NA	NA	NA	rs1263505	NA
chr11	85968623	85968623	C	T	exonic	EED	synonymous SNV	IM_003797:exon6:c.C619T:p.L207L,EED:NM_152991:exon6:c.C619T:p.	NA	rs974144	NA
chr11	89537461	89537461	G	T	exonic	TRIM49	synonymous SNV	TRIM49:NM_020358:exon3:c.C177A:p.T59T	NA	rs585471	NA
chr11	89701844	89701844	T	C	exonic	TRIM64	nonsynonymous SNV	TRIM64:NM_001136486:exon1:c.T173C:p.I58T	NA	rs145750208	NA
chr11	89727291	89727291	A	G	ncRNA_exonic	TRIM53AP	NA	NA	NA	rs200877642	NA
chr11	89732714	89732714	C	G	ncRNA_exonic	TRIM53AP	NA	NA	NA	rs201822208	NA
chr11	93170919	93170919	A	C	UTR3	CCDC67	NA	NA	NA	NA	NA
chr11	93170925	93170925	G	A	UTR3	CCDC67	NA	NA	NA	rs2605584	NA
chr11	94134513	94134513	G	C	UTR5	GPR83	NA	NA	NA	rs12791316	NA
chr11	94803548	94803548	G	A	UTR3	SRSF8	NA	NA	NA	rs201925344	NA
chr11	94863488	94863488	-	T	UTR3	ENDOD1	NA	NA	NA	NA	NA
chr11	96126289	96126290	TT	-	UTR3	JRKL	NA	NA	NA	NA	NA
chr11	1.01E+08	1.01E+08	T	-	UTR3	PGR	NA	NA	NA	NA	NA
chr11	1.01E+08	1.01E+08	C	T	UTR3	PGR	NA	NA	NA	rs11224560	NA
chr11	1.01E+08	1.01E+08	T	G	exonic	PGR	nonsynonymous SNV	3C:p.T430P,PGR:NM_001202474:exon1:c.A796C:p.T266P,PGR:NM_001	NA	NA	B
chr11	1.01E+08	1.01E+08	T	G	exonic	PGR	synonymous SNV	3C:p.P421P,PGR:NM_001202474:exon1:c.A771C:p.P257P,PGR:NM_001	NA	NA	NA
chr11	1.02E+08	1.02E+08	T	A	UTR3	KIAA1377	NA	NA	NA	NA	NA
chr11	1.06E+08	1.06E+08	A	C	UTR3	GRIA4	NA	NA	NA	rs188861527	NA
chr11	1.08E+08	1.08E+08	A	C	UTR3	SLC35F2	NA	NA	NA	rs111576438	NA
chr11	1.08E+08	1.08E+08	C	G	UTR3	RAB39A	NA	NA	NA	rs1027733	NA
chr11	1.08E+08	1.08E+08	T	-	UTR3	CUL5	NA	NA	NA	rs67141256	NA
chr11	1.08E+08	1.08E+08	A	G	exonic	EXPH5	nonsynonymous SNV	EXPH5:NM_015065:exon1:c.T71C:p.V24A	NA	NA	D
chr11	1.12E+08	1.12E+08	G	T	UTR3	C11orf57	NA	NA	NA	NA	NA
chr11	1.13E+08	1.13E+08	C	T	UTR3	NCAM1	NA	NA	NA	NA	NA
chr11	1.18E+08	1.18E+08	CA	-	UTR3	FXYD6	NA	NA	NA	rs57385031	NA
chr11	1.18E+08	1.18E+08	-	T	ncRNA_UTR3	UBE4A	NA	NA	NA	NA	NA
chr11	1.19E+08	1.19E+08	T	C	UTR3	DDX6	NA	NA	NA	NA	NA
chr11	1.19E+08	1.19E+08	A	C	UTR3	DDX6	NA	NA	NA	NA	NA
chr11	1.19E+08	1.19E+08	G	A	UTR3	DDX6	NA	NA	NA	rs76362677	NA
chr11	1.2E+08	1.2E+08	T	C	UTR3	POU2F3	NA	NA	NA	rs78221919	NA
chr11	1.2E+08	1.2E+08	C	T	UTR3	POU2F3	NA	NA	NA	rs4938797	NA
chr11	1.2E+08	1.2E+08	C	T	UTR3	POU2F3	NA	NA	NA	rs200901486	NA
chr11	1.22E+08	1.22E+08	-	A	ncRNA_exonic	MIR100HG	NA	NA	NA	NA	NA
chr11	1.26E+08	1.26E+08	A	G	UTR3	CDON	NA	NA	NA	rs58515733	NA
chr11	1.26E+08	1.26E+08	A	T	UTR3	CDON	NA	NA	NA	rs58251767	NA
chr11	1.26E+08	1.26E+08	C	A	exonic	CDON	stopgain SNV	_001243597:exon6:c.G928T:p.E310X,CDON:NM_016952:exon6:c.G928	NA	NA	NA
chr11	1.29E+08	1.29E+08	C	T	exonic	C11orf45	synonymous SNV	_001256088:exon4:c.G438A:p.X146X,C11orf45:NM_145013:exon4:c.G	NA	rs11221503	NA
chr11	1.29E+08	1.29E+08	TGTTTGTT	-	UTR3	KCNJ5	NA	NA	NA	rs36205004	NA
chr11	1.3E+08	1.3E+08	C	T	UTR3	ZBTB44	NA	NA	NA	NA	NA
chr11	1.34E+08	1.34E+08	T	C	intronic	IGSF9B	NA	NA	NA	rs12800905	NA
chr12	498268	498269	GG	-	UTR5	KDM5A	NA	NA	NA	NA	NA
chr12	863076	863076	T	C	exonic	WNK1	synonymous SNV	NM_014823:exon1:c.T345C:p.A115A,WNK1:NM_018979:exon1:c.T345	NA	NA	NA
chr12	1021912	1021912	C	-	UTR3	RAD52	NA	NA	NA	rs71441627	NA
chr12	4873163	4873163	G	T	exonic	GALNT8	nonsynonymous SNV	GALNT8:NM_017417:exon9:c.G1543T:p.V515F	=COSM1179986;OCCURENCE=1(prostat	rs1468556	P
chr12	5021439	5021439	G	T	exonic	KCNA1	nonsynonymous SNV	KCNA1:NM_000217:exon2:c.G895T:p.V299F	NA	NA	D
chr12	6499908	6499908	A	G	exonic	LTBR	synonymous SNV	01270987:exon10:c.A1056G:p.G352G,LTBR:NM_002342:exon10:c.A111	NA	NA	NA
chr12	6679324	6679324	T	-	UTR3	CHD4	NA	NA	NA	NA	NA
chr12	6775801	6775801	A	-	UTR3	ZNF384	NA	NA	NA	NA	NA
chr12	6858126	6858126	G	A	exonic	MLF2	synonymous SNV	MLF2:NM_005439:exon8:c.C582T:p.D194D	=COSM431733;OCCURENCE=1(pancrea	rs2302371	NA
chr12	7947875	7947877	TTT	-	UTR3	NANOG	NA	NA	NA	rs4012936	NA
chr12	8353374	8353374	G	A	ncRNA_exonic	FAM66C	NA	NA	NA	rs200171719	NA
chr12	8353403	8353403	A	G	ncRNA_exonic	FAM66C	NA	NA	NA	rs200301637	NA
chr12	8927168	8927168	C	T	UTR3	RIMKLB	NA	NA	NA	NA	NA
chr12	9447488	9447488	C	T	ncRNA_exonic	LOC642846	NA	NA	NA	rs61916250	NA
chr12	9571764	9571764	T	G	ncRNA_exonic	DDX12P	NA	NA	D=COSM469094;OCCURENCE=1(kidney	rs117821635	NA
chr12	9585598	9585598	T	C	ncRNA_exonic	DDX12P	NA	NA	NA	rs200479049	NA
chr12	9800675	9800675	-	CC	ncRNA_exonic	LOC374443	NA	NA	NA	NA	NA
chr12	9800777	9800777	T	G	ncRNA_exonic	LOC374443	NA	NA	NA	NA	NA
chr12	9849145	9849145	A	-	UTR3	CLEC2D	NA	NA	NA	rs112075912	NA
chr12	9885707	9885707	-	TAAGT	exonic	CLECL1	frameshift insertion	p.S52fs,CLECL1:NM_001267701:exon1:c.154_155insACTTA:p.S52fs,CLE	NA	rs112979434	NA
chr12	10583696	10583696	C	T	UTR3	KLRC2	NA	NA	NA	rs2981593	NA
chr12	11083356	11083356	A	G	exonic	PRH2	nonsynonymous SNV	_001110213:exon3:c.A196G:p.N66D,PRH2:NM_005042:exon3:c.A196	NA	rs1049112	NA
chr12	11420721	11420721	A	G	exonic	PRB3	synonymous SNV	PRB3:NM_006249:exon3:c.T462C:p.G154G	NA	NA	NA
chr12	11420895	11420895	C	T	exonic	PRB3	synonymous SNV	PRB3:NM_006249:exon3:c.G288A:p.P96P	NA	NA	NA

chr12	11421004	11421004	C	G	exonic	PRB3	nonsynonymous SNV	PRB3:NM_006249:exon3:c.G179C:p.R60P	NA	rs71455364	NA
chr12	11461440	11461440	T	C	exonic	PRB4	synonymous SNV	PRB4:NM_002723:exon3:c.A477G:p.P159P	NA	NA	NA
chr12	11461444	11461444	T	G	exonic	PRB4	nonsynonymous SNV	PRB4:NM_002723:exon3:c.A473C:p.H158P	NA	rs59774427	NA
chr12	11461722	11461722	T	G	exonic	PRB4	synonymous SNV	1_001261399:exon3:c.A195C:p.G65G,PRB4:NM_002723:exon3:c.A195(	NA	rs78020621	NA
chr12	11461738	11461738	G	C	exonic	PRB4	nonsynonymous SNV	/I_001261399:exon3:c.C179G:p.P60R,PRB4:NM_002723:exon3:c.C179(	NA	rs79562958	NA
chr12	11546192	11546192	G	A	exonic	PRB2	nonsynonymous SNV	PRB2:NM_006248:exon3:c.C820T:p.P274S	NA	rs10845349	NA
chr12	11546258	11546258	G	T	exonic	PRB2	nonsynonymous SNV	PRB2:NM_006248:exon3:c.C754A:p.Q252K	NA	NA	NA
chr12	12045145	12045146	TT	-	ncRNA_UTR3	ETV6	NA	NA	NA	NA	NA
chr12	19529083	19529083	A	-	UTR3	PLEKHA5	NA	NA	NA	NA	NA
chr12	22839786	22839793	TTTTTTTT	-	UTR3	ETNK1	NA	NA	NA	rs57569708	NA
chr12	24967769	24967769	C	T	UTR3	BCAT1	NA	NA	NA	NA	NA
chr12	26386265	26386268	ATAT	-	UTR3	SSPN	NA	NA	NA	NA	NA
chr12	27848053	27848053	-	T	UTR3	PPFIBP1	NA	NA	NA	NA	NA
chr12	29604392	29604392	C	G	exonic	OVCH1	nonsynonymous SNV	OVCH1:NM_183378:exon22:c.G2641C:p.A881P	NA	rs1347570	NA
chr12	31648826	31648826	C	T	exonic	DENND5B	nonsynonymous SNV	DENND5B:NM_144973:exon2:c.G155A:p.R52K	NA	rs4930979	NA
chr12	38719971	38719971	-	ATTTT	UTR3	ALG10B	NA	NA	NA	rs141976317	NA
chr12	39047480	39047480	C	A	UTR3	CPNE8	NA	NA	NA	NA	NA
chr12	39687535	39687535	-	A	UTR3	KIF21A	NA	NA	NA	rs34373131	NA
chr12	40742254	40742254	G	A	exonic	LRRK2	synonymous SNV	LRRK2:NM_198578:exon43:c.G6324A:p.E2108E	NA	rs10878405	NA
chr12	43860526	43860526	A	G	exonic	ADAMTS20	synonymous SNV	ADAMTS20:NM_025003:exon9:c.T1296C:p.P432P	NA	rs11182088	NA
chr12	49315300	49315300	-	T	UTR3	CCDC65	NA	NA	NA	NA	NA
chr12	50745739	50745739	A	G	exonic	FAM186A	nonsynonymous SNV	FAM186A:NM_001145475:exon4:c.T4876C:p.S1626P	NA	NA	NA
chr12	50746158	50746158	G	T	exonic	FAM186A	nonsynonymous SNV	FAM186A:NM_001145475:exon4:c.C4457A:p.P1486Q	ID=COSM229789;OCCURENCE=1(skin)	NA	NA
chr12	50746280	50746280	C	G	exonic	FAM186A	nonsynonymous SNV	FAM186A:NM_001145475:exon4:c.G4335C:p.M1445I	NA	NA	NA
chr12	50746320	50746320	T	C	exonic	FAM186A	nonsynonymous SNV	FAM186A:NM_001145475:exon4:c.A4295G:p.E1432G	NA	NA	NA
chr12	50746496	50746496	C	G	exonic	FAM186A	nonsynonymous SNV	FAM186A:NM_001145475:exon4:c.G4119C:p.M1373I	NA	NA	NA
chr12	50746603	50746603	T	G	exonic	FAM186A	nonsynonymous SNV	FAM186A:NM_001145475:exon4:c.A4012C:p.T1338P	NA	NA	NA
chr12	50746608	50746608	T	C	exonic	FAM186A	nonsynonymous SNV	FAM186A:NM_001145475:exon4:c.A4007G:p.E1336G	NA	NA	NA
chr12	50746771	50746771	T	G	exonic	FAM186A	nonsynonymous SNV	FAM186A:NM_001145475:exon4:c.A3844C:p.K1282Q	NA	NA	NA
chr12	50746927	50746927	T	G	exonic	FAM186A	nonsynonymous SNV	FAM186A:NM_001145475:exon4:c.A3688C:p.T1230P	NA	NA	NA
chr12	51891648	51891648	C	A	intronic	SLC4A8	NA	NA	NA	NA	NA
chr12	52374856	52374856	T	G	exonic	ACVR1B	synonymous SNV	4G:p.G228G,ACVR1B:NM_020327:exon4:c.T528G:p.G176G,ACVR1B:NM	NA	NA	NA
chr12	52627215	52627215	A	G	exonic	KRT7	synonymous SNV	KRT7:NM_005556:exon1:c.A135G:p.S45S	NA	rs7308888	NA
chr12	52845601	52845601	C	T	exonic	KRT6B	nonsynonymous SNV	KRT6B:NM_005555:exon1:c.G262A:p.G88R	NA	rs201122967	NA
chr12	52845665	52845665	G	T	exonic	KRT6B	synonymous SNV	KRT6B:NM_005555:exon1:c.C198A:p.G66G	NA	rs141114189	NA
chr12	53162729	53162729	A	C	exonic	KRT76	nonsynonymous SNV	KRT76:NM_015848:exon9:c.T1685G:p.V562G	NA	NA	NA
chr12	53162773	53162773	A	G	exonic	KRT76	synonymous SNV	KRT76:NM_015848:exon9:c.T1641C:p.S547S	NA	rs1464423	NA
chr12	53810134	53810135	TT	-	UTR3	SP1	NA	NA	NA	NA	NA
chr12	54427251	54427251	T	G	exonic	HOXC5	nonsynonymous SNV	HOXC5:NM_018953:exon1:c.T345G:p.S115R	NA	NA	P
chr12	54514236	54514236	G	C	ncRNA_exonic	FLJ12825	NA	NA	NA	rs199990556	NA
chr12	54514262	54514262	C	T	ncRNA_exonic	FLJ12825	NA	NA	NA	rs200768860	NA
chr12	54514291	54514291	G	C	ncRNA_exonic	FLJ12825	NA	NA	NA	rs75934050	NA
chr12	54943322	54943322	A	G	UTR5	PDE1B	NA	NA	NA	NA	NA
chr12	54943340	54943340	A	G	UTR5	PDE1B	NA	NA	NA	NA	NA
chr12	54943354	54943354	A	G	UTR5	PDE1B	NA	NA	NA	NA	NA
chr12	56430860	56430860	-	G	UTR3	IKZF4	NA	NA	NA	NA	NA
chr12	56810470	56810470	G	T	UTR3	TIMELESS	NA	NA	NA	NA	NA
chr12	57487230	57487230	C	A	exonic	NAB2	nonsynonymous SNV	NAB2:NM_005967:exon6:c.C1317A:p.D439E	NA	NA	NA
chr12	57648285	57648285	A	-	UTR3	R3HDM2	NA	NA	NA	rs67514723	NA
chr12	58146158	58146158	G	T	UTR5	CDK4	NA	NA	NA	NA	NA
chr12	58146161	58146161	G	T	UTR5	CDK4	NA	NA	NA	NA	NA
chr12	62988922	62988922	T	-	UTR3	MON2	NA	NA	NA	NA	NA
chr12	62997466	62997466	C	A	ncRNA_exonic	MIRLET7I	NA	NA	NA	NA	NA
chr12	64541157	64541157	A	C	UTR3	SRGAP1	NA	NA	NA	NA	NA
chr12	64541171	64541171	A	C	UTR3	SRGAP1	NA	NA	NA	NA	NA
chr12	64541205	64541205	A	C	UTR3	SRGAP1	NA	NA	NA	NA	NA
chr12	66218517	66218517	C	G	ncRNA_UTR5	HMGA2	NA	NA	NA	rs7965172	NA
chr12	66218519	66218519	C	G	ncRNA_UTR5	HMGA2	NA	NA	NA	rs12425965	NA
chr12	70214497	70214497	G	T	UTR3	RAB3IP	NA	NA	NA	rs11177881	NA
chr12	71519096	71519096	G	C	UTR3	TSPAN8	NA	NA	NA	rs1051344	NA
chr12	72079926	72079926	T	G	UTR5	TMEM19	NA	NA	NA	NA	NA
chr12	88591037	88591038	TT	-	UTR3	TMTC3	NA	NA	NA	NA	NA
chr12	89914880	89914880	A	-	UTR3	GALNT4,POC1B-GALNT4	NA	NA	NA	NA	NA
chr12	89914886	89914886	A	T	UTR3	GALNT4,POC1B-GALNT4	NA	NA	NA	rs10745507	NA
chr12	95473723	95473723	T	-	UTR3	FGD6	NA	NA	NA	NA	NA
chr12	96361580	96361580	G	T	exonic	AMDHD1	stopgain SNV	AMDHD1:NM_152435:exon9:c.G1240T:p.E414X	NA	NA	NA

chr12	96367601	96367601	-	AAA	UTR3	HAL	NA	NA	NA	NA	NA
chr12	1.01E+08	1.01E+08	-	T	UTR3	SLC17A8	NA	NA	NA	NA	NA
chr12	1.02E+08	1.02E+08	-	A	UTR3	MYBPC1	NA	NA	NA	NA	NA
chr12	1.04E+08	1.04E+08	T	G	exonic	C12orf42	nonsynonymous SNV	_001099336:exon6:c.A787C:p.S263R,C12orf42:NM_198521:exon6:c.A	NA	rs79387839	NA
chr12	1.04E+08	1.04E+08	C	T	exonic	STAB2	synonymous SNV	STAB2:NM_017564:exon51:c.C5307T:p.I1769I	NA	rs117118859	NA
chr12	1.04E+08	1.04E+08	-	A	UTR3	NT5DC3	NA	NA	NA	rs11428514	NA
chr12	1.04E+08	1.04E+08	C	A	exonic	TDG	synonymous SNV	TDG:NM_003211:exon3:c.C387A:p.T129T	22020;OCCURENCE=1(upper_aerodiges	rs75200561	NA
chr12	1.07E+08	1.07E+08	T	-	UTR3	CKAP4	NA	NA	NA	rs71442016	NA
chr12	1.07E+08	1.07E+08	T	-	UTR3	C12orf23	NA	NA	NA	rs57234871	NA
chr12	1.08E+08	1.08E+08	A	C	exonic	BTBD11	nonsynonymous SNV	)1017523:exon5:c.A545C:p.H182P,BTBD11:NM_001018072:exon7:c.A	NA	rs76689600	D
chr12	1.09E+08	1.09E+08	C	G	exonic	TMEM119	synonymous SNV	TMEM119:NM_181724:exon2:c.G180C:p.P60P	NA	NA	NA
chr12	1.09E+08	1.09E+08	A	G	exonic	TMEM119	nonsynonymous SNV	TMEM119:NM_181724:exon2:c.T172C:p.W58R	NA	NA	B
chr12	1.09E+08	1.09E+08	T	G	exonic	TMEM119	synonymous SNV	TMEM119:NM_181724:exon2:c.A168C:p.P56P	NA	NA	NA
chr12	1.09E+08	1.09E+08	C	G	exonic	TMEM119	synonymous SNV	TMEM119:NM_181724:exon2:c.G165C:p.P55P	NA	NA	NA
chr12	1.09E+08	1.09E+08	C	G	exonic	TMEM119	synonymous SNV	TMEM119:NM_181724:exon2:c.G156C:p.P52P	NA	NA	NA
chr12	1.1E+08	1.1E+08	CA	-	UTR3	MMAB	NA	NA	NA	NA	NA
chr12	1.1E+08	1.1E+08	A	-	UTR3	GLTP	NA	NA	NA	rs112967311	NA
chr12	1.12E+08	1.12E+08	A	T	UTR3	NAA25	NA	NA	NA	rs58973750	NA
chr12	1.13E+08	1.13E+08	TGA	-	UTR3	PTPN11	NA	NA	NA	rs148422684	NA
chr12	1.13E+08	1.13E+08	AC	-	UTR3	RPH3A	NA	NA	NA	rs10563429	NA
chr12	1.14E+08	1.14E+08	T	G	exonic	TPCN1	nonsynonymous SNV	17901:exon18:c.T1463G:p.V488G,TPCN1:NM_001143819:exon19:c.T1(	NA	NA	P
chr12	1.18E+08	1.18E+08	TGCTCTGC	-	UTR3	KSR2	NA	NA	NA	NA	NA
chr12	1.2E+08	1.2E+08	G	A	UTR3	CIT	NA	NA	NA	rs139790082	NA
chr12	1.21E+08	1.21E+08	G	A	UTR3	MSI1	NA	NA	NA	NA	NA
chr12	1.21E+08	1.21E+08	C	T	UTR3	MSI1	NA	NA	NA	NA	NA
chr12	1.22E+08	1.22E+08	C	A	UTR3	P2RX7	NA	NA	NA	rs79099474	NA
chr12	1.22E+08	1.22E+08	A	C	exonic	CAMKK2	nonsynonymous SNV	CAMKK2:NM_001270486:exon16:c.T1667G:p.V556G	NA	NA	NA
chr12	1.23E+08	1.23E+08	-	AT	UTR3	DENR	NA	NA	NA	rs34625739	NA
chr12	1.24E+08	1.24E+08	C	A	UTR3	SETD8	NA	NA	NA	rs80048501	NA
chr12	1.24E+08	1.24E+08	A	T	UTR3	SETD8	NA	NA	NA	NA	NA
chr12	1.24E+08	1.24E+08	C	T	UTR3	SETD8	NA	NA	NA	rs111418154	NA
chr12	1.25E+08	1.25E+08	T	C	exonic	UBC	synonymous SNV	UBC:NM_021009:exon2:c.A1644G:p.E548E	NA	rs1071722	NA
chr12	1.29E+08	1.29E+08	G	T	UTR3	TMEM132C	NA	NA	NA	NA	NA
chr12	1.29E+08	1.29E+08	G	A	intronic	SLC15A4	NA	NA	NA	rs200176633	NA
chr12	1.31E+08	1.31E+08	-	AAA	ncRNA_exonic	LOC100190940	NA	NA	NA	rs34472041	NA
chr12	1.33E+08	1.33E+08	G	A	exonic	FBRSL1	nonsynonymous SNV	FBRSL1:NM_001142641:exon3:c.G521A:p.R174Q	NA	rs186991682	NA
chr12	1.33E+08	1.33E+08	T	C	UTR3	P2RX2	NA	NA	NA	rs7973007	NA
chr12	1.33E+08	1.33E+08	C	A	exonic	ANKLE2	nonsynonymous SNV	ANKLE2:NM_015114:exon13:c.G2619T:p.W873C	NA	NA	D
chr12	1.34E+08	1.34E+08	A	C	UTR3	ZNF268	NA	NA	NA	NA	NA
chr12	1.34E+08	1.34E+08	A	C	UTR3	ZNF268	NA	NA	NA	NA	NA
chr13	19419783	19419783	C	G	ncRNA_exonic	ANKRD20A9P	NA	NA	NA	rs74692341	NA
chr13	19419808	19419808	G	A	ncRNA_exonic	ANKRD20A9P	NA	NA	NA	rs73435804	NA
chr13	19419847	19419847	C	A	ncRNA_exonic	ANKRD20A9P	NA	NA	NA	rs74349552	NA
chr13	19436148	19436148	C	G	ncRNA_exonic	ANKRD20A9P	NA	NA	NA	rs112815656	NA
chr13	19436179	19436179	A	G	ncRNA_exonic	ANKRD20A9P	NA	NA	NA	rs112011321	NA
chr13	22275849	22275850	TG	-	UTR3	FGF9	NA	NA	NA	NA	NA
chr13	25272870	25272870	C	T	exonic	ATP12A	synonymous SNV	_001185085:exon12:c.C1605T:p.I535I,ATP12A:NM_001676:exon12:c.C	NA	rs2289901	NA
chr13	25875755	25875755	A	C	UTR5	NUPL1	NA	NA	NA	NA	NA
chr13	27847812	27847812	G	T	UTR3	RASL11A	NA	NA	NA	NA	NA
chr13	28874659	28874664	CACACA	-	UTR3	FLT1	NA	NA	NA	rs147176157	NA
chr13	28875310	28875310	A	-	UTR3	FLT1	NA	NA	NA	rs201743756	NA
chr13	31035091	31035092	TT	-	UTR3	HMGB1	NA	NA	NA	NA	NA
chr13	31904677	31904677	-	T	UTR3	B3GALTL	NA	NA	NA	NA	NA
chr13	33349235	33349235	-	TT	UTR3	PDS5B	NA	NA	NA	NA	NA
chr13	33351489	33351489	C	T	UTR3	PDS5B	NA	NA	NA	rs202241635	NA
chr13	33351489	33351489	-	TTT	UTR3	PDS5B	NA	NA	NA	NA	NA
chr13	36744910	36744910	C	T	exonic	CCDC169-SOHLH2,SOHLH2	nonsynonymous SNV	5:exon10:c.G1015A:p.A339T,CCDC169-SOHLH2:NM_001198910:exon1	NA	rs2296968	B
chr13	36876379	36876379	A	G	UTR3	SPG20	NA	NA	NA	rs78129833	NA
chr13	41837625	41837625	G	A	UTR5	MTRF1	NA	NA	NA	NA	NA
chr13	43681661	43681662	AC	-	UTR3	DNAJC15	NA	NA	NA	NA	NA
chr13	49852512	49852512	G	T	exonic	CDADC1	nonsynonymous SNV	001193478:exon7:c.G1077T:p.M359I,CDADC1:NM_030911:exon7:c.G1	NA	NA	D
chr13	58209060	58209060	T	C	exonic	PCDH17	nonsynonymous SNV	PCDH17:NM_001040429:exon1:c.T2380C:p.S794P	NA	NA	D
chr13	66878392	66878392	C	A	UTR3	PCDH9	NA	NA	NA	rs200806980	NA
chr13	75861158	75861158	C	A	exonic	TBC1D4	nonsynonymous SNV	TBC1D4:NM_014832:exon21:c.G3667T:p.A1223S	NA	NA	B
chr13	96485306	96485306	C	A	exonic	UGGT2	nonsynonymous SNV	UGGT2:NM_020121:exon38:c.G4403T:p.C1468F	NA	NA	D
chr13	1.01E+08	1.01E+08	-	T	UTR3	ZIC2	NA	NA	NA	rs34544768	NA

chr13	1.01E+08	1.01E+08	G	C	UTR5	TMTC4	NA	NA	NA	rs1283201	NA
chr13	1.04E+08	1.04E+08	-	T	ncRNA_exonic	METTL21EP	NA	NA	NA	rs34557658	NA
chr13	1.12E+08	1.12E+08	A	-	UTR3	ARHGEF7	NA	NA	NA	rs11319592	NA
chr13	1.14E+08	1.14E+08	C	A	exonic	F10	nonsynonymous SNV	F10:NM_000504:exon1:c.C7A:p.R3S	NA	NA	P
chr13	1.15E+08	1.15E+08	G	C	exonic	UPF3A	nonsynonymous SNV	NM_023011:exon2:c.G208C:p.V70L,UPF3A:NM_080687:exon2:c.G208	OCCURENCE=2(skin),1(lung),1(upper_ae	rs76186578	D
chr14	21965677	21965677	G	T	UTR3	TOX4	NA	NA	NA	rs3929984	NA
chr14	22102293	22102293	C	T	exonic	OR10G2	nonsynonymous SNV	OR10G2:NM_001005466:exon1:c.G706A:p.A236T	NA	rs41307110	B
chr14	23235808	23235808	G	A	exonic	OXA1L	synonymous SNV	OXA1L:NM_005015:exon1:c.G78A:p.L26L	NA	rs1957374	NA
chr14	23518270	23518270	C	A	exonic	CDH24	synonymous SNV	_144985:exon11:c.G1812T:p.L604L,CDH24:NM_022478:exon12:c.G19	NA	NA	NA
chr14	23568766	23568766	-	T	UTR3	C14orf119	NA	NA	NA	NA	NA
chr14	23795205	23795206	TT	-	UTR3	BCL2L2-PABPN1,PABPN1	NA	NA	NA	rs58264930	NA
chr14	29262813	29262813	T	C	ncRNA_exonic	C14orf23	NA	NA	NA	rs71414080	NA
chr14	34272917	34272920	AAAA	-	UTR3	NPAS3	NA	NA	NA	NA	NA
chr14	34394147	34394147	-	AA	UTR3	EGLN3	NA	NA	NA	NA	NA
chr14	36986022	36986022	-	A	UTR3	NKX2-1	NA	NA	NA	rs112799458	NA
chr14	45397887	45397887	T	-	UTR3	KLHL28	NA	NA	NA	NA	NA
chr14	45584495	45584495	T	-	UTR3	PRPF39	NA	NA	NA	NA	NA
chr14	47310821	47310821	C	T	UTR3	MDGA2	NA	NA	NA	rs200571407	NA
chr14	47310822	47310822	T	C	UTR3	MDGA2	NA	NA	NA	NA	NA
chr14	47310830	47310830	A	T	UTR3	MDGA2	NA	NA	NA	NA	NA
chr14	47310830	47310830	-	T	UTR3	MDGA2	NA	NA	NA	rs71448138	NA
chr14	50778816	50778816	A	C	exonic	L2HGDH	nonsynonymous SNV	L2HGDH:NM_024884:exon1:c.T53G:p.L18R	NA	rs2275591	NA
chr14	53619544	53619544	G	A	exonic	DDHD1	synonymous SNV	C273T:p.F91F,DDHD1:NM_001160148:exon1:c.C273T:p.F91F,DDHD1:N	NA	rs2358189	NA
chr14	55494090	55494090	G	A	UTR5	SOCS4	NA	NA	NA	rs77678033	NA
chr14	55494091	55494091	G	A	UTR5	SOCS4	NA	NA	NA	rs77911976	NA
chr14	57114800	57114800	A	-	UTR3	TMEM260	NA	NA	NA	NA	NA
chr14	57280005	57280005	T	-	ncRNA_exonic	OTX2-AS1	NA	NA	NA	NA	NA
chr14	59835968	59835968	C	A	UTR3	DAAM1	NA	NA	NA	rs74057123	NA
chr14	59954397	59954397	C	A	exonic	JKAMP	stopgain SNV	M_001098625:exon3:c.C84A:p.C28X,JKAMP:NM_016475:exon3:c.C10	NA	NA	NA
chr14	60759368	60759368	T	-	UTR3	PPM1A	NA	NA	NA	NA	NA
chr14	65054966	65054966	C	T	exonic	PPP1R36	synonymous SNV	PPP1R36:NM_172365:exon11:c.C1035T:p.A345A	NA	rs116953714	NA
chr14	67852376	67852376	T	-	UTR3	EIF2S1	NA	NA	NA	rs71727390	NA
chr14	68035879	68035879	A	G	exonic	PLEKHH1	nonsynonymous SNV	PLEKHH1:NM_020715:exon8:c.A1288G:p.T430A	NA	rs3825725	NA
chr14	68066949	68066949	G	A	UTR5	PIGH	NA	NA	NA	rs11547225	NA
chr14	73032200	73032200	-	TC	UTR3	RGS6	NA	NA	NA	rs140460180	NA
chr14	73137344	73137345	TC	-	UTR3	DPF3	NA	NA	NA	NA	NA
chr14	74168648	74168648	A	-	UTR3	DNAL1	NA	NA	NA	NA	NA
chr14	74205707	74205707	T	G	exonic	ELMSAN1	synonymous SNV	_001043318:exon2:c.A1005C:p.L335L,ELMSAN1:NM_194278:exon2:c.A	NA	NA	NA
chr14	76668880	76668881	AC	-	UTR3	GPATCH2L	NA	NA	NA	NA	NA
chr14	78045446	78045446	C	A	exonic	SPTLC2	nonsynonymous SNV	SPTLC2:NM_004863:exon3:c.G334T:p.V112L	NA	NA	B
chr14	89627942	89627942	C	T	UTR3	FOXN3	NA	NA	NA	rs7156172	NA
chr14	89627946	89627946	C	T	UTR3	FOXN3	NA	NA	NA	NA	NA
chr14	91084338	91084338	C	T	exonic	TTC7B	synonymous SNV	TTC7B:NM_001010854:exon16:c.G1803A:p.E601E	NA	rs754247	NA
chr14	92527592	92527592	A	-	UTR3	ATXN3	NA	NA	NA	rs201936460	NA
chr14	95655320	95655320	A	-	UTR3	CLMN	NA	NA	NA	NA	NA
chr14	99638737	99638737	T	-	UTR3	BCL11B	NA	NA	NA	rs199890567	NA
chr14	1.03E+08	1.03E+08	A	C	UTR3	RCOR1	NA	NA	NA	NA	NA
chr14	1.03E+08	1.03E+08	A	C	UTR3	RCOR1	NA	NA	NA	NA	NA
chr14	1.03E+08	1.03E+08	T	C	UTR3	RCOR1	NA	NA	NA	NA	NA
chr14	1.05E+08	1.05E+08	A	G	exonic	KIF26A	synonymous SNV	KIF26A:NM_015656:exon12:c.A4284G:p.A1428A	NA	rs2487303	NA
chr14	1.05E+08	1.05E+08	A	G	exonic	AHNAK2	synonymous SNV	AHNAK2:NM_138420:exon7:c.T9783C:p.D3261D	NA	rs28564728	NA
chr14	1.05E+08	1.05E+08	T	G	exonic	AHNAK2	nonsynonymous SNV	AHNAK2:NM_138420:exon7:c.A9563C:p.N3188T	NA	rs201816632	NA
chr14	1.05E+08	1.05E+08	T	C	exonic	AHNAK2	synonymous SNV	AHNAK2:NM_138420:exon7:c.A9513G:p.P3171P	NA	rs2819427	NA
chr14	1.05E+08	1.05E+08	A	G	exonic	AHNAK2	nonsynonymous SNV	AHNAK2:NM_138420:exon7:c.T6326C:p.V2109A	NA	rs201948776	NA
chr14	1.05E+08	1.05E+08	T	C	exonic	AHNAK2	nonsynonymous SNV	AHNAK2:NM_138420:exon7:c.A6145G:p.T2049A	NA	rs201406401	NA
chr14	1.06E+08	1.06E+08	C	T	exonic	BTBD6	synonymous SNV	BTBD6:NM_033271:exon3:c.C237T:p.N79N	NA	rs2816605	NA
chr14	1.06E+08	1.06E+08	C	T	ncRNA_exonic	KIAA0125	NA	NA	NA	rs11850004	NA
chr15	20739885	20739885	T	C	exonic	GOLGA6L6	nonsynonymous SNV	GOLGA6L6:NM_001145004:exon8:c.A1865G:p.Q622R	NA	rs57050697	NA
chr15	21934053	21934053	G	A	ncRNA_exonic	LOC646214	NA	NA	NA	NA	NA
chr15	21938081	21938081	C	T	ncRNA_exonic	LOC646214	NA	NA	NA	rs113837443	NA
chr15	21940266	21940266	-	A	ncRNA_exonic	LOC646214	NA	NA	NA	NA	NA
chr15	21940423	21940423	G	A	ncRNA_exonic	LOC646214	NA	NA	NA	rs12102267	NA
chr15	22074657	22074657	C	G	exonic	100287399,LOC100996331,PC	nonsynonymous SNV	45C:p.E249Q,LOC100996331:NM_001277304:exon4:c.G745C:p.E249Q,	NA	rs200899135	NA
chr15	22138292	22138292	C	T	ncRNA_exonic	NF1P2	NA	NA	NA	rs138146330	NA
chr15	22711791	22711791	A	G	ncRNA_exonic	GOLGA8DP	NA	NA	NA	NA	NA
chr15	22742718	22742718	T	A	exonic	GOLGA6L1	nonsynonymous SNV	GOLGA6L1:NM_001001413:exon8:c.T1103A:p.L368Q	M230296;OCCURENCE=1(NS),2(endom	rs7171381	NA

chr15	23448116	23448116	G	C	ncRNA_exonic	GOLGA8EP	NA	NA	NA	rs1813574	NA
chr15	23448157	23448157	T	C	ncRNA_exonic	GOLGA8EP	NA	NA	NA	rs76705008	NA
chr15	24412403	24412403	A	T	ncRNA_exonic	PWRN2	NA	NA	NA	rs12907382	NA
chr15	25330550	25330550	T	A	ncRNA_exonic	SNORD116-18	NA	NA	NA	rs3803328	NA
chr15	28518112	28518112	C	A	exonic	HERC2	nonsynonymous SNV	HERC2:NM_004667:exon8:c.G839T:p.S280I	NA	rs200859595	B
chr15	28518126	28518126	G	A	exonic	HERC2	synonymous SNV	HERC2:NM_004667:exon8:c.C825T:p.T275T	NA	NA	NA
chr15	28518130	28518130	G	C	exonic	HERC2	nonsynonymous SNV	HERC2:NM_004667:exon8:c.C821G:p.A274G	NA	NA	B
chr15	28518135	28518135	C	T	exonic	HERC2	synonymous SNV	HERC2:NM_004667:exon8:c.G816A:p.T272T	OSM289694;OCCURENCE=1(large_intes	rs141674943	NA
chr15	28518136	28518136	G	A	exonic	HERC2	nonsynonymous SNV	HERC2:NM_004667:exon8:c.C815T:p.T272M	NA	rs201979656	B
chr15	29001133	29001133	C	T	ncRNA_exonic	WHAMMP2	NA	NA	NA	rs142551791	NA
chr15	31664441	31664441	T	C	exonic	KLF13	nonsynonymous SNV	KLF13:NM_015995:exon2:c.T806C:p.L269P	NA	NA	NA
chr15	35812484	35812484	G	A	UTR3	ATPBD4	NA	NA	NA	NA	NA
chr15	40846105	40846140	GTGTGTGT	-	UTR3	C15orf57	NA	NA	NA	NA	NA
chr15	40987565	40987565	G	T	UTR5	RAD51	NA	NA	NA	rs1801321	NA
chr15	41773443	41773443	G	-	UTR3	RTF1	NA	NA	NA	rs56118647	NA
chr15	41865525	41865525	G	T	exonic	TYRO3	nonsynonymous SNV	TYRO3:NM_006293:exon17:c.G2005T:p.V669L	NA	rs62001448	D
chr15	41871513	41871513	T	A	UTR3	TYRO3	NA	NA	NA	NA	NA
chr15	42119323	42119323	-	TC	UTR3	MAPKBP1	NA	NA	NA	rs34551245	NA
chr15	42570718	42570718	A	G	exonic	GANC	nonsynonymous SNV	GANC:NM_198141:exon3:c.A131G:p.Q44R	NA	rs8024732	B
chr15	43650560	43650560	T	-	UTR3	ZSCAN29	NA	NA	NA	NA	NA
chr15	43652835	43652835	A	-	UTR3	ZSCAN29	NA	NA	NA	rs67982601	NA
chr15	48064867	48064867	T	A	UTR3	SEMA6D	NA	NA	NA	rs3803349	NA
chr15	50226359	50226359	G	C	exonic	ATP8B4	nonsynonymous SNV	ATP8B4:NM_024837:exon15:c.C1308G:p.F436L	NA	rs55687265	P
chr15	50643858	50643865	GAGGGAGG	-	ncRNA_exonic	FLJ10038	NA	NA	NA	rs146731069	NA
chr15	50648219	50648219	A	-	ncRNA_exonic	GABPB1-AS1	NA	NA	NA	NA	NA
chr15	52413807	52413807	-	T	UTR3	GNB5	NA	NA	NA	NA	NA
chr15	55904895	55904895	A	T	UTR3	PRTG	NA	NA	NA	NA	NA
chr15	55905957	55905957	-	T	UTR3	PRTG	NA	NA	NA	NA	NA
chr15	57840202	57840202	T	-	UTR3	CGNL1	NA	NA	NA	NA	NA
chr15	60782356	60782356	-	AT	UTR3	RORA	NA	NA	NA	rs35741328	NA
chr15	63362507	63362507	G	A	UTR3	TPM1	NA	NA	NA	rs143569714	NA
chr15	63362509	63362509	G	A	UTR3	TPM1	NA	NA	NA	NA	NA
chr15	64434537	64434537	G	A	UTR3	SNX1	NA	NA	NA	rs17201990	NA
chr15	65739303	65739303	C	A	exonic	DPP8	synonymous SNV	_130434:exon20:c.G2568T:p.S856S,DPP8:NM_197961:exon20:c.G246	NA	rs11009	NA
chr15	68594394	68594423	TGTGTGTG	-	UTR3	ITGA11	NA	NA	NA	NA	NA
chr15	70341342	70341342	A	-	UTR3	TLE3	NA	NA	NA	NA	NA
chr15	70341854	70341854	A	-	UTR3	TLE3	NA	NA	NA	rs11324999	NA
chr15	72452675	72452677	AAA	-	UTR3	GRAMD2	NA	NA	NA	NA	NA
chr15	72642905	72642905	C	T	exonic	HEXA	synonymous SNV	HEXA:NM_000520:exon7:c.G759A:p.V253V	NA	rs117513345	NA
chr15	72953658	72953658	G	A	exonic	GOLGA6B	synonymous SNV	GOLGA6B:NM_018652:exon8:c.G618A:p.K206K	NA	NA	NA
chr15	72958366	72958366	T	G	exonic	GOLGA6B	nonsynonymous SNV	GOLGA6B:NM_018652:exon17:c.T1851G:p.H617Q	NA	rs200655139	NA
chr15	75095093	75095093	A	C	UTR3	CSK	NA	NA	NA	NA	NA
chr15	75564457	75564457	G	C	UTR3	GOLGA6C	NA	NA	NA	rs2740425	NA
chr15	76029087	76029087	T	C	ncRNA_intronic	DNM1P35	NA	NA	NA	rs7496047	NA
chr15	80215597	80215597	G	T	ncRNA_exonic	C15orf37	NA	NA	NA	rs2303824	NA
chr15	82634574	82634574	C	T	UTR3	GOLGA6L10	NA	NA	NA	rs28482898	NA
chr15	82634594	82634594	A	G	UTR3	GOLGA6L10	NA	NA	NA	rs4039143	NA
chr15	82637315	82637315	C	T	exonic	GOLGA6L10	synonymous SNV	GOLGA6L10:NM_001164465:exon6:c.G771A:p.L257L	NA	rs200605893	NA
chr15	82637356	82637356	G	A	exonic	GOLGA6L10	nonsynonymous SNV	GOLGA6L10:NM_001164465:exon6:c.C730T:p.R244C	NA	rs201144848	NA
chr15	82637377	82637377	G	A	exonic	GOLGA6L10	nonsynonymous SNV	GOLGA6L10:NM_001164465:exon6:c.C709T:p.R237C	NA	rs200328108	NA
chr15	82637378	82637378	T	C	exonic	GOLGA6L10	synonymous SNV	GOLGA6L10:NM_001164465:exon6:c.A708G:p.L236L	NA	NA	NA
chr15	82637419	82637419	A	G	exonic	GOLGA6L10	nonsynonymous SNV	GOLGA6L10:NM_001164465:exon6:c.T667C:p.C223R	NA	NA	NA
chr15	82637420	82637420	C	T	exonic	GOLGA6L10	synonymous SNV	GOLGA6L10:NM_001164465:exon6:c.G666A:p.L222L	NA	NA	NA
chr15	82637439	82637439	T	C	exonic	GOLGA6L10	nonsynonymous SNV	GOLGA6L10:NM_001164465:exon6:c.A647G:p.H216R	NA	rs185538129	NA
chr15	82637460	82637460	T	C	exonic	GOLGA6L10	nonsynonymous SNV	GOLGA6L10:NM_001164465:exon6:c.A626G:p.H209R	NA	rs199925595	NA
chr15	83013665	83013665	C	T	intergenic	97(dist=27238),UBE2Q2P3(dis	NA	NA	NA	NA	NA
chr15	83013916	83013916	A	G	intergenic	97(dist=27489),UBE2Q2P3(di	NA	NA	NA	NA	NA
chr15	83013917	83013917	C	T	intergenic	97(dist=27490),UBE2Q2P3(di	NA	NA	NA	NA	NA
chr15	83502439	83502439	T	-	UTR3	WHAMM	NA	NA	NA	NA	NA
chr15	83680329	83680329	G	A	exonic	C15orf40	nonsynonymous SNV	.C31T:p.L11F,C15orf40:NM_001160115:exon1:c.C31T:p.L11F,C15orf40	NA	rs17361375	NA
chr15	85055093	85055093	A	C	ncRNA_exonic	GOLGA6L5	NA	NA	NA	rs227573	NA
chr15	85360004	85360004	A	C	UTR5	ALPK3	NA	NA	NA	rs55736017	NA
chr15	89386652	89386652	G	A	exonic	ACAN	nonsynonymous SNV	M_001135:exon6:c.G824A:p.R275Q,ACAN:NM_013227:exon6:c.G824A	NA	rs34949187	NA
chr15	89398883	89398883	A	T	exonic	ACAN	nonsynonymous SNV	001135:exon12:c.A3067T:p.T1023S,ACAN:NM_013227:exon12:c.A306	NA	NA	NA
chr15	90170933	90170933	G	A	UTR3	TICRR	NA	NA	NA	rs3743371	NA
chr15	90349667	90349667	T	G	exonic	ANPEP	nonsynonymous SNV	ANPEP:NM_001150:exon2:c.A148C:p.T50P	NA	NA	B

chr15	91425783	91425783	T	C	UTR3	FURIN	NA	NA	NA	NA	NA
chr15	92690366	92690366	A	C	exonic	SLCO3A1	synonymous SNV	001145044:exon8:c.A1665C:p.T555T,SLCO3A1:NM_013272:exon8:c.A	NA	NA	NA
chr15	98980925	98980925	G	-	UTR3	FAM169B	NA	NA	NA	NA	NA
chr15	98981194	98981196	GAT	-	UTR3	FAM169B	NA	NA	NA	NA	NA
chr15	98981290	98981290	T	-	UTR3	FAM169B	NA	NA	NA	NA	NA
chr15	1E+08	1E+08	G	C	UTR3	MEF2A	NA	NA	NA	rs325382	NA
chr15	1E+08	1E+08	C	T	ncRNA_exonic	DNM1P46	NA	NA	NA	NA	NA
chr15	1E+08	1E+08	G	T	ncRNA_exonic	DNM1P46	NA	NA	NA	NA	NA
chr15	1E+08	1E+08	T	C	ncRNA_exonic	DNM1P46	NA	NA	NA	NA	NA
chr16	449109	449109	A	G	exonic	NME4	nonsynonymous SNV	NME4:NM_005009:exon2:c.A211G:p.M71V	NA	NA	D
chr16	602313	602313	C	A	exonic	SOLH	synonymous SNV	SOLH:NM_005632:exon11:c.C2520A:p.A840A	NA	rs9930550	NA
chr16	822742	822742	T	G	intergenic	62(dist=2465),RPUSD1(dist=1	NA	NA	NA	NA	NA
chr16	848213	848213	T	G	UTR3	GNG13	NA	NA	NA	NA	NA
chr16	1036733	1036733	C	T	UTR3	SOX8	NA	NA	NA	NA	NA
chr16	1146228	1146228	C	T	UTR5	C1QTNF8	NA	NA	NA	rs2076422	NA
chr16	1252259	1252259	A	G	exonic	CACNA1H	synonymous SNV	001005407:exon9:c.A1809G:p.R603R,CACNA1H:NM_021098:exon9:c.A	NA	rs9934839	NA
chr16	1258193	1258193	A	C	exonic	CACNA1H	nonsynonymous SNV	1005407:exon16:c.A3335C:p.D1112A,CACNA1H:NM_021098:exon16:c	NA	rs202114960	NA
chr16	1291308	1291308	A	G	exonic	TPSAB1	synonymous SNV	TPSAB1:NM_003294:exon3:c.A216G:p.A72A	NA	rs201568970	NA
chr16	1291608	1291608	A	G	exonic	TPSAB1	nonsynonymous SNV	TPSAB1:NM_003294:exon4:c.A407G:p.H136R	NA	rs1064780	B
chr16	1291623	1291623	C	T	exonic	TPSAB1	nonsynonymous SNV	TPSAB1:NM_003294:exon4:c.C422T:p.T141I	NA	rs1064781	P
chr16	1291639	1291639	A	G	exonic	TPSAB1	synonymous SNV	TPSAB1:NM_003294:exon4:c.A438G:p.S146S	NA	rs1064783	NA
chr16	1291941	1291941	G	A	exonic	TPSAB1	nonsynonymous SNV	TPSAB1:NM_003294:exon5:c.G613A:p.V205I	NA	rs1060284	B
chr16	1306523	1306523	G	T	exonic	TPSD1	nonsynonymous SNV	TPSD1:NM_012217:exon2:c.G89T:p.G30V	NA	rs2401937	D
chr16	1414996	1414996	T	-	UTR3	UNKL	NA	NA	NA	rs112378427	NA
chr16	1484392	1484392	G	C	UTR3	CCDC154	NA	NA	NA	rs12598718	NA
chr16	1545448	1545448	A	G	exonic	TELO2	nonsynonymous SNV	TELO2:NM_016111:exon3:c.A437G:p.Q146R	NA	rs2235624	B
chr16	1591962	1591962	G	A	exonic	TMEM204	synonymous SNV	_024600:exon2:c.G321A:p.T107T,TMEM204:NM_001256541:exon3:c.(	NA	rs2076443	NA
chr16	1876949	1876949	G	C	UTR5	HAGH	NA	NA	NA	rs7188401	NA
chr16	1878295	1878295	G	A	UTR3	FAHD1	NA	NA	NA	NA	NA
chr16	2052320	2052320	T	G	exonic	ZNF598	nonsynonymous SNV	ZNF598:NM_178167:exon7:c.A782C:p.D261A	NA	NA	NA
chr16	2052345	2052345	T	G	exonic	ZNF598	nonsynonymous SNV	ZNF598:NM_178167:exon7:c.A757C:p.T253P	NA	NA	NA
chr16	2121850	2121850	-	C	exonic	TSC2	frameshift insertion	71fs,TSC2:NM_001077183:exon19:c.2012_2013insC:p.G671fs,TSC2:NI	NA	NA	NA
chr16	2653536	2653536	T	G	ncRNA_exonic	LOC652276	NA	NA	NA	NA	NA
chr16	3077670	3077670	A	C	UTR3	THOC6	NA	NA	NA	NA	NA
chr16	3079685	3079685	T	C	exonic	CCDC64B	nonsynonymous SNV	CCDC64B:NM_001103175:exon5:c.A818G:p.Q273R	NA	rs2244494	NA
chr16	3292666	3292666	T	C	UTR3	MEFV	NA	NA	NA	rs11466052	NA
chr16	3292673	3292675	AAA	-	UTR3	MEFV	NA	NA	NA	rs61379197	NA
chr16	3304762	3304762	A	G	exonic	MEFV	synonymous SNV	MEFV:NM_000243:exon2:c.T306C:p.D102D	NA	rs224225	NA
chr16	7761230	7761230	A	-	UTR3	RBFOX1	NA	NA	NA	rs59509314	NA
chr16	8986464	8986468	AAAAA	-	UTR3	USP7	NA	NA	NA	rs141993088	NA
chr16	10624908	10624908	G	A	UTR3	EMP2	NA	NA	NA	rs7187959	NA
chr16	11962217	11962217	T	-	UTR3	GSPT1	NA	NA	NA	NA	NA
chr16	15818992	15818992	A	-	UTR3	NDE1	NA	NA	NA	NA	NA
chr16	19131760	19131760	T	-	UTR3	ITPRIPL2	NA	NA	NA	rs112434410	NA
chr16	19513697	19513702	TTTTTT	-	UTR3	GDE1	NA	NA	NA	NA	NA
chr16	19563849	19563851	AAA	-	UTR3	CCP110	NA	NA	NA	NA	NA
chr16	20870169	20870169	A	T	UTR3	DCUN1D3	NA	NA	NA	NA	NA
chr16	21191514	21191514	G	T	UTR3	TMEM159	NA	NA	NA	NA	NA
chr16	22546026	22546026	A	C	exonic	LOC100132247	synonymous SNV	LOC100132247:NM_001135865:exon7:c.A1722C:p.T574T	NA	NA	NA
chr16	22546469	22546469	A	C	exonic	LOC100132247	nonsynonymous SNV	LOC100132247:NM_001135865:exon7:c.A2165C:p.H722P	NA	NA	NA
chr16	23076644	23076644	A	-	UTR3	USP31	NA	NA	NA	NA	NA
chr16	24835459	24835459	G	C	UTR3	TNRC6A	NA	NA	NA	rs112183094	NA
chr16	25250585	25250586	AA	-	UTR3	ZKSCAN2	NA	NA	NA	rs58897018	NA
chr16	29381579	29381579	A	-	intergenic	2(dist=5199),LOC606724(dist	NA	NA	NA	NA	NA
chr16	29821984	29821984	T	-	UTR3	MAZ	NA	NA	NA	rs71650270	NA
chr16	29910394	29910394	A	C	UTR5	SEZ6L2	NA	NA	NA	NA	NA
chr16	31003411	31003411	-	AC	UTR3	STX1B	NA	NA	NA	rs112607901	NA
chr16	34404050	34404050	T	G	ncRNA_exonic	UBE2MP1	NA	NA	NA	NA	NA
chr16	48226479	48226479	C	T	exonic	ABCC11	synonymous SNV	A:p.K886K,ABCC11:NM_032583:exon20:c.G2658A:p.K886K,ABCC11:N	NA	rs12443685	NA
chr16	50832526	50832526	A	-	UTR3	CYLD	NA	NA	NA	rs111804189	NA
chr16	54320197	54320197	G	T	UTR5	IRX3	NA	NA	NA	rs3751723	NA
chr16	54967096	54967096	C	A	exonic	IRX5	nonsynonymous SNV	_001252197:exon3:c.C760A:p.P254T,IRX5:NM_005853:exon3:c.C763A	NA	rs13336114	B
chr16	55798671	55798671	G	A	ncRNA_exonic	CES1P1	NA	NA	NA	rs34649320	NA
chr16	55798733	55798733	G	C	ncRNA_exonic	CES1P1	NA	NA	NA	rs35384853	NA
chr16	55798759	55798759	T	C	ncRNA_exonic	CES1P1	NA	NA	NA	rs71374087	NA
chr16	55798763	55798763	A	G	ncRNA_exonic	CES1P1	NA	NA	NA	rs71374088	NA

chr16	55798778	55798778	A	G	ncRNA_exonic	CES1P1	NA	NA	NA	rs76167618	NA
chr16	55798783	55798783	A	G	ncRNA_exonic	CES1P1	NA	NA	NA	rs75712934	NA
chr16	56691861	56691861	T	C	UTR5	MT1F	NA	NA	NA	NA	NA
chr16	57091977	57091977	T	C	exonic	NLRC5	synonymous SNV	NLRC5:NM_032206:exon27:c.T3747C:p.D1249D	NA	rs7206703	NA
chr16	57398706	57398706	-	T	UTR3	CCL22	NA	NA	NA	rs57450696	NA
chr16	66638219	66638219	A	T	upstream	CMTM3	NA	NA	NA	rs146585124	NA
chr16	67201665	67201665	A	G	exonic	HSF4	synonymous SNV	HSF4:NM_001040667:exon11:c.A897G:p.P299P	NA	NA	NA
chr16	67841129	67841129	T	G	UTR5	TSNAXIP1	NA	NA	NA	rs7199443	NA
chr16	67905065	67905065	C	T	UTR3	NUTF2	NA	NA	NA	NA	NA
chr16	68678505	68678505	G	T	UTR5	CDH3	NA	NA	NA	NA	NA
chr16	69151479	69151479	-	T	UTR3	HAS3	NA	NA	NA	NA	NA
chr16	69339663	69339663	T	-	UTR3	SNTB2	NA	NA	NA	NA	NA
chr16	70010369	70010369	C	G	ncRNA_exonic	PDXDC2P	NA	NA	NA	rs187431035	NA
chr16	70010370	70010370	A	G	ncRNA_exonic	PDXDC2P	NA	NA	NA	NA	NA
chr16	70010374	70010374	A	G	ncRNA_exonic	PDXDC2P	NA	NA	NA	NA	NA
chr16	70010380	70010380	C	T	ncRNA_exonic	PDXDC2P	NA	NA	NA	NA	NA
chr16	70011129	70011129	G	A	ncRNA_exonic	PDXDC2P	NA	NA	NA	NA	NA
chr16	70011173	70011173	A	G	ncRNA_exonic	PDXDC2P	NA	NA	NA	NA	NA
chr16	71025245	71025245	C	T	exonic	HYDIN	synonymous SNV	HYDIN:NM_001270974:exon25:c.G3840A:p.T1280T	NA	rs116894611	NA
chr16	72058667	72058667	G	A	UTR3	DHODH	NA	NA	NA	rs113365769	NA
chr16	72820483	72820483	C	A	UTR3	ZFHX3	NA	NA	NA	rs200323038	NA
chr16	74486093	74486093	A	C	UTR3	GLG1	NA	NA	NA	NA	NA
chr16	74655601	74655601	G	T	UTR3	RFWD3	NA	NA	NA	rs71386224	NA
chr16	81744503	81744503	A	C	UTR3	CMIP	NA	NA	NA	NA	NA
chr16	85125545	85125549	TTTTT	-	UTR3	KIAA0513	NA	NA	NA	NA	NA
chr16	85695241	85695241	G	A	exonic	GSE1	synonymous SNV	001134473:exon8:c.G1818A:p.S606S,GSE1:NM_014615:exon9:c.G213	NA	NA	NA
chr16	85695242	85695242	C	G	exonic	GSE1	nonsynonymous SNV	001134473:exon8:c.C1819G:p.P607A,GSE1:NM_014615:exon9:c.C213	NA	NA	D
chr16	85695243	85695243	C	G	exonic	GSE1	nonsynonymous SNV	001134473:exon8:c.C1820G:p.P607R,GSE1:NM_014615:exon9:c.C213	NA	NA	D
chr16	85695244	85695244	C	A	exonic	GSE1	synonymous SNV	001134473:exon8:c.C1821A:p.P607P,GSE1:NM_014615:exon9:c.C213	NA	NA	NA
chr16	88506287	88506287	-	CTCTGACCA	UTR3	ZNF469	NA	NA	NA	rs3838248	NA
chr16	88552370	88552370	A	G	exonic	ZFPM1	nonsynonymous SNV	ZFPM1:NM_153813:exon2:c.A64G:p.R22G	NA	rs3751673	NA
chr16	88599696	88599697	GA	-	exonic	ZFPM1	frameshift deletion	ZFPM1:NM_153813:exon10:c.1330_1331del:p.444_444del	NA	rs67712719	NA
chr16	88599701	88599701	T	-	exonic	ZFPM1	frameshift deletion	ZFPM1:NM_153813:exon10:c.1335delT:p.P445fs	NA	rs67322929	NA
chr16	88599703	88599705	TGG	-	exonic	ZFPM1	nonframeshift deletion	ZFPM1:NM_153813:exon10:c.1337_1339del:p.446_447del	NA	rs67873604	NA
chr16	89346906	89346906	T	G	exonic	ANKRD11	nonsynonymous SNV	p.Y2015S,ANKRD11:NM_013275:exon9:c.A6044C:p.Y2015S,ANKRD11:	NA	NA	D
chr16	90061507	90061507	T	A	ncRNA_exonic	AFG3L1P	NA	NA	NA	NA	NA
chr16	90061738	90061738	T	C	ncRNA_exonic	AFG3L1P	NA	NA	NA	NA	NA
chr16	90062158	90062158	A	G	ncRNA_exonic	AFG3L1P	NA	NA	NA	NA	NA
chr17	263576	263576	T	C	exonic	C17orf97	synonymous SNV	C17orf97:NM_001013672:exon2:c.T942C:p.G314G	NA	rs111445631	NA
chr17	292322	292322	A	-	UTR3	FAM101B	NA	NA	NA	rs139875162	NA
chr17	416962	416962	T	C	UTR3	VPS53	NA	NA	NA	rs8080942	NA
chr17	417718	417718	T	C	UTR3	VPS53	NA	NA	NA	NA	NA
chr17	418519	418519	-	G	UTR3	VPS53	NA	NA	NA	rs146415154	NA
chr17	1090273	1090273	G	C	UTR5	ABR	NA	NA	NA	rs3813440	NA
chr17	1683882	1683882	C	A	UTR3	SMYD4	NA	NA	NA	NA	NA
chr17	1839009	1839009	A	G	UTR3	RTN4RL1	NA	NA	NA	rs188773038	NA
chr17	1839011	1839011	A	G	UTR3	RTN4RL1	NA	NA	NA	rs201978444	NA
chr17	1964759	1964759	-	G	UTR3	SMG6	NA	NA	NA	rs145926620	NA
chr17	2595964	2595964	G	A	exonic	CLUH	synonymous SNV	CLUH:NM_015229:exon21:c.C3222T:p.A1074A	NA	rs2302199	NA
chr17	2938272	2938272	T	-	UTR3	RAP1GAP2	NA	NA	NA	rs71830536	NA
chr17	3343519	3343519	T	C	exonic	SPATA22	synonymous SNV	G:p.E338E,SPATA22:NM_001170697:exon9:c.A1014G:p.E338E,SPATA2	NA	rs17822627	NA
chr17	3447914	3447914	C	T	exonic	TRPV3	synonymous SNV	_001258205:exon4:c.G270A:p.Q90Q,TRPV3:NM_145068:exon4:c.G27	NA	rs1039519	NA
chr17	3539835	3539835	T	C	UTR5	CTNS	NA	NA	NA	rs111977802	NA
chr17	4461935	4461935	A	C	exonic	GGT6	nonsynonymous SNV	_153338:exon3:c.T761G:p.V254G,GGT6:NM_001122890:exon4:c.T857	NA	NA	B
chr17	6327551	6327551	G	A	UTR3	AIPL1	NA	NA	NA	rs907938	NA
chr17	6328628	6328629	TT	-	UTR3	AIPL1	NA	NA	NA	rs56845881	NA
chr17	6556538	6556538	G	C	UTR3	C17orf100	NA	NA	NA	rs4796533	NA
chr17	6556541	6556541	A	T	UTR3	C17orf100	NA	NA	NA	rs4796343	NA
chr17	7097657	7097657	A	C	exonic	DLG4	nonsynonymous SNV	01128827:exon12:c.T1450G:p.F484V,DLG4:NM_001365:exon14:c.T15	NA	NA	NA
chr17	7190439	7190439	T	C	UTR3	SLC2A4	NA	NA	NA	NA	NA
chr17	7190440	7190440	T	C	UTR3	SLC2A4	NA	NA	NA	rs55782239	NA
chr17	7554536	7554536	T	C	UTR5	ATP1B2	NA	NA	NA	rs1050528	NA
chr17	8294348	8294348	T	-	UTR3	RNF222	NA	NA	NA	NA	NA
chr17	8731943	8731943	T	C	exonic	PIK3R6	unknown	UNKNOWN	NA	rs140947985	NA
chr17	10633539	10633542	AAAA	-	ncRNA_UTR5	TMEM220	NA	NA	NA	rs59392100	NA
chr17	11513845	11513845	G	T	exonic	DNAH9	nonsynonymous SNV	DNAH9:NM_001372:exon3:c.G747T:p.K249N	NA	NA	B

chr17	13928567	13928567	C	G	ncRNA_exonic	CDRT15P1	NA	NA	NA	rs148540138	NA
chr17	15621245	15621245	-	T	UTR3	ZNF286A	NA	NA	NA	NA	NA
chr17	16285719	16285719	T	C	exonic	UBB	synonymous SNV	UBB:NM_018955:exon2:c.T498C:p.T166T	NA	rs1060763	NA
chr17	16318932	16318932	T	C	UTR5	TRPV2	NA	NA	NA	rs3813769	NA
chr17	16344891	16344891	T	-	ncRNA_exonic	C17orf76-AS1	NA	NA	NA	rs71831095	NA
chr17	17680560	17680560	T	G	ncRNA_exonic	SMCR5	NA	NA	NA	rs941445	NA
chr17	17714851	17714851	G	-	UTR3	SREBF1	NA	NA	NA	rs11304210	NA
chr17	17942613	17942613	C	T	UTR5	GID4	NA	NA	NA	rs7406982	NA
chr17	17997209	17997209	G	A	exonic	DRG2	synonymous SNV	DRG2:NM_001388:exon2:c.G147A:p.S49S	NA	rs2230316	NA
chr17	18291544	18291544	A	G	exonic	EVPLL	synonymous SNV	EVPLL:NM_001145127:exon10:c.A888G:p.P296P	NA	rs586322	NA
chr17	20354836	20354836	A	G	exonic	LGALS9B	synonymous SNV	LGALS9B:NM_001042685:exon10:c.T879C:p.S293S	NA	rs150485558	NA
chr17	20407483	20407483	T	C	ncRNA_exonic	KRT16P3	NA	NA	NA	NA	NA
chr17	20767808	20767808	C	G	UTR3	CCDC144NL	NA	NA	NA	rs141460191	NA
chr17	21207813	21207813	T	G	exonic	MAP2K3	nonsynonymous SNV	M_002756:exon8:c.T557G:p.L186W,MAP2K3:NM_145109:exon8:c.T64	NA	rs74575904	D
chr17	21207835	21207835	G	A	exonic	MAP2K3	synonymous SNV	M_002756:exon8:c.G579A:p.T193T,MAP2K3:NM_145109:exon8:c.G66	=COSM1179253;OCCURENCE=1(prostat	rs76111309	NA
chr17	21207844	21207844	C	T	exonic	MAP2K3	synonymous SNV	M_002756:exon8:c.C588T:p.A196A,MAP2K3:NM_145109:exon8:c.C67	NA	rs2230436	NA
chr17	21320787	21320787	G	A	UTR3	KCNJ12	NA	NA	NA	rs76105604	NA
chr17	21320854	21320854	A	-	UTR3	KCNJ12	NA	NA	NA	rs67230821	NA
chr17	21322880	21322880	A	C	UTR3	KCNJ12	NA	NA	NA	rs79110818	NA
chr17	21322883	21322883	G	A	UTR3	KCNJ12	NA	NA	NA	rs78171447	NA
chr17	21911267	21911268	TG	-	ncRNA_exonic	FLJ36000	NA	NA	NA	NA	NA
chr17	27581500	27581500	-	A	UTR3	CRYBA1	NA	NA	NA	NA	NA
chr17	31618865	31618865	A	C	exonic	ASIC2	nonsynonymous SNV	ASIC2:NM_183377:exon1:c.T269G:p.V90G	NA	NA	NA
chr17	32903526	32903526	G	A	UTR3	C17orf102	NA	NA	NA	rs9891306	NA
chr17	33765127	33765127	A	-	UTR3	SLFN13	NA	NA	NA	rs11337992	NA
chr17	34493108	34493108	T	G	UTR3	TBC1D3B	NA	NA	NA	NA	NA
chr17	34493194	34493194	T	C	UTR3	TBC1D3B	NA	NA	NA	rs79500369	NA
chr17	36883387	36883387	T	-	UTR3	MLLT6	NA	NA	NA	NA	NA
chr17	37221649	37221650	TT	-	ncRNA_UTR3	PLXDC1	NA	NA	NA	rs149203975	NA
chr17	37562813	37562813	T	-	UTR3	MED1	NA	NA	NA	NA	NA
chr17	37688356	37688361	TCTCTC	-	UTR3	CDK12	NA	NA	NA	rs71843922	NA
chr17	38122680	38122680	G	T	exonic	GSDMA	nonsynonymous SNV	GSDMA:NM_178171:exon3:c.G382T:p.V128L	NA	rs7212938	NA
chr17	38122686	38122686	G	A	exonic	GSDMA	nonsynonymous SNV	GSDMA:NM_178171:exon3:c.G388A:p.E130K	NA	rs7212944	NA
chr17	39139370	39139370	G	A	exonic	KRT40	nonsynonymous SNV	KRT40:NM_182497:exon4:c.C458T:p.T153M	NA	rs9908304	D
chr17	39240791	39240791	C	T	exonic	KRTAP4-7	synonymous SNV	KRTAP4-7:NM_033061:exon1:c.C333T:p.R111R	NA	NA	NA
chr17	39254143	39254143	C	T	exonic	KRTAP4-8	nonsynonymous SNV	KRTAP4-8:NM_031960:exon1:c.G194A:p.C65Y	NA	NA	NA
chr17	39316570	39316570	T	C	exonic	KRTAP4-4	nonsynonymous SNV	KRTAP4-4:NM_032524:exon1:c.A374G:p.Y125C	NA	rs111563615	NA
chr17	39316746	39316746	A	G	exonic	KRTAP4-4	synonymous SNV	KRTAP4-4:NM_032524:exon1:c.T198C:p.C66C	NA	NA	NA
chr17	39340786	39340786	A	G	exonic	KRTAP4-1	synonymous SNV	KRTAP4-1:NM_033060:exon2:c.T264C:p.C88C	NA	rs2320228	NA
chr17	39675023	39675023	T	C	exonic	KRT15	synonymous SNV	KRT15:NM_002275:exon1:c.A57G:p.R19R	NA	NA	NA
chr17	40465420	40465420	G	-	UTR3	STAT3	NA	NA	NA	NA	NA
chr17	41031196	41031196	A	-	ncRNA_exonic	LINC00671	NA	NA	NA	NA	NA
chr17	41144609	41144609	G	A	UTR3	RUNDC1	NA	NA	NA	rs201072724	NA
chr17	41181922	41181923	AA	-	UTR3	RND2	NA	NA	NA	NA	NA
chr17	42113045	42113045	-	A	UTR3	LSM12	NA	NA	NA	NA	NA
chr17	42248239	42248239	C	A	exonic	ASB16	synonymous SNV	ASB16:NM_080863:exon1:c.C82A:p.R28R	NA	rs144229400	NA
chr17	42266708	42266708	A	G	exonic	TMUB2	synonymous SNV	G:p.G98G,TMUB2:NM_177441:exon2:c.A294G:p.G98G,TMUB2:NM_0	NA	NA	NA
chr17	42284469	42284469	T	-	UTR3	UBTF	NA	NA	NA	NA	NA
chr17	43101699	43101708	TGTGTGTG	-	UTR3	DCAKD	NA	NA	NA	NA	NA
chr17	44374362	44374362	T	C	exonic	LRRC37A	synonymous SNV	LRRC37A:NM_014834:exon1:c.T1863C:p.P621P	NA	rs17854018	NA
chr17	45055759	45055759	-	AA	UTR3	RPRML	NA	NA	NA	NA	NA
chr17	45214564	45214564	A	T	exonic	CDC27	nonsynonymous SNV	01114091:exon14:c.T1885A:p.C629S,CDC27:NM_001256:exon14:c.T1	1179254,COSM1179255;OCCURENCE=1	rs201184967	NA
chr17	45214606	45214606	G	T	exonic	CDC27	nonsynonymous SNV	01114091:exon14:c.C1843A:p.H615N,CDC27:NM_001256:exon14:c.C1	NA	rs79260965	NA
chr17	45214636	45214636	T	C	exonic	CDC27	nonsynonymous SNV	01114091:exon14:c.A1813G:p.N605D,CDC27:NM_001256:exon14:c.A1	NA	rs200200993	NA
chr17	45234430	45234430	A	G	exonic	CDC27	nonsynonymous SNV	_001114091:exon7:c.T691C:p.S231P,CDC27:NM_001256:exon7:c.T69	NA	rs78072949	B
chr17	45234707	45234707	T	A	exonic	CDC27	nonsynonymous SNV	_001114091:exon6:c.A519T:p.L173F,CDC27:NM_001256:exon6:c.A51	NA	rs75353677	B
chr17	45502222	45502222	C	T	intronic	EFCAB13	NA	NA	NA	NA	NA
chr17	47373330	47373330	G	A	UTR3	ZNF652	NA	NA	NA	NA	NA
chr17	47373333	47373333	C	A	UTR3	ZNF652	NA	NA	NA	NA	NA
chr17	47676880	47676880	G	-	UTR3	SPOP	NA	NA	NA	rs11316512	NA
chr17	48542139	48542139	-	T	UTR3	CHAD	NA	NA	NA	rs60053197	NA
chr17	48701735	48701735	G	T	exonic	CACNA1G	nonsynonymous SNV	2T:p.V1978F,CACNA1G:NM_198379:exon34:c.G6007T:p.V2003F,CACN	NA	NA	NA
chr17	48771502	48771502	G	A	UTR3	ANKRD40	NA	NA	NA	rs11079923	NA
chr17	48771507	48771507	G	A	UTR3	ANKRD40	NA	NA	NA	rs199897066	NA
chr17	49041948	49041948	-	A	UTR3	SPAG9	NA	NA	NA	NA	NA
chr17	53402169	53402169	-	GTGTGC	UTR3	HLF	NA	NA	NA	NA	NA

chr17	54450038	54450038	C	A	exonic	ANKFN1	synonymous SNV	ANKFN1:NM_153228:exon6:c.C642A:p.L214L	NA	rs957724	NA
chr17	54965932	54965932	A	G	UTR3	TRIM25	NA	NA	NA	rs7615	NA
chr17	55197932	55197932	A	-	UTR3	AKAP1	NA	NA	NA	NA	NA
chr17	56438301	56438301	G	A	exonic	RNF43	nonsynonymous SNV	RNF43:NM_017763:exon7:c.C692T:p.P231L	NA	rs2680701	P
chr17	57773034	57773037	AAAA	-	UTR3	CLTC	NA	NA	NA	rs139136427	NA
chr17	58524282	58524282	A	-	UTR3	APPBP2	NA	NA	NA	NA	NA
chr17	60342552	60342552	T	C	ncRNA_exonic	TBC1D3P2	NA	NA	NA	rs200812362	NA
chr17	60342600	60342600	T	G	ncRNA_exonic	TBC1D3P2	NA	NA	NA	rs62069087	NA
chr17	62122724	62122724	T	G	exonic	ERN1	nonsynonymous SNV	ERN1:NM_001433:exon20:c.A2648C:p.Q883P	NA	NA	NA
chr17	62400086	62400086	-	C	UTR3	PECAM1	NA	NA	NA	rs35975413	NA
chr17	62541835	62541835	A	-	UTR3	SMURF2	NA	NA	NA	NA	NA
chr17	62541865	62541865	A	-	UTR3	SMURF2	NA	NA	NA	NA	NA
chr17	66122028	66122028	A	-	ncRNA_exonic	LINC00674	NA	NA	NA	NA	NA
chr17	71332317	71332317	A	-	UTR3	SDK2	NA	NA	NA	NA	NA
chr17	72700943	72700943	A	G	exonic	CD300LF	nonsynonymous SNV	CD300LF:NM_139018:exon2:c.T56C:p.V19A	NA	rs35489971	B
chr17	73060246	73060246	A	C	UTR3	KCTD2	NA	NA	NA	NA	NA
chr17	73498845	73498845	T	G	exonic	CASKIN2	synonymous SNV	01142643:exon17:c.A2064C:p.P688P,CASKIN2:NM_020753:exon18:c.A	NA	NA	NA
chr17	73606310	73606310	G	A	ncRNA_exonic	MYO15B	NA	NA	NA	rs11657863	NA
chr17	73812867	73812867	C	T	exonic	UNK	synonymous SNV	UNK:NM_001080419:exon8:c.C978T:p.D326D	NA	rs112695630	NA
chr17	74288463	74288463	G	A	exonic	QRICH2	nonsynonymous SNV	QRICH2:NM_032134:exon4:c.C1847T:p.A616V	COSM984645;OCCURENCE=1(endometr	NA	B
chr17	75090386	75090386	T	C	ncRNA_exonic	LINC00338	NA	NA	NA	NA	NA
chr17	75090396	75090396	G	A	ncRNA_exonic	LINC00338	NA	NA	NA	NA	NA
chr17	75212385	75212385	C	T	UTR3	SEC14L1	NA	NA	NA	rs112436719	NA
chr17	76083017	76083017	A	C	exonic	TNRC6C	synonymous SNV	1142640:exon14:c.A3636C:p.P1212P,TNRC6C:NM_018996:exon14:c.A	NA	NA	NA
chr17	76102335	76102335	A	-	UTR3	TNRC6C	NA	NA	NA	rs58424290	NA
chr17	76421443	76421443	G	A	exonic	DNAH17	synonymous SNV	DNAH17:NM_173628:exon80:c.C13125T:p.Y4375Y	NA	rs1134541	NA
chr17	76798445	76798445	C	A	exonic	USP36	nonsynonymous SNV	USP36:NM_025090:exon17:c.G2983T:p.A995S	NA	rs145562419	B
chr17	77709339	77709339	C	G	exonic	ENPP7	synonymous SNV	ENPP7:NM_178543:exon3:c.C897G:p.A299A	NA	rs11657217	NA
chr17	77756156	77756156	A	C	UTR3	CBX2	NA	NA	NA	NA	NA
chr17	77807715	77807715	G	T	UTR3	CBX4	NA	NA	NA	NA	NA
chr17	78326266	78326266	T	G	ncRNA_exonic	LOC100294362	NA	NA	NA	NA	NA
chr17	78326270	78326270	A	G	ncRNA_exonic	LOC100294362	NA	NA	NA	NA	NA
chr17	78440927	78440927	T	-	UTR3	NPTX1	NA	NA	NA	NA	NA
chr17	79090262	79090262	-	GCCCCA	UTR3	BAIAP2	NA	NA	NA	rs148172788	NA
chr17	79220020	79220020	A	C	exonic	SLC38A10	nonsynonymous SNV	SLC38A10:NM_001037984:exon16:c.T2696G:p.V899G	NA	NA	P
chr17	79419025	79419025	G	A	exonic	BAHCC1	unknown	UNKNOWN	NA	rs35572189	NA
chr17	79517455	79517455	A	C	exonic	C17orf70	synonymous SNV	C17orf70:NM_025161:exon3:c.T1065G:p.G355G	ID=COSM309626;OCCURENCE=1(lung)	rs202147413	NA
chr17	79615572	79615572	A	G	UTR3	TSPAN10	NA	NA	NA	rs7405453	NA
chr17	80332251	80332251	T	C	exonic	UTS2R	synonymous SNV	UTS2R:NM_018949:exon1:c.T51C:p.T17T	NA	rs11654140	NA
chr17	80561596	80561598	TTT	-	UTR3	FOXK2	NA	NA	NA	NA	NA
chr17	81176848	81176848	G	A	ncRNA_exonic	FLJ43681	NA	NA	NA	rs71271552	NA
chr17	81177608	81177608	C	T	ncRNA_exonic	FLJ43681	NA	NA	NA	rs17143775	NA
chr18	319513	319513	A	-	UTR3	COLEC12	NA	NA	NA	rs11336256	NA
chr18	911381	911381	A	T	UTR3	ADCYAP1	NA	NA	NA	rs68021456	NA
chr18	8387195	8387195	G	A	exonic	PTPRM	synonymous SNV	2845:exon29:c.G4131A:p.E1377E,PTPRM:NM_001105244:exon31:c.G4	NA	rs593978	NA
chr18	8639145	8639145	C	T	UTR3	RAB12	NA	NA	NA	rs200644771	NA
chr18	9285128	9285128	G	A	UTR3	ANKRD12	NA	NA	NA	rs149646961	NA
chr18	9861101	9861101	A	-	UTR3	RAB31	NA	NA	NA	NA	NA
chr18	9887252	9887252	C	A	exonic	TXNDC2	nonsynonymous SNV	_001098529:exon2:c.C776A:p.A259E,TXNDC2:NM_032243:exon2:c.C5	NA	NA	NA
chr18	9887256	9887256	G	A	exonic	TXNDC2	synonymous SNV	_001098529:exon2:c.G780A:p.K260K,TXNDC2:NM_032243:exon2:c.G5	NA	NA	NA
chr18	9887388	9887388	G	A	exonic	TXNDC2	synonymous SNV	_001098529:exon2:c.G912A:p.E304E,TXNDC2:NM_032243:exon2:c.G7	NA	rs1784553	NA
chr18	12991393	12991393	T	C	UTR5	CEP192	NA	NA	NA	rs11663049	NA
chr18	13612545	13612545	C	-	UTR5	LDLRAD4	NA	NA	NA	rs11311417	NA
chr18	13665381	13665384	AAAA	-	UTR3	FAM210A	NA	NA	NA	rs56656145	NA
chr18	18530330	18530330	T	G	UTR3	ROCK1	NA	NA	NA	rs200562208	NA
chr18	18534948	18534948	G	C	exonic	ROCK1	nonsynonymous SNV	ROCK1:NM_005406:exon31:c.C3649G:p.Q1217E	=COSM1130086;OCCURENCE=1(prostat	rs201390233	B
chr18	24492378	24492378	G	C	ncRNA_intronic	AQP4-AS1	NA	NA	NA	rs56343734	NA
chr18	31322986	31322986	G	A	exonic	ASXL3	synonymous SNV	ASXL3:NM_030632:exon12:c.G3174A:p.K1058K	NA	NA	NA
chr18	42644796	42644796	C	A	UTR3	SETBP1	NA	NA	NA	NA	NA
chr18	45363933	45363933	A	-	UTR3	SMAD2	NA	NA	NA	NA	NA
chr18	47349287	47349287	T	A	UTR3	MYO5B	NA	NA	NA	NA	NA
chr18	47349335	47349335	G	A	UTR3	MYO5B	NA	NA	NA	rs199813194	NA
chr18	47349337	47349337	A	G	UTR3	MYO5B	NA	NA	NA	NA	NA
chr18	47349344	47349344	C	G	UTR3	MYO5B	NA	NA	NA	NA	NA
chr18	47349346	47349346	T	C	UTR3	MYO5B	NA	NA	NA	NA	NA
chr18	47349368	47349368	A	G	UTR3	MYO5B	NA	NA	NA	NA	NA

chr18	47349398	47349398	T	G	UTR3	MYO5B	NA	NA	NA	NA	NA
chr18	47349401	47349401	T	C	UTR3	MYO5B	NA	NA	NA	NA	NA
chr18	47350146	47350146	T	C	UTR3	MYO5B	NA	NA	NA	NA	NA
chr18	47350148	47350148	A	G	UTR3	MYO5B	NA	NA	NA	NA	NA
chr18	47350224	47350224	-	G	UTR3	MYO5B	NA	NA	NA	NA	NA
chr18	48610376	48610376	G	A	UTR3	SMAD4	NA	NA	NA	NA	NA
chr18	55215503	55215503	C	A	UTR3	FECH	NA	NA	NA	NA	NA
chr18	55289076	55289076	C	T	UTR5	NARS	NA	NA	NA	rs2032938	NA
chr18	56985789	56985789	T	C	UTR5	CPLX4	NA	NA	NA	rs59444152	NA
chr18	61265349	61265349	T	-	UTR3	SERPINB13	NA	NA	NA	rs57085048	NA
chr18	61325815	61325815	A	G	exonic	SERPINB3	nonsynonymous SNV	SERPINB3:NM_006919:exon5:c.T401C:p.V134A	NA	rs148254791	B
chr18	61325816	61325816	C	T	exonic	SERPINB3	nonsynonymous SNV	SERPINB3:NM_006919:exon5:c.G400A:p.V134I	NA	rs61754491	B
chr18	64171528	64171528	-	TT	UTR3	CDH19	NA	NA	NA	NA	NA
chr18	64171913	64171913	A	T	UTR3	CDH19	NA	NA	NA	NA	NA
chr18	72516258	72516258	-	A	UTR3	ZNF407	NA	NA	NA	NA	NA
chr18	72593013	72593013	A	C	exonic	ZNF407	nonsynonymous SNV	01146189:exon5:c.A5066C:p.D1689A,ZNF407:NM_017757:exon5:c.A5(	NA	NA	NA
chr19	111016	111016	T	G	exonic	OR4F17	nonsynonymous SNV	OR4F17:NM_001005240:exon1:c.T338G:p.F113C	NA	rs200336441	D
chr19	199295	199295	G	A	ncRNA_exonic	FLJ45445	NA	NA	NA	NA	NA
chr19	199442	199442	G	T	ncRNA_exonic	FLJ45445	NA	NA	NA	rs57399907	NA
chr19	199588	199588	T	G	ncRNA_exonic	FLJ45445	NA	NA	NA	rs8053947	NA
chr19	501695	501695	C	T	exonic	MADCAM1	nonsynonymous SNV	MADCAM1:NM_130760:exon4:c.C694T:p.P232S	NA	rs77685069	NA
chr19	501725	501725	G	A	exonic	MADCAM1	nonsynonymous SNV	MADCAM1:NM_130760:exon4:c.G724A:p.D242N	NA	rs62130833	NA
chr19	501762	501762	A	C	exonic	MADCAM1	nonsynonymous SNV	MADCAM1:NM_130760:exon4:c.A761C:p.Q254P	D=COSM475040;OCCURENCE=1(kidney	rs200007467	NA
chr19	622336	622336	T	G	exonic	POLRMT	nonsynonymous SNV	POLRMT:NM_005035:exon9:c.A1664C:p.E555A	NA	rs2238549	B
chr19	624882	624882	T	C	exonic	POLRMT	nonsynonymous SNV	POLRMT:NM_005035:exon5:c.A977G:p.E326G	NA	NA	P
chr19	871987	871987	G	A	exonic	MED16	synonymous SNV	MED16:NM_005481:exon12:c.C2037T:p.T679T	NA	rs78047294	NA
chr19	1037971	1037971	-	T	UTR3	CNN2	NA	NA	NA	NA	NA
chr19	1472591	1472591	T	C	UTR3	APC2	NA	NA	NA	NA	NA
chr19	1525392	1525392	C	T	UTR5	PLK5	NA	NA	NA	rs2292451	NA
chr19	1597572	1597572	G	T	UTR3	UQCR11	NA	NA	NA	NA	NA
chr19	1925930	1925930	G	C	UTR3	SCAMP4	NA	NA	NA	NA	NA
chr19	2290870	2290870	G	C	exonic	LINGO3	synonymous SNV	LINGO3:NM_001101391:exon2:c.C906G:p.A302A	NA	rs8111892	NA
chr19	2716453	2716453	T	G	UTR3	DIRAS1	NA	NA	NA	NA	NA
chr19	3121452	3121452	-	A	UTR3	GNA11	NA	NA	NA	NA	NA
chr19	3590837	3590837	T	A	UTR3	GIPC3	NA	NA	NA	NA	NA
chr19	3769253	3769253	G	C	UTR3	RAX2	NA	NA	NA	rs6510769	NA
chr19	4511737	4511737	T	C	exonic	PLIN4	synonymous SNV	PLIN4:NM_001080400:exon3:c.A2193G:p.K731K	NA	rs57610751	NA
chr19	4512676	4512676	T	G	exonic	PLIN4	synonymous SNV	PLIN4:NM_001080400:exon3:c.A1254C:p.T418T	NA	rs59593546	NA
chr19	4513044	4513044	C	T	exonic	PLIN4	nonsynonymous SNV	PLIN4:NM_001080400:exon3:c.G886A:p.G296S	NA	rs56366613	NA
chr19	4513045	4513045	A	G	exonic	PLIN4	synonymous SNV	PLIN4:NM_001080400:exon3:c.T885C:p.T295T	NA	rs12327614	NA
chr19	4523091	4523091	T	-	UTR3	PLIN5	NA	NA	NA	rs71168910	NA
chr19	4524016	4524016	G	A	exonic	PLIN5	nonsynonymous SNV	PLIN5:NM_001013706:exon8:c.C916T:p.R306W	NA	rs1062223	D
chr19	5915980	5915980	T	G	UTR3	CAPS	NA	NA	NA	NA	NA
chr19	6156483	6156483	T	C	exonic	ACSBG2	nonsynonymous SNV	ACSBG2:NM_030924:exon5:c.T428C:p.V143A	NA	rs4807840	B
chr19	7166376	7166376	C	T	exonic	INSR	synonymous SNV	000208:exon8:c.G1650A:p.A550A,INSR:NM_001079817:exon8:c.G165(	=COSM148469;OCCURENCE=1(stomac	rs2059806	NA
chr19	7536496	7536496	T	C	UTR3	ARHGEF18	NA	NA	NA	NA	NA
chr19	7744076	7744077	CC	-	UTR3	C19orf59	NA	NA	NA	rs149073960	NA
chr19	7965897	7965897	C	G	UTR3	LRRC8E	NA	NA	NA	NA	NA
chr19	7965898	7965898	A	G	UTR3	LRRC8E	NA	NA	NA	NA	NA
chr19	8176640	8176640	C	T	exonic	FBN3	nonsynonymous SNV	FBN3:NM_032447:exon31:c.G3976A:p.V1326I	NA	rs12975322	B
chr19	9297874	9297874	G	A	UTR3	OR7D2	NA	NA	NA	rs7246654	NA
chr19	9761701	9761701	C	T	UTR3	ZNF562	NA	NA	NA	NA	NA
chr19	10439540	10439540	A	G	exonic	RAVER1	synonymous SNV	RAVER1:NM_133452:exon3:c.T585C:p.A195A	NA	rs3745262	NA
chr19	10579172	10579172	G	C	UTR3	PDE4A	NA	NA	NA	NA	NA
chr19	10579177	10579177	A	C	UTR3	PDE4A	NA	NA	NA	NA	NA
chr19	11562297	11562297	C	T	UTR3	ELAVL3	NA	NA	NA	NA	NA
chr19	11563263	11563263	G	A	UTR3	ELAVL3	NA	NA	NA	NA	NA
chr19	11564612	11564612	G	C	UTR3	ELAVL3	NA	NA	NA	NA	NA
chr19	12428367	12428367	T	C	UTR3	ZNF563	NA	NA	NA	rs10410020	NA
chr19	12574741	12574742	AA	-	UTR3	ZNF709	NA	NA	NA	NA	NA
chr19	12575260	12575260	C	T	exonic	ZNF709	synonymous SNV	ZNF709:NM_152601:exon4:c.G1476A:p.R492R	NA	NA	NA
chr19	12810471	12810471	T	-	UTR3	TNPO2	NA	NA	NA	NA	NA
chr19	13317759	13317767	TTTTTTTTT	-	UTR3	CACNA1A	NA	NA	NA	NA	NA
chr19	14582539	14582539	C	T	exonic	PKN1	synonymous SNV	_002741:exon22:c.C2772T:p.L924L,PKN1:NM_213560:exon22:c.C279(	NA	rs8598	NA
chr19	14583582	14583582	C	T	exonic	PTGER1	synonymous SNV	PTGER1:NM_000955:exon3:c.G999A:p.L333L	NA	rs11668633	NA
chr19	15770784	15770784	G	A	UTR3	CYP4F3	NA	NA	NA	rs3087897	NA

chr19	17001071	17001071	C	A	exonic	F2RL3	nonsynonymous SNV	F2RL3:NM_003950:exon2:c.C797A:p.A266D	NA	NA	D
chr19	17038890	17038890	C	A	exonic	CPAMD8	nonsynonymous SNV	CPAMD8:NM_015692:exon25:c.G3440T:p.S1147I	NA	NA	NA
chr19	17392483	17392483	C	G	UTR5	ANKLE1	NA	NA	NA	rs7246262	NA
chr19	17393015	17393015	C	T	exonic	ANKLE1	nonsynonymous SNV	ANKLE1:NM_152363:exon2:c.C212T:p.A71V	NA	rs1864116	NA
chr19	17434197	17434197	C	A	UTR3	ANO8	NA	NA	NA	NA	NA
chr19	18260796	18260796	T	-	UTR3	MAST3	NA	NA	NA	rs61501122	NA
chr19	18474961	18474961	C	T	UTR3	PGPEP1	NA	NA	NA	NA	NA
chr19	18479249	18479251	AAA	-	UTR3	PGPEP1	NA	NA	NA	rs59054174	NA
chr19	20004073	20004109	ACAAGTTCT	-	UTR3	ZNF253	NA	NA	NA	NA	NA
chr19	20046147	20046147	G	A	UTR3	ZNF93	NA	NA	NA	rs1048421	NA
chr19	20803578	20803578	A	G	UTR3	ZNF626	NA	NA	NA	rs7250542	NA
chr19	20803652	20803652	-	T	UTR3	ZNF626	NA	NA	NA	rs139108386	NA
chr19	20804513	20804513	G	A	UTR3	ZNF626	NA	NA	NA	rs4485501	NA
chr19	20806673	20806673	C	T	UTR3	ZNF626	NA	NA	NA	NA	NA
chr19	20807270	20807270	A	G	exonic	ZNF626	synonymous SNV	ZNF626:NM_001076675:exon4:c.T1413C:p.H471H	NA	rs4809070	NA
chr19	21566239	21566239	T	C	ncRNA_exonic	ZNF738	NA	NA	NA	NA	NA
chr19	21566240	21566240	A	T	ncRNA_exonic	ZNF738	NA	NA	NA	NA	NA
chr19	21566247	21566247	C	T	ncRNA_exonic	ZNF738	NA	NA	NA	NA	NA
chr19	22363610	22363610	A	G	exonic	ZNF676	synonymous SNV	ZNF676:NM_001001411:exon3:c.T909C:p.H303H	NA	rs201622264	NA
chr19	22363795	22363795	T	C	exonic	ZNF676	nonsynonymous SNV	ZNF676:NM_001001411:exon3:c.A724G:p.K242E	NA	NA	B
chr19	22363796	22363796	A	C	exonic	ZNF676	synonymous SNV	ZNF676:NM_001001411:exon3:c.T723G:p.T241T	NA	NA	NA
chr19	22363797	22363797	G	A	exonic	ZNF676	nonsynonymous SNV	ZNF676:NM_001001411:exon3:c.C722T:p.T241I	NA	NA	B
chr19	22363803	22363803	A	T	exonic	ZNF676	nonsynonymous SNV	ZNF676:NM_001001411:exon3:c.T716A:p.I239N	NA	NA	B
chr19	22363817	22363817	A	G	exonic	ZNF676	synonymous SNV	ZNF676:NM_001001411:exon3:c.T702C:p.F234F	NA	NA	NA
chr19	33698660	33698660	G	C	UTR3	LRP3	NA	NA	NA	NA	NA
chr19	35454604	35454604	A	C	UTR5	ZNF792	NA	NA	NA	rs2546028	NA
chr19	35454605	35454605	A	G	UTR5	ZNF792	NA	NA	NA	rs2546029	NA
chr19	35850711	35850711	A	G	exonic	FFAR3	nonsynonymous SNV	FFAR3:NM_005304:exon2:c.A919G:p.M307V	NA	rs12459836	B
chr19	35851239	35851239	G	A	UTR3	FFAR3	NA	NA	NA	rs147842512	NA
chr19	36114999	36114999	C	T	UTR3	HAUS5	NA	NA	NA	NA	NA
chr19	36135645	36135645	T	A	exonic	ETV2	nonsynonymous SNV	ETV2:NM_014209:exon7:c.T920A:p.I307N	NA	NA	NA
chr19	36135646	36135646	C	G	exonic	ETV2	nonsynonymous SNV	ETV2:NM_014209:exon7:c.C921G:p.I307M	NA	NA	NA
chr19	36135647	36135647	G	A	exonic	ETV2	nonsynonymous SNV	ETV2:NM_014209:exon7:c.G922A:p.V308M	NA	NA	NA
chr19	36135649	36135649	G	C	exonic	ETV2	synonymous SNV	ETV2:NM_014209:exon7:c.G924C:p.V308V	NA	NA	NA
chr19	36135650	36135650	C	G	exonic	ETV2	nonsynonymous SNV	ETV2:NM_014209:exon7:c.C925G:p.R309G	NA	NA	NA
chr19	37036967	37036967	A	G	UTR3	ZNF529	NA	NA	NA	NA	NA
chr19	38028814	38028814	T	-	UTR3	ZNF793	NA	NA	NA	NA	NA
chr19	38031187	38031187	G	A	UTR3	ZNF793	NA	NA	NA	NA	NA
chr19	38795905	38795905	C	A	UTR3	YIF1B	NA	NA	NA	NA	NA
chr19	38819463	38819463	C	G	UTR3	KCNK6	NA	NA	NA	rs61160177	NA
chr19	39327296	39327296	C	A	UTR3	HNRNPL	NA	NA	NA	NA	NA
chr19	39360720	39360720	G	A	exonic	RINL	nonsynonymous SNV	_001195833:exon9:c.C1205T:p.P402L,RINL:NM_198445:exon9:c.C863T	NA	rs8110393	D
chr19	39805325	39805325	T	G	exonic	LRFN1	nonsynonymous SNV	LRFN1:NM_020862:exon1:c.A652C:p.T218P	NA	NA	NA
chr19	40021823	40021823	-	T	UTR3	EID2B	NA	NA	NA	NA	NA
chr19	40399814	40399814	A	G	exonic	FCGBP	nonsynonymous SNV	FCGBP:NM_003890:exon13:c.T5881C:p.S1961P	NA	NA	NA
chr19	41128309	41128309	C	T	exonic	LTBP4	unknown	UNKNOWN	NA	rs10880	NA
chr19	41349517	41349517	G	C	UTR3	CYP2A6	NA	NA	NA	rs113777046	NA
chr19	41349519	41349519	T	A	UTR3	CYP2A6	NA	NA	NA	rs112026263	NA
chr19	41456227	41456227	T	A	ncRNA_exonic	CYP2B7P1	NA	NA	NA	rs117694034	NA
chr19	41811707	41811707	T	C	exonic	HNRNPUL1	nonsynonymous SNV	_007040:exon14:c.T2389C:p.Y797H,HNRNPUL1:NM_144732:exon14:c.	NA	NA	NA
chr19	42799199	42799199	A	C	exonic	CIC	synonymous SNV	CIC:NM_015125:exon20:c.A4683C:p.S1561S	NA	NA	NA
chr19	43376101	43376101	G	T	exonic	PSG1	nonsynonymous SNV	527A:p.A176E,PSG1:NM_001184826:exon3:c.C527A:p.A176E,PSG1:NM	NA	NA	NA
chr19	43853608	43853608	A	G	ncRNA_exonic	PRG1	NA	NA	NA	rs62113732	NA
chr19	43864548	43864548	C	A	exonic	CD177	unknown	UNKNOWN	NA	rs10425835	NA
chr19	44037370	44037370	C	G	UTR5	ZNF575	NA	NA	NA	rs2599459	NA
chr19	44096007	44096007	G	A	UTR3	IRGQ	NA	NA	NA	rs28444861	NA
chr19	44096007	44096007	-	AA	UTR3	IRGQ	NA	NA	NA	NA	NA
chr19	44117603	44117603	T	A	exonic	SRRM5	nonsynonymous SNV	SRRM5:NM_001145641:exon1:c.T1330A:p.Y444N	l474846;OCCURENCE=1(kidney),5(endo	rs201848853	NA
chr19	44576302	44576302	C	T	UTR5	ZNF284	NA	NA	NA	rs11666432	NA
chr19	45323171	45323187	CCCAACTCA	-	UTR3	BCAM	NA	NA	NA	rs3842409	NA
chr19	46021198	46021198	C	A	exonic	VASP	nonsynonymous SNV	VASP:NM_003370:exon3:c.C189A:p.N63K	NA	NA	D
chr19	46030154	46030154	G	A	UTR3	VASP	NA	NA	NA	rs10995	NA
chr19	46049982	46049982	A	G	UTR3	OPA3	NA	NA	NA	rs10422253	NA
chr19	46052048	46052048	-	TTATTTAT	UTR3	OPA3	NA	NA	NA	NA	NA
chr19	46133251	46133251	C	T	exonic	EML2	synonymous SNV	p.S188S,EML2:NM_001193268:exon10:c.G1167A:p.S389S,EML2:NM_00	NA	rs7251952	NA
chr19	46133256	46133256	G	A	exonic	EML2	nonsynonymous SNV	p.L187F,EML2:NM_001193268:exon10:c.C1162T:p.L388F,EML2:NM_00	NA	rs7252175	D

chr19	46273463	46273507	AGCAGCAC	-	UTR3	DMPK	NA	NA	NA	NA	NA
chr19	46286335	46286335	T	G	UTR3	DMWD	NA	NA	NA	NA	NA
chr19	46442771	46442771	G	A	UTR3	NOVA2	NA	NA	NA	NA	NA
chr19	46823751	46823751	C	T	exonic	HIF3A	synonymous SNV	_152796:exon8:c.C870T:p.H290H,HIF3A:NM_152794:exon9:c.C1071T:	NA	rs3764610	NA
chr19	46844456	46844456	T	-	UTR3	HIF3A	NA	NA	NA	rs71769965	NA
chr19	47113764	47113764	G	C	UTR3	CALM3	NA	NA	NA	NA	NA
chr19	47507174	47507175	CG	-	UTR3	ARHGAP35	NA	NA	NA	NA	NA
chr19	47774988	47775001	CGCGCGCG	-	UTR3	CCDC9	NA	NA	NA	NA	NA
chr19	48183771	48183771	C	T	exonic	GLTSCR1	synonymous SNV	GLTSCR1:NM_015711:exon6:c.C1344T:p.S448S	NA	rs1035938	NA
chr19	48984071	48984071	C	G	UTR3	CYTH2	NA	NA	NA	NA	NA
chr19	49891522	49891522	A	G	UTR5	CCDC155	NA	NA	NA	rs9304684	NA
chr19	52273531	52273531	G	A	UTR3	FPR2	NA	NA	NA	NA	NA
chr19	52495250	52495250	A	G	UTR3	ZNF615	NA	NA	NA	rs188828596	NA
chr19	52723101	52723101	T	C	exonic	PPP2R1A	nonsynonymous SNV	PPP2R1A:NM_014225:exon10:c.T1286C:p.L429P	NA	NA	B
chr19	53381523	53381523	G	A	UTR3	ZNF320	NA	NA	NA	rs12978427	NA
chr19	53431263	53431263	C	T	ncRNA_exonic	ZNF321P	NA	NA	NA	rs7250706	NA
chr19	53946857	53946857	C	G	ncRNA_exonic	TPM3P9	NA	NA	NA	rs75274020	NA
chr19	54378026	54378027	TT	-	UTR3	MYADM	NA	NA	NA	NA	NA
chr19	54378031	54378031	T	C	UTR3	MYADM	NA	NA	NA	NA	NA
chr19	54725798	54725798	G	T	exonic	LILRB3	nonsynonymous SNV	_001081450:exon4:c.C560A:p.T187N,LILRB3:NM_006864:exon4:c.C56	=COSM1158374;OCCURENCE=1(pancre	rs1052968	B
chr19	54725835	54725835	G	C	exonic	LILRB3	nonsynonymous SNV	_001081450:exon4:c.C523G:p.R175G,LILRB3:NM_006864:exon4:c.C52	NA	rs201948566	B
chr19	54742303	54742303	C	T	UTR3	LILRA6	NA	NA	NA	rs71365449	NA
chr19	54744732	54744732	G	A	exonic	LILRA6	synonymous SNV	LILRA6:NM_024318:exon5:c.C930T:p.S310S	NA	rs79245076	NA
chr19	54818552	54818552	G	-	UTR3	LILRA5	NA	NA	NA	NA	NA
chr19	54818578	54818578	G	A	UTR3	LILRA5	NA	NA	NA	NA	NA
chr19	55255290	55255290	C	T	exonic	KIR2DL3	synonymous SNV	KIR2DL3:NM_015868:exon4:c.C418T:p.L140L	NA	rs662465	NA
chr19	55255377	55255377	C	T	exonic	KIR2DL3	nonsynonymous SNV	KIR2DL3:NM_015868:exon4:c.C505T:p.R169C	ID=COSM321260;OCCURENCE=1(lung)	rs200686594	NA
chr19	55255534	55255534	C	T	exonic	KIR2DL3	nonsynonymous SNV	KIR2DL3:NM_015868:exon4:c.C662T:p.T221I	NA	rs150145497	NA
chr19	55286864	55286864	A	C	exonic	KIR2DL1	synonymous SNV	KIR2DL1:NM_014218:exon4:c.A618C:p.P206P	NA	rs201422291	NA
chr19	55324674	55324674	-	A	exonic	KIR2DL4,LOC100287534	frameshift insertion	KIR2DL4:NM_001080772:exon6:c.801_802insA:p.S267fs	NA	rs11371265	NA
chr19	55328993	55328993	G	C	exonic	KIR3DL1	nonsynonymous SNV	KIR3DL1:NM_013289:exon2:c.G39C:p.L13F	NA	rs1142881	NA
chr19	55329021	55329021	A	G	exonic	KIR3DL1	nonsynonymous SNV	KIR3DL1:NM_013289:exon2:c.A67G:p.M23V	NA	rs1142882	NA
chr19	55358734	55358734	G	A	exonic	KIR2DS4	unknown	UNKNOWN	NA	rs150361840	NA
chr19	55359234	55359234	A	G	exonic	KIR2DS4	unknown	UNKNOWN	NA	rs113921547	NA
chr19	55360023	55360023	G	C	UTR3	KIR2DS4	NA	NA	NA	NA	NA
chr19	55607307	55607307	T	G	exonic	PPP1R12C	nonsynonymous SNV	001271618:exon9:c.A1155C:p.E385D,PPP1R12C:NM_017607:exon9:c.A	NA	NA	P
chr19	55861504	55861504	T	A	UTR3	COX6B2	NA	NA	NA	rs56986472	NA
chr19	55866173	55866173	C	A	UTR5	COX6B2	NA	NA	NA	NA	NA
chr19	55876654	55876654	G	C	UTR3	IL11	NA	NA	NA	rs4252561	NA
chr19	55941938	55941938	A	G	UTR3	SHISA7	NA	NA	NA	NA	NA
chr19	56274453	56274453	G	A	exonic	RFPL4A	nonsynonymous SNV	RFPL4A:NM_001145014:exon3:c.G776A:p.G259E	)=COSM148754;OCCURENCE=1(stomac	rs12463244	NA
chr19	56652592	56652592	G	A	UTR5	ZNF444	NA	NA	NA	rs3745836	NA
chr19	56889273	56889273	-	AGTCCTTT	ncRNA_exonic	ZNF542	NA	NA	NA	rs11280846	NA
chr19	57732640	57732640	A	C	UTR3	ZNF264	NA	NA	NA	NA	NA
chr19	58383178	58383178	A	-	UTR3	ZNF814	NA	NA	NA	NA	NA
chr19	58386285	58386285	G	A	exonic	ZNF814	nonsynonymous SNV	ZNF814:NM_001144989:exon3:c.C473T:p.A158V	NA	rs2375155	NA
chr19	58963018	58963018	C	G	UTR5	ZNF324B	NA	NA	NA	rs893187	NA
chr20	309179	309179	T	A	UTR3	SOX12	NA	NA	NA	NA	NA
chr20	309180	309180	T	A	UTR3	SOX12	NA	NA	NA	NA	NA
chr20	309181	309181	T	G	UTR3	SOX12	NA	NA	NA	NA	NA
chr20	628742	628742	G	A	UTR3	SRXN1	NA	NA	NA	rs41282132	NA
chr20	1895794	1895794	G	A	exonic	SIRPA	synonymous SNV	i129A:p.V43V,SIRPA:NM_001040022:exon3:c.G129A:p.V43V,SIRPA:NM	NA	rs1135192	NA
chr20	1895796	1895796	T	C	exonic	SIRPA	nonsynonymous SNV	.T131C:p.L44S,SIRPA:NM_001040022:exon3:c.T131C:p.L44S,SIRPA:NM	NA	rs143735290	B
chr20	1895813	1895813	A	T	exonic	SIRPA	nonsynonymous SNV	A148T:p.T50S,SIRPA:NM_001040022:exon3:c.A148T:p.T50S,SIRPA:NM	NA	rs17855609	B
chr20	1895815	1895815	A	G	exonic	SIRPA	synonymous SNV	A150G:p.T50T,SIRPA:NM_001040022:exon3:c.A150G:p.T50T,SIRPA:NM	NA	rs17853846	NA
chr20	3856254	3856254	C	G	UTR3	MAVS	NA	NA	NA	NA	NA
chr20	4706509	4706510	TT	-	UTR3	PRND	NA	NA	NA	NA	NA
chr20	4706782	4706782	-	TTTTTT	UTR3	PRND	NA	NA	NA	NA	NA
chr20	5454959	5454959	G	A	ncRNA_exonic	LOC643406	NA	NA	NA	rs501565	NA
chr20	5456942	5456942	A	T	ncRNA_exonic	LOC643406	NA	NA	NA	rs113222187	NA
chr20	17971045	17971045	A	G	UTR3	MGME1	NA	NA	NA	NA	NA
chr20	19982007	19982010	TTTT	-	UTR3	RIN2	NA	NA	NA	NA	NA
chr20	25262727	25262727	A	C	exonic	PYGB	nonsynonymous SNV	PYGB:NM_002862:exon12:c.A1462C:p.T488P	NA	NA	D
chr20	25595952	25595952	A	-	UTR3	NANP	NA	NA	NA	NA	NA
chr20	25753637	25753637	C	G	ncRNA_exonic	FAM182B	NA	NA	NA	rs75733040	NA
chr20	25753646	25753646	G	C	ncRNA_exonic	FAM182B	NA	NA	NA	rs78538493	NA

chr20	25753853	25753853	A	G	ncRNA_exonic	FAM182B	NA	NA	NA	rs76897919	NA
chr20	25754607	25754607	C	A	ncRNA_exonic	FAM182B	NA	NA	NA	rs115482967	NA
chr20	25754620	25754620	A	G	ncRNA_exonic	FAM182B	NA	NA	NA	NA	NA
chr20	25754622	25754622	A	G	ncRNA_exonic	FAM182B	NA	NA	NA	NA	NA
chr20	25754689	25754689	T	C	ncRNA_exonic	FAM182B	NA	NA	NA	rs79458516	NA
chr20	25754756	25754756	G	A	ncRNA_exonic	FAM182B	NA	NA	NA	rs76339741	NA
chr20	25754774	25754774	A	C	ncRNA_exonic	FAM182B	NA	NA	NA	NA	NA
chr20	25754782	25754782	G	T	ncRNA_exonic	FAM182B	NA	NA	NA	NA	NA
chr20	25755751	25755751	G	A	ncRNA_exonic	FAM182B	NA	NA	NA	NA	NA
chr20	25755770	25755770	C	G	ncRNA_exonic	FAM182B	NA	NA	NA	NA	NA
chr20	26063630	26063630	C	T	ncRNA_exonic	FAM182A	NA	NA	NA	NA	NA
chr20	26063651	26063651	A	G	ncRNA_exonic	FAM182A	NA	NA	NA	NA	NA
chr20	26064144	26064144	T	C	ncRNA_exonic	FAM182A	NA	NA	NA	NA	NA
chr20	26064389	26064389	A	T	ncRNA_exonic	FAM182A	NA	NA	NA	rs200923238	NA
chr20	26064397	26064397	G	C	ncRNA_exonic	FAM182A	NA	NA	NA	rs112883466	NA
chr20	26064961	26064961	G	C	ncRNA_exonic	FAM182A	NA	NA	NA	NA	NA
chr20	26084296	26084296	A	G	ncRNA_exonic	NCOR1P1	NA	NA	D=COSM477938;OCCURENCE=1(kidney	rs1998943	NA
chr20	29611898	29611898	G	C	ncRNA_exonic	FRG1B	NA	NA	NA	NA	NA
chr20	29611950	29611950	C	T	ncRNA_exonic	FRG1B	NA	NA	NA	NA	NA
chr20	29612047	29612047	-	G	ncRNA_exonic	FRG1B	NA	NA	NA	NA	NA
chr20	29612048	29612048	-	CTTC	ncRNA_exonic	FRG1B	NA	NA	NA	NA	NA
chr20	29612050	29612050	-	CGA	ncRNA_exonic	FRG1B	NA	NA	NA	NA	NA
chr20	29612052	29612052	C	T	ncRNA_exonic	FRG1B	NA	NA	NA	NA	NA
chr20	29612063	29612063	T	C	ncRNA_exonic	FRG1B	NA	NA	NA	NA	NA
chr20	29612087	29612087	C	T	ncRNA_exonic	FRG1B	NA	NA	NA	rs199702451	NA
chr20	30921472	30921472	G	A	UTR3	KIF3B	NA	NA	NA	NA	NA
chr20	32664864	32664864	-	CAG	exonic	RALY	nonframeshift insertion	?:c.641_642insCAG:p.A214delinsAS,RALY:NM_016732:exon8:c.689_69	NA	rs10649600	NA
chr20	33098206	33098206	C	T	UTR3	ITCH	NA	NA	NA	NA	NA
chr20	34818695	34818695	T	-	UTR3	EPB41L1	NA	NA	NA	NA	NA
chr20	35401969	35401969	T	G	UTR5	DSN1	NA	NA	NA	NA	NA
chr20	36845738	36845738	C	T	exonic	KIAA1755	nonsynonymous SNV	KIAA1755:NM_001029864:exon13:c.G2818A:p.E940K	NA	rs760998	B
chr20	40706453	40706453	-	TTTT	UTR3	PTPRT	NA	NA	NA	NA	NA
chr20	43955092	43955092	C	A	UTR3	SDC4	NA	NA	NA	NA	NA
chr20	43955096	43955096	C	A	UTR3	SDC4	NA	NA	NA	NA	NA
chr20	48251231	48251231	T	C	UTR3	B4GALT5	NA	NA	NA	rs235033	NA
chr20	48251757	48251757	C	T	UTR3	B4GALT5	NA	NA	NA	NA	NA
chr20	48251758	48251758	A	C	UTR3	B4GALT5	NA	NA	NA	NA	NA
chr20	48251759	48251759	C	G	UTR3	B4GALT5	NA	NA	NA	NA	NA
chr20	49367485	49367485	A	T	UTR3	PARD6B	NA	NA	NA	NA	NA
chr20	49367490	49367490	G	T	UTR3	PARD6B	NA	NA	NA	NA	NA
chr20	49367491	49367491	A	T	UTR3	PARD6B	NA	NA	NA	NA	NA
chr20	49367817	49367817	-	A	UTR3	PARD6B	NA	NA	NA	rs149372097	NA
chr20	51589531	51589531	G	A	UTR5	TSHZ2	NA	NA	NA	NA	NA
chr20	52560855	52560856	TT	-	UTR3	BCAS1	NA	NA	NA	rs74631158	NA
chr20	55213716	55213717	TT	-	UTR3	TFAP2C	NA	NA	NA	NA	NA
chr20	60709499	60709499	C	T	UTR3	LSM14B	NA	NA	NA	NA	NA
chr20	60790986	60790986	T	G	UTR3	HRH3	NA	NA	NA	NA	NA
chr20	61145196	61145196	G	A	ncRNA_exonic	C20orf166-AS1	NA	NA	NA	rs12479469	NA
chr20	61732921	61732921	T	C	ncRNA_exonic	HAR1A	NA	NA	NA	rs73147778	NA
chr20	61733110	61733110	C	T	ncRNA_exonic	HAR1A	NA	NA	NA	rs6122370	NA
chr20	61888686	61888686	A	G	ncRNA_exonic	FLJ16779	NA	NA	NA	NA	NA
chr20	61888896	61888896	A	G	ncRNA_exonic	FLJ16779	NA	NA	NA	rs35166508	NA
chr20	61888957	61888957	T	C	ncRNA_exonic	FLJ16779	NA	NA	NA	rs62207452	NA
chr20	62374441	62374441	T	C	UTR3	SLC2A4RG	NA	NA	NA	rs6742	NA
chr20	62563531	62563531	T	G	UTR3	DNAJC5	NA	NA	NA	NA	NA
chr21	9909179	9909179	C	A	ncRNA_exonic	TEKT4P2	NA	NA	NA	rs1052872	NA
chr21	9909208	9909208	C	T	ncRNA_exonic	TEKT4P2	NA	NA	NA	rs4061664	NA
chr21	9909218	9909218	G	A	ncRNA_exonic	TEKT4P2	NA	NA	NA	rs1052870	NA
chr21	9909219	9909219	T	C	ncRNA_exonic	TEKT4P2	NA	NA	NA	rs1052868	NA
chr21	10942995	10942995	G	A	exonic	TPTE	stopgain SNV	78T:p.R160X,TPTE:NM_199259:exon11:c.C538T:p.R180X,TPTE:NM_19	ID=COSM376393;OCCURENCE=2(lung)	rs147014138	NA
chr21	14414855	14414855	A	G	ncRNA_exonic	ANKRD30BP2	NA	NA	NA	rs201948955	NA
chr21	15481168	15481168	C	A	UTR3	LIPI	NA	NA	NA	rs62208635	NA
chr21	28338423	28338423	C	G	exonic	ADAMTS5	synonymous SNV	ADAMTS5:NM_007038:exon1:c.G288C:p.R96R	NA	rs55933916	NA
chr21	28338953	28338953	G	-	UTR5	ADAMTS5	NA	NA	NA	NA	NA
chr21	28339012	28339012	T	A	UTR5	ADAMTS5	NA	NA	NA	NA	NA
chr21	28339031	28339031	C	A	UTR5	ADAMTS5	NA	NA	NA	NA	NA

chr21	30445948	30445948	G	A	UTR5	CCT8	NA	NA	NA	rs2070610	NA
chr21	32491788	32491788	-	A	UTR3	TIAM1	NA	NA	NA	rs113766273	NA
chr21	34809434	34809434	T	-	UTR3	IFNGR2	NA	NA	NA	rs71657077	NA
chr21	38081480	38081480	C	T	exonic	SIM2	nonsynonymous SNV	NM_005069:exon2:c.C188T:p.A63V,SIM2:NM_009586:exon2:c.C188T:p	NA	rs116988298	B
chr21	40562156	40562156	-	T	UTR3	BRWD1	NA	NA	NA	rs5843953	NA
chr21	42551319	42551319	T	G	exonic	PLAC4	synonymous SNV	PLAC4:NM_182832:exon1:c.A237C:p.S79S	NA	NA	NA
chr21	43099670	43099670	T	C	ncRNA_exonic	LINC00111	NA	NA	NA	rs73216940	NA
chr21	44838931	44838931	A	G	exonic	SIK1	nonsynonymous SNV	SIK1:NM_173354:exon11:c.T1432C:p.S478P	NA	NA	P
chr21	44838946	44838946	T	G	exonic	SIK1	nonsynonymous SNV	SIK1:NM_173354:exon11:c.A1417C:p.T473P	NA	NA	B
chr21	45553596	45553596	T	C	exonic	C21orf33	nonsynonymous SNV	3:NM_004649:exon1:c.T17C:p.V6A,C21orf33:NM_198155:exon1:c.T17	NA	rs968714	NA
chr21	45564932	45564932	T	-	UTR3	C21orf33	NA	NA	NA	rs66662664	NA
chr21	45953778	45953778	G	T	exonic	TSPEAR	nonsynonymous SNV	I_144991:exon3:c.C332A:p.A111E,TSPEAR:NM_001272037:exon4:c.C1	NA	NA	B
chr21	46711357	46711357	T	C	ncRNA_exonic	LOC642852	NA	NA	NA	rs4819053	NA
chr21	47532093	47532093	G	A	exonic	COL6A2	nonsynonymous SNV	.6A:p.E106K,COL6A2:NM_058174:exon3:c.G316A:p.E106K,COL6A2:NM	NA	rs141703710	D
chr21	47721985	47721985	-	TGG	exonic	C21orf58	nonframeshift insertion	C21orf58:NM_058180:exon8:c.897_898insCCA:p.H299delinsHH	NA	rs71318063	NA
chr22	18778584	18778584	A	G	ncRNA_exonic	GGT3P	NA	NA	NA	rs3984302	NA
chr22	18900641	18900641	T	G	UTR3	PRODH	NA	NA	NA	NA	NA
chr22	18900645	18900645	C	G	UTR3	PRODH	NA	NA	NA	NA	NA
chr22	18978385	18978385	A	C	ncRNA_exonic	DGCR5	NA	NA	NA	NA	NA
chr22	19189003	19189003	-	C	exonic	CLTCL1	frameshift insertion	exon23:c.3602_3603insG:p.V1201fs,CLTCL1:NM_007098:exon23:c.3602	NA	rs78649162	NA
chr22	19492888	19492888	G	A	exonic	CDC45	nonsynonymous SNV	DA:p.M190I,CDC45:NM_003504:exon10:c.G708A:p.M236I,CDC45:NM_	NA	NA	B
chr22	19511055	19511055	C	A	UTR3	CLDN5	NA	NA	NA	rs756654	NA
chr22	19834263	19834263	C	A	UTR3	C22orf29	NA	NA	NA	NA	NA
chr22	20385740	20385743	TGTC	-	ncRNA_exonic	PI4KAP1	NA	NA	NA	rs146242498	NA
chr22	21044156	21044156	C	A	ncRNA_exonic	POM121L4P	NA	NA	NA	rs192873282	NA
chr22	21638467	21638467	C	T	ncRNA_exonic	POM121L8P	NA	NA	NA	rs201227017	NA
chr22	21647455	21647455	G	A	ncRNA_exonic	POM121L8P	NA	NA	NA	rs140413	NA
chr22	21649095	21649095	C	-	ncRNA_exonic	POM121L8P	NA	NA	NA	NA	NA
chr22	21838339	21838339	C	T	ncRNA_exonic	PI4KAP2	NA	NA	NA	rs201760046	NA
chr22	22311882	22311882	G	A	exonic	TOP3B	synonymous SNV	TOP3B:NM_003935:exon18:c.C2193T:p.S731S	NA	rs145256528	NA
chr22	22874497	22874497	A	G	UTR5	ZNF280A	NA	NA	NA	rs4822091	NA
chr22	24227246	24227246	A	C	UTR3	SLC2A11	NA	NA	NA	rs6003939	NA
chr22	24314148	24314148	A	C	UTR3	DDTL	NA	NA	NA	NA	NA
chr22	24314187	24314187	G	C	UTR3	DDTL	NA	NA	NA	NA	NA
chr22	24659734	24659734	T	C	ncRNA_exonic	POM121L9P	NA	NA	NA	NA	NA
chr22	24659741	24659741	G	A	ncRNA_exonic	POM121L9P	NA	NA	NA	NA	NA
chr22	24981641	24981641	T	G	UTR3	FAM211B	NA	NA	NA	NA	NA
chr22	25023893	25023893	G	A	exonic	GGT1	nonsynonymous SNV	001032365:exon13:c.G1283A:p.S428N,GGT1:NM_005265:exon13:c.G	OSM137241;OCCURENCE=7(kidney),1(	rs201313233	B
chr22	25042887	25042887	G	A	ncRNA_exonic	POM121L10P	NA	NA	NA	rs75939659	NA
chr22	25044743	25044743	A	G	ncRNA_exonic	POM121L10P	NA	NA	NA	rs71242990	NA
chr22	25264460	25264460	T	C	exonic	SGSM1	nonsynonymous SNV	001098497:exon11:c.T1112C:p.L371P,SGSM1:NM_001098498:exon11:	NA	NA	NA
chr22	25603066	25603066	C	G	exonic	CRYBB3	nonsynonymous SNV	CRYBB3:NM_004076:exon6:c.C523G:p.R175G	NA	NA	P
chr22	26847960	26847974	CGCGCGCG	GCA	UTR3	HPS4	NA	NA	NA	NA	NA
chr22	27066019	27066019	-	T	ncRNA_exonic	MIAT	NA	NA	NA	NA	NA
chr22	29885823	29885823	A	T	exonic	NEFH	nonsynonymous SNV	NEFH:NM_021076:exon4:c.A2194T:p.T732S	NA	rs145125701	NA
chr22	29932727	29932727	C	A	exonic	THOC5	nonsynonymous SNV	_003678:exon7:c.G600T:p.R200S,THOC5:NM_001002877:exon8:c.G60	NA	NA	P
chr22	30221159	30221159	G	A	exonic	ASCC2	nonsynonymous SNV	M_001242906:exon3:c.C169T:p.P57S,ASCC2:NM_032204:exon4:c.C328	NA	NA	B
chr22	31677586	31677586	T	-	UTR3	PIK3IP1	NA	NA	NA	rs11301764	NA
chr22	32353392	32353392	T	-	UTR3	YWHAH	NA	NA	NA	rs71790951	NA
chr22	33256901	33256901	T	-	UTR3	TIMP3	NA	NA	NA	NA	NA
chr22	36587952	36587952	C	T	exonic	APOL4	unknown	UNKNOWN	NA	rs2227169	NA
chr22	36662951	36662951	A	-	UTR3	APOL1	NA	NA	NA	rs11321251	NA
chr22	38120503	38120503	G	A	exonic	TRIOBP	nonsynonymous SNV	TRIOBP:NM_001039141:exon7:c.G1940A:p.S647N	NA	NA	B
chr22	38823832	38823832	A	C	exonic	KCNJ4	synonymous SNV	M_004981:exon2:c.T306G:p.G102G,KCNJ4:NM_152868:exon2:c.T306G	NA	NA	NA
chr22	39414807	39414807	C	A	UTR3	APOBEC3C	NA	NA	NA	NA	NA
chr22	40720295	40720295	T	-	UTR3	TNRC6B	NA	NA	NA	rs71722737	NA
chr22	42524795	42524795	A	G	exonic	CYP2D6	synonymous SNV	I_001025161:exon3:c.T504C:p.F168F,CYP2D6:NM_000106:exon4:c.T6	NA	rs28371713	NA
chr22	46436143	46436143	-	T	ncRNA_exonic	LOC100271722	NA	NA	NA	NA	NA
chr22	46712068	46712068	C	A	exonic	GTSE1	synonymous SNV	GTSE1:NM_016426:exon7:c.C1191A:p.A397A	NA	NA	NA
chr22	50655686	50655686	C	G	exonic	SELO	unknown	UNKNOWN	NA	NA	B
chrX	3523054	3523057	ACAC	-	UTR3	PRKX	NA	NA	NA	NA	NA
chrX	5810090	5810090	A	G	UTR3	NLGN4X	NA	NA	NA	rs3810685	NA
chrX	7269977	7269977	A	-	UTR3	STS	NA	NA	NA	NA	NA
chrX	7272225	7272225	G	A	UTR3	STS	NA	NA	NA	rs13648	NA
chrX	7811747	7811747	T	C	exonic	VCX	nonsynonymous SNV	VCX:NM_013452:exon3:c.T311C:p.L104P	NA	rs71247277	NA
chrX	8434161	8434161	G	A	exonic	VCX3B	nonsynonymous SNV	VCX3B:NM_001001888:exon3:c.G478A:p.V160M	NA	rs6640132	NA

chrX	10416148	10416148	T	-	UTR3	MID1	NA	NA	NA	NA	NA
chrX	12841434	12841434	C	T	UTR3	PRPS2	NA	NA	NA	NA	NA
chrX	15511350	15511350	T	G	UTR5	PIR	NA	NA	NA	NA	NA
chrX	19552343	19552343	G	T	UTR3	SH3KBP1	NA	NA	NA	NA	NA
chrX	20134980	20134980	G	A	exonic	MAP7D2	synonymous SNV	:.C18T:p.G6G,MAP7D2:NM_001168466:exon1:c.C18T:p.G6G,MAP7D2:	NA	rs17523345	NA
chrX	23761386	23761386	C	G	UTR5	ACOT9	NA	NA	NA	rs5925605	NA
chrX	30748293	30748293	T	C	UTR3	GK	NA	NA	NA	rs5972221	NA
chrX	30748338	30748338	T	C	UTR3	GK	NA	NA	NA	rs73452162	NA
chrX	31196550	31196550	T	-	UTR3	DMD	NA	NA	NA	rs11333372	NA
chrX	35816613	35816613	G	A	UTR5	MAGEB16	NA	NA	NA	rs138684392	NA
chrX	37027663	37027663	A	C	exonic	FAM47C	nonsynonymous SNV	FAM47C:NM_001013736:exon1:c.A1180C:p.T394P	NA	NA	NA
chrX	37027874	37027874	T	C	exonic	FAM47C	nonsynonymous SNV	FAM47C:NM_001013736:exon1:c.T1391C:p.L464P	NA	NA	NA
chrX	37028069	37028069	T	C	exonic	FAM47C	nonsynonymous SNV	FAM47C:NM_001013736:exon1:c.T1586C:p.I529T	NA	NA	NA
chrX	37028182	37028182	A	G	exonic	FAM47C	nonsynonymous SNV	FAM47C:NM_001013736:exon1:c.A1699G:p.M567V	NA	NA	NA
chrX	37028402	37028402	A	C	exonic	FAM47C	nonsynonymous SNV	FAM47C:NM_001013736:exon1:c.A1919C:p.Y640S	NA	NA	NA
chrX	38664818	38664818	T	-	UTR3	MID1IP1	NA	NA	NA	NA	NA
chrX	41093299	41093299	T	-	UTR3	USP9X	NA	NA	NA	NA	NA
chrX	46358117	46358117	C	T	UTR3	ZNF674	NA	NA	NA	rs55678135	NA
chrX	47107351	47107351	A	-	UTR3	USP11	NA	NA	NA	NA	NA
chrX	48457034	48457034	-	C	UTR5	WDR13	NA	NA	NA	NA	NA
chrX	48925016	48925016	G	A	exonic	CCDC120	nonsynonymous SNV	)1163323:exon10:c.G1225A:p.A409T,CCDC120:NM_001271835:exon1	NA	NA	P
chrX	49178517	49178517	T	C	UTR5	GAGE12J	NA	NA	NA	NA	NA
chrX	53405283	53405283	C	A	UTR3	SMC1A	NA	NA	NA	NA	NA
chrX	54222891	54222891	-	A	UTR3	WNK3	NA	NA	NA	NA	NA
chrX	54224160	54224160	-	A	UTR3	WNK3	NA	NA	NA	NA	NA
chrX	54587505	54587506	AA	-	UTR3	GNL3L	NA	NA	NA	rs148333990	NA
chrX	55172630	55172630	G	A	exonic	FAM104B	stopgain SNV	/_001166701:exon3:c.C235T:p.Q79X,FAM104B:NM_001166702:exon3	NA	rs113263757	NA
chrX	55478566	55478566	G	T	UTR5	MAGEH1	NA	NA	NA	NA	NA
chrX	68836245	68836245	T	C	exonic	EDA	synonymous SNV	:c.T93C:p.P31P,EDA:NM_001005612:exon1:c.T93C:p.P31P,EDA:NM_0(	NA	NA	NA
chrX	70387344	70387344	A	C	exonic	NLGN3	nonsynonymous SNV	?77C:p.N426T,NLGN3:NM_018977:exon6:c.A1337C:p.N446T,NLGN3:NI	NA	NA	D
chrX	70520238	70520238	T	-	ncRNA_UTR3	NONO	NA	NA	NA	rs34459863	NA
chrX	72297562	72297565	ACAC	-	UTR3	PABPC1L2A	NA	NA	NA	NA	NA
chrX	72298326	72298326	T	C	UTR3	PABPC1L2A	NA	NA	NA	rs182890337	NA
chrX	72346969	72346969	A	C	ncRNA_exonic	NAP1L6	NA	NA	NA	NA	NA
chrX	74590359	74590368	TTTTTTTTTT	-	UTR3	ZDHHC15	NA	NA	NA	NA	NA
chrX	77268407	77268407	C	A	exonic	ATP7A	nonsynonymous SNV	ATP7A:NM_000052:exon10:c.C2204A:p.A735D	NA	NA	D
chrX	86924601	86924601	A	-	UTR3	KLHL4	NA	NA	NA	NA	NA
chrX	1.09E+08	1.09E+08	T	-	UTR3	TMEM164	NA	NA	NA	rs34813944	NA
chrX	1.1E+08	1.1E+08	C	T	UTR3	CHRDL1	NA	NA	NA	rs12007691	NA
chrX	1.1E+08	1.1E+08	A	-	downstream	PAK3	NA	NA	NA	rs67997504	NA
chrX	1.11E+08	1.11E+08	AA	-	UTR3	DCX	NA	NA	NA	NA	NA
chrX	1.2E+08	1.2E+08	GT	-	UTR3	MCTS1	NA	NA	NA	rs10563395	NA
chrX	1.31E+08	1.31E+08	GAGAGAGA	-	UTR3	RAP2C	NA	NA	NA	NA	NA
chrX	1.31E+08	1.31E+08	C	A	UTR3	RAP2C	NA	NA	NA	rs200211416	NA
chrX	1.36E+08	1.36E+08	T	C	exonic	RBMX	synonymous SNV	RBMX:NM_002139:exon5:c.A507G:p.G169G	NA	rs112800567	NA
chrX	1.36E+08	1.36E+08	G	C	exonic	RBMX	nonsynonymous SNV	RBMX:NM_002139:exon5:c.C499G:p.P167A	;OCCURENCE=1(haematopoietic_and_l	rs112089728	D
chrX	1.36E+08	1.36E+08	C	A	exonic	RBMX	nonsynonymous SNV	RBMX:NM_002139:exon5:c.G473T:p.G158V	NA	rs78702689	B
chrX	1.41E+08	1.41E+08	A	T	exonic	MAGEC1	synonymous SNV	MAGEC1:NM_005462:exon4:c.A591T:p.P197P	NA	rs151047723	NA
chrX	1.41E+08	1.41E+08	A	-	UTR3	MAGEC2	NA	NA	NA	NA	NA
chrX	1.45E+08	1.45E+08	TTTT	-	UTR3	SLITRK2	NA	NA	NA	NA	NA
chrX	1.5E+08	1.5E+08	C	A	exonic	MAMLD1	nonsynonymous SNV	IA:p.T125N,MAMLD1:NM_001177465:exon3:c.C374A:p.T125N,MAMLI	NA	NA	P
chrX	1.53E+08	1.53E+08	T	C	UTR3	BGN	NA	NA	NA	rs1126598	NA
chrX	1.53E+08	1.53E+08	CAACCGCCA	-	UTR3	BGN	NA	NA	NA	rs71869600	NA
chrX	1.53E+08	1.53E+08	T	G	UTR3	DUSP9	NA	NA	NA	NA	NA
chrX	1.53E+08	1.53E+08	T	G	UTR3	HCFC1	NA	NA	COSN414031;OCCURENCE=1(urinary_tr	NA	NA
chrX	1.53E+08	1.53E+08	T	G	exonic	OPN1LW	nonsynonymous SNV	OPN1LW:NM_020061:exon3:c.T538G:p.S180A	NA	rs949431	B
chr1	14699	14699	C	G	ncRNA_exonic	WASH7P	NA	NA	NA	rs11490464	NA
chr1	15817	15817	G	T	ncRNA_exonic	WASH7P	NA	NA	NA	rs2691316	NA
chr1	15820	15820	G	T	ncRNA_exonic	WASH7P	NA	NA	NA	rs200482301	NA
chr1	17626	17626	G	A	ncRNA_exonic	WASH7P	NA	NA	NA	rs11555814	NA
chr1	17697	17697	G	C	ncRNA_exonic	WASH7P	NA	NA	NA	rs71260069	NA
chr1	69511	69511	A	G	exonic	OR4F5	nonsynonymous SNV	OR4F5:NM_001005484:exon1:c.A421G:p.T141A	NA	rs2691305	B
chr1	789141	789141	G	C	ncRNA_exonic	LOC643837	NA	NA	NA	rs111606675	NA
chr1	900718	900721	TTAT	-	UTR3	KLHL17	NA	NA	NA	rs142545439	NA
chr1	985362	985362	G	T	exonic	AGRN	nonsynonymous SNV	AGRN:NM_198576:exon27:c.G4824T:p.E1608D	NA	NA	B
chr1	1019753	1019753	A	G	exonic	C1orf159	nonsynonymous SNV	C1orf159:NM_017891:exon9:c.T482C:p.I161T	NA	NA	P

chr1	1139202	1139202	T	C	exonic	TNFRSF18	synonymous SNV	TNFRSF18:NM_148901:exon4:c.A537G:p.P179P	NA	rs2298213	NA
chr1	1263689	1263689	T	G	UTR3	GLTPD1	NA	NA	NA	NA	NA
chr1	1310668	1310668	A	C	UTR5	AURKAIP1	NA	NA	NA	rs2242398	NA
chr1	1650801	1650801	T	C	exonic	CDK11A,CDK11B	synonymous SNV	321G:p.E107E,CDK11B:NM_033493:exon4:c.A321G:p.E107E,CDK11A:N	NA	rs1137004	NA
chr1	2120733	2120733	-	G	intronic	C1orf86	NA	NA	NA	rs146903099	NA
chr1	3319390	3319390	C	T	exonic	PRDM16	nonsynonymous SNV	M_022114:exon6:c.C712T:p.L238F,PRDM16:NM_199454:exon6:c.C71	NA	NA	NA
chr1	3352764	3352765	TT	-	UTR3	PRDM16	NA	NA	NA	NA	NA
chr1	3418391	3418391	C	T	exonic	MEGF6	synonymous SNV	MEGF6:NM_001409:exon18:c.G2283A:p.P761P	NA	rs4648508	NA
chr1	3656396	3656396	C	T	ncRNA_exonic	TP73-AS1	NA	NA	NA	NA	NA
chr1	3656558	3656558	T	C	ncRNA_exonic	TP73-AS1	NA	NA	NA	NA	NA
chr1	3656605	3656605	T	C	ncRNA_exonic	TP73-AS1	NA	NA	NA	NA	NA
chr1	3656637	3656637	T	A	ncRNA_exonic	TP73-AS1	NA	NA	NA	NA	NA
chr1	3696890	3696890	A	C	UTR3	LRRC47	NA	NA	NA	rs8379	NA
chr1	4472155	4472155	A	-	ncRNA_exonic	LOC284661	NA	NA	NA	rs34773421	NA
chr1	6308965	6308965	T	G	UTR3	GPR153	NA	NA	NA	NA	NA
chr1	6308981	6308981	A	G	UTR3	GPR153	NA	NA	NA	NA	NA
chr1	6501044	6501044	C	G	exonic	ESPN	synonymous SNV	ESPN:NM_031475:exon5:c.C909G:p.R303R	NA	rs2311045	NA
chr1	6526465	6526465	C	G	UTR3	PLEKHG5	NA	NA	NA	rs2986754	NA
chr1	6531124	6531124	T	C	exonic	PLEKHG5	nonsynonymous SNV	001265594:exon13:c.A1318G:p.M440V,PLEKHG5:NM_020631:exon13:	NA	rs61740145	P
chr1	6531589	6531589	C	T	exonic	PLEKHG5	nonsynonymous SNV	001265594:exon12:c.G1240A:p.A414T,PLEKHG5:NM_020631:exon12:	NA	rs74809741	B
chr1	6550505	6550505	C	T	UTR5	PLEKHG5	NA	NA	NA	rs11806429	NA
chr1	6682289	6682289	T	G	UTR3	PHF13	NA	NA	NA	NA	NA
chr1	7828174	7828175	AA	-	UTR3	CAMTA1	NA	NA	NA	NA	NA
chr1	7907805	7907805	-	T	UTR3	UTS2	NA	NA	NA	rs112820691	NA
chr1	9328298	9328298	A	-	UTR3	H6PD	NA	NA	NA	NA	NA
chr1	9780836	9780836	T	G	exonic	PIK3CD	nonsynonymous SNV	PIK3CD:NM_005026:exon13:c.T1558G:p.S520A	NA	rs201274224	B
chr1	9910659	9910659	G	A	UTR3	CTNNBIP1	NA	NA	NA	rs71643073	NA
chr1	11014118	11014118	C	T	exonic	C1orf127	nonsynonymous SNV	C1orf127:NM_001170754:exon10:c.G1057A:p.A353T	NA	rs45537241	P
chr1	11562913	11562913	C	T	exonic	PTCHD2	synonymous SNV	PTCHD2:NM_020780:exon3:c.C1275T:p.F425F	NA	rs60362998	NA
chr1	11796169	11796173	GGGCG	-	UTR5	AGTRAP	NA	NA	NA	rs146190997	NA
chr1	11846092	11846092	-	T	UTR3	MTHFR	NA	NA	NA	rs112870018	NA
chr1	12888545	12888545	C	T	UTR5	PRAMEF11	NA	NA	NA	rs2982101	NA
chr1	12888569	12888569	G	C	UTR5	PRAMEF11	NA	NA	NA	rs78453011	NA
chr1	12888595	12888595	G	C	UTR5	PRAMEF11	NA	NA	NA	rs76039145	NA
chr1	12919642	12919642	G	A	exonic	PRAMEF2	nonsynonymous SNV	PRAMEF2:NM_023014:exon3:c.G382A:p.A128T	NA	rs142476002	B
chr1	12919833	12919833	A	G	exonic	PRAMEF2	synonymous SNV	PRAMEF2:NM_023014:exon3:c.A573G:p.P191P	)=COSM246757;OCCURENCE=1(prostat	NA	NA
chr1	12919840	12919840	T	C	exonic	PRAMEF2	nonsynonymous SNV	PRAMEF2:NM_023014:exon3:c.T580C:p.Y194H	)=COSM246759;OCCURENCE=1(prostat	NA	B
chr1	12921127	12921127	A	G	exonic	PRAMEF2	synonymous SNV	PRAMEF2:NM_023014:exon4:c.A918G:p.L306L	NA	rs12139549	NA
chr1	12921136	12921136	C	T	exonic	PRAMEF2	synonymous SNV	PRAMEF2:NM_023014:exon4:c.C927T:p.D309D	21628;OCCURENCE=1(upper_aerodiges	NA	NA
chr1	12921137	12921137	T	G	exonic	PRAMEF2	nonsynonymous SNV	PRAMEF2:NM_023014:exon4:c.T928G:p.L310V	NA	NA	B
chr1	12921635	12921635	G	A	UTR3	PRAMEF2	NA	NA	NA	rs144099057	NA
chr1	12939692	12939692	G	A	exonic	PRAMEF4	synonymous SNV	PRAMEF4:NM_001009611:exon4:c.C1110T:p.N370N	NA	rs142785237	NA
chr1	12939733	12939733	G	A	exonic	PRAMEF4	synonymous SNV	PRAMEF4:NM_001009611:exon4:c.C1069T:p.L357L	NA	rs11578354	NA
chr1	12939904	12939904	A	C	exonic	PRAMEF4	nonsynonymous SNV	PRAMEF4:NM_001009611:exon4:c.T898G:p.F300V	NA	rs148317829	NA
chr1	12942955	12942955	C	A	exonic	PRAMEF4	synonymous SNV	PRAMEF4:NM_001009611:exon2:c.G261T:p.L87L	NA	rs11581721	NA
chr1	12942963	12942963	C	T	exonic	PRAMEF4	nonsynonymous SNV	PRAMEF4:NM_001009611:exon2:c.G253A:p.D85N	NA	rs200120101	NA
chr1	12942981	12942981	C	T	exonic	PRAMEF4	nonsynonymous SNV	PRAMEF4:NM_001009611:exon2:c.G235A:p.A79T	NA	rs199923650	NA
chr1	12943042	12943042	C	T	exonic	PRAMEF4	synonymous SNV	PRAMEF4:NM_001009611:exon2:c.G174A:p.V58V	OSM300667;OCCURENCE=1(large_intes	rs148309168	NA
chr1	12954490	12954490	A	G	exonic	PRAMEF10	nonsynonymous SNV	PRAMEF10:NM_001039361:exon3:c.T793C:p.C265R	NA	rs149281133	P
chr1	13183213	13183213	T	A	exonic	LOC440563	synonymous SNV	LOC440563:NM_001136561:exon2:c.A660T:p.S220S	NA	rs28373323	NA
chr1	13183225	13183225	T	C	exonic	LOC440563	synonymous SNV	LOC440563:NM_001136561:exon2:c.A648G:p.K216K	NA	rs28634306	NA
chr1	13183228	13183228	C	T	exonic	LOC440563	synonymous SNV	LOC440563:NM_001136561:exon2:c.G645A:p.V215V	NA	rs144054379	NA
chr1	13183833	13183833	C	T	exonic	LOC440563	nonsynonymous SNV	LOC440563:NM_001136561:exon2:c.G40A:p.V14M	NA	rs115597766	NA
chr1	13328199	13328199	A	C	UTR3	PRAMEF3	NA	NA	NA	rs56684054	NA
chr1	13328239	13328239	C	A	UTR3	PRAMEF3	NA	NA	NA	rs28394386	NA
chr1	13328576	13328576	C	T	UTR3	PRAMEF3	NA	NA	NA	rs28538410	NA
chr1	13328729	13328729	A	G	UTR3	PRAMEF3	NA	NA	NA	rs60101915	NA
chr1	15438826	15438839	GTGTGTGT	-	ncRNA_exonic	TMEM51-AS1	NA	NA	NA	rs139705142	NA
chr1	15546873	15546874	AA	-	UTR3	TMEM51	NA	NA	NA	rs199943887	NA
chr1	15911597	15911597	A	G	UTR5	AGMAT	NA	NA	NA	rs6687585	NA
chr1	16111413	16111413	C	-	UTR3	FBLIM1	NA	NA	NA	rs149301626	NA
chr1	16160955	16160955	T	-	ncRNA_exonic	FLJ37453	NA	NA	NA	NA	NA
chr1	16162978	16162978	T	-	ncRNA_exonic	FLJ37453	NA	NA	NA	NA	NA
chr1	16375063	16375063	C	G	exonic	CLCNKB	nonsynonymous SNV	CLCNKB:NM_000085:exon7:c.C641G:p.A214G	NA	rs1889789	B
chr1	16375064	16375064	A	C	exonic	CLCNKB	synonymous SNV	CLCNKB:NM_000085:exon7:c.A642C:p.A214A	NA	rs1889790	NA
chr1	16890441	16890441	C	T	exonic	NBPF1	unknown	UNKNOWN	NA	rs3863779	NA

chr1	16892249	16892249	T	G	exonic	NBPF1	unknown	UNKNOWN	NA	rs201179082	NA
chr1	16905718	16905718	A	C	exonic	NBPF1	unknown	UNKNOWN	NA	rs10909417	NA
chr1	16915434	16915434	T	C	exonic	NBPF1	unknown	UNKNOWN	NA	rs202129231	NA
chr1	16945236	16945239	TTAT	-	ncRNA_exonic	CROCCP2	NA	NA	NA	NA	NA
chr1	16954981	16954981	C	A	ncRNA_exonic	CROCCP2	NA	NA	NA	rs10157498	NA
chr1	16974758	16974758	G	A	ncRNA_exonic	MST1P2	NA	NA	NA	rs28484638	NA
chr1	16974780	16974780	C	T	ncRNA_exonic	MST1P2	NA	NA	NA	rs28537673	NA
chr1	16975005	16975005	C	T	ncRNA_exonic	MST1P2	NA	NA	NA	rs75691517	NA
chr1	16975699	16975699	A	C	ncRNA_exonic	MST1P2	NA	NA	NA	rs11488480	NA
chr1	17023376	17023376	C	T	ncRNA_exonic	ESPNP	NA	NA	NA	rs613579	NA
chr1	17023403	17023403	G	A	ncRNA_exonic	ESPNP	NA	NA	NA	rs10907267	NA
chr1	17046549	17046549	G	A	ncRNA_exonic	ESPNP	NA	NA	NA	rs1755332	NA
chr1	17046595	17046595	T	C	ncRNA_exonic	ESPNP	NA	NA	NA	rs3856293	NA
chr1	17081606	17081606	A	C	UTR3	MST1L	NA	NA	NA	rs28661960	NA
chr1	17081886	17081886	T	C	UTR3	MST1L	NA	NA	NA	rs12117984	NA
chr1	17082229	17082229	G	C	UTR3	MST1L	NA	NA	NA	rs2610653	NA
chr1	17082238	17082238	T	C	UTR3	MST1L	NA	NA	NA	NA	NA
chr1	17082487	17082487	G	A	UTR3	MST1L	NA	NA	NA	rs180726840	NA
chr1	17082779	17082779	A	G	UTR3	MST1L	NA	NA	NA	NA	NA
chr1	17082839	17082839	A	C	UTR3	MST1L	NA	NA	NA	NA	NA
chr1	17083075	17083075	T	G	UTR3	MST1L	NA	NA	NA	NA	NA
chr1	17083119	17083119	A	T	UTR3	MST1L	NA	NA	NA	NA	NA
chr1	17083597	17083597	G	A	UTR3	MST1L	NA	NA	NA	rs186155999	NA
chr1	17083778	17083778	C	T	exonic	MST1L	synonymous SNV	MST1L:NM_001271733:exon15:c.G2019A:p.T673T	NA	rs28455793	NA
chr1	17083888	17083888	C	T	exonic	MST1L	nonsynonymous SNV	MST1L:NM_001271733:exon15:c.G1909A:p.G637S	NA	rs1071687	NA
chr1	17085447	17085447	G	A	exonic	MST1L	nonsynonymous SNV	MST1L:NM_001271733:exon10:c.C1244T:p.P415L	NA	rs202225848	NA
chr1	17085995	17085995	-	C	exonic	MST1L	frameshift insertion	MST1L:NM_001271733:exon7:c.902_903insG:p.A301fs	NA	NA	NA
chr1	17086085	17086085	-	C	exonic	MST1L	frameshift insertion	MST1L:NM_001271733:exon7:c.812_813insG:p.A271fs	NA	rs200532237	NA
chr1	17265416	17265416	G	T	exonic	CROCC	nonsynonymous SNV	CROCC:NM_014675:exon12:c.G1387T:p.D463Y	NA	rs114323849	D
chr1	17299014	17299014	A	C	UTR3	CROCC	NA	NA	NA	rs6661019	NA
chr1	17396592	17396592	G	A	exonic	PADI2	synonymous SNV	PADI2:NM_007365:exon15:c.C1755T:p.F585F	NA	rs11581590	NA
chr1	17674537	17674537	C	A	exonic	PADI4	synonymous SNV	PADI4:NM_012387:exon10:c.C1149A:p.R383R	NA	rs2240335	NA
chr1	18809292	18809292	A	C	exonic	KLHDC7A	nonsynonymous SNV	KLHDC7A:NM_152375:exon1:c.A1817C:p.D606A	NA	NA	D
chr1	18812479	18812479	A	-	UTR3	KLHDC7A	NA	NA	NA	rs72275238	NA
chr1	19665557	19665557	A	C	UTR3	CAPZB	NA	NA	NA	NA	NA
chr1	19983863	19983863	-	G	UTR3	C1orf151-NBL1,NBL1	NA	NA	NA	NA	NA
chr1	19992066	19992066	T	C	UTR5	HTR6	NA	NA	NA	rs10917509	NA
chr1	19992204	19992204	T	C	UTR5	HTR6	NA	NA	NA	NA	NA
chr1	19992211	19992211	A	C	UTR5	HTR6	NA	NA	NA	NA	NA
chr1	19992219	19992219	A	C	UTR5	HTR6	NA	NA	NA	NA	NA
chr1	19992231	19992231	T	C	UTR5	HTR6	NA	NA	NA	NA	NA
chr1	19992234	19992234	A	C	UTR5	HTR6	NA	NA	NA	NA	NA
chr1	20208984	20208984	G	T	UTR5	OTUD3	NA	NA	NA	NA	NA
chr1	20237881	20237881	-	T	UTR3	OTUD3	NA	NA	NA	NA	NA
chr1	20617419	20617419	T	C	UTR5	VWA5B1	NA	NA	NA	rs12738826	NA
chr1	20671961	20671961	G	A	exonic	VWA5B1	nonsynonymous SNV	VWA5B1:NM_001039500:exon17:c.G2639A:p.R880H	NA	rs11582960	NA
chr1	20978415	20978415	T	G	UTR3	DDOST	NA	NA	NA	rs150466875	NA
chr1	21766776	21766776	G	C	UTR5	NBPF3	NA	NA	NA	rs113548640	NA
chr1	21808223	21808223	T	C	exonic	NBPF3	synonymous SNV	57C:p.L453L,NBPF3:NM_001256416:exon13:c.T1531C:p.L511L,NBPF3:I	NA	rs145079058	NA
chr1	22160042	22160042	T	G	exonic	HSPG2	synonymous SNV	HSPG2:NM_005529:exon79:c.A10896C:p.R3632R	NA	NA	NA
chr1	22357521	22357526	TTTTTT	-	ncRNA_exonic	LINC00339	NA	NA	NA	NA	NA
chr1	22445719	22445719	G	-	UTR3	WNT4	NA	NA	NA	NA	NA
chr1	22445721	22445721	G	-	UTR3	WNT4	NA	NA	NA	NA	NA
chr1	23695593	23695593	-	G	ncRNA_exonic	C1orf213	NA	NA	NA	NA	NA
chr1	23695637	23695637	T	G	ncRNA_exonic	C1orf213	NA	NA	NA	NA	NA
chr1	23695642	23695642	C	G	ncRNA_exonic	C1orf213	NA	NA	NA	NA	NA
chr1	23695644	23695644	T	G	ncRNA_exonic	C1orf213	NA	NA	NA	NA	NA
chr1	23695669	23695669	T	C	ncRNA_exonic	C1orf213	NA	NA	NA	NA	NA
chr1	23695680	23695680	C	G	ncRNA_exonic	C1orf213	NA	NA	NA	NA	NA
chr1	23695687	23695687	T	C	ncRNA_exonic	C1orf213	NA	NA	NA	NA	NA
chr1	23886300	23886300	C	-	upstream	ID3	NA	NA	NA	rs139377135	NA
chr1	24296853	24296853	-	TT	UTR3	SRSF10	NA	NA	NA	NA	NA
chr1	24798068	24798068	-	A	UTR3	NIPAL3	NA	NA	NA	rs35468932	NA
chr1	24798499	24798500	TT	-	UTR3	NIPAL3	NA	NA	NA	rs148414271	NA
chr1	24999676	24999676	-	T	UTR3	SRRM1	NA	NA	NA	rs113632210	NA
chr1	25747230	25747230	G	C	exonic	RHCE	nonsynonymous SNV	NM_138616:exon1:c.C48G:p.C16W,RHCE:NM_138617:exon1:c.C48G:p	NA	rs201634828	B

chr1	26143704	26143704	-	TTTTG	UTR3	SEPN1	NA	NA	NA	rs3033477	NA
chr1	26211187	26211188	AA	-	UTR3	STMN1	NA	NA	NA	NA	NA
chr1	26487940	26487940	A	G	exonic	FAM110D	nonsynonymous SNV	FAM110D:NM_024869:exon2:c.A158G:p.H53R	NA	rs3748856	D
chr1	26488883	26488883	G	T	UTR3	FAM110D	NA	NA	NA	NA	NA
chr1	26627478	26627478	T	C	exonic	UBXN11	synonymous SNV	/I_001077262:exon4:c.A138G:p.S46S,UBXN11:NM_183008:exon5:c.A1	NA	rs2276712	NA
chr1	26786627	26786627	G	A	exonic	DHDDS	nonsynonymous SNV	1_001243565:exon7:c.G640A:p.V214M,DHDDS:NM_024887:exon8:c.G	=COSM146454;OCCURENCE=1(stomac	rs3816539	B
chr1	26801737	26801737	-	T	UTR3	HMGN2	NA	NA	NA	NA	NA
chr1	27107651	27107651	A	-	UTR3	ARID1A	NA	NA	NA	NA	NA
chr1	27190821	27190821	-	GT	UTR3	SFN	NA	NA	NA	rs3065004	NA
chr1	28475837	28475838	TT	-	UTR3	PTAFR	NA	NA	NA	NA	NA
chr1	31838552	31838552	A	-	UTR3	FABP3	NA	NA	NA	NA	NA
chr1	31907249	31907249	T	C	UTR3	SERINC2	NA	NA	NA	NA	NA
chr1	32673866	32673866	G	C	UTR3	IQCC	NA	NA	NA	rs41302746	NA
chr1	33148362	33148362	-	CT	UTR3	RBBP4	NA	NA	NA	rs5773380	NA
chr1	33148866	33148867	AA	-	UTR3	RBBP4	NA	NA	NA	rs148907756	NA
chr1	33475531	33475531	A	T	UTR3	AK2	NA	NA	NA	NA	NA
chr1	33475556	33475556	A	C	UTR3	AK2	NA	NA	NA	rs201880820	NA
chr1	33475767	33475767	G	A	UTR3	AK2	NA	NA	NA	rs74066436	NA
chr1	33475791	33475791	A	G	UTR3	AK2	NA	NA	NA	rs6884	NA
chr1	33764916	33764916	A	C	UTR3	ZNF362	NA	NA	NA	NA	NA
chr1	35220730	35220730	A	G	UTR5	GJB5	NA	NA	NA	rs2275229	NA
chr1	36032231	36032232	CA	-	UTR3	NCDN	NA	NA	NA	NA	NA
chr1	36770048	36770048	T	-	UTR3	THRAP3	NA	NA	NA	NA	NA
chr1	36770065	36770065	C	T	UTR3	THRAP3	NA	NA	NA	rs75716416	NA
chr1	38226777	38226777	C	A	UTR3	EPHA10	NA	NA	NA	rs116544770	NA
chr1	38327409	38327409	G	A	UTR3	INPP5B	NA	NA	NA	NA	NA
chr1	38423061	38423061	C	T	UTR3	SF3A3	NA	NA	NA	rs28667771	NA
chr1	38490065	38490065	A	T	UTR3	UTP11L	NA	NA	NA	NA	NA
chr1	38510268	38510268	T	-	UTR3	POU3F1	NA	NA	NA	rs5773610	NA
chr1	39340002	39340002	A	T	ncRNA_exonic	GJA9-MYCBP	NA	NA	NA	rs66539868	NA
chr1	39470979	39470979	-	A	UTR3	AKIRIN1	NA	NA	NA	NA	NA
chr1	39796845	39796855	TTTTTTTTT	-	intronic	MACF1	NA	NA	NA	rs201626891	NA
chr1	39835817	39835817	A	G	exonic	MACF1	nonsynonymous SNV	MACF1:NM_012090:exon47:c.A6868G:p.M2290V	NA	rs2296172	B
chr1	40089595	40089595	C	T	UTR3	HEYL	NA	NA	NA	rs12030495	NA
chr1	40235448	40235448	C	T	exonic	OXCT2	nonsynonymous SNV	OXCT2:NM_022120:exon1:c.G1480A:p.D494N	NA	rs150795467	P
chr1	43232997	43232997	C	A	exonic	C1orf50	synonymous SNV	C1orf50:NM_024097:exon1:c.C15A:p.A5A	NA	rs193038877	NA
chr1	43233201	43233201	C	A	exonic	C1orf50	nonsynonymous SNV	C1orf50:NM_024097:exon2:c.C101A:p.P34Q	NA	NA	P
chr1	43272824	43272824	-	TT	UTR3	CCDC23	NA	NA	NA	NA	NA
chr1	43649508	43649508	A	G	exonic	WDR65	nonsynonymous SNV	LG:p.N241D,WDR65:NM_001195831:exon4:c.A721G:p.N241D,WDR65	NA	rs663824	B
chr1	44084739	44084739	C	T	exonic	PTPRF	synonymous SNV	L30440:exon26:c.C4485T:p.R1495R,PTPRF:NM_002840:exon27:c.C451	NA	rs1143701	NA
chr1	45444038	45444038	G	A	exonic	EIF2B3	synonymous SNV	243T:p.D81D,EIF2B3:NM_001261418:exon3:c.C243T:p.D81D,EIF2B3:N	NA	rs11556200	NA
chr1	47099235	47099235	A	C	intergenic	3C(dist=16672),ATPAF1(dist=	NA	NA	NA	rs4660950	NA
chr1	47905783	47905783	T	-	UTR3	FOXD2	NA	NA	NA	NA	NA
chr1	51204557	51204557	G	A	exonic	FAF1	nonsynonymous SNV	FAF1:NM_007051:exon6:c.C529T:p.P177S	NA	rs140027012	B
chr1	51820325	51820325	C	T	UTR3	EPS15	NA	NA	NA	rs11554033	NA
chr1	52520859	52520861	ACA	-	UTR5	TXNDC12	NA	NA	NA	rs142834436	NA
chr1	53289190	53289190	-	T	UTR3	ZYG11B	NA	NA	NA	NA	NA
chr1	53291326	53291327	AA	-	UTR3	ZYG11B	NA	NA	NA	NA	NA
chr1	53537266	53537266	G	A	exonic	PODN	synonymous SNV	:p.T172T,PODN:NM_001199081:exon4:c.G459A:p.T153T,PODN:NM_0	NA	rs1769316	NA
chr1	53662140	53662140	G	T	UTR5	CPT2	NA	NA	NA	NA	NA
chr1	53712557	53712557	-	A	UTR3	LRP8	NA	NA	NA	NA	NA
chr1	53930360	53930360	G	A	exonic	DMRTB1	synonymous SNV	DMRTB1:NM_033067:exon3:c.G801A:p.P267P	NA	rs61738842	NA
chr1	54199823	54199823	G	T	UTR5	GLIS1	NA	NA	NA	NA	NA
chr1	54681920	54681920	G	C	exonic	MRPL37	nonsynonymous SNV	MRPL37:NM_016491:exon6:c.G1097C:p.C366S	NA	rs13571	B
chr1	57179398	57179398	T	-	UTR3	PRKAA2	NA	NA	NA	rs143795464	NA
chr1	58971831	58971831	G	A	exonic	OMA1	synonymous SNV	OMA1:NM_145243:exon8:c.C1266T:p.F422F	NA	rs3087585	NA
chr1	59247323	59247323	T	C	UTR3	JUN	NA	NA	NA	NA	NA
chr1	60280750	60280750	T	-	UTR5	HOOK1	NA	NA	NA	rs113730234	NA
chr1	60466814	60466814	T	C	exonic	C1orf87	nonsynonymous SNV	C1orf87:NM_152377:exon10:c.A1207G:p.K403E	NA	rs626251	B
chr1	61921185	61921185	-	T	UTR3	NFIA	NA	NA	NA	rs111405657	NA
chr1	61924354	61924354	G	T	UTR3	NFIA	NA	NA	NA	NA	NA
chr1	61927697	61927697	T	-	UTR3	NFIA	NA	NA	NA	NA	NA
chr1	61927742	61927742	C	T	UTR3	NFIA	NA	NA	NA	NA	NA
chr1	62627004	62627007	TTTT	-	UTR3	INADL	NA	NA	NA	NA	NA
chr1	62676612	62676612	A	G	exonic	L1TD1	synonymous SNV	019079:exon4:c.A2166G:p.K722K,L1TD1:NM_001164835:exon5:c.A21	SM118430;OCCURENCE=1(ovary),1(sto	rs66958136	NA
chr1	62960101	62960101	A	G	exonic	DOCK7	synonymous SNV	01272001:exon39:c.T4942C:p.L1648L,DOCK7:NM_033407:exon39:c.T4	NA	rs10889335	NA

chr1	64036764	64036764	A	G	exonic	EFCAB7	nonsynonymous SNV	EFCAB7:NM_032437:exon13:c.A1780G:p.I594V	NA	rs199608136	B
chr1	64644708	64644708	A	-	UTR3	ROR1	NA	NA	NA	rs10709706	NA
chr1	66831370	66831370	G	A	exonic	PDE4B	synonymous SNV	1037340:exon11:c.G1260A:p.E420E,PDE4B:NM_001037341:exon13:c.	NA	rs783036	NA
chr1	67288175	67288175	C	A	exonic	WDR78	nonsynonymous SNV	WDR78:NM_024763:exon16:c.G2365T:p.A789S	NA	NA	B
chr1	67303303	67303303	A	G	UTR3	WDR78	NA	NA	NA	rs6698076	NA
chr1	67557966	67557966	G	T	UTR3	C1orf141	NA	NA	NA	rs6672194	NA
chr1	72748272	72748272	A	C	UTR5	NEGR1	NA	NA	NA	NA	NA
chr1	76190216	76190216	G	A	UTR5	ACADM	NA	NA	NA	rs17848068	NA
chr1	78343510	78343510	C	T	UTR3	FAM73A	NA	NA	NA	rs55760347	NA
chr1	78602292	78602292	C	T	UTR3	GIPC2	NA	NA	NA	NA	NA
chr1	78602292	78602292	-	TT	UTR3	GIPC2	NA	NA	NA	NA	NA
chr1	79108070	79108070	A	-	UTR3	IFI44L	NA	NA	NA	NA	NA
chr1	85462582	85462582	C	G	UTR5	MCOLN2	NA	NA	NA	rs11161503	NA
chr1	85462652	85462652	G	T	UTR5	MCOLN2	NA	NA	NA	rs11161504	NA
chr1	85462665	85462665	T	G	UTR5	MCOLN2	NA	NA	NA	rs11161505	NA
chr1	85743584	85743584	C	-	ncRNA_exonic	LOC646626	NA	NA	NA	rs201121966	NA
chr1	86119296	86119296	C	A	UTR3	ZNHIT6	NA	NA	NA	rs821399	NA
chr1	89225976	89225976	T	C	exonic	PKN2	synonymous SNV	PKN2:NM_006256:exon3:c.T421C:p.L141L	NA	rs430600	NA
chr1	89449616	89449616	G	C	UTR5	RBMXL1	NA	NA	NA	NA	NA
chr1	89726090	89726090	-	G	UTR3	GBP5	NA	NA	NA	rs140412648	NA
chr1	93620393	93620393	G	A	exonic	TMED5	nonsynonymous SNV	TMED5:NM_016040:exon4:c.C524T:p.T175I	NA	rs1060622	D
chr1	93811569	93811569	A	G	UTR5	DR1	NA	NA	NA	rs10782959	NA
chr1	94353029	94353029	-	T	UTR3	GCLM	NA	NA	NA	rs146547212	NA
chr1	94995630	94995632	AAA	-	UTR3	F3	NA	NA	NA	rs58927022	NA
chr1	95362928	95362929	AA	-	UTR3	CNN3	NA	NA	NA	rs58217295	NA
chr1	95363001	95363001	T	C	UTR3	CNN3	NA	NA	NA	rs3789699	NA
chr1	95657855	95657855	A	-	UTR3	TMEM56	NA	NA	NA	NA	NA
chr1	97279218	97279223	TTTTTT	-	UTR3	PTBP2	NA	NA	NA	NA	NA
chr1	97770920	97770920	C	T	exonic	DPYD	nonsynonymous SNV	DPYD:NM_000110:exon18:c.G2194A:p.V732I	NA	rs1801160	D
chr1	1.01E+08	1.01E+08	A	-	UTR3	SASS6	NA	NA	NA	NA	NA
chr1	1.01E+08	1.01E+08	A	-	UTR3	DBT	NA	NA	NA	rs71709231	NA
chr1	1.01E+08	1.01E+08	ACACACAC	-	UTR3	DBT	NA	NA	NA	rs140341302	NA
chr1	1.03E+08	1.03E+08	G	A	exonic	COL11A1	nonsynonymous SNV	)1190709:exon51:c.C3851T:p.P1284L,COL11A1:NM_001854:exon52:c.	NA	rs3753841	NA
chr1	1.04E+08	1.04E+08	C	G	exonic	AMY2A	synonymous SNV	AMY2A:NM_000699:exon5:c.C834G:p.G278G	NA	rs201666814	NA
chr1	1.04E+08	1.04E+08	G	A	exonic	AMY2A	nonsynonymous SNV	AMY2A:NM_000699:exon5:c.G838A:p.V280I	NA	rs141685988	B
chr1	1.09E+08	1.09E+08	G	A	exonic	NBPF4	synonymous SNV	NBPF4:NM_001143989:exon14:c.C1869T:p.S623S	COSM893345;OCCURENCE=2(endometr	rs2582071	NA
chr1	1.09E+08	1.09E+08	C	T	UTR3	FAM102B	NA	NA	NA	NA	NA
chr1	1.09E+08	1.09E+08	G	T	UTR3	FAM102B	NA	NA	NA	NA	NA
chr1	1.1E+08	1.1E+08	T	C	UTR5	C1orf194	NA	NA	NA	rs536176	NA
chr1	1.1E+08	1.1E+08	AAA	-	UTR3	KIAA1324	NA	NA	NA	rs149143656	NA
chr1	1.1E+08	1.1E+08	A	C	exonic	AMIGO1	synonymous SNV	AMIGO1:NM_020703:exon2:c.T1356G:p.G452G	NA	NA	NA
chr1	1.1E+08	1.1E+08	C	T	UTR5	GNAI3	NA	NA	NA	rs3737182	NA
chr1	1.1E+08	1.1E+08	G	A	exonic	AMPD2	nonsynonymous SNV	1_004037:exon1:c.G244A:p.A82T,AMPD2:NM_001257360:exon2:c.G2	NA	rs28362581	B
chr1	1.11E+08	1.11E+08	-	C	UTR5	AHCYL1	NA	NA	NA	rs11424431	NA
chr1	1.11E+08	1.11E+08	G	A	UTR5	AHCYL1	NA	NA	NA	rs17551493	NA
chr1	1.11E+08	1.11E+08	A	-	UTR3	AHCYL1	NA	NA	NA	NA	NA
chr1	1.11E+08	1.11E+08	A	C	exonic	SLC6A17	nonsynonymous SNV	SLC6A17:NM_001010898:exon12:c.A2164C:p.T722P	NA	NA	NA
chr1	1.11E+08	1.11E+08	A	G	UTR3	SLC6A17	NA	NA	NA	rs12133992	NA
chr1	1.11E+08	1.11E+08	C	A	UTR3	SLC6A17	NA	NA	NA	NA	NA
chr1	1.11E+08	1.11E+08	G	A	UTR3	SLC16A4	NA	NA	NA	rs185712869	NA
chr1	1.11E+08	1.11E+08	-	CA	UTR3	LRIF1	NA	NA	NA	NA	NA
chr1	1.12E+08	1.12E+08	C	G	exonic	OVGP1	nonsynonymous SNV	OVGP1:NM_002557:exon11:c.G1561C:p.E521Q	NA	rs201662631	B
chr1	1.12E+08	1.12E+08	C	T	exonic	OVGP1	synonymous SNV	OVGP1:NM_002557:exon11:c.G1560A:p.G520G	NA	NA	NA
chr1	1.12E+08	1.12E+08	G	C	exonic	OVGP1	nonsynonymous SNV	OVGP1:NM_002557:exon11:c.C1553G:p.T518S	NA	rs45455292	B
chr1	1.12E+08	1.12E+08	A	G	exonic	OVGP1	nonsynonymous SNV	OVGP1:NM_002557:exon11:c.T1540C:p.Y514H	NA	rs1126656	B
chr1	1.12E+08	1.12E+08	A	G	exonic	OVGP1	nonsynonymous SNV	OVGP1:NM_002557:exon11:c.T1531C:p.S511P	NA	rs56294468	B
chr1	1.13E+08	1.13E+08	T	G	exonic	ST7L	synonymous SNV	M_138727:exon7:c.A672C:p.L224L,ST7L:NM_138728:exon7:c.A723C:p	NA	NA	NA
chr1	1.13E+08	1.13E+08	T	-	UTR3	CAPZA1	NA	NA	NA	NA	NA
chr1	1.15E+08	1.15E+08	T	C	exonic	BCAS2	nonsynonymous SNV	BCAS2:NM_005872:exon1:c.A89G:p.E30G	NA	NA	D
chr1	1.15E+08	1.15E+08	C	A	exonic	BCAS2	nonsynonymous SNV	BCAS2:NM_005872:exon1:c.G58T:p.D20Y	NA	NA	D
chr1	1.15E+08	1.15E+08	T	C	exonic	BCAS2	synonymous SNV	BCAS2:NM_005872:exon1:c.A27G:p.G9G	NA	NA	NA
chr1	1.15E+08	1.15E+08	G	A	UTR5	CSDE1	NA	NA	NA	rs41274132	NA
chr1	1.15E+08	1.15E+08	T	C	exonic	SIKE1	nonsynonymous SNV	1_001102396:exon1:c.A142G:p.T48A,SIKE1:NM_025073:exon1:c.A142	NA	NA	B
chr1	1.16E+08	1.16E+08	-	TA	UTR3	TSPAN2	NA	NA	NA	rs34617679	NA
chr1	1.17E+08	1.17E+08	C	G	exonic	IGSF3	nonsynonymous SNV	001007237:exon5:c.G1050C:p.K350N,IGSF3:NM_001542:exon5:c.G105	NA	rs140699309	B
chr1	1.17E+08	1.17E+08	C	T	exonic	IGSF3	nonsynonymous SNV	M_001007237:exon3:c.G214A:p.V72I,IGSF3:NM_001542:exon3:c.G214	NA	rs201676764	B

chr1	1.17E+08	1.17E+08	C	T	exonic	IGSF3	synonymous SNV	M_001007237:exon3:c.G189A:p.S63S,IGSF3:NM_001542:exon3:c.G189	NA	rs200177381	NA
chr1	1.17E+08	1.17E+08	C	T	exonic	IGSF3	synonymous SNV	M_001007237:exon3:c.G99A:p.T33T,IGSF3:NM_001542:exon3:c.G99A	8218,COSM288219;OCCURENCE=3(larg	rs201692914	NA
chr1	1.18E+08	1.18E+08	GGCGGCGC	-	UTR5	MAN1A2	NA	NA	NA	NA	NA
chr1	1.18E+08	1.18E+08	ATTT	-	UTR3	FAM46C	NA	NA	NA	rs34914954	NA
chr1	1.19E+08	1.19E+08	-	A	UTR3	TBX15	NA	NA	NA	NA	NA
chr1	1.21E+08	1.21E+08	T	A	exonic	NOTCH2	nonsynonymous SNV	_001200001:exon4:c.A703T:p.T235S,NOTCH2:NM_024408:exon4:c.A7	NA	rs200464440	D
chr1	1.21E+08	1.21E+08	G	T	exonic	NOTCH2	nonsynonymous SNV	_001200001:exon4:c.C680A:p.P227H,NOTCH2:NM_024408:exon4:c.C6	NA	rs3899528	D
chr1	1.21E+08	1.21E+08	C	G	UTR5	NOTCH2	NA	NA	NA	rs79247096	NA
chr1	1.21E+08	1.21E+08	C	T	exonic	FAM72B	nonsynonymous SNV	FAM72B:NM_001100910:exon3:c.C281T:p.P94L	NA	rs1572701	B
chr1	1.45E+08	1.45E+08	A	G	UTR5	NBPF8,NBPF9	NA	NA	NA	rs201543878	NA
chr1	1.45E+08	1.45E+08	T	C	UTR5	NBPF8,NBPF9	NA	NA	NA	rs199745076	NA
chr1	1.45E+08	1.45E+08	G	A	exonic	NBPF8,NBPF9	unknown	UNKNOWN	NA	rs200319336	NA
chr1	1.45E+08	1.45E+08	G	T	exonic	NBPF8,NBPF9	unknown	UNKNOWN	191623;OCCURENCE=1(central_nervou	NA	NA
chr1	1.45E+08	1.45E+08	A	T	UTR3	PDE4DIP	NA	NA	NA	rs141695068	NA
chr1	1.45E+08	1.45E+08	T	C	UTR3	SEC22B	NA	NA	NA	rs7550440	NA
chr1	1.45E+08	1.45E+08	G	C	exonic	NBPF10	nonsynonymous SNV	NBPF10:NM_001039703:exon1:c.G85C:p.A29P	NA	rs6671335	NA
chr1	1.45E+08	1.45E+08	G	C	exonic	NBPF10	nonsynonymous SNV	NBPF10:NM_001039703:exon1:c.G93C:p.K31N	NA	rs4409714	NA
chr1	1.48E+08	1.48E+08	T	C	UTR3	NBPF14	NA	NA	NA	rs16826730	NA
chr1	1.48E+08	1.48E+08	G	A	UTR3	NBPF14	NA	NA	NA	rs3961381	NA
chr1	1.48E+08	1.48E+08	T	C	exonic	NBPF14	synonymous SNV	NBPF14:NM_015383:exon22:c.A2766G:p.X922X	NA	rs79370452	NA
chr1	1.48E+08	1.48E+08	T	C	exonic	NBPF14	synonymous SNV	NBPF14:NM_015383:exon5:c.A603G:p.G201G	NA	NA	NA
chr1	1.49E+08	1.49E+08	T	C	exonic	PPIAL4E	synonymous SNV	PPIAL4E:NM_001144032:exon1:c.T345C:p.C115C	NA	rs372485	NA
chr1	1.49E+08	1.49E+08	T	C	UTR3	NBPF16	NA	NA	NA	rs78259445	NA
chr1	1.49E+08	1.49E+08	G	A	ncRNA_exonic	LOC388692	NA	NA	NA	rs3926814	NA
chr1	1.49E+08	1.49E+08	A	G	ncRNA_exonic	LOC388692	NA	NA	NA	rs139360823	NA
chr1	1.49E+08	1.49E+08	T	C	ncRNA_exonic	LOC388692	NA	NA	NA	rs149624901	NA
chr1	1.49E+08	1.49E+08	T	C	ncRNA_exonic	LOC388692	NA	NA	NA	rs593054	NA
chr1	1.49E+08	1.49E+08	-	A	ncRNA_exonic	LOC388692	NA	NA	NA	NA	NA
chr1	1.5E+08	1.5E+08	-	CCG	UTR5	RPRD2	NA	NA	NA	NA	NA
chr1	1.5E+08	1.5E+08	A	-	UTR3	RPRD2	NA	NA	NA	NA	NA
chr1	1.51E+08	1.51E+08	G	A	exonic	CTSS	nonsynonymous SNV	CTSS:NM_004079:exon4:c.C337T:p.R113W	)=COSM146600;OCCURENCE=1(stomac	rs2230061	B
chr1	1.52E+08	1.52E+08	A	G	UTR3	RIIAD1	NA	NA	NA	rs41263712	NA
chr1	1.52E+08	1.52E+08	T	C	UTR3	THEM4	NA	NA	NA	rs74997792	NA
chr1	1.52E+08	1.52E+08	C	G	exonic	TCHH	nonsynonymous SNV	TCHH:NM_007113:exon3:c.G3436C:p.V1146L	NA	NA	NA
chr1	1.52E+08	1.52E+08	T	C	exonic	TCHH	nonsynonymous SNV	TCHH:NM_007113:exon3:c.A3244G:p.K1082E	COSM895998;OCCURENCE=1(endometr	rs199978971	NA
chr1	1.52E+08	1.52E+08	A	C	exonic	TCHH	nonsynonymous SNV	TCHH:NM_007113:exon3:c.T3128G:p.L1043R	ID=COSM528874;OCCURENCE=1(lung)	NA	NA
chr1	1.52E+08	1.52E+08	A	G	exonic	HRNR	synonymous SNV	HRNR:NM_001009931:exon3:c.T6243C:p.Y2081Y	NA	rs143217581	NA
chr1	1.52E+08	1.52E+08	G	A	exonic	HRNR	nonsynonymous SNV	HRNR:NM_001009931:exon3:c.C5642T:p.S1881F	NA	rs61814941	NA
chr1	1.52E+08	1.52E+08	C	T	exonic	CRCT1	synonymous SNV	CRCT1:NM_019060:exon2:c.C120T:p.C40C	NA	rs2282296	NA
chr1	1.52E+08	1.52E+08	C	T	exonic	CRCT1	synonymous SNV	CRCT1:NM_019060:exon2:c.C204T:p.R68R	NA	rs1053588	NA
chr1	1.53E+08	1.53E+08	A	C	exonic	KPRP	nonsynonymous SNV	KPRP:NM_001025231:exon2:c.A886C:p.T296P	NA	NA	D
chr1	1.53E+08	1.53E+08	G	T	UTR3	LCE1E	NA	NA	NA	rs114470093	NA
chr1	1.53E+08	1.53E+08	T	C	exonic	IVL	synonymous SNV	IVL:NM_005547:exon2:c.T975C:p.H325H	NA	NA	NA
chr1	1.53E+08	1.53E+08	C	T	exonic	SPRR2E	nonsynonymous SNV	SPRR2E:NM_001024209:exon2:c.G161A:p.C54Y	=COSM225968;OCCURENCE=1(NS),1(lu	rs75229137	NA
chr1	1.53E+08	1.53E+08	T	C	exonic	SPRR2E	synonymous SNV	SPRR2E:NM_001024209:exon2:c.A156G:p.Q52Q	NA	rs3737865	NA
chr1	1.54E+08	1.54E+08	G	A	UTR5	S100A13	NA	NA	NA	rs3818632	NA
chr1	1.54E+08	1.54E+08	A	G	UTR3	CHTOP	NA	NA	NA	NA	NA
chr1	1.54E+08	1.54E+08	G	C	exonic	SLC27A3	nonsynonymous SNV	SLC27A3:NM_024330:exon1:c.G329C:p.G110A	NA	rs34527123	B
chr1	1.54E+08	1.54E+08	AA	-	UTR3	TPM3	NA	NA	NA	NA	NA
chr1	1.54E+08	1.54E+08	G	A	UTR5	IL6R	NA	NA	NA	rs4845617	NA
chr1	1.55E+08	1.55E+08	C	T	exonic	ADAR	nonsynonymous SNV	4A:p.E982K,ADAR:NM_001193495:exon11:c.G2059A:p.E687K,ADAR:N	NA	NA	P
chr1	1.55E+08	1.55E+08	A	C	exonic	TRIM46	nonsynonymous SNV	M_001256601:exon2:c.A208C:p.T70P,TRIM46:NM_025058:exon2:c.A2	NA	NA	P
chr1	1.56E+08	1.56E+08	G	A	ncRNA_exonic	MSTO2P	NA	NA	NA	rs66501532	NA
chr1	1.56E+08	1.56E+08	T	-	UTR3	MEX3A	NA	NA	NA	rs201492514	NA
chr1	1.56E+08	1.56E+08	A	-	UTR3	LMNA	NA	NA	NA	rs200317083	NA
chr1	1.56E+08	1.56E+08	GA	-	UTR5	CCT3	NA	NA	NA	rs139949895	NA
chr1	1.58E+08	1.58E+08	T	-	UTR3	CD1E	NA	NA	NA	NA	NA
chr1	1.59E+08	1.59E+08	A	G	UTR5	IFI16	NA	NA	NA	rs2276404	NA
chr1	1.59E+08	1.59E+08	G	A	UTR3	FCER1A	NA	NA	NA	rs7549785	NA
chr1	1.6E+08	1.6E+08	AC	-	UTR3	KCNJ10	NA	NA	NA	NA	NA
chr1	1.6E+08	1.6E+08	A	G	exonic	ATP1A4	synonymous SNV	ATP1A4:NM_144699:exon1:c.A69G:p.K23K	NA	rs77332724	NA
chr1	1.6E+08	1.6E+08	T	-	UTR3	PEX19	NA	NA	NA	NA	NA
chr1	1.61E+08	1.61E+08	C	G	exonic	CD48	nonsynonymous SNV	_001256030:exon2:c.G304C:p.E102Q,CD48:NM_001778:exon2:c.G304	)=COSM146667;OCCURENCE=1(stomac	rs2295615	D
chr1	1.61E+08	1.61E+08	G	A	exonic	CD48	synonymous SNV	M_001256030:exon2:c.C276T:p.G92G,CD48:NM_001778:exon2:c.C276	NA	rs1980606	NA
chr1	1.61E+08	1.61E+08	A	C	UTR5	F11R	NA	NA	NA	NA	NA
chr1	1.61E+08	1.61E+08	-	A	UTR3	USF1	NA	NA	NA	NA	NA

chr1	1.61E+08	1.61E+08	A	-	UTR3	DEDD	NA	NA	NA	NA	NA
chr1	1.62E+08	1.62E+08	A	G	UTR3	FCGR2C	NA	NA	NA	rs144426992	NA
chr1	1.62E+08	1.62E+08	T	C	UTR3	FCGR2C	NA	NA	NA	rs199512091	NA
chr1	1.62E+08	1.62E+08	C	G	UTR5	SH2D1B	NA	NA	NA	rs66518016	NA
chr1	1.63E+08	1.63E+08	C	T	UTR3	DDR2	NA	NA	NA	rs1972086	NA
chr1	1.63E+08	1.63E+08	ACACAC	-	UTR3	RGS4	NA	NA	NA	NA	NA
chr1	1.63E+08	1.63E+08	T	-	UTR3	RGS5	NA	NA	NA	NA	NA
chr1	1.67E+08	1.67E+08	-	A	UTR3	POGK	NA	NA	NA	NA	NA
chr1	1.68E+08	1.68E+08	TAAT	-	UTR3	RCSD1	NA	NA	NA	NA	NA
chr1	1.68E+08	1.68E+08	A	G	exonic	ADCY10	synonymous SNV	)1167749:exon16:c.T1938C:p.G646G,ADCY10:NM_018417:exon19:c.T	NA	rs203795	NA
chr1	1.68E+08	1.68E+08	-	ACACAC	UTR3	TIPRL	NA	NA	NA	NA	NA
chr1	1.7E+08	1.7E+08	C	T	exonic	SELP	synonymous SNV	SELP:NM_003005:exon14:c.G2346A:p.T782T	NA	rs6128	NA
chr1	1.7E+08	1.7E+08	C	T	exonic	KIFAP3	synonymous SNV	)1204517:exon14:c.G1398A:p.G466G,KIFAP3:NM_014970:exon14:c.G1	NA	rs33943686	NA
chr1	1.7E+08	1.7E+08	A	G	ncRNA_exonic	LOC284688	NA	NA	NA	rs3795614	NA
chr1	1.7E+08	1.7E+08	T	C	ncRNA_exonic	LOC284688	NA	NA	NA	rs3795615	NA
chr1	1.71E+08	1.71E+08	G	T	ncRNA_exonic	FMO6P	NA	NA	NA	rs11812061	NA
chr1	1.71E+08	1.71E+08	G	A	ncRNA_exonic	FMO6P	NA	NA	NA	rs7886938	NA
chr1	1.72E+08	1.72E+08	-	TG	UTR3	DNM3	NA	NA	NA	rs143989240	NA
chr1	1.75E+08	1.75E+08	T	G	exonic	TNN	nonsynonymous SNV	TNN:NM_022093:exon19:c.T3764G:p.V1255G	NA	NA	D
chr1	1.77E+08	1.77E+08	C	T	exonic	PAPPA2	synonymous SNV	PAPPA2:NM_020318:exon22:c.C5262T:p.D1754D	NA	rs12118034	NA
chr1	1.78E+08	1.78E+08	G	A	exonic	SEC16B	synonymous SNV	SEC16B:NM_033127:exon8:c.C954T:p.S318S	NA	rs35891059	NA
chr1	1.79E+08	1.79E+08	C	T	UTR3	ABL2	NA	NA	NA	NA	NA
chr1	1.8E+08	1.8E+08	A	G	exonic	TDRD5	nonsynonymous SNV	001199089:exon7:c.A1072G:p.K358E,TDRD5:NM_001199091:exon7:c.	NA	rs6704505	B
chr1	1.8E+08	1.8E+08	CGCGCG	-	UTR3	FAM163A	NA	NA	NA	rs71744420	NA
chr1	1.81E+08	1.81E+08	C	T	exonic	KIAA1614	synonymous SNV	KIAA1614:NM_020950:exon4:c.C1065T:p.T355T	NA	rs3747958	NA
chr1	1.82E+08	1.82E+08	G	A	exonic	CACNA1E	nonsynonymous SNV	).A1936T,CACNA1E:NM_000721:exon44:c.G5863A:p.A1955T,CACNA1I	NA	rs704326	NA
chr1	1.83E+08	1.83E+08	C	T	exonic	LAMC1	synonymous SNV	LAMC1:NM_002293:exon2:c.C657T:p.N219N	NA	rs2296289	NA
chr1	1.83E+08	1.83E+08	A	G	UTR5	LAMC2	NA	NA	NA	rs2276542	NA
chr1	1.83E+08	1.83E+08	A	C	UTR5	LAMC2	NA	NA	NA	NA	NA
chr1	1.83E+08	1.83E+08	-	GT	UTR3	NMNAT2	NA	NA	NA	NA	NA
chr1	1.85E+08	1.85E+08	T	A	UTR3	FAM129A	NA	NA	NA	rs526024	NA
chr1	1.85E+08	1.85E+08	T	G	UTR5	TRMT1L	NA	NA	NA	NA	NA
chr1	1.86E+08	1.86E+08	T	C	exonic	PRG4	synonymous SNV	_001127709:exon5:c.T945C:p.T315T,PRG4:NM_001127708:exon6:c.T1	D=COSM463577;OCCURENCE=1(kidney	NA	NA
chr1	1.86E+08	1.86E+08	T	C	exonic	PRG4	nonsynonymous SNV	001127709:exon5:c.T1000C:p.S334P,PRG4:NM_001127708:exon6:c.T1	M228824;OCCURENCE=1(skin),2(endom	NA	NA
chr1	1.86E+08	1.86E+08	T	C	exonic	PRG4	nonsynonymous SNV	_001127709:exon5:c.T1351C:p.S451P,PRG4:NM_001127708:exon6:c.T	NA	NA	NA
chr1	1.92E+08	1.92E+08	-	.AAATTTTG	UTR3	RGS21	NA	NA	NA	rs140108972	NA
chr1	1.93E+08	1.93E+08	-	G	UTR5	CDC73	NA	NA	NA	NA	NA
chr1	1.97E+08	1.97E+08	T	G	UTR3	CFHR5	NA	NA	NA	rs10922153	NA
chr1	2.01E+08	2.01E+08	T	G	UTR3	KIF21B	NA	NA	NA	NA	NA
chr1	2.01E+08	2.01E+08	-	A	UTR3	TMEM9	NA	NA	NA	NA	NA
chr1	2.01E+08	2.01E+08	A	G	exonic	IGFN1	nonsynonymous SNV	IGFN1:NM_001164586:exon12:c.A5416G:p.S1806G	NA	NA	NA
chr1	2.01E+08	2.01E+08	T	A	exonic	IGFN1	nonsynonymous SNV	IGFN1:NM_001164586:exon12:c.T5423A:p.V1808E	NA	NA	NA
chr1	2.01E+08	2.01E+08	G	C	exonic	IGFN1	nonsynonymous SNV	IGFN1:NM_001164586:exon12:c.G5431C:p.G1811R	NA	NA	NA
chr1	2.01E+08	2.01E+08	A	G	exonic	IGFN1	nonsynonymous SNV	IGFN1:NM_001164586:exon12:c.A5963G:p.E1988G	NA	NA	NA
chr1	2.02E+08	2.02E+08	A	G	UTR3	IPO9	NA	NA	NA	rs2644134	NA
chr1	2.02E+08	2.02E+08	AAAC	-	UTR3	IPO9	NA	NA	NA	NA	NA
chr1	2.02E+08	2.02E+08	A	G	UTR3	LMOD1	NA	NA	NA	rs8028	NA
chr1	2.02E+08	2.02E+08	AA	-	UTR3	ELF3	NA	NA	NA	rs77731786	NA
chr1	2.02E+08	2.02E+08	A	C	ncRNA_exonic	PTPRVP	NA	NA	NA	rs7550457	NA
chr1	2.02E+08	2.02E+08	A	G	ncRNA_exonic	PTPRVP	NA	NA	NA	rs7550551	NA
chr1	2.02E+08	2.02E+08	;TGCCACCT	-	ncRNA_exonic	PTPRVP	NA	NA	NA	rs72139344	NA
chr1	2.03E+08	2.03E+08	-	AC	UTR3	SYT2	NA	NA	NA	NA	NA
chr1	2.03E+08	2.03E+08	AAA	-	UTR3	MYOG	NA	NA	NA	rs141029773	NA
chr1	2.04E+08	2.04E+08	T	C	exonic	ATP2B4	synonymous SNV	1_001001396:exon3:c.T318C:p.L106L,ATP2B4:NM_001684:exon3:c.T3	NA	rs2228445	NA
chr1	2.04E+08	2.04E+08	A	G	UTR3	PLEKHA6	NA	NA	NA	rs74141005	NA
chr1	2.05E+08	2.05E+08	T	-	UTR3	MDM4	NA	NA	NA	NA	NA
chr1	2.05E+08	2.05E+08	-	T	UTR3	RBBP5	NA	NA	NA	NA	NA
chr1	2.05E+08	2.05E+08	G	A	UTR5	NUAK2	NA	NA	NA	rs3738746	NA
chr1	2.07E+08	2.07E+08	T	C	exonic	AP2,SRGAP2B,SRGAP2C,SRGA	synonymous SNV	SRGAP2D:NM_001271887:exon6:c.T759C:p.F253F	NA	rs200703951	NA
chr1	2.07E+08	2.07E+08	C	T	UTR3	C1orf116	NA	NA	NA	rs10877	NA
chr1	2.07E+08	2.07E+08	C	T	UTR3	PFKFB2	NA	NA	NA	NA	NA
chr1	2.07E+08	2.07E+08	-	T	UTR3	PFKFB2	NA	NA	NA	NA	NA
chr1	2.07E+08	2.07E+08	-	ACAGACTTT	UTR3	PFKFB2	NA	NA	NA	NA	NA
chr1	2.08E+08	2.08E+08	-	T	UTR3	PLXNA2	NA	NA	NA	NA	NA
chr1	2.1E+08	2.1E+08	T	-	UTR3	DIEXF	NA	NA	NA	NA	NA
chr1	2.1E+08	2.1E+08	C	A	UTR3	SERTAD4	NA	NA	NA	NA	NA

chr1	2.11E+08	2.11E+08	A	-	UTR3	KCNH1	NA	NA	NA	rs56001969	NA
chr1	2.12E+08	2.12E+08	GT	-	UTR5	RD3	NA	NA	NA	rs34485370	NA
chr1	2.12E+08	2.12E+08	T	G	UTR3	DTL	NA	NA	NA	rs1387815	NA
chr1	2.13E+08	2.13E+08	-	A	UTR3	NSL1	NA	NA	NA	NA	NA
chr1	2.13E+08	2.13E+08	T	G	UTR3	NSL1	NA	NA	NA	NA	NA
chr1	2.13E+08	2.13E+08	A	G	UTR3	NSL1	NA	NA	NA	rs11800642	NA
chr1	2.13E+08	2.13E+08	T	-	UTR3	FLVCR1	NA	NA	NA	NA	NA
chr1	2.15E+08	2.15E+08	G	A	exonic	CENPF	nonsynonymous SNV	CENPF:NM_016343:exon16:c.G8186A:p.R2729Q	NA	rs335524	NA
chr1	2.19E+08	2.19E+08	CA	-	UTR5	TGFB2	NA	NA	NA	rs151329324	NA
chr1	2.2E+08	2.2E+08	T	-	UTR3	RAB3GAP2	NA	NA	NA	rs35147354	NA
chr1	2.21E+08	2.21E+08	T	C	UTR5	2-Mar	NA	NA	NA	NA	NA
chr1	2.21E+08	2.21E+08	C	T	UTR5	2-Mar	NA	NA	NA	NA	NA
chr1	2.21E+08	2.21E+08	A	T	UTR5	2-Mar	NA	NA	NA	NA	NA
chr1	2.21E+08	2.21E+08	A	T	UTR5	2-Mar	NA	NA	NA	NA	NA
chr1	2.21E+08	2.21E+08	A	G	UTR5	HLX	NA	NA	NA	rs2738752	NA
chr1	2.22E+08	2.22E+08	T	C	ncRNA_exonic	C1orf140	NA	NA	NA	rs4347216	NA
chr1	2.23E+08	2.23E+08	G	A	exonic	HHIPL2	synonymous SNV	HHIPL2:NM_024746:exon8:c.C1845T:p.P615P	NA	rs199572964	NA
chr1	2.23E+08	2.23E+08	G	C	exonic	TAF1A	synonymous SNV	).G267G,TAF1A:NM_001201536:exon10:c.C1143G:p.G381G,TAF1A:NM	NA	rs1134898	NA
chr1	2.24E+08	2.24E+08	C	T	UTR3	DEGS1	NA	NA	NA	rs908803	NA
chr1	2.25E+08	2.25E+08	A	G	exonic	DNAH14	nonsynonymous SNV	DNAH14:NM_001373:exon37:c.A5771G:p.K1924R	NA	rs191528375	NA
chr1	2.26E+08	2.26E+08	A	G	exonic	EPHX1	nonsynonymous SNV	_000120:exon4:c.A416G:p.H139R,EPHX1:NM_001136018:exon4:c.A41	NA	rs2234922	B
chr1	2.26E+08	2.26E+08	C	A	exonic	LEFTY2	nonsynonymous SNV	_003240:exon2:c.G439T:p.V147F,LEFTY2:NM_001172425:exon3:c.G33	NA	NA	P
chr1	2.26E+08	2.26E+08	G	A	UTR5	LIN9	NA	NA	NA	NA	NA
chr1	2.26E+08	2.26E+08	G	T	UTR5	LIN9	NA	NA	NA	NA	NA
chr1	2.26E+08	2.26E+08	G	A	UTR5	LIN9	NA	NA	NA	NA	NA
chr1	2.27E+08	2.27E+08	A	G	UTR3	C1orf95	NA	NA	NA	rs114523191	NA
chr1	2.28E+08	2.28E+08	AAAA	-	UTR5	CDC42BPA	NA	NA	NA	rs199516755	NA
chr1	2.28E+08	2.28E+08	G	A	exonic	OBSCN	nonsynonymous SNV	).V2720M,OBSCN:NM_052843:exon30:c.G8158A:p.V2720M,OBSCN:NI	NA	rs1188697	NA
chr1	2.29E+08	2.29E+08	C	T	exonic	OBSCN	nonsynonymous SNV	).R4516W,OBSCN:NM_052843:exon51:c.C13546T:p.R4516W,OBSCN:N	NA	rs11810627	NA
chr1	2.3E+08	2.3E+08	G	A	UTR3	TAF5L	NA	NA	NA	rs3820359	NA
chr1	2.3E+08	2.3E+08	C	T	intronic	PGBD5	NA	NA	NA	rs2009265	NA
chr1	2.3E+08	2.3E+08	A	C	intronic	PGBD5	NA	NA	NA	rs74143198	NA
chr1	2.31E+08	2.31E+08	T	G	UTR3	C1orf198	NA	NA	NA	NA	NA
chr1	2.31E+08	2.31E+08	A	G	exonic	ARV1	nonsynonymous SNV	ARV1:NM_022786:exon1:c.A37G:p.K13E	NA	NA	P
chr1	2.31E+08	2.31E+08	C	T	UTR3	FAM89A	NA	NA	NA	NA	NA
chr1	2.31E+08	2.31E+08	A	T	UTR3	FAM89A	NA	NA	NA	NA	NA
chr1	2.31E+08	2.31E+08	C	A	UTR3	FAM89A	NA	NA	NA	NA	NA
chr1	2.32E+08	2.32E+08	-	TTAC	ncRNA_UTR3	DISC1	NA	NA	NA	NA	NA
chr1	2.33E+08	2.33E+08	G	A	exonic	SIPA1L2	nonsynonymous SNV	SIPA1L2:NM_020808:exon14:c.C4208T:p.S1403L	NA	rs1547742	P
chr1	2.33E+08	2.33E+08	C	T	exonic	SIPA1L2	synonymous SNV	SIPA1L2:NM_020808:exon1:c.G219A:p.K73K	NA	rs4649383	NA
chr1	2.34E+08	2.34E+08	A	C	UTR5	SLC35F3	NA	NA	NA	rs12136133	NA
chr1	2.34E+08	2.34E+08	G	C	exonic	SLC35F3	synonymous SNV	SLC35F3:NM_173508:exon2:c.G201C:p.P67P	NA	rs57010808	NA
chr1	2.35E+08	2.35E+08	T	C	exonic	TARBP1	nonsynonymous SNV	TARBP1:NM_005646:exon12:c.A2032G:p.S678G	NA	rs4920246	B
chr1	2.35E+08	2.35E+08	T	C	exonic	ARID4B	synonymous SNV	397G:p.E299E,ARID4B:NM_016374:exon11:c.A897G:p.E299E,ARID4B:N	NA	rs12731746	NA
chr1	2.36E+08	2.36E+08	G	C	UTR3	B3GALNT2	NA	NA	NA	rs6429095	NA
chr1	2.36E+08	2.36E+08	G	A	UTR3	B3GALNT2	NA	NA	NA	rs6429096	NA
chr1	2.36E+08	2.36E+08	A	-	UTR3	B3GALNT2	NA	NA	NA	NA	NA
chr1	2.36E+08	2.36E+08	G	C	UTR3	GNG4	NA	NA	NA	rs12022945	NA
chr1	2.37E+08	2.37E+08	A	-	downstream	EDARADD	NA	NA	NA	rs11358746	NA
chr1	2.37E+08	2.37E+08	T	A	exonic	LGALS8	nonsynonymous SNV	:NM_006499:exon4:c.T56A:p.F19Y,LGALS8:NM_201543:exon4:c.T56A	)=COSM146350;OCCURENCE=1(stomacl	rs1126407	B
chr1	2.37E+08	2.37E+08	T	C	exonic	LGALS8	synonymous SNV	:NM_006499:exon4:c.T72C:p.P24P,LGALS8:NM_201543:exon4:c.T72C	)=COSM146830;OCCURENCE=1(stomacl	rs1041934	NA
chr1	2.37E+08	2.37E+08	C	T	exonic	LGALS8	nonsynonymous SNV	NM_006499:exon4:c.C106T:p.R36C,LGALS8:NM_201543:exon4:c.C106	)=COSM146351;OCCURENCE=1(stomacl	rs1041935	B
chr1	2.37E+08	2.37E+08	G	A	exonic	LGALS8	synonymous SNV	M_006499:exon5:c.G330A:p.L110L,LGALS8:NM_201543:exon5:c.G330	NA	rs2472126	NA
chr1	2.37E+08	2.37E+08	A	C	UTR3	HEATR1,LGALS8	NA	NA	NA	rs6989	NA
chr1	2.37E+08	2.37E+08	C	T	exonic	HEATR1	synonymous SNV	HEATR1:NM_018072:exon43:c.G6159A:p.S2053S	)=COSM146832;OCCURENCE=1(stomacl	rs2275685	NA
chr1	2.37E+08	2.37E+08	C	A	exonic	HEATR1	synonymous SNV	HEATR1:NM_018072:exon20:c.G2565T:p.V855V	)=COSM146833;OCCURENCE=1(stomacl	rs4518892	NA
chr1	2.37E+08	2.37E+08	T	-	UTR3	MTR	NA	NA	NA	rs67705775	NA
chr1	2.37E+08	2.37E+08	A	G	UTR3	MTR	NA	NA	NA	rs1050993	NA
chr1	2.37E+08	2.37E+08	G	T	UTR3	MTR	NA	NA	NA	rs10925264	NA
chr1	2.38E+08	2.38E+08	C	G	UTR3	RYR2	NA	NA	NA	rs12406863	NA
chr1	2.4E+08	2.4E+08	C	T	exonic	FMN2	nonsynonymous SNV	FMN2:NM_020066:exon5:c.C2834T:p.P945L	NA	rs193049501	NA
chr1	2.4E+08	2.4E+08	G	T	exonic	FMN2	synonymous SNV	FMN2:NM_020066:exon5:c.G2868T:p.P956P	ID=COSM679781;OCCURENCE=1(lung)	NA	NA
chr1	2.4E+08	2.4E+08	C	A	exonic	FMN2	synonymous SNV	FMN2:NM_020066:exon5:c.C2988A:p.G996G	NA	rs71646887	NA
chr1	2.4E+08	2.4E+08	C	T	exonic	FMN2	synonymous SNV	FMN2:NM_020066:exon5:c.C2994T:p.P998P	NA	rs11586155	NA
chr1	2.4E+08	2.4E+08	G	A	exonic	FMN2	synonymous SNV	FMN2:NM_020066:exon5:c.G3000A:p.P1000P	NA	rs71646889	NA
chr1	2.45E+08	2.45E+08	G	C	exonic	HNRNPU	synonymous SNV	VI_004501:exon1:c.C519G:p.P173P,HNRNPU:NM_031844:exon1:c.C51	NA	NA	NA

chr1	2.45E+08	2.45E+08	C	T	exonic	HNRNPU	synonymous SNV	NM_004501:exon1:c.G261A:p.E87E,HNRNPU:NM_031844:exon1:c.G26	NA	rs6675421	NA
chr1	2.45E+08	2.45E+08	A	G	intronic	EFCAB2	NA	NA	NA	rs7542813	NA
chr1	2.47E+08	2.47E+08	-	CACACACA	ncRNA_UTR3	ZNF670	NA	NA	NA	NA	NA
chr1	2.47E+08	2.47E+08	G	A	UTR3	ZNF669	NA	NA	NA	rs140375048	NA
chr1	2.47E+08	2.47E+08	G	A	UTR3	ZNF124	NA	NA	NA	NA	NA
chr1	2.48E+08	2.48E+08	G	C	UTR3	OR2C3	NA	NA	NA	rs4925558	NA
chr1	2.48E+08	2.48E+08	T	G	exonic	OR11L1	nonsynonymous SNV	OR11L1:NM_001001959:exon1:c.A539C:p.D180A	NA	NA	P
chr1	2.49E+08	2.49E+08	C	G	exonic	OR2T2	synonymous SNV	OR2T2:NM_001004136:exon1:c.C651G:p.V217V	NA	rs151176830	NA
chr1	2.49E+08	2.49E+08	G	A	exonic	OR2T34	nonsynonymous SNV	OR2T34:NM_001001821:exon1:c.C892T:p.R298C	NA	rs148590921	D
chr1	2.49E+08	2.49E+08	G	A	exonic	OR2T34	nonsynonymous SNV	OR2T34:NM_001001821:exon1:c.C766T:p.L256F	NA	rs200427293	B
chr1	2.49E+08	2.49E+08	C	G	exonic	OR2T34	synonymous SNV	OR2T34:NM_001001821:exon1:c.G765C:p.L255L	NA	rs201475070	NA
chr1	2.49E+08	2.49E+08	A	G	exonic	OR2T34	nonsynonymous SNV	OR2T34:NM_001001821:exon1:c.T464C:p.V155A	ID=COSM120080;OCCURENCE=1(ovary)	rs150601708	B
chr1	2.49E+08	2.49E+08	T	C	exonic	OR2T35	synonymous SNV	OR2T35:NM_001001827:exon1:c.A303G:p.Q101Q	NA	rs145908089	NA
chr1	2.49E+08	2.49E+08	C	-	UTR5	SH3BP5L	NA	NA	NA	rs28362662	NA
chr2	224919	224919	A	G	exonic	SH3YL1	synonymous SNV	SH3YL1:NM_015677:exon9:c.T783C:p.S261S	NA	rs2290911	NA
chr2	905494	905494	C	G	intergenic	822(dist=41382),SNTG2(dist=	NA	NA	NA	NA	NA
chr2	905805	905805	G	C	intergenic	822(dist=41693),SNTG2(dist=	NA	NA	NA	rs111612803	NA
chr2	3718692	3718692	C	T	UTR5	ALLC	NA	NA	NA	rs13409104	NA
chr2	5838684	5838686	AAA	-	UTR3	SOX11	NA	NA	NA	rs201898989	NA
chr2	9544866	9544866	-	T	UTR3	ASAP2	NA	NA	NA	NA	NA
chr2	9546272	9546272	T	G	UTR3	ITGB1BP1	NA	NA	NA	rs10173551	NA
chr2	9546549	9546552	TGTT	-	UTR3	ITGB1BP1	NA	NA	NA	NA	NA
chr2	9629855	9629857	AAA	-	UTR3	ADAM17	NA	NA	NA	NA	NA
chr2	10281514	10281514	C	T	upstream	C2orf48	NA	NA	NA	rs17313542	NA
chr2	11966640	11966641	AA	-	UTR3	LPIN1	NA	NA	NA	NA	NA
chr2	18768850	18768850	A	G	exonic	NT5C1B,NT5C1B-RDH14	synonymous SNV	.P13P,NT5C1B:NM_001199088:exon2:c.T39C:p.P13P,NT5C1B-RDH14:N	NA	rs16985306	NA
chr2	20131079	20131079	T	C	exonic	WDR35	nonsynonymous SNV	20779:exon24:c.A2915G:p.E972G,WDR35:NM_001006657:exon25:c.A	NA	rs1191778	B
chr2	20648145	20648145	A	C	UTR3	RHOB	NA	NA	NA	NA	NA
chr2	24307470	24307470	G	T	UTR5	TP53I3	NA	NA	NA	rs33958836	NA
chr2	25050977	25050977	T	C	exonic	ADCY3	synonymous SNV	ADCY3:NM_004036:exon13:c.A2226G:p.E742E	NA	rs7566416	NA
chr2	27002706	27002706	C	T	UTR3	SLC35F6	NA	NA	NA	NA	NA
chr2	27477981	27477981	A	C	UTR3	SLC30A3	NA	NA	NA	NA	NA
chr2	27804534	27804534	C	G	exonic	C2orf16	nonsynonymous SNV	C2orf16:NM_032266:exon1:c.C5095G:p.Q1699E	NA	NA	NA
chr2	27804548	27804548	T	C	exonic	C2orf16	synonymous SNV	C2orf16:NM_032266:exon1:c.T5109C:p.R1703R	NA	NA	NA
chr2	27804607	27804607	G	A	exonic	C2orf16	nonsynonymous SNV	C2orf16:NM_032266:exon1:c.G5168A:p.R1723Q	NA	rs202029351	NA
chr2	27838058	27838058	C	A	exonic	ZNF512	synonymous SNV	_001271288:exon10:c.C924A:p.L308L,ZNF512:NM_001271289:exon10:	NA	rs11127071	NA
chr2	29023100	29023100	T	C	UTR3	PPP1CB	NA	NA	NA	NA	NA
chr2	29226512	29226512	A	G	exonic	FAM179A	nonsynonymous SNV	FAM179A:NM_199280:exon6:c.A794G:p.Q265R	NA	rs12623297	NA
chr2	30480656	30480656	-	TTTTTT	UTR3	LBH	NA	NA	NA	rs113721073	NA
chr2	30864774	30864774	-	T	UTR3	LCLAT1	NA	NA	NA	NA	NA
chr2	30865267	30865267	T	-	UTR3	LCLAT1	NA	NA	NA	NA	NA
chr2	30865753	30865753	-	TT	UTR3	LCLAT1	NA	NA	NA	NA	NA
chr2	30865757	30865757	C	T	UTR3	LCLAT1	NA	NA	NA	rs201834336	NA
chr2	31396115	31396115	G	A	UTR3	CAPN14	NA	NA	NA	rs2044244	NA
chr2	31457155	31457167	ACCCCGG	-	UTR5	EHD3	NA	NA	NA	rs144609065	NA
chr2	31489628	31489629	AC	-	UTR3	EHD3	NA	NA	NA	rs67643147	NA
chr2	31610701	31610701	C	T	exonic	XDH	synonymous SNV	XDH:NM_000379:exon8:c.G627A:p.E209E	NA	rs45575032	NA
chr2	32843295	32843295	A	-	UTR3	BIRC6	NA	NA	NA	rs140477433	NA
chr2	32983526	32983526	C	T	exonic	TTC27	synonymous SNV	01193509:exon13:c.C1470T:p.N490N,TTC27:NM_017735:exon13:c.C1	=COSM148836;OCCURENCE=1(stomac	rs2273665	NA
chr2	33412077	33412077	G	A	exonic	LTBP1	synonymous SNV	A:p.A126A,LTBP1:NM_001166265:exon2:c.G378A:p.A126A,LTBP1:NM_	NA	rs1065324	NA
chr2	33447202	33447202	C	T	exonic	LTBP1	synonymous SNV	T:p.N294N,LTBP1:NM_001166265:exon5:c.C882T:p.N294N,LTBP1:NM_	NA	rs2290448	NA
chr2	33951299	33951299	-	TT	ncRNA_exonic	MYADML	NA	NA	NA	rs71409649	NA
chr2	36777378	36777378	T	-	UTR3	CRIM1	NA	NA	NA	rs10707574	NA
chr2	37230734	37230734	C	T	exonic	HEATR5B	synonymous SNV	HEATR5B:NM_019024:exon31:c.G5001A:p.Q1667Q	NA	rs17497654	NA
chr2	37310452	37310452	C	A	exonic	HEATR5B	nonsynonymous SNV	HEATR5B:NM_019024:exon2:c.G106T:p.V36F	NA	NA	D
chr2	37323460	37323460	G	C	UTR3	GPATCH11	NA	NA	NA	rs12474977	NA
chr2	38522140	38522140	T	G	UTR3	ATL2	NA	NA	NA	rs200194110	NA
chr2	38522144	38522144	C	-	UTR3	ATL2	NA	NA	NA	NA	NA
chr2	38522260	38522260	A	T	UTR3	ATL2	NA	NA	NA	rs6987	NA
chr2	43015719	43015719	T	C	exonic	HAAO	nonsynonymous SNV	HAAO:NM_012205:exon2:c.A109G:p.I37V	NA	rs3816183	NA
chr2	43019730	43019730	A	G	UTR5	HAAO	NA	NA	NA	rs2304656	NA
chr2	44036922	44036922	T	C	UTR3	DYNC2LI1	NA	NA	NA	rs10186552	NA
chr2	44546016	44546016	C	T	UTR3	PREPL	NA	NA	NA	rs8410	NA
chr2	44999604	44999604	T	-	UTR3	CAMKMT	NA	NA	NA	rs199836049	NA
chr2	45172416	45172416	G	A	UTR3	SIX3	NA	NA	NA	NA	NA
chr2	46746963	46746963	A	G	UTR5	ATP6V1E2	NA	NA	NA	rs4952830	NA

chr2	46852121	46852121	T	-	UTR3	CRIPT	NA	NA	NA	NA	NA
chr2	46852211	46852211	T	-	UTR3	CRIPT	NA	NA	NA	NA	NA
chr2	50147836	50147843	GTGTGTGT	-	UTR3	NRXN1	NA	NA	NA	NA	NA
chr2	54120025	54120025	A	T	exonic	PSME4	nonsynonymous SNV	PSME4:NM_014614:exon36:c.T4111A:p.S1371T	NA	rs805408	NA
chr2	54532431	54532431	T	C	UTR3	ACYP2	NA	NA	NA	rs17045807	NA
chr2	54895818	54895818	T	-	UTR3	SPTBN1	NA	NA	NA	rs201990044	NA
chr2	55199932	55199932	T	-	UTR3	RTN4	NA	NA	NA	NA	NA
chr2	55402225	55402225	C	T	UTR3	CLHC1	NA	NA	NA	rs1133182	NA
chr2	58388696	58388696	A	G	exonic	FANCL	synonymous SNV	_001114636:exon12:c.T996C:p.S332S,FANCL:NM_018062:exon12:c.T9	NA	rs848291	NA
chr2	64119863	64119863	-	T	UTR3	VPS54	NA	NA	NA	NA	NA
chr2	64819250	64819250	A	-	UTR3	AFTPH	NA	NA	NA	rs200722568	NA
chr2	65299085	65299085	A	C	exonic	CEP68	synonymous SNV	CEP68:NM_015147:exon3:c.A855C:p.S285S	NA	NA	NA
chr2	67637218	67637221	TGAT	-	UTR3	ETAA1	NA	NA	NA	rs35271433	NA
chr2	68358046	68358046	C	T	UTR3	WDR92	NA	NA	NA	rs199882206	NA
chr2	68406127	68406127	-	AAAAA	UTR3	PPP3R1	NA	NA	NA	NA	NA
chr2	68406149	68406149	G	A	UTR3	PPP3R1	NA	NA	NA	NA	NA
chr2	68406155	68406155	G	A	UTR3	PPP3R1	NA	NA	NA	rs201762572	NA
chr2	69550811	69550811	T	-	UTR3	GFPT1	NA	NA	NA	rs36057221	NA
chr2	69689509	69689509	A	T	UTR3	AAK1	NA	NA	NA	rs72833931	NA
chr2	69689809	69689809	A	-	UTR3	AAK1	NA	NA	NA	NA	NA
chr2	71004492	71004492	T	C	UTR3	FIGLA	NA	NA	ID=COSN404629;OCCURENCE=1(lung)	rs56316086	NA
chr2	71004494	71004494	A	T	UTR3	FIGLA	NA	NA	ID=COSN404630;OCCURENCE=1(lung)	rs56135050	NA
chr2	71036190	71036190	A	-	UTR3	CLEC4F	NA	NA	NA	rs149426278	NA
chr2	71351487	71351487	G	A	exonic	MCEE	nonsynonymous SNV	MCEE:NM_032601:exon2:c.C227T:p.A76V	NA	rs11541017	B
chr2	72707874	72707874	A	G	exonic	EXOC6B	synonymous SNV	EXOC6B:NM_015189:exon17:c.T1671C:p.V557V	NA	rs653220	NA
chr2	74300717	74300717	T	C	exonic	TET3	synonymous SNV	TET3:NM_144993:exon2:c.T2131C:p.L711L	)=COSM148878;OCCURENCE=1(stomac	rs7560668	NA
chr2	75884989	75884989	T	-	UTR3	MRPL19	NA	NA	NA	rs35072018	NA
chr2	85549595	85549595	T	A	UTR3	TGOLN2	NA	NA	NA	NA	NA
chr2	85549650	85549650	G	C	UTR3	TGOLN2	NA	NA	NA	NA	NA
chr2	85776950	85776950	A	C	UTR3	GGCX	NA	NA	NA	rs78909830	NA
chr2	85833907	85833907	T	-	UTR3	C2orf68	NA	NA	NA	NA	NA
chr2	87012399	87012399	-	AA	UTR3	CD8A	NA	NA	NA	NA	NA
chr2	89065449	89065449	G	A	ncRNA_exonic	ANKRD36BP2	NA	NA	NA	rs113082666	NA
chr2	91843471	91843471	C	T	ncRNA_exonic	LOC654342	NA	NA	NA	rs1048212	NA
chr2	91963845	91963845	G	A	ncRNA_exonic	GGT8P	NA	NA	NA	NA	NA
chr2	91963880	91963880	A	G	ncRNA_exonic	GGT8P	NA	NA	NA	rs114148846	NA
chr2	91963899	91963899	C	T	ncRNA_exonic	GGT8P	NA	NA	NA	NA	NA
chr2	91963908	91963908	T	G	ncRNA_exonic	GGT8P	NA	NA	NA	NA	NA
chr2	91963953	91963953	A	G	ncRNA_exonic	GGT8P	NA	NA	NA	rs4622773	NA
chr2	91970053	91970053	A	G	ncRNA_exonic	GGT8P	NA	NA	NA	rs200455966	NA
chr2	91970089	91970089	G	C	ncRNA_exonic	GGT8P	NA	NA	NA	rs147452528	NA
chr2	91970112	91970112	C	T	ncRNA_exonic	GGT8P	NA	NA	NA	rs139857385	NA
chr2	92129194	92129194	C	T	ncRNA_exonic	ACTR3BP2	NA	NA	NA	rs12483966	NA
chr2	95522747	95522747	C	G	ncRNA_exonic	ANKRD20A8P	NA	NA	NA	rs10874467	NA
chr2	96676301	96676301	C	A	ncRNA_exonic	FAHD2CP	NA	NA	NA	rs2443817	NA
chr2	97808510	97808510	G	A	exonic	ANKRD36	nonsynonymous SNV	ANKRD36:NM_001164315:exon8:c.G839A:p.G280D	NA	rs199920970	NA
chr2	97808515	97808515	G	A	exonic	ANKRD36	nonsynonymous SNV	ANKRD36:NM_001164315:exon8:c.G844A:p.E282K	NA	rs201265245	NA
chr2	97808524	97808524	A	G	exonic	ANKRD36	nonsynonymous SNV	ANKRD36:NM_001164315:exon8:c.A853G:p.I285V	NA	rs202223019	NA
chr2	97808529	97808529	A	G	exonic	ANKRD36	synonymous SNV	ANKRD36:NM_001164315:exon8:c.A858G:p.S286S	NA	NA	NA
chr2	97808562	97808562	A	C	exonic	ANKRD36	nonsynonymous SNV	ANKRD36:NM_001164315:exon8:c.A891C:p.K297N	NA	rs75359815	NA
chr2	97815047	97815047	T	G	exonic	ANKRD36	synonymous SNV	ANKRD36:NM_001164315:exon12:c.T1050G:p.P350P	NA	NA	NA
chr2	97815050	97815050	T	C	exonic	ANKRD36	synonymous SNV	ANKRD36:NM_001164315:exon12:c.T1053C:p.D351D	NA	rs2190034	NA
chr2	97815051	97815051	G	A	exonic	ANKRD36	nonsynonymous SNV	ANKRD36:NM_001164315:exon12:c.G1054A:p.A352T	NA	rs200092365	NA
chr2	97915895	97915895	T	C	exonic	ANKRD36	synonymous SNV	ANKRD36:NM_001164315:exon75:c.T5817C:p.F1939F	NA	rs201565865	NA
chr2	97915896	97915896	G	A	exonic	ANKRD36	nonsynonymous SNV	ANKRD36:NM_001164315:exon75:c.G5818A:p.E1940K	NA	rs199628660	NA
chr2	98127653	98127653	T	G	exonic	ANKRD36B	nonsynonymous SNV	ANKRD36B:NM_025190:exon39:c.A3668C:p.N1223T	NA	rs62157161	NA
chr2	98127659	98127659	T	C	exonic	ANKRD36B	nonsynonymous SNV	ANKRD36B:NM_025190:exon39:c.A3662G:p.E1221G	NA	NA	NA
chr2	98128066	98128066	A	G	exonic	ANKRD36B	synonymous SNV	ANKRD36B:NM_025190:exon39:c.T3255C:p.V1085V	NA	NA	NA
chr2	98128128	98128128	G	A	exonic	ANKRD36B	nonsynonymous SNV	ANKRD36B:NM_025190:exon39:c.C3193T:p.L1065F	NA	rs75197050	NA
chr2	98128364	98128364	T	C	exonic	ANKRD36B	nonsynonymous SNV	ANKRD36B:NM_025190:exon39:c.A2957G:p.H986R	NA	rs200630019	NA
chr2	98128402	98128402	C	A	exonic	ANKRD36B	nonsynonymous SNV	ANKRD36B:NM_025190:exon39:c.G2919T:p.K973N	NA	rs111605455	NA
chr2	98128421	98128421	T	C	exonic	ANKRD36B	nonsynonymous SNV	ANKRD36B:NM_025190:exon39:c.A2900G:p.K967R	NA	rs112192400	NA
chr2	98128472	98128472	C	A	exonic	ANKRD36B	nonsynonymous SNV	ANKRD36B:NM_025190:exon39:c.G2849T:p.R950L	NA	rs200057888	NA
chr2	99237792	99237792	C	T	UTR3	MGAT4A	NA	NA	NA	rs11892744	NA
chr2	1.07E+08	1.07E+08	T	A	exonic	RGPD3	nonsynonymous SNV	RGPD3:NM_001144013:exon17:c.A2468T:p.K823M	132759;OCCURENCE=1(central_nervou	rs62152467	NA
chr2	1.07E+08	1.07E+08	C	G	exonic	RGPD3	nonsynonymous SNV	RGPD3:NM_001144013:exon16:c.G2233C:p.E745Q	NA	rs143879890	NA

chr2	1.07E+08	1.07E+08	C	T	exonic	RGPD3	nonsynonymous SNV	RGPD3:NM_001144013:exon4:c.G343A:p.E115K	NA	rs199932968	NA
chr2	1.07E+08	1.07E+08	AACAAC	-	UTR3	ST6GAL2	NA	NA	NA	rs200860434	NA
chr2	1.09E+08	1.09E+08	T	G	UTR3	RGPD4	NA	NA	NA	rs3202255	NA
chr2	1.09E+08	1.09E+08	G	T	UTR3	RGPD4	NA	NA	NA	rs2439167	NA
chr2	1.09E+08	1.09E+08	T	-	UTR3	SULT1C2	NA	NA	NA	rs35976654	NA
chr2	1.09E+08	1.09E+08	T	-	UTR3	SULT1C4	NA	NA	NA	rs57444248	NA
chr2	1.09E+08	1.09E+08	C	T	UTR3	GCC2	NA	NA	NA	rs1138412	NA
chr2	1.1E+08	1.1E+08	-	T	UTR3	10-Sep	NA	NA	NA	NA	NA
chr2	1.1E+08	1.1E+08	A	-	UTR3	SOWAHC	NA	NA	NA	NA	NA
chr2	1.12E+08	1.12E+08	A	G	intronic	ACOXL	NA	NA	NA	rs7558938	NA
chr2	1.14E+08	1.14E+08	A	C	exonic	FOXD4L1	nonsynonymous SNV	FOXD4L1:NM_012184:exon1:c.A145C:p.K49Q	NA	rs2757970	B
chr2	1.14E+08	1.14E+08	A	G	ncRNA_exonic	WASH2P	NA	NA	NA	rs200864112	NA
chr2	1.14E+08	1.14E+08	C	G	ncRNA_exonic	WASH2P	NA	NA	SN404663,COSN404664;OCCURENCE=1	rs1128453	NA
chr2	1.14E+08	1.14E+08	A	G	ncRNA_exonic	DDX11L2	NA	NA	672,COSN404673,COSN404671;OCCURI	rs2747970	NA
chr2	1.2E+08	1.2E+08	T	G	UTR3	EN1	NA	NA	NA	NA	NA
chr2	1.21E+08	1.21E+08	G	A	exonic	EPB41L5	nonsynonymous SNV	01184937:exon17:c.G1384A:p.A462T,EPB41L5:NM_020909:exon17:c.G	NA	rs1034489	B
chr2	1.21E+08	1.21E+08	T	C	ncRNA_exonic	LOC84931	NA	NA	NA	rs1880544	NA
chr2	1.22E+08	1.22E+08	CACACA	-	UTR3	CLASP1	NA	NA	NA	NA	NA
chr2	1.28E+08	1.28E+08	TT	-	UTR3	MAP3K2	NA	NA	NA	NA	NA
chr2	1.28E+08	1.28E+08	G	T	exonic	PROC	synonymous SNV	PROC:NM_000312:exon6:c.G423T:p.S141S	NA	rs5936	NA
chr2	1.29E+08	1.29E+08	A	-	UTR3	POLR2D	NA	NA	NA	NA	NA
chr2	1.29E+08	1.29E+08	C	T	UTR3	HS6ST1	NA	NA	NA	rs35261195	NA
chr2	1.29E+08	1.29E+08	A	C	UTR3	HS6ST1	NA	NA	NA	NA	NA
chr2	1.29E+08	1.29E+08	T	C	UTR3	HS6ST1	NA	NA	NA	NA	NA
chr2	1.31E+08	1.31E+08	T	C	exonic	POTEF	nonsynonymous SNV	POTEF:NM_001099771:exon17:c.A2687G:p.H896R	NA	rs201946437	NA
chr2	1.31E+08	1.31E+08	C	A	exonic	POTEF	nonsynonymous SNV	POTEF:NM_001099771:exon17:c.G2601T:p.E867D	NA	rs2599794	NA
chr2	1.32E+08	1.32E+08	A	G	UTR5	ARHGEF4	NA	NA	NA	rs3739127	NA
chr2	1.32E+08	1.32E+08	A	-	UTR3	PLEKHB2	NA	NA	NA	NA	NA
chr2	1.33E+08	1.33E+08	G	A	ncRNA_exonic	ANKRD30BL	NA	NA	NA	rs79828184	NA
chr2	1.33E+08	1.33E+08	A	G	ncRNA_exonic	ANKRD30BL	NA	NA	NA	rs74546783	NA
chr2	1.33E+08	1.33E+08	C	T	ncRNA_exonic	ANKRD30BL	NA	NA	NA	rs112570337	NA
chr2	1.33E+08	1.33E+08	G	A	ncRNA_exonic	ANKRD30BL	NA	NA	NA	rs112577594	NA
chr2	1.33E+08	1.33E+08	T	G	ncRNA_exonic	ANKRD30BL	NA	NA	NA	rs75692539	NA
chr2	1.33E+08	1.33E+08	-	GGCG	ncRNA_exonic	ANKRD30BL	NA	NA	NA	NA	NA
chr2	1.36E+08	1.36E+08	T	G	UTR5	CCNT2	NA	NA	NA	NA	NA
chr2	1.36E+08	1.36E+08	A	-	UTR3	RAB3GAP1	NA	NA	NA	rs200869351	NA
chr2	1.39E+08	1.39E+08	T	G	UTR5	HNMT	NA	NA	ID=COSN404685;OCCURENCE=1(lung)	NA	NA
chr2	1.41E+08	1.41E+08	C	T	exonic	LRP1B	synonymous SNV	LRP1B:NM_018557:exon69:c.G10650A:p.E3550E	NA	rs16843864	NA
chr2	1.41E+08	1.41E+08	G	A	exonic	LRP1B	synonymous SNV	LRP1B:NM_018557:exon55:c.C8823T:p.D2941D	NA	rs35296183	NA
chr2	1.49E+08	1.49E+08	A	G	UTR5	ORC4	NA	NA	NA	rs897172	NA
chr2	1.52E+08	1.52E+08	G	C	UTR5	NMI	NA	NA	NA	rs2194492	NA
chr2	1.53E+08	1.53E+08	AA	-	UTR3	STAM2	NA	NA	NA	NA	NA
chr2	1.56E+08	1.56E+08	A	C	UTR5	KCNJ3	NA	NA	NA	NA	NA
chr2	1.57E+08	1.57E+08	G	C	UTR3	GPD2	NA	NA	NA	rs1991776	NA
chr2	1.6E+08	1.6E+08	G	A	UTR5	WDSUB1	NA	NA	NA	rs58048811	NA
chr2	1.66E+08	1.66E+08	A	T	UTR5	SCN2A	NA	NA	NA	rs76193610	NA
chr2	1.67E+08	1.67E+08	A	C	exonic	CSRNP3	nonsynonymous SNV	M_024969:exon3:c.A271C:p.T91P,CSRNP3:NM_001172173:exon5:c.A2	NA	NA	D
chr2	1.7E+08	1.7E+08	A	G	exonic	NOSTRIN	synonymous SNV	52946:exon15:c.A1188G:p.G396G,NOSTRIN:NM_001039724:exon16:c.	NA	rs3931	NA
chr2	1.7E+08	1.7E+08	G	A	UTR3	LRP2	NA	NA	NA	NA	NA
chr2	1.7E+08	1.7E+08	T	C	exonic	LRP2	nonsynonymous SNV	LRP2:NM_004525:exon42:c.A7894G:p.N2632D	NA	rs17848169	P
chr2	1.72E+08	1.72E+08	C	G	UTR3	DCAF17	NA	NA	NA	rs17221367	NA
chr2	1.77E+08	1.77E+08	AAA	-	UTR3	KIAA1715	NA	NA	NA	NA	NA
chr2	1.78E+08	1.78E+08	TA	-	UTR3	AGPS	NA	NA	NA	NA	NA
chr2	1.78E+08	1.78E+08	T	-	UTR3	PDE11A	NA	NA	NA	rs71010806	NA
chr2	1.78E+08	1.78E+08	C	T	UTR3	PDE11A	NA	NA	NA	NA	NA
chr2	1.79E+08	1.79E+08	AA	-	ncRNA_UTR3	OSBPL6	NA	NA	NA	NA	NA
chr2	1.79E+08	1.79E+08	G	T	exonic	TTN	nonsynonymous SNV	exon155:c.C60045A:p.S20015R,TTN:NM_133378:exon275:c.C78960A:	NA	NA	NA
chr2	1.79E+08	1.79E+08	A	T	exonic	TTN	nonsynonymous SNV	:exon155:c.T60026A:p.I20009K,TTN:NM_133378:exon275:c.T78941A:	NA	NA	NA
chr2	1.79E+08	1.79E+08	G	T	exonic	TTN	nonsynonymous SNV	exon155:c.C60024A:p.H20008Q,TTN:NM_133378:exon275:c.C78939A:	NA	NA	NA
chr2	1.84E+08	1.84E+08	G	A	exonic	DNAJC10	nonsynonymous SNV	_001271581:exon4:c.G226A:p.D76N,DNAJC10:NM_018981:exon4:c.G	NA	rs6729801	B
chr2	1.88E+08	1.88E+08	AA	-	UTR3	TFPI	NA	NA	NA	NA	NA
chr2	1.88E+08	1.88E+08	A	-	UTR3	TFPI	NA	NA	NA	rs72023563	NA
chr2	1.89E+08	1.89E+08	T	G	intronic	GULP1	NA	NA	NA	NA	NA
chr2	1.89E+08	1.89E+08	AA	-	UTR3	GULP1	NA	NA	NA	NA	NA
chr2	1.9E+08	1.9E+08	A	C	exonic	DIRC1	nonsynonymous SNV	DIRC1:NM_052952:exon2:c.T151G:p.S51A	NA	rs72902678	NA
chr2	1.91E+08	1.91E+08	G	A	UTR5	INPP1	NA	NA	NA	rs1372054	NA

chr2	1.92E+08	1.92E+08	G	A	exonic	MYO1B	synonymous SNV	0A:p.E490E,MYO1B:NM_001161819:exon16:c.G1470A:p.E490E,MYO1B	NA	rs13018796	NA
chr2	1.97E+08	1.97E+08	TT	-	UTR3	SLC39A10	NA	NA	NA	NA	NA
chr2	1.97E+08	1.97E+08	-	GTGGGGGC	UTR3	STK17B	NA	NA	NA	rs113084773	NA
chr2	1.97E+08	1.97E+08	C	T	UTR3	STK17B	NA	NA	NA	rs6745041	NA
chr2	1.98E+08	1.98E+08	T	C	exonic	HSPD1	synonymous SNV	NM_002156:exon3:c.A273G:p.K91K,HSPD1:NM_199440:exon3:c.A273G	NA	rs8539	NA
chr2	1.99E+08	1.99E+08	G	A	exonic	RFTN2	synonymous SNV	RFTN2:NM_144629:exon3:c.C369T:p.L123L	NA	rs10497806	NA
chr2	2E+08	2E+08	G	T	UTR3	SATB2	NA	NA	NA	NA	NA
chr2	2.01E+08	2.01E+08	-	T	UTR3	SPATS2L	NA	NA	NA	NA	NA
chr2	2.01E+08	2.01E+08	A	-	UTR3	SPATS2L	NA	NA	NA	rs11313633	NA
chr2	2.02E+08	2.02E+08	-	AT	UTR3	NDUFB3	NA	NA	NA	rs150160270	NA
chr2	2.02E+08	2.02E+08	C	T	UTR3	CASP8	NA	NA	NA	rs2141331	NA
chr2	2.02E+08	2.02E+08	A	-	UTR3	TRAK2	NA	NA	NA	rs138690285	NA
chr2	2.02E+08	2.02E+08	A	T	exonic	ALS2CR11	nonsynonymous SNV	01168217:exon11:c.T1128A:p.H376Q,ALS2CR11:NM_001168221:exon1	NA	rs10804117	B
chr2	2.03E+08	2.03E+08	C	T	exonic	ALS2	synonymous SNV	ALS2:NM_020919:exon25:c.G3885A:p.A1295A	NA	rs34946105	NA
chr2	2.04E+08	2.04E+08	A	-	UTR3	ALS2CR8	NA	NA	NA	NA	NA
chr2	2.04E+08	2.04E+08	C	T	exonic	CYP20A1	nonsynonymous SNV	CYP20A1:NM_177538:exon10:c.C1036T:p.L346F	NA	rs1048013	B
chr2	2.04E+08	2.04E+08	C	A	UTR3	CYP20A1	NA	NA	NA	NA	NA
chr2	2.04E+08	2.04E+08	T	G	UTR3	CYP20A1	NA	NA	NA	NA	NA
chr2	2.04E+08	2.04E+08	G	A	UTR3	CYP20A1	NA	NA	NA	rs62183912	NA
chr2	2.04E+08	2.04E+08	A	C	UTR3	CYP20A1	NA	NA	NA	rs6435181	NA
chr2	2.04E+08	2.04E+08	T	-	UTR3	CYP20A1	NA	NA	NA	rs11304494	NA
chr2	2.04E+08	2.04E+08	A	-	UTR3	RAPH1	NA	NA	NA	NA	NA
chr2	2.06E+08	2.06E+08	C	T	exonic	PARD3B	synonymous SNV	9T:p.A113A,PARD3B:NM_152526:exon3:c.C339T:p.A113A,PARD3B:NM	NA	rs236843	NA
chr2	2.07E+08	2.07E+08	T	C	exonic	NRP2	synonymous SNV	M_201264:exon6:c.T870C:p.N290N,NRP2:NM_201266:exon6:c.T870C:	NA	rs2228639	NA
chr2	2.07E+08	2.07E+08	AA	-	UTR3	INO80D	NA	NA	NA	NA	NA
chr2	2.07E+08	2.07E+08	AAA	-	UTR3	INO80D	NA	NA	NA	NA	NA
chr2	2.08E+08	2.08E+08	-	TGTG	UTR3	KLF7	NA	NA	NA	rs113821778	NA
chr2	2.08E+08	2.08E+08	TCTT	-	UTR3	CREB1	NA	NA	NA	rs4025849	NA
chr2	2.09E+08	2.09E+08	AAG	-	UTR3	FZD5	NA	NA	NA	NA	NA
chr2	2.09E+08	2.09E+08	G	-	UTR3	FZD5	NA	NA	NA	NA	NA
chr2	2.09E+08	2.09E+08	G	A	UTR3	FZD5	NA	NA	NA	NA	NA
chr2	2.09E+08	2.09E+08	-	T	UTR3	PLEKHM3	NA	NA	NA	NA	NA
chr2	2.11E+08	2.11E+08	CACACA	-	UTR3	MAP2	NA	NA	NA	NA	NA
chr2	2.11E+08	2.11E+08	T	G	exonic	ACADL	nonsynonymous SNV	ACADL:NM_001608:exon9:c.A997C:p.K333Q	NA	rs2286963	D
chr2	2.11E+08	2.11E+08	-	TCT	exonic	CPS1	nonframeshift insertion	CPS1:NM_001122633:exon2:c.13_14insTCT:p.I5delinsIF	NA	rs3835047	NA
chr2	2.14E+08	2.14E+08	-	A	UTR3	IKZF2	NA	NA	NA	rs11450321	NA
chr2	2.18E+08	2.18E+08	C	T	ncRNA_exonic	DIRC3	NA	NA	NA	rs3732007	NA
chr2	2.19E+08	2.19E+08	C	A	exonic	CTDSP1	synonymous SNV	C204A:p.G68G,CTDSP1:NM_021198:exon2:c.C204A:p.G68G,CTDSP1:NI	NA	rs2227251	NA
chr2	2.2E+08	2.2E+08	A	G	UTR3	PRKAG3	NA	NA	NA	rs6436094	NA
chr2	2.2E+08	2.2E+08	G	C	exonic	PRKAG3	nonsynonymous SNV	PRKAG3:NM_017431:exon3:c.C211G:p.P71A	NA	rs692243	P
chr2	2.2E+08	2.2E+08	A	C	UTR3	CDK5R2	NA	NA	NA	rs142470311	NA
chr2	2.2E+08	2.2E+08	C	T	UTR3	FAM134A	NA	NA	NA	NA	NA
chr2	2.2E+08	2.2E+08	G	T	UTR3	FAM134A	NA	NA	NA	NA	NA
chr2	2.2E+08	2.2E+08	G	T	UTR3	FAM134A	NA	NA	NA	NA	NA
chr2	2.2E+08	2.2E+08	A	C	exonic	CHPF	nonsynonymous SNV	_001195731:exon4:c.T791G:p.L264R,CHPF:NM_024536:exon4:c.T1277	NA	NA	D
chr2	2.24E+08	2.24E+08	T	C	exonic	KCNE4	synonymous SNV	KCNE4:NM_080671:exon2:c.T417C:p.P139P	NA	rs10201907	NA
chr2	2.25E+08	2.25E+08	TT	-	UTR3	AP1S3	NA	NA	NA	NA	NA
chr2	2.25E+08	2.25E+08	A	G	UTR3	AP1S3	NA	NA	NA	NA	NA
chr2	2.26E+08	2.26E+08	TGG	-	UTR5	NYAP2	NA	NA	NA	rs10578238	NA
chr2	2.28E+08	2.28E+08	TA	-	UTR3	IRS1	NA	NA	NA	rs138636103	NA
chr2	2.28E+08	2.28E+08	A	C	exonic	IRS1	nonsynonymous SNV	IRS1:NM_005544:exon1:c.T890G:p.V297G	NA	NA	D
chr2	2.28E+08	2.28E+08	T	G	exonic	IRS1	nonsynonymous SNV	IRS1:NM_005544:exon1:c.A833C:p.N278T	NA	NA	P
chr2	2.28E+08	2.28E+08	C	T	exonic	COL4A4	synonymous SNV	COL4A4:NM_000092:exon42:c.G4080A:p.P1360P	NA	rs2228556	NA
chr2	2.28E+08	2.28E+08	C	T	exonic	COL4A4	nonsynonymous SNV	COL4A4:NM_000092:exon42:c.G3979A:p.V1327M	NA	rs2229813	NA
chr2	2.28E+08	2.28E+08	G	A	exonic	COL4A4	nonsynonymous SNV	COL4A4:NM_000092:exon21:c.C1444T:p.P482S	NA	rs2229814	B
chr2	2.28E+08	2.28E+08	TTTA	-	UTR3	AGFG1	NA	NA	NA	rs55924655	NA
chr2	2.28E+08	2.28E+08	G	A	UTR3	C2orf83	NA	NA	NA	NA	NA
chr2	2.32E+08	2.32E+08	T	-	UTR3	B3GNT7	NA	NA	NA	NA	NA
chr2	2.33E+08	2.33E+08	A	G	exonic	ECEL1	nonsynonymous SNV	ECEL1:NM_004826:exon7:c.T1240C:p.S414P	NA	NA	D
chr2	2.34E+08	2.34E+08	A	G	exonic	DGKD	nonsynonymous SNV	_003648:exon9:c.A1007G:p.D336G,DGKD:NM_152879:exon10:c.A1139	NA	rs73995951	D
chr2	2.35E+08	2.35E+08	AAACAAAC	-	UTR3	HJURP	NA	NA	NA	rs150635580	NA
chr2	2.35E+08	2.35E+08	-	TTAT	UTR3	TRPM8	NA	NA	NA	rs138810119	NA
chr2	2.35E+08	2.35E+08	T	-	UTR3	ARL4C	NA	NA	NA	rs11476419	NA
chr2	2.38E+08	2.38E+08	C	G	exonic	COL6A3	nonsynonymous SNV	p.D2224H,COL6A3:NM_057167:exon38:c.G7873C:p.D2625H,COL6A3:N	NA	rs36104025	D
chr2	2.39E+08	2.39E+08	C	A	exonic	ESPNL	synonymous SNV	ESPNL:NM_194312:exon2:c.C403A:p.R135R	NA	rs34878344	NA
chr2	2.39E+08	2.39E+08	A	C	UTR3	KLHL30	NA	NA	NA	NA	NA

chr2	2.39E+08	2.39E+08	G	T	ncRNA_exonic	LOC151174	NA	NA	NA	NA	NA
chr2	2.4E+08	2.4E+08	-	ATAGATAG	UTR3	HDAC4	NA	NA	NA	NA	NA
chr2	2.4E+08	2.4E+08	G	A	exonic	HDAC4	synonymous SNV	HDAC4:NM_006037:exon21:c.C2565T:p.P855P	NA	rs1063639	NA
chr2	2.4E+08	2.4E+08	T	C	exonic	HDAC4	synonymous SNV	HDAC4:NM_006037:exon18:c.A2361G:p.T787T	NA	rs61752234	NA
chr2	2.41E+08	2.41E+08	T	C	exonic	NDUFA10	synonymous SNV	NDUFA10:NM_004544:exon2:c.A105G:p.K35K	NA	rs2083411	NA
chr2	2.41E+08	2.41E+08	A	G	exonic	PRR21	synonymous SNV	PRR21:NM_001080835:exon1:c.T969C:p.H323H	ID=COSM41568;OCCURENCE=1(lung)	rs112962758	NA
chr2	2.41E+08	2.41E+08	C	T	exonic	PRR21	nonsynonymous SNV	PRR21:NM_001080835:exon1:c.G964A:p.V322I	NA	rs141005183	B
chr2	2.41E+08	2.41E+08	C	T	exonic	PRR21	nonsynonymous SNV	PRR21:NM_001080835:exon1:c.G712A:p.V238I	NA	rs199895422	NA
chr2	2.41E+08	2.41E+08	A	G	exonic	PRR21	synonymous SNV	PRR21:NM_001080835:exon1:c.T633C:p.H211H	NA	NA	NA
chr2	2.41E+08	2.41E+08	C	T	exonic	PRR21	nonsynonymous SNV	PRR21:NM_001080835:exon1:c.G292A:p.V98I	1129269,COSM1129268;OCCURENCE=1	rs112152720	B
chr2	2.41E+08	2.41E+08	A	G	exonic	PRR21	nonsynonymous SNV	PRR21:NM_001080835:exon1:c.T181C:p.S61P	NA	rs74006013	B
chr2	2.41E+08	2.41E+08	G	A	exonic	ANKMY1	nonsynonymous SNV	ANKMY1:NM_016552:exon10:c.C1946T:p.T649M	NA	rs35044862	B
chr2	2.42E+08	2.42E+08	C	T	exonic	PASK	nonsynonymous SNV	_001252124:exon6:c.G748A:p.V250I,PASK:NM_015148:exon6:c.G748	149119,COSM149120;OCCURENCE=1(	rs1470414	B
chr2	2.42E+08	2.42E+08	T	G	exonic	HDLBP	nonsynonymous SNV	24C:p.D775A,HDLBP:NM_005336:exon19:c.A2423C:p.D808A,HDLBP:N	NA	NA	D
chr2	2.43E+08	2.43E+08	G	A	UTR3	GAL3ST2	NA	NA	NA	rs744986	NA
chr2	2.43E+08	2.43E+08	T	A	ncRNA_exonic	LOC728323	NA	NA	NA	rs1817840	NA
chr2	2.43E+08	2.43E+08	C	T	ncRNA_exonic	LOC728323	NA	NA	NA	rs1817839	NA
chr3	3151759	3151759	C	T	UTR5	IL5RA	NA	NA	NA	rs2290608	NA
chr3	5222488	5222488	-	GGGGATA	UTR3	ARL8B	NA	NA	NA	rs143599604	NA
chr3	8615563	8615563	A	-	ncRNA_exonic	LINC00312	NA	NA	NA	rs5846604	NA
chr3	8775661	8775661	C	T	exonic	CAV3	synonymous SNV	NM_001234:exon1:c.C99T:p.N33N,CAV3:NM_033337:exon1:c.C99T:p	NA	rs1008642	NA
chr3	9519364	9519365	TT	-	UTR3	SETD5	NA	NA	NA	NA	NA
chr3	9745687	9745687	C	A	exonic	CPNE9	unknown	UNKNOWN	NA	NA	P
chr3	9862366	9862366	G	T	exonic	ARPC4-TTLL3,TTLL3	synonymous SNV	25930:exon7:c.G1095T:p.P365P,ARPC4-TTLL3:NM_001198793:exon8:	NA	NA	NA
chr3	10284239	10284239	A	C	UTR3	IRAK2	NA	NA	NA	NA	NA
chr3	10443827	10443827	G	C	exonic	ATP2B2	synonymous SNV	_001001331:exon4:c.C603G:p.V201V,ATP2B2:NM_001683:exon4:c.C6	NA	rs13084776	NA
chr3	14106084	14106084	T	C	ncRNA_exonic	TPRXL	NA	NA	NA	NA	NA
chr3	14106117	14106117	T	C	ncRNA_exonic	TPRXL	NA	NA	NA	NA	NA
chr3	14106258	14106258	T	C	ncRNA_exonic	TPRXL	NA	NA	NA	NA	NA
chr3	14106285	14106285	T	C	ncRNA_exonic	TPRXL	NA	NA	NA	NA	NA
chr3	14526667	14526667	-	GTGTGT	UTR3	SLC6A6	NA	NA	NA	NA	NA
chr3	14745857	14745857	G	A	exonic	C3orf20	nonsynonymous SNV	6A:p.A176T,C3orf20:NM_001184958:exon7:c.G526A:p.A176T,C3orf20	NA	rs17040196	B
chr3	18427924	18427924	G	T	exonic	SATB1	synonymous SNV	36A:p.P462P,SATB1:NM_001195470:exon8:c.C1386A:p.P462P,SATB1:N	NA	rs2229261	NA
chr3	18465235	18465235	A	-	UTR5	SATB1	NA	NA	NA	rs3838737	NA
chr3	19962044	19962044	G	A	exonic	EFHB	nonsynonymous SNV	EFHB:NM_144715:exon2:c.C805T:p.P269S	NA	rs13078867	NA
chr3	19988758	19988758	-	CGG	UTR5	RAB5A	NA	NA	NA	NA	NA
chr3	20212189	20212189	A	G	UTR3	SGOL1	NA	NA	NA	rs1052930	NA
chr3	21448109	21448109	A	C	ncRNA_exonic	VENTXP7	NA	NA	NA	NA	NA
chr3	21448155	21448155	G	T	ncRNA_exonic	VENTXP7	NA	NA	NA	NA	NA
chr3	21448176	21448181	AAAAAA	-	ncRNA_exonic	VENTXP7	NA	NA	NA	NA	NA
chr3	27414561	27414561	-	A	UTR3	SLC4A7	NA	NA	NA	NA	NA
chr3	29322966	29322966	-	T	UTR5	RBMS3	NA	NA	NA	NA	NA
chr3	33174156	33174156	T	G	exonic	CRTAP	synonymous SNV	CRTAP:NM_006371:exon5:c.T1032G:p.T344T	=COSM149349;OCCURENCE=1(stomac	rs1135127	NA
chr3	33174168	33174168	G	A	exonic	CRTAP	synonymous SNV	CRTAP:NM_006371:exon5:c.G1044A:p.S348S	NA	rs1135128	NA
chr3	33188385	33188385	A	-	UTR3	CRTAP	NA	NA	NA	rs57153345	NA
chr3	33430960	33430960	T	G	UTR3	UBP1	NA	NA	NA	NA	NA
chr3	37028931	37028931	-	T	UTR3	EPM2AIP1	NA	NA	NA	rs199570773	NA
chr3	37030404	37030404	A	-	UTR3	EPM2AIP1	NA	NA	NA	NA	NA
chr3	38179998	38179998	A	C	UTR5	MYD88	NA	NA	NA	NA	NA
chr3	38493852	38493852	C	A	ncRNA_exonic	ACVR2B-AS1	NA	NA	NA	rs3762788	NA
chr3	38534223	38534258	ACACACAC	-	UTR3	ACVR2B	NA	NA	NA	NA	NA
chr3	38908944	38908944	T	C	exonic	SCN11A	synonymous SNV	SCN11A:NM_014139:exon23:c.A3819G:p.E1273E	NA	rs148945365	NA
chr3	39186728	39186728	C	T	exonic	CSRNP1	synonymous SNV	CSRNP1:NM_033027:exon3:c.G225A:p.R75R	NA	rs784517	NA
chr3	39521532	39521532	A	-	UTR5	MOBP	NA	NA	NA	NA	NA
chr3	39556457	39556468	GTGTGTGT	-	UTR3	MOBP	NA	NA	NA	rs147433364	NA
chr3	40503520	40503520	-	CTGCTGCT	exonic	RPL14	nonframeshift insertion	46insCTGCTGCTG:p.T149delinsTAAA,RPL14:NM_003973:exon6:c.445_	NA	NA	NA
chr3	41281412	41281412	-	T	UTR3	CTNNB1	NA	NA	NA	rs34653633	NA
chr3	41877414	41877414	T	C	exonic	ULK4	nonsynonymous SNV	ULK4:NM_017886:exon18:c.A1706G:p.K569R	NA	rs3774372	P
chr3	42706403	42706403	T	G	UTR3	ZBTB47	NA	NA	NA	NA	NA
chr3	42825115	42825115	C	T	UTR3	HIGD1A	NA	NA	NA	rs6442067	NA
chr3	42825568	42825569	AA	-	UTR3	HIGD1A	NA	NA	NA	NA	NA
chr3	45077441	45077441	G	T	UTR3	CLEC3B	NA	NA	NA	NA	NA
chr3	45153708	45153708	G	A	exonic	CDCP1	synonymous SNV	NM_022842:exon3:c.C522T:p.I174I,CDCP1:NM_178181:exon3:c.C522T	NA	rs35501071	NA
chr3	46501213	46501213	T	C	exonic	LTF	nonsynonymous SNV	IM_001199149:exon2:c.A8G:p.K3R,LTF:NM_002343:exon2:c.A140G:p.	NA	rs1126478	B
chr3	46501268	46501268	C	T	exonic	LTF	nonsynonymous SNV	LTF:NM_002343:exon2:c.G85A:p.A29T	NA	rs1126477	B
chr3	46501284	46501284	-	CTT	exonic	LTF	nonframeshift insertion	LTF:NM_002343:exon2:c.69_70insAAG:p.R23delinsRR	NA	rs10662431	NA

chr3	46945263	46945263	-	A	UTR3	PTH1R	NA	NA	NA	NA	NA
chr3	48016254	48016254	-	A	UTR3	MAP4	NA	NA	NA	NA	NA
chr3	48419897	48419897	C	T	exonic	FBXW12	nonsynonymous SNV	_001159929:exon5:c.C439T:p.R147W,FBXW12:NM_207102:exon6:c.C4	NA	rs6442117	P
chr3	48485493	48485493	A	G	UTR3	TMA7	NA	NA	NA	rs7126	NA
chr3	49067296	49067296	A	-	UTR3	QRICH1	NA	NA	NA	NA	NA
chr3	49273996	49273996	C	T	exonic	CCDC36	synonymous SNV	NM_001135197:exon3:c.C72T:p.S24S,CCDC36:NM_178173:exon5:c.C7	NA	rs12631989	NA
chr3	49315576	49315576	T	-	UTR3	USP4	NA	NA	NA	rs11309567	NA
chr3	49395674	49395679	GCCGCC	-	exonic	GPX1	unknown	UNKNOWN	D=COSM446703;OCCURENCE=1(breast	rs28362588	NA
chr3	49723823	49723823	A	G	exonic	MST1	synonymous SNV	MST1:NM_020998:exon8:c.T939C:p.T313T	NA	rs7431215	NA
chr3	50306752	50306752	-	C	exonic	SEMA3B	unknown	UNKNOWN	NA	rs112058630	NA
chr3	52407041	52407041	C	T	exonic	DNAH1	synonymous SNV	DNAH1:NM_015512:exon44:c.C6957T:p.H2319H	NA	rs1546737	NA
chr3	53226653	53226653	-	AA	UTR3	PRKCD	NA	NA	NA	NA	NA
chr3	53321361	53321365	TTTTT	-	UTR3	DCP1A	NA	NA	NA	NA	NA
chr3	54354510	54354510	T	C	exonic	CACNA2D3	synonymous SNV	CACNA2D3:NM_018398:exon3:c.T246C:p.I82I	NA	rs9879885	NA
chr3	54952397	54952397	G	A	UTR3	LRTM1	NA	NA	NA	NA	NA
chr3	54952419	54952419	C	A	UTR3	LRTM1	NA	NA	NA	NA	NA
chr3	55502166	55502166	-	A	UTR3	WNT5A	NA	NA	NA	NA	NA
chr3	57401178	57401178	G	C	exonic	DNAH12	nonsynonymous SNV	DNAH12:NM_178504:exon38:c.C5771G:p.A1924G	NA	rs140326175	NA
chr3	57430988	57430988	G	A	exonic	DNAH12	synonymous SNV	DNAH12:NM_178504:exon28:c.C4269T:p.Y1423Y	NA	rs6768943	NA
chr3	57857404	57857404	C	T	exonic	SLMAP	nonsynonymous SNV	SLMAP:NM_007159:exon11:c.C1177T:p.H393Y	NA	NA	P
chr3	58304608	58304608	C	G	UTR3	RPP14	NA	NA	NA	rs3210776	NA
chr3	59908126	59908126	A	G	exonic	FHIT	synonymous SNV	M_001166243:exon8:c.T294C:p.H98H,FHIT:NM_002012:exon8:c.T294C	)=COSM149406;OCCURENCE=1(stomacl	rs1385816	NA
chr3	63985905	63985905	T	A	UTR3	ATXN7	NA	NA	NA	NA	NA
chr3	63985952	63985952	T	G	UTR3	ATXN7	NA	NA	NA	NA	NA
chr3	63985956	63985956	T	G	UTR3	ATXN7	NA	NA	NA	NA	NA
chr3	63988880	63988880	G	T	UTR3	ATXN7	NA	NA	NA	NA	NA
chr3	64672480	64672480	T	G	exonic	ADAMTS9	nonsynonymous SNV	ADAMTS9:NM_182920:exon2:c.A280C:p.T94P	NA	NA	P
chr3	65346027	65346027	T	-	UTR3	MAGI1	NA	NA	NA	NA	NA
chr3	66271429	66271429	A	C	UTR5	SLC25A26	NA	NA	NA	NA	NA
chr3	67048762	67048762	T	C	UTR5	KBTBD8	NA	NA	NA	rs4856833	NA
chr3	69070836	69070836	-	T	UTR3	TMF1	NA	NA	NA	NA	NA
chr3	71007806	71007806	T	A	UTR3	FOXP1	NA	NA	NA	NA	NA
chr3	71007843	71007843	T	C	UTR3	FOXP1	NA	NA	NA	NA	NA
chr3	72426068	72426068	T	A	UTR3	RYBP	NA	NA	NA	rs13065475	NA
chr3	72427252	72427252	-	T	UTR3	RYBP	NA	NA	NA	rs11455962	NA
chr3	74344356	74344356	T	G	exonic	CNTN3	synonymous SNV	CNTN3:NM_020872:exon18:c.A2433C:p.A811A	NA	rs10490832	NA
chr3	75713507	75713507	C	T	ncRNA_exonic	FLJ20518	NA	NA	NA	rs199666615	NA
chr3	75715734	75715734	G	A	ncRNA_exonic	FLJ20518	NA	NA	NA	rs138571914	NA
chr3	75716122	75716122	T	C	ncRNA_exonic	FLJ20518	NA	NA	NA	rs139598107	NA
chr3	75786042	75786042	G	A	exonic	ZNF717	nonsynonymous SNV	ZNF717:NM_001128223:exon5:c.C2732T:p.S911F	NA	rs140243156	NA
chr3	75786288	75786288	A	G	exonic	ZNF717	nonsynonymous SNV	ZNF717:NM_001128223:exon5:c.T2486C:p.L829P	l191698;OCCURENCE=1(central_nervou	rs147227180	NA
chr3	75786866	75786866	G	T	exonic	ZNF717	synonymous SNV	ZNF717:NM_001128223:exon5:c.C1908A:p.T636T	NA	rs79516801	NA
chr3	75787099	75787100	CT	-	exonic	ZNF717	frameshift deletion	ZNF717:NM_001128223:exon5:c.1674_1675del:p.558_559del	l192025;OCCURENCE=1(central_nervou	rs146514807	NA
chr3	75787202	75787202	G	T	exonic	ZNF717	synonymous SNV	ZNF717:NM_001128223:exon5:c.C1572A:p.V524V	NA	rs76713735	NA
chr3	75787204	75787204	C	A	exonic	ZNF717	nonsynonymous SNV	ZNF717:NM_001128223:exon5:c.G1570T:p.V524F	NA	rs75063165	NA
chr3	75787208	75787208	G	A	exonic	ZNF717	synonymous SNV	ZNF717:NM_001128223:exon5:c.C1566T:p.L522L	NA	rs79982156	NA
chr3	75787225	75787225	G	A	exonic	ZNF717	nonsynonymous SNV	ZNF717:NM_001128223:exon5:c.C1549T:p.R517C	NA	NA	NA
chr3	75787228	75787228	A	C	exonic	ZNF717	nonsynonymous SNV	ZNF717:NM_001128223:exon5:c.T1546G:p.F516V	NA	rs202139504	NA
chr3	75787235	75787235	C	T	exonic	ZNF717	synonymous SNV	ZNF717:NM_001128223:exon5:c.G1539A:p.G513G	NA	rs200228654	NA
chr3	75787251	75787251	T	C	exonic	ZNF717	nonsynonymous SNV	ZNF717:NM_001128223:exon5:c.A1523G:p.E508G	NA	NA	NA
chr3	75787253	75787253	G	A	exonic	ZNF717	synonymous SNV	ZNF717:NM_001128223:exon5:c.C1521T:p.Y507Y	NA	NA	NA
chr3	75787265	75787265	C	G	exonic	ZNF717	synonymous SNV	ZNF717:NM_001128223:exon5:c.G1509C:p.G503G	NA	rs144538707	NA
chr3	75787305	75787305	C	T	exonic	ZNF717	nonsynonymous SNV	ZNF717:NM_001128223:exon5:c.G1469A:p.R490H	NA	rs77389541	NA
chr3	75787353	75787353	G	A	exonic	ZNF717	nonsynonymous SNV	ZNF717:NM_001128223:exon5:c.C1421T:p.T474I	NA	rs141733687	NA
chr3	75787405	75787405	C	T	exonic	ZNF717	nonsynonymous SNV	ZNF717:NM_001128223:exon5:c.G1369A:p.G457R	NA	rs141106119	NA
chr3	75787427	75787427	T	C	exonic	ZNF717	synonymous SNV	ZNF717:NM_001128223:exon5:c.A1347G:p.K449K	NA	rs199721002	NA
chr3	75787712	75787712	G	A	exonic	ZNF717	synonymous SNV	ZNF717:NM_001128223:exon5:c.C1062T:p.L354L	NA	NA	NA
chr3	75787725	75787725	C	T	exonic	ZNF717	nonsynonymous SNV	ZNF717:NM_001128223:exon5:c.G1049A:p.R350H	NA	rs186706183	NA
chr3	75787729	75787729	G	A	exonic	ZNF717	nonsynonymous SNV	ZNF717:NM_001128223:exon5:c.C1045T:p.R349C	NA	NA	NA
chr3	75788088	75788088	C	T	exonic	ZNF717	nonsynonymous SNV	ZNF717:NM_001128223:exon5:c.G686A:p.R229K	NA	rs151216671	NA
chr3	75788199	75788199	T	C	exonic	ZNF717	nonsynonymous SNV	ZNF717:NM_001128223:exon5:c.A575G:p.H192R	NA	rs146090776	NA
chr3	97517180	97517180	C	A	UTR3	ARL6	NA	NA	NA	NA	NA
chr3	98304467	98304467	T	C	exonic	CPOX	synonymous SNV	CPOX:NM_000097:exon5:c.A990G:p.E330E	NA	rs1729995	NA
chr3	98515882	98515883	AA	-	UTR3	DCBLD2	NA	NA	NA	NA	NA
chr3	1.01E+08	1.01E+08	T	A	exonic	SENP7	nonsynonymous SNV	)1077203:exon12:c.A1641T:p.Q547H,SENP7:NM_020654:exon13:c.A1	=COSM1130394;OCCURENCE=1(prostat	rs2433031	P
chr3	1.08E+08	1.08E+08	AA	-	UTR3	BBX	NA	NA	NA	NA	NA

chr3	1.08E+08	1.08E+08	A	-	UTR3	BBX	NA	NA	NA	NA	NA
chr3	1.11E+08	1.11E+08	-	TGACTTAT	intronic	PLCXD2	NA	NA	NA	NA	NA
chr3	1.12E+08	1.12E+08	A	G	UTR3	ABHD10	NA	NA	NA	rs1127853	NA
chr3	1.12E+08	1.12E+08	T	G	UTR5	TAGLN3	NA	NA	NA	NA	NA
chr3	1.13E+08	1.13E+08	A	-	UTR3	GTPBP8	NA	NA	NA	rs200301737	NA
chr3	1.13E+08	1.13E+08	-	T	UTR3	BOC	NA	NA	NA	NA	NA
chr3	1.13E+08	1.13E+08	A	T	UTR3	BOC	NA	NA	NA	rs5022662	NA
chr3	1.14E+08	1.14E+08	G	A	UTR3	ZNF80	NA	NA	NA	NA	NA
chr3	1.14E+08	1.14E+08	A	C	UTR3	ZNF80	NA	NA	NA	NA	NA
chr3	1.14E+08	1.14E+08	C	A	UTR3	ZNF80	NA	NA	NA	NA	NA
chr3	1.14E+08	1.14E+08	T	A	UTR3	ZNF80	NA	NA	NA	NA	NA
chr3	1.19E+08	1.19E+08	C	G	exonic	C3orf30	nonsynonymous SNV	C3orf30:NM_152539:exon2:c.C1419G:p.D473E	NA	rs9289122	B
chr3	1.19E+08	1.19E+08	T	-	UTR3	PLA1A	NA	NA	NA	NA	NA
chr3	1.21E+08	1.21E+08	T	C	UTR3	GTF2E1	NA	NA	NA	rs55697401	NA
chr3	1.21E+08	1.21E+08	T	C	exonic	POLQ	nonsynonymous SNV	POLQ:NM_199420:exon28:c.A7538G:p.Q2513R	NA	rs1381057	NA
chr3	1.21E+08	1.21E+08	T	C	UTR3	ARGFX	NA	NA	NA	rs11711973	NA
chr3	1.21E+08	1.21E+08	T	G	UTR3	ARGFX	NA	NA	NA	rs11712050	NA
chr3	1.21E+08	1.21E+08	AT	-	UTR3	ARGFX	NA	NA	NA	rs3044706	NA
chr3	1.22E+08	1.22E+08	A	G	UTR3	CASR	NA	NA	NA	rs9740	NA
chr3	1.22E+08	1.22E+08	A	T	UTR3	CCDC58	NA	NA	NA	rs73855768	NA
chr3	1.22E+08	1.22E+08	-	A	UTR3	KPNA1	NA	NA	NA	NA	NA
chr3	1.22E+08	1.22E+08	G	A	UTR3	DTX3L	NA	NA	NA	rs73192142	NA
chr3	1.22E+08	1.22E+08	A	C	UTR3	DTX3L	NA	NA	NA	NA	NA
chr3	1.22E+08	1.22E+08	T	-	UTR3	DTX3L	NA	NA	NA	NA	NA
chr3	1.23E+08	1.23E+08	T	A	ncRNA_exonic	LOC100129550	NA	NA	NA	rs28375649	NA
chr3	1.24E+08	1.24E+08	C	A	UTR3	CCDC14	NA	NA	NA	NA	NA
chr3	1.24E+08	1.24E+08	G	A	exonic	KALRN	synonymous SNV	007064:exon2:c.G135A:p.V45V,KALRN:NM_001024660:exon35:c.G522	NA	rs1708303	NA
chr3	1.24E+08	1.24E+08	T	C	exonic	KALRN	synonymous SNV	_007064:exon2:c.T243C:p.L81L,KALRN:NM_001024660:exon35:c.T533	NA	rs1660038	NA
chr3	1.26E+08	1.26E+08	G	C	UTR5	ROPN1B	NA	NA	NA	NA	NA
chr3	1.26E+08	1.26E+08	T	G	UTR5	ROPN1B	NA	NA	NA	NA	NA
chr3	1.26E+08	1.26E+08	G	A	UTR3	KLF15	NA	NA	NA	rs190340804	NA
chr3	1.26E+08	1.26E+08	T	-	UTR3	ZXDC	NA	NA	NA	NA	NA
chr3	1.26E+08	1.26E+08	G	A	UTR3	ZXDC	NA	NA	NA	rs813556	NA
chr3	1.28E+08	1.28E+08	C	T	ncRNA_UTR5	DNAJB8	NA	NA	NA	rs12634139	NA
chr3	1.28E+08	1.28E+08	C	A	UTR5	GATA2	NA	NA	NA	rs1806462	NA
chr3	1.28E+08	1.28E+08	T	-	UTR3	C3orf27	NA	NA	NA	rs200346912	NA
chr3	1.29E+08	1.29E+08	-	T	UTR3	COPG1	NA	NA	NA	rs35073725	NA
chr3	1.29E+08	1.29E+08	C	T	exonic	EFCAB12	nonsynonymous SNV	EFCAB12:NM_207307:exon3:c.G590A:p.R197H	NA	rs62266958	NA
chr3	1.29E+08	1.29E+08	G	A	UTR5	EFCAB12	NA	NA	NA	rs3796391	NA
chr3	1.29E+08	1.29E+08	T	-	UTR3	RHO	NA	NA	NA	rs11359208	NA
chr3	1.3E+08	1.3E+08	G	A	exonic	ALG1L2	synonymous SNV	ALG1L2:NM_001136152:exon2:c.G45A:p.P15P	NA	rs6804080	NA
chr3	1.3E+08	1.3E+08	-	TTT	ncRNA_exonic	COL6A4P2	NA	NA	NA	NA	NA
chr3	1.3E+08	1.3E+08	T	-	intronic	COL6A5	NA	NA	NA	rs11355796	NA
chr3	1.34E+08	1.34E+08	G	C	UTR3	RAB6B	NA	NA	NA	NA	NA
chr3	1.36E+08	1.36E+08	A	-	UTR3	MSL2	NA	NA	NA	NA	NA
chr3	1.36E+08	1.36E+08	A	-	UTR3	MSL2	NA	NA	NA	NA	NA
chr3	1.36E+08	1.36E+08	G	A	UTR3	MSL2	NA	NA	NA	rs78145147	NA
chr3	1.39E+08	1.39E+08	-	TT	UTR3	PRR23C	NA	NA	NA	rs61429017	NA
chr3	1.39E+08	1.39E+08	TGC	-	UTR5	PRR23C	NA	NA	NA	rs63140560	NA
chr3	1.41E+08	1.41E+08	-	A	UTR3	ZBTB38	NA	NA	NA	NA	NA
chr3	1.42E+08	1.42E+08	A	-	UTR3	GK5	NA	NA	NA	rs71153943	NA
chr3	1.42E+08	1.42E+08	A	G	UTR3	GK5	NA	NA	NA	rs112882257	NA
chr3	1.42E+08	1.42E+08	T	-	UTR3	GK5	NA	NA	NA	rs139089663	NA
chr3	1.42E+08	1.42E+08	C	T	UTR3	GK5	NA	NA	NA	rs115936014	NA
chr3	1.42E+08	1.42E+08	A	-	UTR3	GK5	NA	NA	NA	rs137909394	NA
chr3	1.44E+08	1.44E+08	T	-	UTR3	C3orf58	NA	NA	NA	NA	NA
chr3	1.49E+08	1.49E+08	A	G	UTR3	TM4SF18	NA	NA	NA	rs76335061	NA
chr3	1.5E+08	1.5E+08	AAA	-	UTR3	TSC22D2	NA	NA	NA	rs71739355	NA
chr3	1.51E+08	1.51E+08	AC	-	UTR3	CLRN1	NA	NA	NA	rs34027634	NA
chr3	1.51E+08	1.51E+08	A	G	ncRNA_exonic	LOC201651	NA	NA	NA	rs6809359	NA
chr3	1.52E+08	1.52E+08	C	T	ncRNA_UTR5	AADAC	NA	NA	NA	rs2293004	NA
chr3	1.53E+08	1.53E+08	T	-	UTR3	RAP2B	NA	NA	NA	rs112079528	NA
chr3	1.53E+08	1.53E+08	C	T	UTR3	RAP2B	NA	NA	NA	rs138115718	NA
chr3	1.53E+08	1.53E+08	C	T	UTR3	RAP2B	NA	NA	NA	rs144462183	NA
chr3	1.54E+08	1.54E+08	AC	-	UTR3	ARHGEF26	NA	NA	NA	NA	NA
chr3	1.54E+08	1.54E+08	G	A	UTR3	ARHGEF26	NA	NA	NA	NA	NA

chr3	1.54E+08	1.54E+08	TATA	-	UTR3	DHX36	NA	NA	NA	NA	NA
chr3	1.56E+08	1.56E+08	-	G	ncRNA_exonic	TIPARP-AS1	NA	NA	NA	rs74614741	NA
chr3	1.57E+08	1.57E+08	T	-	ncRNA_exonic	PA2G4P4	NA	NA	NA	NA	NA
chr3	1.57E+08	1.57E+08	C	T	UTR3	C3orf55	NA	NA	NA	rs4395360	NA
chr3	1.59E+08	1.59E+08	A	G	exonic	MFSD1	nonsynonymous SNV	_001167903:exon7:c.A688G:p.I230V,MFSD1:NM_022736:exon8:c.A8(	NA	rs3765083	B
chr3	1.65E+08	1.65E+08	T	C	exonic	SI	nonsynonymous SNV	SI:NM_001041:exon7:c.A691G:p.T231A	NA	rs9283633	NA
chr3	1.65E+08	1.65E+08	C	T	UTR3	BCHE	NA	NA	NA	rs3495	NA
chr3	1.67E+08	1.67E+08	T	A	exonic	ZBBX	nonsynonymous SNV	480T:p.K160N,ZBBX:NM_001199202:exon9:c.A393T:p.K131N,ZBBX:NM	NA	rs4619784	P
chr3	1.67E+08	1.67E+08	A	G	exonic	WDR49	nonsynonymous SNV	WDR49:NM_178824:exon14:c.T1952C:p.L651P	NA	rs13060964	B
chr3	1.69E+08	1.69E+08	G	A	ncRNA_exonic	EGFEM1P	NA	NA	NA	rs139803907	NA
chr3	1.7E+08	1.7E+08	G	T	UTR3	CLDN11	NA	NA	NA	NA	NA
chr3	1.71E+08	1.71E+08	G	T	UTR3	EIF5A2	NA	NA	NA	rs192130148	NA
chr3	1.71E+08	1.71E+08	C	T	UTR3	EIF5A2	NA	NA	NA	NA	NA
chr3	1.71E+08	1.71E+08	C	T	UTR3	EIF5A2	NA	NA	NA	NA	NA
chr3	1.71E+08	1.71E+08	C	T	exonic	TNIK	nonsynonymous SNV	1161565:exon24:c.G2893A:p.E965K,TNIK:NM_001161560:exon25:c.G3	NA	NA	NA
chr3	1.71E+08	1.71E+08	C	T	exonic	TNIK	nonsynonymous SNV	01161565:exon23:c.G2743A:p.A915T,TNIK:NM_001161560:exon24:c.G	NA	rs17857452	NA
chr3	1.71E+08	1.71E+08	A	G	exonic	PLD1	synonymous SNV	01130081:exon16:c.T1770C:p.R590R,PLD1:NM_002662:exon17:c.T18	NA	rs2124147	NA
chr3	1.77E+08	1.77E+08	A	-	UTR3	TBL1XR1	NA	NA	NA	NA	NA
chr3	1.8E+08	1.8E+08	T	G	UTR3	CCDC39	NA	NA	NA	NA	NA
chr3	1.81E+08	1.81E+08	CACACACA	-	ncRNA_exonic	SOX2-OT	NA	NA	NA	NA	NA
chr3	1.83E+08	1.83E+08	T	G	UTR3	KLHL6	NA	NA	NA	NA	NA
chr3	1.84E+08	1.84E+08	A	G	exonic	PARL	synonymous SNV	PARL:NM_018622:exon6:c.T648C:p.H216H	)=COSM149527;OCCURENCE=1(stomac	rs13091	NA
chr3	1.84E+08	1.84E+08	G	-	exonic	HTR3E	frameshift deletion	52delG:p.R21fs,HTR3E:NM_182589:exon1:c.62delG:p.R21fs,HTR3E:NM	NA	rs5855015	NA
chr3	1.84E+08	1.84E+08	G	A	exonic	HTR3E	nonsynonymous SNV	256A:p.A86T,HTR3E:NM_198314:exon1:c.G256A:p.A86T,HTR3E:NM_(	NA	rs7627615	B
chr3	1.85E+08	1.85E+08	T	-	UTR3	IGF2BP2	NA	NA	NA	NA	NA
chr3	1.86E+08	1.86E+08	G	-	UTR3	ETV5	NA	NA	NA	rs200001879	NA
chr3	1.86E+08	1.86E+08	A	G	UTR3	DGKG	NA	NA	NA	rs13089860	NA
chr3	1.86E+08	1.86E+08	T	G	UTR3	TBCCD1	NA	NA	NA	rs4686430	NA
chr3	1.87E+08	1.87E+08	C	T	UTR3	RTP4	NA	NA	NA	rs9028	NA
chr3	1.88E+08	1.88E+08	AGAG	-	ncRNA_exonic	FLJ42393	NA	NA	NA	NA	NA
chr3	1.88E+08	1.88E+08	G	A	ncRNA_exonic	FLJ42393	NA	NA	NA	rs344952	NA
chr3	1.9E+08	1.9E+08	TGTGTG	-	UTR3	LEPREL1	NA	NA	NA	rs150725503	NA
chr3	1.9E+08	1.9E+08	T	C	exonic	LEPREL1	synonymous SNV	LEPREL1:NM_018192:exon2:c.A507G:p.E169E	NA	rs9821880	NA
chr3	1.9E+08	1.9E+08	C	A	UTR5	CLDN1	NA	NA	NA	rs12696600	NA
chr3	1.91E+08	1.91E+08	C	T	UTR3	UTS2B	NA	NA	NA	rs10937466	NA
chr3	1.94E+08	1.94E+08	-	AAA	UTR5	HES1	NA	NA	NA	NA	NA
chr3	1.94E+08	1.94E+08	G	A	UTR3	CPN2	NA	NA	NA	rs1048996	NA
chr3	1.94E+08	1.94E+08	-	G	UTR3	ATP13A3	NA	NA	NA	NA	NA
chr3	1.94E+08	1.94E+08	-	A	UTR3	FAM43A	NA	NA	NA	rs11457805	NA
chr3	1.95E+08	1.95E+08	G	A	UTR3	XXYLT1	NA	NA	NA	rs2720930	NA
chr3	1.95E+08	1.95E+08	A	-	UTR3	ACAP2	NA	NA	NA	rs200248324	NA
chr3	1.95E+08	1.95E+08	-	GGCG	UTR3	APOD	NA	NA	NA	rs146445395	NA
chr3	1.95E+08	1.95E+08	T	G	UTR3	APOD	NA	NA	NA	NA	NA
chr3	1.95E+08	1.95E+08	T	A	ncRNA_exonic	SDHAP2	NA	NA	ID=COSN404760;OCCURENCE=1(lung)	rs6583274	NA
chr3	1.95E+08	1.95E+08	C	T	ncRNA_exonic	SDHAP2	NA	NA	ID=COSN404761;OCCURENCE=1(lung)	rs6583275	NA
chr3	1.95E+08	1.95E+08	T	A	UTR3	MUC20	NA	NA	NA	rs1808432	NA
chr3	1.95E+08	1.95E+08	G	C	UTR3	MUC20	NA	NA	NA	rs903195	NA
chr3	1.95E+08	1.95E+08	G	-	UTR3	MUC4	NA	NA	NA	NA	NA
chr3	1.95E+08	1.95E+08	G	-	UTR3	MUC4	NA	NA	NA	rs145605189	NA
chr3	1.96E+08	1.96E+08	C	T	exonic	MUC4	nonsynonymous SNV	)A:p.G37D,MUC4:NM_004532:exon3:c.G263A:p.G88D,MUC4:NM_018	NA	rs2259292	NA
chr3	1.96E+08	1.96E+08	A	G	exonic	MUC4	synonymous SNV	MUC4:NM_018406:exon2:c.T12255C:p.D4085D	NA	rs143382980	NA
chr3	1.96E+08	1.96E+08	T	G	exonic	MUC4	nonsynonymous SNV	MUC4:NM_018406:exon2:c.A12016C:p.T4006P	NA	rs199490754	NA
chr3	1.96E+08	1.96E+08	C	T	exonic	MUC4	nonsynonymous SNV	MUC4:NM_018406:exon2:c.G11296A:p.A3766T	NA	rs202193697	NA
chr3	1.96E+08	1.96E+08	G	A	exonic	MUC4	nonsynonymous SNV	MUC4:NM_018406:exon2:c.C11258T:p.A3753V	NA	rs201474621	NA
chr3	1.96E+08	1.96E+08	C	A	exonic	MUC4	nonsynonymous SNV	MUC4:NM_018406:exon2:c.G11209T:p.A3737S	NA	rs112808291	NA
chr3	1.96E+08	1.96E+08	G	T	exonic	MUC4	nonsynonymous SNV	MUC4:NM_018406:exon2:c.C11200A:p.P3734T	NA	rs201456607	NA
chr3	1.96E+08	1.96E+08	C	T	exonic	MUC4	nonsynonymous SNV	MUC4:NM_018406:exon2:c.G11053A:p.D3685N	NA	NA	NA
chr3	1.96E+08	1.96E+08	T	C	exonic	MUC4	nonsynonymous SNV	MUC4:NM_018406:exon2:c.A11032G:p.T3678A	NA	NA	NA
chr3	1.96E+08	1.96E+08	C	G	exonic	MUC4	nonsynonymous SNV	MUC4:NM_018406:exon2:c.G10768C:p.A3590P	NA	NA	NA
chr3	1.96E+08	1.96E+08	G	T	exonic	MUC4	synonymous SNV	MUC4:NM_018406:exon2:c.C10734A:p.S3578S	)=COSM1136990;OCCURENCE=1(kidney	NA	NA
chr3	1.96E+08	1.96E+08	G	C	exonic	MUC4	synonymous SNV	MUC4:NM_018406:exon2:c.C10653G:p.T3551T	NA	NA	NA
chr3	1.96E+08	1.96E+08	C	G	exonic	MUC4	nonsynonymous SNV	MUC4:NM_018406:exon2:c.G10213C:p.D3405H	NA	rs199592227	NA
chr3	1.96E+08	1.96E+08	C	G	exonic	MUC4	nonsynonymous SNV	MUC4:NM_018406:exon2:c.G9903C:p.E3301D	NA	NA	NA
chr3	1.96E+08	1.96E+08	A	G	exonic	MUC4	nonsynonymous SNV	MUC4:NM_018406:exon2:c.T9290C:p.V3097A	NA	rs79582237	NA
chr3	1.96E+08	1.96E+08	C	T	exonic	MUC4	nonsynonymous SNV	MUC4:NM_018406:exon2:c.G8656A:p.A2886T	NA	rs201497773	NA
chr3	1.96E+08	1.96E+08	A	G	exonic	MUC4	nonsynonymous SNV	MUC4:NM_018406:exon2:c.T8572C:p.S2858P	OSM1042886;OCCURENCE=2(endometr	NA	NA

chr3	1.96E+08	1.96E+08	T	C	exonic	MUC4	nonsynonymous SNV	MUC4:NM_018406:exon2:c.A8200G:p.T2734A	NA	NA	NA
chr3	1.96E+08	1.96E+08	G	C	exonic	MUC4	nonsynonymous SNV	MUC4:NM_018406:exon2:c.C8199G:p.D2733E	NA	NA	NA
chr3	1.96E+08	1.96E+08	T	C	exonic	MUC4	nonsynonymous SNV	MUC4:NM_018406:exon2:c.A8029G:p.S2677G	NA	NA	NA
chr3	1.96E+08	1.96E+08	C	G	exonic	MUC4	nonsynonymous SNV	MUC4:NM_018406:exon2:c.G7168C:p.A2390P	NA	NA	NA
chr3	1.96E+08	1.96E+08	C	T	exonic	MUC4	nonsynonymous SNV	MUC4:NM_018406:exon2:c.G7048A:p.A2350T	NA	rs78846267	NA
chr3	1.96E+08	1.96E+08	C	T	exonic	MUC4	nonsynonymous SNV	MUC4:NM_018406:exon2:c.G6952A:p.A2318T	NA	rs71321831	NA
chr3	1.96E+08	1.96E+08	T	A	exonic	MUC4	nonsynonymous SNV	MUC4:NM_018406:exon2:c.A6895T:p.T2299S	149600,COSM149599;OCCURENCE=1(	rs79961534	NA
chr3	1.96E+08	1.96E+08	A	G	exonic	MUC4	nonsynonymous SNV	MUC4:NM_018406:exon2:c.T6814C:p.S2272P	ID=COSM396033;OCCURENCE=1(lung)	rs71187734	NA
chr3	1.96E+08	1.96E+08	G	A	exonic	MUC4	synonymous SNV	MUC4:NM_018406:exon2:c.C6783T:p.D2261D	NA	rs3107748	NA
chr3	1.96E+08	1.96E+08	G	A	exonic	MUC4	nonsynonymous SNV	MUC4:NM_018406:exon2:c.C6164T:p.S2055F	NA	rs113602668	NA
chr3	1.96E+08	1.96E+08	G	A	exonic	MUC4	synonymous SNV	MUC4:NM_018406:exon2:c.C6108T:p.T2036T	149604,COSM149603;OCCURENCE=1(	rs113457754	NA
chr3	1.96E+08	1.96E+08	A	G	exonic	MUC4	synonymous SNV	MUC4:NM_018406:exon2:c.T5700C:p.G1900G	NA	NA	NA
chr3	1.96E+08	1.96E+08	G	C	exonic	MUC4	nonsynonymous SNV	MUC4:NM_018406:exon2:c.C5315G:p.A1772G	NA	rs140116971	NA
chr3	1.96E+08	1.96E+08	A	G	exonic	MUC4	synonymous SNV	MUC4:NM_018406:exon2:c.T5196C:p.T1732T	NA	NA	NA
chr3	1.96E+08	1.96E+08	A	G	exonic	MUC4	nonsynonymous SNV	MUC4:NM_018406:exon2:c.T5162C:p.V1721A	NA	rs202065211	NA
chr3	1.96E+08	1.96E+08	A	G	exonic	MUC4	nonsynonymous SNV	MUC4:NM_018406:exon2:c.T4442C:p.V1481A	NA	NA	NA
chr3	1.96E+08	1.96E+08	A	G	exonic	MUC4	synonymous SNV	MUC4:NM_018406:exon2:c.T3996C:p.T1332T	NA	rs79619815	NA
chr3	1.96E+08	1.96E+08	C	G	exonic	MUC4	nonsynonymous SNV	MUC4:NM_018406:exon2:c.G3981C:p.M1327I	NA	rs200851738	NA
chr3	1.96E+08	1.96E+08	T	A	exonic	MUC4	synonymous SNV	MUC4:NM_018406:exon2:c.A3723T:p.A1241A	NA	NA	NA
chr3	1.96E+08	1.96E+08	T	A	exonic	MUC4	synonymous SNV	MUC4:NM_018406:exon2:c.A3636T:p.G1212G	NA	rs201186280	NA
chr3	1.96E+08	1.96E+08	C	G	exonic	MUC4	nonsynonymous SNV	MUC4:NM_018406:exon2:c.G3443C:p.G1148A	NA	rs201451131	NA
chr3	1.98E+08	1.98E+08	T	C	exonic	LRCH3	synonymous SNV	LRCH3:NM_032773:exon10:c.T1314C:p.Y438Y	NA	rs17850206	NA
chr3	1.98E+08	1.98E+08	T	C	UTR3	LMLN	NA	NA	NA	NA	NA
chr4	664028	664028	-	T	UTR3	PDE6B	NA	NA	NA	NA	NA
chr4	844781	844781	G	A	exonic	GAK	synonymous SNV	GAK:NM_005255:exon26:c.C3600T:p.T1200T	OSM287830;OCCURENCE=1(large_intes	rs75072999	NA
chr4	860192	860192	A	G	exonic	GAK	synonymous SNV	GAK:NM_005255:exon22:c.T3003C:p.S1001S	NA	rs1064207	NA
chr4	944210	944210	A	C	exonic	TMEM175	nonsynonymous SNV	TMEM175:NM_032326:exon4:c.A194C:p.Q65P	NA	rs34884217	B
chr4	1191486	1191486	-	T	ncRNA_exonic	LOC100130872	NA	NA	NA	NA	NA
chr4	1192409	1192409	G	A	ncRNA_exonic	LOC100130872	NA	NA	NA	rs875475	NA
chr4	1388379	1388379	G	A	exonic	CRIPAK	nonsynonymous SNV	CRIPAK:NM_175918:exon1:c.G80A:p.C27Y	NA	rs56109734	D
chr4	1388429	1388429	G	A	exonic	CRIPAK	nonsynonymous SNV	CRIPAK:NM_175918:exon1:c.G130A:p.A44T	NA	rs79298048	B
chr4	1388631	1388631	C	G	exonic	CRIPAK	nonsynonymous SNV	CRIPAK:NM_175918:exon1:c.C332G:p.P111R	ID=COSM227471;OCCURENCE=1(skin)	rs200849975	NA
chr4	1388867	1388867	A	C	exonic	CRIPAK	nonsynonymous SNV	CRIPAK:NM_175918:exon1:c.A568C:p.I190L	NA	rs76058011	NA
chr4	1389154	1389154	G	C	exonic	CRIPAK	synonymous SNV	CRIPAK:NM_175918:exon1:c.G855C:p.P285P	NA	NA	NA
chr4	1389380	1389380	T	G	exonic	CRIPAK	nonsynonymous SNV	CRIPAK:NM_175918:exon1:c.T1081G:p.C361G	NA	NA	D
chr4	1813925	1813925	-	A	UTR3	LETM1	NA	NA	NA	NA	NA
chr4	1814119	1814119	T	G	UTR3	LETM1	NA	NA	NA	NA	NA
chr4	2233250	2233250	C	T	UTR3	HAUS3	NA	NA	NA	rs183516458	NA
chr4	2252201	2252201	C	T	UTR3	MXD4	NA	NA	NA	rs1052682	NA
chr4	3240568	3240568	C	A	exonic	HTT	synonymous SNV	HTT:NM_002111:exon66:c.C9078A:p.T3026T	NA	NA	NA
chr4	3475252	3475252	T	C	exonic	DOK7	synonymous SNV	M_001164673:exon3:c.T220C:p.L74L,DOK7:NM_173660:exon3:c.T220	NA	rs4325970	NA
chr4	4190576	4190576	C	G	exonic	OTOP1	nonsynonymous SNV	OTOP1:NM_177998:exon6:c.G1793C:p.R598P	NA	rs199742451	D
chr4	4190577	4190577	G	C	exonic	OTOP1	nonsynonymous SNV	OTOP1:NM_177998:exon6:c.C1792G:p.R598G	NA	rs200368405	D
chr4	4190595	4190595	G	C	exonic	OTOP1	nonsynonymous SNV	OTOP1:NM_177998:exon6:c.C1774G:p.P592A	NA	rs2310687	D
chr4	5814052	5814052	T	C	UTR3	EVC	NA	NA	NA	NA	NA
chr4	7044357	7044357	A	G	exonic	CCDC96	synonymous SNV	CCDC96:NM_153376:exon1:c.T309C:p.V103V	NA	rs871133	NA
chr4	8583312	8583312	A	C	exonic	GPR78	nonsynonymous SNV	GPR78:NM_080819:exon1:c.A603C:p.R201S	NA	rs17844778	B
chr4	8869257	8869257	A	G	UTR3	HMX1	NA	NA	NA	rs13303067	NA
chr4	10515142	10515142	C	T	exonic	CLNK	synonymous SNV	CLNK:NM_052964:exon16:c.G852A:p.L284L	NA	rs114623663	NA
chr4	11400431	11400431	T	-	UTR3	HS3ST1	NA	NA	NA	NA	NA
chr4	13335614	13335614	A	G	intergenic	1(dist=1905077),RAB28(dist=	NA	NA	NA	rs150340205	NA
chr4	17634213	17634213	A	G	exonic	FAM184B	nonsynonymous SNV	FAM184B:NM_015688:exon18:c.T3125C:p.V1042A	NA	rs6825562	NA
chr4	20731089	20731089	-	AC	UTR3	KCNIP4	NA	NA	NA	NA	NA
chr4	20731092	20731092	-	TTTAAATGT	UTR3	KCNIP4	NA	NA	NA	NA	NA
chr4	20731213	20731213	-	TTTTAATCT	UTR3	KCNIP4	NA	NA	NA	NA	NA
chr4	20731232	20731242	AATGGAA	-	UTR3	KCNIP4	NA	NA	NA	rs151224175	NA
chr4	20852244	20852244	A	G	exonic	KCNIP4	synonymous SNV	4:NM_001035004:exon2:c.T24C:p.P8P,KCNIP4:NM_025221:exon2:c.T1	NA	rs3765122	NA
chr4	21850576	21850576	-	GTGTGTGC	ncRNA_exonic	KCNIP4-IT1	NA	NA	NA	rs145269724	NA
chr4	26585881	26585881	C	T	exonic	TBC1D19	synonymous SNV	TBC1D19:NM_018317:exon1:c.C66T:p.S22S	NA	rs4692549	NA
chr4	31147973	31147980	AAATAAAT	-	UTR3	PCDH7	NA	NA	NA	rs145102344	NA
chr4	37593975	37593975	-	A	UTR3	C4orf19	NA	NA	NA	NA	NA
chr4	37613930	37613930	G	A	UTR3	RELL1	NA	NA	NA	rs17495047	NA
chr4	39450940	39450940	-	A	UTR3	KLB	NA	NA	NA	rs35648143	NA
chr4	39451560	39451565	AATAAA	-	UTR3	KLB	NA	NA	NA	rs145759953	NA
chr4	39458051	39458051	A	G	exonic	RPL9	synonymous SNV	_001024921:exon4:c.T366C:p.Y122Y,RPL9:NM_000661:exon5:c.T366C	NA	rs1065744	NA
chr4	39478832	39478832	G	A	UTR3	LIAS	NA	NA	NA	rs1048140	NA

chr4	39501723	39501723	A	-	UTR3	UGDH	NA	NA	NA	NA	NA
chr4	39529146	39529146	T	C	UTR5	UGDH	NA	NA	NA	rs12642711	NA
chr4	40044556	40044556	G	T	ncRNA_exonic	LOC344967	NA	NA	NA	NA	NA
chr4	40438576	40438576	A	G	exonic	RBM47	synonymous SNV	RBM47:NM_001098634:exon5:c.T1212C:p.G404G	NA	rs2307046	NA
chr4	40812639	40812639	C	A	UTR3	APBB2	NA	NA	NA	NA	NA
chr4	41746101	41746101	G	A	UTR3	PHOX2B	NA	NA	NA	NA	NA
chr4	41746127	41746127	C	A	UTR3	PHOX2B	NA	NA	NA	NA	NA
chr4	43032612	43032612	G	A	UTR3	GRXCR1	NA	NA	NA	rs4861050	NA
chr4	44701750	44701750	A	C	UTR3	GUF1	NA	NA	NA	rs6812211	NA
chr4	44701764	44701764	T	G	UTR3	GUF1	NA	NA	NA	rs6817397	NA
chr4	47428446	47428447	AA	-	UTR3	GABRB1	NA	NA	NA	NA	NA
chr4	48038716	48038716	G	A	UTR3	NIPAL1	NA	NA	NA	rs11722463	NA
chr4	48500089	48500089	T	-	UTR3	FRYL	NA	NA	NA	rs59265109	NA
chr4	52780989	52780989	-	T	UTR3	DCUN1D4	NA	NA	NA	NA	NA
chr4	53739938	53739938	T	-	UTR3	SCFD2	NA	NA	NA	NA	NA
chr4	54876132	54876132	C	-	UTR3	CHIC2	NA	NA	NA	rs68107873	NA
chr4	56899013	56899013	-	TGTGTGTG	UTR3	CEP135	NA	NA	NA	NA	NA
chr4	56899377	56899377	A	G	UTR3	CEP135	NA	NA	NA	rs12512313	NA
chr4	57220242	57220242	C	A	exonic	AASDH	nonsynonymous SNV	AASDH:NM_181806:exon8:c.G1346T:p.R449L	NA	NA	D
chr4	57326996	57326996	C	T	UTR3	PAICS	NA	NA	NA	rs1140982	NA
chr4	57371391	57371391	A	T	UTR5	ARL9	NA	NA	NA	rs17086919	NA
chr4	68483772	68483772	C	A	UTR3	UBA6	NA	NA	NA	rs75660867	NA
chr4	69512381	69512381	C	T	UTR3	UGT2B15	NA	NA	NA	NA	NA
chr4	71029602	71029602	T	-	UTR3	C4orf40	NA	NA	NA	rs71210152	NA
chr4	71117016	71117016	T	G	UTR3	CSN3	NA	NA	NA	rs62308392	NA
chr4	72435944	72435973	TTCAGCCTC	-	UTR3	SLC4A4	NA	NA	NA	NA	NA
chr4	75975077	75975077	T	-	UTR3	PARM1	NA	NA	NA	NA	NA
chr4	76720885	76720885	T	C	exonic	USO1	unknown	UNKNOWN	NA	rs13119659	NA
chr4	76871264	76871264	C	A	UTR3	SDAD1	NA	NA	NA	NA	NA
chr4	76871270	76871270	C	A	UTR3	SDAD1	NA	NA	NA	NA	NA
chr4	77662125	77662125	C	T	exonic	SHROOM3	synonymous SNV	SHROOM3:NM_020859:exon5:c.C2799T:p.S933S	NA	rs36036308	NA
chr4	77701688	77701688	A	-	UTR3	SHROOM3	NA	NA	NA	rs11325137	NA
chr4	78635594	78635615	AAAAAAAA	-	UTR3	CNOT6L	NA	NA	NA	NA	NA
chr4	84011704	84011704	G	A	UTR3	PLAC8	NA	NA	NA	NA	NA
chr4	84383373	84383373	C	-	UTR3	FAM175A	NA	NA	NA	rs34610900	NA
chr4	85927783	85927783	A	-	ncRNA_exonic	WDFY3-AS2	NA	NA	NA	rs10715207	NA
chr4	86396465	86396465	T	G	UTR5	ARHGAP24	NA	NA	NA	NA	NA
chr4	88536869	88536886	CAGCAGCA	-	exonic	DSPP	nonframeshift deletion	DSPP:NM_014208:exon5:c.3055_3072del:p.1019_1024del	NA	NA	NA
chr4	88536980	88536980	A	G	exonic	DSPP	nonsynonymous SNV	DSPP:NM_014208:exon5:c.A3166G:p.N1056D	NA	rs201074114	NA
chr4	88537024	88537024	T	C	exonic	DSPP	synonymous SNV	DSPP:NM_014208:exon5:c.T3210C:p.S1070S	NA	NA	NA
chr4	88537123	88537123	C	T	exonic	DSPP	synonymous SNV	DSPP:NM_014208:exon5:c.C3309T:p.S1103S	NA	NA	NA
chr4	88537180	88537180	T	C	exonic	DSPP	synonymous SNV	DSPP:NM_014208:exon5:c.T3366C:p.N1122N	NA	NA	NA
chr4	88537216	88537216	T	C	exonic	DSPP	synonymous SNV	DSPP:NM_014208:exon5:c.T3402C:p.D1134D	NA	NA	NA
chr4	89650211	89650212	TT	-	ncRNA_exonic	FAM13A-AS1	NA	NA	NA	NA	NA
chr4	90645853	90645853	C	T	UTR3	SNCA	NA	NA	NA	NA	NA
chr4	90646469	90646469	-	AAAAA	UTR3	SNCA	NA	NA	NA	NA	NA
chr4	90875762	90875762	A	T	UTR3	MMRN1	NA	NA	NA	rs114018162	NA
chr4	91759710	91759710	-	A	intronic	CCSER1	NA	NA	NA	NA	NA
chr4	96076621	96076621	G	A	UTR3	BMPR1B	NA	NA	NA	NA	NA
chr4	99850448	99850448	A	C	UTR5	EIF4E	NA	NA	NA	NA	NA
chr4	99850451	99850451	T	-	UTR5	EIF4E	NA	NA	NA	NA	NA
chr4	1E+08	1E+08	A	T	exonic	ADH6	nonsynonymous SNV	:NM_000672:exon1:c.T4A:p.C2S,ADH6:NM_001102470:exon1:c.T4A:	NA	rs4699735	B
chr4	1E+08	1E+08	A	G	exonic	ADH1B	synonymous SNV	ADH1B:NM_000668:exon6:c.T753C:p.I251I	NA	rs1789882	NA
chr4	1E+08	1E+08	C	T	exonic	ADH1C	unknown	UNKNOWN	NA	rs1693425	NA
chr4	1E+08	1E+08	-	T	UTR3	TRMT10A	NA	NA	NA	rs149160816	NA
chr4	1.01E+08	1.01E+08	AA	-	UTR3	MTTP	NA	NA	NA	NA	NA
chr4	1.01E+08	1.01E+08	C	T	UTR3	EMCN	NA	NA	NA	NA	NA
chr4	1.02E+08	1.02E+08	-	TT	UTR3	PPP3CA	NA	NA	NA	NA	NA
chr4	1.03E+08	1.03E+08	G	A	UTR3	SLC39A8	NA	NA	NA	rs190178	NA
chr4	1.03E+08	1.03E+08	C	T	UTR3	SLC39A8	NA	NA	NA	rs58321370	NA
chr4	1.04E+08	1.04E+08	C	A	UTR3	NFKB1	NA	NA	NA	NA	NA
chr4	1.04E+08	1.04E+08	T	C	UTR3	CISD2	NA	NA	NA	rs7439174	NA
chr4	1.04E+08	1.04E+08	TG	-	UTR3	CISD2	NA	NA	NA	rs199492696	NA
chr4	1.09E+08	1.09E+08	G	A	ncRNA_exonic	RPL34-AS1	NA	NA	NA	rs74676576	NA
chr4	1.1E+08	1.1E+08	T	G	exonic	COL25A1	synonymous SNV	3C:p.G616G,COL25A1:NM_032518:exon34:c.A1839C:p.G613G,COL25A	NA	rs7689008	NA
chr4	1.14E+08	1.14E+08	C	T	UTR3	CAMK2D	NA	NA	NA	rs12511604	NA

chr4	1.14E+08	1.14E+08	-	A	UTR3	CAMK2D	NA	NA	NA	NA	NA
chr4	1.2E+08	1.2E+08	AAA	-	UTR5	USP53	NA	NA	NA	NA	NA
chr4	1.22E+08	1.22E+08	T	-	UTR3	TNIP3	NA	NA	NA	NA	NA
chr4	1.22E+08	1.22E+08	T	G	UTR3	TNIP3	NA	NA	NA	NA	NA
chr4	1.22E+08	1.22E+08	ACACACAC	-	UTR3	TNIP3	NA	NA	NA	rs10561132	NA
chr4	1.23E+08	1.23E+08	T	C	UTR3	TMEM155	NA	NA	NA	rs4342205	NA
chr4	1.23E+08	1.23E+08	G	C	ncRNA_exonic	PP12613	NA	NA	NA	rs4833772	NA
chr4	1.23E+08	1.23E+08	G	C	UTR5	BBS7	NA	NA	NA	rs2271176	NA
chr4	1.24E+08	1.24E+08	T	-	UTR3	SPATA5	NA	NA	NA	rs11306876	NA
chr4	1.24E+08	1.24E+08	G	A	UTR3	SPATA5	NA	NA	NA	rs2132078	NA
chr4	1.29E+08	1.29E+08	G	T	UTR5	HSPA4L	NA	NA	NA	NA	NA
chr4	1.4E+08	1.4E+08	TA	-	UTR3	ELF2	NA	NA	NA	rs113800639	NA
chr4	1.41E+08	1.41E+08	C	T	exonic	MAML3	synonymous SNV	MAML3:NM_018717:exon2:c.G1479A:p.Q493Q	NA	rs62344937	NA
chr4	1.43E+08	1.43E+08	G	A	exonic	INPP4B	synonymous SNV	_001101669:exon7:c.C369T:p.D123D,INPP4B:NM_003866:exon8:c.C3	=COSM149743;OCCURENCE=1(stomac	rs1982966	NA
chr4	1.46E+08	1.46E+08	C	A	UTR3	SMAD1	NA	NA	NA	rs62343509	NA
chr4	1.47E+08	1.47E+08	A	-	UTR3	MMAA	NA	NA	NA	rs33978754	NA
chr4	1.51E+08	1.51E+08	C	T	exonic	DCLK2	nonsynonymous SNV	1040260:exon16:c.C2242T:p.P748S,DCLK2:NM_001040261:exon17:c.C	NA	rs13152819	NA
chr4	1.51E+08	1.51E+08	C	G	exonic	DCLK2	nonsynonymous SNV	.040260:exon16:c.C2243G:p.P748R,DCLK2:NM_001040261:exon17:c.C	NA	rs60939896	NA
chr4	1.51E+08	1.51E+08	C	G	UTR3	DCLK2	NA	NA	NA	rs13101461	NA
chr4	1.52E+08	1.52E+08	C	-	UTR3	MAB21L2	NA	NA	NA	rs67191302	NA
chr4	1.52E+08	1.52E+08	TCT	-	UTR5	FAM160A1	NA	NA	NA	rs201341310	NA
chr4	1.54E+08	1.54E+08	T	C	exonic	FHDC1	nonsynonymous SNV	FHDC1:NM_033393:exon11:c.T1579C:p.S527P	NA	NA	B
chr4	1.54E+08	1.54E+08	A	C	exonic	FHDC1	nonsynonymous SNV	FHDC1:NM_033393:exon11:c.A1606C:p.T536P	NA	NA	P
chr4	1.54E+08	1.54E+08	T	G	exonic	TRIM2	synonymous SNV	_001130067:exon3:c.T324G:p.T108T,TRIM2:NM_015271:exon3:c.T40!	NA	rs2289409	NA
chr4	1.54E+08	1.54E+08	-	T	UTR3	TRIM2	NA	NA	NA	rs71598263	NA
chr4	1.56E+08	1.56E+08	A	G	UTR3	LRAT	NA	NA	NA	rs77071498	NA
chr4	1.56E+08	1.56E+08	T	-	UTR3	RBM46	NA	NA	NA	rs34773010	NA
chr4	1.56E+08	1.56E+08	A	G	UTR5	MAP9	NA	NA	ID=COSN404819;OCCURENCE=1(lung)	rs17377679	NA
chr4	1.57E+08	1.57E+08	-	A	UTR3	GUCY1A3	NA	NA	NA	NA	NA
chr4	1.58E+08	1.58E+08	TG	-	UTR5	GRIA2	NA	NA	NA	rs71934280	NA
chr4	1.6E+08	1.6E+08	C	T	UTR5	PPID	NA	NA	NA	rs2070629	NA
chr4	1.6E+08	1.6E+08	A	-	UTR3	FNIP2	NA	NA	NA	rs11340968	NA
chr4	1.66E+08	1.66E+08	T	C	UTR3	TRIM61	NA	NA	NA	rs6834073	NA
chr4	1.66E+08	1.66E+08	T	-	UTR3	TMEM192	NA	NA	NA	NA	NA
chr4	1.67E+08	1.67E+08	-	AA	UTR3	TLL1	NA	NA	NA	NA	NA
chr4	1.69E+08	1.69E+08	T	C	exonic	DDX60L	nonsynonymous SNV	DDX60L:NM_001012967:exon37:c.A4936G:p.M1646V	NA	rs2684348	NA
chr4	1.7E+08	1.7E+08	G	T	exonic	CBR4	nonsynonymous SNV	CBR4:NM_032783:exon2:c.C208A:p.L70M	NA	rs2877380	B
chr4	1.7E+08	1.7E+08	A	G	exonic	SH3RF1	nonsynonymous SNV	SH3RF1:NM_020870:exon5:c.T943C:p.S315P	NA	NA	P
chr4	1.76E+08	1.76E+08	G	T	UTR5	ADAM29	NA	NA	NA	rs7689299	NA
chr4	1.76E+08	1.76E+08	C	T	UTR5	ADAM29	NA	NA	NA	rs62334419	NA
chr4	1.77E+08	1.77E+08	A	G	exonic	SPATA4	synonymous SNV	SPATA4:NM_144644:exon4:c.T510C:p.Y170Y	NA	rs2291244	NA
chr4	1.77E+08	1.77E+08	T	C	UTR5	SPATA4	NA	NA	NA	rs4565028	NA
chr4	1.77E+08	1.77E+08	T	-	UTR3	SPCS3	NA	NA	NA	rs67194984	NA
chr4	1.84E+08	1.84E+08	AAA	-	ncRNA_exonic	FAM92A1P2	NA	NA	NA	NA	NA
chr4	1.84E+08	1.84E+08	G	T	exonic	WWC2	nonsynonymous SNV	WWC2:NM_024949:exon16:c.G2446T:p.V816F	NA	rs11734376	NA
chr4	1.86E+08	1.86E+08	-	ATT	UTR3	CASP3	NA	NA	NA	rs4647696	NA
chr4	1.86E+08	1.86E+08	G	A	exonic	CCDC111	synonymous SNV	CCDC111:NM_152683:exon8:c.G948A:p.Q316Q	NA	rs34985821	NA
chr4	1.86E+08	1.86E+08	T	A	UTR3	LRP2BP	NA	NA	NA	rs28723682	NA
chr4	1.87E+08	1.87E+08	T	C	UTR3	CYP4V2	NA	NA	NA	rs62348764	NA
chr4	1.91E+08	1.91E+08	A	G	exonic	FRG1	nonsynonymous SNV	FRG1:NM_004477:exon3:c.A196G:p.K66E	ID=COSM232360;OCCURENCE=1(skin)	rs112612436	B
chr4	1.91E+08	1.91E+08	C	T	exonic	FRG1	nonsynonymous SNV	FRG1:NM_004477:exon4:c.C293T:p.T98M	NA	rs201275486	B
chr4	1.91E+08	1.91E+08	G	A	exonic	FRG1	nonsynonymous SNV	FRG1:NM_004477:exon5:c.G383A:p.R128H	OSM1054421;OCCURENCE=1(endometi	rs75585360	P
chr4	1.91E+08	1.91E+08	A	G	exonic	FRG1	nonsynonymous SNV	FRG1:NM_004477:exon7:c.A568G:p.K190E	NA	rs184307882	B
chr4	1.91E+08	1.91E+08	G	A	exonic	FRG1	nonsynonymous SNV	FRG1:NM_004477:exon7:c.G604A:p.V202I	NA	rs6846627	B
chr4	1.91E+08	1.91E+08	T	C	exonic	FRG1	synonymous SNV	FRG1:NM_004477:exon7:c.T627C:p.Y209Y	NA	rs75112782	NA
chr5	185498	185498	C	T	UTR3	PLEKHG4B	NA	NA	NA	rs7727015	NA
chr5	661392	661392	G	T	UTR3	TPPP	NA	NA	NA	rs13159892	NA
chr5	796054	796055	CT	-	UTR3	ZDHHC11	NA	NA	NA	NA	NA
chr5	843815	843815	C	T	exonic	ZDHHC11	synonymous SNV	ZDHHC11:NM_024786:exon4:c.G528A:p.S176S	NA	rs71591190	NA
chr5	1222213	1222214	TG	-	UTR3	SLC6A19	NA	NA	NA	rs35482871	NA
chr5	1572289	1572289	A	G	ncRNA_exonic	SDHAP3	NA	NA	NA	rs147001297	NA
chr5	6311304	6311304	T	G	ncRNA_exonic	FLJ33360	NA	NA	NA	rs56073709	NA
chr5	9036071	9036071	-	A	UTR3	SEMA5A	NA	NA	NA	NA	NA
chr5	10278910	10278910	C	A	UTR3	CMBL	NA	NA	NA	NA	NA
chr5	10279657	10279658	TT	-	UTR3	CMBL	NA	NA	NA	rs140959227	NA
chr5	10657091	10657091	-	AA	UTR3	ANKRD33B	NA	NA	NA	NA	NA

chr5	10972889	10972889	T	-	UTR3	CTNND2	NA	NA	NA	rs70947237	NA
chr5	13701536	13701536	T	C	exonic	DNAH5	nonsynonymous SNV	DNAH5:NM_001369:exon77:c.A13348G:p.I4450V	NA	rs3734110	B
chr5	14696103	14696104	TT	-	UTR3	FAM105B	NA	NA	NA	NA	NA
chr5	16508898	16508898	T	C	UTR5	FAM134B	NA	NA	NA	rs33684	NA
chr5	16662426	16662432	TTTTTTT	-	UTR3	MYO10	NA	NA	NA	NA	NA
chr5	17217178	17217178	-	GT	ncRNA_exonic	LOC285696	NA	NA	NA	NA	NA
chr5	21586917	21586917	-	AA	ncRNA_exonic	GUSBP1	NA	NA	NA	NA	NA
chr5	23526971	23526971	C	A	exonic	PRDM9	nonsynonymous SNV	PRDM9:NM_020227:exon11:c.C1774A:p.Q592K	NA	NA	B
chr5	31317952	31317952	T	C	exonic	CDH6	synonymous SNV	CDH6:NM_004932:exon11:c.T1803C:p.H601H	NA	rs2229575	NA
chr5	33938092	33938092	G	C	exonic	RXFP3	nonsynonymous SNV	RXFP3:NM_016568:exon1:c.G1247C:p.R416P	NA	NA	D
chr5	34908276	34908276	-	TT	UTR3	RAD1	NA	NA	NA	NA	NA
chr5	34956158	34956158	A	-	UTR3	DNAJC21	NA	NA	NA	NA	NA
chr5	35644621	35644621	T	C	exonic	SPEF2	synonymous SNV	NM_024867:exon4:c.T579C:p.I193I,SPEF2:NM_144722:exon4:c.T579C:	NA	rs7706444	NA
chr5	36242212	36242212	T	C	UTR5	NADKD1	NA	NA	NA	rs67061065	NA
chr5	40692116	40692116	G	A	exonic	PTGER4	nonsynonymous SNV	PTGER4:NM_000958:exon3:c.G1103A:p.R368K	NA	NA	P
chr5	40964885	40964885	A	T	exonic	C7	nonsynonymous SNV	C7:NM_000587:exon14:c.A1792T:p.T598S	NA	rs60714178	NA
chr5	42756940	42756940	C	T	UTR5	CCDC152	NA	NA	NA	rs3797310	NA
chr5	42801949	42801949	A	G	UTR3	CCDC152	NA	NA	NA	rs230816	NA
chr5	42802310	42802310	A	G	UTR3	CCDC152	NA	NA	NA	rs28919916	NA
chr5	52193287	52193287	C	T	exonic	ITGA1	synonymous SNV	ITGA1:NM_181501:exon10:c.C1092T:p.A364A	)=COSM149830;OCCURENCE=1(stomacl	rs1531545	NA
chr5	54274656	54274656	-	A	UTR3	ESM1	NA	NA	NA	rs35235179	NA
chr5	55206444	55206444	G	A	exonic	IL31RA	nonsynonymous SNV	5A:p.S529N,IL31RA:NM_139017:exon12:c.G1586A:p.S529N,IL31RA:NM	NA	rs161704	P
chr5	59825300	59825300	T	C	ncRNA_exonic	PART1	NA	NA	NA	rs26949	NA
chr5	59842971	59842971	A	G	ncRNA_exonic	PART1	NA	NA	NA	rs153152	NA
chr5	61000547	61000548	TT	-	UTR3	C5orf64	NA	NA	NA	rs140973829	NA
chr5	61874567	61874567	G	T	ncRNA_exonic	IPO11-LRRC70	NA	NA	NA	NA	NA
chr5	61874620	61874620	A	-	ncRNA_exonic	IPO11-LRRC70	NA	NA	NA	NA	NA
chr5	63668039	63668039	A	G	UTR3	RNF180	NA	NA	NA	rs3797052	NA
chr5	64920159	64920159	T	G	UTR5	TRIM23	NA	NA	NA	NA	NA
chr5	64963082	64963082	-	CT	UTR3	SGTB	NA	NA	NA	rs2067306	NA
chr5	64963466	64963469	TTTG	-	UTR3	SGTB	NA	NA	NA	rs33916729	NA
chr5	66492591	66492591	G	A	UTR5	CD180	NA	NA	NA	NA	NA
chr5	68739836	68739836	-	AAAAAAAA	intergenic	'ELD2(dist=1946),OCLN(dist=4	NA	NA	NA	NA	NA
chr5	69723444	69723444	A	G	ncRNA_exonic	GTF2H2B	NA	NA	NA	rs162961	NA
chr5	70672628	70672629	TT	-	ncRNA_exonic	PMCHL2	NA	NA	NA	NA	NA
chr5	70673207	70673207	A	-	ncRNA_exonic	PMCHL2	NA	NA	NA	NA	NA
chr5	70800475	70800475	C	T	exonic	BDP1	nonsynonymous SNV	BDP1:NM_018429:exon16:c.C2269T:p.R757C	NA	rs3761966	NA
chr5	70883136	70883136	A	G	UTR5	MCCC2	NA	NA	NA	rs11746722	NA
chr5	70952934	70952934	C	T	UTR3	MCCC2	NA	NA	NA	rs1135667	NA
chr5	72876880	72876880	T	-	UTR3	UTP15	NA	NA	NA	NA	NA
chr5	74506658	74506658	C	T	exonic	ANKRD31	synonymous SNV	ANKRD31:NM_001164443:exon4:c.G321A:p.L107L	11137379,COSM1137380;OCCURENCE=	rs2219745	NA
chr5	75001582	75001582	A	G	exonic	POC5	nonsynonymous SNV	M_152408:exon3:c.T179C:p.I60T,POC5:NM_001099271:exon4:c.T254	NA	rs17672542	NA
chr5	75379321	75379321	G	C	UTR5	SV2C	NA	NA	NA	rs30199	NA
chr5	75948650	75948650	A	G	exonic	IQGAP2	nonsynonymous SNV	IQGAP2:NM_006633:exon18:c.A2170G:p.I724V	NA	rs2431363	B
chr5	76003258	76003258	C	T	UTR3	IQGAP2	NA	NA	NA	rs464494	NA
chr5	76007997	76007998	AA	-	upstream	NCRUPAR	NA	NA	NA	NA	NA
chr5	76129965	76129965	-	ATAT	UTR3	F2RL1	NA	NA	NA	rs35226539	NA
chr5	78181577	78181577	T	C	exonic	ARSB	synonymous SNV	/_000046:exon5:c.A972G:p.G324G,ARSB:NM_198709:exon6:c.A972G:	NA	rs72762973	NA
chr5	78365401	78365401	G	A	exonic	DMGDH	nonsynonymous SNV	DMGDH:NM_013391:exon1:c.C43T:p.R15W	NA	NA	P
chr5	79048562	79048562	C	T	exonic	CMYA5	synonymous SNV	CMYA5:NM_153610:exon6:c.C11055T:p.F3685F	)=COSM149872;OCCURENCE=1(stomacl	rs1366272	NA
chr5	82936729	82936729	T	-	UTR3	HAPLN1	NA	NA	NA	NA	NA
chr5	83239133	83239133	A	-	UTR3	EDIL3	NA	NA	NA	rs146077789	NA
chr5	87980581	87980581	-	TCTC	ncRNA_exonic	LINC00461	NA	NA	NA	NA	NA
chr5	94786142	94786142	C	T	UTR3	FAM81B	NA	NA	NA	rs7709828	NA
chr5	95115959	95115959	C	T	exonic	RHOBTB3	nonsynonymous SNV	RHOBTB3:NM_014899:exon9:c.C1286T:p.T429M	NA	rs41276257	P
chr5	96111093	96111096	TAGT	-	UTR3	ERAP1	NA	NA	NA	rs138961474	NA
chr5	96111228	96111228	A	G	UTR3	ERAP1	NA	NA	NA	rs3198304	NA
chr5	96111801	96111801	-	TG	UTR3	ERAP1	NA	NA	NA	rs148692674	NA
chr5	96111927	96111927	-	:CATTCATG.	UTR3	ERAP1	NA	NA	NA	rs142866043	NA
chr5	96143836	96143836	G	A	UTR5	ERAP1	NA	NA	NA	NA	NA
chr5	96143837	96143837	T	G	UTR5	ERAP1	NA	NA	NA	NA	NA
chr5	96143838	96143838	T	A	UTR5	ERAP1	NA	NA	NA	NA	NA
chr5	96245439	96245439	C	T	exonic	ERAP2	synonymous SNV	)01130140:exon15:c.C2325T:p.S775S,ERAP2:NM_022350:exon15:c.C2:	NA	rs1056893	NA
chr5	96322360	96322360	G	A	exonic	LNPEP	nonsynonymous SNV	/_005575:exon4:c.G1117A:p.V373I,LNPEP:NM_175920:exon4:c.G1075	NA	rs41276279	B
chr5	98132052	98132052	A	C	UTR3	RGMB	NA	NA	NA	rs1053451	NA
chr5	98218907	98218907	C	A	exonic	CHD1	nonsynonymous SNV	CHD1:NM_001270:exon18:c.G2603T:p.G868V	NA	NA	D

chr5	1.03E+08	1.03E+08	A	G	exonic	NUDT12	synonymous SNV	NUDT12:NM_031438:exon4:c.T901C:p.L301L	NA	rs10045774	NA
chr5	1.07E+08	1.07E+08	-	T	UTR3	EFNA5	NA	NA	NA	NA	NA
chr5	1.07E+08	1.07E+08	-	AA	UTR3	EFNA5	NA	NA	NA	NA	NA
chr5	1.07E+08	1.07E+08	G	A	UTR3	EFNA5	NA	NA	NA	NA	NA
chr5	1.07E+08	1.07E+08	A	C	UTR3	FBXL17	NA	NA	NA	NA	NA
chr5	1.1E+08	1.1E+08	T	-	UTR3	WDR36	NA	NA	NA	NA	NA
chr5	1.1E+08	1.1E+08	T	G	UTR3	WDR36	NA	NA	NA	NA	NA
chr5	1.15E+08	1.15E+08	AAAA	-	UTR3	PGGT1B	NA	NA	NA	NA	NA
chr5	1.15E+08	1.15E+08	C	A	UTR3	TMED7	NA	NA	NA	NA	NA
chr5	1.15E+08	1.15E+08	G	A	UTR3	AP3S1	NA	NA	NA	rs6895932	NA
chr5	1.15E+08	1.15E+08	G	C	UTR5	AQPEP	NA	NA	ID=COSN404863;OCCURENCE=1(lung)	rs9326980	NA
chr5	1.15E+08	1.15E+08	A	G	exonic	AQPEP	synonymous SNV	AQPEP:NM_173800:exon1:c.A204G:p.P68P	NA	rs12522632	NA
chr5	1.16E+08	1.16E+08	T	G	UTR3	SEMA6A	NA	NA	NA	rs12523054	NA
chr5	1.22E+08	1.22E+08	CACACACA	-	UTR3	SNX2	NA	NA	NA	rs138443824	NA
chr5	1.22E+08	1.22E+08	-	A	UTR3	SNX24	NA	NA	NA	NA	NA
chr5	1.22E+08	1.22E+08	A	G	UTR5	PRDM6	NA	NA	NA	rs72800825	NA
chr5	1.26E+08	1.26E+08	A	G	UTR3	LMNB1	NA	NA	NA	NA	NA
chr5	1.26E+08	1.26E+08	C	T	UTR3	3-Mar	NA	NA	NA	NA	NA
chr5	1.28E+08	1.28E+08	-	T	UTR3	SLC12A2	NA	NA	NA	NA	NA
chr5	1.28E+08	1.28E+08	G	C	exonic	FBN2	synonymous SNV	FBN2:NM_001999:exon1:c.C183G:p.P61P	NA	rs73348287	NA
chr5	1.31E+08	1.31E+08	C	T	UTR3	RAPGEF6	NA	NA	NA	NA	NA
chr5	1.31E+08	1.31E+08	T	-	UTR3	RAPGEF6	NA	NA	NA	NA	NA
chr5	1.31E+08	1.31E+08	G	A	UTR3	RAPGEF6	NA	NA	NA	NA	NA
chr5	1.32E+08	1.32E+08	C	T	exonic	SLC22A4	nonsynonymous SNV	SLC22A4:NM_003059:exon9:c.C1507T:p.L503F	NA	rs1050152	B
chr5	1.32E+08	1.32E+08	A	G	UTR5	RAD50	NA	NA	NA	NA	NA
chr5	1.32E+08	1.32E+08	C	T	UTR3	CCNI2	NA	NA	NA	NA	NA
chr5	1.32E+08	1.32E+08	TTT	-	UTR3	CCNI2	NA	NA	NA	NA	NA
chr5	1.34E+08	1.34E+08	AAAT	-	UTR3	C5orf24	NA	NA	NA	rs202226698	NA
chr5	1.35E+08	1.35E+08	TTTTTTTTTT	-	UTR3	CXCL14	NA	NA	NA	NA	NA
chr5	1.35E+08	1.35E+08	GGG	-	ncRNA_exonic	SMAD5-AS1	NA	NA	NA	NA	NA
chr5	1.36E+08	1.36E+08	-	AA	UTR3	SMAD5	NA	NA	NA	NA	NA
chr5	1.37E+08	1.37E+08	A	-	UTR3	HNRNPA0	NA	NA	NA	NA	NA
chr5	1.37E+08	1.37E+08	G	A	exonic	PKD2L2	nonsynonymous SNV	001258448:exon8:c.G1144A:p.V382I,PKD2L2:NM_014386:exon8:c.G1	NA	rs1880458	B
chr5	1.38E+08	1.38E+08	G	C	exonic	EGR1	synonymous SNV	EGR1:NM_001964:exon2:c.G993C:p.T331T	NA	NA	NA
chr5	1.39E+08	1.39E+08	-	T	UTR5	MATR3	NA	NA	NA	rs112107315	NA
chr5	1.39E+08	1.39E+08	T	A	UTR5	MATR3	NA	NA	NA	rs12153162	NA
chr5	1.39E+08	1.39E+08	A	-	UTR3	PURA	NA	NA	NA	NA	NA
chr5	1.4E+08	1.4E+08	A	C	UTR3	WDR55	NA	NA	NA	NA	NA
chr5	1.41E+08	1.41E+08	A	C	UTR3	PCDHB6	NA	NA	NA	rs56172324	NA
chr5	1.41E+08	1.41E+08	-	ACACAC	UTR5	PCDHB16	NA	NA	NA	NA	NA
chr5	1.41E+08	1.41E+08	A	G	exonic	PCDHB16	synonymous SNV	PCDHB16:NM_020957:exon1:c.A1524G:p.A508A	NA	rs2697533	NA
chr5	1.41E+08	1.41E+08	G	T	UTR3	PCDHB9	NA	NA	NA	NA	NA
chr5	1.41E+08	1.41E+08	A	G	UTR3	PCDHB9	NA	NA	NA	rs2910324	NA
chr5	1.41E+08	1.41E+08	C	T	UTR3	PCDHB9	NA	NA	NA	rs2907325	NA
chr5	1.41E+08	1.41E+08	-	A	UTR3	PCDHB9	NA	NA	NA	NA	NA
chr5	1.44E+08	1.44E+08	-	T	UTR3	YIPF5	NA	NA	NA	NA	NA
chr5	1.44E+08	1.44E+08	-	TT	UTR3	YIPF5	NA	NA	NA	rs138694329	NA
chr5	1.44E+08	1.44E+08	T	-	UTR3	YIPF5	NA	NA	NA	rs144356469	NA
chr5	1.45E+08	1.45E+08	C	A	UTR3	LARS	NA	NA	NA	rs58569901	NA
chr5	1.46E+08	1.46E+08	G	A	exonic	LARS	synonymous SNV	LARS:NM_020117:exon28:c.C2889T:p.N963N	NA	rs11540216	NA
chr5	1.46E+08	1.46E+08	G	A	exonic	LARS	synonymous SNV	LARS:NM_020117:exon19:c.C1747T:p.L583L	NA	rs11540217	NA
chr5	1.46E+08	1.46E+08	T	-	UTR3	RBM27	NA	NA	NA	NA	NA
chr5	1.46E+08	1.46E+08	A	G	exonic	TCERG1	synonymous SNV	1040006:exon21:c.A3057G:p.S1019S,TCERG1:NM_006706:exon22:c.A	NA	rs4705103	NA
chr5	1.48E+08	1.48E+08	TTTT	-	UTR3	SH3TC2	NA	NA	NA	NA	NA
chr5	1.48E+08	1.48E+08	G	A	exonic	SH3TC2	nonsynonymous SNV	SH3TC2:NM_024577:exon14:c.C3239T:p.P1080L	NA	NA	B
chr5	1.49E+08	1.49E+08	G	C	ncRNA_exonic	MIR143HG	NA	NA	NA	rs28418464	NA
chr5	1.49E+08	1.49E+08	-	T	ncRNA_exonic	MIR143HG	NA	NA	NA	NA	NA
chr5	1.49E+08	1.49E+08	A	G	exonic	ARHGEF37	nonsynonymous SNV	ARHGEF37:NM_001001669:exon12:c.A1810G:p.M604V	NA	rs1135093	B
chr5	1.49E+08	1.49E+08	T	C	UTR3	PDE6A	NA	NA	NA	rs759816	NA
chr5	1.49E+08	1.49E+08	A	G	UTR3	PDE6A	NA	NA	NA	rs2005909	NA
chr5	1.5E+08	1.5E+08	TTA	-	UTR3	CAMK2A	NA	NA	NA	rs147490193	NA
chr5	1.5E+08	1.5E+08	T	G	UTR3	SYNPO	NA	NA	NA	NA	NA
chr5	1.5E+08	1.5E+08	C	A	UTR5	SMIM3	NA	NA	NA	NA	NA
chr5	1.51E+08	1.51E+08	A	G	UTR5	GM2A	NA	NA	NA	rs2277028	NA
chr5	1.51E+08	1.51E+08	A	G	exonic	GM2A	nonsynonymous SNV	M_000405:exon2:c.A175G:p.I59V,GM2A:NM_001167607:exon2:c.A17	NA	rs153477	B
chr5	1.51E+08	1.51E+08	A	G	exonic	GM2A	nonsynonymous SNV	_000405:exon2:c.A205G:p.M69V,GM2A:NM_001167607:exon2:c.A20	NA	rs153478	B

chr5	1.51E+08	1.51E+08	-	T	UTR3	GM2A	NA	NA	NA	rs113437403	NA
chr5	1.51E+08	1.51E+08	-	A	UTR3	SLC36A2	NA	NA	NA	NA	NA
chr5	1.51E+08	1.51E+08	G	A	exonic	SLC36A2	synonymous SNV	SLC36A2:NM_181776:exon2:c.C187T:p.L63L	NA	rs192192	NA
chr5	1.51E+08	1.51E+08	GCA	-	UTR5	SLC36A1	NA	NA	NA	NA	NA
chr5	1.51E+08	1.51E+08	T	-	UTR3	G3BP1	NA	NA	NA	NA	NA
chr5	1.51E+08	1.51E+08	G	A	UTR5	GLRA1	NA	NA	NA	rs2071221	NA
chr5	1.54E+08	1.54E+08	G	A	UTR5	HAND1	NA	NA	NA	rs181499341	NA
chr5	1.54E+08	1.54E+08	C	T	UTR3	FAXDC2	NA	NA	NA	rs4865	NA
chr5	1.54E+08	1.54E+08	C	T	UTR3	MRPL22	NA	NA	NA	rs286595	NA
chr5	1.57E+08	1.57E+08	A	-	UTR3	CYFIP2	NA	NA	NA	NA	NA
chr5	1.57E+08	1.57E+08	T	C	UTR5	THG1L	NA	NA	NA	rs2270820	NA
chr5	1.57E+08	1.57E+08	G	T	exonic	THG1L	stopgain SNV	THG1L:NM_017872:exon3:c.G436T:p.E146X	NA	rs78234336	NA
chr5	1.58E+08	1.58E+08	T	-	UTR3	EBF1	NA	NA	NA	NA	NA
chr5	1.59E+08	1.59E+08	A	-	UTR5	EBF1	NA	NA	NA	rs5872586	NA
chr5	1.59E+08	1.59E+08	CACA	-	UTR3	UBLCP1	NA	NA	NA	NA	NA
chr5	1.6E+08	1.6E+08	-	A	UTR3	PWWP2A	NA	NA	NA	NA	NA
chr5	1.6E+08	1.6E+08	C	T	exonic	FABP6	nonsynonymous SNV	:p.T79M,FABP6:NM_001040442:exon4:c.C383T:p.T128M,FABP6:NM_(	NA	rs1130435	B
chr5	1.61E+08	1.61E+08	C	T	UTR3	GABRB2	NA	NA	NA	NA	NA
chr5	1.61E+08	1.61E+08	G	A	UTR3	GABRB2	NA	NA	NA	NA	NA
chr5	1.63E+08	1.63E+08	C	G	UTR5	CCNG1	NA	NA	NA	rs2069339	NA
chr5	1.7E+08	1.7E+08	C	T	exonic	LCP2	synonymous SNV	LCP2:NM_005565:exon8:c.G564A:p.V188V	NA	rs2292254	NA
chr5	1.71E+08	1.71E+08	T	-	UTR3	STK10	NA	NA	NA	rs11353357	NA
chr5	1.72E+08	1.72E+08	-	A	UTR3	ERGIC1	NA	NA	NA	NA	NA
chr5	1.73E+08	1.73E+08	-	T	UTR3	CPEB4	NA	NA	NA	NA	NA
chr5	1.75E+08	1.75E+08	A	-	UTR5	HRH2	NA	NA	NA	NA	NA
chr5	1.75E+08	1.75E+08	G	A	exonic	THOC3	synonymous SNV	THOC3:NM_032361:exon2:c.C357T:p.S119S	NA	rs200408233	NA
chr5	1.77E+08	1.77E+08	AAAAAAAA	-	UTR3	DBN1	NA	NA	NA	NA	NA
chr5	1.77E+08	1.77E+08	T	G	UTR3	DBN1	NA	NA	NA	NA	NA
chr5	1.77E+08	1.77E+08	A	C	intronic	FAM193B	NA	NA	NA	NA	NA
chr5	1.77E+08	1.77E+08	T	G	exonic	PROP1	nonsynonymous SNV	PROP1:NM_006261:exon3:c.A640C:p.M214L	NA	NA	B
chr5	1.77E+08	1.77E+08	A	G	exonic	PROP1	synonymous SNV	PROP1:NM_006261:exon3:c.T636C:p.P212P	NA	NA	NA
chr5	1.77E+08	1.77E+08	T	G	exonic	PROP1	synonymous SNV	PROP1:NM_006261:exon3:c.A630C:p.P210P	NA	NA	NA
chr5	1.78E+08	1.78E+08	T	C	exonic	RMND5B	synonymous SNV	RMND5B:NM_022762:exon9:c.T888C:p.P296P	NA	rs61751561	NA
chr5	1.78E+08	1.78E+08	T	C	exonic	ZNF354B	synonymous SNV	ZNF354B:NM_058230:exon4:c.T243C:p.G81G	)=COSM150026;OCCURENCE=1(stomacl	rs11952817	NA
chr5	1.79E+08	1.79E+08	A	G	exonic	ADAMTS2	synonymous SNV	M_014244:exon2:c.T321C:p.S107S,ADAMTS2:NM_021599:exon2:c.T3	NA	rs2271212	NA
chr5	1.79E+08	1.79E+08	AG	-	UTR3	HNRNPH1	NA	NA	NA	NA	NA
chr5	1.79E+08	1.79E+08	G	A	exonic	SQSTM1	synonymous SNV	).R312R,SQSTM1:NM_001142298:exon7:c.G684A:p.R228R,SQSTM1:NI	NA	rs4797	NA
chr5	1.79E+08	1.79E+08	G	A	exonic	C5orf45	synonymous SNV	IM_001017987:exon1:c.C45T:p.C15C,C5orf45:NM_016175:exon1:c.C4	NA	rs10060182	NA
chr5	1.8E+08	1.8E+08	C	T	ncRNA_exonic	LINC00847	NA	NA	NA	rs899546	NA
chr5	1.8E+08	1.8E+08	A	G	intronic	ZFP62	NA	NA	NA	rs2127380	NA
chr6	407630	407630	T	-	UTR3	IRF4	NA	NA	NA	NA	NA
chr6	2971853	2971853	A	G	UTR5	SERPINB6	NA	NA	NA	NA	NA
chr6	6007196	6007196	G	-	UTR5	NRN1	NA	NA	NA	rs11285278	NA
chr6	7247248	7247248	T	C	exonic	RREB1	nonsynonymous SNV	p.L1467P,RREB1:NM_001168344:exon11:c.T4400C:p.L1467P,RREB1:N	NA	rs2256596	B
chr6	7249296	7249301	GAGAGA	-	UTR3	RREB1	NA	NA	NA	rs4053178	NA
chr6	7283735	7283735	C	T	UTR3	SSR1	NA	NA	NA	rs111496266	NA
chr6	7283759	7283759	T	C	UTR3	SSR1	NA	NA	NA	rs9505115	NA
chr6	7284948	7284948	-	T	UTR3	SSR1	NA	NA	NA	NA	NA
chr6	7285912	7285912	A	-	UTR3	SSR1	NA	NA	NA	rs71649195	NA
chr6	7287220	7287220	C	A	UTR3	SSR1	NA	NA	NA	NA	NA
chr6	7288297	7288297	G	A	UTR3	SSR1	NA	NA	NA	rs8955	NA
chr6	7289958	7289959	CA	-	UTR3	SSR1	NA	NA	NA	rs16740	NA
chr6	7611993	7611998	TTTCTG	-	UTR3	SNRNP48	NA	NA	NA	rs146960918	NA
chr6	10410434	10410434	T	G	exonic	TFAP2A	synonymous SNV	162C:p.P54P,TFAP2A:NM_001042425:exon2:c.A168C:p.P56P,TFAP2A:I	NA	NA	NA
chr6	10723449	10723449	A	C	UTR5	TMEM14C	NA	NA	NA	rs1045911	NA
chr6	10763780	10763780	A	-	UTR3	MAK	NA	NA	NA	rs35696238	NA
chr6	10887251	10887251	T	C	UTR5	SYCP2L	NA	NA	NA	rs9366663	NA
chr6	10887253	10887253	G	C	UTR5	SYCP2L	NA	NA	NA	rs9358956	NA
chr6	13579828	13579828	A	C	UTR5	SIRT5	NA	NA	NA	rs2804911	NA
chr6	15521302	15521302	-	AA	UTR3	JARID2	NA	NA	NA	NA	NA
chr6	15523807	15523807	-	A	UTR3	DTNBP1	NA	NA	NA	rs142659737	NA
chr6	17393781	17393781	-	C	UTR5	CAP2	NA	NA	NA	NA	NA
chr6	17799575	17799575	A	G	exonic	KIF13A	synonymous SNV	01105567:exon22:c.T2712C:p.A904A,KIF13A:NM_001105568:exon22:(	NA	rs3734235	NA
chr6	18387815	18387815	A	C	UTR5	RNF144B	NA	NA	NA	rs764885	NA
chr6	18467582	18467582	A	G	UTR3	RNF144B	NA	NA	NA	rs531972	NA
chr6	18467626	18467626	C	T	UTR3	RNF144B	NA	NA	NA	NA	NA

chr6	20102263	20102263	G	A	UTR3	MBOAT1	NA	NA	NA	NA	NA
chr6	20534770	20534770	C	T	UTR5	CDKAL1	NA	NA	NA	NA	NA
chr6	20534778	20534778	G	T	UTR5	CDKAL1	NA	NA	NA	NA	NA
chr6	20534791	20534791	C	T	UTR5	CDKAL1	NA	NA	NA	NA	NA
chr6	21594175	21594175	C	T	UTR5	SOX4	NA	NA	NA	NA	NA
chr6	22194615	22194616	AA	-	ncRNA_exonic	LINC00340	NA	NA	NA	NA	NA
chr6	24719384	24719384	A	-	UTR5	C6orf62	NA	NA	NA	rs34131350	NA
chr6	24805465	24805466	AA	-	UTR3	FAM65B	NA	NA	NA	rs67150453	NA
chr6	25115296	25115296	G	T	ncRNA_exonic	CMAHP	NA	NA	NA	rs2273671	NA
chr6	25619946	25619946	A	C	UTR3	LRRC16A	NA	NA	NA	NA	NA
chr6	25913572	25913572	G	T	exonic	SLC17A2	nonsynonymous SNV	SLC17A2:NM_005835:exon11:c.C1262A:p.P421Q	NA	NA	P
chr6	26637020	26637020	C	T	UTR3	ZNF322	NA	NA	NA	rs199645934	NA
chr6	28411220	28411220	T	G	UTR5	ZSCAN23	NA	NA	NA	rs2531803	NA
chr6	29640095	29640111	AAATAAAT	-	UTR3	MOG	NA	NA	NA	NA	NA
chr6	29856508	29856508	T	G	ncRNA_exonic	HLA-H	NA	NA	NA	rs200904024	NA
chr6	29857105	29857105	-	C	ncRNA_exonic	HLA-H	NA	NA	NA	NA	NA
chr6	29866970	29866970	T	C	intergenic	-H(dist=8114),HCG4B(dist=25	NA	NA	NA	rs9259495	NA
chr6	29867247	29867247	A	C	intergenic	-H(dist=8391),HCG4B(dist=25	NA	NA	NA	rs3128995	NA
chr6	29911222	29911222	C	T	exonic	HLA-A	nonsynonymous SNV	_001242758:exon3:c.C521T:p.A174V,HLA-A:NM_002116:exon3:c.C52	NA	rs1059535	B
chr6	29911240	29911240	T	G	exonic	HLA-A	nonsynonymous SNV	_001242758:exon3:c.T539G:p.L180W,HLA-A:NM_002116:exon3:c.T53	NA	rs9260156	B
chr6	29911246	29911246	C	T	exonic	HLA-A	nonsynonymous SNV	_001242758:exon3:c.C545T:p.A182V,HLA-A:NM_002116:exon3:c.C54	NA	rs1059539	B
chr6	29911970	29911970	G	A	exonic	HLA-A	nonsynonymous SNV	_001242758:exon4:c.G691A:p.G231S,HLA-A:NM_002116:exon4:c.G69	NA	rs1059578	B
chr6	29912087	29912087	G	T	exonic	HLA-A	nonsynonymous SNV	_001242758:exon4:c.G808T:p.A270S,HLA-A:NM_002116:exon4:c.G80	NA	rs1059632	B
chr6	29913355	29913355	A	G	UTR3	HLA-A	NA	NA	NA	NA	NA
chr6	29913367	29913367	G	A	UTR3	HLA-A	NA	NA	NA	rs113042145	NA
chr6	29913377	29913377	C	T	UTR3	HLA-A	NA	NA	NA	NA	NA
chr6	29913383	29913383	C	T	UTR3	HLA-A	NA	NA	NA	NA	NA
chr6	30075864	30075864	A	G	exonic	TRIM31	synonymous SNV	TRIM31:NM_007028:exon6:c.T849C:p.H283H	=COSM1131939;OCCURENCE=1(prostat	rs2023472	NA
chr6	30078330	30078330	C	T	exonic	TRIM31	synonymous SNV	TRIM31:NM_007028:exon4:c.G639A:p.A213A	NA	rs2239529	NA
chr6	30227745	30227745	T	G	ncRNA_exonic	HLA-L	NA	NA	NA	NA	NA
chr6	30232250	30232250	A	G	ncRNA_exonic	HLA-L	NA	NA	NA	rs3130404	NA
chr6	30232538	30232538	G	T	ncRNA_exonic	HLA-L	NA	NA	NA	rs28780091	NA
chr6	30232672	30232672	C	T	ncRNA_exonic	HLA-L	NA	NA	NA	rs3129701	NA
chr6	30234627	30234627	A	T	ncRNA_exonic	HLA-L	NA	NA	NA	rs3129831	NA
chr6	30531427	30531427	-	T	UTR3	PRR3	NA	NA	NA	NA	NA
chr6	30573725	30573725	G	T	exonic	PPP1R10	synonymous SNV	PPP1R10:NM_002714:exon10:c.C828A:p.I276I	NA	NA	NA
chr6	30918925	30918925	T	C	exonic	DPCR1	nonsynonymous SNV	DPCR1:NM_080870:exon2:c.T2684C:p.L895P	NA	NA	NA
chr6	30919147	30919147	T	C	exonic	DPCR1	nonsynonymous SNV	DPCR1:NM_080870:exon2:c.T2906C:p.V969A	NA	NA	NA
chr6	30954516	30954516	G	C	exonic	MUC21	synonymous SNV	MUC21:NM_001010909:exon2:c.G564C:p.V188V	NA	NA	NA
chr6	30955101	30955101	G	C	exonic	MUC21	synonymous SNV	MUC21:NM_001010909:exon2:c.G1149C:p.V383V	NA	rs9262395	NA
chr6	31022500	31022500	A	C	ncRNA_intronic	HCG22	NA	NA	NA	rs2523854	NA
chr6	31026434	31026434	A	G	ncRNA_intronic	HCG22	NA	NA	NA	rs2517523	NA
chr6	31026534	31026534	C	T	ncRNA_intronic	HCG22	NA	NA	NA	rs2523847	NA
chr6	31088202	31088202	A	G	UTR5	CDSN	NA	NA	NA	rs2302399	NA
chr6	31237767	31237767	T	C	exonic	HLA-C	nonsynonymous SNV	001243042:exon5:c.A991G:p.M331V,HLA-C:NM_002117:exon5:c.A99	NA	rs1130935	B
chr6	31237769	31237769	G	A	exonic	HLA-C	nonsynonymous SNV	_001243042:exon5:c.C989T:p.A330V,HLA-C:NM_002117:exon5:c.C98	NA	rs1050105	B
chr6	31237771	31237771	G	A	exonic	HLA-C	synonymous SNV	_001243042:exon5:c.C987T:p.T329T,HLA-C:NM_002117:exon5:c.C98	NA	rs1050106	NA
chr6	31237773	31237773	T	C	exonic	HLA-C	nonsynonymous SNV	_001243042:exon5:c.A985G:p.T329A,HLA-C:NM_002117:exon5:c.A98	NA	rs1130947	B
chr6	31237774	31237774	G	C	exonic	HLA-C	synonymous SNV	_001243042:exon5:c.C984G:p.V328V,HLA-C:NM_002117:exon5:c.C98	NA	rs41540512	NA
chr6	31238851	31238851	T	C	exonic	HLA-C	synonymous SNV	_001243042:exon3:c.A618G:p.A206A,HLA-C:NM_002117:exon3:c.A61	NA	rs2308604	NA
chr6	31238880	31238880	C	T	exonic	HLA-C	nonsynonymous SNV	_001243042:exon3:c.G589A:p.E197K,HLA-C:NM_002117:exon3:c.G58	NA	rs1050357	B
chr6	31323945	31323945	A	C	exonic	HLA-B	synonymous SNV	HLA-B:NM_005514:exon3:c.T618G:p.A206A	NA	rs2596493	NA
chr6	31496776	31496776	T	G	UTR5	MCCD1	NA	NA	NA	rs3130054	NA
chr6	31905130	31905130	G	A	exonic	C2	synonymous SNV	381A:p.A127A,C2:NM_001145903:exon6:c.G627A:p.A209A,C2:NM_00	NA	rs1042663	NA
chr6	32010572	32010572	G	T	exonic	TNXB	nonsynonymous SNV	TNXB:NM_019105:exon39:c.C11956A:p.L3986I	NA	rs202148050	P
chr6	32487153	32487153	C	G	exonic	HLA-DRB5	nonsynonymous SNV	HLA-DRB5:NM_002125:exon3:c.G646C:p.E216Q	NA	rs112024745	B
chr6	32487170	32487170	G	A	exonic	HLA-DRB5	nonsynonymous SNV	HLA-DRB5:NM_002125:exon3:c.C629T:p.T210M	NA	rs1136633	B
chr6	32487214	32487214	T	C	exonic	HLA-DRB5	synonymous SNV	HLA-DRB5:NM_002125:exon3:c.A585G:p.R195R	NA	rs144016913	NA
chr6	32549399	32549399	C	A	exonic	HLA-DRB1	nonsynonymous SNV	HLA-DRB1:NM_002124:exon3:c.G587T:p.S196I	NA	NA	NA
chr6	32549525	32549525	C	G	exonic	HLA-DRB1	nonsynonymous SNV	HLA-DRB1:NM_002124:exon3:c.G461C:p.G154A	NA	rs111965977	NA
chr6	32549531	32549531	T	C	exonic	HLA-DRB1	nonsynonymous SNV	HLA-DRB1:NM_002124:exon3:c.A455G:p.Y152C	NA	rs112796209	NA
chr6	32549588	32549588	G	T	exonic	HLA-DRB1	stopgain SNV	HLA-DRB1:NM_002124:exon3:c.C398A:p.S133X	NA	NA	NA
chr6	32557422	32557422	C	T	exonic	HLA-DRB1	nonsynonymous SNV	HLA-DRB1:NM_002124:exon1:c.G98A:p.R33Q	NA	rs17879746	NA
chr6	32557461	32557461	A	G	exonic	HLA-DRB1	nonsynonymous SNV	HLA-DRB1:NM_002124:exon1:c.T59C:p.M20T	NA	rs35053532	NA
chr6	32557465	32557465	G	A	exonic	HLA-DRB1	synonymous SNV	HLA-DRB1:NM_002124:exon1:c.C55T:p.L19L	NA	rs34187469	NA
chr6	32557487	32557487	G	A	exonic	HLA-DRB1	synonymous SNV	HLA-DRB1:NM_002124:exon1:c.C33T:p.C11C	NA	rs34396110	NA

chr6	32609873	32609873	G	C	exonic	HLA-DQA1	nonsynonymous SNV	HLA-DQA1:NM_002122:exon3:c.G456C:p.Q152H	NA	rs707950	B
chr6	32610401	32610401	G	A	exonic	HLA-DQA1	nonsynonymous SNV	HLA-DQA1:NM_002122:exon4:c.G628A:p.A210T	NA	rs9272785	B
chr6	32610436	32610436	T	C	exonic	HLA-DQA1	synonymous SNV	HLA-DQA1:NM_002122:exon4:c.T663C:p.C221C	NA	rs1048372	NA
chr6	32610478	32610478	C	G	exonic	HLA-DQA1	synonymous SNV	HLA-DQA1:NM_002122:exon4:c.C705G:p.G235G	NA	rs1048414	NA
chr6	32610481	32610481	T	C	exonic	HLA-DQA1	synonymous SNV	HLA-DQA1:NM_002122:exon4:c.T708C:p.T236T	NA	rs1048419	NA
chr6	32610757	32610757	A	G	UTR3	HLA-DQA1	NA	NA	NA	rs8227	NA
chr6	32610769	32610769	A	C	UTR3	HLA-DQA1	NA	NA	NA	rs112931520	NA
chr6	32610806	32610806	T	C	UTR3	HLA-DQA1	NA	NA	NA	rs9272896	NA
chr6	32610809	32610809	C	T	UTR3	HLA-DQA1	NA	NA	NA	rs9272899	NA
chr6	32610819	32610819	C	G	UTR3	HLA-DQA1	NA	NA	NA	rs28371169	NA
chr6	32610825	32610825	A	G	UTR3	HLA-DQA1	NA	NA	NA	rs77015062	NA
chr6	32610836	32610836	C	T	UTR3	HLA-DQA1	NA	NA	NA	rs74417865	NA
chr6	32627976	32627976	G	A	UTR3	HLA-DQB1	NA	NA	NA	rs1063345	NA
chr6	32627992	32627992	T	C	UTR3	HLA-DQB1	NA	NA	NA	rs1063343	NA
chr6	32629137	32629137	T	G	exonic	HLA-DQB1	nonsynonymous SNV	_001243961:exon4:c.A759C:p.Q253H,HLA-DQB1:NM_002123:exon4:c.	NA	rs1140343	B
chr6	32629141	32629141	C	T	exonic	HLA-DQB1	nonsynonymous SNV	_001243961:exon4:c.G755A:p.R252H,HLA-DQB1:NM_002123:exon4:c.	NA	rs1140342	B
chr6	32629161	32629161	A	G	exonic	HLA-DQB1	synonymous SNV	1_001243961:exon4:c.T735C:p.L245L,HLA-DQB1:NM_002123:exon4:c.	NA	rs1130430	NA
chr6	32629193	32629193	C	T	exonic	HLA-DQB1	nonsynonymous SNV	_001243961:exon4:c.G703A:p.V235I,HLA-DQB1:NM_002123:exon4:c.	NA	rs1049163	B
chr6	32629847	32629847	A	G	exonic	HLA-DQB1	synonymous SNV	1_001243961:exon3:c.T558C:p.T186T,HLA-DQB1:NM_002123:exon3:c.	NA	rs1049133	NA
chr6	32713827	32713827	C	T	exonic	HLA-DQA2	synonymous SNV	HLA-DQA2:NM_020056:exon3:c.C591T:p.D197D	NA	rs1129956	NA
chr6	32714901	32714901	C	T	downstream	HLA-DQA2	NA	NA	NA	rs9276442	NA
chr6	32714958	32714958	T	A	downstream	HLA-DQA2	NA	NA	NA	rs78430708	NA
chr6	32845561	32845562	AA	-	intergenic	ist=17933),LOC100294145(dis	NA	NA	NA	NA	NA
chr6	33032822	33032822	G	A	UTR3	HLA-DPA1	NA	NA	NA	rs1042926	NA
chr6	33032831	33032831	G	A	UTR3	HLA-DPA1	NA	NA	NA	rs1042920	NA
chr6	33032905	33032905	T	C	UTR3	HLA-DPA1	NA	NA	NA	rs1042872	NA
chr6	33032913	33032913	C	T	UTR3	HLA-DPA1	NA	NA	NA	rs1042866	NA
chr6	33037419	33037419	G	A	exonic	HLA-DPA1	synonymous SNV	N115N,HLA-DPA1:NM_001242524:exon3:c.C345T:p.N115N,HLA-DPA1	NA	rs1126543	NA
chr6	33037424	33037424	T	C	exonic	HLA-DPA1	nonsynonymous SNV	.T114A,HLA-DPA1:NM_001242524:exon3:c.A340G:p.T114A,HLA-DPA1	NA	rs1126542	B
chr6	33037452	33037452	C	A	exonic	HLA-DPA1	nonsynonymous SNV	ɔ.L104F,HLA-DPA1:NM_001242524:exon3:c.G312T:p.L104F,HLA-DPA1:	NA	rs41548220	B
chr6	33037639	33037639	G	A	exonic	HLA-DPA1	nonsynonymous SNV	:p.A42V,HLA-DPA1:NM_001242524:exon3:c.C125T:p.A42V,HLA-DPA1:	D=COSM328641;OCCURENCE=4(kidney	rs1126534	B
chr6	33037640	33037640	C	T	exonic	HLA-DPA1	nonsynonymous SNV	:p.A42T,HLA-DPA1:NM_001242524:exon3:c.G124A:p.A42T,HLA-DPA1:	NA	rs1126533	B
chr6	33054331	33054331	T	C	UTR3	HLA-DPB1	NA	NA	NA	rs1126723	NA
chr6	33054550	33054550	G	A	UTR3	HLA-DPB1	NA	NA	NA	rs931	NA
chr6	33054552	33054552	C	G	UTR3	HLA-DPB1	NA	NA	NA	rs928	NA
chr6	33054559	33054559	T	C	UTR3	HLA-DPB1	NA	NA	NA	rs112596157	NA
chr6	33054659	33054659	A	G	UTR3	HLA-DPB1	NA	NA	NA	rs934	NA
chr6	33179664	33179664	A	G	exonic	RING1	nonsynonymous SNV	RING1:NM_002931:exon6:c.A1004G:p.E335G	NA	rs73741545	NA
chr6	33422438	33422438	A	G	UTR5	ZBTB9	NA	NA	NA	NA	NA
chr6	33422474	33422474	A	G	UTR5	ZBTB9	NA	NA	NA	NA	NA
chr6	33631640	33631640	C	T	exonic	ITPR3	synonymous SNV	ITPR3:NM_002224:exon11:c.C1131T:p.T377T	NA	rs2229631	NA
chr6	34204963	34204963	-	CT	UTR5	HMGA1	NA	NA	NA	NA	NA
chr6	34855748	34855748	G	C	UTR5	TAF11	NA	NA	NA	rs1051115	NA
chr6	35090097	35090097	T	C	exonic	TCP11	synonymous SNV	414G:p.L138L,TCP11:NM_001261817:exon5:c.A399G:p.L133L,TCP11:N	NA	rs2234044	NA
chr6	35791166	35791167	AC	-	UTR3	LHFPL5	NA	NA	NA	rs71840857	NA
chr6	36355372	36355372	T	C	UTR5	ETV7	NA	NA	NA	rs2234071	NA
chr6	36996773	36996773	C	G	UTR3	FGD2	NA	NA	NA	rs708017	NA
chr6	36996840	36996840	A	G	UTR3	FGD2	NA	NA	NA	rs708016	NA
chr6	37185945	37185945	-	T	UTR3	TMEM217	NA	NA	NA	rs34393938	NA
chr6	39041502	39041502	A	C	exonic	GLP1R	nonsynonymous SNV	GLP1R:NM_002062:exon7:c.A780C:p.L260F	NA	rs1042044	NA
chr6	39048491	39048491	A	C	exonic	GLP1R	synonymous SNV	GLP1R:NM_002062:exon12:c.A1200C:p.I400I	NA	rs1126476	NA
chr6	39267139	39267139	C	G	UTR3	KCNK17	NA	NA	NA	rs143564858	NA
chr6	41765828	41765828	A	C	UTR3	USP49	NA	NA	NA	NA	NA
chr6	42047454	42047454	T	-	UTR3	TAF8	NA	NA	NA	NA	NA
chr6	42048115	42048115	-	C	UTR3	TAF8	NA	NA	NA	rs5875787	NA
chr6	42069104	42069104	C	T	UTR3	C6orf132	NA	NA	NA	rs138794174	NA
chr6	42069534	42069534	C	-	UTR3	C6orf132	NA	NA	NA	rs34066163	NA
chr6	42070030	42070030	T	C	UTR3	C6orf132	NA	NA	NA	rs6912553	NA
chr6	43021977	43021977	G	-	UTR3	MRPL2	NA	NA	NA	rs34298469	NA
chr6	43230970	43230970	G	C	exonic	TTBK1	nonsynonymous SNV	TTBK1:NM_032538:exon13:c.G1868C:p.G623A	NA	rs3800294	B
chr6	43586698	43586698	-	G	UTR3	POLH	NA	NA	NA	NA	NA
chr6	44266648	44266648	-	TT	UTR3	AARS2	NA	NA	NA	NA	NA
chr6	45517740	45517741	TT	-	UTR3	RUNX2	NA	NA	NA	NA	NA
chr6	46672208	46672223	CTCTCACA(	-	UTR3	PLA2G7	NA	NA	NA	NA	NA
chr6	46672210	46672225	CTCACACA(	-	UTR3	PLA2G7	NA	NA	NA	NA	NA
chr6	46726915	46726915	C	A	UTR3	ANKRD66	NA	NA	NA	rs9472840	NA

chr6	46832813	46832813	G	A	exonic	GPR116	synonymous SNV	01098518:exon14:c.C1956T:p.V652V,GPR116:NM_015234:exon14:c.C	NA	rs614826	NA
chr6	46834685	46834685	G	A	exonic	GPR116	nonsynonymous SNV	)1098518:exon13:c.C1811T:p.T604M,GPR116:NM_015234:exon13:c.C	NA	rs586024	B
chr6	47200361	47200362	AC	-	UTR3	TNFRSF21	NA	NA	NA	NA	NA
chr6	51481326	51481326	T	-	UTR3	PKHD1	NA	NA	NA	NA	NA
chr6	52101844	52101844	T	C	exonic	IL17F	nonsynonymous SNV	IL17F:NM_052872:exon3:c.A377G:p.E126G	NA	rs2397084	D
chr6	52360331	52360331	G	A	UTR3	EFHC1	NA	NA	NA	rs3789771	NA
chr6	52362416	52362416	T	A	UTR3	TRAM2	NA	NA	NA	NA	NA
chr6	52362443	52362443	G	T	UTR3	TRAM2	NA	NA	NA	NA	NA
chr6	52365335	52365335	T	G	UTR3	TRAM2	NA	NA	NA	NA	NA
chr6	52529413	52529413	A	G	ncRNA_exonic	LOC730101	NA	NA	NA	NA	NA
chr6	53516173	53516173	G	T	UTR3	KLHL31	NA	NA	NA	NA	NA
chr6	53787794	53787794	T	-	UTR3	LRRC1	NA	NA	NA	rs11307213	NA
chr6	55266896	55266896	T	-	UTR3	GFRAL	NA	NA	NA	rs34070544	NA
chr6	55267231	55267231	A	-	UTR3	GFRAL	NA	NA	NA	rs34277824	NA
chr6	57086591	57086591	C	A	UTR5	RAB23	NA	NA	NA	NA	NA
chr6	57512775	57512775	T	G	UTR3	PRIM2	NA	NA	NA	rs7773110	NA
chr6	57512779	57512779	G	T	UTR3	PRIM2	NA	NA	NA	rs76296076	NA
chr6	57513126	57513126	C	G	UTR3	PRIM2	NA	NA	NA	rs56375552	NA
chr6	57513182	57513182	A	G	UTR3	PRIM2	NA	NA	NA	rs1801400	NA
chr6	70048876	70048876	C	T	exonic	BAI3	nonsynonymous SNV	BAI3:NM_001704:exon25:c.C3257T:p.A1086V	NA	NA	P
chr6	70992802	70992802	T	C	UTR5	COL9A1	NA	NA	NA	NA	NA
chr6	71298533	71298533	A	G	UTR3	C6orf57	NA	NA	ID=COSN404929;OCCURENCE=1(lung)	rs13654	NA
chr6	71666146	71666146	T	C	UTR5	B3GAT2	NA	NA	NA	rs1320315	NA
chr6	74497152	74497152	G	A	exonic	CD109	nonsynonymous SNV	)2A:p.V768I,CD109:NM_001159587:exon21:c.G2533A:p.V845I,CD109:I	NA	rs5023688	B
chr6	80411320	80411321	TT	-	UTR3	SH3BGRL2	NA	NA	NA	rs35718956	NA
chr6	81054252	81054252	C	T	UTR3	BCKDHB	NA	NA	NA	rs4706117	NA
chr6	87993555	87993555	T	-	UTR3	GJB7	NA	NA	NA	NA	NA
chr6	89874642	89874642	A	-	UTR3	PM20D2	NA	NA	NA	NA	NA
chr6	89888744	89888744	C	T	exonic	GABRR1	synonymous SNV	02042:exon10:c.G1185A:p.A395A,GABRR1:NM_001256704:exon11:c.(	NA	rs1796743	NA
chr6	90076245	90076245	A	-	UTR3	RRAGD	NA	NA	NA	rs5878092	NA
chr6	94486141	94486141	C	T	ncRNA_exonic	TSG1	NA	NA	NA	rs72922212	NA
chr6	97592900	97592900	C	A	ncRNA_UTR3	MMS22L	NA	NA	NA	NA	NA
chr6	97592942	97592942	-	C	ncRNA_UTR3	MMS22L	NA	NA	NA	NA	NA
chr6	99956560	99956560	T	C	exonic	USP45	nonsynonymous SNV	USP45:NM_001080481:exon3:c.A199G:p.K67E	)=COSM150144;OCCURENCE=1(stomacl	rs7744845	D
chr6	1.06E+08	1.06E+08	A	-	UTR3	BVES	NA	NA	NA	rs35412424	NA
chr6	1.07E+08	1.07E+08	T	G	UTR3	QRSL1	NA	NA	NA	NA	NA
chr6	1.07E+08	1.07E+08	-	T	UTR5	C6orf203	NA	NA	NA	rs144920055	NA
chr6	1.07E+08	1.07E+08	AA	-	UTR3	BEND3	NA	NA	NA	rs200868848	NA
chr6	1.08E+08	1.08E+08	T	G	UTR3	SEC63	NA	NA	NA	rs592989	NA
chr6	1.08E+08	1.08E+08	T	-	UTR3	OSTM1	NA	NA	NA	NA	NA
chr6	1.09E+08	1.09E+08	AC	-	UTR3	NR2E1	NA	NA	NA	rs71757629	NA
chr6	1.1E+08	1.1E+08	G	C	UTR3	PPIL6	NA	NA	NA	rs746794	NA
chr6	1.12E+08	1.12E+08	T	A	UTR3	LAMA4	NA	NA	NA	rs7758715	NA
chr6	1.12E+08	1.12E+08	C	T	UTR3	LAMA4	NA	NA	NA	rs7738331	NA
chr6	1.17E+08	1.17E+08	AAAAAA	-	UTR3	TSPYL1	NA	NA	NA	NA	NA
chr6	1.17E+08	1.17E+08	ATATATAT	-	UTR3	FAM26E	NA	NA	NA	rs72003602	NA
chr6	1.17E+08	1.17E+08	G	A	UTR5	RWDD1	NA	NA	NA	rs62622856	NA
chr6	1.19E+08	1.19E+08	A	G	UTR5	MCM9	NA	NA	NA	rs1885125	NA
chr6	1.23E+08	1.23E+08	G	T	UTR5	PKIB	NA	NA	NA	rs2288661	NA
chr6	1.25E+08	1.25E+08	G	A	ncRNA_exonic	STL	NA	NA	NA	rs1761560	NA
chr6	1.3E+08	1.3E+08	C	T	UTR3	L3MBTL3	NA	NA	NA	NA	NA
chr6	1.3E+08	1.3E+08	C	T	UTR3	L3MBTL3	NA	NA	NA	NA	NA
chr6	1.3E+08	1.3E+08	T	G	UTR3	L3MBTL3	NA	NA	NA	NA	NA
chr6	1.3E+08	1.3E+08	A	G	UTR3	L3MBTL3	NA	NA	NA	NA	NA
chr6	1.32E+08	1.32E+08	A	G	exonic	ENPP3	synonymous SNV	ENPP3:NM_005021:exon8:c.A654G:p.P218P	NA	rs9493048	NA
chr6	1.32E+08	1.32E+08	G	T	UTR3	ENPP1	NA	NA	NA	rs11154649	NA
chr6	1.32E+08	1.32E+08	C	T	UTR3	ENPP1	NA	NA	NA	rs13216504	NA
chr6	1.32E+08	1.32E+08	TT	-	UTR3	ENPP1	NA	NA	NA	NA	NA
chr6	1.32E+08	1.32E+08	C	A	UTR3	ENPP1	NA	NA	NA	NA	NA
chr6	1.33E+08	1.33E+08	A	G	ncRNA_exonic	VNN3	NA	NA	NA	rs2294759	NA
chr6	1.34E+08	1.34E+08	TGTA	-	UTR3	SLC2A12	NA	NA	NA	rs146405495	NA
chr6	1.35E+08	1.35E+08	G	T	UTR3	HBS1L	NA	NA	NA	rs77051210	NA
chr6	1.35E+08	1.35E+08	C	T	UTR3	HBS1L	NA	NA	NA	NA	NA
chr6	1.35E+08	1.35E+08	G	T	exonic	HBS1L	nonsynonymous SNV	)1145158:exon15:c.C1687A:p.Q563K,HBS1L:NM_006620:exon16:c.C1ℇ	NA	NA	D
chr6	1.37E+08	1.37E+08	A	-	UTR3	PDE7B	NA	NA	NA	NA	NA
chr6	1.38E+08	1.38E+08	A	-	UTR3	TNFAIP3	NA	NA	NA	NA	NA

chr6	1.39E+08	1.39E+08	T	C	UTR3	NHSL1	NA	NA	NA	NA	NA
chr6	1.39E+08	1.39E+08	T	G	UTR3	NHSL1	NA	NA	NA	NA	NA
chr6	1.39E+08	1.39E+08	C	T	exonic	CCDC28A	synonymous SNV	CCDC28A:NM_015439:exon1:c.C126T:p.S42S	NA	rs11154999	NA
chr6	1.39E+08	1.39E+08	TT	-	UTR3	HECA	NA	NA	NA	NA	NA
chr6	1.43E+08	1.43E+08	A	C	UTR3	VTA1	NA	NA	NA	rs225711	NA
chr6	1.44E+08	1.44E+08	C	G	UTR3	ADAT2	NA	NA	NA	rs9496617	NA
chr6	1.44E+08	1.44E+08	A	G	UTR3	ADAT2	NA	NA	NA	rs9496619	NA
chr6	1.44E+08	1.44E+08	A	T	UTR3	PEX3	NA	NA	NA	rs223234	NA
chr6	1.44E+08	1.44E+08	A	-	UTR3	FUCA2	NA	NA	NA	rs67844126	NA
chr6	1.44E+08	1.44E+08	TTT	-	UTR3	PHACTR2	NA	NA	NA	NA	NA
chr6	1.45E+08	1.45E+08	G	T	UTR3	STX11	NA	NA	NA	NA	NA
chr6	1.46E+08	1.46E+08	G	A	ncRNA_UTR5	EPM2A	NA	NA	NA	rs2072911	NA
chr6	1.5E+08	1.5E+08	C	T	UTR3	PPIL4	NA	NA	NA	NA	NA
chr6	1.5E+08	1.5E+08	T	C	UTR5	LATS1	NA	NA	NA	rs9767113	NA
chr6	1.5E+08	1.5E+08	C	G	UTR5	LATS1	NA	NA	NA	rs2297932	NA
chr6	1.5E+08	1.5E+08	A	-	UTR3	NUP43	NA	NA	NA	rs11305807	NA
chr6	1.5E+08	1.5E+08	T	C	UTR3	ULBP1	NA	NA	NA	rs12110668	NA
chr6	1.51E+08	1.51E+08	A	G	UTR3	MTHFD1L	NA	NA	NA	rs7646	NA
chr6	1.51E+08	1.51E+08	G	A	UTR3	MTHFD1L	NA	NA	NA	rs7543	NA
chr6	1.51E+08	1.51E+08	A	G	UTR3	MTHFD1L	NA	NA	NA	rs1047665	NA
chr6	1.52E+08	1.52E+08	TT	-	UTR3	AKAP12	NA	NA	NA	NA	NA
chr6	1.52E+08	1.52E+08	AA	-	UTR3	ESR1	NA	NA	NA	NA	NA
chr6	1.53E+08	1.53E+08	C	A	exonic	SYNE1	nonsynonymous SNV	3071:exon76:c.G12566T:p.W4189L,SYNE1:NM_182961:exon77:c.G127	NA	NA	D
chr6	1.53E+08	1.53E+08	G	A	exonic	SYNE1	nonsynonymous SNV	033071:exon54:c.C8405T:p.A2802V,SYNE1:NM_182961:exon54:c.C838	NA	rs214950	B
chr6	1.53E+08	1.53E+08	G	A	UTR5	SYNE1	NA	NA	NA	rs9478345	NA
chr6	1.53E+08	1.53E+08	T	C	UTR5	FBXO5	NA	NA	NA	NA	NA
chr6	1.53E+08	1.53E+08	G	T	UTR3	MTRF1L	NA	NA	NA	rs111229801	NA
chr6	1.53E+08	1.53E+08	C	T	exonic	MTRF1L	nonsynonymous SNV	_001114184:exon1:c.G227A:p.R76Q,MTRF1L:NM_019041:exon1:c.G2	NA	rs3818123	B
chr6	1.54E+08	1.54E+08	A	G	exonic	OPRM1	synonymous SNV	OPRM1:NM_001008505:exon4:c.A1323G:p.G441G	NA	rs675026	NA
chr6	1.54E+08	1.54E+08	C	T	exonic	OPRM1	synonymous SNV	OPRM1:NM_001008505:exon4:c.C1333T:p.L445L	NA	rs562859	NA
chr6	1.55E+08	1.55E+08	G	A	exonic	TIAM2	synonymous SNV	TIAM2:NM_012454:exon7:c.G2034A:p.Q678Q	NA	rs1032141	NA
chr6	1.56E+08	1.56E+08	AAA	-	UTR3	TFB1M,TIAM2	NA	NA	NA	NA	NA
chr6	1.56E+08	1.56E+08	A	G	upstream	TFB1M	NA	NA	NA	rs1334688	NA
chr6	1.58E+08	1.58E+08	A	-	UTR3	ZDHHC14	NA	NA	NA	NA	NA
chr6	1.58E+08	1.58E+08	A	T	UTR3	ZDHHC14	NA	NA	NA	NA	NA
chr6	1.59E+08	1.59E+08	G	A	UTR3	GTF2H5	NA	NA	NA	rs9457163	NA
chr6	1.59E+08	1.59E+08	TTT	-	UTR3	TULP4	NA	NA	NA	NA	NA
chr6	1.59E+08	1.59E+08	C	T	exonic	SYTL3	synonymous SNV	1009991:exon14:c.C1173T:p.L391L,SYTL3:NM_001242394:exon15:c.C	NA	rs117895837	NA
chr6	1.59E+08	1.59E+08	A	-	UTR3	EZR	NA	NA	NA	NA	NA
chr6	1.59E+08	1.59E+08	A	C	UTR3	EZR	NA	NA	NA	NA	NA
chr6	1.6E+08	1.6E+08	T	C	exonic	FNDC1	synonymous SNV	FNDC1:NM_032532:exon11:c.T3270C:p.D1090D	NA	NA	NA
chr6	1.61E+08	1.61E+08	TGTG	-	UTR3	IGF2R	NA	NA	NA	NA	NA
chr6	1.61E+08	1.61E+08	C	T	exonic	SLC22A2	synonymous SNV	SLC22A2:NM_003058:exon10:c.G1506A:p.V502V	NA	rs316003	NA
chr6	1.64E+08	1.64E+08	G	C	UTR3	QKI	NA	NA	NA	NA	NA
chr6	1.67E+08	1.67E+08	T	C	UTR5	SFT2D1	NA	NA	NA	rs10862	NA
chr6	1.67E+08	1.67E+08	A	C	UTR5	SFT2D1	NA	NA	NA	rs11168	NA
chr6	1.68E+08	1.68E+08	G	C	UTR5	CCR6	NA	NA	NA	rs1012656	NA
chr6	1.68E+08	1.68E+08	A	C	ncRNA_exonic	MLLT4-AS1	NA	NA	NA	NA	NA
chr6	1.68E+08	1.68E+08	A	C	ncRNA_exonic	MLLT4-AS1	NA	NA	NA	NA	NA
chr6	1.68E+08	1.68E+08	A	C	ncRNA_exonic	MLLT4-AS1	NA	NA	NA	NA	NA
chr6	1.68E+08	1.68E+08	GTGTGTGT	-	UTR3	MLLT4	NA	NA	NA	NA	NA
chr6	1.68E+08	1.68E+08	G	T	exonic	HGC6.3	synonymous SNV	HGC6.3:NM_001129895:exon1:c.C282A:p.P94P	NA	NA	NA
chr6	1.68E+08	1.68E+08	G	C	exonic	FRMD1	nonsynonymous SNV	01122841:exon10:c.C1162G:p.Q388E,FRMD1:NM_024919:exon10:c.C1	NA	rs1548349	B
chr6	1.68E+08	1.68E+08	G	A	exonic	FRMD1	synonymous SNV	FRMD1:NM_024919:exon1:c.C171T:p.L57L	NA	rs34054991	NA
chr6	1.7E+08	1.7E+08	C	T	exonic	THBS2	synonymous SNV	THBS2:NM_003247:exon6:c.G777A:p.S259S	NA	rs61730651	NA
chr7	537542	537542	G	A	UTR3	PDGFA	NA	NA	NA	NA	NA
chr7	537560	537560	T	A	UTR3	PDGFA	NA	NA	NA	NA	NA
chr7	537690	537690	T	G	UTR3	PDGFA	NA	NA	NA	NA	NA
chr7	1478615	1478615	T	G	exonic	MICALL2	synonymous SNV	MICALL2:NM_182924:exon10:c.A1983C:p.P661P	NA	NA	NA
chr7	1787520	1787520	A	-	UTR3	ELFN1	NA	NA	NA	rs5881906	NA
chr7	2567705	2567705	A	G	UTR3	LFNG	NA	NA	NA	NA	NA
chr7	4809075	4809075	T	-	UTR3	FOXK1	NA	NA	NA	NA	NA
chr7	4810496	4810501	GTGTGT	-	UTR3	FOXK1	NA	NA	NA	NA	NA
chr7	4841318	4841318	G	A	exonic	RADIL	synonymous SNV	RADIL:NM_018059:exon12:c.C2808T:p.N936N	NA	rs6945581	NA
chr7	5013688	5013688	A	G	ncRNA_exonic	RNF216P1	NA	NA	NA	rs6978215	NA
chr7	5346838	5346838	-	T	UTR3	TNRC18	NA	NA	NA	NA	NA

chr7	5352665	5352665	-	GAGGAG	exonic	TNRC18	nonframeshift insertion	RC18:NM_001080495:exon27:c.7857_7858insCTCCTC:p.S2619delinsS	NA	NA	NA
chr7	5660802	5660802	-	ATCTTTT	UTR3	RNF216	NA	NA	NA	NA	NA
chr7	6448941	6448941	T	G	UTR3	DAGLB	NA	NA	NA	NA	NA
chr7	9673930	9673930	G	-	ncRNA_exonic	PER4	NA	NA	NA	rs5882228	NA
chr7	9675063	9675063	C	A	ncRNA_exonic	PER4	NA	NA	NA	NA	NA
chr7	11411963	11411963	-	TT	UTR3	THSD7A	NA	NA	NA	NA	NA
chr7	12370657	12370657	A	G	UTR3	VWDE	NA	NA	NA	rs2253353	NA
chr7	12370794	12370794	T	C	UTR3	VWDE	NA	NA	NA	rs1059002	NA
chr7	12730250	12730250	-	T	UTR3	ARL4A	NA	NA	NA	NA	NA
chr7	15725798	15725800	TGG	-	exonic	MEOX2	nonframeshift deletion	MEOX2:NM_005924:exon1:c.228_230del:p.76_77del	l150907;OCCURENCE=1(stomach),1(oes	rs113582077	NA
chr7	15726129	15726129	A	C	UTR5	MEOX2	NA	NA	NA	rs11975534	NA
chr7	16130175	16130175	A	-	UTR3	ISPD	NA	NA	NA	NA	NA
chr7	16666759	16666759	G	C	exonic	ANKMY2	synonymous SNV	ANKMY2:NM_020319:exon3:c.C177G:p.L59L	NA	rs11531477	NA
chr7	20180473	20180473	C	G	UTR3	MACC1	NA	NA	NA	NA	NA
chr7	21599233	21599233	C	T	exonic	DNAH11	synonymous SNV	DNAH11:NM_001277115:exon4:c.C705T:p.N235N	NA	rs10950854	NA
chr7	22158518	22158518	G	C	UTR3	RAPGEF5	NA	NA	NA	NA	NA
chr7	22158526	22158526	A	C	UTR3	RAPGEF5	NA	NA	NA	rs111375567	NA
chr7	22160845	22160845	A	-	UTR3	RAPGEF5	NA	NA	NA	rs10716365	NA
chr7	23531029	23531029	-	A	ncRNA_exonic	RPS2P32	NA	NA	NA	NA	NA
chr7	23545140	23545140	G	A	UTR3	TRA2A	NA	NA	NA	NA	NA
chr7	23545142	23545142	G	A	UTR3	TRA2A	NA	NA	NA	NA	NA
chr7	23545143	23545143	G	A	UTR3	TRA2A	NA	NA	NA	NA	NA
chr7	23749888	23749888	A	G	UTR5	STK31	NA	NA	NA	NA	NA
chr7	25219341	25219341	C	T	UTR5	C7orf31	NA	NA	NA	rs117565579	NA
chr7	27147452	27147452	A	-	UTR3	HOXA3	NA	NA	NA	rs59078276	NA
chr7	27224599	27224599	T	G	exonic	HOXA11	nonsynonymous SNV	HOXA11:NM_005523:exon1:c.A165C:p.Q55H	NA	NA	D
chr7	28860743	28860743	-	TGTG	UTR3	CREB5	NA	NA	NA	NA	NA
chr7	29688566	29688566	-	AAA	ncRNA_exonic	LOC646762	NA	NA	NA	NA	NA
chr7	30796038	30796038	A	T	ncRNA_UTR3	INMT	NA	NA	NA	rs10258337	NA
chr7	32525555	32525557	AAA	-	UTR3	LSM5	NA	NA	NA	NA	NA
chr7	32525561	32525561	A	C	UTR3	LSM5	NA	NA	NA	rs7784803	NA
chr7	32525564	32525564	A	C	UTR3	LSM5	NA	NA	NA	NA	NA
chr7	32613020	32613020	A	G	exonic	AVL9	synonymous SNV	AVL9:NM_015060:exon12:c.A1560G:p.T520T	)=COSM150265;OCCURENCE=1(stomac	rs1993050	NA
chr7	33045704	33045704	T	-	UTR3	FKBP9	NA	NA	NA	NA	NA
chr7	36447349	36447349	-	CTT	exonic	ANLN	nonframeshift insertion	ANLN:NM_018685:exon5:c.880_881insCTT:p.T294delinsTS	NA	rs143969069	NA
chr7	37991430	37991430	T	-	UTR3	EPDR1	NA	NA	NA	rs5883621	NA
chr7	41728701	41728702	TG	-	UTR3	INHBA	NA	NA	NA	NA	NA
chr7	44836268	44836268	C	G	UTR5	PPIA	NA	NA	NA	rs8177826	NA
chr7	44868379	44868379	A	G	UTR3	H2AFV	NA	NA	NA	rs1058056	NA
chr7	44868971	44868971	G	A	UTR3	H2AFV	NA	NA	NA	rs66566005	NA
chr7	44919001	44919002	TT	-	UTR3	PURB	NA	NA	NA	rs71710319	NA
chr7	45123577	45123577	C	T	exonic	NACAD	synonymous SNV	NACAD:NM_001146334:exon2:c.G2202A:p.S734S	NA	NA	NA
chr7	45123660	45123660	A	G	exonic	NACAD	nonsynonymous SNV	NACAD:NM_001146334:exon2:c.T2119C:p.S707P	NA	rs202009438	NA
chr7	48965141	48965141	A	G	ncRNA_exonic	CDC14C	NA	NA	NA	rs28454487	NA
chr7	50199066	50199066	G	C	downstream	C7orf72	NA	NA	NA	rs6966228	NA
chr7	50628740	50628743	CTCT	-	UTR5	DDC	NA	NA	NA	rs3837091	NA
chr7	56079222	56079222	A	-	UTR3	PSPH	NA	NA	NA	rs34727399	NA
chr7	56147931	56147931	C	A	UTR3	SUMF2	NA	NA	NA	NA	NA
chr7	64254822	64254832	3CTGCAGG	-	UTR5	ZNF138	NA	NA	NA	rs112918223	NA
chr7	64436819	64436819	-	A	UTR3	ZNF117	NA	NA	NA	NA	NA
chr7	65159983	65159983	A	G	ncRNA_exonic	INTS4L2	NA	NA	NA	rs147104756	NA
chr7	65228273	65228273	G	A	ncRNA_exonic	CCT6P1	NA	NA	NA	rs1131689	NA
chr7	70257602	70257602	T	-	UTR3	AUTS2	NA	NA	NA	rs71777072	NA
chr7	72304471	72304471	T	-	ncRNA_exonic	SBDSP1	NA	NA	NA	rs5884895	NA
chr7	73112466	73112466	G	A	UTR3	WBSCR22	NA	NA	NA	NA	NA
chr7	73818404	73818404	C	T	UTR3	CLIP2	NA	NA	NA	rs2240436	NA
chr7	76669028	76669028	A	C	ncRNA_exonic	LOC100132832	NA	NA	NA	NA	NA
chr7	76669044	76669044	G	C	ncRNA_exonic	LOC100132832	NA	NA	NA	rs1063947	NA
chr7	76669060	76669060	G	A	ncRNA_exonic	LOC100132832	NA	NA	NA	NA	NA
chr7	76669085	76669085	T	A	ncRNA_exonic	LOC100132832	NA	NA	ID=COSN405014;OCCURENCE=1(lung)	NA	NA
chr7	76991935	76991935	C	T	exonic	GSAP	nonsynonymous SNV	GSAP:NM_017439:exon13:c.G914A:p.G305E	NA	rs1527263	B
chr7	77408761	77408762	AT	-	UTR3	RSBN1L	NA	NA	NA	rs10569815	NA
chr7	82784471	82784471	A	G	exonic	PCLO	nonsynonymous SNV	∕_014510:exon2:c.T1486C:p.S496P,PCLO:NM_033026:exon2:c.T1486C	46480,COSM246482;OCCURENCE=2(ki	rs199515717	NA
chr7	83278198	83278198	G	A	UTR5	SEMA3E	NA	NA	NA	rs200070960	NA
chr7	84625293	84625293	T	-	UTR3	SEMA3D	NA	NA	NA	NA	NA
chr7	89863860	89863860	-	T	UTR3	STEAP2	NA	NA	NA	NA	NA

chr7	91742142	91742142	-	A	UTR3	CYP51A1	NA	NA	NA	NA	NA
chr7	91829671	91829671	-	T	UTR3	KRIT1	NA	NA	NA	NA	NA
chr7	92088521	92088521	-	AG	UTR3	GATAD1	NA	NA	NA	rs112382994	NA
chr7	92775019	92775019	T	C	UTR5	SAMD9L	NA	NA	NA	rs28662	NA
chr7	94030899	94030899	T	C	exonic	COL1A2	synonymous SNV	COL1A2:NM_000089:exon6:c.T246C:p.D82D	NA	rs1800222	NA
chr7	94989256	94989256	T	-	UTR3	PON3	NA	NA	NA	NA	NA
chr7	95025600	95025600	G	A	exonic	PON3	synonymous SNV	PON3:NM_000940:exon1:c.C63T:p.F21F	NA	rs13226149	NA
chr7	97837250	97837251	AT	-	UTR3	LMTK2	NA	NA	NA	rs140647225	NA
chr7	98445139	98445139	G	C	UTR3	TMEM130	NA	NA	NA	rs147506687	NA
chr7	98792835	98792835	G	A	exonic	KPNA7	synonymous SNV	KPNA7:NM_001145715:exon4:c.C411T:p.A137A	NA	rs61751722	NA
chr7	99752122	99752122	A	-	UTR3	C7orf43	NA	NA	NA	NA	NA
chr7	1E+08	1E+08	T	G	exonic	MEPCE	nonsynonymous SNV	MEPCE:NM_019606:exon1:c.T518G:p.V173G	NA	NA	D
chr7	1E+08	1E+08	A	G	UTR3	NYAP1	NA	NA	NA	NA	NA
chr7	1E+08	1E+08	C	G	UTR3	NYAP1	NA	NA	NA	NA	NA
chr7	1E+08	1E+08	T	G	UTR3	NYAP1	NA	NA	NA	NA	NA
chr7	1E+08	1E+08	T	G	UTR3	NYAP1	NA	NA	NA	NA	NA
chr7	1E+08	1E+08	TT	-	UTR3	AGFG2	NA	NA	NA	NA	NA
chr7	1E+08	1E+08	T	G	UTR3	GIGYF1	NA	NA	NA	NA	NA
chr7	1E+08	1E+08	A	C	UTR3	GIGYF1	NA	NA	NA	NA	NA
chr7	1E+08	1E+08	AA	-	UTR3	GIGYF1	NA	NA	NA	NA	NA
chr7	1E+08	1E+08	T	-	UTR3	GIGYF1	NA	NA	NA	rs146231586	NA
chr7	1E+08	1E+08	T	C	exonic	ZAN	unknown	UNKNOWN	NA	NA	NA
chr7	1E+08	1E+08	T	C	exonic	ZAN	unknown	UNKNOWN	NA	rs200387712	NA
chr7	1E+08	1E+08	T	C	exonic	ZAN	unknown	UNKNOWN	NA	rs201073731	NA
chr7	1E+08	1E+08	C	A	exonic	ZAN	unknown	UNKNOWN	NA	rs2734880	NA
chr7	1E+08	1E+08	C	T	exonic	ZAN	unknown	UNKNOWN	NA	rs314298	NA
chr7	1E+08	1E+08	-	G	exonic	ZAN	unknown	UNKNOWN	NA	rs148800656	NA
chr7	1E+08	1E+08	T	C	exonic	ZAN	unknown	UNKNOWN	NA	rs531503	NA
chr7	1E+08	1E+08	-	G	UTR5	SRRT	NA	NA	NA	rs11462700	NA
chr7	1.01E+08	1.01E+08	C	A	exonic	MUC12	nonsynonymous SNV	MUC12:NM_001164462:exon2:c.C5574A:p.D1858E	NA	rs200702634	NA
chr7	1.01E+08	1.01E+08	G	A	exonic	MUC12	nonsynonymous SNV	MUC12:NM_001164462:exon2:c.G5575A:p.A1859T	NA	rs201905745	NA
chr7	1.01E+08	1.01E+08	C	T	exonic	MUC12	nonsynonymous SNV	MUC12:NM_001164462:exon2:c.C7787T:p.T2596I	NA	NA	NA
chr7	1.01E+08	1.01E+08	C	G	exonic	MUC12	nonsynonymous SNV	MUC12:NM_001164462:exon2:c.C8078G:p.P2693R	NA	NA	NA
chr7	1.01E+08	1.01E+08	G	A	exonic	MUC12	nonsynonymous SNV	MUC12:NM_001164462:exon2:c.G11150A:p.R3717H	NA	rs200935425	NA
chr7	1.01E+08	1.01E+08	A	T	exonic	MUC12	nonsynonymous SNV	MUC12:NM_001164462:exon2:c.A12560T:p.K4187I	NA	rs202045957	NA
chr7	1.01E+08	1.01E+08	T	C	UTR3	MUC17	NA	NA	NA	rs4729655	NA
chr7	1.01E+08	1.01E+08	T	A	UTR3	MUC17	NA	NA	NA	rs4729656	NA
chr7	1.01E+08	1.01E+08	G	A	exonic	COL26A1	unknown	UNKNOWN	NA	rs17393069	NA
chr7	1.02E+08	1.02E+08	A	-	UTR3	ORAI2	NA	NA	NA	rs11303681	NA
chr7	1.02E+08	1.02E+08	A	-	UTR3	ORAI2	NA	NA	NA	NA	NA
chr7	1.02E+08	1.02E+08	C	A	UTR3	RASA4	NA	NA	NA	rs11547188	NA
chr7	1.02E+08	1.02E+08	T	C	exonic	RASA4,RASA4B	nonsynonymous SNV	2G:p.E141G,RASA4B:NM_001277335:exon5:c.A422G:p.E141G,RASA4:I	NA	rs11547189	D
chr7	1.02E+08	1.02E+08	A	G	exonic	RASA4,RASA4B	synonymous SNV	9C:p.D113D,RASA4B:NM_001277335:exon5:c.C339C:p.D113D,RASA4:I	NA	rs11547191	NA
chr7	1.02E+08	1.02E+08	C	G	UTR5	POLR2J2	NA	NA	NA	rs71559483	NA
chr7	1.03E+08	1.03E+08	G	-	ncRNA_exonic	DPY19L2P2	NA	NA	NA	NA	NA
chr7	1.03E+08	1.03E+08	A	G	exonic	RELN	synonymous SNV	005045:exon50:c.T7887C:p.P2629P,RELN:NM_173054:exon50:c.T7887	NA	rs56345626	NA
chr7	1.07E+08	1.07E+08	A	C	UTR3	BCAP29	NA	NA	NA	rs9691194	NA
chr7	1.08E+08	1.08E+08	A	G	UTR5	LAMB1	NA	NA	NA	rs2070917	NA
chr7	1.15E+08	1.15E+08	C	T	UTR3	MDFIC	NA	NA	NA	NA	NA
chr7	1.16E+08	1.16E+08	AAAAAAAG	-	UTR3	CAV2	NA	NA	NA	rs201863771	NA
chr7	1.16E+08	1.16E+08	A	-	UTR3	CAV1	NA	NA	NA	NA	NA
chr7	1.17E+08	1.17E+08	C	T	exonic	CFTR	nonsynonymous SNV	CFTR:NM_000492:exon10:c.C1265T:p.S422F	NA	rs201880593	B
chr7	1.17E+08	1.17E+08	A	G	exonic	CFTR	nonsynonymous SNV	CFTR:NM_000492:exon10:c.A1312G:p.T438A	NA	rs201434579	B
chr7	1.17E+08	1.17E+08	G	T	exonic	CFTR	synonymous SNV	CFTR:NM_000492:exon10:c.G1365T:p.A455A	NA	rs79074685	NA
chr7	1.17E+08	1.17E+08	TCT	-	exonic	CFTR	nonframeshift deletion	CFTR:NM_000492:exon11:c.1520_1522del:p.507_508del	NA	rs199826652	NA
chr7	1.17E+08	1.17E+08	G	A	exonic	CFTR	synonymous SNV	CFTR:NM_000492:exon11:c.G1584A:p.E528E	NA	rs1800095	NA
chr7	1.21E+08	1.21E+08	A	-	UTR3	ING3	NA	NA	NA	NA	NA
chr7	1.21E+08	1.21E+08	C	G	UTR3	ING3	NA	NA	NA	NA	NA
chr7	1.21E+08	1.21E+08	-	CACC	UTR5	WNT16	NA	NA	NA	rs10668066	NA
chr7	1.22E+08	1.22E+08	T	G	exonic	PTPRZ1	nonsynonymous SNV	on1:c.T8G:p.I3S,PTPRZ1:NM_001206839:exon1:c.T8G:p.I3S,PTPRZ1:NM	NA	rs740965	NA
chr7	1.27E+08	1.27E+08	-	GT	UTR3	PAX4	NA	NA	NA	rs36159526	NA
chr7	1.28E+08	1.28E+08	C	T	exonic	FAM71F1	nonsynonymous SNV	FAM71F1:NM_032599:exon3:c.C683T:p.S228L	NA	rs6949056	P
chr7	1.28E+08	1.28E+08	T	G	UTR5	CALU	NA	NA	NA	NA	NA
chr7	1.3E+08	1.3E+08	-	TG	UTR3	CPA4	NA	NA	NA	NA	NA
chr7	1.3E+08	1.3E+08	T	G	UTR3	CPA4	NA	NA	NA	rs138933581	NA
chr7	1.3E+08	1.3E+08	A	-	ncRNA_exonic	MESTIT1	NA	NA	NA	rs201085778	NA

chr7	1.3E+08	1.3E+08	A	-	UTR3	COPG2	NA	NA	NA	rs61354155	NA
chr7	1.3E+08	1.3E+08	G	A	UTR3	COPG2	NA	NA	NA	NA	NA
chr7	1.3E+08	1.3E+08	A	G	exonic	KLF14	synonymous SNV	KLF14:NM_138693:exon1:c.T879C:p.T293T	NA	NA	NA
chr7	1.31E+08	1.31E+08	AA	-	UTR3	MKLN1	NA	NA	NA	NA	NA
chr7	1.35E+08	1.35E+08	C	T	UTR3	C7orf73	NA	NA	NA	NA	NA
chr7	1.37E+08	1.37E+08	CACA	-	UTR5	CHRM2	NA	NA	NA	NA	NA
chr7	1.38E+08	1.38E+08	A	T	UTR3	SVOPL	NA	NA	NA	rs1439793	NA
chr7	1.38E+08	1.38E+08	A	-	UTR3	TMEM213	NA	NA	NA	rs61044050	NA
chr7	1.39E+08	1.39E+08	T	-	UTR3	KIAA1549	NA	NA	NA	NA	NA
chr7	1.4E+08	1.4E+08	CTTCCTTC(	-	ncRNA_exonic	LOC100134229	NA	NA	NA	NA	NA
chr7	1.4E+08	1.4E+08	CCTCCCTTT(	-	ncRNA_exonic	LOC100134229	NA	NA	NA	NA	NA
chr7	1.4E+08	1.4E+08	CCTTTCCTT	-	ncRNA_exonic	LOC100134229	NA	NA	NA	NA	NA
chr7	1.4E+08	1.4E+08	A	G	exonic	DENND2A	synonymous SNV	DENND2A:NM_015689:exon1:c.T909C:p.P303P	NA	NA	NA
chr7	1.42E+08	1.42E+08	C	T	exonic	MGAM	nonsynonymous SNV	MGAM:NM_004668:exon10:c.C1201T:p.R401C	NA	rs188481752	NA
chr7	1.42E+08	1.42E+08	T	C	exonic	PRSS2	unknown	UNKNOWN	NA	NA	NA
chr7	1.43E+08	1.43E+08	C	T	exonic	CLCN1	synonymous SNV	CLCN1:NM_000083:exon17:c.C2154T:p.D718D	NA	rs2272251	NA
chr7	1.43E+08	1.43E+08	T	C	exonic	EPHA1	synonymous SNV	EPHA1:NM_005232:exon17:c.A2742G:p.P914P	NA	rs1804527	NA
chr7	1.43E+08	1.43E+08	G	C	exonic	CTAGE15	nonsynonymous SNV	CTAGE15:NM_001008747:exon1:c.G463C:p.E155Q	NA	rs201542928	NA
chr7	1.43E+08	1.43E+08	C	T	exonic	CTAGE15	nonsynonymous SNV	CTAGE15:NM_001008747:exon1:c.C1091T:p.A364V	NA	rs201104924	NA
chr7	1.43E+08	1.43E+08	C	T	exonic	FAM115C	nonsynonymous SNV	T:p.R101W,FAM115C:NM_001130025:exon3:c.C793T:p.R265W,FAM11	NA	rs143124178	D
chr7	1.43E+08	1.43E+08	C	T	UTR3	FAM115C	NA	NA	NA	rs139515914	NA
chr7	1.43E+08	1.43E+08	A	G	exonic	CTAGE6	nonsynonymous SNV	CTAGE6:NM_178561:exon1:c.T2077C:p.F693L	NA	rs62484795	NA
chr7	1.44E+08	1.44E+08	G	A	ncRNA_UTR3	CTAGE4	NA	NA	NA	rs1048325	NA
chr7	1.49E+08	1.49E+08	G	-	UTR3	EZH2	NA	NA	NA	rs3217095	NA
chr7	1.49E+08	1.49E+08	A	-	UTR3	PDIA4	NA	NA	NA	NA	NA
chr7	1.49E+08	1.49E+08	A	-	UTR3	ZNF786	NA	NA	NA	NA	NA
chr7	1.49E+08	1.49E+08	A	G	ncRNA_exonic	LOC155060	NA	NA	NA	rs13236978	NA
chr7	1.5E+08	1.5E+08	A	C	UTR3	ZNF862	NA	NA	NA	NA	NA
chr7	1.51E+08	1.51E+08	C	A	UTR3	PRKAG2	NA	NA	NA	rs202151040	NA
chr7	1.52E+08	1.52E+08	T	G	UTR3	GALNT11	NA	NA	NA	NA	NA
chr7	1.52E+08	1.52E+08	T	G	UTR3	GALNT11	NA	NA	NA	NA	NA
chr7	1.52E+08	1.52E+08	G	T	exonic	MLL3	synonymous SNV	MLL3:NM_170606:exon15:c.C2646A:p.I882I	NA	rs201452267	NA
chr7	1.52E+08	1.52E+08	G	A	exonic	MLL3	nonsynonymous SNV	MLL3:NM_170606:exon15:c.C2578T:p.P860S	NA	rs112515611	D
chr7	1.55E+08	1.55E+08	T	C	UTR3	EN2	NA	NA	NA	rs55729502	NA
chr7	1.55E+08	1.55E+08	C	T	UTR3	EN2	NA	NA	NA	rs3808326	NA
chr7	1.55E+08	1.55E+08	A	G	UTR5	CNPY1	NA	NA	NA	rs6947243	NA
chr7	1.57E+08	1.57E+08	C	T	UTR3	MNX1	NA	NA	NA	NA	NA
chr7	1.59E+08	1.59E+08	G	C	UTR3	VIPR2	NA	NA	NA	rs1965613	NA
chr8	1733482	1733482	C	G	UTR3	CLN8	NA	NA	NA	rs111861349	NA
chr8	1733695	1733695	A	C	UTR3	CLN8	NA	NA	NA	NA	NA
chr8	6793581	6793581	A	G	exonic	DEFA4	synonymous SNV	DEFA4:NM_001925:exon3:c.T255C:p.G85G	NA	rs2738100	NA
chr8	7718180	7718180	T	C	exonic	SPAG11A	nonsynonymous SNV	SPAG11A:NM_001081552:exon3:c.T227C:p.L76P	NA	rs1071602	NA
chr8	7721244	7721244	C	T	UTR3	SPAG11A	NA	NA	NA	rs2740048	NA
chr8	8176554	8176554	C	T	exonic	SGK223	nonsynonymous SNV	SGK223:NM_001080826:exon5:c.G3331A:p.A1111T	NA	rs12549973	NA
chr8	10390452	10390452	C	T	exonic	PRSS55	nonsynonymous SNV	_001197020:exon4:c.C635T:p.A212V,PRSS55:NM_198464:exon4:c.C6	NA	rs4406360	B
chr8	10465247	10465247	T	C	exonic	RP1L1	nonsynonymous SNV	RP1L1:NM_178857:exon4:c.A6361G:p.T2121A	NA	NA	NA
chr8	11183978	11183978	-	TTGTTG	UTR3	MTMR9	NA	NA	NA	rs145931471	NA
chr8	11644751	11644751	A	G	UTR3	NEIL2	NA	NA	NA	rs4639	NA
chr8	12039778	12039778	G	A	UTR3	FAM86B1	NA	NA	NA	rs2698910	NA
chr8	12044328	12044328	T	C	exonic	FAM86B1	synonymous SNV	FAM86B1:NM_001083537:exon4:c.A255G:p.S85S	NA	rs138175362	NA
chr8	12881550	12881550	-	TT	UTR3	KIAA1456	NA	NA	NA	NA	NA
chr8	12886083	12886083	C	T	UTR3	KIAA1456	NA	NA	NA	rs7464342	NA
chr8	12943086	12943086	T	-	UTR3	DLC1	NA	NA	NA	rs60094700	NA
chr8	17080240	17080264	ACATTATCT	-	UTR3	ZDHHC2	NA	NA	NA	rs11271419	NA
chr8	17796382	17796382	A	G	exonic	PCM1	nonsynonymous SNV	PCM1:NM_006197:exon5:c.A476G:p.N159S	NA	rs41364448	NA
chr8	17796383	17796383	C	T	exonic	PCM1	synonymous SNV	PCM1:NM_006197:exon5:c.C477T:p.N159N	NA	rs114261839	NA
chr8	18081198	18081198	A	-	UTR3	NAT1	NA	NA	NA	rs68160149	NA
chr8	20002739	20002739	-	G	UTR3	SLC18A1	NA	NA	NA	rs140084839	NA
chr8	22069386	22069386	T	C	UTR3	BMP1	NA	NA	NA	rs7001	NA
chr8	22546609	22546609	G	A	UTR3	EGR3	NA	NA	NA	NA	NA
chr8	23190941	23190941	T	C	exonic	LOXL2	synonymous SNV	LOXL2:NM_002318:exon5:c.A939G:p.S313S	NA	rs1010156	NA
chr8	23429691	23429691	G	A	UTR3	SLC25A37	NA	NA	NA	NA	NA
chr8	23536563	23536563	-	T	UTR3	NKX3-1	NA	NA	NA	NA	NA
chr8	23537685	23537685	T	A	UTR3	NKX3-1	NA	NA	NA	NA	NA
chr8	25269595	25269595	T	-	UTR3	DOCK5	NA	NA	NA	NA	NA
chr8	26240646	26240646	A	G	UTR5	BNIP3L	NA	NA	NA	rs1055479	NA

chr8	26435510	26435511	TC	-	UTR5	DPYSL2	NA	NA	NA	rs145510839	NA
chr8	26605958	26605958	A	-	UTR3	ADRA1A	NA	NA	NA	rs34923219	NA
chr8	27143206	27143206	C	-	UTR3	TRIM35	NA	NA	NA	NA	NA
chr8	27336446	27336446	T	G	UTR5	CHRNA2	NA	NA	NA	NA	NA
chr8	27879668	27879668	C	T	UTR3	NUGGC	NA	NA	NA	NA	NA
chr8	27879674	27879674	C	T	UTR3	NUGGC	NA	NA	NA	NA	NA
chr8	28203280	28203280	-	TTTT	UTR3	ZNF395	NA	NA	NA	NA	NA
chr8	28205777	28205778	TT	-	UTR3	ZNF395	NA	NA	NA	NA	NA
chr8	28205806	28205806	G	A	UTR3	ZNF395	NA	NA	NA	rs144407947	NA
chr8	28633328	28633328	A	C	exonic	INTS9	nonsynonymous SNV	3G:p.V483G,INTS9:NM_001172562:exon14:c.T1439G:p.V480G,INTS9:N	NA	NA	D
chr8	31024654	31024654	T	C	exonic	WRN	nonsynonymous SNV	WRN:NM_000553:exon34:c.T4099C:p.C1367R	NA	rs1346044	D
chr8	33356074	33356074	A	G	exonic	MAK16	nonsynonymous SNV	MAK16:NM_032509:exon10:c.A830G:p.Q277R	NA	rs6468171	B
chr8	33356191	33356191	-	T	UTR3	MAK16,TTI2	NA	NA	NA	NA	NA
chr8	33370396	33370396	A	-	UTR5	TTI2	NA	NA	NA	NA	NA
chr8	37636436	37636436	A	-	UTR3	PROSC	NA	NA	NA	NA	NA
chr8	38000824	38000825	TT	-	UTR3	STAR	NA	NA	NA	NA	NA
chr8	38033989	38033989	G	C	UTR5	LSM1	NA	NA	NA	rs2270376	NA
chr8	38034322	38034322	C	A	UTR5	BAG4	NA	NA	NA	NA	NA
chr8	38096451	38096451	-	TG	UTR3	DDHD2	NA	NA	NA	rs145118988	NA
chr8	38096587	38096587	G	A	UTR3	DDHD2	NA	NA	NA	rs10103315	NA
chr8	38281181	38281181	A	-	intronic	FGFR1	NA	NA	NA	NA	NA
chr8	38585785	38585785	G	A	UTR5	TACC1	NA	NA	NA	NA	NA
chr8	38585831	38585831	T	G	UTR5	TACC1	NA	NA	NA	NA	NA
chr8	39311600	39311600	C	T	ncRNA_exonic	ADAM3A	NA	NA	NA	rs7813010	NA
chr8	40438757	40438757	T	C	exonic	ZMAT4	nonsynonymous SNV	_001135731:exon5:c.A373G:p.T125A,ZMAT4:NM_024645:exon6:c.A6C	=COSM1179839;OCCURENCE=1(prostat	rs17851751	B
chr8	41399511	41399511	G	T	exonic	GINS4	nonsynonymous SNV	GINS4:NM_032336:exon8:c.G577T:p.D193Y	NA	NA	P
chr8	41503854	41503857	AAAG	-	UTR3	NKX6-3	NA	NA	NA	rs140821380	NA
chr8	41790809	41790809	A	G	exonic	KAT6A	synonymous SNV	P1643P,KAT6A:NM_001099412:exon18:c.T4929C:p.P1643P,KAT6A:NM	NA	NA	NA
chr8	42010466	42010466	A	G	UTR5	AP3M2	NA	NA	NA	rs6982888	NA
chr8	52732287	52732287	C	T	UTR3	PCMTD1	NA	NA	NA	rs75854138	NA
chr8	52732781	52732781	A	T	UTR3	PCMTD1	NA	NA	NA	rs72644314	NA
chr8	52732792	52732792	T	A	UTR3	PCMTD1	NA	NA	NA	rs111380744	NA
chr8	52732831	52732834	TCTC	-	UTR3	PCMTD1	NA	NA	NA	NA	NA
chr8	52732843	52732843	C	T	UTR3	PCMTD1	NA	NA	ID=COSN405080;OCCURENCE=1(lung)	rs116069210	NA
chr8	52732871	52732871	T	C	UTR3	PCMTD1	NA	NA	ID=COSN405081;OCCURENCE=1(lung)	rs77261625	NA
chr8	59363065	59363065	C	G	UTR3	UBXN2B	NA	NA	NA	NA	NA
chr8	63891662	63891662	C	A	ncRNA_exonic	UG0898H09	NA	NA	NA	NA	NA
chr8	69143713	69143714	TT	-	UTR3	PREX2	NA	NA	NA	rs148008471	NA
chr8	70745171	70745171	C	A	UTR5	SLCO5A1	NA	NA	NA	rs3750268	NA
chr8	71520608	71520608	-	A	UTR5	TRAM1	NA	NA	NA	rs111278931	NA
chr8	74235889	74235890	TT	-	UTR3	RDH10	NA	NA	NA	NA	NA
chr8	76476959	76476959	C	A	UTR3	HNF4G	NA	NA	NA	rs2941479	NA
chr8	76478991	76478991	G	A	UTR3	HNF4G	NA	NA	NA	rs11774375	NA
chr8	76479060	76479060	A	-	UTR3	HNF4G	NA	NA	NA	rs11309608	NA
chr8	77895047	77895047	A	-	UTR3	PEX2	NA	NA	NA	NA	NA
chr8	82665477	82665480	GTGA	-	splicing	CHMP4C	NA	NA	NA	rs137960856	NA
chr8	82671241	82671241	C	A	UTR3	CHMP4C	NA	NA	NA	NA	NA
chr8	82671242	82671242	T	A	UTR3	CHMP4C	NA	NA	NA	NA	NA
chr8	82671267	82671267	A	G	UTR3	CHMP4C	NA	NA	NA	NA	NA
chr8	82712242	82712242	-	AA	UTR3	SNX16	NA	NA	NA	NA	NA
chr8	87666251	87666251	T	G	exonic	CNGB3	nonsynonymous SNV	CNGB3:NM_019098:exon7:c.A892C:p.T298P	NA	rs4961206	B
chr8	90914272	90914272	A	G	UTR5	OSGIN2	NA	NA	NA	rs2250670	NA
chr8	92971674	92971674	G	A	UTR3	RUNX1T1	NA	NA	NA	rs57856280	NA
chr8	95158382	95158382	C	T	exonic	CDH17	synonymous SNV	01144663:exon15:c.G1941A:p.L647L,CDH17:NM_004063:exon15:c.G1	NA	rs1131830	NA
chr8	97244167	97244167	C	A	exonic	UQCRB	synonymous SNV	M_001254752:exon3:c.G93T:p.G31G,UQCRB:NM_006294:exon3:c.G9	NA	NA	NA
chr8	98741705	98741705	T	C	UTR3	MTDH	NA	NA	NA	rs1045444	NA
chr8	99956761	99956761	G	C	UTR5	OSR2	NA	NA	NA	rs4735586	NA
chr8	1.03E+08	1.03E+08	A	-	UTR3	NCALD	NA	NA	NA	NA	NA
chr8	1.04E+08	1.04E+08	C	G	exonic	ODF1	synonymous SNV	ODF1:NM_024410:exon2:c.C642G:p.P214P	NA	rs3018445	NA
chr8	1.04E+08	1.04E+08	G	A	exonic	ODF1	nonsynonymous SNV	ODF1:NM_024410:exon2:c.G647A:p.S216N	NA	rs2916569	NA
chr8	1.05E+08	1.05E+08	A	-	UTR3	RIMS2	NA	NA	NA	NA	NA
chr8	1.1E+08	1.1E+08	A	T	exonic	PKHD1L1	synonymous SNV	PKHD1L1:NM_177531:exon53:c.A9084T:p.T3028T	NA	rs1783148	NA
chr8	1.19E+08	1.19E+08	AAA	-	UTR3	SAMD12	NA	NA	NA	NA	NA
chr8	1.19E+08	1.19E+08	T	G	UTR3	SAMD12	NA	NA	NA	NA	NA
chr8	1.19E+08	1.19E+08	T	G	UTR3	SAMD12	NA	NA	NA	NA	NA
chr8	1.19E+08	1.19E+08	T	G	UTR3	SAMD12	NA	NA	NA	NA	NA

chr8	1.19E+08	1.19E+08	T	G	UTR3	SAMD12	NA	NA	NA	NA	NA
chr8	1.19E+08	1.19E+08	T	A	UTR3	SAMD12	NA	NA	NA	NA	NA
chr8	1.21E+08	1.21E+08	C	T	exonic	TAF2	synonymous SNV	TAF2:NM_003184:exon17:c.G2250A:p.Q750Q	NA	rs7002501	NA
chr8	1.21E+08	1.21E+08	C	G	exonic	TAF2	nonsynonymous SNV	TAF2:NM_003184:exon11:c.G1340C:p.S447T	)=COSM150523;OCCURENCE=1(stomac	rs9297605	B
chr8	1.21E+08	1.21E+08	G	A	UTR5	TAF2	NA	NA	NA	rs16893214	NA
chr8	1.23E+08	1.23E+08	G	C	ncRNA_exonic	HAS2-AS1	NA	NA	NA	rs2028506	NA
chr8	1.23E+08	1.23E+08	T	A	ncRNA_UTR5	HAS2	NA	NA	NA	rs3853253	NA
chr8	1.23E+08	1.23E+08	C	T	ncRNA_UTR5	HAS2	NA	NA	NA	NA	NA
chr8	1.24E+08	1.24E+08	-	T	UTR3	ZHX1	NA	NA	NA	NA	NA
chr8	1.26E+08	1.26E+08	C	T	UTR5	TRIB1	NA	NA	NA	rs3201475	NA
chr8	1.31E+08	1.31E+08	A	G	exonic	FAM49B	synonymous SNV	I_016623:exon8:c.T585C:p.T195T,FAM49B:NM_001256763:exon9:c.T5	NA	rs1051893	NA
chr8	1.31E+08	1.31E+08	A	C	ncRNA_exonic	ASAP1-IT1	NA	NA	NA	NA	NA
chr8	1.34E+08	1.34E+08	G	A	exonic	TG	nonsynonymous SNV	TG:NM_003235:exon44:c.G7589A:p.R2530Q	NA	rs1133076	B
chr8	1.4E+08	1.4E+08	G	A	UTR5	COL22A1	NA	NA	NA	NA	NA
chr8	1.42E+08	1.42E+08	A	G	exonic	SLC45A4	synonymous SNV	SLC45A4:NM_001080431:exon7:c.T2070C:p.G690G	NA	rs7017848	NA
chr8	1.42E+08	1.42E+08	T	C	exonic	SLC45A4	nonsynonymous SNV	SLC45A4:NM_001080431:exon7:c.A1999G:p.N667D	NA	rs3739238	B
chr8	1.42E+08	1.42E+08	T	C	ncRNA_exonic	LOC731779	NA	NA	NA	rs6987289	NA
chr8	1.42E+08	1.42E+08	G	A	exonic	MROH5	unknown	UNKNOWN	NA	rs12547980	NA
chr8	1.43E+08	1.43E+08	A	G	exonic	MROH5	unknown	UNKNOWN	OSN168449;OCCURENCE=1(large_intes	rs2613637	NA
chr8	1.43E+08	1.43E+08	T	C	exonic	TSNARE1	nonsynonymous SNV	TSNARE1:NM_145003:exon4:c.A352G:p.T118A	NA	rs10100935	B
chr8	1.44E+08	1.44E+08	G	A	UTR3	PSCA	NA	NA	NA	rs10216533	NA
chr8	1.44E+08	1.44E+08	G	A	UTR3	ZFP41	NA	NA	NA	rs34386291	NA
chr8	1.45E+08	1.45E+08	A	T	ncRNA_exonic	BREA2	NA	NA	NA	rs1141722	NA
chr8	1.45E+08	1.45E+08	CT	-	UTR3	FAM83H	NA	NA	NA	rs34283115	NA
chr8	1.45E+08	1.45E+08	C	T	UTR3	PARP10	NA	NA	NA	rs1134030	NA
chr8	1.45E+08	1.45E+08	G	T	UTR3	GRINA	NA	NA	NA	rs9100	NA
chr8	1.45E+08	1.45E+08	A	C	exonic	SPATC1	nonsynonymous SNV	_001134374:exon3:c.A979C:p.T327P,SPATC1:NM_198572:exon3:c.A9	NA	NA	D
chr8	1.45E+08	1.45E+08	T	C	exonic	SPATC1	nonsynonymous SNV	I_001134374:exon3:c.T985C:p.S329P,SPATC1:NM_198572:exon3:c.T9	NA	NA	P
chr8	1.46E+08	1.46E+08	-	A	UTR3	TONSL	NA	NA	NA	rs35298598	NA
chr8	1.46E+08	1.46E+08	T	G	UTR3	GPT	NA	NA	NA	NA	NA
chr8	1.46E+08	1.46E+08	C	G	UTR3	GPT	NA	NA	NA	NA	NA
chr8	1.46E+08	1.46E+08	T	G	UTR3	GPT	NA	NA	NA	NA	NA
chr8	1.46E+08	1.46E+08	G	A	ncRNA_exonic	TMED10P1	NA	NA	NA	NA	NA
chr8	1.46E+08	1.46E+08	C	T	UTR3	C8orf33	NA	NA	NA	rs6986329	NA
chr9	14710	14710	T	A	UTR3	WASH1	NA	NA	NA	NA	NA
chr9	14863	14863	A	G	exonic	WASH1	synonymous SNV	WASH1:NM_182905:exon11:c.T1342C:p.L448L	OSN405104,COSN405105;OCCURENCE=3	rs2974794	NA
chr9	163985	163985	A	G	exonic	CBWD1	synonymous SNV	83C:p.Y161Y,CBWD1:NM_018491:exon5:c.T483C:p.Y161Y,CBWD1:NM	NA	rs1127420	NA
chr9	214027	214027	G	T	UTR3	C9orf66	NA	NA	NA	NA	NA
chr9	2029199	2029199	G	A	exonic	SMARCA2	synonymous SNV	NM_003070:exon2:c.G177A:p.T59T,SMARCA2:NM_139045:exon2:c.G1	NA	rs10964471	NA
chr9	2039777	2039779	CAG	-	exonic	SMARCA2	nonframeshift deletion	:exon4:c.667_669del:p.223_223del,SMARCA2:NM_139045:exon4:c.66	NA	rs113070757	NA
chr9	4586213	4586213	-	TA	UTR3	SLC1A1	NA	NA	NA	rs150334575	NA
chr9	4622453	4622453	T	C	exonic	SPATA6L	nonsynonymous SNV	SPATA6L:NM_001039395:exon6:c.A553G:p.R185G	NA	rs10974657	P
chr9	4712080	4712080	A	G	UTR3	AK3	NA	NA	NA	NA	NA
chr9	6757661	6757661	C	A	UTR5	KDM4C	NA	NA	NA	NA	NA
chr9	12775861	12775861	-	GCGGCGG	exonic	LURAP1L	nonframeshift insertion	JRAP1L:NM_203403:exon1:c.147_148insGGCGGCGGC:p.G49delinsGGC	NA	rs139315731	NA
chr9	14993419	14993419	G	T	ncRNA_exonic	LOC389705	NA	NA	NA	rs4548277	NA
chr9	16412721	16412732	GAGAGAGA	-	UTR3	BNC2	NA	NA	NA	NA	NA
chr9	16413355	16413356	TT	-	UTR3	BNC2	NA	NA	NA	NA	NA
chr9	16414917	16414917	T	-	UTR3	BNC2	NA	NA	NA	NA	NA
chr9	16418871	16418871	-	CACACACA	UTR3	BNC2	NA	NA	NA	rs139614646	NA
chr9	17273731	17273731	A	G	exonic	CNTLN	nonsynonymous SNV	_001114395:exon6:c.A850G:p.T284A,CNTLN:NM_017738:exon6:c.A85	NA	rs3808795	B
chr9	19049596	19049596	C	A	UTR5	RRAGA	NA	NA	NA	rs2233802	NA
chr9	19054549	19054549	T	G	UTR3	HAUS6	NA	NA	NA	rs151216206	NA
chr9	19060197	19060197	A	T	exonic	HAUS6	nonsynonymous SNV	001270890:exon15:c.T1549A:p.S517T,HAUS6:NM_017645:exon15:c.T1	NA	rs41269003	D
chr9	20346360	20346360	G	A	UTR3	MLLT3	NA	NA	NA	NA	NA
chr9	27062666	27062666	G	A	exonic	IFT74	nonsynonymous SNV	A:p.V579M,IFT74:NM_001099223:exon20:c.G1735A:p.V579M,IFT74:N	NA	rs138591335	P
chr9	27205021	27205021	G	A	exonic	TEK	synonymous SNV	TEK:NM_000459:exon14:c.G2322A:p.R774R	NA	rs542913	NA
chr9	27284708	27284708	T	A	UTR3	EQTN	NA	NA	OSN168495;OCCURENCE=1(large_intes	rs1057524	NA
chr9	32553781	32553781	C	A	ncRNA_UTR3	NDUFB6	NA	NA	NA	NA	NA
chr9	33043991	33043993	AAA	-	UTR3	SMU1	NA	NA	NA	rs57341069	NA
chr9	33047211	33047211	A	-	UTR3	SMU1	NA	NA	NA	NA	NA
chr9	33111199	33111199	C	T	UTR3	B4GALT1	NA	NA	NA	rs74995636	NA
chr9	33111274	33111274	-	AAAAAAAA	UTR3	B4GALT1	NA	NA	NA	NA	NA
chr9	33384951	33384951	C	G	UTR3	AQP7	NA	NA	NA	rs75724774	NA
chr9	33384960	33384960	C	T	UTR3	AQP7	NA	NA	NA	rs76796426	NA
chr9	33384977	33384977	T	G	UTR3	AQP7	NA	NA	ID=COSN405112;OCCURENCE=1(lung)	rs13434	NA

chr9	33385047	33385047	G	A	exonic	AQP7	nonsynonymous SNV	AQP7:NM_001170:exon8:c.C985T:p.P329S	NA	rs78431873	B
chr9	33385709	33385709	C	T	exonic	AQP7	synonymous SNV	AQP7:NM_001170:exon7:c.G681A:p.P227P	NA	rs116294914	NA
chr9	33386465	33386465	A	G	exonic	AQP7	nonsynonymous SNV	AQP7:NM_001170:exon5:c.T343C:p.Y115H	NA	rs74668961	B
chr9	33402374	33402374	G	C	UTR5	AQP7	NA	NA	NA	NA	NA
chr9	34834937	34834937	A	G	ncRNA_exonic	FAM205B	NA	NA	OSM1108519;OCCURENCE=2(endometr	rs202203144	NA
chr9	35609148	35609148	A	C	exonic	TESK1	synonymous SNV	TESK1:NM_006285:exon10:c.A1290C:p.T430T	NA	NA	NA
chr9	35609172	35609172	A	C	exonic	TESK1	synonymous SNV	TESK1:NM_006285:exon10:c.A1314C:p.L438L	NA	NA	NA
chr9	35609179	35609179	T	C	exonic	TESK1	nonsynonymous SNV	TESK1:NM_006285:exon10:c.T1321C:p.S441P	NA	NA	D
chr9	36216667	36216672	TATTTA	-	UTR3	GNE	NA	NA	NA	rs201131813	NA
chr9	37778498	37778498	C	T	UTR3	TRMT10B	NA	NA	NA	rs45539136	NA
chr9	37863149	37863149	G	C	UTR3	DCAF10	NA	NA	NA	rs73449401	NA
chr9	37863196	37863196	A	G	UTR3	DCAF10	NA	NA	NA	rs112696997	NA
chr9	37864020	37864020	C	-	UTR3	DCAF10	NA	NA	NA	rs35009204	NA
chr9	37918485	37918486	TG	-	UTR3	SHB	NA	NA	NA	rs66479155	NA
chr9	38398153	38398153	A	T	UTR3	ALDH1B1	NA	NA	NA	rs74363930	NA
chr9	38409062	38409062	A	T	UTR3	IGFBPL1	NA	NA	NA	rs73646183	NA
chr9	41952522	41952522	T	A	ncRNA_exonic	MGC21881	NA	NA	NA	rs62539305	NA
chr9	41952549	41952549	G	C	ncRNA_exonic	MGC21881	NA	NA	NA	rs1054552	NA
chr9	41953020	41953020	T	C	ncRNA_exonic	MGC21881	NA	NA	NA	rs201318708	NA
chr9	41959519	41959519	A	G	ncRNA_exonic	KGFLP2	NA	NA	NA	rs62563796	NA
chr9	41960898	41960898	G	T	ncRNA_exonic	KGFLP2	NA	NA	NA	rs201761160	NA
chr9	43630712	43630712	A	G	UTR5	SPATA31A6	NA	NA	NA	rs62556735	NA
chr9	66499694	66499694	A	G	ncRNA_exonic	PTGER4P2-CDK2AP2P2	NA	NA	NA	rs111388370	NA
chr9	66499867	66499867	T	C	ncRNA_exonic	PTGER4P2-CDK2AP2P2	NA	NA	NA	rs7862419	NA
chr9	66501254	66501254	A	C	ncRNA_exonic	PTGER4P2-CDK2AP2P2	NA	NA	NA	rs10442087	NA
chr9	66501326	66501326	T	C	ncRNA_exonic	PTGER4P2-CDK2AP2P2	NA	NA	NA	rs28692535	NA
chr9	66501329	66501329	C	G	ncRNA_exonic	PTGER4P2-CDK2AP2P2	NA	NA	NA	rs201855807	NA
chr9	66501437	66501437	A	-	ncRNA_exonic	PTGER4P2-CDK2AP2P2	NA	NA	NA	NA	NA
chr9	66502241	66502241	G	A	ncRNA_exonic	PTGER4P2-CDK2AP2P2	NA	NA	NA	rs1492778	NA
chr9	66502640	66502640	A	G	ncRNA_exonic	PTGER4P2-CDK2AP2P2	NA	NA	NA	rs74961113	NA
chr9	67281729	67281729	G	A	ncRNA_exonic	AQP7P1	NA	NA	NA	rs9696090	NA
chr9	67281733	67281733	A	G	ncRNA_exonic	AQP7P1	NA	NA	NA	NA	NA
chr9	67281734	67281734	G	A	ncRNA_exonic	AQP7P1	NA	NA	NA	rs2321020	NA
chr9	69200873	69200873	G	A	exonic	FOXD4L6	nonsynonymous SNV	FOXD4L6:NM_001085476:exon1:c.C740T:p.P247L	NA	NA	D
chr9	69206924	69206924	T	A	exonic	CBWD6	nonsynonymous SNV	CBWD6:NM_001085457:exon12:c.A864T:p.E288D	NA	rs1127334	B
chr9	69238294	69238294	T	C	exonic	CBWD6	nonsynonymous SNV	CBWD6:NM_001085457:exon8:c.A598G:p.I200V	NA	NA	B
chr9	69416170	69416170	C	G	exonic	ANKRD20A4	nonsynonymous SNV	ANKRD20A4:NM_001098805:exon12:c.C1117G:p.Q373E	NA	rs199732379	B
chr9	69424030	69424030	T	C	exonic	ANKRD20A4	nonsynonymous SNV	ANKRD20A4:NM_001098805:exon15:c.T2326C:p.F776L	NA	rs78100933	B
chr9	69424049	69424049	G	A	exonic	ANKRD20A4	nonsynonymous SNV	ANKRD20A4:NM_001098805:exon15:c.G2345A:p.S782N	NA	rs200653869	B
chr9	69424057	69424057	C	G	exonic	ANKRD20A4	nonsynonymous SNV	ANKRD20A4:NM_001098805:exon15:c.C2353G:p.L785V	NA	NA	B
chr9	69424098	69424098	G	A	exonic	ANKRD20A4	synonymous SNV	ANKRD20A4:NM_001098805:exon15:c.G2394A:p.Q798Q	NA	NA	NA
chr9	69424114	69424114	A	G	exonic	ANKRD20A4	nonsynonymous SNV	ANKRD20A4:NM_001098805:exon15:c.A2410G:p.T804A	NA	rs4032009	B
chr9	69424120	69424120	G	A	exonic	ANKRD20A4	nonsynonymous SNV	ANKRD20A4:NM_001098805:exon15:c.G2416A:p.V806I	NA	NA	B
chr9	69424121	69424121	T	G	exonic	ANKRD20A4	nonsynonymous SNV	ANKRD20A4:NM_001098805:exon15:c.T2417G:p.V806G	NA	rs201002653	B
chr9	69424385	69424385	A	G	UTR3	ANKRD20A4	NA	NA	NA	rs4116477	NA
chr9	69663721	69663721	C	G	ncRNA_exonic	LOC100133920	NA	NA	NA	rs199847383	NA
chr9	69664229	69664229	C	A	ncRNA_exonic	LOC100133920	NA	NA	NA	rs200049495	NA
chr9	69664284	69664284	A	G	ncRNA_exonic	LOC100133920	NA	NA	NA	rs200802718	NA
chr9	69664315	69664315	G	A	ncRNA_exonic	LOC100133920	NA	NA	NA	rs4248773	NA
chr9	69664842	69664842	T	A	ncRNA_exonic	LOC100133920	NA	NA	NA	rs75601058	NA
chr9	70175856	70175856	C	T	UTR3	FOXD4L5	NA	NA	NA	rs187159666	NA
chr9	70900911	70900911	-	A	exonic	CBWD3	frameshift insertion	CBWD3:NM_201453:exon11:c.771_772insA:p.Q257fs	NA	NA	NA
chr9	70918189	70918189	A	T	exonic	FOXD4L3	stopgain SNV	FOXD4L3:NM_199135:exon1:c.A322T:p.K108X	NA	rs7034645	NA
chr9	70970401	70970401	T	C	ncRNA_exonic	PGM5-AS1	NA	NA	NA	NA	NA
chr9	72046058	72046059	AA	-	UTR3	APBA1	NA	NA	NA	NA	NA
chr9	73002115	73002115	-	T	UTR3	KLF9	NA	NA	NA	NA	NA
chr9	74298435	74298435	-	ACAC	UTR3	TMEM2	NA	NA	NA	NA	NA
chr9	77407636	77407636	C	T	exonic	TRPM6	synonymous SNV	A:p.Q809Q,TRPM6:NM_001177311:exon19:c.G2427A:p.Q809Q,TRPM6	NA	rs4145894	NA
chr9	77761920	77761920	T	-	UTR3	OSTF1	NA	NA	NA	rs5898396	NA
chr9	77761950	77761951	TT	-	UTR3	OSTF1	NA	NA	NA	NA	NA
chr9	78773958	78773958	A	C	exonic	PCSK5	nonsynonymous SNV	01190482:exon12:c.A1490C:p.N497T,PCSK5:NM_006200:exon12:c.A14	NA	NA	D
chr9	78808329	78808329	G	A	UTR3	PCSK5	NA	NA	NA	rs151056382	NA
chr9	78808329	78808329	-	AAAA	UTR3	PCSK5	NA	NA	NA	NA	NA
chr9	79465559	79465559	C	A	exonic	PRUNE2	nonsynonymous SNV	PRUNE2:NM_015225:exon3:c.G164T:p.C55F	NA	NA	NA
chr9	86153159	86153159	T	G	UTR5	FRMD3	NA	NA	NA	rs4529536	NA
chr9	86354511	86354511	G	C	UTR3	GKAP1	NA	NA	NA	rs4750	NA

chr9	86595070	86595070	C	T	UTR5	HNRNPK	NA	NA	NA	rs296887	NA
chr9	87285595	87285595	C	G	UTR5	NTRK2	NA	NA	NA	rs1187325	NA
chr9	87430433	87430433	-	T	UTR3	NTRK2	NA	NA	NA	NA	NA
chr9	88959938	88959938	C	T	exonic	ZCCHC6	synonymous SNV	51A:p.L317L,ZCCHC6:NM_001185074:exon5:c.G951A:p.L317L,ZCCHC6	NA	rs791323	NA
chr9	89763574	89763574	A	C	UTR5	C9orf170	NA	NA	NA	rs461126	NA
chr9	90323492	90323492	G	A	UTR3	DAPK1	NA	NA	NA	rs36220453	NA
chr9	94896220	94896220	-	CCCA	ncRNA_exonic	LOC100128076	NA	NA	NA	rs111380347	NA
chr9	95993326	95993326	T	C	exonic	WNK2	synonymous SNV	WNK2:NM_006648:exon3:c.T1011C:p.S337S	NA	rs11787888	NA
chr9	96026243	96026243	G	A	exonic	WNK2	synonymous SNV	WNK2:NM_006648:exon15:c.G3624A:p.T1208T	NA	rs55981123	NA
chr9	96080699	96080699	C	T	exonic	C9orf129	nonsynonymous SNV	C9orf129:NM_001098808:exon5:c.G572A:p.R191H	NA	rs36081907	NA
chr9	99264877	99264878	TT	-	UTR3	CDC14B	NA	NA	NA	rs138076839	NA
chr9	99702876	99702876	T	C	UTR3	NUTMG	NA	NA	NA	rs144789903	NA
chr9	1E+08	1E+08	G	A	ncRNA_exonic	00499484,LOC100499484-C9O	NA	NA	NA	rs7020002	NA
chr9	1E+08	1E+08	-	GAGGAG	exonic	CCDC180	nonframeshift insertion	CDC180:NM_020893:exon18:c.2325_2326insGAGGAG:p.E775delinsEE	NA	rs113264216	NA
chr9	1.01E+08	1.01E+08	G	C	UTR5	FOXE1	NA	NA	NA	rs7849497	NA
chr9	1.01E+08	1.01E+08	C	G	UTR3	HEMGN	NA	NA	NA	NA	NA
chr9	1.01E+08	1.01E+08	T	C	UTR3	CORO2A	NA	NA	NA	rs1746239	NA
chr9	1.02E+08	1.02E+08	A	T	exonic	GALNT12	nonsynonymous SNV	GALNT12:NM_024642:exon1:c.A356T:p.E119V	NA	rs1137654	P
chr9	1.05E+08	1.05E+08	C	T	UTR5	GRIN3A	NA	NA	NA	rs62577464	NA
chr9	1.12E+08	1.12E+08	G	A	exonic	TMEM245	synonymous SNV	TMEM245:NM_032012:exon3:c.C702T:p.S234S	NA	rs2271878	NA
chr9	1.12E+08	1.12E+08	G	T	exonic	EPB41L4B	nonsynonymous SNV	/_018424:exon3:c.C418A:p.L140M,EPB41L4B:NM_019114:exon3:c.C4	NA	NA	D
chr9	1.13E+08	1.13E+08	C	T	UTR3	PALM2	NA	NA	NA	rs10816907	NA
chr9	1.13E+08	1.13E+08	C	T	exonic	SVEP1	nonsynonymous SNV	SVEP1:NM_153366:exon7:c.G1519A:p.V507I	NA	rs872665	NA
chr9	1.14E+08	1.14E+08	A	G	UTR3	ZNF483	NA	NA	NA	rs7029060	NA
chr9	1.14E+08	1.14E+08	G	C	UTR5	PTGR1	NA	NA	NA	rs41279067	NA
chr9	1.14E+08	1.14E+08	G	C	UTR5	PTGR1	NA	NA	NA	rs41279069	NA
chr9	1.14E+08	1.14E+08	A	G	exonic	C9orf84	nonsynonymous SNV	001080551:exon20:c.T2786C:p.L929P,C9orf84:NM_173521:exon22:c.T	NA	rs6477845	B
chr9	1.14E+08	1.14E+08	A	C	exonic	C9orf84	nonsynonymous SNV	01080551:exon16:c.T2310G:p.N770K,C9orf84:NM_173521:exon18:c.T	NA	rs7036568	B
chr9	1.16E+08	1.16E+08	GT	-	UTR3	SNX30	NA	NA	NA	NA	NA
chr9	1.16E+08	1.16E+08	TT	-	UTR3	SNX30	NA	NA	NA	rs138062484	NA
chr9	1.16E+08	1.16E+08	\GTGTGCTC	-	UTR5	SLC46A2	NA	NA	NA	rs200371278	NA
chr9	1.16E+08	1.16E+08	C	A	UTR5	ZNF883	NA	NA	NA	rs1324934	NA
chr9	1.16E+08	1.16E+08	C	T	ncRNA_exonic	FAM225A	NA	NA	NA	rs62576060	NA
chr9	1.16E+08	1.16E+08	T	-	UTR3	RNF183	NA	NA	NA	NA	NA
chr9	1.17E+08	1.17E+08	CA	-	UTR3	ZNF618	NA	NA	NA	rs147537659	NA
chr9	1.17E+08	1.17E+08	A	G	exonic	AKNA	nonsynonymous SNV	AKNA:NM_030767:exon21:c.T3907C:p.S1303P	NA	rs2250242	B
chr9	1.17E+08	1.17E+08	A	G	exonic	DFNB31	synonymous SNV	C:p.G68G,DFNB31:NM_001173425:exon6:c.T1353C:p.G451G,DFNB31	NA	rs4979387	NA
chr9	1.18E+08	1.18E+08	T	C	UTR3	TNFSF8	NA	NA	NA	rs3181370	NA
chr9	1.18E+08	1.18E+08	G	A	UTR3	TNFSF8	NA	NA	NA	rs3181369	NA
chr9	1.18E+08	1.18E+08	C	T	exonic	TNC	nonsynonymous SNV	TNC:NM_002160:exon19:c.G5341A:p.A1781T	NA	rs2274750	P
chr9	1.19E+08	1.19E+08	-	AA	UTR3	PAPPA	NA	NA	NA	NA	NA
chr9	1.23E+08	1.23E+08	C	G	exonic	CDK5RAP2	nonsynonymous SNV	).V1310L,CDK5RAP2:NM_001011649:exon31:c.G4618C:p.V1540L,CDK5	NA	rs4837768	B
chr9	1.26E+08	1.26E+08	-	TA	UTR3	RABGAP1	NA	NA	NA	rs112214533	NA
chr9	1.26E+08	1.26E+08	G	C	exonic	CRB2	nonsynonymous SNV	CRB2:NM_173689:exon3:c.G476C:p.G159A	NA	rs1105222	D
chr9	1.26E+08	1.26E+08	G	T	exonic	DENND1A	nonsynonymous SNV	DENND1A:NM_024820:exon21:c.C1623A:p.S541R	NA	NA	NA
chr9	1.31E+08	1.31E+08	A	G	UTR3	AK1	NA	NA	NA	NA	NA
chr9	1.31E+08	1.31E+08	A	G	intronic	SLC25A25	NA	NA	NA	rs947623	NA
chr9	1.31E+08	1.31E+08	G	A	UTR5	PTGES2	NA	NA	NA	rs10819365	NA
chr9	1.31E+08	1.31E+08	-	GTGCGCGC	UTR5	PTGES2	NA	NA	NA	rs11283643	NA
chr9	1.31E+08	1.31E+08	A	C	exonic	CERCAM	nonsynonymous SNV	CERCAM:NM_016174:exon5:c.A602C:p.N201T	NA	rs75750764	B
chr9	1.32E+08	1.32E+08	T	C	exonic	TBC1D13	nonsynonymous SNV	TBC1D13:NM_018201:exon8:c.T569C:p.V190A	NA	rs1572912	B
chr9	1.32E+08	1.32E+08	C	A	UTR5	PHYHD1	NA	NA	NA	rs7049155	NA
chr9	1.32E+08	1.32E+08	A	C	exonic	FAM73B	nonsynonymous SNV	FAM73B:NM_032809:exon9:c.A923C:p.Y308S	NA	NA	B
chr9	1.32E+08	1.32E+08	G	T	UTR3	PPP2R4	NA	NA	NA	rs746489	NA
chr9	1.32E+08	1.32E+08	G	C	UTR3	PPP2R4	NA	NA	NA	rs913273	NA
chr9	1.32E+08	1.32E+08	T	G	UTR3	PPP2R4	NA	NA	NA	NA	NA
chr9	1.32E+08	1.32E+08	T	G	UTR3	PPP2R4	NA	NA	NA	NA	NA
chr9	1.32E+08	1.32E+08	C	G	UTR3	PPP2R4	NA	NA	NA	rs17459361	NA
chr9	1.33E+08	1.33E+08	AC	-	UTR3	PTGES	NA	NA	NA	NA	NA
chr9	1.33E+08	1.33E+08	T	G	UTR3	TOR1B	NA	NA	NA	rs1220509	NA
chr9	1.33E+08	1.33E+08	T	G	UTR3	TOR1B	NA	NA	NA	NA	NA
chr9	1.33E+08	1.33E+08	C	T	UTR3	C9orf78	NA	NA	NA	NA	NA
chr9	1.33E+08	1.33E+08	G	A	ncRNA_exonic	LOC100272217	NA	NA	NA	rs4740351	NA
chr9	1.35E+08	1.35E+08	G	T	UTR3	SETX	NA	NA	NA	rs11787894	NA
chr9	1.36E+08	1.36E+08	C	T	exonic	RALGDS	synonymous SNV	iA:p.V108V,RALGDS:NM_001271775:exon3:c.G372A:p.V124V,RALGDS	NA	rs3761824	NA
chr9	1.36E+08	1.36E+08	A	G	UTR3	ABO	NA	NA	NA	NA	NA

chr9	1.36E+08	1.36E+08	T	G	UTR3	ABO	NA	NA	NA	rs7469752	NA
chr9	1.36E+08	1.36E+08	TGTGTG	-	UTR3	CACFD1	NA	NA	NA	NA	NA
chr9	1.36E+08	1.36E+08	C	T	exonic	ADAMTSL2	synonymous SNV	01145320:exon17:c.C2436T:p.R812R,ADAMTSL2:NM_014694:exon17:	NA	rs147931149	NA
chr9	1.37E+08	1.37E+08	T	C	UTR3	VAV2	NA	NA	NA	NA	NA
chr9	1.37E+08	1.37E+08	C	-	ncRNA_exonic	LINC00094	NA	NA	NA	NA	NA
chr9	1.37E+08	1.37E+08	T	G	UTR3	RXRA	NA	NA	NA	NA	NA
chr9	1.37E+08	1.37E+08	C	G	UTR3	RXRA	NA	NA	NA	NA	NA
chr9	1.38E+08	1.38E+08	T	C	exonic	FCN2	synonymous SNV	NM_015837:exon2:c.T108C:p.R36R,FCN2:NM_004108:exon3:c.T222C:|	NA	rs4520243	NA
chr9	1.38E+08	1.38E+08	-	C	UTR3	OBP2A	NA	NA	NA	rs112851559	NA
chr9	1.39E+08	1.39E+08	ACACAC	-	UTR3	QSOX2	NA	NA	NA	NA	NA
chr9	1.39E+08	1.39E+08	T	G	exonic	SNAPC4	nonsynonymous SNV	SNAPC4:NM_003086:exon17:c.A2245C:p.T749P	NA	NA	P
chr9	1.4E+08	1.4E+08	C	G	exonic	KIAA1984	nonsynonymous SNV	KIAA1984:NM_001039374:exon10:c.C1073G:p.A358G	NA	NA	P
chr9	1.4E+08	1.4E+08	A	G	ncRNA_exonic	LOC100289341	NA	NA	NA	NA	NA
chr9	1.4E+08	1.4E+08	C	G	ncRNA_exonic	LOC100289341	NA	NA	NA	NA	NA
chr9	1.4E+08	1.4E+08	-	CCG	UTR5	TUBB4B	NA	NA	NA	NA	NA
chr9	1.4E+08	1.4E+08	-	G	UTR3	NRARP	NA	NA	NA	rs34679617	NA
chr9	1.41E+08	1.41E+08	A	-	splicing	exon1:c.284+1A>-,NM_00124	NA	NA	NA	NA	NA
chr9	1.41E+08	1.41E+08	TGTG	-	UTR3	CACNA1B	NA	NA	NA	rs72450095	NA
chr9	1.41E+08	1.41E+08	A	C	ncRNA_exonic	TUBBP5	NA	NA	D=COSM487273;OCCURENCE=1(kidney	rs62581043	NA
chr9	1.41E+08	1.41E+08	A	G	ncRNA_exonic	TUBBP5	NA	NA	NA	rs184986292	NA
chr10	858482	858482	A	-	UTR3	LARP4B	NA	NA	NA	NA	NA
chr10	1102868	1102868	T	C	UTR5	WDR37	NA	NA	NA	rs10047382	NA
chr10	1205784	1205784	G	A	ncRNA_exonic	LINC00200	NA	NA	NA	rs12762051	NA
chr10	1205788	1205788	C	A	ncRNA_exonic	LINC00200	NA	NA	NA	NA	NA
chr10	1205792	1205792	G	A	ncRNA_exonic	LINC00200	NA	NA	NA	rs12762059	NA
chr10	1205801	1205801	C	A	ncRNA_exonic	LINC00200	NA	NA	NA	rs12763661	NA
chr10	1228330	1228330	G	T	UTR3	ADARB2	NA	NA	NA	rs1129226	NA
chr10	3172121	3172121	C	T	exonic	PFKP	synonymous SNV	02627:exon17:c.C1794T:p.A598A,PFKP:NM_001242339:exon19:c.C17	NA	rs1052333	NA
chr10	3172145	3172145	C	T	exonic	PFKP	synonymous SNV	002627:exon17:c.C1818T:p.F606F,PFKP:NM_001242339:exon19:c.C17	NA	rs1052337	NA
chr10	3178965	3178968	AATT	-	UTR3	PFKP	NA	NA	NA	rs151101324	NA
chr10	3178986	3178986	T	C	UTR3	PFKP	NA	NA	NA	rs3816712	NA
chr10	3178987	3178987	G	A	UTR3	PFKP	NA	NA	NA	rs2388564	NA
chr10	5014488	5014488	C	T	exonic	AKR1C1	synonymous SNV	AKR1C1:NM_001353:exon6:c.C666T:p.H222H	NA	rs13222	NA
chr10	5903837	5903837	A	G	UTR3	ANKRD16	NA	NA	NA	rs67091121	NA
chr10	7285632	7285632	A	G	exonic	SFMBT2	synonymous SNV	01018039:exon9:c.T1008C:p.D336D,SFMBT2:NM_001029880:exon9:c.T	NA	rs2248474	NA
chr10	7769054	7769054	T	C	exonic	ITIH2	nonsynonymous SNV	ITIH2:NM_002216:exon10:c.T1126C:p.Y376H	NA	NA	D
chr10	7780655	7780655	G	A	exonic	ITIH2	nonsynonymous SNV	ITIH2:NM_002216:exon16:c.G2029A:p.V677M	NA	rs188916946	B
chr10	16547058	16547058	A	G	exonic	PTER	synonymous SNV	001261836:exon4:c.A738G:p.Q246Q,PTER:NM_001001484:exon5:c.A7	NA	rs7904014	NA
chr10	17088006	17088006	T	C	exonic	CUBN	synonymous SNV	CUBN:NM_001081:exon24:c.A3417G:p.L1139L	NA	rs1801228	NA
chr10	17130199	17130199	G	A	exonic	CUBN	synonymous SNV	CUBN:NM_001081:exon15:c.C1911T:p.L637L	NA	rs41289311	NA
chr10	17270264	17270264	C	T	UTR5	VIM	NA	NA	NA	NA	NA
chr10	17839374	17839374	C	A	UTR3	TMEM236	NA	NA	NA	NA	NA
chr10	18242311	18242311	A	G	exonic	SLC39A12	nonsynonymous SNV	M_001145195:exon2:c.A106G:p.S36G,SLC39A12:NM_152725:exon2:c.A	NA	rs10764176	B
chr10	18266989	18266989	G	A	exonic	SLC39A12	nonsynonymous SNV	_001145195:exon5:c.G910A:p.V304I,SLC39A12:NM_152725:exon5:c.G	NA	rs2478568	NA
chr10	21803143	21803149	AAAAAAA	-	UTR3	SKIDA1	NA	NA	NA	NA	NA
chr10	21803848	21803848	-	A	UTR3	SKIDA1	NA	NA	NA	NA	NA
chr10	21807780	21807780	T	-	UTR5	SKIDA1	NA	NA	NA	rs112940679	NA
chr10	27224492	27224492	G	C	ncRNA_exonic	LINC00202-1	NA	NA	NA	rs11015390	NA
chr10	30599511	30599511	A	-	UTR3	MTPAP	NA	NA	NA	rs11310410	NA
chr10	32580205	32580205	C	T	exonic	EPC1	nonsynonymous SNV	61A:p.M287I,EPC1:NM_001272019:exon6:c.G648A:p.M216I,EPC1:NM	NA	rs72789797	P
chr10	33165422	33165422	G	A	UTR3	C10orf68	NA	NA	NA	rs12217414	NA
chr10	33214802	33214802	A	G	exonic	ITGB1	synonymous SNV	83C:p.C261C,ITGB1:NM_002211:exon6:c.T783C:p.C261C,ITGB1:NM_1	NA	rs2298141	NA
chr10	35772402	35772402	G	A	exonic	CCNY	synonymous SNV	NM_145012:exon2:c.G225A:p.T75T,CCNY:NM_181698:exon4:c.G63A:|	NA	rs3802509	NA
chr10	35896957	35896957	G	A	exonic	GJD4	synonymous SNV	GJD4:NM_153368:exon2:c.G516A:p.K172K	NA	rs595652	NA
chr10	42832213	42832213	T	C	ncRNA_exonic	LOC441666	NA	NA	NA	NA	NA
chr10	42832258	42832258	C	T	ncRNA_exonic	LOC441666	NA	NA	NA	rs73253652	NA
chr10	43010627	43010627	C	T	ncRNA_exonic	ZNF37BP	NA	NA	NA	rs210233	NA
chr10	43014380	43014380	A	-	ncRNA_exonic	ZNF37BP	NA	NA	NA	NA	NA
chr10	43327873	43327873	A	-	UTR3	BMS1	NA	NA	NA	rs112489895	NA
chr10	44284919	44284919	A	C	ncRNA_exonic	HNRNPA3P1	NA	NA	NA	NA	NA
chr10	44284934	44284934	A	C	ncRNA_exonic	HNRNPA3P1	NA	NA	NA	NA	NA
chr10	45649320	45649320	A	G	ncRNA_exonic	RSU1P2	NA	NA	NA	rs7478624	NA
chr10	46254783	46254783	A	G	exonic	FAM21C	synonymous SNV	G:p.K499K,FAM21C:NM_001169106:exon17:c.A1569G:p.K523K,FAM2	NA	rs186891138	NA
chr10	46958953	46958953	A	-	UTR3	SYT15	NA	NA	NA	rs111970300	NA
chr10	47754794	47754794	C	T	exonic	ANXA8L2	nonsynonymous SNV	ANXA8L2:NM_001630:exon5:c.C401T:p.A134V	NA	rs200976247	D
chr10	47915898	47915898	A	G	exonic	FAM21B	synonymous SNV	FAM21B:NM_018232:exon15:c.A1305G:p.K435K	)=COSM146931;OCCURENCE=1(stomac	rs183064568	NA

chr10	47919971	47919971	G	C	exonic	FAM21B	nonsynonymous SNV	FAM21B:NM_018232:exon16:c.G1401C:p.K467N	NA	rs16914400	NA
chr10	47929847	47929847	C	G	exonic	FAM21B	nonsynonymous SNV	FAM21B:NM_018232:exon19:c.C1828G:p.R610G	NA	rs2610489	NA
chr10	50029121	50029121	C	T	exonic	WDFY4	synonymous SNV	WDFY4:NM_020945:exon34:c.C5724T:p.H1908H	NA	rs79406124	NA
chr10	50117863	50117863	A	G	UTR3	LRRC18	NA	NA	NA	rs3954099	NA
chr10	50822192	50822192	G	C	UTR5	CHAT	NA	NA	NA	rs7903315	NA
chr10	50830171	50830171	C	T	exonic	CHAT	nonsynonymous SNV	73T:p.L125F,CHAT:NM_020986:exon5:c.C373T:p.L125F,CHAT:NM_001	NA	rs8178990	P
chr10	51486325	51486325	T	C	UTR5	AGAP7	NA	NA	NA	rs113081690	NA
chr10	60145225	60145225	T	G	UTR5	TFAM	NA	NA	NA	NA	NA
chr10	63955374	63955381	GAAGGAA	-	UTR3	RTKN2	NA	NA	NA	NA	NA
chr10	65225899	65225899	-	GGCGGC	ncRNA_exonic	JMJD1C-AS1	NA	NA	NA	rs3841602	NA
chr10	70103564	70103564	-	T	UTR3	RUFY2	NA	NA	NA	rs139789920	NA
chr10	70103597	70103597	C	A	UTR3	RUFY2	NA	NA	NA	rs1162754	NA
chr10	70166913	70166913	T	G	UTR5	RUFY2	NA	NA	NA	rs59831114	NA
chr10	70743494	70743494	T	A	UTR3	DDX21	NA	NA	NA	NA	NA
chr10	70743515	70743515	C	A	UTR3	DDX21	NA	NA	NA	NA	NA
chr10	72187716	72187717	TT	-	UTR3	EIF4EBP2	NA	NA	NA	NA	NA
chr10	72307101	72307101	C	T	exonic	PALD1	nonsynonymous SNV	PALD1:NM_014431:exon18:c.C2161T:p.R721C	NA	rs3740447	D
chr10	72357600	72357600	A	-	UTR3	PRF1	NA	NA	NA	rs34914326	NA
chr10	72536921	72536921	A	G	exonic	TBATA	synonymous SNV	TBATA:NM_152710:exon7:c.T678C:p.D226D	NA	rs151019808	NA
chr10	73050759	73050759	T	G	exonic	UNC5B	nonsynonymous SNV	001244889:exon8:c.T1154G:p.V385G,UNC5B:NM_170744:exon9:c.T11	NA	rs117156661	P
chr10	73059788	73059788	-	TG	UTR3	UNC5B	NA	NA	NA	NA	NA
chr10	73848106	73848106	G	-	UTR5	SPOCK2	NA	NA	NA	rs10713496	NA
chr10	73992853	73992853	A	G	exonic	ANAPC16	synonymous SNV	001242547:exon4:c.A312G:p.G104G,ANAPC16:NM_173473:exon4:c.A	NA	NA	NA
chr10	73992863	73992863	T	C	exonic	ANAPC16	nonsynonymous SNV	_001242547:exon4:c.T322C:p.S108P,ANAPC16:NM_173473:exon4:c.T	NA	NA	P
chr10	75879769	75879769	-	AA	UTR3	VCL	NA	NA	NA	NA	NA
chr10	77158032	77158032	G	T	UTR3	ZNF503	NA	NA	NA	NA	NA
chr10	77160058	77160058	G	A	exonic	ZNF503	synonymous SNV	ZNF503:NM_032772:exon2:c.C390T:p.P130P	NA	rs72807457	NA
chr10	79551102	79551102	A	-	UTR3	DLG5	NA	NA	NA	NA	NA
chr10	81114813	81114813	C	G	UTR3	PPIF	NA	NA	NA	rs8837	NA
chr10	81273879	81273879	A	G	UTR3	EIF5AL1	NA	NA	NA	rs1250786	NA
chr10	81374261	81374261	C	G	UTR3	SFTPA1	NA	NA	NA	rs4253530	NA
chr10	81374262	81374262	T	C	UTR3	SFTPA1	NA	NA	NA	rs4253531	NA
chr10	82049251	82049251	-	TTCTTC	UTR5	MAT1A	NA	NA	NA	rs145126070	NA
chr10	82126808	82126808	C	G	UTR3	DYDC2	NA	NA	NA	rs1047952	NA
chr10	82192125	82192125	A	G	UTR3	FAM213A	NA	NA	NA	rs1063161	NA
chr10	82281978	82281978	T	C	UTR3	TSPAN14	NA	NA	NA	rs1878034	NA
chr10	88441223	88441223	G	A	exonic	LDB3	nonsynonymous SNV	001171611:exon4:c.G352A:p.V118M,LDB3:NM_007078:exon4:c.G352	NA	rs35507268	NA
chr10	88494810	88494813	ACAC	-	UTR3	LDB3	NA	NA	NA	NA	NA
chr10	88810077	88810077	A	-	UTR3	GLUD1	NA	NA	NA	NA	NA
chr10	88988555	88988555	C	T	exonic	NUTM2A	synonymous SNV	NUTM2A:NM_001099338:exon2:c.C918T:p.H306H	NA	rs200884340	NA
chr10	88992622	88992622	C	T	exonic	NUTM2A	synonymous SNV	NUTM2A:NM_001099338:exon5:c.C1614T:p.P538P	NA	rs200484233	NA
chr10	88992644	88992644	G	A	exonic	NUTM2A	nonsynonymous SNV	NUTM2A:NM_001099338:exon5:c.G1636A:p.E546K	NA	rs199899922	NA
chr10	88994452	88994452	G	A	exonic	NUTM2A	nonsynonymous SNV	NUTM2A:NM_001099338:exon7:c.G2624A:p.R875H	NA	rs199530790	NA
chr10	89124840	89124840	C	T	ncRNA_exonic	NUTM2D	NA	NA	NA	rs200082791	NA
chr10	89124862	89124862	G	A	ncRNA_exonic	NUTM2D	NA	NA	NA	rs200907148	B
chr10	89127510	89127523	GGACTGG	-	ncRNA_exonic	NUTM2D	NA	NA	NA	NA	NA
chr10	89128071	89128071	C	T	ncRNA_exonic	NUTM2D	NA	NA	NA	rs71237016	NA
chr10	89506941	89506941	A	-	UTR3	PAPSS2	NA	NA	NA	NA	NA
chr10	89619792	89619792	G	A	UTR3	KLLN	NA	NA	NA	rs113730541	NA
chr10	90585819	90585819	G	A	exonic	ANKRD22	synonymous SNV	ANKRD22:NM_144590:exon4:c.C375T:p.G125G	NA	rs7893917	NA
chr10	92660898	92660898	T	-	UTR3	RPP30	NA	NA	NA	rs59418716	NA
chr10	92661579	92661579	T	-	UTR3	RPP30	NA	NA	NA	rs58754872	NA
chr10	92672048	92672048	-	AA	UTR3	ANKRD1	NA	NA	NA	NA	NA
chr10	94111658	94111658	-	GTGTGTGT	UTR3	5-Mar	NA	NA	NA	NA	NA
chr10	99371776	99371776	C	T	UTR3	HOGA1	NA	NA	NA	rs114372258	NA
chr10	1.01E+08	1.01E+08	G	A	UTR5	COX15	NA	NA	NA	rs2231675	NA
chr10	1.02E+08	1.02E+08	C	T	exonic	PKD2L1	nonsynonymous SNV	_001253837:exon6:c.G1036A:p.V346I,PKD2L1:NM_016112:exon6:c.G1	NA	rs2278842	P
chr10	1.02E+08	1.02E+08	T	-	UTR3	SCD	NA	NA	NA	rs201898361	NA
chr10	1.02E+08	1.02E+08	T	-	UTR3	HIF1AN	NA	NA	NA	NA	NA
chr10	1.03E+08	1.03E+08	T	G	UTR5	C10orf2	NA	NA	NA	NA	NA
chr10	1.03E+08	1.03E+08	T	-	UTR3	SFXN3	NA	NA	NA	rs66614203	NA
chr10	1.04E+08	1.04E+08	T	-	UTR3	TMEM180	NA	NA	NA	NA	NA
chr10	1.05E+08	1.05E+08	-	ATC	UTR3	CALHM1	NA	NA	NA	NA	NA
chr10	1.12E+08	1.12E+08	AA	-	UTR3	ADD3	NA	NA	NA	rs57430745	NA
chr10	1.13E+08	1.13E+08	T	-	UTR3	SHOC2	NA	NA	NA	NA	NA
chr10	1.14E+08	1.14E+08	T	C	exonic	GPAM	nonsynonymous SNV	M_001244949:exon4:c.A127G:p.I43V,GPAM:NM_020918:exon4:c.A12	NA	rs2792751	B

chr10	1.14E+08	1.14E+08	A	G	ncRNA_exonic	GUCY2GP	NA	NA	NA	rs1887136	NA
chr10	1.15E+08	1.15E+08	A	C	intergenic	A(dist=36524),TCF7L2(dist=94	NA	NA	NA	NA	NA
chr10	1.15E+08	1.15E+08	C	-	UTR5	TCF7L2	NA	NA	NA	rs113102683	NA
chr10	1.16E+08	1.16E+08	-	A	UTR3	PLEKHS1	NA	NA	NA	rs142169841	NA
chr10	1.16E+08	1.16E+08	A	C	UTR3	PLEKHS1	NA	NA	NA	NA	NA
chr10	1.16E+08	1.16E+08	CC	-	UTR3	PLEKHS1	NA	NA	NA	NA	NA
chr10	1.16E+08	1.16E+08	TCCCCAACA	-	UTR3	PLEKHS1	NA	NA	NA	NA	NA
chr10	1.16E+08	1.16E+08	TTTTT	-	UTR3	ADRB1	NA	NA	NA	rs61645383	NA
chr10	1.16E+08	1.16E+08	A	-	UTR3	TDRD1	NA	NA	NA	rs11316882	NA
chr10	1.16E+08	1.16E+08	G	A	UTR3	AFAP1L2	NA	NA	NA	NA	NA
chr10	1.16E+08	1.16E+08	C	G	UTR5	ABLIM1	NA	NA	NA	rs141394498	NA
chr10	1.17E+08	1.17E+08	T	C	exonic	FAM160B1	synonymous SNV	001135051:exon14:c.T1893C:p.F631F,FAM160B1:NM_020940:exon14:	NA	rs3180654	NA
chr10	1.18E+08	1.18E+08	-	TT	UTR3	GFRA1	NA	NA	NA	NA	NA
chr10	1.18E+08	1.18E+08	A	-	UTR3	GFRA1	NA	NA	NA	rs144589291	NA
chr10	1.18E+08	1.18E+08	-	G	exonic	PNLIPRP2	unknown	UNKNOWN	NA	NA	NA
chr10	1.18E+08	1.18E+08	A	G	UTR3	HSPA12A	NA	NA	NA	rs7084566	NA
chr10	1.19E+08	1.19E+08	-	GT	UTR3	VAX1	NA	NA	NA	rs139596848	NA
chr10	1.19E+08	1.19E+08	C	A	exonic	VAX1	nonsynonymous SNV	:NM_001112704:exon1:c.G3T:p.M1I,VAX1:NM_199131:exon1:c.G3T:p	NA	NA	D
chr10	1.19E+08	1.19E+08	G	A	UTR5	VAX1	NA	NA	NA	NA	NA
chr10	1.19E+08	1.19E+08	C	A	UTR5	VAX1	NA	NA	NA	NA	NA
chr10	1.19E+08	1.19E+08	C	A	UTR5	VAX1	NA	NA	NA	NA	NA
chr10	1.19E+08	1.19E+08	GC	-	UTR5	VAX1	NA	NA	NA	rs71677026	NA
chr10	1.19E+08	1.19E+08	CG	-	UTR5	VAX1	NA	NA	NA	rs148359585	NA
chr10	1.19E+08	1.19E+08	T	C	ncRNA_exonic	EMX2OS	NA	NA	NA	rs2768325	NA
chr10	1.19E+08	1.19E+08	-	GTGT	ncRNA_exonic	EMX2OS	NA	NA	NA	rs111365239	NA
chr10	1.19E+08	1.19E+08	C	G	ncRNA_exonic	EMX2OS	NA	NA	NA	rs2768330	NA
chr10	1.2E+08	1.2E+08	-	A	UTR3	RAB11FIP2	NA	NA	NA	NA	NA
chr10	1.2E+08	1.2E+08	G	-	UTR5	RAB11FIP2	NA	NA	NA	rs67350544	NA
chr10	1.21E+08	1.21E+08	A	-	UTR3	GRK5	NA	NA	NA	NA	NA
chr10	1.24E+08	1.24E+08	G	T	exonic	TACC2	synonymous SNV	206860:exon8:c.G2121T:p.A707A,TACC2:NM_206861:exon11:c.G2325	NA	NA	NA
chr10	1.27E+08	1.27E+08	-	T	UTR3	ZRANB1	NA	NA	NA	rs200438486	NA
chr10	1.27E+08	1.27E+08	G	A	UTR3	CTBP2	NA	NA	NA	rs74874473	NA
chr10	1.27E+08	1.27E+08	G	A	UTR3	CTBP2	NA	NA	NA	rs1058493	NA
chr10	1.27E+08	1.27E+08	A	G	UTR3	CTBP2	NA	NA	NA	rs3208591	NA
chr10	1.27E+08	1.27E+08	T	G	UTR3	CTBP2	NA	NA	NA	rs3208590	NA
chr10	1.27E+08	1.27E+08	T	A	UTR3	CTBP2	NA	NA	NA	NA	NA
chr10	1.27E+08	1.27E+08	G	C	exonic	CTBP2	nonsynonymous SNV	7G:p.N789K,CTBP2:NM_001083914:exon7:c.C747G:p.N249K,CTBP2:NM	D=COSM213712;OCCURENCE=1(breast	rs61870306	D
chr10	1.27E+08	1.27E+08	T	C	exonic	CTBP2	nonsynonymous SNV	1G:p.N784S,CTBP2:NM_001083914:exon7:c.A731G:p.N244S,CTBP2:NM	NA	rs61870307	B
chr10	1.27E+08	1.27E+08	A	C	exonic	CTBP2	nonsynonymous SNV	G:p.L772W,CTBP2:NM_001083914:exon7:c.T695G:p.L232W,CTBP2:NM	NA	rs79936509	D
chr10	1.27E+08	1.27E+08	C	T	exonic	CTBP2	nonsynonymous SNV	A:p.V763M,CTBP2:NM_001083914:exon7:c.G667A:p.V223M,CTBP2:NM	NA	rs76203768	P
chr10	1.27E+08	1.27E+08	C	T	exonic	CTBP2	nonsynonymous SNV	6A:p.R759Q,CTBP2:NM_001083914:exon7:c.G656A:p.R219Q,CTBP2:NM	NA	rs75420260	D
chr10	1.27E+08	1.27E+08	C	A	exonic	CTBP2	nonsynonymous SNV	8T:p.D750Y,CTBP2:NM_001083914:exon7:c.G628T:p.D210Y,CTBP2:NM	NA	rs78681531	D
chr10	1.27E+08	1.27E+08	G	C	exonic	CTBP2	nonsynonymous SNV	CTBP2:NM_022802:exon1:c.C1615G:p.Q539E	NA	rs2946994	P
chr10	1.27E+08	1.27E+08	A	G	exonic	CTBP2	nonsynonymous SNV	CTBP2:NM_022802:exon1:c.T1363C:p.Y455H	NA	rs3012075	D
chr10	1.29E+08	1.29E+08	C	A	UTR3	FAM196A	NA	NA	NA	rs74687417	NA
chr10	1.3E+08	1.3E+08	T	C	UTR3	FOXI2	NA	NA	NA	rs4440944	NA
chr10	1.3E+08	1.3E+08	A	T	UTR3	FOXI2	NA	NA	NA	NA	NA
chr10	1.3E+08	1.3E+08	GG	-	UTR3	FOXI2	NA	NA	NA	NA	NA
chr10	1.3E+08	1.3E+08	A	G	exonic	PTPRE	synonymous SNV	130435:exon14:c.A1380G:p.K460K,PTPRE:NM_006504:exon17:c.A155	=COSM147089;OCCURENCE=1(stomach	rs2275803	NA
chr10	1.3E+08	1.3E+08	C	T	UTR3	PTPRE	NA	NA	NA	NA	NA
chr10	1.34E+08	1.34E+08	-	TA	UTR3	STK32C	NA	NA	NA	rs77786797	NA
chr10	1.34E+08	1.34E+08	C	T	UTR5	LRRC27	NA	NA	NA	rs7084379	NA
chr10	1.34E+08	1.34E+08	C	T	UTR3	LRRC27	NA	NA	NA	rs72863837	NA
chr10	1.34E+08	1.34E+08	A	G	UTR3	LRRC27	NA	NA	NA	rs72863838	NA
chr10	1.35E+08	1.35E+08	T	A	exonic	KNDC1	nonsynonymous SNV	KNDC1:NM_152643:exon14:c.T2417A:p.V806D	NA	rs3008390	B
chr10	1.35E+08	1.35E+08	T	C	exonic	KNDC1	synonymous SNV	KNDC1:NM_152643:exon14:c.T2418C:p.V806V	NA	rs3008389	NA
chr10	1.35E+08	1.35E+08	T	G	UTR3	KNDC1	NA	NA	NA	NA	NA
chr10	1.35E+08	1.35E+08	A	C	UTR3	KNDC1	NA	NA	NA	NA	NA
chr10	1.35E+08	1.35E+08	C	T	ncRNA_exonic	MIR202	NA	NA	NA	rs12355840	NA
chr10	1.35E+08	1.35E+08	T	C	exonic	SYCE1	nonsynonymous SNV	SYCE1:NM_001143764:exon13:c.A962G:p.H321R	=COSM147095;OCCURENCE=1(stomach	rs3737031	NA
chr10	1.35E+08	1.35E+08	T	G	exonic	FRG2B	nonsynonymous SNV	FRG2B:NM_001080998:exon4:c.A805C:p.T269P	NA	rs199913567	NA
chr10	1.35E+08	1.35E+08	G	C	exonic	FRG2B	nonsynonymous SNV	FRG2B:NM_001080998:exon3:c.C326G:p.T109R	NA	rs200530375	NA
chr10	1.35E+08	1.35E+08	C	T	exonic	FRG2B	synonymous SNV	FRG2B:NM_001080998:exon2:c.G183A:p.S61S	NA	rs202189720	NA
chr10	1.35E+08	1.35E+08	A	T	exonic	FRG2B	nonsynonymous SNV	FRG2B:NM_001080998:exon1:c.T44A:p.I15N	NA	rs200661929	NA
chr11	128506	128506	T	C	ncRNA_exonic	LOC100133161	NA	NA	NA	NA	NA
chr11	237087	237087	A	G	exonic	PSMD13	nonsynonymous SNV	NM_002817:exon1:c.A38G:p.N13S,PSMD13:NM_175932:exon1:c.A38	NA	rs1045288	B

chr11	284257	284257	G	A	exonic	NLRP6	synonymous SNV	001276700:exon6:c.G2226A:p.A742A,NLRP6:NM_138329:exon6:c.G22	NA	rs11246050	NA
chr11	406043	406043	G	A	exonic	SIGIRR	synonymous SNV	6T:p.V362V,SIGIRR:NM_001135054:exon10:c.C1086T:p.V362V,SIGIRR:	NA	rs7947	NA
chr11	574754	574754	C	T	ncRNA_exonic	LOC143666	NA	NA	NA	rs12285890	NA
chr11	574800	574800	C	A	ncRNA_exonic	LOC143666	NA	NA	NA	NA	NA
chr11	574813	574813	C	A	ncRNA_exonic	LOC143666	NA	NA	NA	rs12285942	NA
chr11	611919	611919	C	G	UTR3	PHRF1	NA	NA	NA	rs702966	NA
chr11	621227	621227	T	G	exonic	CDHR5	nonsynonymous SNV	642C:p.E214D,CDHR5:NM_021924:exon7:c.A642C:p.E214D,CDHR5:NM	NA	NA	P
chr11	795097	795097	T	G	UTR5	SLC25A22	NA	NA	NA	NA	NA
chr11	838760	838760	T	A	UTR3	CD151	NA	NA	NA	rs1130719	NA
chr11	1016067	1016067	G	C	exonic	MUC6	nonsynonymous SNV	MUC6:NM_005961:exon31:c.C6734G:p.T2245S	NA	NA	NA
chr11	1016074	1016074	A	T	exonic	MUC6	nonsynonymous SNV	MUC6:NM_005961:exon31:c.T6727A:p.S2243T	NA	NA	NA
chr11	1016704	1016704	C	A	exonic	MUC6	nonsynonymous SNV	MUC6:NM_005961:exon31:c.G6097T:p.A2033S	NA	rs75826443	NA
chr11	1016842	1016842	T	G	exonic	MUC6	nonsynonymous SNV	MUC6:NM_005961:exon31:c.A5959C:p.T1987P	NA	rs34490696	NA
chr11	1017154	1017154	T	G	exonic	MUC6	nonsynonymous SNV	MUC6:NM_005961:exon31:c.A5647C:p.I1883L	NA	rs112965257	NA
chr11	1017158	1017158	G	T	exonic	MUC6	synonymous SNV	MUC6:NM_005961:exon31:c.C5643A:p.T1881T	NA	rs36143620	NA
chr11	1017317	1017317	G	T	exonic	MUC6	synonymous SNV	MUC6:NM_005961:exon31:c.C5484A:p.T1828T	NA	rs33943903	NA
chr11	1017337	1017337	T	C	exonic	MUC6	nonsynonymous SNV	MUC6:NM_005961:exon31:c.A5464G:p.T1822A	NA	rs76686156	NA
chr11	1017338	1017338	C	A	exonic	MUC6	nonsynonymous SNV	MUC6:NM_005961:exon31:c.G5463T:p.Q1821H	NA	rs78943453	NA
chr11	1018168	1018170	TCG	-	exonic	MUC6	nonframeshift deletion	MUC6:NM_005961:exon31:c.4631_4633del:p.1544_1545del	NA	rs111749447	NA
chr11	1018304	1018304	G	C	exonic	MUC6	synonymous SNV	MUC6:NM_005961:exon31:c.C4497G:p.A1499A	NA	rs113835477	NA
chr11	1081757	1081757	G	C	exonic	MUC2	nonsynonymous SNV	MUC2:NM_002457:exon13:c.G1685C:p.S562T	NA	rs57737240	NA
chr11	1093487	1093487	T	C	exonic	MUC2	nonsynonymous SNV	MUC2:NM_002457:exon30:c.T5306C:p.L1769P	NA	NA	NA
chr11	1264058	1264058	T	C	exonic	MUC5B	nonsynonymous SNV	MUC5B:NM_002458:exon31:c.T5948C:p.I1983T	NA	NA	NA
chr11	1264069	1264069	T	C	exonic	MUC5B	nonsynonymous SNV	MUC5B:NM_002458:exon31:c.T5959C:p.S1987P	NA	NA	NA
chr11	1264631	1264631	A	G	exonic	MUC5B	nonsynonymous SNV	MUC5B:NM_002458:exon31:c.A6521G:p.N2174S	=COSM1127860;OCCURENCE=1(prostat	rs56726556	NA
chr11	1264717	1264717	C	T	exonic	MUC5B	nonsynonymous SNV	MUC5B:NM_002458:exon31:c.C6607T:p.P2203S	M1146630,COSM687359;OCCURENCE=	rs200947535	NA
chr11	1575443	1575443	T	C	UTR3	DUSP8	NA	NA	NA	NA	NA
chr11	1605904	1605904	T	G	exonic	KRTAP5-1	synonymous SNV	KRTAP5-1:NM_001005922:exon1:c.A576C:p.G192G	NA	rs113408385	NA
chr11	1606147	1606147	A	G	exonic	KRTAP5-1	synonymous SNV	KRTAP5-1:NM_001005922:exon1:c.T333C:p.S111S	NA	rs138363822	NA
chr11	1629373	1629373	T	G	exonic	KRTAP5-3	synonymous SNV	KRTAP5-3:NM_001012708:exon1:c.A243C:p.G81G	NA	NA	NA
chr11	1642305	1642305	T	G	UTR3	KRTAP5-4	NA	NA	NA	NA	NA
chr11	1651199	1651199	-	CTGTGGCT	exonic	KRTAP5-5	nonframeshift insertion	5-5:NM_001001480:exon1:c.129_130insGGCTGTGGCTCC:p.G43delins	NA	NA	NA
chr11	1687684	1687684	G	C	ncRNA_exonic	FAM99A	NA	NA	NA	rs202130518	NA
chr11	2011144	2011144	T	C	ncRNA_exonic	MRPL23-AS1	NA	NA	NA	rs217716	NA
chr11	2292139	2292139	A	C	UTR5	ASCL2	NA	NA	NA	NA	NA
chr11	2435956	2435956	C	T	exonic	TRPM5	nonsynonymous SNV	TRPM5:NM_014555:exon11:c.G1733A:p.R578Q	NA	rs4929982	NA
chr11	2672192	2672195	ATCT	-	ncRNA_exonic	KCNQ1OT1	NA	NA	NA	rs143246270	NA
chr11	2672237	2672237	G	A	ncRNA_exonic	KCNQ1OT1	NA	NA	NA	rs170762	NA
chr11	2672432	2672432	-	A	ncRNA_exonic	KCNQ1OT1	NA	NA	NA	rs34514246	NA
chr11	2699252	2699252	-	T	ncRNA_exonic	KCNQ1OT1	NA	NA	NA	NA	NA
chr11	2701076	2701076	C	A	ncRNA_exonic	KCNQ1OT1	NA	NA	NA	NA	NA
chr11	2720899	2720899	G	C	ncRNA_exonic	KCNQ1OT1	NA	NA	ID=COSN217036;OCCURENCE=2(liver)	rs145762501	NA
chr11	2924850	2924850	-	C	UTR5	SLC22A18AS	NA	NA	NA	NA	NA
chr11	3663273	3663273	C	T	UTR5	ART5	NA	NA	NA	rs7112533	NA
chr11	3877114	3877118	TCTCT	-	UTR5	STIM1	NA	NA	NA	rs147711952	NA
chr11	4141132	4141132	C	A	exonic	RRM1	synonymous SNV	RRM1:NM_001033:exon9:c.C850A:p.R284R	NA	rs183484	NA
chr11	5269586	5269586	A	G	UTR3	HBG1	NA	NA	NA	rs62755960	NA
chr11	6239344	6239344	G	A	exonic	FAM160A2	nonsynonymous SNV	001098794:exon9:c.C1472T:p.T491M,FAM160A2:NM_032127:exon9:c	NA	rs3750944	B
chr11	6411935	6411935	T	C	exonic	SMPD1	nonsynonymous SNV	/I_000543:exon1:c.T107C:p.V36A,SMPD1:NM_001007593:exon1:c.T10	NA	rs1050228	NA
chr11	6411936	6411941	GCTGGC	-	exonic	SMPD1	nonframeshift deletion	:exon1:c.108_113del:p.36_38del,SMPD1:NM_001007593:exon1:c.108	NA	rs3838786	NA
chr11	6549995	6549995	A	G	exonic	DNHD1	nonsynonymous SNV	DNHD1:NM_144666:exon11:c.A2081G:p.N694S	NA	rs7480644	NA
chr11	8009107	8009107	G	T	exonic	EIF3F	nonsynonymous SNV	EIF3F:NM_003754:exon1:c.G208T:p.A70S	NA	NA	NA
chr11	8708939	8708939	A	C	UTR3	RPL27A	NA	NA	NA	rs1057198	NA
chr11	9003014	9003014	A	-	UTR3	NRIP3	NA	NA	NA	rs10714611	NA
chr11	9051475	9051475	G	C	exonic	SCUBE2	nonsynonymous SNV	01170690:exon15:c.C1994G:p.T665S,SCUBE2:NM_020974:exon18:c.C	NA	rs3751057	D
chr11	9117694	9117694	T	-	ncRNA_exonic	KRT8P41	NA	NA	NA	NA	NA
chr11	10772951	10772951	G	T	UTR5	CTR9	NA	NA	NA	NA	NA
chr11	11985134	11985139	TTTACT	-	UTR3	DKK3	NA	NA	NA	rs142843448	NA
chr11	12316389	12316389	A	C	exonic	MICALCL	nonsynonymous SNV	MICALCL:NM_032867:exon3:c.A1411C:p.T471P	NA	rs3812754	B
chr11	13031253	13031253	C	A	exonic	RASSF10	synonymous SNV	RASSF10:NM_001080521:exon1:c.C130A:p.R44R	NA	rs61879068	NA
chr11	15268574	15268574	T	-	UTR3	INSC	NA	NA	NA	NA	NA
chr11	15990619	15990619	A	-	UTR3	SOX6	NA	NA	NA	NA	NA
chr11	16778455	16778455	-	A	UTR3	C11orf58	NA	NA	NA	NA	NA
chr11	17519742	17519742	C	G	exonic	USH1C	nonsynonymous SNV	005709:exon19:c.G1557C:p.E519D,USH1C:NM_153676:exon24:c.G24	NA	rs1064074	P
chr11	17743207	17743207	G	C	UTR3	MYOD1	NA	NA	NA	NA	NA
chr11	18234755	18234755	G	A	ncRNA_exonic	LOC494141	NA	NA	NA	rs11024553	NA

chr11	18388442	18388442	A	-	UTR3	GTF2H1	NA	NA	NA	NA	NA
chr11	20623023	20623023	C	T	exonic	SLC6A5	synonymous SNV	SLC6A5:NM_004211:exon2:c.C352T:p.L118L	NA	rs2241941	NA
chr11	22401038	22401038	T	-	UTR3	SLC17A6	NA	NA	NA	rs35743767	NA
chr11	26581382	26581382	-	AC	UTR3	MUC15	NA	NA	NA	NA	NA
chr11	26690702	26690702	C	T	UTR3	SLC5A12	NA	NA	NA	rs76831336	NA
chr11	26690715	26690718	TTTC	-	UTR3	SLC5A12	NA	NA	NA	rs138812062	NA
chr11	26692594	26692594	-	TGTGTGTG	UTR3	SLC5A12	NA	NA	NA	NA	NA
chr11	26705307	26705307	C	A	exonic	SLC5A12	nonsynonymous SNV	SLC5A12:NM_178498:exon11:c.G1305T:p.W435C	NA	NA	D
chr11	27360070	27360070	T	C	UTR3	CCDC34	NA	NA	NA	rs79626653	NA
chr11	28354102	28354102	T	A	UTR3	METTL15	NA	NA	NA	rs142327989	NA
chr11	31453369	31453369	T	A	UTR3	DNAJC24	NA	NA	NA	rs12362759	NA
chr11	32875597	32875597	T	C	UTR3	PRRG4	NA	NA	NA	rs7933966	NA
chr11	33094949	33094949	-	CTTTGACTT	UTR3	TCP11L1	NA	NA	NA	NA	NA
chr11	33730147	33730147	A	-	UTR3	CD59	NA	NA	NA	NA	NA
chr11	34502345	34502345	T	C	exonic	ELF5	synonymous SNV	M_001243081:exon5:c.A441G:p.T147T,ELF5:NM_001422:exon6:c.A64	NA	rs2231828	NA
chr11	34642668	34642668	A	G	UTR5	EHF	NA	NA	NA	rs286925	NA
chr11	34642728	34642728	A	T	UTR5	EHF	NA	NA	NA	rs286924	NA
chr11	35201842	35201842	C	T	exonic	CD44	synonymous SNV	T:p.H85H,CD44:NM_001001391:exon3:c.C255T:p.H85H,CD44:NM_00	NA	rs1071695	NA
chr11	35280491	35280492	AA	-	UTR3	SLC1A2	NA	NA	NA	NA	NA
chr11	43940644	43940644	G	T	exonic	ALKBH3	synonymous SNV	ALKBH3:NM_139178:exon9:c.G726T:p.G242G	NA	rs1048928	NA
chr11	46342943	46342943	A	-	UTR3	CREB3L1	NA	NA	NA	NA	NA
chr11	46699495	46699495	A	-	UTR3	ARHGAP1	NA	NA	NA	rs34845803	NA
chr11	48192308	48192308	C	T	UTR3	PTPRJ	NA	NA	NA	rs199522084	NA
chr11	48192308	48192308	-	T	UTR3	PTPRJ	NA	NA	NA	rs11389144	NA
chr11	48373748	48373748	C	G	exonic	OR4C45	unknown	UNKNOWN	ID=COSN229011;OCCURENCE=1(skin)	rs73453188	NA
chr11	48373827	48373827	G	A	exonic	OR4C45	unknown	UNKNOWN	NA	rs80081241	NA
chr11	48373833	48373833	G	A	exonic	OR4C45	unknown	UNKNOWN	NA	rs75965588	NA
chr11	48373834	48373834	A	G	exonic	OR4C45	unknown	UNKNOWN	NA	rs75261463	NA
chr11	57313469	57313469	C	G	exonic	SMTNL1	nonsynonymous SNV	SMTNL1:NM_001105565:exon4:c.C1033G:p.R345G	NA	rs12223229	NA
chr11	57479502	57479502	C	G	exonic	MED19	synonymous SNV	MED19:NM_153450:exon1:c.G150C:p.T50T	NA	NA	NA
chr11	59341901	59341901	T	-	UTR3	OSBP	NA	NA	NA	NA	NA
chr11	59383241	59383241	C	A	UTR5	OSBP	NA	NA	NA	NA	NA
chr11	59863498	59863498	T	-	UTR3	MS4A2	NA	NA	NA	rs5792168	NA
chr11	60971694	60971694	A	G	exonic	PGA3	nonsynonymous SNV	PGA3:NM_001079807:exon2:c.A172G:p.K58E	NA	rs117494851	B
chr11	61405617	61405617	G	A	ncRNA_exonic	RPLP0P2	NA	NA	NA	NA	NA
chr11	61560399	61560399	A	C	UTR5	FEN1	NA	NA	NA	NA	NA
chr11	61569354	61569354	-	T	UTR3	FADS1	NA	NA	NA	rs34397549	NA
chr11	62294717	62294717	A	T	exonic	AHNAK	nonsynonymous SNV	AHNAK:NM_001620:exon5:c.T7172A:p.L2391H	NA	NA	P
chr11	62360961	62360961	T	C	UTR3	MTA2	NA	NA	NA	NA	NA
chr11	62378801	62378801	T	C	exonic	EML3	synonymous SNV	EML3:NM_153265:exon3:c.A210G:p.P70P	NA	rs11553576	NA
chr11	62434173	62434173	G	A	exonic	METTL12	nonsynonymous SNV	METTL12:NM_001043229:exon3:c.G373A:p.G125S	NA	rs11231181	B
chr11	62453952	62453952	G	A	UTR3	LRRN4CL	NA	NA	NA	rs2512561	NA
chr11	62766507	62766507	T	A	exonic	SLC22A8	nonsynonymous SNV	_001184732:exon5:c.A647T:p.Y216F,SLC22A8:NM_001184733:exon5:	NA	NA	B
chr11	62766508	62766508	A	T	exonic	SLC22A8	nonsynonymous SNV	_001184732:exon5:c.T646A:p.Y216N,SLC22A8:NM_001184733:exon5:	NA	NA	D
chr11	62766510	62766510	C	G	exonic	SLC22A8	nonsynonymous SNV	001184732:exon5:c.G644C:p.G215A,SLC22A8:NM_001184733:exon5:	NA	NA	D
chr11	62766511	62766511	C	G	exonic	SLC22A8	nonsynonymous SNV	001184732:exon5:c.G643C:p.G215R,SLC22A8:NM_001184733:exon5:	NA	NA	D
chr11	62766512	62766512	G	A	exonic	SLC22A8	synonymous SNV	_001184732:exon5:c.C642T:p.L214L,SLC22A8:NM_001184733:exon5:	NA	NA	NA
chr11	64107735	64107735	G	A	UTR5	CCDC88B	NA	NA	NA	rs663743	NA
chr11	64137079	64137079	C	A	exonic	RPS6KA4	nonsynonymous SNV	01006944:exon13:c.C1572A:p.D524E,RPS6KA4:NM_003942:exon13:c.	NA	NA	D
chr11	64138805	64138805	T	C	exonic	RPS6KA4	synonymous SNV	01006944:exon17:c.T2154C:p.N718N,RPS6KA4:NM_003942:exon17:c.	NA	rs11542299	NA
chr11	64577370	64577370	G	A	exonic	MEN1	nonsynonymous SNV	C212T:p.P71L,MEN1:NM_130801:exon2:c.C212T:p.P71L,MEN1:NM_1	NA	NA	D
chr11	64794918	64794918	-	G	ncRNA_exonic	ARL2-SNX15	NA	NA	NA	rs3832791	NA
chr11	64807447	64807447	A	C	ncRNA_exonic	ARL2-SNX15	NA	NA	NA	rs664118	NA
chr11	64988520	64988520	T	G	intronic	SLC22A20	NA	NA	NA	NA	NA
chr11	64990041	64990041	G	C	exonic	SLC22A20	nonsynonymous SNV	SLC22A20:NM_001004326:exon4:c.G737C:p.G246A	NA	rs514076	NA
chr11	65403717	65403717	T	G	exonic	PCNXL3	synonymous SNV	PCNXL3:NM_032223:exon33:c.T5532G:p.G1844G	NA	NA	NA
chr11	65764798	65764798	A	-	UTR3	EIF1AD	NA	NA	NA	NA	NA
chr11	66035287	66035287	A	-	UTR3	KLC2	NA	NA	NA	rs5792385	NA
chr11	66393035	66393035	A	G	exonic	RBM14	nonsynonymous SNV	RBM14:NM_006328:exon2:c.A1688G:p.Q563R	NA	NA	NA
chr11	66452843	66452843	T	G	UTR3	SPTBN2	NA	NA	NA	NA	NA
chr11	66452849	66452849	T	G	UTR3	SPTBN2	NA	NA	NA	NA	NA
chr11	67415054	67415054	C	T	UTR5	ACY3	NA	NA	NA	rs2514036	NA
chr11	67572698	67572698	G	T	ncRNA_exonic	FAM86C2P	NA	NA	NA	rs7129283	NA
chr11	67924559	67924559	A	-	UTR3	SUV420H1	NA	NA	NA	NA	NA
chr11	67924560	67924560	T	G	UTR3	SUV420H1	NA	NA	NA	NA	NA
chr11	68381367	68381367	T	-	UTR3	PPP6R3	NA	NA	NA	NA	NA

chr11	68816370	68816370	A	G	UTR5	TPCN2	NA	NA	NA	rs4930642	NA
chr11	68856711	68856716	AATAAC	-	UTR3	TPCN2	NA	NA	NA	rs150950408	NA
chr11	70281360	70281363	TTTT	-	UTR3	CTTN	NA	NA	NA	NA	NA
chr11	70315627	70315627	-	T	UTR3	SHANK2	NA	NA	NA	NA	NA
chr11	71146249	71146260	CAAGGAAC	-	UTR3	DHCR7	NA	NA	NA	rs141483210	NA
chr11	71708250	71708253	GTGT	-	UTR3	RNF121	NA	NA	NA	NA	NA
chr11	72290629	72290629	G	A	exonic	PDE2A	synonymous SNV	_001243784:exon26:c.C2142T:p.I714I,PDE2A:NM_002599:exon26:c.C2	NA	rs392565	NA
chr11	72385393	72385393	A	C	UTR5	PDE2A	NA	NA	NA	NA	NA
chr11	72385400	72385400	T	C	UTR5	PDE2A	NA	NA	NA	NA	NA
chr11	73670645	73670645	T	C	exonic	DNAJB13	synonymous SNV	DNAJB13:NM_153614:exon3:c.T279C:p.H93H	NA	rs653263	NA
chr11	74042308	74042308	C	T	UTR3	PGM2L1	NA	NA	NA	NA	NA
chr11	74042308	74042308	-	TTT	UTR3	PGM2L1	NA	NA	NA	NA	NA
chr11	74045953	74045953	T	-	UTR3	PGM2L1	NA	NA	NA	rs34926765	NA
chr11	74551831	74551831	A	C	UTR3	RNF169	NA	NA	NA	rs200744330	NA
chr11	76372180	76372180	T	G	exonic	LRRC32	nonsynonymous SNV	_001128922:exon3:c.A457C:p.T153P,LRRC32:NM_005512:exon3:c.A4	NA	NA	B
chr11	76372193	76372193	T	G	exonic	LRRC32	synonymous SNV	_001128922:exon3:c.A444C:p.A148A,LRRC32:NM_005512:exon3:c.A4	NA	NA	NA
chr11	76751585	76751585	T	-	splicing	:exon3:c.988+1T>-,NM_1387	NA	NA	NA	rs11292199	NA
chr11	76751604	76751604	T	-	splicing	exon4:c.1006+1T>-,NM_1387	NA	NA	NA	rs11292200	NA
chr11	76836661	76836662	TA	-	UTR3	CAPN5	NA	NA	NA	rs10543158	NA
chr11	76954788	76954788	-	A	exonic	GDPD4	frameshift insertion	GDPD4:NM_182833:exon12:c.1192_1193insT:p.I398fs	NA	NA	NA
chr11	89059054	89059055	TA	-	UTR3	NOX4	NA	NA	NA	NA	NA
chr11	89392653	89392653	A	G	UTR5	FOLH1B	NA	NA	NA	rs10765230	NA
chr11	89531619	89531619	A	G	exonic	TRIM49	synonymous SNV	TRIM49:NM_020358:exon8:c.T1038C:p.H346H	NA	rs11824402	NA
chr11	94150790	94150790	T	C	UTR3	MRE11A	NA	NA	NA	rs2155209	NA
chr11	94803971	94803971	C	T	UTR3	SRSF8	NA	NA	NA	rs61895522	NA
chr11	94863499	94863499	G	T	UTR3	ENDOD1	NA	NA	NA	rs500849	NA
chr11	96126289	96126289	T	-	UTR3	JRKL	NA	NA	NA	rs201157332	NA
chr11	99690286	99690286	T	G	exonic	CNTN5	nonsynonymous SNV	.T67G:p.S23A,CNTN5:NM_001243271:exon4:c.T67G:p.S23A,CNTN5:NI	NA	rs10790978	NA
chr11	1.02E+08	1.02E+08	G	A	UTR3	KIAA1377	NA	NA	NA	NA	NA
chr11	1.03E+08	1.03E+08	C	A	UTR5	MMP27	NA	NA	NA	rs11225389	NA
chr11	1.03E+08	1.03E+08	A	G	UTR3	DCUN1D5	NA	NA	NA	NA	NA
chr11	1.03E+08	1.03E+08	T	G	UTR3	DCUN1D5	NA	NA	NA	NA	NA
chr11	1.03E+08	1.03E+08	C	A	UTR3	DCUN1D5	NA	NA	NA	NA	NA
chr11	1.05E+08	1.05E+08	T	-	exonic	CASP5	frameshift deletion	_001136112:exon2:c.67delA:p.R23fs,CASP5:NM_004347:exon2:c.28de	NA	NA	NA
chr11	1.05E+08	1.05E+08	TTT	-	UTR5	GRIA4	NA	NA	NA	NA	NA
chr11	1.06E+08	1.06E+08	-	A	UTR3	GRIA4	NA	NA	NA	NA	NA
chr11	1.06E+08	1.06E+08	-	CACACA	UTR3	GRIA4	NA	NA	NA	NA	NA
chr11	1.08E+08	1.08E+08	A	G	UTR3	SLC35F2	NA	NA	NA	rs4754259	NA
chr11	1.08E+08	1.08E+08	TT	-	UTR3	CUL5	NA	NA	NA	NA	NA
chr11	1.08E+08	1.08E+08	G	A	UTR5	ATM	NA	NA	NA	rs189037	NA
chr11	1.08E+08	1.08E+08	T	C	exonic	EXPH5	nonsynonymous SNV	EXPH5:NM_015065:exon1:c.A55G:p.R19G	)=COSM147323;OCCURENCE=1(stomacl	rs2640738	D
chr11	1.1E+08	1.1E+08	A	C	UTR3	FDX1	NA	NA	NA	rs4753894	NA
chr11	1.11E+08	1.11E+08	-	AGGA	UTR3	POU2AF1	NA	NA	NA	NA	NA
chr11	1.11E+08	1.11E+08	-	AAGAAG	UTR3	POU2AF1	NA	NA	NA	NA	NA
chr11	1.12E+08	1.12E+08	G	-	splicing	on3:c.61-1C>-,NM_00107769(	NA	NA	NA	rs10708475	NA
chr11	1.13E+08	1.13E+08	-	G	UTR5	NCAM1	NA	NA	NA	rs145493999	NA
chr11	1.14E+08	1.14E+08	G	A	UTR3	USP28	NA	NA	NA	NA	NA
chr11	1.17E+08	1.17E+08	C	G	exonic	BACE1	synonymous SNV	1:NM_012104:exon5:c.G786C:p.V262V,BACE1:NM_138971:exon5:c.G6	NA	rs638405	NA
chr11	1.17E+08	1.17E+08	A	C	exonic	CEP164	nonsynonymous SNV	01271933:exon13:c.A1711C:p.T571P,CEP164:NM_014956:exon14:c.A1	NA	rs74388237	B
chr11	1.17E+08	1.17E+08	G	T	exonic	DSCAML1	nonsynonymous SNV	DSCAML1:NM_020693:exon4:c.C694A:p.H232N	)=COSM147330;OCCURENCE=1(stomacl	rs3741280	NA
chr11	1.18E+08	1.18E+08	CACA	-	UTR3	FXYD6	NA	NA	NA	NA	NA
chr11	1.18E+08	1.18E+08	G	A	UTR3	IL10RA	NA	NA	NA	rs9610	NA
chr11	1.19E+08	1.19E+08	G	A	UTR3	DDX6	NA	NA	NA	rs78116693	NA
chr11	1.19E+08	1.19E+08	G	C	UTR3	DDX6	NA	NA	NA	rs487728	NA
chr11	1.19E+08	1.19E+08	-	T	UTR3	DDX6	NA	NA	NA	rs139078958	NA
chr11	1.19E+08	1.19E+08	C	T	exonic	BCL9L	synonymous SNV	BCL9L:NM_182557:exon6:c.G1014A:p.S338S	NA	rs35346926	NA
chr11	1.19E+08	1.19E+08	T	G	UTR3	HYOU1	NA	NA	NA	NA	NA
chr11	1.23E+08	1.23E+08	C	A	exonic	UBASH3B	nonsynonymous SNV	UBASH3B:NM_032873:exon4:c.C411A:p.D137E	NA	NA	D
chr11	1.23E+08	1.23E+08	C	T	exonic	CLMP	nonsynonymous SNV	CLMP:NM_024769:exon3:c.G206A:p.R69H	NA	rs2276348	B
chr11	1.24E+08	1.24E+08	C	T	exonic	VWA5A	synonymous SNV	14622:exon17:c.C2205T:p.H735H,VWA5A:NM_001130142:exon18:c.C	NA	rs1056615	NA
chr11	1.25E+08	1.25E+08	-	T	UTR3	MSANTD2	NA	NA	NA	rs137996267	NA
chr11	1.25E+08	1.25E+08	T	G	exonic	ROBO3	synonymous SNV	ROBO3:NM_022370:exon15:c.T2343G:p.G781G	NA	NA	NA
chr11	1.25E+08	1.25E+08	G	A	exonic	ROBO4	synonymous SNV	ROBO4:NM_019055:exon2:c.C103T:p.L35L	NA	rs73016370	NA
chr11	1.25E+08	1.25E+08	G	A	UTR3	SLC37A2	NA	NA	NA	NA	NA
chr11	1.25E+08	1.25E+08	-	C	exonic	EI24	frameshift insertion	EI24:NM_001007277:exon9:c.732_733insC:p.R244fs	NA	rs150851358	NA
chr11	1.25E+08	1.25E+08	AAAA	-	UTR3	EI24	NA	NA	NA	NA	NA

chr11	1.25E+08	1.25E+08	C	T	exonic	STT3A	synonymous SNV	STT3A:NM_152713:exon16:c.C1911T:p.C637C	NA	rs17140116	NA
chr11	1.25E+08	1.25E+08	C	T	UTR5	CHEK1	NA	NA	NA	rs61451117	NA
chr11	1.25E+08	1.25E+08	C	T	UTR5	CHEK1	NA	NA	NA	rs558351	NA
chr11	1.26E+08	1.26E+08	C	T	UTR5	HYLS1	NA	NA	NA	rs583355	NA
chr11	1.26E+08	1.26E+08	T	G	UTR3	CDON	NA	NA	NA	rs61917812	NA
chr11	1.26E+08	1.26E+08	C	T	exonic	DCPS	synonymous SNV	DCPS:NM_014026:exon1:c.C63T:p.H21H	NA	rs3740915	NA
chr11	1.29E+08	1.29E+08	C	G	UTR5	KCNJ5	NA	NA	NA	NA	NA
chr11	1.29E+08	1.29E+08	C	G	UTR5	KCNJ5	NA	NA	NA	rs10893931	NA
chr11	1.3E+08	1.3E+08	C	T	exonic	PRDM10	nonsynonymous SNV	NM_020228:exon2:c.G64A:p.A22T,PRDM10:NM_199437:exon2:c.G64	=COSM147383;OCCURENCE=1(stomac	rs11221912	B
chr11	1.3E+08	1.3E+08	T	G	ncRNA_exonic	LINC00167	NA	NA	NA	NA	NA
chr11	1.3E+08	1.3E+08	-	T	UTR3	ZBTB44	NA	NA	NA	NA	NA
chr11	1.32E+08	1.32E+08	T	-	UTR3	NTM	NA	NA	NA	rs61142048	NA
chr11	1.34E+08	1.34E+08	C	G	exonic	IGSF9B	synonymous SNV	IGSF9B:NM_001277285:exon2:c.G237C:p.P79P	NA	rs10894768	NA
chr11	1.34E+08	1.34E+08	G	A	UTR3	JAM3	NA	NA	NA	rs597320	NA
chr12	147969	147969	T	-	ncRNA_exonic	FAM138D	NA	NA	NA	rs112827715	NA
chr12	427575	427575	A	G	exonic	KDM5A	nonsynonymous SNV	KDM5A:NM_001042603:exon19:c.T2594C:p.M865T	NA	rs11062385	B
chr12	498268	498270	GGG	-	UTR5	KDM5A	NA	NA	NA	NA	NA
chr12	1022452	1022452	C	T	UTR3	RAD52	NA	NA	NA	rs1051669	NA
chr12	1613273	1613273	T	-	ncRNA_exonic	LOC100292680	NA	NA	NA	rs34677864	NA
chr12	1613488	1613488	G	-	ncRNA_exonic	LOC100292680	NA	NA	NA	rs58197174	NA
chr12	3049488	3049488	A	-	UTR3	TULP3	NA	NA	NA	NA	NA
chr12	3393127	3393127	T	G	UTR3	TSPAN9	NA	NA	NA	NA	NA
chr12	3393144	3393144	T	G	UTR3	TSPAN9	NA	NA	NA	NA	NA
chr12	3394436	3394436	T	G	UTR3	TSPAN9	NA	NA	NA	NA	NA
chr12	4410639	4410639	T	-	UTR3	CCND2	NA	NA	NA	NA	NA
chr12	4414167	4414167	T	-	UTR3	CCND2	NA	NA	NA	rs61152953	NA
chr12	4463020	4463020	C	G	UTR3	C12orf5	NA	NA	NA	rs7297048	NA
chr12	4463032	4463032	G	T	UTR3	C12orf5	NA	NA	NA	rs7296130	NA
chr12	4627414	4627414	C	T	exonic	C12orf4	synonymous SNV	C12orf4:NM_020374:exon8:c.G843A:p.Q281Q	NA	rs35924298	NA
chr12	4919197	4919197	T	C	UTR5	KCNA6	NA	NA	NA	NA	NA
chr12	4919248	4919248	A	G	exonic	KCNA6	nonsynonymous SNV	KCNA6:NM_002235:exon1:c.A41G:p.E14G	NA	NA	P
chr12	4919251	4919251	T	G	exonic	KCNA6	nonsynonymous SNV	KCNA6:NM_002235:exon1:c.T44G:p.V15G	NA	NA	P
chr12	4919269	4919269	A	G	exonic	KCNA6	nonsynonymous SNV	KCNA6:NM_002235:exon1:c.A62G:p.E21G	NA	NA	B
chr12	6560338	6560338	A	C	ncRNA_exonic	CD27-AS1	NA	NA	NA	NA	NA
chr12	6560343	6560343	T	C	ncRNA_exonic	CD27-AS1	NA	NA	NA	NA	NA
chr12	6715593	6715593	A	G	UTR5	CHD4	NA	NA	NA	rs1734116	NA
chr12	6862174	6862174	C	G	upstream	MLF2	NA	NA	NA	rs13402	NA
chr12	6980076	6980076	A	-	UTR3	TPI1	NA	NA	NA	NA	NA
chr12	7023123	7023123	A	G	exonic	LRRC23	nonsynonymous SNV	LRRC23:NM_006992:exon7:c.A827G:p.E276G	NA	rs710415	NA
chr12	7247166	7247166	-	A	UTR3	C1RL	NA	NA	NA	NA	NA
chr12	7311419	7311420	TG	-	UTR3	CLSTN3	NA	NA	NA	rs55888711	NA
chr12	7649484	7649484	T	C	exonic	CD163	nonsynonymous SNV	M_004244:exon5:c.A1024G:p.I342V,CD163:NM_203416:exon5:c.A1024	NA	rs4883263	NA
chr12	7945559	7945559	T	C	exonic	NANOG	synonymous SNV	NANOG:NM_024865:exon2:c.T165C:p.P55P	NA	rs4294629	NA
chr12	7945640	7945640	G	T	exonic	NANOG	nonsynonymous SNV	NANOG:NM_024865:exon2:c.G246T:p.K82N	NA	rs2889551	B
chr12	7945670	7945670	G	A	exonic	NANOG	synonymous SNV	NANOG:NM_024865:exon2:c.G276A:p.P92P	NA	rs4354764	NA
chr12	7945757	7945757	C	T	exonic	NANOG	synonymous SNV	NANOG:NM_024865:exon2:c.C363T:p.S121S	NA	rs4438116	NA
chr12	7948108	7948108	G	A	UTR3	NANOG	NA	NA	NA	rs61937162	NA
chr12	7948160	7948160	A	G	UTR3	NANOG	NA	NA	NA	rs28538179	NA
chr12	7948476	7948476	C	T	UTR3	NANOG	NA	NA	NA	rs35472045	NA
chr12	7948517	7948517	C	T	UTR3	NANOG	NA	NA	NA	rs34237790	NA
chr12	7948549	7948549	G	A	UTR3	NANOG	NA	NA	NA	rs34367740	NA
chr12	7948584	7948584	C	A	UTR3	NANOG	NA	NA	NA	rs35212015	NA
chr12	8186173	8186173	G	T	UTR5	FOXJ2	NA	NA	NA	NA	NA
chr12	8353200	8353200	G	T	ncRNA_exonic	FAM66C	NA	NA	NA	rs71189369	NA
chr12	8353252	8353252	G	A	ncRNA_exonic	FAM66C	NA	NA	NA	rs200087976	NA
chr12	8353486	8353486	C	G	ncRNA_exonic	FAM66C	NA	NA	NA	rs200786680	NA
chr12	8799388	8799388	G	T	UTR3	MFAP5	NA	NA	NA	NA	NA
chr12	8927169	8927169	T	-	UTR3	RIMKLB	NA	NA	NA	rs56912350	NA
chr12	9098995	9098995	-	AAC	exonic	M6PR	nonframeshift insertion	'024:exon2:c.6_7insGTT:p.F2delinsLF,M6PR:NM_002355:exon2:c.6_7ir	NA	rs149871778	NA
chr12	9162783	9162783	A	-	UTR3	KLRG1	NA	NA	NA	NA	NA
chr12	9436262	9436262	T	G	ncRNA_exonic	LOC642846	NA	NA	NA	NA	NA
chr12	9466225	9466225	C	T	ncRNA_exonic	LOC642846	NA	NA	NA	rs2911815	NA
chr12	9585079	9585079	G	A	ncRNA_exonic	DDX12P	NA	NA	NA	NA	NA
chr12	9851808	9851808	G	T	UTR3	CLEC2D	NA	NA	NA	rs10772046	NA
chr12	10022448	10022448	-	CACACACA	UTR5	CLEC2B	NA	NA	NA	rs10644863	NA
chr12	10365574	10365574	C	G	UTR5	GABARAPL1	NA	NA	NA	rs2302486	NA

chr12	10468648	10468648	T	C	UTR3	KLRD1	NA	NA	NA	rs7966660	NA
chr12	10560957	10560957	T	C	exonic	KLRC4	nonsynonymous SNV	KLRC4:NM_013431:exon3:c.A311G:p.N104S	NA	rs2617170	NA
chr12	10746385	10746385	G	A	ncRNA_exonic	KLRAP1	NA	NA	NA	rs2244387	NA
chr12	11183752	11183752	T	C	exonic	TAS2R31	synonymous SNV	TAS2R31:NM_176885:exon1:c.A183G:p.V61V	NA	rs72475489	NA
chr12	11183920	11183920	T	C	exonic	TAS2R31	nonsynonymous SNV	TAS2R31:NM_176885:exon1:c.A15G:p.I5M	NA	rs201034225	NA
chr12	11183922	11183922	T	G	exonic	TAS2R31	nonsynonymous SNV	TAS2R31:NM_176885:exon1:c.A13C:p.I5L	NA	NA	NA
chr12	11183930	11183930	G	A	exonic	TAS2R31	nonsynonymous SNV	TAS2R31:NM_176885:exon1:c.C5T:p.T2I	NA	NA	NA
chr12	11183952	11183952	A	G	ncRNA_UTR5	TAS2R31	NA	NA	NA	NA	NA
chr12	11183958	11183958	G	T	ncRNA_UTR5	TAS2R31	NA	NA	NA	NA	NA
chr12	11183961	11183961	T	A	ncRNA_UTR5	TAS2R31	NA	NA	NA	NA	NA
chr12	11183962	11183962	T	A	ncRNA_UTR5	TAS2R31	NA	NA	NA	NA	NA
chr12	11183977	11183977	G	A	ncRNA_UTR5	TAS2R31	NA	NA	NA	rs73254862	NA
chr12	11420471	11420471	C	G	exonic	PRB3	nonsynonymous SNV	PRB3:NM_006249:exon3:c.G712C:p.G238R	NA	NA	NA
chr12	11420647	11420647	T	C	exonic	PRB3	nonsynonymous SNV	PRB3:NM_006249:exon3:c.A536G:p.H179R	NA	rs78040729	NA
chr12	11420715	11420715	C	G	exonic	PRB3	synonymous SNV	PRB3:NM_006249:exon3:c.G468C:p.P156P	NA	NA	NA
chr12	11461533	11461533	G	T	exonic	PRB4	synonymous SNV	PRB4:NM_002723:exon3:c.C384A:p.G128G	NA	NA	NA
chr12	11461742	11461742	C	T	exonic	PRB4	nonsynonymous SNV	_001261399:exon3:c.G175A:p.G59R,PRB4:NM_002723:exon3:c.G175	NA	NA	NA
chr12	11461802	11461802	G	A	exonic	PRB4	stopgain SNV	_001261399:exon3:c.C115T:p.R39X,PRB4:NM_002723:exon3:c.C115	NA	rs12829245	NA
chr12	12045145	12045147	TTT	-	ncRNA_UTR3	ETV6	NA	NA	NA	NA	NA
chr12	12046785	12046785	C	T	ncRNA_UTR3	ETV6	NA	NA	NA	rs59866364	NA
chr12	12046813	12046813	A	G	ncRNA_UTR3	ETV6	NA	NA	NA	rs2710275	NA
chr12	12943043	12943045	TTC	-	UTR3	APOLD1	NA	NA	NA	rs146851735	NA
chr12	13029070	13029071	AA	-	ncRNA_exonic	RPL13AP20	NA	NA	NA	NA	NA
chr12	13153388	13153388	G	T	ncRNA_exonic	HTR7P1	NA	NA	NA	rs77117939	NA
chr12	18435399	18435401	CCC	-	exonic	PIK3C2G	nonframeshift deletion	PIK3C2G:NM_004570:exon2:c.384_386del:p.128_129del	NA	rs55845540	NA
chr12	18499636	18499636	C	T	exonic	PIK3C2G	synonymous SNV	PIK3C2G:NM_004570:exon11:c.C1491T:p.Y497Y	=COSM147454;OCCURENCE=1(stomac	rs17847788	NA
chr12	18656225	18656225	G	A	exonic	PIK3C2G	synonymous SNV	PIK3C2G:NM_004570:exon22:c.G2904A:p.Q968Q	NA	rs11044142	NA
chr12	20968645	20968662	CACTTGGT	-	UTR5	SLCO1B3	NA	NA	NA	NA	NA
chr12	20968666	20968669	TTTA	-	UTR5	SLCO1B3	NA	NA	NA	rs4149158	NA
chr12	21329738	21329738	A	G	exonic	SLCO1B1	nonsynonymous SNV	SLCO1B1:NM_006446:exon5:c.A388G:p.N130D	NA	rs2306283	B
chr12	21487769	21487769	-	T	UTR5	SLCO1A2	NA	NA	NA	rs3834939	NA
chr12	22351457	22351458	AC	-	UTR3	ST8SIA1	NA	NA	NA	rs71718015	NA
chr12	22839786	22839794	TTTTTTTTT	-	UTR3	ETNK1	NA	NA	NA	NA	NA
chr12	24967736	24967736	G	-	UTR3	BCAT1	NA	NA	NA	rs139849074	NA
chr12	26386265	26386266	AT	-	UTR3	SSPN	NA	NA	NA	rs10547333	NA
chr12	27846972	27846972	G	A	UTR3	PPFIBP1	NA	NA	NA	rs151006610	NA
chr12	27953288	27953288	T	G	UTR3	KLHL42	NA	NA	NA	rs150881191	NA
chr12	29493586	29493586	T	-	UTR3	ERGIC2	NA	NA	NA	NA	NA
chr12	31821428	31821428	-	T	UTR3	METTL20	NA	NA	NA	NA	NA
chr12	31821464	31821464	C	A	UTR3	METTL20	NA	NA	NA	rs137891657	NA
chr12	32797823	32797823	A	-	UTR3	FGD4	NA	NA	NA	NA	NA
chr12	34175263	34175263	G	C	UTR5	ALG10	NA	NA	NA	NA	NA
chr12	39687536	39687536	A	-	UTR3	KIF21A	NA	NA	NA	rs201147142	NA
chr12	45307633	45307633	T	C	UTR5	NELL2	NA	NA	NA	rs374144	NA
chr12	48176656	48176657	CA	-	UTR3	HDAC7	NA	NA	NA	rs57423868	NA
chr12	48398080	48398080	T	A	exonic	COL2A1	nonsynonymous SNV	1:NM_001844:exon1:c.A25T:p.T9S,COL2A1:NM_033150:exon1:c.A25T	NA	rs3803183	NA
chr12	48542411	48542411	C	T	UTR3	ASB8	NA	NA	NA	NA	NA
chr12	48542415	48542415	A	G	UTR3	ASB8	NA	NA	NA	rs12310813	NA
chr12	49251014	49251014	T	C	UTR3	RND1	NA	NA	NA	NA	NA
chr12	49251047	49251047	C	A	UTR3	RND1	NA	NA	NA	NA	NA
chr12	49298059	49298059	G	A	UTR5	CCDC65	NA	NA	NA	rs3809147	NA
chr12	49445517	49445517	A	T	exonic	MLL2	nonsynonymous SNV	MLL2:NM_003482:exon10:c.T1949A:p.V650E	NA	NA	NA
chr12	49483423	49483423	C	T	UTR3	DHH	NA	NA	NA	rs145466782	NA
chr12	49726255	49726255	-	G	UTR3	C1QL4	NA	NA	NA	rs112341833	NA
chr12	50262401	50262401	A	C	UTR3	FAIM2	NA	NA	NA	NA	NA
chr12	50589666	50589666	G	A	exonic	LIMA1	nonsynonymous SNV	_001113547:exon5:c.C497T:p.S166F,LIMA1:NM_001113546:exon8:c.C	NA	NA	P
chr12	50746568	50746568	C	G	exonic	FAM186A	nonsynonymous SNV	FAM186A:NM_001145475:exon4:c.G4047C:p.M1349I	NA	NA	NA
chr12	50747134	50747134	T	G	exonic	FAM186A	nonsynonymous SNV	FAM186A:NM_001145475:exon4:c.A3481C:p.T1161P	NA	NA	NA
chr12	50747160	50747160	T	G	exonic	FAM186A	nonsynonymous SNV	FAM186A:NM_001145475:exon4:c.A3455C:p.D1152A	NA	NA	NA
chr12	51203371	51203371	C	T	exonic	ATF1	synonymous SNV	ATF1:NM_005171:exon4:c.C327T:p.Y109Y	NA	rs1129406	NA
chr12	51324874	51324875	TT	-	UTR3	METTL7A	NA	NA	NA	rs56130607	NA
chr12	51723499	51723499	T	C	exonic	CELA1	nonsynonymous SNV	CELA1:NM_001971:exon7:c.A728G:p.Q243R	NA	rs17860364	B
chr12	51740413	51740413	-	C	exonic	CELA1	frameshift insertion	CELA1:NM_001971:exon1:c.10_11insG:p.L4fs	NA	NA	NA
chr12	52212288	52212288	T	C	UTR3	FIGNL2	NA	NA	NA	rs303821	NA
chr12	52285209	52285209	-	TA	UTR3	ANKRD33	NA	NA	NA	rs35582441	NA
chr12	52409005	52409005	T	G	UTR3	GRASP	NA	NA	NA	NA	NA

chr12	52844243	52844243	A	G	exonic	KRT6B	synonymous SNV	KRT6B:NM_005555:exon2:c.T702C:p.R234R	NA	rs28414881	NA
chr12	52844246	52844246	A	G	exonic	KRT6B	synonymous SNV	KRT6B:NM_005555:exon2:c.T699C:p.G233G	ID=COSM118699;OCCURENCE=1(ovary)	rs28542657	NA
chr12	52908789	52908789	A	C	exonic	KRT5	synonymous SNV	KRT5:NM_000424:exon9:c.T1710G:p.G570G	NA	rs201553490	NA
chr12	52986187	52986187	T	C	exonic	KRT72	nonsynonymous SNV	91G:p.Y264C,KRT72:NM_001146226:exon4:c.A791G:p.Y264C,KRT72:N	NA	rs12833456	B
chr12	52994955	52994955	G	A	exonic	KRT72	synonymous SNV	C282T:p.P94P,KRT72:NM_001146226:exon1:c.C282T:p.P94P,KRT72:NM	NA	rs61747192	NA
chr12	53045626	53045626	T	C	exonic	KRT2	nonsynonymous SNV	KRT2:NM_000423:exon1:c.A301G:p.S101G	NA	rs2634041	NA
chr12	53070145	53070145	G	A	exonic	KRT1	synonymous SNV	KRT1:NM_006121:exon7:c.C1389T:p.R463R	NA	rs936958	NA
chr12	53085032	53085032	T	C	exonic	KRT77	nonsynonymous SNV	KRT77:NM_175078:exon9:c.A1654G:p.S552G	NA	NA	NA
chr12	53085044	53085044	T	C	exonic	KRT77	nonsynonymous SNV	KRT77:NM_175078:exon9:c.A1642G:p.S548G	NA	NA	NA
chr12	53729491	53729491	T	G	UTR5	SP7	NA	NA	NA	NA	NA
chr12	53810124	53810124	G	T	UTR3	SP1	NA	NA	NA	NA	NA
chr12	53810134	53810136	TTT	-	UTR3	SP1	NA	NA	NA	rs147945503	NA
chr12	53877766	53877766	C	T	exonic	MAP3K12	synonymous SNV	001193511:exon8:c.G1287A:p.K429K,MAP3K12:NM_006301:exon9:c.G	NA	NA	NA
chr12	54427103	54427103	A	C	exonic	HOXC5	nonsynonymous SNV	HOXC5:NM_018953:exon1:c.A197C:p.N66T	NA	NA	B
chr12	54427115	54427115	A	C	exonic	HOXC5	nonsynonymous SNV	HOXC5:NM_018953:exon1:c.A209C:p.H70P	ID=COSM548948;OCCURENCE=1(lung)	NA	B
chr12	54475448	54475448	G	C	ncRNA_exonic	LOC100240735	NA	NA	NA	rs4016762	NA
chr12	54475535	54475535	C	A	ncRNA_exonic	LOC100240735	NA	NA	NA	rs80341901	NA
chr12	54515276	54515276	A	G	ncRNA_exonic	FLJ12825	NA	NA	NA	rs200300428	NA
chr12	54515279	54515279	T	C	ncRNA_exonic	FLJ12825	NA	NA	NA	rs151283170	NA
chr12	54515281	54515281	A	G	ncRNA_exonic	FLJ12825	NA	NA	NA	rs145203398	NA
chr12	54515302	54515302	T	C	ncRNA_exonic	FLJ12825	NA	NA	NA	rs139731963	NA
chr12	54515663	54515663	C	G	ncRNA_exonic	FLJ12825	NA	NA	NA	rs77340285	NA
chr12	54802551	54802551	T	A	exonic	ITGA5	nonsynonymous SNV	ITGA5:NM_002205:exon6:c.A680T:p.Y227F	NA	NA	P
chr12	54905815	54905815	C	G	exonic	NCKAP1L	synonymous SNV	I_001184976:exon9:c.C717G:p.L239L,NCKAP1L:NM_005337:exon9:c.C	NA	rs2458409	NA
chr12	54973122	54973123	AT	-	UTR3	PPP1R1A	NA	NA	NA	rs67431222	NA
chr12	56295237	56295237	A	-	UTR3	WIBG	NA	NA	NA	NA	NA
chr12	56991346	56991346	G	A	UTR3	BAZ2A	NA	NA	NA	rs202206071	NA
chr12	56991346	56991346	-	CACACACA	UTR3	BAZ2A	NA	NA	NA	rs138865943	NA
chr12	57030026	57030026	T	C	UTR5	BAZ2A	NA	NA	NA	rs2255074	NA
chr12	57111185	57111185	A	G	intronic	NACA	NA	NA	NA	NA	NA
chr12	57607124	57607126	AAA	-	UTR3	LRP1	NA	NA	NA	rs147945774	NA
chr12	57978383	57978386	CACG	-	UTR3	KIF5A	NA	NA	NA	rs150797197	NA
chr12	57985204	57985204	C	T	exonic	PIP4K2C	synonymous SNV	/I_001146259:exon1:c.C132T:p.A44A,PIP4K2C:NM_001146260:exon1:c	NA	rs11537654	NA
chr12	58119120	58119120	-	AA	UTR3	AGAP2	NA	NA	NA	NA	NA
chr12	58166403	58166403	T	G	UTR5	METTL21B	NA	NA	NA	rs2291617	NA
chr12	63041688	63041688	-	A	UTR3	PPM1H	NA	NA	NA	NA	NA
chr12	64472873	64472873	G	A	exonic	SRGAP1	nonsynonymous SNV	SRGAP1:NM_020762:exon9:c.G1300A:p.E434K	NA	NA	D
chr12	64537561	64537561	A	-	UTR3	SRGAP1	NA	NA	NA	NA	NA
chr12	64538324	64538324	G	T	UTR3	SRGAP1	NA	NA	NA	rs789736	NA
chr12	64538615	64538615	A	C	UTR3	SRGAP1	NA	NA	NA	rs2643664	NA
chr12	64539337	64539337	T	-	UTR3	SRGAP1	NA	NA	NA	NA	NA
chr12	64746833	64746833	C	T	exonic	C12orf56	nonsynonymous SNV	001099676:exon2:c.G256A:p.D86N,C12orf56:NM_001170633:exon2:c.	NA	rs73118352	NA
chr12	69159402	69159402	A	G	UTR3	SLC35E3	NA	NA	NA	rs111267703	NA
chr12	69159404	69159404	A	G	UTR3	SLC35E3	NA	NA	NA	NA	NA
chr12	69159420	69159420	G	A	UTR3	SLC35E3	NA	NA	NA	NA	NA
chr12	69236874	69236874	T	-	UTR3	MDM2	NA	NA	NA	NA	NA
chr12	69246336	69246336	-	T	UTR3	CPM	NA	NA	NA	NA	NA
chr12	69667890	69667890	T	-	UTR3	CPSF6	NA	NA	NA	NA	NA
chr12	70213140	70213141	AG	-	UTR3	RAB3IP	NA	NA	NA	rs10578304	NA
chr12	71094955	71094955	C	T	exonic	PTPRR	nonsynonymous SNV	001207016:exon4:c.G538A:p.V180I,PTPRR:NM_001207015:exon6:c.G8	NA	rs35387004	B
chr12	72079955	72079955	T	C	UTR5	TMEM19	NA	NA	NA	NA	NA
chr12	72318795	72318795	A	-	UTR3	TBC1D15	NA	NA	NA	NA	NA
chr12	76747082	76747082	T	-	UTR3	OSBPL8	NA	NA	NA	rs34398462	NA
chr12	78530979	78530979	G	A	exonic	NAV3	synonymous SNV	NAV3:NM_014903:exon19:c.G4464A:p.Q1488Q	NA	rs1852464	NA
chr12	85285920	85285920	-	A	UTR5	SLC6A15	NA	NA	NA	rs36111430	NA
chr12	88591980	88591980	T	-	UTR3	TMTC3	NA	NA	NA	NA	NA
chr12	89914880	89914886	ATTATTA	-	UTR3	GALNT4,POC1B-GALNT4	NA	NA	NA	NA	NA
chr12	89984194	89984194	T	-	UTR3	ATP2B1	NA	NA	NA	NA	NA
chr12	93196422	93196422	T	G	exonic	EEA1	nonsynonymous SNV	EEA1:NM_003566:exon19:c.A2428C:p.K810Q	NA	rs10745623	B
chr12	93963873	93963873	C	T	ncRNA_UTR5	SOCS2	NA	NA	NA	rs2200160	NA
chr12	94631496	94631496	C	T	exonic	PLXNC1	synonymous SNV	PLXNC1:NM_005761:exon10:c.C2037T:p.N679N	NA	rs2230755	NA
chr12	99139154	99139156	TTT	-	UTR3	ANKS1B	NA	NA	NA	NA	NA
chr12	1.01E+08	1.01E+08	G	T	UTR3	SLC17A8	NA	NA	NA	NA	NA
chr12	1.01E+08	1.01E+08	TTTTTTTTT	-	UTR3	SLC17A8	NA	NA	NA	rs71091442	NA
chr12	1.01E+08	1.01E+08	G	A	exonic	ANO4	nonsynonymous SNV	ANO4:NM_178826:exon6:c.G455A:p.R152K	NA	NA	B
chr12	1.01E+08	1.01E+08	A	C	exonic	ANO4	nonsynonymous SNV	ANO4:NM_178826:exon6:c.A467C:p.Y156S	NA	rs201084190	D

chr12	1.02E+08	1.02E+08	C	A	exonic	ANO4	nonsynonymous SNV	ANO4:NM_178826:exon22:c.C2177A:p.A726D	NA	NA	P
chr12	1.02E+08	1.02E+08	G	A	exonic	SLC5A8	synonymous SNV	SLC5A8:NM_145913:exon14:c.C1662T:p.Y554Y	NA	rs2671444	NA
chr12	1.02E+08	1.02E+08	C	T	UTR3	UTP20	NA	NA	NA	rs3199856	NA
chr12	1.03E+08	1.03E+08	G	A	exonic	PARPBP	synonymous SNV	PARPBP:NM_017915:exon2:c.G138A:p.A46A	NA	rs2036771	NA
chr12	1.03E+08	1.03E+08	-	TG	UTR3	IGF1	NA	NA	NA	rs34621369	NA
chr12	1.04E+08	1.04E+08	G	A	exonic	STAB2	synonymous SNV	STAB2:NM_017564:exon6:c.G558A:p.A186A	NA	rs147053330	NA
chr12	1.04E+08	1.04E+08	C	T	exonic	STAB2	synonymous SNV	STAB2:NM_017564:exon31:c.C3339T:p.N1113N	)=COSM147590;OCCURENCE=1(stomac	rs703651	NA
chr12	1.04E+08	1.04E+08	A	G	UTR3	NT5DC3	NA	NA	NA	rs10735389	NA
chr12	1.04E+08	1.04E+08	-	A	UTR3	NT5DC3	NA	NA	NA	NA	NA
chr12	1.04E+08	1.04E+08	C	T	ncRNA_exonic	GNN	NA	NA	NA	rs11612613	NA
chr12	1.05E+08	1.05E+08	-	A	UTR3	ALDH1L2	NA	NA	NA	rs36037315	NA
chr12	1.06E+08	1.06E+08	TT	-	UTR3	NUAK1	NA	NA	NA	rs60661051	NA
chr12	1.06E+08	1.06E+08	-	ATGAAG	UTR3	NUAK1	NA	NA	NA	rs11283073	NA
chr12	1.07E+08	1.07E+08	T	-	UTR3	CKAP4	NA	NA	NA	NA	NA
chr12	1.07E+08	1.07E+08	A	G	exonic	CRY1	synonymous SNV	CRY1:NM_004075:exon5:c.T636C:p.G212G	NA	rs8192440	NA
chr12	1.08E+08	1.08E+08	A	G	UTR5	BTBD11	NA	NA	NA	rs1107438	NA
chr12	1.09E+08	1.09E+08	C	G	exonic	SELPLG	nonsynonymous SNV	_001206609:exon2:c.G611C:p.G204A,SELPLG:NM_003006:exon2:c.G5(	NA	NA	NA
chr12	1.1E+08	1.1E+08	G	A	exonic	ACACB	nonsynonymous SNV	ACACB:NM_001093:exon46:c.G6421A:p.V2141I	NA	rs2075260	NA
chr12	1.1E+08	1.1E+08	TT	-	UTR3	MMAB	NA	NA	NA	NA	NA
chr12	1.12E+08	1.12E+08	-	T	UTR3	SH2B3	NA	NA	NA	NA	NA
chr12	1.12E+08	1.12E+08	T	-	UTR3	ATXN2	NA	NA	NA	rs11348701	NA
chr12	1.13E+08	1.13E+08	T	C	UTR3	TRAFD1	NA	NA	NA	rs149284206	NA
chr12	1.13E+08	1.13E+08	T	G	exonic	HECTD4	nonsynonymous SNV	HECTD4:NM_001109662:exon61:c.A10030C:p.T3344P	NA	NA	NA
chr12	1.13E+08	1.13E+08	ACAC	-	UTR3	RPH3A	NA	NA	NA	rs67569142	NA
chr12	1.13E+08	1.13E+08	T	C	exonic	OAS3	synonymous SNV	OAS3:NM_006187:exon6:c.T1314C:p.I438I	NA	rs2285932	NA
chr12	1.14E+08	1.14E+08	G	A	UTR3	RBM19	NA	NA	NA	rs7312304	NA
chr12	1.14E+08	1.14E+08	G	C	exonic	RBM19	synonymous SNV	3G:p.T606T,RBM19:NM_001146699:exon15:c.C1818G:p.T606T,RBM19	NA	rs2290790	NA
chr12	1.18E+08	1.18E+08	A	G	exonic	NOS1	synonymous SNV	_001204214:exon12:c.T1194C:p.I398I,NOS1:NM_000620:exon13:c.T22	NA	rs2293054	NA
chr12	1.18E+08	1.18E+08	G	C	UTR3	KSR2	NA	NA	NA	NA	NA
chr12	1.18E+08	1.18E+08	C	G	UTR3	KSR2	NA	NA	NA	NA	NA
chr12	1.18E+08	1.18E+08	C	A	UTR3	KSR2	NA	NA	NA	rs201802824	NA
chr12	1.18E+08	1.18E+08	A	G	UTR3	KSR2	NA	NA	NA	rs11068502	NA
chr12	1.19E+08	1.19E+08	C	T	exonic	TAOK3	nonsynonymous SNV	TAOK3:NM_016281:exon4:c.G140A:p.S47N	NA	rs428073	B
chr12	1.19E+08	1.19E+08	C	T	UTR3	SUDS3	NA	NA	NA	NA	NA
chr12	1.2E+08	1.2E+08	A	-	UTR3	SRRM4	NA	NA	NA	rs11305482	NA
chr12	1.2E+08	1.2E+08	G	T	exonic	CCDC64	nonsynonymous SNV	CCDC64:NM_207311:exon2:c.G438T:p.E146D	NA	NA	D
chr12	1.21E+08	1.21E+08	A	-	UTR3	MSI1	NA	NA	NA	NA	NA
chr12	1.21E+08	1.21E+08	-	A	UTR3	GATC,SRSF9	NA	NA	NA	rs34136998	NA
chr12	1.21E+08	1.21E+08	T	G	UTR5	DYNLL1	NA	NA	NA	rs12857	NA
chr12	1.21E+08	1.21E+08	G	A	ncRNA_exonic	HNF1A-AS1	NA	NA	NA	rs2254779	NA
chr12	1.22E+08	1.22E+08	T	-	UTR3	TMEM120B	NA	NA	NA	NA	NA
chr12	1.22E+08	1.22E+08	C	A	UTR3	TMEM120B	NA	NA	NA	NA	NA
chr12	1.22E+08	1.22E+08	A	C	exonic	SETD1B	synonymous SNV	SETD1B:NM_015048:exon5:c.A1227C:p.P409P	NA	NA	NA
chr12	1.23E+08	1.23E+08	A	G	UTR3	HCAR1	NA	NA	NA	rs7313367	NA
chr12	1.23E+08	1.23E+08	C	A	exonic	ARL6IP4	nonsynonymous SNV	)03A:p.Q335K,ARL6IP4:NM_016638:exon6:c.C970A:p.Q324K,ARL6IP4:I	NA	NA	D
chr12	1.24E+08	1.24E+08	T	C	UTR3	SETD8	NA	NA	NA	rs61955129	NA
chr12	1.24E+08	1.24E+08	G	A	UTR3	SETD8	NA	NA	NA	rs61953481	NA
chr12	1.24E+08	1.24E+08	A	G	UTR3	SETD8	NA	NA	NA	rs142906941	NA
chr12	1.25E+08	1.25E+08	T	C	exonic	NCOR2	synonymous SNV	:p.R1643R,NCOR2:NM_001206654:exon35:c.A4929G:p.R1643R,NCOR:	NA	rs2230942	NA
chr12	1.25E+08	1.25E+08	C	T	exonic	NCOR2	synonymous SNV	A:p.L1531L,NCOR2:NM_001206654:exon33:c.G4593A:p.L1531L,NCOR2	NA	rs1263992	NA
chr12	1.25E+08	1.25E+08	A	G	exonic	NCOR2	synonymous SNV	3C:p.A901A,NCOR2:NM_001206654:exon21:c.T2703C:p.A901A,NCOR2	NA	rs7961196	NA
chr12	1.29E+08	1.29E+08	G	A	exonic	TMEM132C	nonsynonymous SNV	TMEM132C:NM_001136103:exon5:c.G1330A:p.V444I	NA	rs4272850	NA
chr12	1.29E+08	1.29E+08	A	G	UTR3	TMEM132C	NA	NA	NA	NA	NA
chr12	1.31E+08	1.31E+08	-	T	ncRNA_exonic	LOC100190940	NA	NA	NA	NA	NA
chr12	1.31E+08	1.31E+08	G	T	UTR5	GPR133	NA	NA	NA	rs56090874	NA
chr12	1.32E+08	1.32E+08	G	T	exonic	ULK1	nonsynonymous SNV	ULK1:NM_003565:exon22:c.G2234T:p.G745V	NA	rs34936984	D
chr12	1.32E+08	1.32E+08	A	G	exonic	ULK1	nonsynonymous SNV	ULK1:NM_003565:exon23:c.A2446G:p.T816A	NA	rs11609348	NA
chr12	1.33E+08	1.33E+08	-	TT	UTR3	DDX51	NA	NA	NA	rs112928855	NA
chr12	1.33E+08	1.33E+08	T	C	exonic	P2RX2	synonymous SNV	/_016318:exon4:c.T396C:p.T132T,P2RX2:NM_170682:exon5:c.T468C:		NA	rs7964634	NA
chr12	1.33E+08	1.33E+08	AA	-	UTR3	GOLGA3	NA	NA	NA	rs139681552	NA
chr12	1.33E+08	1.33E+08	T	G	exonic	GOLGA3	nonsynonymous SNV	GOLGA3:NM_005895:exon23:c.A4178C:p.N1393T	NA	NA	B
chr12	1.34E+08	1.34E+08	A	G	UTR3	ZNF605	NA	NA	NA	rs61951591	NA
chr12	1.34E+08	1.34E+08	G	T	UTR3	ZNF26	NA	NA	NA	NA	NA
chr12	1.34E+08	1.34E+08	G	A	UTR5	ZNF84	NA	NA	NA	rs10870574	NA
chr13	19409503	19409503	T	C	ncRNA_exonic	ANKRD20A9P	NA	NA	NA	rs141688139	NA
chr13	19409611	19409611	T	C	ncRNA_exonic	ANKRD20A9P	NA	NA	NA	rs200316004	NA

chr13	19418641	19418641	T	-	ncRNA_exonic	ANKRD20A9P	NA	NA	NA	NA	NA
chr13	19431858	19431858	T	-	ncRNA_exonic	ANKRD20A9P	NA	NA	NA	rs33949959	NA
chr13	19442031	19442031	C	A	ncRNA_exonic	ANKRD20A9P	NA	NA	NA	rs2997166	NA
chr13	21348028	21348028	A	C	UTR5	N6AMT2	NA	NA	NA	NA	NA
chr13	23755127	23755127	G	A	UTR5	SGCG	NA	NA	NA	rs4770403	NA
chr13	23808782	23808782	T	C	exonic	SGCG	synonymous SNV	SGCG:NM_000231:exon3:c.T228C:p.D76D	NA	rs1800350	NA
chr13	25161438	25161438	T	A	ncRNA_exonic	TPTE2P6	NA	NA	NA	rs3869320	NA
chr13	25356053	25356053	T	C	exonic	RNF17	synonymous SNV	I_001184993:exon6:c.T582C:p.F194F,RNF17:NM_031277:exon6:c.T58	NA	rs9707144	NA
chr13	25505593	25505593	T	G	ncRNA_exonic	TPTE2P1	NA	NA	NA	rs9553473	NA
chr13	26125490	26125490	G	A	exonic	ATP8A2	synonymous SNV	ATP8A2:NM_016529:exon11:c.G906A:p.A302A	NA	rs9581388	NA
chr13	27847807	27847807	G	T	UTR3	RASL11A	NA	NA	NA	NA	NA
chr13	28867525	28867529	TTTTT	-	UTR3	PAN3	NA	NA	NA	NA	NA
chr13	28867565	28867565	A	G	UTR3	PAN3	NA	NA	NA	NA	NA
chr13	28874659	28874666	CACACACA	-	UTR3	FLT1	NA	NA	NA	rs200186804	NA
chr13	28961961	28961961	G	A	UTR3	FLT1	NA	NA	NA	rs199630957	NA
chr13	30779887	30779887	-	TG	UTR3	KATNAL1	NA	NA	NA	NA	NA
chr13	31035091	31035091	T	-	UTR3	HMGB1	NA	NA	NA	rs138159768	NA
chr13	31735759	31735759	G	A	UTR5	HSPH1	NA	NA	NA	rs2280059	NA
chr13	32745397	32745397	G	A	exonic	FRY	nonsynonymous SNV	FRY:NM_023037:exon18:c.G2141A:p.R714K	NA	NA	B
chr13	32890572	32890572	G	A	UTR5	BRCA2	NA	NA	NA	rs1799943	NA
chr13	36744800	36744800	G	A	exonic	CCDC169-SOHLH2,SOHLH2	synonymous SNV	6:exon10:c.C1125T:p.Y375Y,CCDC169-SOHLH2:NM_001198910:exon1	=COSM147666;OCCURENCE=1(stomac	rs2296967	NA
chr13	36805290	36805290	A	-	UTR3	CCDC169	NA	NA	NA	rs67328439	NA
chr13	36920918	36920918	C	G	ncRNA_UTR5	SPG20	NA	NA	NA	rs9531842	NA
chr13	37271429	37271430	CA	-	UTR3	SERTM1	NA	NA	NA	rs58941896	NA
chr13	39623185	39623185	T	-	UTR3	NHLRC3	NA	NA	NA	NA	NA
chr13	40922821	40922821	A	-	ncRNA_exonic	LINC00598	NA	NA	NA	rs112830018	NA
chr13	41133064	41133064	-	T	UTR3	FOXO1	NA	NA	NA	NA	NA
chr13	41385658	41385658	C	A	ncRNA_UTR3	SLC25A15	NA	NA	NA	rs41515050	NA
chr13	41703785	41703785	C	T	UTR3	KBTBD6	NA	NA	NA	NA	NA
chr13	43148313	43148313	C	G	UTR5	TNFSF11	NA	NA	NA	rs9533157	NA
chr13	43639845	43639845	A	C	exonic	DNAJC15	synonymous SNV	DNAJC15:NM_013238:exon2:c.A132C:p.G44G	NA	rs3783044	NA
chr13	43681661	43681666	ACACAC	-	UTR3	DNAJC15	NA	NA	NA	rs72088427	NA
chr13	43787681	43787683	TTT	-	UTR3	ENOX1	NA	NA	NA	NA	NA
chr13	45915481	45915481	A	C	ncRNA_exonic	TPT1-AS1	NA	NA	NA	rs12872310	NA
chr13	45915500	45915500	C	T	ncRNA_exonic	TPT1-AS1	NA	NA	NA	rs12872567	NA
chr13	46537757	46537757	G	A	UTR3	ZC3H13	NA	NA	NA	rs17350833	NA
chr13	48669146	48669146	A	C	exonic	MED4	synonymous SNV	MED4:NM_014166:exon1:c.T69G:p.G23G	NA	NA	NA
chr13	50102992	50102992	T	C	UTR3	PHF11	NA	NA	NA	rs3033	NA
chr13	50466774	50466774	G	A	ncRNA_exonic	CTAGE10P	NA	NA	NA	rs58542163	NA
chr13	50466780	50466780	C	T	ncRNA_exonic	CTAGE10P	NA	NA	NA	rs189224500	NA
chr13	50588496	50588496	T	C	ncRNA_UTR3	TRIM13	NA	NA	NA	rs199807501	NA
chr13	52390790	52390791	TG	-	ncRNA_exonic	LINC00282	NA	NA	NA	rs151038091	NA
chr13	58302675	58302676	GC	-	UTR3	PCDH17	NA	NA	NA	NA	NA
chr13	58302684	58302684	T	A	UTR3	PCDH17	NA	NA	NA	NA	NA
chr13	60240491	60240491	-	A	UTR3	DIAPH3	NA	NA	NA	NA	NA
chr13	70713512	70713512	A	G	ncRNA_exonic	ATXN8OS	NA	NA	NA	rs2021426	NA
chr13	73329358	73329358	G	A	UTR3	BORA	NA	NA	NA	rs10887	NA
chr13	73369642	73369642	A	G	exonic	PIBF1	nonsynonymous SNV	PIBF1:NM_006346:exon4:c.A499G:p.I167V	NA	rs1372000	B
chr13	77458312	77458312	G	C	UTR3	KCTD12	NA	NA	NA	NA	NA
chr13	77619037	77619037	T	-	UTR3	MYCBP2	NA	NA	NA	rs56131306	NA
chr13	78216915	78216915	C	T	exonic	SCEL	synonymous SNV	396T:p.H632H,SCEL:NM_003843:exon31:c.C1962T:p.H654H,SCEL:NM_	NA	rs2813739	NA
chr13	79894106	79894106	G	-	UTR3	RBM26	NA	NA	NA	NA	NA
chr13	79894118	79894118	C	T	UTR3	RBM26	NA	NA	NA	NA	NA
chr13	79894162	79894162	A	T	UTR3	RBM26	NA	NA	NA	NA	NA
chr13	84451509	84451509	A	-	UTR3	SLITRK1	NA	NA	NA	rs66938370	NA
chr13	96230279	96230279	T	G	UTR3	CLDN10	NA	NA	NA	rs1325774	NA
chr13	96232591	96232591	A	-	UTR3	DZIP1	NA	NA	NA	NA	NA
chr13	98118464	98118464	A	-	UTR3	RAP2A	NA	NA	NA	rs61387793	NA
chr13	1.01E+08	1.01E+08	T	-	UTR3	CLYBL	NA	NA	NA	NA	NA
chr13	1.01E+08	1.01E+08	TATTTAT	-	UTR3	GGACT	NA	NA	NA	rs10581954	NA
chr13	1.03E+08	1.03E+08	TTTTTT	-	UTR3	TPP2	NA	NA	NA	rs202012610	NA
chr13	1.03E+08	1.03E+08	AA	-	UTR3	BIVM	NA	NA	NA	NA	NA
chr13	1.04E+08	1.04E+08	-	T	ncRNA_exonic	METTL21EP	NA	NA	NA	NA	NA
chr13	1.04E+08	1.04E+08	CA	-	ncRNA_exonic	METTL21EP	NA	NA	NA	NA	NA
chr13	1.09E+08	1.09E+08	CTCT	-	UTR5	FAM155A	NA	NA	NA	rs35028871	NA
chr13	1.1E+08	1.1E+08	T	C	exonic	MYO16	synonymous SNV	001198950:exon12:c.T1413C:p.I471I,MYO16:NM_015011:exon12:c.T1	NA	rs9559428	NA

chr13	1.11E+08	1.11E+08	G	A	exonic	COL4A2	nonsynonymous SNV	COL4A2:NM_001846:exon22:c.G1550A:p.R517K		NA	rs7990383	B
chr13	1.11E+08	1.11E+08	T	C	exonic	CARS2	synonymous SNV	CARS2:NM_024537:exon8:c.A852G:p.E284E		NA	rs4628819	NA
chr13	1.12E+08	1.12E+08	C	T	exonic	ARHGEF7	synonymous SNV	C198T:p.S66S,ARHGEF7:NM_003899:exon4:c.C198T:p.S66S,ARHGEF7:I		NA	rs41275140	NA
chr13	1.12E+08	1.12E+08	AA	-	UTR3	ARHGEF7	NA	NA		NA	rs202074614	NA
chr13	1.14E+08	1.14E+08	ACAC	-	UTR3	ATP11A	NA	NA		NA	rs72007310	NA
chr13	1.14E+08	1.14E+08	AT	-	UTR3	ATP11A	NA	NA		NA	rs3832908	NA
chr13	1.14E+08	1.14E+08	C	A	UTR3	DCUN1D2	NA	NA		NA	rs9577553	NA
chr13	1.14E+08	1.14E+08	C	T	exonic	TMCO3	nonsynonymous SNV	TMCO3:NM_017905:exon9:c.C1414T:p.L472F		NA	rs77834374	P
chr13	1.14E+08	1.14E+08	A	T	exonic	TMCO3	nonsynonymous SNV	TMCO3:NM_017905:exon9:c.A1419T:p.L473F		NA	NA	P
chr13	1.14E+08	1.14E+08	-	GTGTGCAC	ncRNA_exonic	LINC00552	NA	NA		NA	NA	NA
chr14	20811332	20811332	C	T	ncRNA_exonic	RPPH1	NA	NA		NA	rs3093872	NA
chr14	20811476	20811476	-	A	ncRNA_exonic	RPPH1	NA	NA		NA	NA	NA
chr14	20822308	20822308	A	G	exonic	PARP2	nonsynonymous SNV	_001042618:exon8:c.A665G:p.D222G,PARP2:NM_005484:exon8:c.A70		NA	rs3093921	P
chr14	21965670	21965670	G	T	UTR3	TOX4	NA	NA		NA	NA	NA
chr14	21965672	21965672	T	G	UTR3	TOX4	NA	NA		NA	rs28506904	NA
chr14	21965689	21965689	T	G	UTR3	TOX4	NA	NA		NA	NA	NA
chr14	23304057	23304057	A	-	UTR3	MRPL52	NA	NA		NA	rs56341631	NA
chr14	23352465	23352470	GCACAC	-	UTR5	REM2	NA	NA		NA	rs72363604	NA
chr14	23530622	23530622	T	C	exonic	ACIN1	synonymous SNV	G:p.S403S,ACIN1:NM_001164815:exon16:c.A3363G:p.S1121S,ACIN1:N		NA	rs60168438	NA
chr14	23652346	23652346	-	AA	UTR5	SLC7A8	NA	NA		NA	NA	NA
chr14	23795205	23795205	T	-	UTR3	BCL2L2-PABPN1,PABPN1	NA	NA		NA	rs71119016	NA
chr14	24408866	24408866	G	A	ncRNA_exonic	DHRS4-AS1	NA	NA		NA	rs1885810	NA
chr14	24409799	24409799	A	G	ncRNA_exonic	DHRS4-AS1	NA	NA		NA	rs113551151	NA
chr14	24410262	24410262	C	G	ncRNA_exonic	DHRS4-AS1	NA	NA		NA	rs58941536	NA
chr14	24837338	24837338	G	-	UTR5	NFATC4	NA	NA		NA	rs3215609	NA
chr14	24899194	24899194	C	T	UTR5	KHNYN	NA	NA		NA	rs10146759	NA
chr14	25103414	25103414	G	A	UTR5	GZMB	NA	NA	)=COSM147760;OCCURENCE=1(stomacl		rs2273844	NA
chr14	31344039	31344039	A	G	UTR5	COCH	NA	NA		NA	rs11555426	NA
chr14	31364292	31364292	G	A	UTR3	STRN3	NA	NA		NA	NA	NA
chr14	31364323	31364323	G	A	UTR3	STRN3	NA	NA		NA	NA	NA
chr14	31647241	31647241	G	A	exonic	HECTD1	synonymous SNV	HECTD1:NM_015382:exon3:c.C360T:p.A120A		NA	rs2274201	NA
chr14	34272917	34272919	AAA	-	UTR3	NPAS3	NA	NA		NA	rs146767978	NA
chr14	34394149	34394149	-	AA	UTR3	EGLN3	NA	NA		NA	NA	NA
chr14	38678356	38678356	G	A	UTR5	SSTR1	NA	NA		NA	rs12437095	NA
chr14	39867658	39867658	G	T	UTR3	FBXO33	NA	NA		NA	rs201516221	NA
chr14	39867669	39867669	C	T	UTR3	FBXO33	NA	NA		NA	rs202105812	NA
chr14	50092078	50092078	A	-	UTR3	DNAAF2	NA	NA		NA	NA	NA
chr14	50361154	50361154	G	T	UTR3	ARF6	NA	NA		NA	NA	NA
chr14	50361173	50361174	TT	-	UTR3	ARF6	NA	NA		NA	NA	NA
chr14	50448984	50448984	A	C	UTR3	C14orf182	NA	NA		NA	rs7161196	NA
chr14	53241309	53241309	-	T	UTR3	STYX	NA	NA		NA	NA	NA
chr14	54416602	54416602	T	A	UTR3	BMP4	NA	NA		NA	rs76335800	NA
chr14	55429883	55429883	C	A	exonic	WDHD1	nonsynonymous SNV	01008396:exon18:c.G1949T:p.C650F,WDHD1:NM_007086:exon19:c.G		NA	NA	D
chr14	55429886	55429886	G	A	exonic	WDHD1	nonsynonymous SNV	)01008396:exon18:c.C1946T:p.S649F,WDHD1:NM_007086:exon19:c.C		NA	NA	D
chr14	55533924	55533925	TT	-	UTR3	MAPK1IP1L	NA	NA		NA	NA	NA
chr14	55864146	55864146	G	A	exonic	ATG14	synonymous SNV	ATG14:NM_014924:exon2:c.C228T:p.I76I		NA	rs61743178	NA
chr14	56249141	56249141	-	T	ncRNA_exonic	LINC00520	NA	NA		NA	NA	NA
chr14	56767165	56767165	T	-	UTR3	PELI2	NA	NA		NA	NA	NA
chr14	57396821	57396821	T	C	ncRNA_exonic	OTX2-AS1	NA	NA		NA	NA	NA
chr14	57396858	57396858	T	C	ncRNA_exonic	OTX2-AS1	NA	NA		NA	rs199531630	NA
chr14	57396873	57396873	T	C	ncRNA_exonic	OTX2-AS1	NA	NA		NA	rs12894513	NA
chr14	57396877	57396877	T	C	ncRNA_exonic	OTX2-AS1	NA	NA		NA	NA	NA
chr14	57396889	57396892	TTTC	-	ncRNA_exonic	OTX2-AS1	NA	NA		NA	rs150682540	NA
chr14	57396893	57396893	T	C	ncRNA_exonic	OTX2-AS1	NA	NA		NA	NA	NA
chr14	57396901	57396901	T	C	ncRNA_exonic	OTX2-AS1	NA	NA		NA	NA	NA
chr14	57396905	57396905	T	C	ncRNA_exonic	OTX2-AS1	NA	NA		NA	NA	NA
chr14	57396919	57396919	T	C	ncRNA_exonic	OTX2-AS1	NA	NA		NA	rs28595791	NA
chr14	57396928	57396929	TT	-	ncRNA_exonic	OTX2-AS1	NA	NA		NA	NA	NA
chr14	57396935	57396935	C	T	ncRNA_exonic	OTX2-AS1	NA	NA		NA	NA	NA
chr14	57396937	57396938	CC	-	ncRNA_exonic	OTX2-AS1	NA	NA		NA	NA	NA
chr14	60759367	60759367	-	T	UTR3	PPM1A	NA	NA		NA	NA	NA
chr14	61177729	61177729	-	TATATA	UTR3	SIX4	NA	NA		NA	rs142920801	NA
chr14	61190053	61190053	T	G	exonic	SIX4	nonsynonymous SNV	SIX4:NM_017420:exon1:c.A740C:p.Y247S		NA	NA	D
chr14	61745694	61745697	CACA	-	UTR3	TMEM30B	NA	NA		NA	NA	NA
chr14	64693058	64693058	C	G	UTR3	SYNE2	NA	NA		NA	NA	NA
chr14	64937308	64937317	AAAAAAAA	-	UTR3	AKAP5	NA	NA		NA	NA	NA

chr14	64937889	64937889	G	T	UTR3	AKAP5	NA	NA	NA	rs75057565	NA
chr14	65213611	65213611	A	-	UTR3	SPTB	NA	NA	NA	NA	NA
chr14	65399263	65399263	A	G	UTR3	CHURC1	NA	NA	NA	rs8943	NA
chr14	65399306	65399306	G	A	UTR3	CHURC1	NA	NA	NA	rs8984	NA
chr14	65400265	65400265	C	T	UTR3	CHURC1	NA	NA	NA	rs1064108	NA
chr14	65400745	65400745	-	A	UTR3	CHURC1	NA	NA	NA	rs35743220	NA
chr14	65400785	65400786	GT	-	UTR3	CHURC1	NA	NA	NA	rs141703694	NA
chr14	68144049	68144049	G	A	UTR3	RDH11	NA	NA	NA	NA	NA
chr14	69255652	69255652	G	T	UTR3	ZFP36L1	NA	NA	NA	rs6739	NA
chr14	69255656	69255658	GGT	-	UTR3	ZFP36L1	NA	NA	NA	rs138429203	NA
chr14	69255660	69255660	G	C	UTR3	ZFP36L1	NA	NA	NA	rs10656	NA
chr14	69352230	69352230	G	A	exonic	ACTN1	synonymous SNV	p.L433L,ACTN1:NM_001130004:exon12:c.C1297T:p.L433L,ACTN1:NM_	NA	rs15993	NA
chr14	69926273	69926273	-	T	UTR3	SLC39A9	NA	NA	NA	NA	NA
chr14	70233152	70233152	-	A	ncRNA_exonic	LOC100289511	NA	NA	NA	rs138453813	NA
chr14	70546744	70546744	G	A	UTR5	SLC8A3	NA	NA	NA	rs72729857	NA
chr14	70834315	70834315	G	A	UTR3	SYNJ2BP	NA	NA	NA	NA	NA
chr14	70834325	70834325	C	T	UTR3	SYNJ2BP	NA	NA	NA	NA	NA
chr14	70834332	70834332	G	A	UTR3	SYNJ2BP	NA	NA	NA	NA	NA
chr14	73422350	73422350	T	G	exonic	DCAF4	synonymous SNV	.S275S,DCAF4:NM_001163508:exon12:c.T1107G:p.S369S,DCAF4:NM_	NA	rs2806034	NA
chr14	73459917	73459917	C	T	exonic	ZFYVE1	synonymous SNV	ZFYVE1:NM_021260:exon4:c.G1137A:p.E379E	NA	rs2333016	NA
chr14	73959703	73959703	-	T	UTR3	C14orf169	NA	NA	NA	NA	NA
chr14	74168648	74168649	AA	-	UTR3	DNAL1	NA	NA	NA	rs147918155	NA
chr14	74183386	74183391	AAAAAA	-	UTR3	ELMSAN1	NA	NA	NA	NA	NA
chr14	74815239	74815239	C	T	UTR5	VRTN	NA	NA	NA	rs2286425	NA
chr14	75388803	75388803	G	A	UTR5	RPS6KL1	NA	NA	NA	rs7158047	NA
chr14	77491190	77491190	-	AG	UTR3	IRF2BPL	NA	NA	NA	rs200696373	NA
chr14	77599140	77599140	-	TCTCTCTC	UTR3	ZDHHC22	NA	NA	NA	NA	NA
chr14	77648238	77648240	GTG	-	splicing	TMEM63C	NA	NA	NA	rs35484514	NA
chr14	77973676	77973676	T	-	UTR3	SPTLC2	NA	NA	NA	NA	NA
chr14	78390839	78390839	T	G	exonic	ADCK1	nonsynonymous SNV	_001142545:exon7:c.T694G:p.F232V,ADCK1:NM_020421:exon8:c.T89	NA	NA	D
chr14	78390880	78390880	T	C	exonic	ADCK1	synonymous SNV	_001142545:exon7:c.T735C:p.N245N,ADCK1:NM_020421:exon8:c.T93	NA	rs2302944	NA
chr14	80678116	80678116	G	T	ncRNA_intronic	DIO2-AS1	NA	NA	NA	NA	NA
chr14	81940588	81940588	-	A	UTR3	SEL1L	NA	NA	NA	NA	NA
chr14	81942830	81942830	T	-	UTR3	SEL1L	NA	NA	NA	NA	NA
chr14	88647752	88647752	A	G	UTR3	KCNK10	NA	NA	NA	rs72698030	NA
chr14	88852166	88852166	G	A	exonic	SPATA7	nonsynonymous SNV	:NM_001040428:exon1:c.G4A:p.D2N,SPATA7:NM_018418:exon1:c.G4	NA	rs4904448	P
chr14	89622979	89622979	-	T	UTR3	FOXN3	NA	NA	NA	NA	NA
chr14	89626561	89626561	G	A	UTR3	FOXN3	NA	NA	NA	rs11627037	NA
chr14	89627901	89627904	GTGT	-	UTR3	FOXN3	NA	NA	NA	NA	NA
chr14	92041294	92041294	A	G	intergenic	1(dist=64650),CATSPERB(dist=	NA	NA	NA	rs1743066	NA
chr14	92582472	92582472	G	A	UTR3	NDUFB1	NA	NA	NA	rs13350	NA
chr14	94749972	94749972	A	G	UTR3	SERPINA10	NA	NA	NA	rs12793	NA
chr14	94776221	94776221	A	C	exonic	SERPINA6	nonsynonymous SNV	SERPINA6:NM_001756:exon3:c.T736G:p.S246A	NA	rs2228541	B
chr14	94912896	94912896	T	G	exonic	SERPINA11	nonsynonymous SNV	SERPINA11:NM_001080451:exon3:c.A689C:p.E230A	NA	rs57740714	D
chr14	94933709	94933709	C	T	exonic	SERPINA9	synonymous SNV	_175739:exon3:c.G693A:p.E231E,SERPINA9:NM_001042518:exon4:c.(	NA	rs6575433	NA
chr14	95107973	95107973	C	T	ncRNA_exonic	SERPINA13P	NA	NA	NA	rs4905226	NA
chr14	95655319	95655319	-	A	UTR3	CLMN	NA	NA	NA	NA	NA
chr14	95657743	95657743	-	A	UTR3	CLMN	NA	NA	NA	NA	NA
chr14	96000550	96000550	A	C	ncRNA_exonic	SNHG10	NA	NA	NA	NA	NA
chr14	96152836	96152836	T	G	exonic	TCL1B	nonsynonymous SNV	TCL1B:NM_004918:exon1:c.T32G:p.V11G	NA	NA	B
chr14	96871104	96871104	G	A	exonic	AK7	nonsynonymous SNV	AK7:NM_152327:exon3:c.G305A:p.R102Q	NA	rs2275554	P
chr14	1.01E+08	1.01E+08	A	T	UTR5	WARS	NA	NA	NA	rs7143006	NA
chr14	1.05E+08	1.05E+08	G	A	exonic	ASPG	nonsynonymous SNV	ASPG:NM_001080464:exon2:c.G161A:p.R54H	NA	rs201325717	NA
chr14	1.05E+08	1.05E+08	T	C	exonic	KIF26A	synonymous SNV	KIF26A:NM_015656:exon12:c.T2487C:p.G829G	NA	rs4906422	NA
chr14	1.05E+08	1.05E+08	C	T	UTR5	AKT1	NA	NA	NA	rs10138227	NA
chr14	1.05E+08	1.05E+08	A	G	UTR3	ZBTB42	NA	NA	NA	rs140954212	NA
chr14	1.05E+08	1.05E+08	C	T	ncRNA_exonic	LINC00638	NA	NA	NA	rs12894160	NA
chr14	1.05E+08	1.05E+08	C	G	exonic	AHNAK2	nonsynonymous SNV	AHNAK2:NM_138420:exon7:c.G9625C:p.V3209L	NA	rs201181175	NA
chr14	1.05E+08	1.05E+08	T	C	exonic	AHNAK2	nonsynonymous SNV	AHNAK2:NM_138420:exon7:c.A6509G:p.K2170R	NA	rs201127689	NA
chr14	1.05E+08	1.05E+08	G	C	exonic	AHNAK2	synonymous SNV	AHNAK2:NM_138420:exon7:c.C6507G:p.G2169G	NA	rs146449084	NA
chr14	1.05E+08	1.05E+08	T	C	exonic	AHNAK2	synonymous SNV	AHNAK2:NM_138420:exon7:c.A6291G:p.K2097K	NA	rs201263008	NA
chr14	1.05E+08	1.05E+08	G	C	exonic	AHNAK2	nonsynonymous SNV	AHNAK2:NM_138420:exon7:c.C6133G:p.L2045V	NA	rs11850885	NA
chr14	1.06E+08	1.06E+08	T	G	exonic	JAG2	nonsynonymous SNV	_145159:exon22:c.A2755C:p.T919P,JAG2:NM_002226:exon23:c.A2869	NA	NA	D
chr14	1.06E+08	1.06E+08	C	A	UTR3	BRF1	NA	NA	NA	rs8270	NA
chr14	1.07E+08	1.07E+08	G	C	ncRNA_exonic	LINC00221	NA	NA	NA	rs10873578	NA
chr14	1.07E+08	1.07E+08	C	G	ncRNA_exonic	LINC00221	NA	NA	ID=COSN393151;OCCURENCE=1(lung)	rs11160980	NA

chr14	1.07E+08	1.07E+08	T	G	ncRNA_exonic	LINC00221	NA	NA	NA	rs11160981	NA
chr14	1.07E+08	1.07E+08	A	-	ncRNA_exonic	LINC00221	NA	NA	NA	rs140975989	NA
chr15	20740129	20740129	G	A	exonic	GOLGA6L6	nonsynonymous SNV	GOLGA6L6:NM_001145004:exon8:c.C1621T:p.R541W	NA	rs199932545	NA
chr15	21127120	21127120	C	T	ncRNA_exonic	NF1P2	NA	NA	NA	rs2672341	NA
chr15	21134563	21134563	A	G	ncRNA_exonic	NF1P2	NA	NA	NA	rs145897182	NA
chr15	21938564	21938564	A	G	ncRNA_exonic	LOC646214	NA	NA	NA	rs4383099	NA
chr15	21939417	21939417	T	C	ncRNA_exonic	LOC646214	NA	NA	NA	rs28478588	NA
chr15	21940430	21940430	-	TTGTTTATC	ncRNA_exonic	LOC646214	NA	NA	NA	rs6145499	NA
chr15	22703218	22703218	A	G	ncRNA_exonic	GOLGA8DP	NA	NA	NA	rs1822555	NA
chr15	22743252	22743252	T	C	exonic	GOLGA6L1	nonsynonymous SNV	GOLGA6L1:NM_001001413:exon8:c.T1637C:p.M546T	NA	NA	NA
chr15	23005380	23005380	-	C	UTR3	NIPA2	NA	NA	NA	rs35420735	NA
chr15	23441131	23441131	C	A	ncRNA_exonic	GOLGA8EP	NA	NA	NA	rs112118972	B
chr15	23446011	23446011	C	T	ncRNA_exonic	GOLGA8EP	NA	NA	NA	rs2120419	NA
chr15	24414994	24414994	T	C	ncRNA_exonic	PWRN2	NA	NA	NA	rs4029933	NA
chr15	24415020	24415020	T	C	ncRNA_exonic	PWRN2	NA	NA	NA	rs3950711	NA
chr15	24813154	24813154	T	C	ncRNA_exonic	PWRN1	NA	NA	NA	rs28574764	NA
chr15	28096538	28096538	A	G	exonic	OCA2	synonymous SNV	OCA2:NM_000275:exon22:c.T2328C:p.A776A	NA	rs1800419	NA
chr15	28502279	28502279	A	G	exonic	HERC2	synonymous SNV	HERC2:NM_004667:exon17:c.T2445C:p.G815G	NA	rs11631797	NA
chr15	28566548	28566548	T	C	exonic	HERC2	nonsynonymous SNV	HERC2:NM_004667:exon2:c.A32G:p.Q11R	NA	rs201990459	P
chr15	29002606	29002606	C	T	ncRNA_exonic	WHAMMP2	NA	NA	NA	rs200451851	NA
chr15	29992941	29992941	G	A	UTR3	TJP1	NA	NA	NA	NA	NA
chr15	30664453	30664453	C	T	exonic	CHRFAM7A	synonymous SNV	M_148911:exon6:c.G147A:p.T49T,CHRFAM7A:NM_139320:exon7:c.G	NA	rs1042722	NA
chr15	31669511	31669511	-	AG	UTR3	KLF13	NA	NA	NA	rs138437319	NA
chr15	33261185	33261185	G	A	exonic	FMN1	nonsynonymous SNV	01103184:exon4:c.C2048T:p.P683L,FMN1:NM_001277313:exon8:c.C2	NA	rs117804335	NA
chr15	34523794	34523794	T	-	UTR3	SLC12A6	NA	NA	NA	NA	NA
chr15	34525152	34525152	T	A	UTR3	SLC12A6	NA	NA	NA	NA	NA
chr15	34525170	34525170	T	G	UTR3	SLC12A6	NA	NA	NA	NA	NA
chr15	37102239	37102239	-	AC	ncRNA_UTR3	C15orf41	NA	NA	NA	NA	NA
chr15	39889456	39889456	G	T	UTR3	THBS1	NA	NA	NA	NA	NA
chr15	39889476	39889476	G	T	UTR3	THBS1	NA	NA	NA	rs202242472	NA
chr15	40711933	40711933	G	A	UTR3	IVD	NA	NA	NA	rs4923865	NA
chr15	41024303	41024303	T	C	UTR3	RAD51	NA	NA	NA	rs11855560	NA
chr15	41099899	41099899	-	GGGGC	exonic	ZFYVE19	frameshift insertion	xon1:c.112_113insGGGGC:p.W38fs,ZFYVE19:NM_001258420:exon1:c.	NA	rs142730574	NA
chr15	41192602	41192602	G	C	exonic	VPS18	nonsynonymous SNV	VPS18:NM_020857:exon4:c.G1586C:p.S529T	NA	NA	B
chr15	41231084	41231084	A	C	UTR3	DLL4	NA	NA	NA	NA	NA
chr15	41590298	41590298	-	T	ncRNA_exonic	OIP5-AS1	NA	NA	NA	rs34536592	NA
chr15	41591315	41591318	TTTA	-	ncRNA_exonic	OIP5-AS1	NA	NA	NA	rs139720436	NA
chr15	41773443	41773444	GG	-	UTR3	RTF1	NA	NA	NA	rs10593971	NA
chr15	42159290	42159290	C	T	exonic	SPTBN5	nonsynonymous SNV	SPTBN5:NM_016642:exon36:c.G6347A:p.R2116Q	NA	rs2290553	NA
chr15	42707911	42707911	-	T	UTR3	ZNF106	NA	NA	NA	rs145603791	NA
chr15	42707938	42707941	TTTT	-	UTR3	ZNF106	NA	NA	NA	NA	NA
chr15	42834962	42834962	C	T	UTR3	LRRC57	NA	NA	NA	rs75705010	NA
chr15	42861496	42861496	T	-	UTR3	HAUS2	NA	NA	NA	NA	NA
chr15	43652542	43652542	-	A	UTR3	ZSCAN29	NA	NA	NA	rs35479272	NA
chr15	43707808	43707808	A	T	exonic	TP53BP1	synonymous SNV	:p.S1691S,TP53BP1:NM_001141980:exon23:c.T5073A:p.S1691S,TP53E	NA	rs2230451	NA
chr15	45694579	45694579	C	T	UTR5	SPATA5L1	NA	NA	NA	rs1820518	NA
chr15	45694610	45694610	C	T	UTR5	SPATA5L1	NA	NA	NA	rs1365610	NA
chr15	45848231	45848231	T	-	ncRNA_exonic	HMGN2P46	NA	NA	NA	NA	NA
chr15	50888568	50888568	A	G	exonic	TRPM7	synonymous SNV	TRPM7:NM_017672:exon23:c.T3174C:p.N1058N	NA	rs543821	NA
chr15	51697575	51697575	T	-	UTR3	GLDN	NA	NA	NA	rs5812554	NA
chr15	51914662	51914662	G	A	exonic	DMXL2	synonymous SNV	:.C81T:p.P27P,DMXL2:NM_001174117:exon1:c.C81T:p.P27P,DMXL2:N	NA	rs2278990	NA
chr15	51914716	51914716	T	C	exonic	DMXL2	synonymous SNV	:.A27G:p.G9G,DMXL2:NM_001174117:exon1:c.A27G:p.G9G,DMXL2:NI	NA	rs2278989	NA
chr15	51988178	51988178	T	A	exonic	SCG3	synonymous SNV	I_001165257:exon7:c.T279A:p.V93V,SCG3:NM_013243:exon8:c.T975A	NA	NA	NA
chr15	52485512	52485514	AAA	-	UTR3	MYO5C	NA	NA	NA	rs143753379	NA
chr15	53807252	53807252	-	TA	UTR3	WDR72	NA	NA	NA	rs141109063	NA
chr15	55495963	55495963	T	C	UTR3	RAB27A	NA	NA	NA	rs1061824	NA
chr15	55496043	55496043	T	A	UTR3	RAB27A	NA	NA	NA	rs1061823	NA
chr15	55496058	55496058	A	C	UTR3	RAB27A	NA	NA	NA	rs1061822	NA
chr15	56032872	56032872	G	A	exonic	PRTG	synonymous SNV	PRTG:NM_173814:exon2:c.C105T:p.C35C	NA	rs77118243	NA
chr15	56757188	56757188	A	C	UTR5	MNS1	NA	NA	NA	NA	NA
chr15	58476533	58476533	A	C	UTR3	AQP9	NA	NA	NA	NA	NA
chr15	59149732	59149732	C	A	UTR3	FAM63B	NA	NA	NA	NA	NA
chr15	60714879	60714879	-	A	UTR3	NARG2	NA	NA	NA	NA	NA
chr15	62146078	62146078	A	-	UTR3	VPS13C	NA	NA	NA	rs5813123	NA
chr15	62146252	62146252	-	C	UTR3	VPS13C	NA	NA	NA	NA	NA
chr15	63362505	63362505	A	G	UTR3	TPM1	NA	NA	NA	rs77963487	NA

chr15	63950887	63950887	G	A	exonic	HERC1	nonsynonymous SNV	HERC1:NM_003922:exon48:c.C9455T:p.S3152F	NA	rs2228513	NA
chr15	64433178	64433178	-	ATAT	UTR3	SNX1	NA	NA	NA	rs10678400	NA
chr15	64433233	64433236	ATAG	-	UTR3	SNX1	NA	NA	NA	rs150403479	NA
chr15	64447710	64447717	AAAAAAAA	-	UTR3	SNX22	NA	NA	NA	rs200256434	NA
chr15	64458282	64458282	A	-	UTR3	CSNK1G1	NA	NA	NA	rs143314613	NA
chr15	65674738	65674738	A	C	UTR3	IGDCC4	NA	NA	NA	rs79447611	NA
chr15	65684533	65684533	A	G	exonic	IGDCC4	synonymous SNV	IGDCC4:NM_020962:exon11:c.T2061C:p.A687A	NA	rs2277582	NA
chr15	65738042	65738042	-	A	UTR3	DPP8	NA	NA	NA	NA	NA
chr15	65738954	65738954	-	T	UTR3	DPP8	NA	NA	NA	NA	NA
chr15	66626067	66626075	AAAAAAAA	-	UTR3	DIS3L	NA	NA	NA	NA	NA
chr15	67493516	67493516	T	-	UTR3	AAGAB	NA	NA	NA	NA	NA
chr15	68124665	68124665	C	A	exonic	SKOR1	synonymous SNV	SKOR1:NM_001258024:exon12:c.C2350A:p.R784R	NA	rs72751453	NA
chr15	69113060	69113060	G	T	exonic	ANP32A	nonsynonymous SNV	ANP32A:NM_006305:exon1:c.C31A:p.L11M	NA	NA	NA
chr15	69113063	69113063	C	T	exonic	ANP32A	nonsynonymous SNV	ANP32A:NM_006305:exon1:c.G28A:p.E10K	NA	NA	NA
chr15	69113065	69113065	A	G	exonic	ANP32A	nonsynonymous SNV	ANP32A:NM_006305:exon1:c.T26C:p.L9S	NA	NA	NA
chr15	69113067	69113067	A	T	exonic	ANP32A	nonsynonymous SNV	ANP32A:NM_006305:exon1:c.T24A:p.H8Q	NA	NA	NA
chr15	69699209	69699209	A	-	UTR3	PAQR5	NA	NA	NA	NA	NA
chr15	71276491	71276491	G	A	exonic	LRRC49	nonsynonymous SNV	9A:p.R360Q,LRRC49:NM_017691:exon11:c.G1064A:p.R355Q,LRRC49:N	NA	NA	B
chr15	72452675	72452676	AA	-	UTR3	GRAMD2	NA	NA	NA	NA	NA
chr15	73614703	73614704	GT	-	UTR3	HCN4	NA	NA	NA	rs35177144	NA
chr15	74419238	74419241	TGTG	-	ncRNA_exonic	LOC283731	NA	NA	NA	NA	NA
chr15	75047699	75047699	A	C	UTR3	CYP1A2	NA	NA	NA	rs58661304	NA
chr15	75095027	75095027	T	G	UTR3	CSK	NA	NA	NA	NA	NA
chr15	75581791	75581791	G	A	exonic	GOLGA6D	synonymous SNV	GOLGA6D:NM_001145224:exon8:c.G618A:p.K206K	NA	NA	NA
chr15	76029269	76029269	G	A	ncRNA_intronic	DNM1P35	NA	NA	NA	rs199786499	NA
chr15	76552478	76552478	T	A	ncRNA_exonic	TYRO3P	NA	NA	NA	NA	NA
chr15	76552480	76552480	T	C	ncRNA_exonic	TYRO3P	NA	NA	NA	NA	NA
chr15	76552485	76552485	A	G	ncRNA_exonic	TYRO3P	NA	NA	NA	NA	NA
chr15	77228049	77228049	G	A	exonic	RCN2	nonsynonymous SNV	_001271837:exon3:c.G433A:p.E145K,RCN2:NM_002902:exon3:c.G433	NA	NA	B
chr15	77228050	77228050	A	G	exonic	RCN2	nonsynonymous SNV	_001271837:exon3:c.A434G:p.E145G,RCN2:NM_002902:exon3:c.A434	NA	NA	B
chr15	77905661	77905661	A	G	UTR3	LINGO1	NA	NA	NA	rs1058129	NA
chr15	78792881	78792883	AAA	-	UTR3	IREB2	NA	NA	NA	NA	NA
chr15	79054900	79054900	C	G	exonic	ADAMTS7	nonsynonymous SNV	ADAMTS7:NM_014272:exon23:c.G4748C:p.G1583A	NA	rs7495616	B
chr15	79237247	79237247	C	G	exonic	CTSH	nonsynonymous SNV	CTSH:NM_004390:exon1:c.G77C:p.C26S	NA	rs1036938	B
chr15	79237324	79237324	C	A	UTR5	CTSH	NA	NA	NA	rs1036939	NA
chr15	79575294	79575294	C	T	ncRNA_UTR5	ANKRD34C	NA	NA	NA	rs35531114	NA
chr15	79575486	79575486	G	A	ncRNA_UTR5	ANKRD34C	NA	NA	NA	rs62025009	NA
chr15	83013936	83013936	T	C	intergenic	297(dist=27509),UBE2Q2P3(di	NA	NA	NA	rs202104598	NA
chr15	83659296	83659296	T	-	UTR3	FAM103A1	NA	NA	NA	NA	NA
chr15	85053254	85053254	T	C	ncRNA_exonic	GOLGA6L5	NA	NA	NA	NA	NA
chr15	85055683	85055683	C	T	ncRNA_exonic	GOLGA6L5	NA	NA	NA	rs199636264	NA
chr15	85185577	85185583	AAAAAAA	-	ncRNA_exonic	SCAND2P	NA	NA	NA	rs201991585	NA
chr15	89398997	89398997	A	T	exonic	ACAN	nonsynonymous SNV	001135:exon12:c.A3181T:p.T1061S,ACAN:NM_013227:exon12:c.A318	NA	NA	NA
chr15	89399054	89399054	A	T	exonic	ACAN	nonsynonymous SNV	001135:exon12:c.A3238T:p.T1080S,ACAN:NM_013227:exon12:c.A323	NA	NA	NA
chr15	89399453	89399453	G	T	exonic	ACAN	nonsynonymous SNV	001135:exon12:c.G3637T:p.A1213S,ACAN:NM_013227:exon12:c.G363	NA	NA	NA
chr15	89400097	89400097	T	G	exonic	ACAN	nonsynonymous SNV	001135:exon12:c.T4281G:p.D1427E,ACAN:NM_013227:exon12:c.T428	NA	NA	NA
chr15	89400100	89400100	G	C	exonic	ACAN	nonsynonymous SNV	001135:exon12:c.G4284C:p.E1428D,ACAN:NM_013227:exon12:c.G428	NA	NA	NA
chr15	89400106	89400106	T	C	exonic	ACAN	synonymous SNV	001135:exon12:c.T4290C:p.S1430S,ACAN:NM_013227:exon12:c.T429	NA	rs78806382	NA
chr15	89442606	89442606	C	T	UTR3	MFGE8	NA	NA	NA	rs2280214	NA
chr15	93008848	93008848	A	C	UTR3	ST8SIA2	NA	NA	NA	NA	NA
chr15	93114612	93114612	G	A	ncRNA_exonic	LOC100144604	NA	NA	NA	NA	NA
chr15	93616283	93616283	-	AGG	UTR5	RGMA	NA	NA	NA	rs112633977	NA
chr15	98981199	98981199	A	T	UTR3	FAM169B	NA	NA	NA	NA	NA
chr15	98981248	98981248	A	G	UTR3	FAM169B	NA	NA	NA	NA	NA
chr15	1E+08	1E+08	A	C	exonic	MEF2A	nonsynonymous SNV	C:p.Q420P,MEF2A:NM_001130927:exon10:c.A1079C:p.Q360P,MEF2A:	NA	rs199811207	NA
chr15	1E+08	1E+08	T	A	ncRNA_exonic	DNM1P46	NA	NA	NA	rs200691929	NA
chr15	1E+08	1E+08	A	G	ncRNA_exonic	DNM1P46	NA	NA	NA	rs2458579	NA
chr15	1.02E+08	1.02E+08	G	A	ncRNA_exonic	FAM138E	NA	NA	NA	NA	NA
chr15	1.03E+08	1.03E+08	C	G	ncRNA_exonic	WASH3P	NA	NA	NA	rs7350147	NA
chr15	1.03E+08	1.03E+08	C	T	ncRNA_exonic	WASH3P	NA	NA	NA	rs1045862	NA
chr16	332904	332912	CCCCTGCTG	-	UTR3	ARHGDIG	NA	NA	NA	rs147027565	NA
chr16	332992	332992	T	C	UTR3	ARHGDIG	NA	NA	NA	rs443338	NA
chr16	442014	442014	G	A	ncRNA_exonic	LOC100134368	NA	NA	NA	rs57899228	NA
chr16	678121	678121	A	C	UTR3	RAB40C	NA	NA	NA	NA	NA
chr16	678133	678133	A	G	UTR3	RAB40C	NA	NA	NA	NA	NA
chr16	781633	781633	G	A	exonic	NARFL	synonymous SNV	NARFL:NM_022493:exon9:c.C966T:p.H322H	=COSM1178439;OCCURENCE=1(prostat	rs3829492	NA

chr16	1036768	1036768	G	A	UTR3	SOX8	NA	NA	NA	NA	NA
chr16	1036795	1036795	A	T	UTR3	SOX8	NA	NA	NA	NA	NA
chr16	1115843	1115846	GGCA	-	ncRNA_exonic	SSTR5-AS1	NA	NA	NA	rs143173222	NA
chr16	1116220	1116220	-	G	ncRNA_exonic	SSTR5-AS1	NA	NA	NA	rs113450650	NA
chr16	1139446	1139446	G	T	UTR3	C1QTNF8	NA	NA	NA	rs183188	NA
chr16	1290700	1290700	A	G	UTR5	TPSAB1	NA	NA	NA	rs202022651	NA
chr16	1291454	1291454	G	A	exonic	TPSAB1	nonsynonymous SNV	TPSAB1:NM_003294:exon4:c.G253A:p.A85T	NA	rs1141968	B
chr16	1292425	1292425	T	C	UTR3	TPSAB1	NA	NA	NA	rs67723611	NA
chr16	1448690	1448690	A	C	UTR3	UNKL	NA	NA	NA	NA	NA
chr16	1608082	1608082	A	G	exonic	IFT140	synonymous SNV	IFT140:NM_014714:exon19:c.T2253C:p.P751P	)=COSM148006;OCCURENCE=1(stomacl	rs2076436	NA
chr16	1843672	1843672	T	C	UTR5	IGFALS	NA	NA	NA	rs3817902	NA
chr16	1912021	1912021	T	G	exonic	MEIOB	nonsynonymous SNV	/I_001163560:exon4:c.A224C:p.K75T,MEIOB:NM_152764:exon4:c.A22	NA	rs1657125	NA
chr16	1962132	1962132	G	A	exonic	HS3ST6	nonsynonymous SNV	HS3ST6:NM_001009606:exon2:c.C395T:p.A132V	NA	rs8055325	NA
chr16	2004119	2004119	C	T	exonic	RPL3L	nonsynonymous SNV	RPL3L:NM_005061:exon2:c.G34A:p.G12R	NA	rs146294352	P
chr16	2031405	2031405	T	C	UTR5	NOXO1	NA	NA	NA	rs56268858	NA
chr16	2088407	2088417	CCCTTCCCC	-	UTR3	SLC9A3R2	NA	NA	NA	NA	NA
chr16	2391186	2391186	C	G	ncRNA_exonic	ABCA17P	NA	NA	NA	NA	NA
chr16	2508255	2508255	T	-	UTR3	CCNF	NA	NA	NA	NA	NA
chr16	2821114	2821114	T	C	UTR3	SRRM2	NA	NA	NA	NA	NA
chr16	2902840	2902840	-	G	UTR3	PRSS22	NA	NA	NA	rs145631527	NA
chr16	3078416	3078416	-	G	exonic	CCDC64B	frameshift insertion	CCDC64B:NM_001103175:exon8:c.1292_1293insC:p.L431fs	NA	NA	NA
chr16	3304654	3304654	T	C	exonic	MEFV	synonymous SNV	MEFV:NM_000243:exon2:c.A414G:p.G138G	NA	rs224224	NA
chr16	3726074	3726074	G	C	exonic	TRAP1	synonymous SNV	_001272049:exon6:c.C618G:p.S206S,TRAP1:NM_016292:exon7:c.C77	NA	rs61753377	NA
chr16	3726075	3726075	G	A	exonic	TRAP1	nonsynonymous SNV	I_001272049:exon6:c.C617T:p.S206F,TRAP1:NM_016292:exon7:c.C77	NA	rs61753378	B
chr16	4626026	4626026	G	A	exonic	C16orf96	synonymous SNV	C16orf96:NM_001145011:exon5:c.G1545A:p.K515K	NA	NA	NA
chr16	4626031	4626031	T	G	exonic	C16orf96	nonsynonymous SNV	C16orf96:NM_001145011:exon5:c.T1550G:p.V517G	NA	NA	NA
chr16	4751045	4751045	C	T	exonic	ANKS3	nonsynonymous SNV	001242929:exon8:c.G889A:p.A297T,ANKS3:NM_133450:exon11:c.G12	)=COSM148013;OCCURENCE=1(stomacl	rs863980	B
chr16	4752134	4752134	A	G	exonic	ANKS3	synonymous SNV	_001242929:exon6:c.T657C:p.D219D,ANKS3:NM_133450:exon9:c.T97	NA	rs841210	NA
chr16	4777071	4777071	A	C	exonic	ANKS3	nonsynonymous SNV	ANKS3:NM_133450:exon4:c.T278G:p.V93G	NA	NA	D
chr16	5067196	5067196	A	G	UTR3	SEC14L5	NA	NA	NA	NA	NA
chr16	5067198	5067198	T	G	UTR3	SEC14L5	NA	NA	NA	NA	NA
chr16	5068586	5068586	T	C	UTR3	SEC14L5	NA	NA	NA	rs2270257	NA
chr16	7761230	7761231	AA	-	UTR3	RBFOX1	NA	NA	NA	NA	NA
chr16	8889470	8889470	T	C	UTR3	TMEM186	NA	NA	NA	NA	NA
chr16	9211118	9211118	T	-	UTR3	C16orf72	NA	NA	NA	rs34120073	NA
chr16	9916204	9916204	C	G	exonic	GRIN2A	synonymous SNV	C:p.R695R,GRIN2A:NM_001134408:exon10:c.G2085C:p.R695R,GRIN2	NA	rs9806806	NA
chr16	10524657	10524659	GAC	-	exonic	ATF7IP2	nonframeshift deletion	:exon3:c.180_182del:p.60_61del,ATF7IP2:NM_001256160:exon4:c.18	NA	rs56252625	NA
chr16	10565979	10565981	TGT	-	exonic	ATF7IP2	nonframeshift deletion	n8:c.1365_1367del:p.455_456del,ATF7IP2:NM_001256160:exon9:c.13	NA	NA	NA
chr16	10625175	10625175	G	A	UTR3	EMP2	NA	NA	NA	rs10459782	NA
chr16	10625606	10625613	ATGAATGA	-	UTR3	EMP2	NA	NA	NA	rs149380057	NA
chr16	10626608	10626608	C	T	UTR3	EMP2	NA	NA	NA	rs12930183	NA
chr16	10626615	10626615	C	T	UTR3	EMP2	NA	NA	NA	rs12930185	NA
chr16	12897610	12897610	A	C	exonic	CPPED1	synonymous SNV	NM_001099455:exon1:c.T24G:p.G8G,CPPED1:NM_018340:exon1:c.T2	NA	NA	NA
chr16	13333322	13333322	T	A	UTR3	SHISA9	NA	NA	NA	rs202142648	NA
chr16	14529754	14529755	CA	-	UTR3	PARN	NA	NA	NA	NA	NA
chr16	15166891	15166891	T	C	exonic	RRN3	nonsynonymous SNV	RRN3:NM_018427:exon12:c.A1044G:p.I348M	NA	rs2941256	B
chr16	16367678	16367678	T	C	exonic	NOMO3	synonymous SNV	NOMO3:NM_001004067:exon19:c.T2187C:p.G729G	NA	rs11554237	NA
chr16	18839362	18839362	T	C	exonic	SMG1	synonymous SNV	SMG1:NM_015092:exon55:c.A9732G:p.A3244A	NA	rs12445870	NA
chr16	19278429	19278429	-	A	UTR3	SYT17	NA	NA	NA	rs11379176	NA
chr16	19563849	19563850	AA	-	UTR3	CCP110	NA	NA	NA	rs148157558	NA
chr16	20746239	20746239	G	C	UTR3	THUMPD1	NA	NA	NA	rs67088894	NA
chr16	20748331	20748331	C	A	exonic	THUMPD1	nonsynonymous SNV	THUMPD1:NM_017736:exon4:c.G933T:p.E311D	NA	rs11074471	NA
chr16	21078693	21078693	T	G	exonic	DNAH3	synonymous SNV	DNAH3:NM_017539:exon24:c.A3429C:p.A1143A	NA	rs861424	NA
chr16	21191548	21191548	C	T	UTR3	TMEM159	NA	NA	NA	NA	NA
chr16	21329340	21329351	CACACACA	-	ncRNA_exonic	CRYM-AS1	NA	NA	NA	rs147884336	NA
chr16	21513228	21513228	G	A	ncRNA_exonic	LOC100271836	NA	NA	NA	rs62047609	NA
chr16	22299310	22299313	TTTT	-	UTR3	EEF2K	NA	NA	NA	NA	NA
chr16	22546421	22546421	A	G	exonic	LOC100132247	nonsynonymous SNV	LOC100132247:NM_001135865:exon7:c.A2117G:p.Q706R	NA	NA	NA
chr16	23598584	23598584	C	T	exonic	NDUFAB1	synonymous SNV	NDUFAB1:NM_005003:exon2:c.G225A:p.T75T	NA	rs139679834	NA
chr16	24230566	24230566	-	TTTTG	UTR3	PRKCB	NA	NA	NA	NA	NA
chr16	24230976	24230976	A	-	UTR3	PRKCB	NA	NA	NA	rs200055220	NA
chr16	24230979	24230983	ACACA	-	UTR3	PRKCB	NA	NA	NA	NA	NA
chr16	24836091	24836091	A	-	UTR3	TNRC6A	NA	NA	NA	NA	NA
chr16	25250586	25250587	AA	-	UTR3	ZKSCAN2	NA	NA	NA	NA	NA
chr16	28332504	28332521	CACACACA	-	UTR3	SBK1	NA	NA	NA	rs67770198	NA
chr16	28875215	28875223	GCCGCCGCC	-	UTR5	SH2B1	NA	NA	NA	rs138146565	NA

chr16	28877938	28877938	A	G	exonic	SH2B1	nonsynonymous SNV	:p.T175A,SH2B1:NM_001145796:exon2:c.A523G:p.T175A,SH2B1:NM_	NA	rs181294111	B
chr16	28877949	28877949	T	C	exonic	SH2B1	synonymous SNV	:p.P178P,SH2B1:NM_001145796:exon2:c.T534C:p.P178P,SH2B1:NM_	NA	NA	NA
chr16	28877991	28877991	A	C	exonic	SH2B1	synonymous SNV	:p.P192P,SH2B1:NM_001145796:exon2:c.A576C:p.P192P,SH2B1:NM_	NA	NA	NA
chr16	29562570	29562570	C	A	ncRNA_exonic	LOC440354	NA	NA	NA	rs200496369	NA
chr16	29576811	29576811	A	G	ncRNA_exonic	LOC440354	NA	NA	NA	rs201149124	NA
chr16	29679738	29679741	TGAA	-	UTR3	SPN	NA	NA	NA	rs10537821	NA
chr16	29681197	29681197	-	A	UTR3	SPN	NA	NA	NA	rs112370778	NA
chr16	29706379	29706379	C	T	exonic	QPRT	synonymous SNV	QPRT:NM_014298:exon2:c.C408T:p.G136G	NA	rs113148463	NA
chr16	29819087	29819087	C	G	exonic	MAZ	synonymous SNV	981G:p.R327R,MAZ:NM_002383:exon2:c.C981G:p.R327R,MAZ:NM_00	NA	NA	NA
chr16	29827029	29827029	C	G	UTR3	PRRT2	NA	NA	NA	NA	NA
chr16	30581031	30581031	C	A	UTR3	ZNF688	NA	NA	NA	NA	NA
chr16	30581051	30581051	T	G	UTR3	ZNF688	NA	NA	NA	NA	NA
chr16	30592781	30592781	A	-	UTR3	ZNF785	NA	NA	NA	NA	NA
chr16	30593451	30593451	G	T	UTR3	ZNF785	NA	NA	NA	NA	NA
chr16	31003963	31003963	G	-	UTR3	STX1B	NA	NA	NA	rs67009097	NA
chr16	31092888	31092888	A	G	exonic	ZNF646	nonsynonymous SNV	ZNF646:NM_014699:exon2:c.A5243G:p.E1748G	NA	NA	P
chr16	31092899	31092899	C	G	exonic	ZNF646	nonsynonymous SNV	ZNF646:NM_014699:exon2:c.C5254G:p.R1752G	NA	NA	D
chr16	31092933	31092933	G	C	exonic	ZNF646	nonsynonymous SNV	ZNF646:NM_014699:exon2:c.G5288C:p.C1763S	NA	NA	D
chr16	46602819	46602819	C	T	ncRNA_exonic	ANKRD26P1	NA	NA	NA	rs12934443	NA
chr16	46693621	46693621	-	A	UTR3	VPS35	NA	NA	NA	rs34926621	NA
chr16	46694101	46694101	G	T	UTR3	VPS35	NA	NA	NA	rs199765664	NA
chr16	46694103	46694103	G	T	UTR3	VPS35	NA	NA	NA	rs808078	NA
chr16	46773999	46773999	C	A	exonic	MYLK3	nonsynonymous SNV	MYLK3:NM_182493:exon2:c.G538T:p.V180L	M148070,COSM148071;OCCURENCE=1(	rs28407821	D
chr16	46836143	46836143	T	-	UTR3	C16orf87	NA	NA	NA	NA	NA
chr16	47732497	47732497	C	A	exonic	PHKB	nonsynonymous SNV	0293:exon30:c.C3142A:p.Q1048K,PHKB:NM_001031835:exon31:c.C31	NA	NA	P
chr16	48258198	48258198	C	T	exonic	ABCC11	nonsynonymous SNV	8A:p.G180R,ABCC11:NM_032583:exon5:c.G538A:p.G180R,ABCC11:NM	NA	rs17822931	D
chr16	50264211	50264211	-	A	UTR3	PAPD5	NA	NA	NA	rs34413257	NA
chr16	53504029	53504029	C	T	exonic	RBL2	nonsynonymous SNV	RBL2:NM_005611:exon15:c.C2177T:p.T726I	NA	NA	D
chr16	55794514	55794514	C	T	ncRNA_exonic	CES1P1	NA	NA	NA	rs7199449	NA
chr16	55862691	55862691	G	A	exonic	CES1	nonsynonymous SNV	:.C245T:p.S82L,CES1:NM_001025195:exon2:c.C248T:p.S83L,CES1:NM_	NA	rs62028647	D
chr16	55862717	55862717	T	C	exonic	CES1	synonymous SNV	A219G:p.P73P,CES1:NM_001025195:exon2:c.A222G:p.P74P,CES1:NM_	NA	rs76828834	NA
chr16	55862720	55862720	T	C	exonic	CES1	synonymous SNV	A216G:p.E72E,CES1:NM_001025195:exon2:c.A219G:p.E73E,CES1:NM_	NA	rs74019278	NA
chr16	55862762	55862762	C	G	exonic	CES1	synonymous SNV	G174C:p.P58P,CES1:NM_001025195:exon2:c.G177C:p.P59P,CES1:NM_	NA	rs35384853	NA
chr16	56509445	56509445	A	T	exonic	OGFOD1	nonsynonymous SNV	OGFOD1:NM_018233:exon12:c.A1435T:p.T479S	NA	NA	D
chr16	56509452	56509452	A	T	exonic	OGFOD1	nonsynonymous SNV	OGFOD1:NM_018233:exon12:c.A1442T:p.Y481F	NA	NA	D
chr16	56510842	56510842	G	C	UTR3	OGFOD1	NA	NA	NA	rs111625702	NA
chr16	56691866	56691866	T	C	UTR5	MT1F	NA	NA	NA	NA	NA
chr16	56966071	56966071	T	G	UTR5	HERPUD1	NA	NA	NA	rs37024	NA
chr16	57398718	57398718	-	T	UTR3	CCL22	NA	NA	NA	rs34460030	NA
chr16	58054465	58054465	-	CTCT	UTR3	USB1	NA	NA	NA	rs112874007	NA
chr16	58315899	58315899	A	C	UTR3	CCDC113	NA	NA	NA	NA	NA
chr16	58546850	58546850	C	T	UTR3	NDRG4	NA	NA	NA	NA	NA
chr16	58553551	58553551	A	-	UTR3	SETD6	NA	NA	NA	NA	NA
chr16	67200249	67200249	T	G	exonic	HSF4	nonsynonymous SNV	_001040667:exon7:c.T512G:p.V171G,HSF4:NM_001538:exon7:c.T512	NA	rs201403716	D
chr16	67200252	67200252	T	G	exonic	HSF4	nonsynonymous SNV	_001040667:exon7:c.T515G:p.V172G,HSF4:NM_001538:exon7:c.T515	NA	NA	D
chr16	68599864	68599864	-	A	UTR3	ZFP90	NA	NA	NA	NA	NA
chr16	68849519	68849519	C	A	exonic	CDH1	synonymous SNV	CDH1:NM_004360:exon10:c.C1422A:p.T474T	NA	NA	NA
chr16	69341282	69341282	T	-	UTR3	SNTB2	NA	NA	NA	rs71752949	NA
chr16	69341695	69341695	A	-	UTR3	SNTB2	NA	NA	NA	rs200699081	NA
chr16	69385641	69385641	A	G	exonic	TMED6	nonsynonymous SNV	TMED6:NM_144676:exon1:c.T16C:p.F6L	NA	rs76116020	B
chr16	69599088	69599088	-	AAAAAAAA	upstream;downstream	NFAT5;MIR1538	NA	NA	NA	NA	NA
chr16	70164334	70164334	A	G	exonic	PDPR	nonsynonymous SNV	PDPR:NM_017990:exon7:c.A616G:p.I206V	)=COSM148098;OCCURENCE=1(stomac	rs10852462	NA
chr16	70182375	70182375	T	C	exonic	PDPR	synonymous SNV	PDPR:NM_017990:exon17:c.T1971C:p.S657S	NA	rs11548413	NA
chr16	70182390	70182390	T	G	exonic	PDPR	nonsynonymous SNV	PDPR:NM_017990:exon17:c.T1986G:p.N662K	NA	rs62050978	NA
chr16	70506907	70506907	T	C	exonic	FUK	synonymous SNV	FUK:NM_145059:exon15:c.T1428C:p.P476P	=COSM1128894;OCCURENCE=1(prostat	rs7192865	NA
chr16	70569215	70569215	A	T	exonic	SF3B3	synonymous SNV	SF3B3:NM_012426:exon6:c.A717T:p.P239P	NA	rs33910368	NA
chr16	70609852	70609852	T	C	UTR3	SF3B3	NA	NA	NA	rs2168606	NA
chr16	70609997	70609997	A	T	UTR3	SF3B3	NA	NA	NA	rs79714557	NA
chr16	70721428	70721429	AC	-	UTR3	VAC14	NA	NA	NA	rs3833084	NA
chr16	71981414	71981414	-	TTTG	exonic	PKD1L3	unknown	UNKNOWN	NA	rs149635567	NA
chr16	72820141	72820141	T	-	UTR3	ZFHX3	NA	NA	NA	NA	NA
chr16	72820315	72820315	T	-	UTR3	ZFHX3	NA	NA	NA	rs199690302	NA
chr16	72820938	72820938	-	T	UTR3	ZFHX3	NA	NA	NA	NA	NA
chr16	74452071	74452071	G	A	exonic	CLEC18B	synonymous SNV	CLEC18B:NM_001011880:exon3:c.C342T:p.P114P	NA	rs62056016	NA
chr16	74655597	74655597	-	T	UTR3	RFWD3	NA	NA	NA	NA	NA
chr16	74907485	74907485	T	-	UTR3	WDR59	NA	NA	NA	rs34150235	NA

chr16	74920191	74920191	G	A	exonic	WDR59	synonymous SNV	WDR59:NM_030581:exon24:c.C2523T:p.R841R	NA	rs14308	NA
chr16	75327916	75327916	A	G	exonic	CFDP1	synonymous SNV	CFDP1:NM_006324:exon7:c.T834C:p.L278L	NA	rs6624	NA
chr16	75565219	75565219	A	-	UTR5	CHST5	NA	NA	NA	NA	NA
chr16	77228764	77228764	C	T	exonic	MON1B	synonymous SNV	MON1B:NM_014940:exon4:c.C1008T:p.F336F	NA	NA	NA
chr16	77316803	77316803	C	T	UTR3	ADAMTS18	NA	NA	NA	rs28385461	NA
chr16	77316803	77316803	-	TCTT	UTR3	ADAMTS18	NA	NA	NA	NA	NA
chr16	81253759	81253759	A	G	exonic	PKD1L2	nonsynonymous SNV	PKD1L2:NM_001076780:exon1:c.T217C:p.W73R	NA	rs9924371	B
chr16	81253917	81253917	A	G	exonic	PKD1L2	nonsynonymous SNV	PKD1L2:NM_001076780:exon1:c.T59C:p.V20A	NA	rs9924530	B
chr16	82203742	82203742	T	C	exonic	MPHOSPH6	synonymous SNV	MPHOSPH6:NM_005792:exon1:c.A39G:p.L13L	NA	rs1134847	NA
chr16	82203758	82203758	C	T	exonic	MPHOSPH6	nonsynonymous SNV	MPHOSPH6:NM_005792:exon1:c.G23A:p.R8K	NA	rs2303262	B
chr16	82203768	82203768	G	T	exonic	MPHOSPH6	synonymous SNV	MPHOSPH6:NM_005792:exon1:c.C13A:p.R5R	NA	rs2303261	NA
chr16	82203786	82203786	C	G	UTR5	MPHOSPH6	NA	NA	NA	rs2303260	NA
chr16	84203833	84203833	A	C	exonic	DNAAF1	nonsynonymous SNV	DNAAF1:NM_178452:exon8:c.A1399C:p.T467P	NA	NA	P
chr16	84203862	84203862	A	G	exonic	DNAAF1	synonymous SNV	DNAAF1:NM_178452:exon8:c.A1428G:p.S476S	NA	NA	NA
chr16	84522897	84522897	G	C	exonic	TLDC1	nonsynonymous SNV	TLDC1:NM_020947:exon4:c.C516G:p.D172E	=COSM1128850;OCCURENCE=1(prostat	rs436278	B
chr16	85125545	85125546	TT	-	UTR3	KIAA0513	NA	NA	NA	NA	NA
chr16	85707085	85707085	A	-	UTR3	GSE1	NA	NA	NA	NA	NA
chr16	87493737	87493737	T	C	exonic	ZCCHC14	nonsynonymous SNV	ZCCHC14:NM_015144:exon2:c.A160G:p.I54V	NA	rs11648852	NA
chr16	87723838	87723838	T	C	exonic	JPH3	synonymous SNV	JPH3:NM_020655:exon4:c.T1872C:p.H624H	NA	rs34767155	NA
chr16	88497394	88497394	T	C	exonic	ZNF469	synonymous SNV	ZNF469:NM_001127464:exon2:c.T3432C:p.R1144R	NA	rs111557381	NA
chr16	88947735	88947735	G	A	exonic	CBFA2T3	nonsynonymous SNV	1_175931:exon8:c.C1108T:p.R370C,CBFA2T3:NM_005187:exon9:c.C13	NA	rs202023439	P
chr16	89259993	89259993	A	G	exonic	CDH15	synonymous SNV	CDH15:NM_004933:exon12:c.A1971G:p.Q657Q	NA	rs3815615	NA
chr16	89777761	89777761	C	T	exonic	VPS9D1	synonymous SNV	VPS9D1:NM_004913:exon9:c.G807A:p.V269V	NA	rs12920315	NA
chr16	90061629	90061629	T	C	ncRNA_exonic	AFG3L1P	NA	NA	NA	NA	NA
chr16	90095573	90095573	C	T	exonic	C16orf3	nonsynonymous SNV	C16orf3:NM_001214:exon1:c.G178A:p.V60I	NA	rs77382359	NA
chr16	90095582	90095582	T	C	exonic	C16orf3	nonsynonymous SNV	C16orf3:NM_001214:exon1:c.A169G:p.S57G	NA	rs62640380	NA
chr16	90095596	90095596	A	G	exonic	C16orf3	nonsynonymous SNV	C16orf3:NM_001214:exon1:c.T155C:p.I52T	NA	rs55742939	NA
chr16	90095597	90095597	T	C	exonic	C16orf3	nonsynonymous SNV	C16orf3:NM_001214:exon1:c.A154G:p.I52V	NA	rs61118444	NA
chr16	90095620	90095620	A	G	exonic	C16orf3	nonsynonymous SNV	C16orf3:NM_001214:exon1:c.T131C:p.V44A	NA	rs61740023	NA
chr17	263367	263367	A	T	exonic	C17orf97	nonsynonymous SNV	C17orf97:NM_001013672:exon2:c.A733T:p.I245F	NA	rs35400518	B
chr17	263387	263387	T	C	exonic	C17orf97	synonymous SNV	C17orf97:NM_001013672:exon2:c.T753C:p.A251A	NA	rs34825436	NA
chr17	292322	292324	AAA	-	UTR3	FAM101B	NA	NA	NA	NA	NA
chr17	1326755	1326755	A	-	UTR3	CRK	NA	NA	NA	NA	NA
chr17	1373612	1373612	C	T	exonic	MYO1C	nonsynonymous SNV	3A:p.V795I,MYO1C:NM_001080950:exon24:c.G2326A:p.V776I,MYO1C	NA	rs8081370	NA
chr17	1606883	1606883	T	G	UTR3	TLCD2	NA	NA	NA	rs8068949	NA
chr17	1607517	1607517	-	A	UTR3	TLCD2	NA	NA	NA	NA	NA
chr17	1609930	1609930	G	A	UTR3	TLCD2	NA	NA	NA	rs4790808	NA
chr17	1960044	1960044	C	A	exonic	HIC1	nonsynonymous SNV	M_001098202:exon2:c.C117A:p.N39K,HIC1:NM_006497:exon2:c.C60A	NA	NA	B
chr17	2287891	2287891	-	GTGTGT	UTR3	MNT	NA	NA	NA	NA	NA
chr17	2310332	2310332	G	A	ncRNA_exonic	LOC284009	NA	NA	NA	rs74415860	NA
chr17	2883588	2883588	C	A	exonic	RAP1GAP2	nonsynonymous SNV	_001100398:exon8:c.C559A:p.L187M,RAP1GAP2:NM_015085:exon9:c.	NA	rs17762452	NA
chr17	2929392	2929392	G	A	exonic	RAP1GAP2	synonymous SNV	01100398:exon19:c.G1797A:p.P599P,RAP1GAP2:NM_015085:exon20:	=COSM148146;OCCURENCE=1(stomac	rs55904912	NA
chr17	2940848	2940851	AGTG	-	UTR3	RAP1GAP2	NA	NA	NA	NA	NA
chr17	3343406	3343406	G	A	UTR3	SPATA22	NA	NA	NA	rs2257732	NA
chr17	3352294	3352294	A	G	exonic	SPATA22	nonsynonymous SNV	M_001170697:exon6:c.T479C:p.I160T,SPATA22:NM_001170698:exon6:	NA	rs1488689	B
chr17	3352331	3352331	C	T	exonic	SPATA22	nonsynonymous SNV	001170697:exon6:c.G442A:p.V148M,SPATA22:NM_001170698:exon6:	NA	rs1488690	B
chr17	3445901	3445901	T	G	exonic	TRPV3	synonymous SNV	M_001258205:exon6:c.A558C:p.I186I,TRPV3:NM_145068:exon6:c.A55	NA	rs11078458	NA
chr17	3627473	3627473	C	T	exonic	GSG2	nonsynonymous SNV	GSG2:NM_031965:exon1:c.C244T:p.R82C	NA	rs9907144	D
chr17	4066687	4066687	G	-	UTR3	ANKFY1	NA	NA	NA	rs11307002	NA
chr17	5037195	5037195	G	A	exonic	USP6	nonsynonymous SNV	USP6:NM_004505:exon7:c.G398A:p.R133K	NA	rs74900103	NA
chr17	5288983	5288983	T	C	UTR3	RABEP1	NA	NA	NA	rs7426	NA
chr17	6328628	6328628	T	-	UTR3	AIPL1	NA	NA	NA	NA	NA
chr17	6350949	6350949	A	C	exonic	FAM64A	nonsynonymous SNV	_001195228:exon3:c.A461C:p.D154A,FAM64A:NM_019013:exon3:c.A4	NA	NA	B
chr17	6355187	6355187	A	C	UTR3	PITPNM3	NA	NA	NA	NA	NA
chr17	6355210	6355210	G	C	UTR3	PITPNM3	NA	NA	NA	NA	NA
chr17	6556194	6556194	-	AGAAG	UTR3	C17orf100	NA	NA	NA	rs144808411	NA
chr17	6690858	6690858	-	T	UTR3	FBXO39	NA	NA	NA	NA	NA
chr17	6983433	6983433	C	T	UTR5	CLEC10A	NA	NA	NA	rs148814818	NA
chr17	7093397	7093397	A	-	UTR3	DLG4	NA	NA	NA	rs5819149	NA
chr17	7097648	7097648	T	G	exonic	DLG4	nonsynonymous SNV	01128827:exon12:c.A1459C:p.S487R,DLG4:NM_001365:exon14:c.A15	NA	NA	NA
chr17	7215397	7215397	A	G	UTR3	EIF5A	NA	NA	NA	NA	NA
chr17	7475159	7475159	A	T	ncRNA_UTR3	SENP3	NA	NA	NA	rs1133249	NA
chr17	7482019	7482019	A	-	ncRNA_exonic	SENP3-EIF4A1	NA	NA	NA	NA	NA
chr17	7658285	7658285	A	-	ncRNA_exonic	RPL29P2	NA	NA	NA	NA	NA
chr17	7750178	7750183	ACCACC	-	exonic	KDM6B	nonframeshift deletion	KDM6B:NM_001080424:exon9:c.753_758del:p.251_253del	NA	NA	NA
chr17	7751859	7751864	CACCAC	-	exonic	KDM6B	nonframeshift deletion	KDM6B:NM_001080424:exon11:c.2253_2258del:p.751_753del	NA	NA	NA

chr17	7757725	7757725	-	A	UTR3	KDM6B	NA	NA	NA	rs112843870	NA
chr17	7942901	7942901	C	G	exonic	ALOX15B	synonymous SNV	5G:p.T115T,ALOX15B:NM_001039131:exon2:c.C345G:p.T115T,ALOX15	NA	rs76589243	NA
chr17	8064081	8064081	-	G	UTR3	VAMP2	NA	NA	NA	NA	NA
chr17	8173199	8173199	T	-	UTR3	PFAS	NA	NA	NA	NA	NA
chr17	8173767	8173767	C	T	UTR3	PFAS	NA	NA	NA	rs1132554	NA
chr17	8215408	8215408	T	C	exonic	ARHGEF15	synonymous SNV	:NM_025014:exon2:c.T51C:p.P17P,ARHGEF15:NM_173728:exon2:c.T5	NA	NA	NA
chr17	8215425	8215425	G	C	exonic	ARHGEF15	nonsynonymous SNV	:NM_025014:exon2:c.G68C:p.R23P,ARHGEF15:NM_173728:exon2:c.G	NA	NA	P
chr17	8633310	8633310	C	T	UTR3	CCDC42	NA	NA	NA	rs1971773	NA
chr17	10618236	10618236	-	AA	UTR3	TMEM220	NA	NA	NA	NA	NA
chr17	12668632	12668632	A	-	UTR3	MYOCD	NA	NA	NA	rs200654104	NA
chr17	15531723	15531723	A	-	UTR3	TRIM16	NA	NA	NA	NA	NA
chr17	15622453	15622453	A	-	UTR3	ZNF286A	NA	NA	NA	NA	NA
chr17	16285320	16285320	G	A	exonic	UBB	synonymous SNV	UBB:NM_018955:exon2:c.G99A:p.K33K	NA	NA	NA
chr17	16842912	16842912	A	G	exonic	TNFRSF13B	synonymous SNV	TNFRSF13B:NM_012452:exon5:c.T831C:p.S277S	NA	rs11078355	NA
chr17	16979078	16979078	G	T	exonic	MPRIP	nonsynonymous SNV	NM_015134:exon2:c.G178T:p.D60Y,MPRIP:NM_201274:exon2:c.G178T	NA	NA	NA
chr17	17115873	17115873	T	G	UTR3	FLCN	NA	NA	NA	NA	NA
chr17	18024013	18024013	A	G	exonic	MYO15A	synonymous SNV	MYO15A:NM_016239:exon2:c.A1899G:p.P633P	NA	rs2955366	NA
chr17	18327352	18327352	C	T	ncRNA_exonic	LOC339240	NA	NA	NA	rs3869522	NA
chr17	18573286	18573288	TTG	-	ncRNA_exonic	FOXO3B	NA	NA	NA	rs200452589	NA
chr17	18682505	18682505	T	C	exonic	FBXW10	nonsynonymous SNV	267586:exon13:c.T2894C:p.V965A,FBXW10:NM_001267585:exon14:c.	=COSM436168;OCCURENCE=1(prostat	rs1024657	B
chr17	18682632	18682632	A	G	UTR3	FBXW10	NA	NA	NA	rs2014354	NA
chr17	18694277	18694277	G	A	exonic	TVP23B	nonsynonymous SNV	TVP23B:NM_016078:exon3:c.G164A:p.G55E	NA	rs61075345	B
chr17	20163529	20163529	A	G	exonic	SPECC1	synonymous SNV	p.A873A,SPECC1:NM_001033553:exon12:c.A2862G:p.A954A,SPECC1:	NA	rs2703791	NA
chr17	20768134	20768134	C	T	UTR3	CCDC144NL	NA	NA	NA	rs201667905	NA
chr17	20768143	20768143	G	A	UTR3	CCDC144NL	NA	NA	NA	NA	NA
chr17	20768314	20768314	A	G	UTR3	CCDC144NL	NA	NA	NA	rs77283162	NA
chr17	20768333	20768333	G	A	UTR3	CCDC144NL	NA	NA	NA	rs113358321	NA
chr17	20768342	20768342	A	C	UTR3	CCDC144NL	NA	NA	NA	rs75123357	NA
chr17	20768390	20768390	C	A	UTR3	CCDC144NL	NA	NA	NA	rs146578750	NA
chr17	20768608	20768608	G	A	UTR3	CCDC144NL	NA	NA	NA	rs4347684	NA
chr17	21117837	21117837	T	C	UTR5	TMEM11	NA	NA	NA	rs4985988	NA
chr17	21203964	21203964	C	G	exonic	MAP2K3	synonymous SNV	IM_002756:exon4:c.C186G:p.A62A,MAP2K3:NM_145109:exon4:c.C27	;OCCURENCE=1(haematopoietic_and_l	rs34458870	NA
chr17	21204290	21204290	G	C	exonic	MAP2K3	synonymous SNV	M_002756:exon5:c.G297C:p.G99G,MAP2K3:NM_145109:exon5:c.G38	;OCCURENCE=2(haematopoietic_and_l	rs2305872	NA
chr17	21207834	21207834	C	T	exonic	MAP2K3	nonsynonymous SNV	M_002756:exon8:c.C578T:p.T193M,MAP2K3:NM_145109:exon8:c.C66	NA	rs58609466	D
chr17	21217727	21217727	T	C	UTR3	MAP2K3	NA	NA	NA	rs2363370	NA
chr17	21217764	21217764	G	A	UTR3	MAP2K3	NA	NA	NA	rs2363192	NA
chr17	21311916	21311916	C	T	UTR5	KCNJ12,KCNJ18	NA	NA	NA	rs1657716	NA
chr17	21311940	21311940	T	C	UTR5	KCNJ12,KCNJ18	NA	NA	NA	rs1714903	NA
chr17	21319407	21319407	C	T	exonic	KCNJ12,KCNJ18	synonymous SNV	M_001194958:exon3:c.C753T:p.I251I,KCNJ12:NM_021012:exon3:c.C75	NA	rs16962951	NA
chr17	21319436	21319436	G	A	exonic	KCNJ12,KCNJ18	nonsynonymous SNV	_001194958:exon3:c.G782A:p.R261H,KCNJ12:NM_021012:exon3:c.G7	ID=COSM312197;OCCURENCE=1(lung)	rs77270326	P
chr17	21319439	21319439	T	G	exonic	KCNJ12,KCNJ18	nonsynonymous SNV	M_001194958:exon3:c.T785G:p.I262S,KCNJ12:NM_021012:exon3:c.T7	ID=COSM312198;OCCURENCE=1(lung)	rs76684759	P
chr17	21319452	21319452	G	A	exonic	KCNJ12,KCNJ18	synonymous SNV	_001194958:exon3:c.G798A:p.S266S,KCNJ12:NM_021012:exon3:c.G7	NA	rs73313929	NA
chr17	21319465	21319465	T	C	exonic	KCNJ12,KCNJ18	synonymous SNV	1_001194958:exon3:c.T811C:p.L271L,KCNJ12:NM_021012:exon3:c.T81	ID=COSM312199;OCCURENCE=1(lung)	rs77724065	NA
chr17	21322607	21322607	-	GT	UTR3	KCNJ12	NA	NA	NA	NA	NA
chr17	21323033	21323033	C	T	UTR3	KCNJ12	NA	NA	NA	rs72842126	NA
chr17	21323034	21323034	G	A	UTR3	KCNJ12	NA	NA	NA	rs72842128	NA
chr17	21323049	21323049	C	T	UTR3	KCNJ12	NA	NA	NA	rs75724776	NA
chr17	25640280	25640280	-	T	UTR3	WSB1	NA	NA	NA	NA	NA
chr17	26206414	26206414	C	A	UTR3	LYRM9	NA	NA	NA	rs3751972	NA
chr17	26522009	26522009	-	CA	UTR3	NLK	NA	NA	NA	NA	NA
chr17	26653991	26653991	T	-	UTR3	TMEM97	NA	NA	NA	rs71748594	NA
chr17	27187789	27187789	C	T	UTR3	ERAL1	NA	NA	NA	rs6803	NA
chr17	28523383	28523383	T	-	UTR3	SLC6A4	NA	NA	NA	NA	NA
chr17	29302644	29302644	C	A	ncRNA_exonic	DPRXP4	NA	NA	NA	NA	NA
chr17	29644387	29644388	AC	-	UTR3	EVI2A	NA	NA	NA	rs111459872	NA
chr17	29862282	29862282	A	-	UTR3	RAB11FIP4	NA	NA	NA	rs35637322	NA
chr17	33307586	33307586	G	A	UTR5	LIG3	NA	NA	NA	rs12945428	NA
chr17	33448818	33448818	T	C	exonic	FNDC8	nonsynonymous SNV	FNDC8:NM_017559:exon1:c.T106C:p.S36P	NA	rs1871892	B
chr17	33881718	33881718	G	A	exonic	SLFN14	nonsynonymous SNV	SLFN14:NM_001129820:exon2:c.C1066T:p.P356S	NA	rs321613	NA
chr17	34151149	34151149	T	G	exonic	TAF15	synonymous SNV	M_003487:exon7:c.T543G:p.G181G,TAF15:NM_139215:exon7:c.T552G	NA	NA	NA
chr17	34151164	34151164	A	G	exonic	TAF15	synonymous SNV	M_003487:exon7:c.A558G:p.G186G,TAF15:NM_139215:exon7:c.A567G	NA	NA	NA
chr17	34195864	34195864	G	T	UTR5	C17orf66	NA	NA	NA	NA	NA
chr17	36715976	36715976	T	G	exonic	SRCIN1	nonsynonymous SNV	SRCIN1:NM_025248:exon10:c.A1870C:p.T624P	NA	NA	NA
chr17	36896661	36896661	C	T	UTR5	PCGF2	NA	NA	NA	rs182900980	NA
chr17	37366899	37366899	-	G	UTR3	STAC2	NA	NA	NA	rs35966204	NA
chr17	37367542	37367542	-	T	UTR3	STAC2	NA	NA	NA	NA	NA

chr17	37562812	37562812	-	T	UTR3	MED1	NA	NA	NA	rs11443056	NA
chr17	37761057	37761057	G	T	UTR3	NEUROD2	NA	NA	NA	NA	NA
chr17	37761061	37761061	G	T	UTR3	NEUROD2	NA	NA	NA	NA	NA
chr17	38028634	38028634	G	T	exonic	ZPBP2	nonsynonymous SNV	IM_198844:exon4:c.G452T:p.S151I,ZPBP2:NM_199321:exon5:c.G518T	NA	rs11557467	P
chr17	38133914	38133914	-	A	UTR3	GSDMA	NA	NA	NA	NA	NA
chr17	38175428	38175428	-	ACACAC	UTR3	MED24	NA	NA	NA	NA	NA
chr17	38510712	38510712	T	A	exonic	RARA	nonsynonymous SNV	_001024809:exon6:c.T951A:p.D317E,RARA:NM_000964:exon7:c.T966	NA	NA	D
chr17	38510716	38510716	G	C	exonic	RARA	nonsynonymous SNV	_001024809:exon6:c.G955C:p.A319P,RARA:NM_000964:exon7:c.G970	NA	NA	P
chr17	38510717	38510717	C	G	exonic	RARA	nonsynonymous SNV	_001024809:exon6:c.C956G:p.A319G,RARA:NM_000964:exon7:c.C971	NA	NA	P
chr17	38510719	38510719	G	A	exonic	RARA	nonsynonymous SNV	_001024809:exon6:c.G958A:p.E320K,RARA:NM_000964:exon7:c.G973	NA	NA	D
chr17	38974612	38974612	T	C	UTR3	KRT10	NA	NA	NA	rs1132367	NA
chr17	39211189	39211189	C	G	exonic	KRTAP2-2	nonsynonymous SNV	KRTAP2-2:NM_033032:exon1:c.G275C:p.C92S	NA	NA	NA
chr17	39240641	39240641	A	G	exonic	KRTAP4-7	synonymous SNV	KRTAP4-7:NM_033061:exon1:c.A183G:p.Q61Q	NA	NA	NA
chr17	39240653	39240653	T	C	exonic	KRTAP4-7	synonymous SNV	KRTAP4-7:NM_033061:exon1:c.T195C:p.C65C	NA	NA	NA
chr17	39395019	39395019	G	T	UTR3	KRTAP9-8	NA	NA	NA	rs74975222	NA
chr17	39727816	39727816	T	G	exonic	KRT9	synonymous SNV	KRT9:NM_000226:exon1:c.A429C:p.G143G	NA	rs8075921	NA
chr17	39728050	39728050	G	A	exonic	KRT9	synonymous SNV	KRT9:NM_000226:exon1:c.C195T:p.G65G	NA	rs8070680	NA
chr17	39958344	39958344	T	C	UTR3	LEPREL4	NA	NA	NA	rs1061843	NA
chr17	40023618	40023618	A	-	UTR3	ACLY	NA	NA	NA	NA	NA
chr17	40465437	40465437	T	A	UTR3	STAT3	NA	NA	NA	NA	NA
chr17	41031195	41031195	-	A	ncRNA_exonic	LINC00671	NA	NA	NA	NA	NA
chr17	41064988	41064995	TTTTAGAA	-	UTR3	G6PC	NA	NA	NA	rs71157674	NA
chr17	41120550	41120550	A	-	UTR3	PTGES3L	NA	NA	NA	rs71690436	NA
chr17	41133071	41133071	T	C	exonic	RUNDC1	nonsynonymous SNV	RUNDC1:NM_173079:exon1:c.T478C:p.W160R	NA	rs1708875	B
chr17	41181922	41181924	AAA	-	UTR3	RND2	NA	NA	NA	NA	NA
chr17	42266722	42266722	A	G	exonic	TMUB2	nonsynonymous SNV	:p.E103G,TMUB2:NM_177441:exon2:c.A308G:p.E103G,TMUB2:NM_	NA	NA	D
chr17	42266741	42266741	T	G	exonic	TMUB2	synonymous SNV	:p.G109G,TMUB2:NM_177441:exon2:c.T327G:p.G109G,TMUB2:NM_	NA	NA	NA
chr17	42747647	42747647	G	A	intronic	C17orf104	NA	NA	NA	rs199529649	NA
chr17	42747657	42747657	G	A	intronic	C17orf104	NA	NA	NA	NA	NA
chr17	42747947	42747947	A	C	intronic	C17orf104	NA	NA	NA	NA	NA
chr17	42876925	42876925	-	A	UTR3	GJC1	NA	NA	NA	NA	NA
chr17	42878302	42878302	A	-	UTR3	GJC1	NA	NA	NA	NA	NA
chr17	44412780	44412780	A	G	UTR3	ARL17A	NA	NA	NA	rs62073354	NA
chr17	44950122	44950122	T	C	exonic	WNT9B	nonsynonymous SNV	WNT9B:NM_003396:exon2:c.T317C:p.M106T	NA	rs4968281	NA
chr17	45214604	45214604	A	T	exonic	CDC27	nonsynonymous SNV	)1114091:exon14:c.T1845A:p.H615Q,CDC27:NM_001256:exon14:c.T1	NA	rs75661039	NA
chr17	45214605	45214605	T	C	exonic	CDC27	nonsynonymous SNV	)1114091:exon14:c.A1844G:p.H615R,CDC27:NM_001256:exon14:c.A1	NA	rs76926116	NA
chr17	45214643	45214643	A	C	exonic	CDC27	synonymous SNV	)1114091:exon14:c.T1806G:p.V602V,CDC27:NM_001256:exon14:c.T1	NA	rs138020641	NA
chr17	45214682	45214682	A	G	exonic	CDC27	synonymous SNV	)1114091:exon14:c.T1767C:p.H589H,CDC27:NM_001256:exon14:c.T1	NA	rs62075622	NA
chr17	45214690	45214690	G	A	exonic	CDC27	nonsynonymous SNV	)1114091:exon14:c.C1759T:p.R587W,CDC27:NM_001256:exon14:c.C1	NA	rs74390782	NA
chr17	46669247	46669247	T	-	ncRNA_UTR3	HOXB5	NA	NA	NA	NA	NA
chr17	46802286	46802286	T	C	UTR3	HOXB13	NA	NA	NA	NA	NA
chr17	46802323	46802323	G	C	UTR3	HOXB13	NA	NA	NA	NA	NA
chr17	46802341	46802341	T	C	UTR3	HOXB13	NA	NA	NA	NA	NA
chr17	46941419	46941419	G	T	UTR3	CALCOCO2	NA	NA	NA	rs6504584	NA
chr17	47132505	47132505	A	-	UTR3	IGF2BP1	NA	NA	NA	NA	NA
chr17	47132517	47132517	A	C	UTR3	IGF2BP1	NA	NA	NA	rs56112814	NA
chr17	47132531	47132531	T	A	UTR3	IGF2BP1	NA	NA	NA	NA	NA
chr17	47133198	47133198	-	T	UTR3	IGF2BP1	NA	NA	NA	NA	NA
chr17	47373328	47373330	CAG	-	UTR3	ZNF652	NA	NA	NA	NA	NA
chr17	48046626	48046626	-	G	UTR5	DLX4	NA	NA	NA	rs71352544	NA
chr17	48046963	48046963	A	G	exonic	DLX4	nonsynonymous SNV	DLX4:NM_138281:exon1:c.A131G:p.N44S	NA	rs61749026	B
chr17	48151353	48151353	T	C	exonic	ITGA3	synonymous SNV	_002204:exon8:c.T1230C:p.L410L,ITGA3:NM_005501:exon8:c.T1230	NA	rs2285524	NA
chr17	48262615	48262615	A	T	UTR3	COL1A1	NA	NA	NA	NA	NA
chr17	48267226	48267226	A	G	exonic	COL1A1	synonymous SNV	COL1A1:NM_000088:exon37:c.T2607C:p.G869G	NA	NA	NA
chr17	48768770	48768770	C	A	UTR3	ABCC3	NA	NA	NA	NA	NA
chr17	48772571	48772571	C	G	UTR3	ANKRD40	NA	NA	NA	NA	NA
chr17	49197932	49197932	C	A	exonic	SPAG9	nonsynonymous SNV	.G86T:p.G29V,SPAG9:NM_001130528:exon1:c.G86T:p.G29V,SPAG9:N	NA	NA	P
chr17	49350802	49350802	A	C	exonic	UTP18	synonymous SNV	UTP18:NM_016001:exon5:c.A702C:p.G234G	NA	rs2318789	NA
chr17	53241197	53241197	-	A	UTR3	STXBP4	NA	NA	NA	rs11387036	NA
chr17	53828706	53828706	C	G	UTR5	PCTP	NA	NA	NA	rs890488	NA
chr17	54450134	54450134	G	A	exonic	ANKFN1	synonymous SNV	ANKFN1:NM_153228:exon6:c.G738A:p.K246K	NA	rs8069322	NA
chr17	54672363	54672363	A	C	UTR3	NOG	NA	NA	NA	rs1442829	NA
chr17	54672366	54672366	A	C	UTR3	NOG	NA	NA	NA	NA	NA
chr17	54672393	54672394	TT	-	UTR3	NOG	NA	NA	NA	NA	NA
chr17	54912339	54912339	G	A	exonic	DGKE	synonymous SNV	DGKE:NM_003647:exon2:c.G183A:p.G61G	NA	rs1048159	NA
chr17	55940421	55940421	A	-	UTR3	CUEDC1	NA	NA	NA	rs11335586	NA

chr17	59943721	59943721	A	-	UTR3	INTS2	NA	NA	NA	NA	NA
chr17	60020017	60020017	-	T	UTR3	MED13	NA	NA	NA	NA	NA
chr17	60342186	60342186	T	C	ncRNA_exonic	TBC1D3P2	NA	NA	NA	rs11550753	NA
chr17	62122727	62122727	A	G	exonic	ERN1	nonsynonymous SNV	ERN1:NM_001433:exon20:c.T2645C:p.L882P	NA	NA	NA
chr17	62399872	62399872	C	T	UTR3	PECAM1	NA	NA	NA	rs9902260	NA
chr17	62892413	62892413	G	A	exonic	LRRC37A3	synonymous SNV	LRRC37A3:NM_199340:exon3:c.C963T:p.A321A	NA	rs3894937	NA
chr17	62892442	62892442	G	C	exonic	LRRC37A3	nonsynonymous SNV	LRRC37A3:NM_199340:exon3:c.C934G:p.P312A	NA	rs201370086	P
chr17	64208285	64208285	C	G	exonic	APOH	nonsynonymous SNV	APOH:NM_000042:exon8:c.G1004C:p.W335S	NA	rs1801690	D
chr17	65070977	65070977	A	-	UTR3	HELZ	NA	NA	NA	rs199909135	NA
chr17	65073836	65073836	A	-	UTR3	HELZ	NA	NA	NA	NA	NA
chr17	65074050	65074050	T	-	UTR3	HELZ	NA	NA	NA	NA	NA
chr17	66129341	66129341	T	-	ncRNA_exonic	LINC00674	NA	NA	NA	NA	NA
chr17	66196220	66196220	C	A	ncRNA_exonic	LOC440461	NA	NA	NA	NA	NA
chr17	67017930	67017930	T	C	exonic	ABCA9	nonsynonymous SNV	ABCA9:NM_080283:exon18:c.A2354G:p.N785S	NA	rs17684521	D
chr17	68131149	68131149	T	-	UTR3	KCNJ16	NA	NA	NA	NA	NA
chr17	71745520	71745520	A	G	ncRNA_exonic	LINC00469	NA	NA	NA	rs12450057	NA
chr17	72270424	72270433	GCCGCGAC	-	UTR5	DNAI2	NA	NA	NA	rs140867882	NA
chr17	72350362	72350362	G	A	exonic	KIF19	synonymous SNV	KIF19:NM_153209:exon18:c.G2370A:p.S790S	NA	rs72852235	NA
chr17	72443258	72443258	T	C	UTR3	GPRC5C	NA	NA	NA	NA	NA
chr17	72521897	72521897	G	A	exonic	CD300LB	synonymous SNV	CD300LB:NM_174892:exon2:c.C471T:p.I157I	NA	rs17553512	NA
chr17	72869078	72869078	A	G	UTR5	FDXR	NA	NA	NA	rs492095	NA
chr17	72875677	72875677	G	T	exonic	FADS6	nonsynonymous SNV	FADS6:NM_178128:exon5:c.C763A:p.P255T	NA	NA	NA
chr17	73234012	73234012	C	T	UTR3	GGA3	NA	NA	NA	rs113574403	NA
chr17	73498623	73498623	A	G	exonic	CASKIN2	synonymous SNV	001142643:exon17:c.T2286C:p.S762S,CASKIN2:NM_020753:exon18:c.T	NA	rs7502835	NA
chr17	73498796	73498796	T	G	exonic	CASKIN2	nonsynonymous SNV	01142643:exon17:c.A2113C:p.T705P,CASKIN2:NM_020753:exon18:c.A	NA	NA	B
chr17	73498833	73498833	T	G	exonic	CASKIN2	synonymous SNV	01142643:exon17:c.A2076C:p.A692A,CASKIN2:NM_020753:exon18:c.A	NA	NA	NA
chr17	73926121	73926121	C	A	exonic	FBF1	nonsynonymous SNV	FBF1:NM_001080542:exon6:c.G194T:p.G65V	NA	rs1135889	NA
chr17	73949540	73949540	G	C	exonic	ACOX1	nonsynonymous SNV	822G:p.I274M,ACOX1:NM_004035:exon7:c.C936G:p.I312M,ACOX1:NM	NA	rs1135640	B
chr17	74288298	74288298	A	T	exonic	QRICH2	nonsynonymous SNV	QRICH2:NM_032134:exon4:c.T2012A:p.V671D	NA	NA	B
chr17	74288943	74288943	C	T	exonic	QRICH2	stopgain SNV	QRICH2:NM_032134:exon4:c.G1367A:p.W456X	NA	NA	NA
chr17	74288944	74288944	A	G	exonic	QRICH2	nonsynonymous SNV	QRICH2:NM_032134:exon4:c.T1366C:p.W456R	NA	rs11869057	B
chr17	74675810	74675810	G	A	UTR3	MXRA7	NA	NA	NA	rs1065604	NA
chr17	74922706	74922706	C	T	exonic	MGAT5B	synonymous SNV	p.F406F,MGAT5B:NM_001199172:exon10:c.C1185T:p.F395F,MGAT5B	NA	rs33966966	NA
chr17	74928758	74928758	C	T	exonic	MGAT5B	synonymous SNV	:p.F452F,MGAT5B:NM_001199172:exon11:c.C1323T:p.F441F,MGAT5B	NA	rs79286496	NA
chr17	75494705	75494705	A	G	exonic	9-Sep	nonsynonymous SNV	G:p.M569V,SEPT9:NM_001113494:exon11:c.A1234G:p.M412V,SEPT9:N	NA	rs2627223	NA
chr17	75494746	75494746	A	G	UTR3	9-Sep	NA	NA	NA	rs1059485	NA
chr17	75494761	75494761	G	C	UTR3	9-Sep	NA	NA	NA	NA	NA
chr17	76102136	76102136	-	A	UTR3	TNRC6C	NA	NA	NA	NA	NA
chr17	76102618	76102618	C	-	UTR3	TNRC6C	NA	NA	NA	NA	NA
chr17	76102966	76102966	A	-	UTR3	TNRC6C	NA	NA	NA	rs72217904	NA
chr17	76497920	76497920	C	A	exonic	DNAH17	nonsynonymous SNV	DNAH17:NM_173628:exon34:c.G5226T:p.M1742I	NA	rs690844	NA
chr17	76525759	76525759	G	C	exonic	DNAH17	nonsynonymous SNV	DNAH17:NM_173628:exon22:c.C3302G:p.A1101G	NA	rs61741523	NA
chr17	76670610	76670610	T	G	UTR3	CYTH1	NA	NA	NA	NA	NA
chr17	76670626	76670626	T	C	UTR3	CYTH1	NA	NA	NA	NA	NA
chr17	76993546	76993546	A	G	exonic	CANT1	synonymous SNV	C:p.A53A,CANT1:NM_001159773:exon3:c.T159C:p.A53A,CANT1:NM_(	NA	rs8077024	NA
chr17	77807074	77807074	T	-	UTR3	CBX4	NA	NA	NA	rs11350225	NA
chr17	79083194	79083196	AAG	-	UTR3	BAIAP2	NA	NA	NA	NA	NA
chr17	79140505	79140505	C	G	ncRNA_exonic	AATK-AS1	NA	NA	NA	rs12946891	NA
chr17	79612161	79612161	C	T	exonic	TSPAN10	unknown	UNKNOWN	NA	rs6565616	NA
chr17	79615604	79615604	A	C	UTR3	TSPAN10	NA	NA	NA	NA	NA
chr17	79878961	79878961	T	G	UTR3	MAFG	NA	NA	NA	NA	NA
chr17	79879436	79879436	T	-	UTR3	MAFG	NA	NA	NA	NA	NA
chr17	79888377	79888377	A	-	ncRNA_exonic	MAFG-AS1	NA	NA	NA	NA	NA
chr17	80086386	80086386	C	T	exonic	CCDC57	nonsynonymous SNV	CCDC57:NM_198082:exon15:c.G2329A:p.A777T	NA	rs7406162	NA
chr17	80086389	80086389	C	T	exonic	CCDC57	nonsynonymous SNV	CCDC57:NM_198082:exon15:c.G2326A:p.D776N	NA	rs7406163	NA
chr17	80279412	80279412	T	A	UTR3	SECTM1	NA	NA	NA	rs1132115	NA
chr17	80279428	80279428	G	A	UTR3	SECTM1	NA	NA	NA	rs1132114	NA
chr17	80561596	80561599	TTTT	-	UTR3	FOXK2	NA	NA	NA	NA	NA
chr17	81177461	81177461	A	G	ncRNA_exonic	FLJ43681	NA	NA	NA	rs3897846	NA
chr18	319513	319514	AA	-	UTR3	COLEC12	NA	NA	NA	NA	NA
chr18	334742	334742	C	T	exonic	COLEC12	nonsynonymous SNV	COLEC12:NM_130386:exon6:c.G1816A:p.G606S	NA	rs2305027	D
chr18	2943456	2943456	-	TGTGTT	ncRNA_exonic	LOC727896	NA	NA	NA	NA	NA
chr18	3168816	3168816	G	A	exonic	MYOM1	synonymous SNV	_003803:exon9:c.C1338T:p.N446N,MYOM1:NM_019856:exon9:c.C13	NA	rs2230167	NA
chr18	5244962	5244962	-	TG	ncRNA_exonic	LINC00667	NA	NA	NA	rs202003428	NA
chr18	5244995	5244995	T	C	ncRNA_exonic	LINC00667	NA	NA	NA	rs15920	NA
chr18	6943348	6943348	G	T	exonic	LAMA1	synonymous SNV	LAMA1:NM_005559:exon62:c.C8898A:p.P2966P	NA	NA	NA

chr18	6986227	6986227	G	A	exonic	LAMA1	nonsynonymous SNV	LAMA1:NM_005559:exon37:c.C5288T:p.A1763V	NA	rs12607841	B
chr18	8069868	8069868	C	T	exonic	PTPRM	synonymous SNV	)01105244:exon8:c.C1317T:p.N439N,PTPRM:NM_002845:exon8:c.C13	NA	rs2230601	NA
chr18	8387219	8387219	G	C	exonic	PTPRM	synonymous SNV	2845:exon29:c.G4155C:p.T1385T,PTPRM:NM_001105244:exon31:c.G4	NA	rs593950	NA
chr18	9959719	9959722	AAAA	-	UTR3	VAPA	NA	NA	NA	NA	NA
chr18	12986727	12986727	T	-	UTR3	SEH1L	NA	NA	NA	rs199544910	NA
chr18	13612545	13612546	CC	-	UTR5	LDLRAD4	NA	NA	NA	NA	NA
chr18	13665381	13665385	AAAAA	-	UTR3	FAM210A	NA	NA	NA	rs71174180	NA
chr18	14542867	14542867	C	T	exonic	POTEC	synonymous SNV	POTEC:NM_001137671:exon1:c.G279A:p.T93T	NA	rs200779556	NA
chr18	14779969	14779969	C	G	exonic	ANKRD30B	nonsynonymous SNV	ANKRD30B:NM_001145029:exon11:c.C1431G:p.F477L	1130088,COSM1130087;OCCURENCE=1	rs9675365	NA
chr18	20577669	20577669	G	A	exonic	RBBP8	synonymous SNV	5A:p.K705K,RBBP8:NM_203291:exon14:c.G2115A:p.K705K,RBBP8:NM	NA	rs17852769	NA
chr18	21390368	21390368	T	C	exonic	LAMA3	nonsynonymous SNV	01127717:exon13:c.T1642C:p.C548R,LAMA3:NM_198129:exon13:c.T1	NA	NA	D
chr18	24442392	24442392	C	A	exonic	AQP4	synonymous SNV	NM_004028:exon1:c.G135T:p.P45P,AQP4:NM_001650:exon2:c.G201T:	NA	rs35248760	NA
chr18	24492336	24492336	-	ACAC	ncRNA_intronic	AQP4-AS1	NA	NA	NA	NA	NA
chr18	24492338	24492338	T	C	ncRNA_intronic	AQP4-AS1	NA	NA	NA	rs76675133	NA
chr18	29649164	29649164	A	-	UTR3	RNF125	NA	NA	NA	rs11340446	NA
chr18	31327378	31327378	A	-	UTR3	ASXL3	NA	NA	NA	NA	NA
chr18	32826672	32826672	A	-	UTR3	ZNF397	NA	NA	NA	NA	NA
chr18	32888397	32888397	G	A	ncRNA_exonic	ZNF271	NA	NA	NA	rs63352060	NA
chr18	33848663	33848663	-	TACAT	UTR3	MOCOS	NA	NA	NA	rs111317566	NA
chr18	42644774	42644774	A	-	UTR3	SETBP1	NA	NA	NA	rs57937684	NA
chr18	43331751	43331751	A	T	UTR3	SLC14A1	NA	NA	NA	rs544373	NA
chr18	43844698	43844698	-	T	UTR3	C18orf25	NA	NA	NA	NA	NA
chr18	44229149	44229149	C	T	exonic	LOXHD1	nonsynonymous SNV	LOXHD1:NM_144612:exon2:c.G214A:p.G72R	NA	NA	NA
chr18	47088655	47088655	T	G	UTR5	LIPG	NA	NA	NA	rs34474737	NA
chr18	47349245	47349245	A	C	UTR3	MYO5B	NA	NA	NA	NA	NA
chr18	47349683	47349683	C	T	UTR3	MYO5B	NA	NA	NA	NA	NA
chr18	47349684	47349684	C	G	UTR3	MYO5B	NA	NA	NA	NA	NA
chr18	47349692	47349692	T	G	UTR3	MYO5B	NA	NA	NA	NA	NA
chr18	47350330	47350330	C	A	UTR3	MYO5B	NA	NA	NA	NA	NA
chr18	47350346	47350346	G	C	UTR3	MYO5B	NA	NA	NA	NA	NA
chr18	47350353	47350353	C	T	UTR3	MYO5B	NA	NA	NA	NA	NA
chr18	47350357	47350357	T	A	UTR3	MYO5B	NA	NA	NA	NA	NA
chr18	47350373	47350373	A	C	UTR3	MYO5B	NA	NA	NA	NA	NA
chr18	47350377	47350377	G	A	UTR3	MYO5B	NA	NA	NA	NA	NA
chr18	47350394	47350394	A	G	UTR3	MYO5B	NA	NA	NA	NA	NA
chr18	47350395	47350395	A	G	UTR3	MYO5B	NA	NA	NA	NA	NA
chr18	47350400	47350400	A	G	UTR3	MYO5B	NA	NA	NA	NA	NA
chr18	47350406	47350406	A	T	UTR3	MYO5B	NA	NA	NA	NA	NA
chr18	47350683	47350683	C	T	UTR3	MYO5B	NA	NA	NA	rs74651470	NA
chr18	47908556	47908556	G	A	exonic	SKA1	nonsynonymous SNV	M_001039535:exon4:c.G271A:p.V91I,SKA1:NM_145060:exon4:c.G271	NA	rs6507992	B
chr18	48325302	48325302	C	A	UTR3	MRO	NA	NA	NA	rs2849256	NA
chr18	48584791	48584791	A	C	exonic	SMAD4	nonsynonymous SNV	SMAD4:NM_005359:exon7:c.A869C:p.H290P	NA	NA	D
chr18	48584794	48584794	A	C	exonic	SMAD4	nonsynonymous SNV	SMAD4:NM_005359:exon7:c.A872C:p.H291P	NA	NA	P
chr18	48610371	48610372	CA	-	UTR3	SMAD4	NA	NA	NA	NA	NA
chr18	48610377	48610388	CGCGCACA	-	UTR3	SMAD4	NA	NA	NA	NA	NA
chr18	51822710	51822710	C	T	UTR3	POLI	NA	NA	NA	rs518069	NA
chr18	51887123	51887123	C	T	exonic	C18orf54	synonymous SNV	C18orf54:NM_173529:exon2:c.C181T:p.L61L	NA	rs1657904	NA
chr18	51905870	51905870	T	-	UTR3	C18orf54	NA	NA	NA	NA	NA
chr18	52890959	52890961	TTT	-	UTR3	TCF4	NA	NA	NA	NA	NA
chr18	55215119	55215119	T	C	UTR3	FECH	NA	NA	NA	rs8090261	NA
chr18	55216655	55216655	-	T	UTR3	FECH	NA	NA	NA	NA	NA
chr18	55216771	55216771	T	G	UTR3	FECH	NA	NA	NA	NA	NA
chr18	55216772	55216772	T	A	UTR3	FECH	NA	NA	NA	NA	NA
chr18	55216773	55216773	T	G	UTR3	FECH	NA	NA	NA	NA	NA
chr18	55862847	55862847	G	A	UTR5	NEDD4L	NA	NA	NA	rs1008899	NA
chr18	56415902	56415902	G	A	UTR3	MALT1	NA	NA	NA	rs62093496	NA
chr18	56962800	56962801	AA	-	UTR3	CPLX4	NA	NA	NA	rs61050481	NA
chr18	56963852	56963852	T	G	UTR3	CPLX4	NA	NA	NA	rs17696504	NA
chr18	59712463	59712463	G	A	UTR3	PIGN	NA	NA	NA	rs28375341	NA
chr18	59780393	59780393	T	A	exonic	PIGN	nonsynonymous SNV	I_012327:exon15:c.A1408T:p.I470L,PIGN:NM_176787:exon16:c.A1408	NA	rs3862712	NA
chr18	59814268	59814268	G	A	exonic	PIGN	synonymous SNV	M_012327:exon8:c.C741T:p.H247H,PIGN:NM_176787:exon9:c.C741T:p	NA	rs9320000	NA
chr18	60052586	60052586	G	T	UTR3	TNFRSF11A	NA	NA	NA	NA	NA
chr18	60052587	60052587	G	C	UTR3	TNFRSF11A	NA	NA	NA	NA	NA
chr18	60237388	60237388	A	G	exonic	ZCCHC2	synonymous SNV	ZCCHC2:NM_017742:exon12:c.A1899G:p.S633S	NA	rs8096750	NA
chr18	60995589	60995589	T	-	UTR3	KDSR	NA	NA	NA	rs11349419	NA
chr18	61377579	61377579	A	C	exonic	SERPINB11	unknown	UNKNOWN	NA	rs1395268	NA

chr18	67509199	67509199	-	GTGTGTGT	UTR3	DOK6	NA	NA	NA	rs143503551	NA
chr18	67515008	67515008	-	T	UTR3	DOK6	NA	NA	NA	NA	NA
chr18	72168608	72168608	G	A	exonic	CNDP2	synonymous SNV	M_001168499:exon2:c.G105A:p.P35P,CNDP2:NM_018235:exon3:c.G10	=COSM148444;OCCURENCE=1(stomac	rs2303463	NA
chr18	72234635	72234635	C	T	exonic	CNDP1	synonymous SNV	CNDP1:NM_032649:exon6:c.C723T:p.Y241Y	NA	rs12960862	NA
chr18	72260399	72260399	G	A	ncRNA_exonic	LOC400657	NA	NA	NA	rs10782060	NA
chr18	72593032	72593032	C	G	exonic	ZNF407	synonymous SNV	01146189:exon5:c.C5085G:p.G1695G,ZNF407:NM_017757:exon5:c.C5(	NA	NA	NA
chr18	72922851	72922851	-	GG	UTR5	TSHZ1	NA	NA	NA	NA	NA
chr18	74728109	74728109	-	TT	UTR3	MBP	NA	NA	NA	NA	NA
chr18	77473127	77473127	C	T	exonic	CTDP1	nonsynonymous SNV	52T:p.T221M,CTDP1:NM_004715:exon7:c.C1019T:p.T340M,CTDP1:NM	NA	rs2279103	B
chr18	77916875	77916875	T	-	ncRNA_UTR3	PARD6G	NA	NA	NA	rs145797175	NA
chr18	77917479	77917479	G	A	ncRNA_UTR3	PARD6G	NA	NA	NA	rs62101564	NA
chr18	77936214	77936214	G	A	ncRNA_exonic	PARD6G-AS1	NA	NA	NA	rs11660391	NA
chr19	199636	199636	G	T	ncRNA_exonic	FLJ45445	NA	NA	NA	rs4897924	NA
chr19	199880	199880	T	G	ncRNA_exonic	FLJ45445	NA	NA	ID=COSN394552;OCCURENCE=1(lung)	rs11878357	NA
chr19	536265	536265	T	C	exonic	CDC34	nonsynonymous SNV	CDC34:NM_004359:exon3:c.T287C:p.I96T	NA	NA	D
chr19	536268	536268	T	C	exonic	CDC34	nonsynonymous SNV	CDC34:NM_004359:exon3:c.T290C:p.L97P	NA	NA	D
chr19	583453	583453	A	C	UTR3	BSG	NA	NA	NA	NA	NA
chr19	682259	682259	A	G	UTR3	FSTL3	NA	NA	NA	NA	NA
chr19	863548	863548	G	T	UTR3	CFD	NA	NA	NA	NA	NA
chr19	1008683	1008683	G	A	exonic	GRIN3B	nonsynonymous SNV	GRIN3B:NM_138690:exon7:c.G2533A:p.A845T	NA	rs2285906	P
chr19	1037969	1037971	TTT	-	UTR3	CNN2	NA	NA	NA	rs150855033	NA
chr19	1047161	1047161	A	G	exonic	ABCA7	synonymous SNV	ABCA7:NM_019112:exon15:c.A1851G:p.G617G	NA	rs3752237	NA
chr19	1049269	1049269	G	A	exonic	ABCA7	synonymous SNV	ABCA7:NM_019112:exon18:c.G2385A:p.L795L	NA	rs4147914	NA
chr19	1398611	1398611	T	-	UTR3	GAMT	NA	NA	NA	rs61684562	NA
chr19	1472595	1472595	A	C	UTR3	APC2	NA	NA	NA	NA	NA
chr19	1554912	1554912	-	A	UTR3	MEX3D	NA	NA	NA	NA	NA
chr19	1954126	1954126	-	A	ncRNA_exonic	CSNK1G2-AS1	NA	NA	NA	rs34622118	NA
chr19	1954128	1954128	T	C	ncRNA_exonic	CSNK1G2-AS1	NA	NA	NA	rs62129474	NA
chr19	2072038	2072038	T	C	UTR3	MOB3A	NA	NA	NA	rs8102231	NA
chr19	2072746	2072746	A	-	UTR3	MOB3A	NA	NA	NA	NA	NA
chr19	2226847	2226847	G	T	exonic	DOT1L	nonsynonymous SNV	DOT1L:NM_032482:exon27:c.G4327T:p.G1443C	NA	rs113842228	D
chr19	3198841	3198841	G	C	exonic	NCLN	nonsynonymous SNV	NCLN:NM_020170:exon5:c.G642C:p.E214D	NA	rs11671067	B
chr19	3593004	3593004	A	G	UTR3	GIPC3	NA	NA	NA	rs17433775	NA
chr19	3767265	3767265	C	T	exonic	MRPL54	synonymous SNV	MRPL54:NM_172251:exon3:c.C291T:p.F97F	NA	rs7239	NA
chr19	3823292	3823292	G	A	exonic	ZFR2	synonymous SNV	ZFR2:NM_015174:exon8:c.C1323T:p.P441P	NA	rs61744821	NA
chr19	4045564	4045564	C	A	UTR3	ZBTB7A	NA	NA	NA	NA	NA
chr19	4045639	4045639	A	C	UTR3	ZBTB7A	NA	NA	NA	NA	NA
chr19	4046431	4046432	TT	-	UTR3	ZBTB7A	NA	NA	NA	NA	NA
chr19	4153655	4153655	G	T	UTR5	CREB3L3	NA	NA	NA	NA	NA
chr19	4280186	4280186	C	T	exonic	SHD	synonymous SNV	SHD:NM_020209:exon1:c.C126T:p.F42F	NA	rs10419363	NA
chr19	4442999	4442999	T	G	exonic	CHAF1A	nonsynonymous SNV	CHAF1A:NM_005483:exon15:c.T2848G:p.S950A	NA	rs243383	B
chr19	4502937	4502937	A	C	UTR3	PLIN4	NA	NA	NA	NA	NA
chr19	4511680	4511680	A	G	exonic	PLIN4	synonymous SNV	PLIN4:NM_001080400:exon3:c.T2250C:p.D750D	NA	NA	NA
chr19	4512877	4512877	G	A	exonic	PLIN4	synonymous SNV	PLIN4:NM_001080400:exon3:c.C1053T:p.G351G	NA	rs61730751	NA
chr19	4513006	4513006	G	A	exonic	PLIN4	synonymous SNV	PLIN4:NM_001080400:exon3:c.C924T:p.G308G	NA	rs73920831	NA
chr19	4675418	4675418	A	G	UTR3	DPP9	NA	NA	NA	NA	NA
chr19	4867690	4867690	A	G	UTR5	PLIN3	NA	NA	NA	rs262559	NA
chr19	5153606	5153606	G	A	UTR3	KDM4B	NA	NA	NA	rs11514	NA
chr19	5206072	5206072	A	-	UTR3	PTPRS	NA	NA	NA	NA	NA
chr19	5206246	5206249	TTTG	-	UTR3	PTPRS	NA	NA	NA	rs138275722	NA
chr19	5206261	5206261	-	A	UTR3	PTPRS	NA	NA	NA	rs199687996	NA
chr19	5206276	5206276	A	-	UTR3	PTPRS	NA	NA	NA	rs141036801	NA
chr19	5587299	5587299	T	G	exonic	SAFB2	synonymous SNV	SAFB2:NM_014649:exon21:c.A2817C:p.P939P	NA	NA	NA
chr19	5587303	5587303	T	G	exonic	SAFB2	nonsynonymous SNV	SAFB2:NM_014649:exon21:c.A2813C:p.H938P	NA	NA	NA
chr19	5622905	5622905	-	C	UTR5	SAFB2	NA	NA	NA	NA	NA
chr19	5910015	5910015	-	G	UTR3	VMAC	NA	NA	NA	rs76336657	NA
chr19	6312290	6312290	T	C	exonic	ACER1	nonsynonymous SNV	ACER1:NM_133492:exon3:c.A220G:p.M74V	NA	rs72981971	D
chr19	6535372	6535373	GG	-	UTR3	TNFSF9	NA	NA	NA	NA	NA
chr19	6718387	6718387	G	C	exonic	C3	nonsynonymous SNV	C3:NM_000064:exon3:c.C304G:p.R102G	NA	rs2230199	B
chr19	7051376	7051376	G	A	exonic	MBD3L2	nonsynonymous SNV	MBD3L2:NM_144614:exon2:c.G370A:p.G124S	NA	rs200081225	B
chr19	7056553	7056553	C	T	exonic	MBD3L3	nonsynonymous SNV	MBD3L3:NM_001164425:exon2:c.G407A:p.R136H	NA	rs199680919	NA
chr19	7086367	7086367	C	T	UTR3	ZNF557	NA	NA	NA	NA	NA
chr19	7087549	7087549	A	-	UTR3	ZNF557	NA	NA	NA	rs11287183	NA
chr19	7114059	7114060	AA	-	UTR3	INSR	NA	NA	NA	NA	NA
chr19	7116712	7116713	AA	-	UTR3	INSR	NA	NA	NA	rs71177157	NA
chr19	7116733	7116733	G	A	UTR3	INSR	NA	NA	NA	NA	NA

chr19	7528734	7528734	A	G	exonic	ARHGEF18	nonsynonymous SNV	01130955:exon12:c.A2102G:p.Q701R,ARHGEF18:NM_015318:exon13:c	NA	rs2287918	B		
chr19	7536477	7536477	C	G	UTR3	ARHGEF18	NA	NA	NA	NA	NA		
chr19	7536507	7536507	T	G	UTR3	ARHGEF18	NA	NA	NA	NA	NA		
chr19	7547074	7547074	A	C	exonic	PEX11G	nonsynonymous SNV	_001270539:exon3:c.T273G:p.C91W,PEX11G:NM_080662:exon3:c.T27	NA	rs2303146	B		
chr19	7571030	7571030	T	A	exonic	C19orf45	nonsynonymous SNV	C19orf45:NM_198534:exon7:c.T1186A:p.Y396N	NA	rs3826736	B		
chr19	7744076	7744076	C	-	UTR3	C19orf59	NA	NA	NA	NA	NA		
chr19	7855113	7855114	GT	-	ncRNA_exonic	CLEC4GP1	NA	NA	NA	NA	NA		
chr19	7977929	7977929	C	-	UTR3	MAP2K7	NA	NA	NA	NA	NA		
chr19	7977931	7977933	CTC	-	UTR3	MAP2K7	NA	NA	NA	NA	NA		
chr19	7978407	7978407	G	A	UTR3	MAP2K7	NA	NA	NA	rs4804835	NA		
chr19	7989578	7989578	G	T	UTR3	CTXN1	NA	NA	NA	NA	NA		
chr19	8176569	8176569	G	A	exonic	FBN3	synonymous SNV	FBN3:NM_032447:exon31:c.C4047T:p.C1349C	NA	rs35306870	NA		
chr19	9878987	9878987	G	-	UTR5	ZNF846	NA	NA	NA	rs147535386	NA		
chr19	10089262	10089262	G	A	exonic	COL5A3	synonymous SNV	COL5A3:NM_015719:exon41:c.C3006T:p.P1002P	NA	rs12610207	NA		
chr19	10179657	10179657	G	A	ncRNA_exonic	C3P1	NA	NA	NA	rs897803	NA		
chr19	10267077	10267077	T	C	exonic	DNMT1	synonymous SNV	01379:exon17:c.A1341G:p.P447P,DNMT1:NM_001130823:exon18:c.A1	NA	rs2228611	NA		
chr19	10397238	10397238	C	T	UTR3	ICAM1	NA	NA	NA	rs281437	NA		
chr19	10449358	10449358	T	C	exonic	ICAM3	nonsynonymous SNV	ICAM3:NM_002162:exon2:c.A343G:p.R115G	NA	rs7258015	B		
chr19	10556897	10556897	C	T	exonic	PDE4A	synonymous SNV	I_001111308:exon2:c.C246T:p.F82F,PDE4A:NM_001111309:exon2:c.C	NA	rs118115488	NA		
chr19	10579165	10579165	G	C	UTR3	PDE4A	NA	NA	NA	NA	NA		
chr19	10664256	10664256	-	TAA	UTR3	KRI1	NA	NA	NA	rs201847069	NA		
chr19	11406839	11406839	C	G	UTR5	TSPAN16	NA	NA	NA	rs453766	NA		
chr19	11562302	11562302	G	T	UTR3	ELAVL3	NA	NA	NA	NA	NA		
chr19	11564606	11564606	A	C	UTR3	ELAVL3	NA	NA	NA	NA	NA		
chr19	11708361	11708361	C	T	UTR5	ZNF627	NA	NA	NA	rs11551815	NA		
chr19	12091196	12091196	-	A	UTR3	ZNF763	NA	NA	NA	NA	NA		
chr19	12405552	12405552	T	C	UTR5	ZNF44	NA	NA	NA	rs386901	NA		
chr19	12428307	12428307	T	G	UTR3	ZNF563	NA	NA	NA	rs78023129	NA		
chr19	12572300	12572300	G	A	UTR3	ZNF709	NA	NA	NA	rs4804194	NA		
chr19	12574741	12574741	A	-	UTR3	ZNF709	NA	NA	NA	NA	NA		
chr19	12774537	12774537	G	A	exonic	MAN2B1	nonsynonymous SNV	I_000528:exon5:c.C743T:p.P248L,MAN2B1:NM_001173498:exon5:c.C7	NA	rs117843968	D		
chr19	12958697	12958697	T	C	exonic	MAST1	synonymous SNV	MAST1:NM_014975:exon7:c.T600C:p.F200F	NA	rs2290688	NA		
chr19	12996740	12996740	A	G	exonic	KLF1	nonsynonymous SNV	KLF1:NM_006563:exon2:c.T304C:p.S102P	NA	rs2072597	B		
chr19	13054781	13054781	G	T	UTR3	CALR	NA	NA	NA	rs1049481	NA		
chr19	13942986	13942986	A	T	UTR3	ZSWIM4	NA	NA	NA	NA	NA		
chr19	13943034	13943034	G	T	UTR3	ZSWIM4	NA	NA	NA	NA	NA		
chr19	14627776	14627776	A	G	exonic	DNAJB1	synonymous SNV	DNAJB1:NM_006145:exon2:c.T294C:p.P98P	NA	NA	NA		
chr19	14627800	14627800	G	A	exonic	DNAJB1	synonymous SNV	DNAJB1:NM_006145:exon2:c.C270T:p.F90F	NA	NA	NA		
chr19	14627816	14627816	-	C	exonic	DNAJB1	frameshift insertion	DNAJB1:NM_006145:exon2:c.254_255insG:p.A85fs	NA	NA	NA		
chr19	14627818	14627818	A	C	exonic	DNAJB1	synonymous SNV	DNAJB1:NM_006145:exon2:c.T252G:p.G84G	NA	NA	NA		
chr19	14627821	14627821	A	C	exonic	DNAJB1	synonymous SNV	DNAJB1:NM_006145:exon2:c.T249G:p.G83G	NA	NA	NA		
chr19	14682804	14682804	G	C	exonic	NDUFB7	synonymous SNV	NDUFB7:NM_004146:exon1:c.C9G:p.A3A	NA	rs9543	NA		
chr19	14769339	14769339	C	G	exonic	EMR3	nonsynonymous SNV	EMR3:NM_032571:exon5:c.G379C:p.E127Q	NA	rs4606855	B		
chr19	14862430	14862430	C	G	exonic	EMR2	nonsynonymous SNV	01271052:exon14:c.G1668C:p.L556F,EMR2:NM_013447:exon16:c.G18	NA	rs2524383	B		
chr19	15292605	15292605	G	T	exonic	NOTCH3	stopgain SNV	NOTCH3:NM_000435:exon17:c.C2574A:p.C858X	NA	NA	NA		
chr19	15575174	15575174	G	-	UTR5	RASAL3	NA	NA	NA	NA	NA		
chr19	15996820	15996820	G	A	exonic	CYP4F2	synonymous SNV	CYP4F2:NM_001082:exon9:c.C1029T:p.H343H	NA	rs2074900	NA		
chr19	16023492	16023492	T	A	UTR3	CYP4F11	NA	NA	NA	NA	NA		
chr19	16040292	16040292	A	G	exonic	CYP4F11	synonymous SNV	M_021187:exon2:c.T318C:p.I106I,CYP4F11:NM_001128932:exon3:c.T3	NA	rs3765070	NA		
chr19	16662564	16662565	TT	-	UTR3	SLC35E1	NA	NA	NA	NA	NA		
chr19	16902167	16902167	G	T	exonic	NWD1	nonsynonymous SNV	NWD1:NM_001007525:exon14:c.G2947T:p.A983S	NA	NA	B		
chr19	17434120	17434120	-	TGTG	UTR3	ANO8	NA	NA	NA	NA	NA		
chr19	17660300	17660300	G	A	exonic	FAM129C	nonsynonymous SNV	01098524:exon15:c.G1807A:p.G603S,FAM129C:NM_173544:exon15:c.	=COSM148530;OCCURENCE=1(stomach	rs11666267	B		
chr19	17692719	17692719	T	-	UTR3	COLGALT1	NA	NA	NA	rs34244232	NA		
chr19	17716794	17716794	C	T	UTR3	UNC13A	NA	NA	NA	rs12974304	NA		
chr19	17932190	17932190	T	C	exonic	INSL3	synonymous SNV	M_001265587:exon1:c.A126G:p.L42L,INSL3:NM_005543:exon1:c.A126	NA	rs1047233	NA		
chr19	18054643	18054643	C	T	UTR3	CCDC124	NA	NA	NA	rs1052564	NA		
chr19	18260786	18260789	TTTT	-	UTR3	MAST3	NA	NA	NA	rs71872759	NA		
chr19	18260796	18260797	TT	-	UTR3	MAST3	NA	NA	NA	NA	NA		
chr19	18479249	18479252	AAAA	-	UTR3	PGPEP1	NA	NA	NA	NA	NA		
chr19	18499815	18499815	G	C	UTR3	GDF15	NA	NA	NA	rs1054564	NA		
chr19	18499858	18499858	T	C	UTR3	GDF15	NA	NA	NA	rs1054221	NA		
chr19	19023853	19023853	T	C	exonic	COPE	synonymous SNV	A129G:p.L43L,COPE:NM_199442:exon2:c.A129G:p.L43L,COPE:NM_19	=COSM1178458;OCCURENCE=1(prostat	rs3177137	NA		
chr19	19618060	19618060	-	A	UTR3	GATAD2A	NA	NA	NA	rs35542121	NA		
chr19	20115647	20115647	-	AC	UTR3	ZNF682	NA	NA	NA	rs201489153	NA		
chr19	20575357	20575357	T	C	intergenic	'0-1(dist=65194),ZNF826P(dis	NA	NA	NA	rs10425673	NA		

chr19	20722725	20722725	A	G	UTR3	ZNF737	NA	NA	NA	NA	NA
chr19	20722726	20722726	C	T	UTR3	ZNF737	NA	NA	NA	rs73924681	NA
chr19	20722727	20722727	T	A	UTR3	ZNF737	NA	NA	NA	NA	NA
chr19	20722743	20722743	T	A	UTR3	ZNF737	NA	NA	NA	rs73924682	NA
chr19	20722787	20722787	A	T	UTR3	ZNF737	NA	NA	NA	rs138165402	NA
chr19	21306251	21306251	G	A	UTR3	ZNF714	NA	NA	NA	rs10413226	NA
chr19	21368412	21368412	A	G	UTR3	ZNF431	NA	NA	NA	rs113635752	NA
chr19	21561326	21561327	CA	-	ncRNA_exonic	ZNF738	NA	NA	NA	NA	NA
chr19	21561509	21561511	CTC	-	ncRNA_exonic	ZNF738	NA	NA	NA	rs145776873	NA
chr19	21561863	21561863	T	-	ncRNA_exonic	ZNF738	NA	NA	NA	rs35081700	NA
chr19	21564868	21564868	C	T	ncRNA_exonic	ZNF738	NA	NA	NA	NA	NA
chr19	21564868	21564868	-	T	ncRNA_exonic	ZNF738	NA	NA	NA	NA	NA
chr19	21565050	21565050	-	T	ncRNA_exonic	ZNF738	NA	NA	NA	NA	NA
chr19	21566249	21566249	T	A	ncRNA_exonic	ZNF738	NA	NA	NA	NA	NA
chr19	21590636	21590636	C	T	UTR3	ZNF493	NA	NA	NA	rs2359144	NA
chr19	22156855	22156855	G	A	exonic	ZNF208	synonymous SNV	ZNF208:NM_007153:exon4:c.C981T:p.T327T	NA	rs200969060	NA
chr19	22156858	22156858	T	C	exonic	ZNF208	synonymous SNV	ZNF208:NM_007153:exon4:c.A978G:p.S326S	NA	rs200113618	NA
chr19	22156863	22156863	C	A	exonic	ZNF208	nonsynonymous SNV	ZNF208:NM_007153:exon4:c.G973T:p.V325F	ID=COSM230374;OCCURENCE=1(NS)	rs202200782	NA
chr19	22363413	22363413	C	T	exonic	ZNF676	nonsynonymous SNV	ZNF676:NM_001001411:exon3:c.G1106A:p.G369E	NA	rs75690456	B
chr19	22363826	22363826	G	C	exonic	ZNF676	synonymous SNV	ZNF676:NM_001001411:exon3:c.C693G:p.G231G	NA	rs7252593	NA
chr19	22363869	22363869	A	C	exonic	ZNF676	nonsynonymous SNV	ZNF676:NM_001001411:exon3:c.T650G:p.V217G	NA	NA	B
chr19	22363893	22363893	A	G	exonic	ZNF676	nonsynonymous SNV	ZNF676:NM_001001411:exon3:c.T626C:p.F209S	NA	NA	B
chr19	22363896	22363896	T	C	exonic	ZNF676	nonsynonymous SNV	ZNF676:NM_001001411:exon3:c.A623G:p.K208R	NA	NA	B
chr19	22375868	22375868	C	T	exonic	ZNF676	nonsynonymous SNV	ZNF676:NM_001001411:exon2:c.G80A:p.G27E	NA	rs8104929	B
chr19	22379587	22379587	G	T	UTR5	ZNF676	NA	NA	NA	rs2360001	NA
chr19	22848991	22848991	T	-	UTR3	ZNF492	NA	NA	NA	rs202104125	NA
chr19	23556605	23556605	A	G	exonic	ZNF91	synonymous SNV	ZNF91:NM_003430:exon3:c.T192C:p.Y64Y	OSM288088;OCCURENCE=1(large_intes	rs34740519	NA
chr19	31766336	31766336	T	-	UTR3	TSHZ3	NA	NA	NA	rs57983390	NA
chr19	33088072	33088072	-	ATCA	UTR3	ANKRD27	NA	NA	NA	rs143829091	NA
chr19	33169172	33169172	-	C	UTR3	RGS9BP	NA	NA	NA	NA	NA
chr19	33467620	33467620	T	C	UTR3	C19orf40	NA	NA	NA	rs7247715	NA
chr19	34718608	34718608	T	-	UTR3	LSM14A	NA	NA	NA	NA	NA
chr19	34718735	34718735	A	G	UTR3	LSM14A	NA	NA	NA	rs201090664	NA
chr19	35175179	35175179	C	A	exonic	ZNF302	nonsynonymous SNV	M_001012320:exon5:c.C237A:p.N79K,ZNF302:NM_018443:exon5:c.C23	NA	NA	NA
chr19	35577870	35577870	G	A	ncRNA_exonic	HPN-AS1	NA	NA	NA	rs7250814	NA
chr19	35612159	35612159	C	G	exonic	FXYD3	nonsynonymous SNV	001136010:exon5:c.C107G:p.P36R,FXYD3:NM_001136009:exon6:c.C1	NA	rs2290649	NA
chr19	35617639	35617639	C	G	exonic	LGI4	synonymous SNV	LGI4:NM_139284:exon8:c.G834C:p.P278P	NA	rs1687998	NA
chr19	35739702	35739702	G	A	UTR5	LSR	NA	NA	NA	rs16970163	NA
chr19	35942122	35942122	T	A	UTR3	FFAR2	NA	NA	NA	NA	NA
chr19	35942146	35942146	G	A	UTR3	FFAR2	NA	NA	NA	NA	NA
chr19	36164350	36164350	A	C	exonic	UPK1A	nonsynonymous SNV	UPK1A:NM_007000:exon4:c.A371C:p.N124T	NA	NA	D
chr19	36430384	36430384	A	C	exonic	LRFN3	synonymous SNV	LRFN3:NM_024509:exon2:c.A57C:p.P19P	NA	NA	NA
chr19	36523759	36523759	G	T	UTR5	CLIP3	NA	NA	NA	rs62109714	NA
chr19	36546015	36546015	C	T	exonic	WDR62	nonsynonymous SNV	M_001083961:exon1:c.C142T:p.L48F,WDR62:NM_173636:exon1:c.C14	NA	rs62109744	B
chr19	36606379	36606379	-	CAG	UTR5	TBCB	NA	NA	NA	rs199646187	NA
chr19	36643433	36643438	GGGGCT	-	UTR5	COX7A1	NA	NA	NA	rs139129236	NA
chr19	36827322	36827322	A	G	UTR3	ZFP14	NA	NA	NA	rs2967502	NA
chr19	36827856	36827856	A	T	UTR3	ZFP14	NA	NA	NA	NA	NA
chr19	36827857	36827857	T	A	UTR3	ZFP14	NA	NA	NA	NA	NA
chr19	36827859	36827859	A	T	UTR3	ZFP14	NA	NA	NA	NA	NA
chr19	36830726	36830731	TATAAT	-	UTR3	ZFP14	NA	NA	NA	rs75308843	NA
chr19	36937172	36937173	TT	-	UTR3	ZNF566	NA	NA	NA	NA	NA
chr19	37620610	37620610	A	-	UTR3	ZNF420	NA	NA	NA	NA	NA
chr19	37759202	37759202	T	-	ncRNA_exonic	LOC284412	NA	NA	NA	NA	NA
chr19	38031167	38031167	-	A	UTR3	ZNF793	NA	NA	NA	NA	NA
chr19	39359607	39359607	T	-	UTR3	RINL	NA	NA	NA	NA	NA
chr19	39980412	39980412	C	T	exonic	TIMM50	synonymous SNV	TIMM50:NM_001001563:exon11:c.C1323T:p.F441F	NA	rs114194716	NA
chr19	40021719	40021719	A	-	UTR3	EID2B	NA	NA	NA	rs34833151	NA
chr19	40376662	40376662	G	C	exonic	FCGBP	nonsynonymous SNV	FCGBP:NM_003890:exon24:c.C11760G:p.H3920Q	NA	rs2542318	NA
chr19	40389741	40389741	A	G	exonic	FCGBP	nonsynonymous SNV	FCGBP:NM_003890:exon18:c.T8441C:p.V2814A	NA	rs141158749	NA
chr19	40946794	40946794	A	-	UTR3	SERTAD3	NA	NA	NA	NA	NA
chr19	41082868	41082868	T	C	exonic	SHKBP1	synonymous SNV	SHKBP1:NM_138392:exon1:c.T63C:p.I21I	NA	rs16974365	NA
chr19	41524087	41524087	A	G	UTR3	CYP2B6	NA	NA	NA	rs707265	NA
chr19	41903220	41903220	G	A	exonic	EXOSC5	nonsynonymous SNV	EXOSC5:NM_020158:exon1:c.C14T:p.T5M	NA	rs10853751	B
chr19	42470853	42470853	A	G	UTR3	ATP1A3	NA	NA	NA	rs199854166	NA
chr19	42595123	42595123	T	-	UTR3	POU2F2	NA	NA	NA	NA	NA

chr19	42595247	42595247	T	-	UTR3	POU2F2	NA	NA	NA	NA	NA
chr19	42728379	42728379	T	G	UTR5	ZNF526	NA	NA	NA	NA	NA
chr19	42730237	42730237	G	C	exonic	ZNF526	nonsynonymous SNV	ZNF526:NM_133444:exon3:c.G1682C:p.R561P	NA	NA	D
chr19	43697229	43697229	G	A	UTR3	PSG4	NA	NA	NA	rs8603	NA
chr19	44126738	44126738	G	A	UTR3	CADM4	NA	NA	NA	rs433226	NA
chr19	44127125	44127125	A	C	UTR3	CADM4	NA	NA	NA	NA	NA
chr19	44127129	44127129	T	C	UTR3	CADM4	NA	NA	NA	NA	NA
chr19	44127153	44127153	A	C	UTR3	CADM4	NA	NA	NA	NA	NA
chr19	44174249	44174249	C	T	exonic	PLAUR	synonymous SNV	:c.G24A:p.P8P,PLAUR:NM_001005377:exon1:c.G24A:p.P8P,PLAUR:NM	NA	rs62639324	NA
chr19	45001346	45001346	G	A	exonic	ZNF180	nonsynonymous SNV	ZNF180:NM_013256:exon2:c.C122T:p.A41V	NA	rs2571108	B
chr19	45004305	45004305	G	C	UTR5	ZNF180	NA	NA	NA	rs12977470	NA
chr19	45161174	45161174	C	A	exonic	PVR	synonymous SNV	_001135769:exon5:c.C987A:p.V329V,PVR:NM_001135770:exon5:c.C98	NA	NA	NA
chr19	45452694	45452694	-	T	ncRNA_exonic	APOC4-APOC2	NA	NA	NA	rs150448996	NA
chr19	45683494	45683494	T	-	UTR3	BLOC1S3	NA	NA	NA	rs60498449	NA
chr19	45806163	45806163	G	-	UTR3	MARK4	NA	NA	NA	rs34473699	NA
chr19	45911330	45911330	T	A	UTR3	ERCC1	NA	NA	NA	rs2013521	NA
chr19	45976591	45976591	A	G	UTR3	FOSB	NA	NA	NA	NA	NA
chr19	45976636	45976636	T	G	UTR3	FOSB	NA	NA	NA	rs10424098	NA
chr19	45977630	45977630	A	C	UTR3	FOSB	NA	NA	NA	NA	NA
chr19	46005564	46005564	T	A	UTR3	PPM1N	NA	NA	NA	rs73568912	NA
chr19	46093211	46093211	T	C	UTR3	GPR4	NA	NA	NA	rs1045217	NA
chr19	46214291	46214291	G	T	UTR3	FBXO46	NA	NA	NA	rs66535058	NA
chr19	46272195	46272195	A	C	UTR5	SIX5	NA	NA	NA	NA	NA
chr19	46286273	46286273	A	G	UTR3	DMWD	NA	NA	NA	NA	NA
chr19	46442775	46442775	G	A	UTR3	NOVA2	NA	NA	NA	NA	NA
chr19	46893943	46893943	A	G	UTR3	PPP5C	NA	NA	NA	NA	NA
chr19	46974652	46974652	G	C	UTR5	PNMAL1	NA	NA	NA	rs3826855	NA
chr19	46974678	46974678	C	G	UTR5	PNMAL1	NA	NA	NA	rs8103767	NA
chr19	47333843	47333843	A	T	ncRNA_exonic	SNAR-E	NA	NA	NA	rs59789527	NA
chr19	47724683	47724683	-	C	UTR3	BBC3	NA	NA	NA	NA	NA
chr19	47724692	47724692	T	C	UTR3	BBC3	NA	NA	NA	rs2862236	NA
chr19	47768128	47768128	G	C	exonic	CCDC9	nonsynonymous SNV	CCDC9:NM_015603:exon7:c.G645C:p.E215D	NA	rs2032811	NA
chr19	47824788	47824788	-	C	UTR3	C5AR1	NA	NA	NA	rs35042977	NA
chr19	48182787	48182787	C	T	exonic	GLTSCR1	synonymous SNV	GLTSCR1:NM_015711:exon6:c.C360T:p.A120A	NA	rs4802382	NA
chr19	48305566	48305566	C	T	exonic	TPRX1	synonymous SNV	TPRX1:NM_198479:exon2:c.G702A:p.P234P	COSM998808;OCCURENCE=1(endometr	rs12463317	NA
chr19	48305622	48305622	A	G	exonic	TPRX1	nonsynonymous SNV	TPRX1:NM_198479:exon2:c.T646C:p.S216P	NA	rs201007421	NA
chr19	48305634	48305634	A	G	exonic	TPRX1	nonsynonymous SNV	TPRX1:NM_198479:exon2:c.T634C:p.S212P	NA	rs201640420	NA
chr19	48305670	48305670	A	G	exonic	TPRX1	nonsynonymous SNV	TPRX1:NM_198479:exon2:c.T598C:p.S200P	NA	rs201483839	NA
chr19	48305694	48305694	A	G	exonic	TPRX1	nonsynonymous SNV	TPRX1:NM_198479:exon2:c.T574C:p.S192P	NA	rs200053895	NA
chr19	48343993	48343993	G	A	UTR3	CRX	NA	NA	NA	rs55835533	NA
chr19	48344829	48344830	TT	-	UTR3	CRX	NA	NA	NA	NA	NA
chr19	48374415	48374415	G	T	UTR3	SULT2A1	NA	NA	NA	rs296367	NA
chr19	48711375	48711375	-	T	UTR3	CARD8	NA	NA	NA	NA	NA
chr19	48964622	48964622	-	A	intronic	KCNJ14	NA	NA	NA	rs35350309	NA
chr19	48969339	48969339	T	-	UTR3	KCNJ14	NA	NA	NA	rs72206497	NA
chr19	48984059	48984059	C	G	UTR3	CYTH2	NA	NA	NA	NA	NA
chr19	49132198	49132198	A	C	exonic	SPHK2	nonsynonymous SNV	15C:p.Y172S,SPHK2:NM_001204158:exon7:c.A956C:p.Y319S,SPHK2:NI	NA	rs200347384	D
chr19	49132201	49132201	T	C	exonic	SPHK2	nonsynonymous SNV	18C:p.L173P,SPHK2:NM_001204158:exon7:c.T959C:p.L320P,SPHK2:NI	NA	rs201628054	D
chr19	49185082	49185082	A	-	ncRNA_exonic	SEC1P	NA	NA	NA	NA	NA
chr19	49207689	49207699	AAAAAAAA	-	UTR3	FUT2	NA	NA	NA	NA	NA
chr19	49209139	49209139	C	A	UTR3	FUT2	NA	NA	NA	NA	NA
chr19	49571148	49571148	-	TTTTC	UTR3	KCNA7	NA	NA	NA	rs113434270	NA
chr19	49571425	49571425	G	C	UTR3	KCNA7	NA	NA	NA	rs62127899	NA
chr19	51412666	51412666	C	A	exonic	KLK4	synonymous SNV	KLK4:NM_004917:exon2:c.G66T:p.S22S	NA	rs1654552	NA
chr19	51841417	51841417	C	T	exonic	VSIG10L	nonsynonymous SNV	VSIG10L:NM_001163922:exon6:c.G1775A:p.R592Q	NA	rs34380065	NA
chr19	51843808	51843808	C	T	exonic	VSIG10L	nonsynonymous SNV	VSIG10L:NM_001163922:exon3:c.G1068A:p.M356I	NA	rs7259266	NA
chr19	51850290	51850290	G	A	exonic	ETFB	nonsynonymous SNV	_001014763:exon4:c.C734T:p.T245M,ETFB:NM_001985:exon5:c.C461T	NA	rs1130426	P
chr19	52132668	52132668	T	C	exonic	SIGLEC5	nonsynonymous SNV	SIGLEC5:NM_003830:exon3:c.A643G:p.M215V	=COSM1129737;OCCURENCE=1(prostat	rs1807124	B
chr19	52197556	52197556	-	AGACTCAC	ncRNA_exonic	LINC00085	NA	NA	NA	rs150736418	NA
chr19	53381429	53381429	G	T	UTR3	ZNF320	NA	NA	NA	rs2162919	NA
chr19	53381495	53381507	TTTTTTTTT	-	UTR3	ZNF320	NA	NA	NA	rs71183850	NA
chr19	53431516	53431516	T	C	ncRNA_exonic	ZNF321P	NA	NA	NA	rs12984436	NA
chr19	53667387	53667387	T	-	UTR3	ZNF665	NA	NA	NA	rs35735454	NA
chr19	53761591	53761591	T	G	UTR5	VN1R2	NA	NA	NA	rs61576844	NA
chr19	53914545	53914545	-	T	UTR3	ZNF765	NA	NA	NA	NA	NA
chr19	53945959	53945959	T	C	ncRNA_exonic	TPM3P9	NA	NA	NA	rs113488663	NA

chr19	54378026	54378026	T	-	UTR3	MYADM	NA	NA	NA	rs67727940	NA
chr19	54378030	54378030	T	C	UTR3	MYADM	NA	NA	NA	rs200046918	NA
chr19	54378454	54378467	TGTGTGTG	-	UTR3	MYADM	NA	NA	NA	NA	NA
chr19	54693602	54693602	C	T	UTR5	MBOAT7	NA	NA	NA	NA	NA
chr19	54698196	54698198	GGA	-	UTR3	TSEN34	NA	NA	NA	rs146122151	NA
chr19	54724407	54724407	C	T	exonic	LILRB3	nonsynonymous SNV	01081450:exon6:c.G1249A:p.V417M,LILRB3:NM_006864:exon6:c.G12	NA	rs1132608	B
chr19	54724411	54724411	C	T	exonic	LILRB3	synonymous SNV	001081450:exon6:c.G1245A:p.E415E,LILRB3:NM_006864:exon6:c.G12	NA	rs1132607	NA
chr19	54724430	54724430	T	A	exonic	LILRB3	nonsynonymous SNV	001081450:exon6:c.A1226T:p.H409L,LILRB3:NM_006864:exon6:c.A12	NA	rs1132606	B
chr19	54725745	54725745	A	G	exonic	LILRB3	nonsynonymous SNV	_001081450:exon4:c.T613C:p.W205R,LILRB3:NM_006864:exon4:c.T61	NA	rs1052973	B
chr19	54725755	54725755	T	C	exonic	LILRB3	synonymous SNV	_001081450:exon4:c.A603G:p.T201T,LILRB3:NM_006864:exon4:c.A60	NA	rs1052971	NA
chr19	54725756	54725756	G	A	exonic	LILRB3	nonsynonymous SNV	M_001081450:exon4:c.C602T:p.T201I,LILRB3:NM_006864:exon4:c.C60	NA	rs1052970	B
chr19	54726870	54726870	C	G	UTR5	LILRB3	NA	NA	NA	rs117942579	NA
chr19	54745978	54745978	T	C	exonic	LILRA6	synonymous SNV	LILRA6:NM_024318:exon3:c.A279G:p.A93A	NA	rs1052964	NA
chr19	54745989	54745989	G	C	exonic	LILRA6	nonsynonymous SNV	LILRA6:NM_024318:exon3:c.C268G:p.Q90E	NA	rs1052963	NA
chr19	54948053	54948053	A	-	downstream	TTYH1	NA	NA	NA	NA	NA
chr19	54962467	54962467	G	A	UTR5	LENG8	NA	NA	NA	rs1035451	NA
chr19	54974167	54974167	A	C	exonic	LENG9	synonymous SNV	LENG9:NM_198988:exon1:c.T609G:p.G203G	NA	NA	NA
chr19	55241209	55241209	C	T	exonic	KIR3DL3	synonymous SNV	KIR3DL3:NM_153443:exon5:c.C906T:p.H302H	NA	rs34847288	NA
chr19	55247987	55247987	-	CTTTCCTC	UTR3	KIR3DL3	NA	NA	NA	NA	NA
chr19	55253465	55253465	C	G	exonic	KIR2DL3	nonsynonymous SNV	KIR2DL3:NM_015868:exon3:c.C110G:p.P37R	NA	rs201272358	NA
chr19	55253544	55253544	A	G	exonic	KIR2DL3	synonymous SNV	KIR2DL3:NM_015868:exon3:c.A189G:p.E63E	NA	rs34790392	NA
chr19	55253552	55253552	T	A	exonic	KIR2DL3	nonsynonymous SNV	KIR2DL3:NM_015868:exon3:c.T197A:p.F66Y	ID=COSM321259;OCCURENCE=1(lung)	rs673568	NA
chr19	55253601	55253601	G	A	exonic	KIR2DL3	synonymous SNV	KIR2DL3:NM_015868:exon3:c.G246A:p.K82K	NA	rs201337670	NA
chr19	55294454	55294454	C	T	exonic	KIR2DL1	nonsynonymous SNV	KIR2DL1:NM_014218:exon6:c.C796T:p.R266C	NA	rs151328241	NA
chr19	55330019	55330019	C	T	exonic	KIR3DL1	nonsynonymous SNV	KIR3DL1:NM_013289:exon3:c.C320T:p.S107L	NA	rs143159382	NA
chr19	55358681	55358681	T	A	exonic	KIR2DS4	unknown	UNKNOWN	NA	NA	NA
chr19	55359227	55359227	A	G	exonic	KIR2DS4	unknown	UNKNOWN	NA	rs199738147	NA
chr19	55359654	55359654	C	G	UTR3	KIR2DS4	NA	NA	NA	rs1743310	NA
chr19	55359891	55359891	A	G	UTR3	KIR2DS4	NA	NA	NA	NA	NA
chr19	55359921	55359921	-	TGTTTCAC	UTR3	KIR2DS4	NA	NA	NA	NA	NA
chr19	55359931	55359931	C	T	UTR3	KIR2DS4	NA	NA	NA	NA	NA
chr19	55359934	55359934	T	C	UTR3	KIR2DS4	NA	NA	NA	NA	NA
chr19	55359996	55359996	C	T	UTR3	KIR2DS4	NA	NA	NA	NA	NA
chr19	55401412	55401412	T	C	UTR3	FCAR	NA	NA	NA	rs59103589	NA
chr19	55401447	55401447	G	T	UTR3	FCAR	NA	NA	NA	rs58560391	NA
chr19	55401474	55401474	T	C	UTR3	FCAR	NA	NA	NA	rs60304316	NA
chr19	55401481	55401481	A	G	UTR3	FCAR	NA	NA	NA	rs59309328	NA
chr19	55424424	55424426	TTT	-	UTR3	NCR1	NA	NA	NA	rs200857637	NA
chr19	55435140	55435140	T	C	exonic	NLRP7	nonsynonymous SNV	p.T971A,NLRP7:NM_001127255:exon11:c.A3082G:p.T1028A,NLRP7:NI	NA	rs7256020	B
chr19	55441995	55441995	A	G	exonic	NLRP7	synonymous SNV	682C:p.Y894Y,NLRP7:NM_139176:exon9:c.T2598C:p.Y866Y,NLRP7:NM	NA	rs269951	NA
chr19	55715309	55715309	C	T	exonic	PTPRH	nonsynonymous SNV	PTPRH:NM_002842:exon5:c.G727A:p.V243I	NA	rs45535035	B
chr19	55715319	55715319	C	G	exonic	PTPRH	synonymous SNV	PTPRH:NM_002842:exon5:c.G717C:p.S239S	NA	rs9304763	NA
chr19	55873642	55873642	C	T	exonic	FAM71E2	nonsynonymous SNV	FAM71E2:NM_001145402:exon3:c.G535A:p.E179K	NA	rs4252574	NA
chr19	55987871	55987871	A	C	UTR5	ZNF628	NA	NA	NA	rs11667987	NA
chr19	56029616	56029616	-	CCA	exonic	SSC5D	nonframeshift insertion	SSC5D:NM_001144950:exon14:c.3973_3974insCCA:p.P1325delinsPT	NA	rs150781976	NA
chr19	56047448	56047448	A	G	exonic	SBK2	nonsynonymous SNV	SBK2:NM_001101401:exon2:c.T214C:p.C72R	NA	rs310453	B
chr19	56162776	56162776	C	T	exonic	CCDC106	synonymous SNV	CCDC106:NM_013301:exon5:c.C441T:p.S147S	NA	rs2287791	NA
chr19	56171893	56171893	G	C	exonic	U2AF2	nonsynonymous SNV	M_001012478:exon4:c.G242C:p.R81P,U2AF2:NM_007279:exon4:c.G24	NA	NA	B
chr19	56388364	56388364	T	A	exonic	NLRP4	stopgain SNV	NLRP4:NM_134444:exon8:c.T2528A:p.L843X	NA	NA	NA
chr19	56499769	56499769	A	G	UTR3	NLRP8	NA	NA	NA	rs76087581	NA
chr19	57019251	57019253	CTT	-	UTR5	ZNF471	NA	NA	NA	rs202156180	NA
chr19	57039314	57039314	A	G	UTR3	ZNF471	NA	NA	NA	rs960996	NA
chr19	57039791	57039791	A	-	UTR3	ZNF471	NA	NA	NA	rs5828685	NA
chr19	57050515	57050515	C	G	exonic	ZFP28	nonsynonymous SNV	ZFP28:NM_020828:exon1:c.C128G:p.A43G	NA	rs7258088	B
chr19	57079083	57079083	T	C	UTR5	ZNF470	NA	NA	NA	rs62124111	NA
chr19	57091966	57091966	-	TT	UTR3	ZNF470	NA	NA	NA	NA	NA
chr19	57135240	57135240	A	G	UTR3	ZNF71	NA	NA	NA	rs76311202	NA
chr19	57703077	57703077	T	G	UTR5	ZNF264	NA	NA	NA	rs2302058	NA
chr19	57733715	57733719	AAAAA	-	UTR3	ZNF264	NA	NA	NA	NA	NA
chr19	57841082	57841082	G	A	UTR3	ZNF543	NA	NA	NA	rs78764250	NA
chr19	57890360	57890360	T	C	UTR3	ZNF547	NA	NA	NA	rs8100747	NA
chr19	57922664	57922664	T	C	UTR5	ZNF17	NA	NA	NA	rs4801480	NA
chr19	58220579	58220579	-	C	UTR5	ZNF154	NA	NA	NA	rs112786452	NA
chr19	58231745	58231745	A	G	UTR3	ZNF671	NA	NA	NA	NA	NA
chr19	58638852	58638852	A	-	UTR3	ZNF329	NA	NA	NA	NA	NA
chr19	58790359	58790359	C	A	UTR5	ZNF8	NA	NA	NA	rs11666156	NA

chr19	58968875	58968875	A	C	UTR3	ZNF324B	NA	NA	NA	rs1077420	NA
chr19	59028629	59028629	T	G	exonic	ZBTB45	nonsynonymous SNV	ZBTB45:NM_032792:exon2:c.A412C:p.T138P	NA	NA	B
chr19	59085786	59085786	-	C	ncRNA_exonic	LOC100131691	NA	NA	NA	rs201939924	NA
chr19	59093604	59093604	A	-	ncRNA_exonic	MGC2752	NA	NA	NA	NA	NA
chr20	309162	309162	T	-	UTR3	SOX12	NA	NA	NA	rs5839854	NA
chr20	361316	361316	A	G	UTR5	TRIB3	NA	NA	NA	rs7261666	NA
chr20	361323	361323	T	C	UTR5	TRIB3	NA	NA	NA	rs7263534	NA
chr20	825318	825318	-	T	UTR5	FAM110A	NA	NA	NA	NA	NA
chr20	939130	939130	T	A	UTR3	RSPO4	NA	NA	NA	rs480012	NA
chr20	1286474	1286474	T	G	exonic	SNPH	nonsynonymous SNV	SNPH:NM_014723:exon6:c.T1261G:p.W421G	NA	NA	D
chr20	1551485	1551485	C	T	exonic	SIRPB1	synonymous SNV	SIRPB1:NM_006065:exon4:c.G1050A:p.A350A	NA	rs2254458	NA
chr20	1615959	1615959	T	C	exonic	SIRPG	synonymous SNV	SIRPG:NM_018556:exon4:c.A1035G:p.L345L	NA	rs9305125	NA
chr20	1895950	1895950	C	G	exonic	SIRPA	nonsynonymous SNV	285G:p.D95E,SIRPA:NM_001040022:exon3:c.C285G:p.D95E,SIRPA:NM	NA	rs1135200	B
chr20	1895951	1895951	C	T	exonic	SIRPA	nonsynonymous SNV	.C286T:p.L96F,SIRPA:NM_001040022:exon3:c.C286T:p.L96F,SIRPA:NM	NA	rs149634649	B
chr20	1895952	1895952	T	C	exonic	SIRPA	nonsynonymous SNV	T287C:p.L96P,SIRPA:NM_001040022:exon3:c.T287C:p.L96P,SIRPA:NM	NA	rs146163282	B
chr20	1895963	1895963	A	G	exonic	SIRPA	nonsynonymous SNV	8G:p.N100D,SIRPA:NM_001040022:exon3:c.A298G:p.N100D,SIRPA:NI	NA	rs17855613	B
chr20	1895965	1895965	C	A	exonic	SIRPA	nonsynonymous SNV	0A:p.N100K,SIRPA:NM_001040022:exon3:c.C300A:p.N100K,SIRPA:NN	NA	rs17855614	B
chr20	1895984	1895984	C	A	exonic	SIRPA	nonsynonymous SNV	19A:p.R107S,SIRPA:NM_001040022:exon3:c.C319A:p.R107S,SIRPA:NN	NA	rs17855615	P
chr20	1895990	1895990	G	A	exonic	SIRPA	nonsynonymous SNV	25A:p.G109S,SIRPA:NM_001040022:exon3:c.G325A:p.G109S,SIRPA:NN	NA	rs1135202	B
chr20	2945759	2945759	C	T	exonic	PTPRA	nonsynonymous SNV	26T:p.P109L,PTPRA:NM_080841:exon5:c.C326T:p.P109L,PTPRA:NM_0	=COSM149129;OCCURENCE=1(stomac	rs1178027	D
chr20	3089363	3089364	GA	-	ncRNA_UTR3	UBOX5	NA	NA	NA	rs142269465	NA
chr20	3204434	3204434	G	T	UTR3	ITPA	NA	NA	NA	NA	NA
chr20	3515924	3515924	G	A	exonic	ATRN	synonymous SNV	G87A:p.V29V,ATRN:NM_139321:exon2:c.G435A:p.V145V,ATRN:NM_1	NA	rs151518	NA
chr20	3515951	3515951	C	T	exonic	ATRN	synonymous SNV	.C114T:p.Y38Y,ATRN:NM_139321:exon2:c.C462T:p.Y154Y,ATRN:NM_1	NA	rs151519	NA
chr20	3564672	3564672	C	T	exonic	ATRN	synonymous SNV	544T:p.Y848Y,ATRN:NM_139321:exon17:c.C2892T:p.Y964Y,ATRN:NM	NA	rs235540	NA
chr20	3677736	3677736	T	G	exonic	SIGLEC1	synonymous SNV	SIGLEC1:NM_023068:exon9:c.A2376C:p.V792V	NA	rs673114	NA
chr20	3762095	3762095	T	C	UTR5	SPEF1	NA	NA	NA	rs2281479	NA
chr20	4666924	4666924	C	G	UTR5	PRNP	NA	NA	NA	rs13040327	NA
chr20	4708730	4708730	T	C	UTR3	PRND	NA	NA	NA	rs186333650	NA
chr20	5454400	5454400	A	T	ncRNA_exonic	LOC643406	NA	NA	NA	rs56059321	NA
chr20	5481480	5481480	C	T	ncRNA_exonic	LINC00654	NA	NA	NA	NA	NA
chr20	5482058	5482058	C	T	ncRNA_exonic	LINC00654	NA	NA	NA	rs201872341	NA
chr20	5482103	5482103	T	C	ncRNA_exonic	LINC00654	NA	NA	NA	rs200323153	NA
chr20	5482155	5482155	T	C	ncRNA_exonic	LINC00654	NA	NA	NA	rs113305726	NA
chr20	6104068	6104068	C	T	UTR5	FERMT1	NA	NA	NA	rs2295433	NA
chr20	10037110	10037110	-	GATA	ncRNA_UTR3	ANKEF1	NA	NA	NA	rs5840352	NA
chr20	10654563	10654563	A	C	UTR5	JAG1	NA	NA	NA	rs1051412	NA
chr20	13695607	13695607	T	G	exonic	ESF1	nonsynonymous SNV	01276380:exon14:c.A2470C:p.I824L,ESF1:NM_016649:exon14:c.A247	NA	rs34414644	B
chr20	13747441	13747441	A	G	exonic	ESF1	nonsynonymous SNV	_001276380:exon8:c.T1649C:p.I550T,ESF1:NM_016649:exon8:c.T1649	NA	rs3180370	B
chr20	17932210	17932210	C	T	exonic	SNX5	synonymous SNV	_014426:exon6:c.G543A:p.E181E,SNX5:NM_152227:exon7:c.G543A:	NA	rs2273448	NA
chr20	17971053	17971053	-	G	UTR3	MGME1	NA	NA	NA	NA	NA
chr20	19982007	19982011	TTTTT	-	UTR3	RIN2	NA	NA	NA	NA	NA
chr20	22542568	22542575	GAAAGAAA	-	ncRNA_exonic	LINC00261	NA	NA	NA	NA	NA
chr20	23966378	23966378	A	G	exonic	GGTLC1	nonsynonymous SNV	_178311:exon5:c.T457C:p.W153R,GGTLC1:NM_178312:exon5:c.T457	NA	rs3954815	B
chr20	23966384	23966384	C	T	exonic	GGTLC1	nonsynonymous SNV	_178311:exon5:c.G451A:p.V151M,GGTLC1:NM_178312:exon5:c.G45	NA	rs140956367	B
chr20	25124088	25124088	G	A	ncRNA_exonic	LOC284798	NA	NA	NA	rs6050363	NA
chr20	25754574	25754581	ATATATAT	-	ncRNA_exonic	FAM182B	NA	NA	NA	NA	NA
chr20	25755940	25755940	G	T	ncRNA_exonic	FAM182B	NA	NA	NA	rs79936808	NA
chr20	26061803	26061803	C	A	ncRNA_exonic	FAM182A	NA	NA	NA	rs78281752	NA
chr20	26063547	26063547	G	T	ncRNA_exonic	FAM182A	NA	NA	NA	NA	NA
chr20	26063560	26063560	G	A	ncRNA_exonic	FAM182A	NA	NA	NA	rs5742261	NA
chr20	26063577	26063577	C	T	ncRNA_exonic	FAM182A	NA	NA	NA	rs201158839	NA
chr20	26063579	26063579	C	A	ncRNA_exonic	FAM182A	NA	NA	NA	rs201752002	NA
chr20	26063612	26063612	C	T	ncRNA_exonic	FAM182A	NA	NA	NA	NA	NA
chr20	26063665	26063665	G	A	ncRNA_exonic	FAM182A	NA	NA	NA	NA	NA
chr20	26063726	26063726	T	C	ncRNA_exonic	FAM182A	NA	NA	NA	rs199551690	NA
chr20	26063729	26063729	G	T	ncRNA_exonic	FAM182A	NA	NA	NA	NA	NA
chr20	26063752	26063752	T	C	ncRNA_exonic	FAM182A	NA	NA	NA	rs78544920	NA
chr20	26063805	26063805	A	G	ncRNA_exonic	FAM182A	NA	NA	NA	rs78113344	NA
chr20	26064303	26064303	G	C	ncRNA_exonic	FAM182A	NA	NA	NA	rs200946927	NA
chr20	26064305	26064305	A	G	ncRNA_exonic	FAM182A	NA	NA	NA	rs201971148	NA
chr20	26064328	26064328	G	T	ncRNA_exonic	FAM182A	NA	NA	NA	rs140014678	NA
chr20	26064330	26064330	C	T	ncRNA_exonic	FAM182A	NA	NA	NA	rs151022794	NA
chr20	26064408	26064408	T	A	ncRNA_exonic	FAM182A	NA	NA	NA	NA	NA
chr20	29623156	29623156	G	A	ncRNA_exonic	FRG1B	NA	NA	NA	rs62198335	NA
chr20	29625984	29625984	T	C	ncRNA_exonic	FRG1B	NA	NA	NA	rs80066412	NA

chr20	29631613	29631613	T	C	ncRNA_exonic	FRG1B	NA	NA	NA	rs199635479	NA
chr20	29631614	29631614	G	A	ncRNA_exonic	FRG1B	NA	NA	NA	rs200793872	NA
chr20	29632638	29632638	C	A	ncRNA_exonic	FRG1B	NA	NA	NA	rs9647043	NA
chr20	29632674	29632674	-	A	ncRNA_exonic	FRG1B	NA	NA	NA	rs138867112	NA
chr20	29633915	29633915	C	T	ncRNA_exonic	FRG1B	NA	NA	NA	rs4066495	NA
chr20	30433228	30433228	T	G	exonic	FOXS1	nonsynonymous SNV	FOXS1:NM_004118:exon1:c.A118C:p.T40P	NA	NA	D
chr20	31505189	31505189	C	T	intergenic	RE1(dist=66978),SUN5(dist=6	NA	NA	NA	rs13043425	NA
chr20	31627291	31627291	A	G	exonic	BPIFB6	nonsynonymous SNV	BPIFB6:NM_174897:exon10:c.A1039G:p.S347G	NA	rs4911287	B
chr20	31656632	31656632	C	G	exonic	BPIFB3	nonsynonymous SNV	BPIFB3:NM_182658:exon10:c.C1002G:p.H334Q	NA	rs6057717	P
chr20	31676804	31676804	A	C	exonic	BPIFB4	nonsynonymous SNV	BPIFB4:NM_182519:exon6:c.A959C:p.N320T	NA	rs2889732	D
chr20	31790318	31790318	G	T	ncRNA_exonic	BPIFA4P	NA	NA	NA	NA	NA
chr20	32378915	32378915	C	T	exonic	ZNF341	synonymous SNV	ZNF341:NM_032819:exon15:c.C2136T:p.G712G	NA	NA	NA
chr20	33470694	33470694	C	T	exonic	ACSS2	synonymous SNV	VI_001076552:exon2:c.C276T:p.F92F,ACSS2:NM_018677:exon2:c.C27	NA	rs4911163	NA
chr20	33872109	33872109	A	C	exonic	EIF6	nonsynonymous SNV	EIF6:NM_181466:exon2:c.T182G:p.V61G	NA	NA	NA
chr20	33873827	33873827	T	-	UTR3	FAM83C	NA	NA	NA	rs11346452	NA
chr20	34633828	34633828	A	-	ncRNA_exonic	LINC00657	NA	NA	NA	NA	NA
chr20	35740794	35740794	T	C	exonic	MROH8	unknown	UNKNOWN	NA	rs1744760	NA
chr20	35807790	35807790	-	;GGCCCCGC	exonic	MROH8	unknown	UNKNOWN	NA	NA	NA
chr20	36033692	36033692	A	C	UTR3	SRC	NA	NA	NA	NA	NA
chr20	36033745	36033745	C	G	UTR3	SRC	NA	NA	NA	NA	NA
chr20	42170327	42170327	C	T	UTR3	L3MBTL1	NA	NA	NA	rs1062943	NA
chr20	43707209	43707209	T	-	UTR3	STK4	NA	NA	NA	rs34932056	NA
chr20	43933163	43933163	T	C	exonic	MATN4	synonymous SNV	8G:p.A116A,MATN4:NM_030590:exon3:c.A348G:p.A116A,MATN4:NM	NA	rs2233094	NA
chr20	43954939	43954939	A	-	UTR3	SDC4	NA	NA	NA	rs3091706	NA
chr20	44141202	44141207	CACACA	-	UTR3	SPINT3	NA	NA	NA	rs201209920	NA
chr20	44512996	44512996	C	T	UTR3	ZSWIM1	NA	NA	NA	NA	NA
chr20	46284869	46284869	-	T	UTR3	NCOA3	NA	NA	NA	NA	NA
chr20	46998794	46998794	-	A	ncRNA_exonic	LINC00494	NA	NA	NA	NA	NA
chr20	48570261	48570261	A	G	UTR3	RNF114	NA	NA	NA	rs141239729	NA
chr20	48604985	48604985	A	C	UTR3	SNAI1	NA	NA	NA	rs1063211	NA
chr20	50214746	50214746	T	G	UTR3	ATP9A	NA	NA	NA	NA	NA
chr20	50768400	50768400	A	C	UTR3	ZFP64	NA	NA	NA	NA	NA
chr20	52184384	52184384	T	A	UTR3	ZNF217	NA	NA	NA	rs718889	NA
chr20	52560856	52560857	TT	-	UTR3	BCAS1	NA	NA	NA	NA	NA
chr20	52836074	52836074	T	C	UTR3	PFDN4	NA	NA	NA	rs6023044	NA
chr20	55940426	55940426	G	A	exonic	RAE1	synonymous SNV	_001015885:exon5:c.G303A:p.V101V,RAE1:NM_003610:exon5:c.G303	NA	rs6099582	NA
chr20	56141218	56141219	TG	-	UTR3	PCK1	NA	NA	NA	rs59710283	NA
chr20	56141291	56141291	T	C	UTR3	PCK1	NA	NA	NA	rs28359554	NA
chr20	57253695	57253695	-	AA	ncRNA_UTR3	STX16	NA	NA	NA	NA	NA
chr20	57415823	57415823	C	A	exonic	GNAS	nonsynonymous SNV	GNAS:NM_016592:exon1:c.C662A:p.P221Q	NA	NA	D
chr20	57429696	57429696	C	G	exonic	GNAS	nonsynonymous SNV	001077490:exon1:c.C1189G:p.L397V,GNAS:NM_080425:exon1:c.C137	NA	rs148033592	NA
chr20	57429715	57429715	A	C	exonic	GNAS	nonsynonymous SNV	GNAS:NM_001077490:exon1:c.A1208C:p.Q403P	NA	rs56213454	NA
chr20	57429718	57429718	T	C	exonic	GNAS	nonsynonymous SNV	GNAS:NM_001077490:exon1:c.T1211C:p.M404T	NA	NA	NA
chr20	57429719	57429719	G	T	exonic	GNAS	nonsynonymous SNV	001077490:exon1:c.G1212T:p.M404I,GNAS:NM_080425:exon1:c.G13	NA	rs56371919	NA
chr20	57571763	57571763	A	G	exonic	CTSZ	synonymous SNV	CTSZ:NM_001336:exon5:c.T732C:p.S244S	NA	rs9760	NA
chr20	57609945	57609945	C	A	ncRNA_UTR3	SLMO2	NA	NA	NA	NA	NA
chr20	57609945	57609945	-	A	ncRNA_UTR3	SLMO2	NA	NA	NA	NA	NA
chr20	58442826	58442826	C	A	exonic	SYCP2	synonymous SNV	SYCP2:NM_014258:exon38:c.G4065T:p.G1355G	NA	rs6070981	NA
chr20	60709531	60709531	T	-	UTR3	LSM14B	NA	NA	NA	rs11477488	NA
chr20	60897487	60897487	C	T	exonic	LAMA5	nonsynonymous SNV	LAMA5:NM_005560:exon47:c.G6184A:p.D2062N	NA	rs2274934	P
chr20	60908279	60908279	A	G	exonic	LAMA5	nonsynonymous SNV	LAMA5:NM_005560:exon26:c.T3149C:p.L1050P	NA	NA	D
chr20	60908282	60908282	T	G	exonic	LAMA5	nonsynonymous SNV	LAMA5:NM_005560:exon26:c.A3146C:p.H1049P	NA	NA	D
chr20	61040453	61040453	C	G	exonic	GATA5	synonymous SNV	GATA5:NM_080473:exon6:c.G981C:p.S327S	NA	rs6061243	NA
chr20	61431557	61431557	A	C	UTR3	MRGBP	NA	NA	NA	rs7397	NA
chr20	61732917	61732917	-	CTCC	ncRNA_exonic	HAR1A	NA	NA	NA	rs111489452	NA
chr20	61733058	61733058	C	T	ncRNA_exonic	HAR1A	NA	NA	NA	rs199531153	NA
chr20	61733262	61733262	C	A	ncRNA_exonic	HAR1A	NA	NA	NA	rs6122371	NA
chr20	61888814	61888814	G	C	ncRNA_exonic	FLJ16779	NA	NA	NA	NA	NA
chr20	62037879	62037879	A	C	UTR3	KCNQ2	NA	NA	NA	NA	NA
chr20	62037884	62037884	T	C	UTR3	KCNQ2	NA	NA	NA	NA	NA
chr20	62038277	62038277	T	G	exonic	KCNQ2	nonsynonymous SNV	_172106:exon16:c.A2285C:p.N762T,KCNQ2:NM_172108:exon16:c.A22	NA	rs1801475	B
chr20	62370295	62370295	T	C	UTR3	LIME1	NA	NA	NA	NA	NA
chr20	62378121	62378121	C	T	UTR3	ZBTB46	NA	NA	NA	rs35982738	NA
chr20	62591140	62591140	T	G	UTR3	ZNF512B	NA	NA	NA	NA	NA
chr21	9907345	9907345	C	T	ncRNA_exonic	TEKT4P2	NA	NA	NA	rs3694	NA
chr21	9968544	9968544	C	G	ncRNA_exonic	TEKT4P2	NA	NA	NA	rs14244	NA

chr21	14424534	14424534	A	C	ncRNA_exonic	ANKRD30BP2	NA	NA	NA	NA	NA
chr21	14439197	14439197	G	C	ncRNA_exonic	ANKRD30BP2	NA	NA	NA	rs184573568	NA
chr21	15558346	15558346	G	A	exonic	LIPI	synonymous SNV	LIPI:NM_198996:exon3:c.C540T:p.S180S	NA	rs397517	NA
chr21	15646405	15646405	C	A	ncRNA_exonic	ABCC13	NA	NA	NA	rs2236001	NA
chr21	26969703	26969703	T	C	exonic	MRPL39	synonymous SNV	M_017446:exon6:c.A612G:p.K204K,MRPL39:NM_080794:exon6:c.A61	NA	rs1135618	NA
chr21	27253248	27253248	T	C	UTR3	APP	NA	NA	NA	NA	NA
chr21	27253253	27253253	A	C	UTR3	APP	NA	NA	NA	NA	NA
chr21	27253262	27253262	G	C	UTR3	APP	NA	NA	NA	NA	NA
chr21	28338997	28338997	T	G	UTR5	ADAMTS5	NA	NA	NA	NA	NA
chr21	29912452	29912452	C	A	ncRNA_exonic	LINC00161	NA	NA	NA	rs2150392	NA
chr21	31964885	31964885	T	G	exonic	KRTAP6-3	nonsynonymous SNV	KRTAP6-3:NM_181605:exon1:c.T121G:p.C41G	NA	NA	NA
chr21	33043422	33043422	A	-	UTR3	SCAF4	NA	NA	NA	rs71772238	NA
chr21	34164046	34164046	A	-	ncRNA_UTR3	C21orf62	NA	NA	NA	NA	NA
chr21	34443831	34443831	T	G	UTR3	OLIG1	NA	NA	NA	NA	NA
chr21	34669259	34669259	A	-	UTR3	IL10RB	NA	NA	NA	NA	NA
chr21	34923934	34923934	T	C	exonic	SON	synonymous SNV	M_032195:exon3:c.T2397C:p.S799S,SON:NM_138927:exon3:c.T2397C:	30344,COSM1030343;OCCURENCE=2(er	rs200724919	NA
chr21	35237608	35237608	T	C	exonic	ITSN1	synonymous SNV	ITSN1:NM_003024:exon32:c.T4044C:p.D1348D	NA	rs9976801	NA
chr21	35561665	35561665	A	G	ncRNA_exonic	LINC00310	NA	NA	NA	rs6517211	NA
chr21	35736384	35736384	G	A	UTR5	KCNE2	NA	NA	NA	rs41260744	NA
chr21	36089160	36089160	-	CACACA	UTR3	CLIC6	NA	NA	NA	NA	NA
chr21	37518850	37518850	G	A	ncRNA_UTR3	CBR3	NA	NA	NA	rs34911362	NA
chr21	38121746	38121750	TGACT	-	UTR3	SIM2	NA	NA	NA	rs113168269	NA
chr21	38121750	38121750	T	-	UTR3	SIM2	NA	NA	NA	rs61018468	NA
chr21	38885228	38885229	TT	-	UTR3	DYRK1A	NA	NA	NA	NA	NA
chr21	40557441	40557442	CT	-	UTR3	BRWD1	NA	NA	NA	rs202009663	NA
chr21	42551109	42551109	T	G	exonic	PLAC4	synonymous SNV	PLAC4:NM_182832:exon1:c.A447C:p.S149S	NA	NA	NA
chr21	43131734	43131734	T	A	ncRNA_exonic	LINC00479	NA	NA	NA	rs2838094	NA
chr21	43306769	43306769	T	-	UTR3	C2CD2	NA	NA	NA	rs11347156	NA
chr21	44438244	44438244	T	G	exonic	PKNOX1	synonymous SNV	PKNOX1:NM_004571:exon7:c.T624G:p.G208G	NA	NA	NA
chr21	44451935	44451935	-	A	UTR3	PKNOX1	NA	NA	NA	rs138842830	NA
chr21	44838948	44838948	T	G	exonic	SIK1	nonsynonymous SNV	SIK1:NM_173354:exon11:c.A1415C:p.H472P	NA	NA	NA
chr21	45177868	45177868	T	-	UTR3	PDXK	NA	NA	NA	NA	NA
chr21	45230317	45230317	T	C	ncRNA_exonic	LOC284837	NA	NA	NA	NA	NA
chr21	45230318	45230318	G	C	ncRNA_exonic	LOC284837	NA	NA	NA	NA	NA
chr21	45230345	45230345	G	C	ncRNA_exonic	LOC284837	NA	NA	NA	NA	NA
chr21	45538647	45538647	T	C	exonic	PWP2	synonymous SNV	PWP2:NM_005049:exon9:c.T984C:p.I328I	NA	rs756553	NA
chr21	45647837	45647853	ACCACCCC	-	UTR3	ICOSLG	NA	NA	NA	rs67226187	NA
chr21	45732175	45732175	A	G	exonic	PFKL	nonsynonymous SNV	PFKL:NM_002626:exon4:c.A425G:p.E142G	NA	NA	B
chr21	45811294	45811294	A	C	exonic	TRPM2	nonsynonymous SNV	TRPM2:NM_003307:exon11:c.A1580C:p.D527A	NA	NA	B
chr21	46058252	46058252	C	A	UTR3	KRTAP10-10	NA	NA	NA	rs142579519	NA
chr21	46058255	46058255	T	A	UTR3	KRTAP10-10	NA	NA	NA	rs117595201	NA
chr21	46058257	46058257	G	A	UTR3	KRTAP10-10	NA	NA	NA	rs112728315	NA
chr21	47603591	47603591	A	G	UTR5	SPATC1L	NA	NA	NA	NA	NA
chr21	47603676	47603676	C	G	UTR5	SPATC1L	NA	NA	NA	NA	NA
chr21	47670633	47670633	C	G	ncRNA_exonic	MCM3AP-AS1	NA	NA	NA	rs4818829	NA
chr21	47671275	47671275	-	AAT	ncRNA_exonic	MCM3AP-AS1	NA	NA	NA	rs17176758	NA
chr21	47855876	47855876	A	G	exonic	PCNT	synonymous SNV	PCNT:NM_006031:exon39:c.A8811G:p.T2937T	NA	rs17371795	NA
chr22	17469049	17469049	C	A	exonic	GAB4	stopgain SNV	GAB4:NM_001037814:exon3:c.G487T:p.G163X	NA	rs28502153	NA
chr22	18300879	18300879	G	A	exonic	MICAL3	synonymous SNV	MICAL3:NM_015241:exon26:c.C4548T:p.S1516S	NA	rs11704809	NA
chr22	18519998	18519998	T	C	ncRNA_exonic	FLJ41941	NA	NA	NA	rs465508	NA
chr22	18520582	18520582	G	A	ncRNA_exonic	FLJ41941	NA	NA	NA	rs975826	NA
chr22	18775061	18775061	G	A	ncRNA_exonic	GGT3P	NA	NA	NA	rs189425726	NA
chr22	18779428	18779428	A	G	ncRNA_exonic	GGT3P	NA	NA	NA	rs111623219	NA
chr22	19025396	19025396	T	G	UTR3	DGCR2	NA	NA	NA	NA	NA
chr22	19025446	19025446	T	C	UTR3	DGCR2	NA	NA	NA	NA	NA
chr22	19511925	19511925	G	A	exonic	CLDN5	stopgain SNV	M_001130861:exon1:c.C109T:p.Q37X,CLDN5:NM_003277:exon2:c.C10	NA	rs885985	NA
chr22	20098887	20098887	C	T	UTR3	DGCR8	NA	NA	NA	NA	NA
chr22	20387276	20387276	A	G	ncRNA_exonic	PI4KAP1	NA	NA	NA	rs2629350	NA
chr22	20390444	20390444	A	G	ncRNA_exonic	PI4KAP1	NA	NA	NA	rs1892850	NA
chr22	20394526	20394526	C	T	ncRNA_exonic	PI4KAP1	NA	NA	NA	rs201289136	NA
chr22	21242600	21242603	CACA	-	UTR3	SNAP29	NA	NA	NA	rs34474453	NA
chr22	21244940	21244940	A	G	UTR3	SNAP29	NA	NA	NA	rs165739	NA
chr22	21327589	21327589	C	T	exonic	AIFM3	nonsynonymous SNV	AIFM3:NM_001146288:exon3:c.C43T:p.P15S	NA	rs178264	NA
chr22	21369454	21369454	A	G	UTR5	P2RX6	NA	NA	NA	rs71314758	NA
chr22	21638109	21638109	A	G	ncRNA_exonic	POM121L8P	NA	NA	NA	rs199883726	NA
chr22	21641886	21641886	G	A	ncRNA_exonic	POM121L8P	NA	NA	NA	rs60992581	NA

chr22	21649456	21649456	G	A	ncRNA_exonic	POM121L8P	NA	NA	NA	rs111255493	NA
chr22	21649845	21649845	A	-	ncRNA_exonic	POM121L8P	NA	NA	NA	NA	NA
chr22	21650235	21650235	G	A	ncRNA_exonic	POM121L8P	NA	NA	NA	rs478288	NA
chr22	21650770	21650770	C	T	ncRNA_exonic	POM121L8P	NA	NA	NA	rs680955	NA
chr22	21802007	21802007	T	G	UTR3	HIC2	NA	NA	NA	NA	NA
chr22	21802070	21802070	-	GT	UTR3	HIC2	NA	NA	NA	rs142556171	NA
chr22	22054753	22054753	A	C	UTR3	YPEL1	NA	NA	NA	NA	NA
chr22	22054764	22054764	-	T	UTR3	YPEL1	NA	NA	NA	NA	NA
chr22	23733547	23733547	T	-	ncRNA_exonic	ZDHHC8P1	NA	NA	NA	rs11326923	NA
chr22	23736180	23736180	C	T	ncRNA_exonic	ZDHHC8P1	NA	NA	NA	rs5759766	NA
chr22	24084365	24084365	G	T	UTR3	ZNF70	NA	NA	NA	NA	NA
chr22	24314006	24314006	G	T	UTR3	DDTL	NA	NA	NA	rs1006771	NA
chr22	24314132	24314132	T	C	UTR3	DDTL	NA	NA	NA	NA	NA
chr22	24314182	24314182	A	G	UTR3	DDTL	NA	NA	NA	NA	NA
chr22	24657678	24657678	T	C	ncRNA_exonic	POM121L9P	NA	NA	NA	NA	NA
chr22	24657714	24657714	C	G	ncRNA_exonic	POM121L9P	NA	NA	NA	rs80002636	NA
chr22	24660060	24660060	C	A	ncRNA_exonic	POM121L9P	NA	NA	NA	NA	NA
chr22	24981608	24981608	T	G	UTR3	FAM211B	NA	NA	NA	NA	NA
chr22	24981657	24981657	A	C	UTR3	FAM211B	NA	NA	NA	NA	NA
chr22	25016442	25016442	C	T	exonic	GGT1	nonsynonymous SNV	M_001032365:exon8:c.C530T:p.A177V,GGT1:NM_005265:exon8:c.C53	=COSM1130481;OCCURENCE=1(prostat	rs3895576	B
chr22	25023441	25023441	C	T	exonic	GGT1	nonsynonymous SNV	001032365:exon12:c.C1063T:p.R355W,GGT1:NM_005265:exon12:c.C1	NA	rs200419006	B
chr22	25023459	25023459	G	A	exonic	GGT1	nonsynonymous SNV	001032365:exon12:c.G1081A:p.D361N,GGT1:NM_005265:exon12:c.G1	NA	rs138813205	B
chr22	25041202	25041202	T	A	ncRNA_exonic	POM121L10P	NA	NA	NA	rs1055003	NA
chr22	25042640	25042640	G	T	ncRNA_exonic	POM121L10P	NA	NA	NA	rs62231246	NA
chr22	25043061	25043061	-	CCGG	ncRNA_exonic	POM121L10P	NA	NA	NA	NA	NA
chr22	25603245	25603245	C	G	UTR3	CRYBB3	NA	NA	NA	rs79515519	NA
chr22	26120084	26120084	-	AC	UTR3	ADRBK2	NA	NA	NA	NA	NA
chr22	26122880	26122881	GT	-	UTR3	ADRBK2	NA	NA	NA	NA	NA
chr22	26839586	26839586	A	-	UTR3	ASPHD2	NA	NA	NA	NA	NA
chr22	26840194	26840194	T	-	UTR3	ASPHD2	NA	NA	NA	NA	NA
chr22	26847961	26847972	CGCGCGCG	-	UTR3	HPS4	NA	NA	NA	NA	NA
chr22	27066566	27066569	AAAA	-	ncRNA_exonic	MIAT	NA	NA	NA	NA	NA
chr22	27067794	27067794	A	-	ncRNA_exonic	MIAT	NA	NA	NA	NA	NA
chr22	28146770	28146770	A	G	UTR3	MN1	NA	NA	NA	rs28501997	NA
chr22	29754944	29754944	T	G	exonic	AP1B1	nonsynonymous SNV	96C:p.N99T,AP1B1:NM_001166019:exon5:c.A296C:p.N99T,AP1B1:NM_	NA	NA	D
chr22	30092040	30092040	A	C	UTR3	NF2	NA	NA	NA	NA	NA
chr22	30092785	30092785	T	-	UTR3	NF2	NA	NA	NA	NA	NA
chr22	30093556	30093556	T	-	UTR3	NF2	NA	NA	NA	rs113760386	NA
chr22	30422037	30422037	-	T	UTR3	MTMR3	NA	NA	NA	NA	NA
chr22	30951320	30951320	T	G	exonic	GAL3ST1	nonsynonymous SNV	GAL3ST1:NM_004861:exon4:c.A892C:p.T298P	NA	NA	D
chr22	31043406	31043406	T	G	UTR3	SLC35E4	NA	NA	NA	NA	NA
chr22	31303442	31303442	C	A	UTR3	OSBP2	NA	NA	NA	NA	NA
chr22	31322642	31322642	G	A	UTR3	MORC2	NA	NA	NA	NA	NA
chr22	32875190	32875190	G	A	exonic	FBXO7	nonsynonymous SNV	G108A:p.M36I,FBXO7:NM_001257990:exon2:c.G3A:p.M1I,FBXO7:NM_	NA	rs11107	B
chr22	33256901	33256902	TT	-	UTR3	TIMP3	NA	NA	NA	rs60978089	NA
chr22	36139978	36139978	T	-	UTR3	RBFOX2	NA	NA	NA	rs71658610	NA
chr22	36623362	36623362	A	-	UTR3	APOL2	NA	NA	NA	NA	NA
chr22	37769479	37769479	A	C	exonic	ELFN2	nonsynonymous SNV	ELFN2:NM_052906:exon3:c.T2096G:p.V699G	NA	NA	D
chr22	37769491	37769491	T	G	exonic	ELFN2	nonsynonymous SNV	ELFN2:NM_052906:exon3:c.A2084C:p.H695P	NA	NA	D
chr22	37962312	37962312	G	C	UTR5	CDC42EP1	NA	NA	NA	NA	NA
chr22	38120176	38120178	CCT	-	exonic	TRIOBP	nonframeshift deletion	TRIOBP:NM_001039141:exon7:c.1613_1615del:p.538_539del	ID=COSM111672;OCCURENCE=1(ovary)	rs146565844	NA
chr22	38121740	38121740	A	C	exonic	TRIOBP	synonymous SNV	TRIOBP:NM_001039141:exon7:c.A3177C:p.I1059I	NA	NA	NA
chr22	38170309	38170309	A	C	UTR3	TRIOBP	NA	NA	NA	NA	NA
chr22	38170313	38170313	T	C	UTR3	TRIOBP	NA	NA	NA	NA	NA
chr22	38482353	38482394	GCCATCCCA	-	exonic	BAIAP2L2	nonframeshift deletion	BAIAP2L2:NM_025045:exon12:c.1322_1363del:p.441_455del	NA	NA	NA
chr22	38687872	38687872	G	T	UTR3	CSNK1E	NA	NA	NA	NA	NA
chr22	38881822	38881822	-	A	UTR3	DDX17	NA	NA	NA	rs35742916	NA
chr22	39214774	39214774	G	T	UTR3	NPTXR	NA	NA	NA	rs7288826	NA
chr22	39428595	39428596	TT	-	UTR3	APOBEC3D	NA	NA	NA	NA	NA
chr22	39428750	39428750	A	C	UTR3	APOBEC3D	NA	NA	NA	NA	NA
chr22	40719728	40719728	T	G	UTR3	TNRC6B	NA	NA	NA	NA	NA
chr22	40720294	40720294	-	T	UTR3	TNRC6B	NA	NA	NA	NA	NA
chr22	40727650	40727652	TTT	-	UTR3	TNRC6B	NA	NA	NA	NA	NA
chr22	41855923	41855923	A	C	UTR3	PHF5A	NA	NA	NA	rs61211718	NA
chr22	42470317	42470317	T	C	UTR5	FAM109B	NA	NA	NA	rs13057094	NA
chr22	42524323	42524323	A	G	exonic	CYP2D6	synonymous SNV	_001025161:exon4:c.T543C:p.H181H,CYP2D6:NM_000106:exon5:c.T6	NA	rs150518553	NA

chr22	44276171	44276171	T	G	UTR3	PNPLA5	NA	NA	NA	rs470093	NA
chr22	44708655	44708655	T	C	UTR5	KIAA1644	NA	NA	NA	rs7510924	NA
chr22	45583065	45583065	A	-	UTR3	NUP50	NA	NA	NA	rs201638442	NA
chr22	45592689	45592689	T	G	UTR3	KIAA0930	NA	NA	NA	NA	NA
chr22	45996713	45996713	T	-	UTR3	FBLN1	NA	NA	NA	NA	NA
chr22	46436144	46436144	T	-	ncRNA_exonic	LOC100271722	NA	NA	NA	NA	NA
chr22	46633508	46633508	A	-	UTR3	PPARA	NA	NA	NA	NA	NA
chr22	46638629	46638630	CA	-	UTR3	PPARA	NA	NA	NA	NA	NA
chr22	50659471	50659471	A	G	exonic	TUBGCP6	nonsynonymous SNV	TUBGCP6:NM_020461:exon16:c.T3317C:p.I1106T	NA	NA	B
chr22	50882753	50882753	T	-	UTR3	PPP6R2	NA	NA	NA	rs59017944	NA
chr22	50884801	50884801	G	-	UTR3	SBF1	NA	NA	NA	NA	NA
chr22	50884814	50884814	A	C	UTR3	SBF1	NA	NA	NA	NA	NA
chr22	50969668	50969668	T	A	exonic	ODF3B	nonsynonymous SNV	ODF3B:NM_001014440:exon4:c.A370T:p.T124S	NA	NA	B
chr22	50969670	50969670	T	A	exonic	ODF3B	nonsynonymous SNV	ODF3B:NM_001014440:exon4:c.A368T:p.H123L	NA	NA	D
chr22	51020668	51020668	C	A	ncRNA_intronic	CHKB-CPT1B	NA	NA	NA	rs86337	NA
chrX	2822945	2822945	T	-	UTR3	ARSD	NA	NA	NA	rs60974016	NA
chrX	2824178	2824178	T	-	UTR3	ARSD	NA	NA	NA	rs71883506	NA
chrX	3523054	3523055	AC	-	UTR3	PRKX	NA	NA	NA	NA	NA
chrX	6145855	6145855	C	G	UTR5	NLGN4X	NA	NA	NA	rs2290488	NA
chrX	7023835	7023835	G	A	exonic	HDHD1	nonsynonymous SNV	M_001178136:exon2:c.C106T:p.R36C,HDHD1:NM_012080:exon2:c.C10	NA	NA	NA
chrX	7867727	7867727	T	C	UTR3	PNPLA4	NA	NA	NA	rs191932908	NA
chrX	7867732	7867732	A	G	UTR3	PNPLA4	NA	NA	NA	rs6639976	NA
chrX	8433461	8433461	T	C	UTR5	VCX3B	NA	NA	COSN414126;OCCURENCE=1(urinary_tr	rs200308612	NA
chrX	11129475	11129475	-	GGC	UTR5	HCCS	NA	NA	NA	rs35297358	NA
chrX	13731474	13731474	A	G	UTR3	TRAPPC2	NA	NA	NA	rs5979952	NA
chrX	16859628	16859628	G	A	exonic	TXLNG	synonymous SNV	01168683:exon8:c.G930A:p.Q310Q,TXLNG:NM_018360:exon10:c.G13	NA	rs5924530	NA
chrX	18910862	18910862	T	-	ncRNA_UTR3	PHKA2	NA	NA	NA	NA	NA
chrX	20005366	20005366	G	A	ncRNA_exonic	LOC729609	NA	NA	NA	NA	NA
chrX	20145719	20145719	G	A	UTR3	EIF1AX	NA	NA	NA	rs6527969	NA
chrX	23801311	23801311	C	T	UTR5	SAT1	NA	NA	NA	rs11550721	NA
chrX	24095043	24095043	C	-	UTR3	EIF2S3	NA	NA	NA	NA	NA
chrX	24382429	24382429	C	G	exonic	SUPT20HL1	nonsynonymous SNV	SUPT20HL1:NM_001136234:exon1:c.C1552G:p.P518A	NA	rs113091397	NA
chrX	24382435	24382435	C	G	exonic	SUPT20HL1	nonsynonymous SNV	SUPT20HL1:NM_001136234:exon1:c.C1558G:p.P520A	NA	NA	NA
chrX	24577435	24577435	A	-	UTR3	PCYT1B	NA	NA	NA	rs56367400	NA
chrX	24577537	24577537	-	AGAA	UTR3	PCYT1B	NA	NA	NA	NA	NA
chrX	24577574	24577574	A	G	UTR3	PCYT1B	NA	NA	NA	rs55732431	NA
chrX	28807442	28807442	G	A	UTR5	IL1RAPL1	NA	NA	14057,COSN414056;OCCURENCE=2(uri	rs6526806	NA
chrX	34148841	34148841	A	G	exonic	FAM47A	nonsynonymous SNV	FAM47A:NM_203408:exon1:c.T1555C:p.S519P	NA	NA	B
chrX	34148844	34148844	G	C	exonic	FAM47A	nonsynonymous SNV	FAM47A:NM_203408:exon1:c.C1552G:p.R518G	NA	rs17855514	B
chrX	34148877	34148877	C	G	exonic	FAM47A	nonsynonymous SNV	FAM47A:NM_203408:exon1:c.G1519C:p.E507Q	D=COSM1138136;OCCURENCE=1(kidney	rs5973088	P
chrX	34148882	34148882	C	T	exonic	FAM47A	nonsynonymous SNV	FAM47A:NM_203408:exon1:c.G1514A:p.R505H	I488335;OCCURENCE=1(kidney),1(endo	rs5973089	B
chrX	37027745	37027745	T	C	exonic	FAM47C	nonsynonymous SNV	FAM47C:NM_001013736:exon1:c.T1262C:p.I421T	NA	NA	NA
chrX	37028026	37028026	T	C	exonic	FAM47C	nonsynonymous SNV	FAM47C:NM_001013736:exon1:c.T1543C:p.S515P	NA	rs141294322	NA
chrX	37028094	37028094	A	G	exonic	FAM47C	synonymous SNV	FAM47C:NM_001013736:exon1:c.A1611G:p.A537A	NA	NA	NA
chrX	37028645	37028645	G	C	exonic	FAM47C	nonsynonymous SNV	FAM47C:NM_001013736:exon1:c.G2162C:p.S721T	NA	NA	NA
chrX	37672462	37672462	T	-	UTR3	CYBB	NA	NA	NA	rs11338914	NA
chrX	46407207	46407207	A	G	ncRNA_exonic	ZNF674-AS1	NA	NA	NA	rs5905550	NA
chrX	46940123	46940123	A	G	UTR5	RGN	NA	NA	NA	NA	NA
chrX	47341999	47341999	-	CGTGCGCG	UTR5	ZNF41	NA	NA	NA	NA	NA
chrX	48206014	48206023	GTGTGTGTC	-	UTR3	SSX3	NA	NA	NA	rs72172699	NA
chrX	48382099	48382099	-	T	UTR5	EBP	NA	NA	NA	NA	NA
chrX	48567295	48567295	A	G	UTR3	SUV39H1	NA	NA	NA	rs3373	NA
chrX	48770831	48770831	-	A	UTR3	PIM2	NA	NA	NA	NA	NA
chrX	48770852	48770852	G	A	UTR3	PIM2	NA	NA	NA	NA	NA
chrX	48847497	48847497	T	C	exonic	GRIPAP1	synonymous SNV	M_207672:exon6:c.A324G:p.E108E,GRIPAP1:NM_020137:exon7:c.A48	NA	rs11545861	NA
chrX	48922629	48922629	G	A	exonic	CCDC120	nonsynonymous SNV	_001163323:exon7:c.G646A:p.E216K,CCDC120:NM_001271835:exon7	NA	NA	P
chrX	49107557	49107557	C	G	UTR3	FOXP3	NA	NA	NA	NA	NA
chrX	49107563	49107563	T	G	UTR3	FOXP3	NA	NA	NA	NA	NA
chrX	49113939	49113939	T	G	exonic	FOXP3	synonymous SNV	I_001114377:exon3:c.A294C:p.P98P,FOXP3:NM_014009:exon4:c.A39	NA	NA	NA
chrX	49113976	49113976	T	G	exonic	FOXP3	nonsynonymous SNV	_001114377:exon3:c.A257C:p.H86P,FOXP3:NM_014009:exon4:c.A36	NA	NA	P
chrX	51150629	51150629	A	C	intergenic	10(dist=70252),NUDT11(dist=	NA	NA	NA	NA	D
chrX	53222050	53222050	T	C	UTR3	KDM5C	NA	NA	NA	NA	NA
chrX	53279837	53279837	C	G	exonic	IQSEC2	nonsynonymous SNV	001111125:exon5:c.G1921C:p.A641P,IQSEC2:NM_015075:exon5:c.G1	NA	NA	P
chrX	54224161	54224161	A	-	UTR3	WNK3	NA	NA	NA	NA	NA
chrX	54466901	54466901	T	G	exonic	TSR2	nonsynonymous SNV	TSR2:NM_058163:exon1:c.T47G:p.V16G	NA	NA	D
chrX	54497147	54497147	T	G	exonic	FGD1	synonymous SNV	FGD1:NM_004463:exon3:c.A528C:p.P176P	NA	NA	NA

chrX	54587505	54587505	A	-	UTR3	GNL3L	NA	NA	NA	NA	NA
chrX	62857727	62857728	TG	-	UTR3	ARHGEF9	NA	NA	NA	rs10542660	NA
chrX	68061228	68061228	-	C	UTR3	EFNB1	NA	NA	NA	NA	NA
chrX	69260733	69260733	A	G	UTR3	AWAT2	NA	NA	NA	rs151003736	NA
chrX	70798076	70798076	A	G	ncRNA_UTR5	ACRC	NA	NA	NA	NA	NA
chrX	71347480	71347480	-	AGAATAGC	UTR3	RGAG4	NA	NA	NA	NA	NA
chrX	73014138	73014142	AAGGT	-	ncRNA_exonic	TSIX	NA	NA	NA	rs201154934	NA
chrX	73037456	73037456	G	A	ncRNA_exonic	TSIX	NA	NA	NA	NA	NA
chrX	73289498	73289498	G	A	ncRNA_exonic	JPX	NA	NA	NA	NA	NA
chrX	73641221	73641221	T	G	upstream	SLC16A2	NA	NA	NA	NA	NA
chrX	75648999	75648999	G	C	exonic	MAGEE1	nonsynonymous SNV	MAGEE1:NM_020932:exon1:c.G676C:p.A226P	NA	NA	B
chrX	83591866	83591866	C	T	exonic	HDX	synonymous SNV	09A:p.P503P,HDX:NM_144657:exon7:c.G1683A:p.P561P,HDX:NM_00	NA	rs5922966	NA
chrX	84343158	84343161	ATAC	-	UTR3	APOOL	NA	NA	NA	NA	NA
chrX	85118101	85118101	T	-	UTR3	CHM	NA	NA	NA	NA	NA
chrX	85225107	85225107	-	A	UTR3	CHM	NA	NA	NA	rs199510529	NA
chrX	91089881	91089881	C	G	UTR5	PCDH11X	NA	NA	NA	rs3795211	NA
chrX	1E+08	1E+08	-	A	UTR3	TMEM35	NA	NA	NA	NA	NA
chrX	1.01E+08	1.01E+08	G	C	exonic	ARMCX2	nonsynonymous SNV	NM_014782:exon5:c.C53G:p.A18G,ARMCX2:NM_177949:exon6:c.C53	NA	NA	B
chrX	1.01E+08	1.01E+08	T	C	exonic	ARMCX2	nonsynonymous SNV	:NM_014782:exon5:c.A40G:p.I14V,ARMCX2:NM_177949:exon6:c.A40	NA	rs200580160	B
chrX	1.01E+08	1.01E+08	-	T	UTR5	BEX5	NA	NA	NA	NA	NA
chrX	1.03E+08	1.03E+08	G	T	UTR3	TCEAL7	NA	NA	NA	NA	NA
chrX	1.03E+08	1.03E+08	G	C	exonic	ESX1	nonsynonymous SNV	ESX1:NM_153448:exon4:c.C1042G:p.L348V	ID=COSM226883;OCCURENCE=1(skin)	rs76090537	NA
chrX	1.03E+08	1.03E+08	C	G	exonic	ESX1	nonsynonymous SNV	ESX1:NM_153448:exon4:c.G907C:p.V303L	NA	NA	NA
chrX	1.03E+08	1.03E+08	A	C	exonic	ESX1	nonsynonymous SNV	ESX1:NM_153448:exon4:c.T889G:p.W297G	NA	NA	NA
chrX	1.06E+08	1.06E+08	-	GA	UTR3	MORC4	NA	NA	NA	NA	NA
chrX	1.1E+08	1.1E+08	GAGG	-	UTR3	CHRDL1	NA	NA	NA	rs71931043	NA
chrX	1.11E+08	1.11E+08	T	-	UTR3	DCX	NA	NA	NA	NA	NA
chrX	1.14E+08	1.14E+08	G	A	exonic	RBMXL3	nonsynonymous SNV	RBMXL3:NM_001145346:exon1:c.G1192A:p.D398N	NA	rs12399211	NA
chrX	1.16E+08	1.16E+08	T	-	UTR3	SLC6A14	NA	NA	NA	rs202199898	NA
chrX	1.19E+08	1.19E+08	G	A	exonic	CXorf56	synonymous SNV	33T:p.G111G,CXorf56:NM_001170569:exon4:c.C228T:p.G76G,CXorf56:	NA	rs5910611	NA
chrX	1.19E+08	1.19E+08	GT	-	UTR3	6-Sep	NA	NA	NA	NA	NA
chrX	1.19E+08	1.19E+08	TT	-	UTR3	6-Sep	NA	NA	NA	NA	NA
chrX	1.19E+08	1.19E+08	T	-	UTR3	ZBTB33	NA	NA	NA	NA	NA
chrX	1.2E+08	1.2E+08	GTGT	-	UTR3	MCTS1	NA	NA	NA	NA	NA
chrX	1.23E+08	1.23E+08	T	G	UTR3	XIAP	NA	NA	NA	rs28382747	NA
chrX	1.23E+08	1.23E+08	C	A	UTR3	XIAP	NA	NA	NA	rs28382751	NA
chrX	1.23E+08	1.23E+08	-	T	UTR3	XIAP	NA	NA	NA	rs58114044	NA
chrX	1.23E+08	1.23E+08	A	T	exonic	STAG2	nonsynonymous SNV	042749:exon22:c.A2124T:p.L708F,STAG2:NM_001042750:exon22:c.A2	NA	NA	B
chrX	1.31E+08	1.31E+08	GA	-	UTR3	RAP2C	NA	NA	NA	NA	NA
chrX	1.34E+08	1.34E+08	T	-	ncRNA_exonic	LINC00087	NA	NA	NA	NA	NA
chrX	1.35E+08	1.35E+08	C	A	UTR3	CT45A1	NA	NA	NA	rs1064036	NA
chrX	1.35E+08	1.35E+08	G	A	UTR3	CT45A1	NA	NA	NA	rs1064037	NA
chrX	1.35E+08	1.35E+08	GAT	-	exonic	GPR112	nonframeshift deletion	GPR112:NM_153834:exon17:c.7966_7968del:p.2656_2656del	NA	rs34255702	NA
chrX	1.36E+08	1.36E+08	G	A	UTR3	RBMX	NA	NA	NA	rs850	NA
chrX	1.4E+08	1.4E+08	G	C	UTR5	LDOC1	NA	NA	NA	rs2038375	NA
chrX	1.41E+08	1.41E+08	C	G	exonic	MAGEC1	synonymous SNV	MAGEC1:NM_005462:exon4:c.C513G:p.S171S	NA	rs75782140	NA
chrX	1.41E+08	1.41E+08	G	C	exonic	MAGEC1	nonsynonymous SNV	MAGEC1:NM_005462:exon4:c.G841C:p.V281L	NA	rs176044	NA
chrX	1.41E+08	1.41E+08	G	T	exonic	MAGEC1	nonsynonymous SNV	MAGEC1:NM_005462:exon4:c.G1130T:p.G377V	NA	rs7063168	NA
chrX	1.41E+08	1.41E+08	A	C	exonic	MAGEC1	nonsynonymous SNV	MAGEC1:NM_005462:exon4:c.A1272C:p.L424F	NA	rs56675069	NA
chrX	1.41E+08	1.41E+08	A	C	exonic	MAGEC1	nonsynonymous SNV	MAGEC1:NM_005462:exon4:c.A1358C:p.Y453S	NA	rs58117541	NA
chrX	1.45E+08	1.45E+08	A	T	UTR3	SLITRK2	NA	NA	NA	NA	NA
chrX	1.48E+08	1.48E+08	G	A	UTR3	AFF2	NA	NA	NA	rs144890448	NA
chrX	1.52E+08	1.52E+08	G	C	ncRNA_UTR5	MAGEA12	NA	NA	NA	rs73639100	NA
chrX	1.52E+08	1.52E+08	T	C	ncRNA_UTR5	MAGEA12	NA	NA	NA	rs2515675	NA
chrX	1.52E+08	1.52E+08	C	T	UTR3	PNMA5	NA	NA	NA	NA	NA
chrX	1.52E+08	1.52E+08	C	T	UTR3	PNMA5	NA	NA	NA	NA	NA
chrX	1.52E+08	1.52E+08	C	T	UTR3	PNMA5	NA	NA	NA	NA	NA
chrX	1.52E+08	1.52E+08	A	G	UTR3	PNMA5	NA	NA	NA	NA	NA
chrX	1.52E+08	1.52E+08	A	G	UTR3	PNMA5	NA	NA	NA	NA	NA
chrX	1.52E+08	1.52E+08	T	C	UTR5	PNMA5	NA	NA	NA	rs67383647	NA
chrX	1.53E+08	1.53E+08	CTCTCTCTC	-	UTR3	BGN	NA	NA	NA	rs36205859	NA
chrX	1.53E+08	1.53E+08	-	G	UTR3	ATP2B3	NA	NA	NA	rs144678509	NA
chrX	1.53E+08	1.53E+08	T	G	UTR3	ATP2B3	NA	NA	NA	rs28706355	NA
chrX	1.53E+08	1.53E+08	A	C	exonic	PLXNB3	synonymous SNV	5393:exon20:c.A3453C:p.A1151A,PLXNB3:NM_001163257:exon21:c.A	NA	NA	NA
chrX	1.53E+08	1.53E+08	C	G	exonic	AVPR2	synonymous SNV	_000054:exon2:c.C207G:p.G69G,AVPR2:NM_001146151:exon2:c.C20	NA	NA	NA
chrY	5606261	5606261	C	G	UTR3	PCDH11Y	NA	NA	NA	rs12559026	NA

chrY	5607230	5607230	G	C	UTR3	PCDH11Y	NA	NA	NA	rs2556910	NA
chrY	5607799	5607799	A	T	UTR3	PCDH11Y	NA	NA	NA	NA	NA

	Transversion										
**Chr**	**Start**	**End**	**Ref**	**Alt**	**Func.refGene**	**Gene.refGene**	**ExonicFunc.refGene**	**AAChange.refGene**	**cosmic64**	**snp137**	**PolyPhen2 Pred**
chr1	65901244	65901244	G	T	UTR3	LEPROT	NA	NA	NA	NA	NA
chr1	1.15E+08	1.15E+08	G	T	exonic	AMPD1	synonymous SNV	:exon3:c.C322A:p.R108R,AMPD1:NM_000036:	NA	NA	NA
chr1	1.21E+08	1.21E+08	G	T	UTR5	NOTCH2	NA	NA	NA	rs57122008	NA
chr1	2.37E+08	2.37E+08	G	T	UTR3	MTR	NA	NA	NA	NA	NA
chr2	46852206	46852206	G	T	UTR3	CRIPT	NA	NA	NA	NA	NA
chr2	92129614	92129614	G	T	ncRNA_exonic	ACTR3BP2	NA	NA	NA	NA	NA
chr2	1.13E+08	1.13E+08	G	T	UTR3	ZC3H6	NA	NA	NA	NA	NA
chr2	2.2E+08	2.2E+08	G	T	UTR3	FAM134A	NA	NA	NA	NA	NA
chr3	67049615	67049615	G	T	exonic	KBTBD8	nonsynonymous SNV	KBTBD8:NM_032505:exon2:c.G227T:p.R76I	NA	NA	D
chr3	1.05E+08	1.05E+08	G	T	exonic	CBLB	nonsynonymous SNV	CBLB:NM_170662:exon12:c.C1618A:p.Q540K	NA	NA	D
chr3	1.5E+08	1.5E+08	G	T	UTR3	TSC22D2	NA	NA	NA	s20149715	NA
chr3	1.7E+08	1.7E+08	G	T	UTR3	CLDN11	NA	NA	NA	NA	NA
chr4	87567	87567	G	T	UTR3	ZNF595	NA	NA	NA	NA	NA
chr4	87574	87574	G	T	UTR3	ZNF595	NA	NA	NA	NA	NA
chr4	2087366	2087366	G	T	exonic	POLN	nonsynonymous SNV	POLN:NM_181808:exon19:c.C2171A:p.A724E	NA	NA	B
chr4	1.39E+08	1.39E+08	G	T	ncRNA_UTR3	SLC7A11	NA	NA	NA	NA	NA
chr5	23521146	23521146	G	T	exonic	PRDM9	synonymous SNV	PRDM9:NM_020227:exon6:c.G366T:p.A122A	NA	NA	NA
chr5	55168106	55168106	G	T	exonic	IL31RA	nonsynonymous SNV	7:exon4:c.G281T:p.G94V,IL31RA:NM_139017:	NA	NA	B
chr5	71503811	71503811	G	T	UTR3	MAP1B	NA	NA	NA	NA	NA
chr5	80738520	80738520	G	T	exonic	SSBP2	nonsynonymous SNV	exon12:c.C711A:p.N237K,SSBP2:NM_0012567	NA	NA	D
chr5	1.41E+08	1.41E+08	G	T	UTR3	PCDHB9	NA	NA	NA	NA	NA
chr6	24775181	24775181	G	T	UTR5	GMNN	NA	NA	NA	rs2754775	NA
chr6	31324528	31324528	G	T	exonic	HLA-B	nonsynonymous SNV	HLA-B:NM_005514:exon2:c.C280A:p.Q94K	NA	rs1071817	NA
chr6	49460762	49460762	G	T	UTR3	CENPQ	NA	NA	NA	NA	NA
chr6	57398207	57398207	G	T	exonic	PRIM2	unknown	UNKNOWN	NA	rs71214816	NA
chr6	1.6E+08	1.6E+08	G	T	UTR3	WTAP	NA	NA	NA	NA	NA
chr6	1.6E+08	1.6E+08	G	T	UTR3	WTAP	NA	NA	NA	NA	NA
chr6	1.6E+08	1.6E+08	G	T	UTR3	WTAP	NA	NA	NA	NA	NA
chr6	1.6E+08	1.6E+08	G	T	UTR3	WTAP	NA	NA	NA	NA	NA
chr7	44258943	44258943	G	T	UTR3	CAMK2B	NA	NA	NA	rs74582205	NA
chr7	66774667	66774667	G	T	ncRNA_exonic	STAG3L4	NA	NA	NA	NA	NA
chr7	77558429	77558429	G	T	exonic	PHTF2	nonsynonymous SNV	F2:NM_001127357:exon10:c.G1021T:p.A341S,	NA	NA	NA
chr9	117998	117998	G	T	exonic	FOXD4	nonsynonymous SNV	FOXD4:NM_207305:exon1:c.C122A:p.A41E	ID=COSM226644;OCCURENCE=1(skin)	rs66612967	B
chr9	1.02E+08	1.02E+08	G	T	UTR3	TGFBR1	NA	NA	NA	s19954527	NA
chr9	1.16E+08	1.16E+08	G	T	UTR3	SNX30	NA	NA	NA	s11341690	NA
chr9	1.36E+08	1.36E+08	G	T	UTR5	ADAMTSL2	NA	NA	NA	NA	NA
chr10	62544601	62544601	G	T	exonic	CDK1	nonsynonymous SNV	407:exon3:c.G176T:p.R59L,CDK1:NM_001786:	NA	NA	B
chr10	71267385	71267385	G	T	UTR3	TSPAN15	NA	NA	NA	NA	NA
chr10	1.02E+08	1.02E+08	G	T	UTR3	HIF1AN	NA	NA	NA	rs75780010	NA
chr10	1.02E+08	1.02E+08	G	T	UTR3	HIF1AN	NA	NA	NA	NA	NA
chr11	1.12E+08	1.12E+08	G	T	UTR3	C11orf57	NA	NA	NA	NA	NA
chr12	5021439	5021439	G	T	exonic	KCNA1	nonsynonymous SNV	KCNA1:NM_000217:exon2:c.G895T:p.V299F	NA	NA	D
chr12	8927164	8927164	G	T	UTR3	RIMKLB	NA	NA	NA	NA	NA
chr12	11546258	11546258	G	T	exonic	PRB2	nonsynonymous SNV	PRB2:NM_006248:exon3:c.C754A:p.Q252K	NA	NA	NA
chr12	52845665	52845665	G	T	exonic	KRT6B	synonymous SNV	KRT6B:NM_005555:exon1:c.C198A:p.G66G	NA	s14111418	NA
chr12	56810470	56810470	G	T	UTR3	TIMELESS	NA	NA	NA	NA	NA
chr12	58146158	58146158	G	T	UTR5	CDK4	NA	NA	NA	NA	NA
chr12	58146161	58146161	G	T	UTR5	CDK4	NA	NA	NA	NA	NA
chr12	70214497	70214497	G	T	UTR3	RAB3IP	NA	NA	NA	rs11177881	NA
chr12	96361580	96361580	G	T	exonic	AMDHD1	stopgain SNV	AMDHD1:NM_152435:exon9:c.G1240T:p.E414X	NA	NA	NA
chr13	49852512	49852512	G	T	exonic	CDADC1	nonsynonymous SNV	exon7:c.G1077T:p.M359I,CDADC1:NM_030911	NA	NA	D
chr16	68678505	68678505	G	T	UTR5	CDH3	NA	NA	NA	NA	NA
chr17	11513845	11513845	G	T	exonic	DNAH9	nonsynonymous SNV	DNAH9:NM_001372:exon3:c.G747T:p.K249N	NA	NA	B
chr17	45214606	45214606	G	T	exonic	CDC27	nonsynonymous SNV	xon14:c.C1843A:p.H615N,CDC27:NM_001256:	NA	rs79260965	NA
chr17	77807715	77807715	G	T	UTR3	CBX4	NA	NA	NA	NA	NA

chr19	199442	199442	G	T	ncRNA_exonic	FLJ45445	NA	NA	NA	rs57399907	NA
chr19	1597572	1597572	G	T	UTR3	UQCR11	NA	NA	NA	NA	NA
chr19	43376101	43376101	G	T	exonic	PSG1	nonsynonymous SNV	E,PSG1:NM_001184826:exon3:c.C527A:p.A17	NA	NA	NA
chr19	54725798	54725798	G	T	exonic	LILRB3	nonsynonymous SNV	:exon4:c.C560A:p.T187N,LILRB3:NM_006864:	ID=COSM1158374;OCCURENCE=1(pancreas)	rs1052968	B
chr20	25754782	25754782	G	T	ncRNA_exonic	FAM182B	NA	NA	NA	NA	NA
chr20	33098244	33098244	G	T	UTR3	ITCH	NA	NA	NA	NA	NA
chr20	49367490	49367490	G	T	UTR3	PARD6B	NA	NA	NA	NA	NA
chrX	19552343	19552343	G	T	UTR3	SH3KBP1	NA	NA	NA	NA	NA
chrX	55478566	55478566	G	T	UTR5	MAGEH1	NA	NA	NA	NA	NA
chr1	22357549	22357549	G	T	ncRNA_exonic	LINC00339	NA	NA	NA	rs2473291	NA
chr1	22418256	22418256	G	T	UTR3	CDC42	NA	NA	NA	NA	NA
chr1	28087457	28087457	G	T	UTR3	FAM76A	NA	NA	NA	rs7442230	NA
chr1	1.45E+08	1.45E+08	G	T	exonic	SEC22B	unknown	UNKNOWN	NA	rs2596331	NA
chr1	1.53E+08	1.53E+08	G	T	UTR3	LCE1E	NA	NA	NA	s11447009	NA
chr1	1.71E+08	1.71E+08	G	T	ncRNA_exonic	FMO6P	NA	NA	NA	rs11812061	NA
chr3	18427924	18427924	G	T	exonic	SATB1	synonymous SNV	,SATB1:NM_001195470:exon8:c.C1386A:p.P4	NA	rs2229261	NA
chr3	75787202	75787202	G	T	exonic	ZNF717	synonymous SNV	NF717:NM_001128223:exon5:c.C1572A:p.V52	NA	rs7671373	NA
chr3	1.96E+08	1.96E+08	G	T	exonic	MUC4	nonsynonymous SNV	MUC4:NM_018406:exon2:c.C11200A:p.P3734T	NA	s20145660	NA
chr4	1.76E+08	1.76E+08	G	T	UTR5	ADAM29	NA	NA	NA	rs7689299	NA
chr4	1.84E+08	1.84E+08	G	T	exonic	WWC2	nonsynonymous SNV	WWC2:NM_024949:exon16:c.G2446T:p.V816F	NA	rs1173437	NA
chr5	1.31E+08	1.31E+08	G	T	UTR3	CDC42SE2	NA	NA	NA	NA	NA
chr6	29912087	29912087	G	T	exonic	HLA-A	nonsynonymous SNV	:exon4:c.G808T:p.A270S,HLA-A:NM_002116:e	NA	rs1059632	B
chr6	30232538	30232538	G	T	ncRNA_exonic	HLA-L	NA	NA	NA	rs28780091	NA
chr6	57512779	57512779	G	T	UTR3	PRIM2	NA	NA	NA	rs7629607	NA
chr6	1.62E+08	1.62E+08	G	T	UTR3	PARK2	NA	NA	NA	rs6812138	NA
chr8	1.45E+08	1.45E+08	G	T	UTR3	GRINA	NA	NA	NA	rs9100	NA
chr9	14993419	14993419	G	T	ncRNA_exonic	LOC389705	NA	NA	NA	rs4548277	NA
chr9	41960898	41960898	G	T	ncRNA_exonic	KGFLP2	NA	NA	NA	s20176116	NA
chr9	1.35E+08	1.35E+08	G	T	UTR3	SETX	NA	NA	NA	rs11787894	NA
chr10	1228330	1228330	G	T	UTR3	ADARB2	NA	NA	NA	rs1129226	NA
chr11	1017317	1017317	G	T	exonic	MUC6	synonymous SNV	MUC6:NM_005961:exon31:c.C5484A:p.T1828T	NA	rs3394390	NA
chr11	1018300	1018300	G	T	exonic	MUC6	nonsynonymous SNV	MUC6:NM_005961:exon31:c.C4501A:p.P1501T	NA	s11374499	NA
chr11	43940644	43940644	G	T	exonic	ALKBH3	synonymous SNV	ALKBH3:NM_139178:exon9:c.G726T:p.G242G	NA	rs1048928	NA
chr11	1.17E+08	1.17E+08	G	T	exonic	DSCAML1	nonsynonymous SNV	DSCAML1:NM_020693:exon4:c.C694A:p.H232N	ID=COSM147330;OCCURENCE=1(stomach)	rs3741280	NA
chr12	7945640	7945640	G	T	exonic	NANOG	nonsynonymous SNV	NANOG:NM_024865:exon2:c.G246T:p.K82N	NA	rs2889551	B
chr12	64538324	64538324	G	T	UTR3	SRGAP1	NA	NA	NA	rs789736	NA
chr12	1.31E+08	1.31E+08	G	T	UTR5	GPR133	NA	NA	NA	rs56090874	NA
chr15	23448270	23448270	G	T	ncRNA_exonic	GOLGA8EP	NA	NA	NA	s20023585	NA
chr16	1139446	1139446	G	T	UTR3	C1QTNF8	NA	NA	NA	rs183188	NA
chr17	37223344	37223344	G	T	ncRNA_UTR3	PLXDC1	NA	NA	NA	rs72821577	NA
chr17	39395019	39395019	G	T	UTR3	KRTAP9-8	NA	NA	NA	rs74975222	NA
chr17	46941419	46941419	G	T	UTR3	CALCOCO2	NA	NA	NA	rs6504584	NA
chr19	4153655	4153655	G	T	UTR5	CREB3L3	NA	NA	NA	NA	NA
chr19	13054781	13054781	G	T	UTR3	CALR	NA	NA	NA	rs1049481	NA
chr19	22379587	22379587	G	T	UTR5	ZNF676	NA	NA	NA	rs2360001	NA
chr19	53381429	53381429	G	T	UTR3	ZNF320	NA	NA	NA	rs2162919	NA
chr19	55401447	55401447	G	T	UTR3	FCAR	NA	NA	NA	rs58560391	NA
chr22	24314006	24314006	G	T	UTR3	DDTL	NA	NA	NA	rs1006771	NA
chr22	25024072	25024072	G	T	exonic	GGT1	nonsynonymous SNV	:exon14:c.G1361T:p.C454F,GGT1:NM_005265:	NA	s19968146	D
